# Context-independent essential regulatory interactions for apoptosis and hypertrophy in the cardiac signaling network

**DOI:** 10.1038/s41598-017-00086-y

**Published:** 2017-02-24

**Authors:** Jun Hyuk Kang, Ho-Sung Lee, Daebeom Park, Yun-Won Kang, Seon Myeong Kim, Jeong-Ryeol Gong, Kwang-Hyun Cho

**Affiliations:** 1 Graduate School of Medical Science and Engineering, Korea Advanced Institute of Science and Technology (KAIST), Daejeon, 34141 Republic of Korea; 2 Laboratory for Systems Biology and Bio-inspired Engineering, Department of Bio and Brain Engineering, Korea Advanced Institute of Science and Technology (KAIST), Daejeon, 34141 Republic of Korea

## Abstract

Apoptosis and hypertrophy of cardiomyocytes are the primary causes of heart failure and are known to be regulated by complex interactions in the underlying intracellular signaling network. Previous experimental studies were successful in identifying some key signaling components, but most of the findings were confined to particular experimental conditions corresponding to specific cellular contexts. A question then arises as to whether there might be essential regulatory interactions that prevail across diverse cellular contexts. To address this question, we have constructed a large-scale cardiac signaling network by integrating previous experimental results and developed a mathematical model using normalized ordinary differential equations. Specific cellular contexts were reflected to different kinetic parameters sampled from random distributions. Through extensive computer simulations with various parameter distributions, we revealed the five most essential context-independent regulatory interactions (between: (1) αAR and Gαq, (2) IP3 and calcium, (3) epac and CaMK, (4) JNK and NFAT, and (5) p38 and NFAT) for hypertrophy and apoptosis that were consistently found over all our perturbation analyses. These essential interactions are expected to be the most promising therapeutic targets across a broad spectrum of individual conditions of heart failure patients.

## Introduction

Heart failure is a typical complex disease that is often accompanied by apoptosis and hypertrophy of cardiomyocytes. Loss of cardiomyocytes owing to apoptosis causes a permanent reduction in myocardial function leading to the development of heart failure^1–4^. Hypertrophy of cardiomyocytes (i.e., increased length and width) impairs the coordination of myocardial contraction, predisposing individuals to heart failure and sudden death. Both apoptosis and hypertrophy are regulated by complex interactions in the intracellular signaling pathways (e.g., for apoptosis: β-adrenergic receptor (AR) pathway, mitogen associated protein kinase (MAPK) pathway, and phosphoinositide-3-kinase (PI3K)-Akt pathway; for hypertrophy: calcineurin-nuclear factor of activated T cells (CaN-NFAT) pathway, PI3K-Akt pathway, and MAPK pathways)^5^.

Many experimental studies have sought to identify key signaling components involved in the development of apoptosis or hypertrophy by investigating the following three relationships between signaling components and the phenotypes: (i) a significant association that shows a substantial difference in expression or activity of signaling components between pathologic (apoptotic and/or hypertrophic) myocardium and normal myocardium^6^; (ii) inducing a relationship where a signaling component is enhanced by treatment of a stimulant or a transfection experiment^7, 8^; and (iii) suppressing a relationship where a signaling component is inhibited by treatment of an inhibitor or a knock-out experiment^9, 10^. However, these relationships can vary depending on the experimental conditions, thus leading to their inapplicability to other cellular contexts. For instance, CREB activation, which is directly involved in apoptosis and hypertrophy, was induced by phenylephrine (an α-adrenergic agonist) in adult rat cardiomyocytes, but not in neonatal rat cardiomyocytes^11, 12^. As another example, in some experimental conditions, the key signaling component that mediates hypertrophy was found to be extracellular signal-related kinase (ERK), but in other conditions, p38 MAPK or c-Jun N-terminal kinase (JNK) fulfills this role^13, 14^.

Systems biology is a promising interdisciplinary research field that focuses on understanding complex biological processes at a system level to predict cellular behavior and to facilitate the drug development process^15–18^. Previous systems biology studies have attempted to identify key interactions between the signaling components in regulating apoptosis and/or hypertrophy from the perspective of the biological network by mathematical modeling and computer simulation^19–21^. However, most of the mathematical models did not represent diverse cellular contexts, since they analyzed experimental results obtained under specific experimental conditions by adopting pre-defined model formulations and fixed model parameters. Therefore, their findings could not be interpreted as suggesting relevance to other cellular contexts.

Thus, to the best of our knowledge, there are no results in the literature showing a key interaction whose domain of involvement in apoptosis and/or hypertrophy is not limited to a specific cellular context. In this study, we aimed to investigate and identify essential interactions that constantly maintain their involvement in regulating apoptosis and/or hypertrophy at a network level irrespective of various cellular contexts.

For this purpose, we constructed a large-scale cardiac signaling network by integrating signaling pathways related with apoptosis or hypertrophy and developed novel systems biological methods that can identify the essential interactions in the network by evaluating three relationships (i.e., significant association; inducing relationship; and suppressing relationship). In the employed methods, diverse cellular contexts can be represented by unfixed model parameters sampled from random distributions. In addition, perturbation of those sampling distributions enables the investigation of inducing and suppressing relationships between the parameter and the phenotypes.

Our analyses revealed that five interactions (αAR–Gαq; IP3–calcium; epac–CaMK; JNK–NFAT; and p38–NFAT) were consistently and predominantly involved in inducing or suppressing hypertrophy and/or apoptosis in any model formulations and model parameters. Furthermore, among them, the interaction (IP3–calcium) was identified to act as a primary mediator of apoptosis by inducing it synergistically in combination with other interactions.

## Results

### Constructing a cardiac signaling network by integration of experimental results

**Figure 1:**
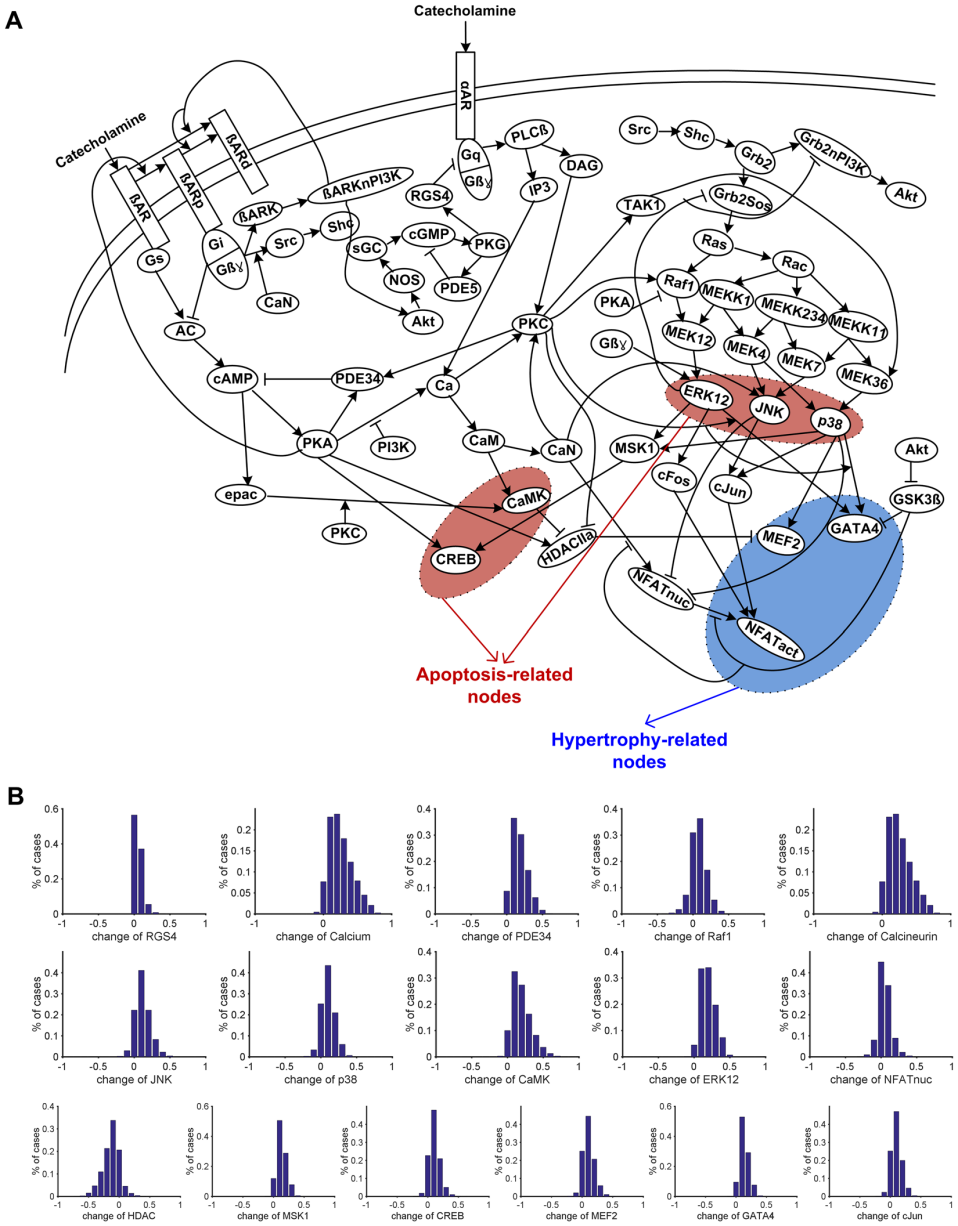
Cardiac signaling network. (**A**) The network consists of 59 signaling components. (**B**) The model is verified by observing the distribution of the activity of 17 signaling components after the treatment of catecholamine.

**Figure 2:**
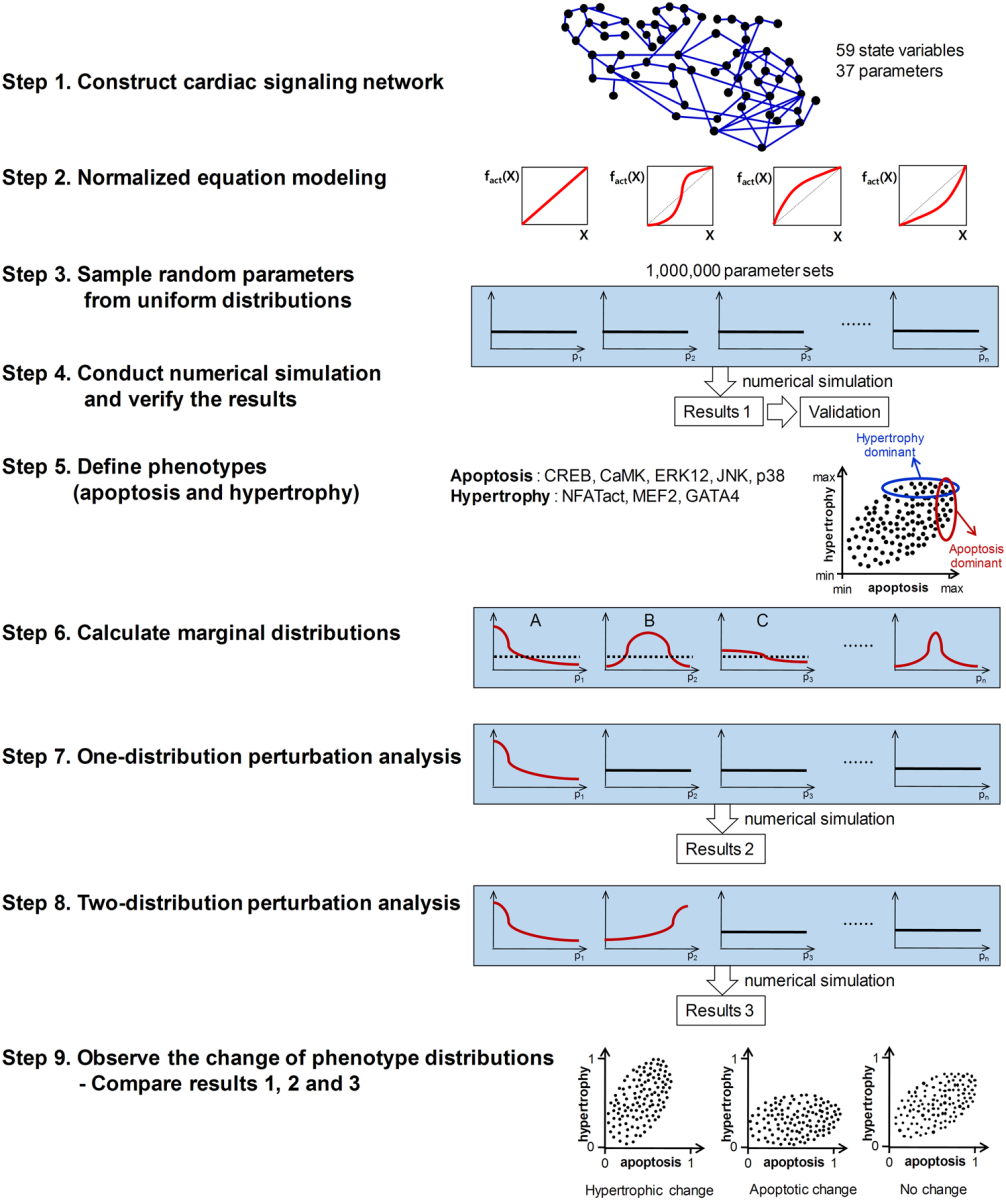
Analysis workflows for the cardiac signaling network. **Step 1**. Construct a large-scale cardiac signaling network; **Step 2**. Formulate the network as a mathematical model using the normalized equation modeling method; **Step 3**. Generate one million random parameter sets from standard uniform distributions; **Step 4**. Conduct the numerical simulation using ode15s function in MATLAB and verify the model by comparing the simulation results with experimental data; **Step 5**. Define apoptotic and hypertrophic phenotypes; **Step 6**. Calculate marginal distributions of parameters that are associated with apoptotic or hypertrophic phenotypes (plot A and plot B represent non-uniform marginal distributions and plot C represents a near-uniform marginal distribution; the lines colored in red denote a marginal distribution and dotted lines denote a uniform distribution); **Step 7**. Perform one distribution perturbation analyses; **Step 8**. Perform two distribution perturbation analyses; **Step 9**. Observe the change of phenotype distributions by distribution perturbation analyses.

We collected information for a cardiac signaling network from 134 relevant papers and manually summarized a total of 5,463 experimental results into five categories: (1) experimental conditions (e.g., the type of cell lines, the presence of genetic manipulation such as transfection or knock-out); (2) input (e.g., ligand to the receptor); (3) output (e.g., measured molecules); (4) effect (e.g., activation or inhibition, level of activation or inhibition); and (5) time (e.g., the time of peak effect). The entire data are provided in Supplementary Data Set S1. Based on the data, we constructed a large-scale cardiac signaling network that includes the links repeatedly validated by independent biochemical experiments (Fig. 1A, step 1 in Fig. 2). The constructed network includes apoptotic signaling pathways (e.g., β-AR signaling pathway) and hypertrophic signaling pathways (e.g., CaN-NFAT signaling pathway, PI3K-Akt signaling pathway, and MAPK signaling pathway) and crosstalk links between the apoptotic and hypertrophic signaling pathways. Detailed information of each node and each link is provided in Supplementary Data Set S2 (hereinafter, each node will be denoted by an abbreviation as defined in Supplementary Data Set S2).

### Formulating the constructed network as a normalized equation model

Normalized equation modeling was adopted to formulate the cardiac signaling network as a mathematical model. This modeling allows easy comparison of the simulation results under various conditions by standardizing the values of state variables and parameters between 0 and 1. The model has 59 state variables and 37 parameters (all equations for the model are provided in Supplementary Table S1 and the representation of regulatory processes by parameters is provided in Supplementary Table S2; hereinafter, each parameter will be denoted by ‘pm’ with n, one of the natural numbers from 1 to 37, as defined in Supplementary Tables S2–3).

To represent diverse cellular contexts, four types of response functions (i.e., Linear (Lin), Hill, Saturating (Sat), and Accelerating (Acc)) were used and one million parameter sets (each parameter set has 37 parameter values) were generated by random sampling from the standard uniform distribution between 0 and 1, resulting in four million models in total (step 2 and step 3 in Fig. 2) (see Methods for details). Numerical simulations were then conducted in the absence of catecholamines, important biochemical stimuli for the regulation of cellular functions in cardiomyocytes^22^, for each parameter set. After stabilization, the simulation was carried out in the presence of catecholamine stimulation (step 4 in Fig. 2).

To verify whether the constructed models are consistent with the published experimental results, we investigated the changes in the activity of each signaling component under the catecholamine treatment (the results obtained with the function type Lin is shown in Fig. 1B). Previous studies have shown that the catecholamine treatment induces an increase of 15 signaling components (i.e., RGS4, Calcium, PDE34, Raf1, CaN, JNK, p38, CaMK, ERK12, NFAT, MSK1, CREB, MEF2, GATA4, and cJun) and a decrease of one signaling component (i.e., HDAC) that are included in the model (Supplementary Table S4). From extensive simulation analyses, we found that the mathematical model successfully captures the qualitative features of known biological activities of 14 signaling components in response to catecholamine stimulation for more than 80% of 1 million randomly sampled parameter sets when Lin, Sat, Acc, or a combination of four different response function types was applied. In the case when function type Hill was applied, more than half of the parameter sets successfully reproduced the previous known activities of 15 signaling components (Supplementary Table S5). Thus, we can confirm that the cardiac signaling network model can reasonably reproduce previous experimental results irrespective of the specific biochemical response functions (i.e., Lin, Acc, Sat, and Hill) employed to the mathematical model.

In addition, changes in the distributions of the activity of apoptosis and hypertrophy induced by catecholamine were investigated. Apoptosis and hypertrophy were determined as following algebraic eqs (i) and (ii) that are derived based on the activities of pro-apoptotic proteins (CaMK, JNK, and p38), anti-apoptotic proteins (CREB and ERK12), and hypertrophy–related proteins (NFATact, MEF2, and GATA4) (step 5 in Fig. 2, see also Supplementary Table S1).

i $$Apoptosis=\frac{[CaMK]+[JNK]+[p38]-[CREB]-[ERK12]}{5}$$

ii $$Hypertrophy=\frac{[NFATact]+[MEF2]+[GATA4]}{5}$$

Distributions of these two phenotypes were used as a control to compare them with the phenotype distributions obtained by subsequent distribution perturbation analyses.

### Evaluating associations between interactions and phenotypes by observation of marginal distributions of parameters

**Figure 3:**
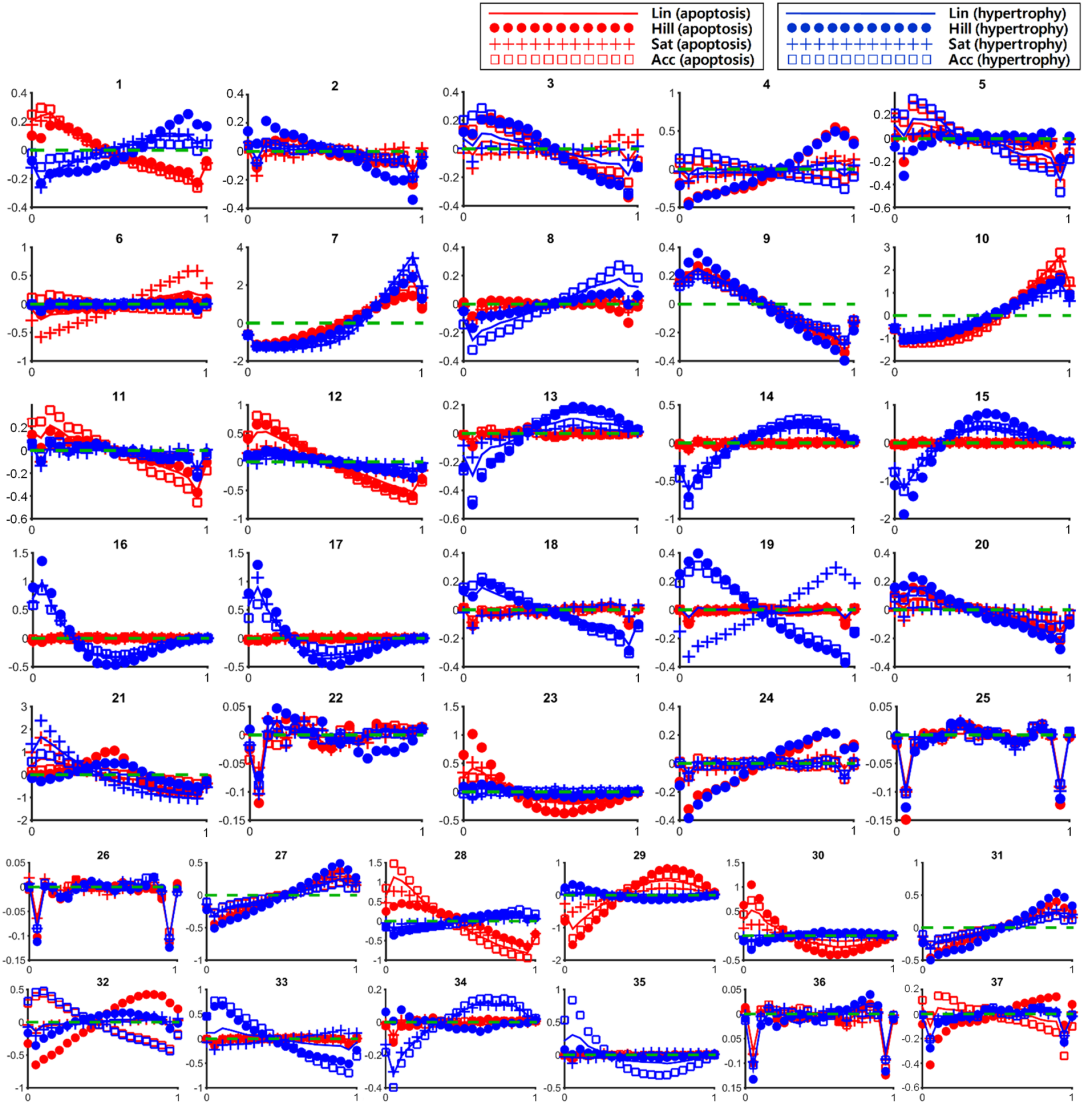
Marginal distribution for apoptosis (red) and hypertrophy (blue). A green dotted line marks the same level of density between the marginal distribution and the uniform distribution. In the area above the green line, the density is higher than that of the uniform distribution, whereas in the area below the green line, the density is lower than that of the uniform distribution.

The control phenotype distributions were used to investigate the parameters that are closely associated with apoptosis and hypertrophy (step 6 in Fig. 2). We used ‘top 10%’ as a threshold value to determine the parameter sets that highly induce apoptosis or hypertrophy in the mathematical model. The distributions of parameters that resulted in top 10% of apoptosis and top 10% of hypertrophy were named marginal distributions for apoptosis and marginal distributions for hypertrophy, respectively. We calculated a marginal distribution and compared it with the uniform distributions of each parameter (Fig. 3). The association of a parameter with a phenotype was explored by observation of the shape of its marginal distribution for the phenotype. If a marginal distribution of parameter p1 for apoptosis displays a high density in the low range of p1, a low p1 can be interpreted as indicating its association with apoptosis (step 6, plot A in Fig. 2). Accordingly, if a marginal distribution of parameter p2 for apoptosis displays a high density in the middle range of p2, neither high nor low p2 can be regarded as being associated with apoptosis (step 6, plot B in Fig. 2).

However, the small difference between the marginal distribution of a parameter from the uniform distribution (near-uniform distribution) suggests the absence of association of the parameter with the phenotypes (step 6, plot C in Fig. 2). For instance, pm22 (Ras/(PKC, PKA) → Raf1), pm25 (MEKK1/MEKK234 → MEK4), pm26 (MEKK234/MEKK11 → cJun), and pm36 (JNK/p38 → MSK1) may not be associated with the regulation of apoptosis or hypertrophy since the difference between their marginal distribution and uniform distribution was relatively very small when compared with the cases of other parameters (panel 22, 25, 26, and 36 in Fig. 3).

For some parameter distributions, different distribution patterns were observed depending on the applied function types (incoherent distributions), whereas, for the others, similar distribution patterns were observed regardless of the applied function types (coherent distributions). Incoherent distributions indicate an inconsistent association with the phenotype. Therefore, only those parameters that exhibit non-uniform and coherent marginal distributions were considered to be closely associated with the phenotypes. The characteristics of the marginal distributions for apoptosis and hypertrophy of 37 parameters are summarized in Table 1.

**Table 1:** Characteristics of marginal distributions of 37 parameters.

		Apoptosis	Hypertrophy
Coherent and non-uniform	Low	pm1: maximal degree of β-AR phosphorylation pm9^a^: (PKA & PI3K)/PKA → activated PKA for Ca pm11^a^: (epac& PKC)/epac → activated epac for CaMK pm12^a,b,c^: (CaM& activated epac)/CaM → CaMK pm23^c^: PKC/(Ras, PKA) → Raf1 pm28^b,c^: Gbg/MEK12 → ERK12 pm30: MEK4/(CaN, MEK7) → JNK	pm9^a^: (PKA & PI3K)/PKA → activated PKA for Ca pm16^a,b,c^: JNK/(CaN, p38) → NFATnuc pm17^a,b,c^: p38/(CaN, JNK) → NFATnuc pm29^a^: CaN/(MEK4, MEK7) → JNK
	High	pm7^a,b,c^: αAR/RGS4 → Gq pm10^a,b,c^: IP3/activated PKA → Ca pm27^a^: TAK1/MEKK11 → MEK36 pm29^a^: CaN/(MEK4, MEK7) → JNK pm31^a^: MEK36/MEK4 → p38	pm1: maximal degree of β-AR phosphorylation pm7^a,b,c^: αAR/RGS4 → Gq pm8: (DAG &Ca)/(DAG &Ca&CaN) → PKC pm10^a,b,c^: IP3/activated PKA → Ca pm13^a^: CaMK/(PKC, PKA) → HDAC pm14^a^: PKC/(CaMK, PKA) → HDAC pm15^a,b^: CaN/(JNK, p38) → NFATnuc pm27^a^: TAK1/MEKK11 → MEK36 pm28^b,c^: Gbg/MEK12 → ERK12 pm30: MEK4/(CaN, MEK7) → JNK pm31^a^: MEK36/MEK4 → p38
Incoherent or near-uniform		pm2, pm3, pm4, pm5, pm6, pm8, pm13, pm14, pm15, pm16, pm17, pm18, pm19, pm20, pm21^b^, pm22, pm24, pm25, pm26, pm32, pm34, pm35, pm36, pm37	pm2, pm3, pm4, pm5, pm6, pm11, pm12, pm18, pm19, pm20, pm21^b^, pm22, pm23, pm24, pm25,pm 26, pm32, pm33, pm34, pm35^c^, pm36, pm37

This table outlines the biological role of parameters that showed coherent and non-uniform marginal distributions. ^a^The marginal distributions indicate a non-inverse association of the parameters with apoptosis and/or hypertrophy. ^b^The marginal distributions indicate an inducing relationship of the parameters with apoptosis and/or hypertrophy. ^c^The marginal distributions indicate a suppressing relationship of the parameters with apoptosis and/or hypertrophy.

### Identifying essential regulatory interactions

To identify essential regulatory interactions that maintain a constant role as a regulator of apoptosis and/or hypertrophy irrespective of model formulations and model parameters, we established the following three criteria: criterion 1–a non-inverse association between a parameter and a phenotype; criterion 2–an inducing relationship between them; and criterion 3–a suppressing relationship between them. The interactions that satisfy all these criteria were determined as essential regulatory interactions.

### Criterion 1: Investigating a non-inverse association

Eighteen parameters provided in Table 1 show non-uniform, coherent marginal distributions. Among them, three parameters (pm1, pm28, and pm30) are significantly associated with both apoptosis and hypertrophy; however, the marginal distribution for apoptosis showed a pattern resembling a reversed shape of the marginal distribution for hypertrophy. For instance, when ERK12 is activated by Gbg and MEK12 (pm28), in comparison with the influence of MEK12 on ERK12, the relatively small influence of Gbg on ERK12 is associated with apoptosis and its relatively large influence is associated with hypertrophy. In this case, the weakening or strengthening of the influence will only result in switching between the phenotypes (i.e., apoptosis to hypertrophy or hypertrophy to apoptosis) rather than eliminating the phenotype completely, thus showing an inverse association with apoptosis and hypertrophy. This observation suggests unsuitability of these three interactions as a therapeutic target for apoptosis and/or hypertrophy.

The remaining 15 parameters have a non-inverse association with apoptosis and/or hypertrophy: nine parameters (pm8, pm11, pm12, pm13, pm14, pm15, pm 16, pm17, and pm23) with either apoptosis or hypertrophy; six parameters (pm7, pm9, pm10, pm27, pm29 and pm31) with both apoptosis and hypertrophy (Supplementary Fig. S1 and Table 1).

### Criterion 2: Investigating an inducing relationship by one-distribution perturbation analyses

The significant association between a parameter and a phenotype does not indicate that the phenotype is induced by the specific distribution of the parameter. The parameters may be distributed coincidentally in such a way that they appear to be related with the phenotypes in the course of complex interactions between network components. To investigate the inducing relationship, we conducted a one-distribution perturbation analysis (step 7 in Fig. 2). One parameter out of 37 was sampled from its marginal distribution and the remaining 36 parameters were sampled from standard uniform distributions until one million random parameter sets were generated. This sampling process was repeated to allow every possible parameter (i.e., 37 parameters) to be sampled in combination with each phenotype (i.e., apoptosis and hypertrophy) and each function type (i.e., Lin, Hill, Sat, and Acc) to obtain a total of 296 combinations. A numerical simulation was performed for each parameter set, and the distribution of the obtained phenotypes was compared with the control phenotype distribution. The above mathematical analysis was all repeated for 10 times using different random seeds of one million parameter sets while following the same procedure. The differences between the two distributions provide a basis for determining the inducing relationship.

**Figure 4:**
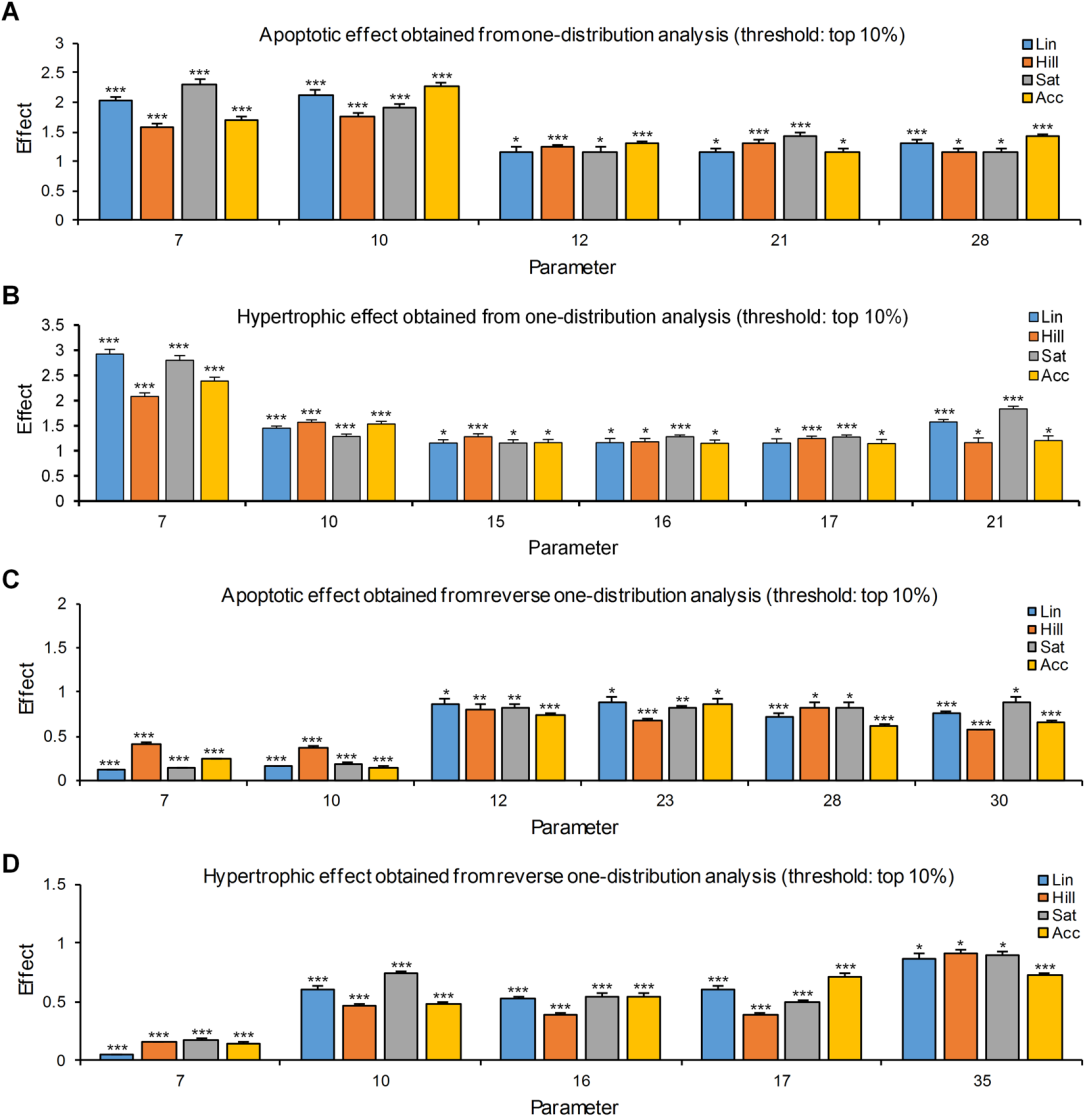
Results of one-distribution perturbation analysis for apoptosis and hypertrophy. The effect is represented as the ratio between the degree of appearance of phenotypes in one-distribution perturbation analysis and that in the control distributions. Parameters of which the marginal distributions significantly (p < 0.05) changed apoptosis or hypertrophy in all response function types are shown. Data represent means + S.E.M of 10 repetitive simulation results using different seeds of parameter sets. *p < 0.05; **p < 0.01; ***p < 0.001; Student’s *t* test.

We defined the inducing relationship as follows: the phenotype should be observed more frequently in all function types with statistical significance (p < 0.05) in comparison with respective control phenotypes. Eight parameters met the definition: three parameters (pm7, pm10 and pm21) with both apoptosis and hypertrophy; two parameters (pm12 and pm28) with apoptosis; and three parameters (pm15, pm16 and pm17) with hypertrophy (Fig. 4, Supplementary Fig. S1 and Tables 1 and 2). Among 15 parameters that have a non-inverse association, six parameters (i.e. pm7: αAR/RGS4 → Gq, pm10: IP3/activated PKA → Ca, pm12: (CaM& activated epac)/CaM → CaMK, pm15: CaN/(JNK, p38) → NFATnuc, pm16: JNK/(CaN, p38) → NFATnuc, and pm17: p38/(CaN, JNK) → NFATnuc) showed an inducing relationship. These results were consistent even when different threshold values (i.e. top 5% or 20%) for determining the marginal distributions were used or when a combination of four different response function types were applied to the model during the mathematical analysis (Supplementary Table S6).

**Table 2:** Result of one-distribution or reverse one-distribution perturbation analysis for apoptosis or hypertrophy.

	Lin		Hill		Sat		Acc	
	Effect	p-value	Effect	p-value	Effect	p-value	Effect	p-value
**One-distribution perturbation analysis**								
Apoptosis								
pm7	2.034	<0.001	1.582	<0.001	2.310	<0.001	1.690	<0.001
pm10	2.130	<0.001	1.769	<0.001	1.906	<0.001	2.271	<0.001
pm12	1.123	0.046	1.206	<0.001	1.122	0.045	1.282	<0.001
pm21	1.147	0.022	1.315	<0.001	1.428	<0.001	1.151	0.024
pm28	1.319	<0.001	1.137	0.042	1.143	0.027	1.422	<0.001
Hypertrophy								
pm7	2.920	<0.001	2.081	<0.001	2.798	<0.001	2.387	<0.001
pm10	1.448	<0.001	1.565	<0.001	1.286	<0.001	1.532	<0.001
pm15	1.126	0.033	1.260	<0.001	1.125	0.033	1.144	0.021
pm16	1.151	0.020	1.171	0.011	1.207	<0.001	1.101	0.043
pm17	1.156	0.021	1.185	<0.001	1.221	<0.001	1.142	0.022
pm21	1.574	<0.001	1.129	0.036	1.831	<0.001	1.183	0.012
**Reverse one-distribution perturbation analysis**								
Apoptosis								
pm7	0.116	<0.001	0.412	<0.001	0.139	<0.001	0.242	<0.001
pm10	0.158	<0.001	0.376	<0.001	0.180	<0.001	0.150	<0.001
pm12	0.854	0.010	0.806	0.006	0.814	0.008	0.746	<0.001
pm23	0.876	0.032	0.670	<0.001	0.817	0.009	0.837	0.011
pm28	0.728	<0.001	0.811	0.011	0.822	0.013	0.605	<0.001
pm30	0.749	<0.001	0.565	<0.001	0.886	0.038	0.661	<0.001
Hypertrophy								
pm7	0.048	<0.001	0.156	<0.001	0.178	<0.001	0.149	<0.001
pm10	0.610	<0.001	0.466	<0.001	0.737	<0.001	0.477	<0.001
pm16	0.526	<0.001	0.385	<0.001	0.543	<0.001	0.551	<0.001
pm17	0.613	<0.001	0.395	<0.001	0.500	<0.001	0.714	<0.001
pm35	0.872	0.016	0.918	0.041	0.898	0.031	0.726	<0.001

Results of one-distribution or reverse one-perturbation analysis for apoptosis and hypertrophy. The threshold for determining the marginal distribution is set to top 10%. The effect is represented as the ratio between the degree of appearance of phenotypes in the one-distribution perturbation analysis and that in the control distributions. Parameters of which the marginal distributions significantly (p<0.05) increased (in one-distribution perturbation analysis) or decreased (in reverse one-distribution perturbation analysis) apoptosis/hypertrophy in all response function types are shown. The mathematical analysis was all repeated for 10 times using different random seeds of 1 million parameter sets for each case. P-values were determined by comparison with the control distributions using Student’s *t* test. See Supplementary Data Sets for full data.

### Criterion 3: Investigating a suppressing relationship by reverse one-distribution perturbation analyses

An inducing relationship does not support sufficiently that the phenotypes will be suppressed by regulating the parameters. To investigate the suppressing relationship, we conducted reverse one-distribution perturbation analyses. One parameter out of 37 was sampled from its reverse marginal distribution (bilaterally symmetrical to the marginal distribution). If in a marginal distribution of parameter p1, a high density is observed in the low range of p1, a high density should be observed in the high range of p1 in its reverse marginal distribution. The remaining processes are same as employed for one-distribution perturbation analyses.

The suppressing relationship was defined as follows: the phenotype should be observed less frequently in all function types with statistical significance (p < 0.05) in comparison with respective control phenotypes. Nine parameters met the definition: two parameters (pm7 and pm10) with both apoptosis and hypertrophy; four parameters (pm12, pm23, pm28, and pm30) with apoptosis; and three parameters (pm16, pm17, and pm35) with hypertrophy (Figs 4 and S1, Tables 1 and 2). Among six parameters that showed both a non-inverse association and an inducing relationship, five parameters (i.e. pm7: αAR/RGS4 → Gq, pm10: IP3/activated PKA → Ca, pm12: (CaM& activated epac)/CaM → CaMK, pm16: JNK/(CaN, p38) → NFATnuc, and pm17: p38/(CaN, JNK) → NFATnuc) have a suppressing relationship that implicates promising therapeutic targets. The results were also consistent even when different threshold values (i.e. top 5% or 20%) for determining the marginal distributions were used or when a combination of four different response function types were applied to the model during the mathematical analysis (Supplementary Table S6).

To validate the predictions made by the mathematical analysis, we used a pharmacological inhibitor of epac or CaMKII to suppress the epac-CaMK interaction, which is found to be important in the regulation of apoptosis from the distribution perturbation analysis, and monitored the cellular response (Supplementary Fig. S2). Note that the marginal distribution of epac-CaMK interaction for apoptosis tends to have higher values and therefore inhibition of this interaction would reduce cardiomyocytes apoptosis. Inhibition of epac or CaMKII by treating HL-1 cells (a mouse cardiomyocyte cell line) or H9C2 cells (a rat cardiac cell line) with the chemical ESI-09 (an epac inhibitor) or KN93 (a CaMKII inhibitor) suppressed CaMKII activity in the presence of isoproterenol (ISO, a synthetic catecholamine that stimulates beta-adrenergic receptor signaling) (Supplementary Fig. S2A). Moreover, treatment with ESI-09 or KN93 significantly reduced the cell death but increased viability in ISO-treated cardiac cells (Supplementary Fig. S2B,C). These results suggest that the epac-CaMK interaction plays a crucial role in the regulation of apoptosis of cardiomyocytes as predicted by the mathematical analysis.

### Identifying synergistic effects by two-distribution perturbation analysis

Although a marginal distribution of a certain parameter has no inducing relationship with the phenotypes, it may acquire an inducing relationship if another marginal distribution is applied simultaneously. To investigate such a synergistic effect, we conducted two-distribution perturbation analyses (step 8 in Fig. 2). Two out of 37 parameters were selected to be sampled from their marginal distributions and the remaining 35 parameters were sampled from standard uniform distributions until 1 million random parameter sets were generated (see Methods for details). This sampling process was repeated to obtain all possible pairs of two parameters (i.e., 666), for each phenotype (i.e., apoptosis and hypertrophy), and for each function type (i.e., Lin, Hill, Sat, and Acc) (total 5,328 combinations). After performing the numerical simulation for each parameter set, the distribution of the phenotypes was compared with that obtained from the one-distribution perturbation analysis to determine the synergistic effects (step 9 in Fig. 2, see Methods for details). The above mathematical analysis was all repeated for 10 times using different random seeds of 1, 10, or 100 million parameter sets while following the same procedure.

The analysis results for apoptosis showed a synergistic effect with significance (p < 0.05) in 24 parameter combinations (Table 3). pm10 appeared 22 times out of 24 (91.7%) combinations, implying that the regulation of calcium by IP3 and PKA is overwhelmingly involved in exerting a synergistic effect for inducing apoptosis. These results were consistent when four different response functions were applied in a combined manner (Supplementary Table S7).

**Table 3:** Result of two-distribution perturbation analysis for apoptosis or hypertrophy.

Pair of perturbed parameter distributions	1 million parameter sets		10 million parameter sets		100 million parameter sets	
	Synergistic effect	p-value	Synergistic effect	p-value	Synergistic effect	p-value
Apoptosis						
pm1-pm10	0.078	0.012	0.082	0.012	0.076	0.018
pm2-pm10	0.15	0.009	0.132	0.009	0.139	0.009
pm3-pm10	0.155	0.002	0.17	0.002	0.156	0.004
pm6-pm10	0.161	0.004	0.167	0.002	0.17	0.005
pm7-pm10	0.528	<0.001	0.514	<0.001	0.527	<0.001
pm7-pm30	0.086	0.015	0.085	0.015	0.091	0.012
pm8-pm10	0.102	0.008	0.099	0.01	0.089	0.014
pm9-pm10	0.089	0.017	0.088	0.013	0.1	0.016
pm10-pm11	0.096	0.014	0.106	0.007	0.107	0.007
pm10-pm13	0.08	0.016	0.086	0.018	0.093	0.013
pm10-pm14	0.081	0.018	0.08	0.014	0.069	0.03
pm10-pm15	0.067	0.03	0.063	0.028	0.065	0.040
pm10-pm17	0.092	0.014	0.087	0.019	0.079	0.011
pm10-pm19	0.083	0.013	0.082	0.017	0.085	0.011
pm10-pm20	0.107	0.009	0.108	0.008	0.115	0.005
pm10-pm26	0.104	0.009	0.099	0.018	0.105	0.008
pm10-pm30	0.076	0.013	0.119	0.008	0.094	0.018
pm10-pm32	0.074	0.017	0.085	0.011	0.08	0.02
pm10-pm33	0.079	0.017	0.089	0.015	0.074	0.015
pm10-pm34	0.108	0.009	0.108	0.01	0.096	0.011
pm10-pm35	0.089	0.019	0.084	0.01	0.085	0.016
pm10-pm36	0.096	0.013	0.083	0.016	0.098	0.012
pm10-pm37	0.1	0.008	0.1	0.02	0.086	0.019
pm22-pm23	0.145	0.009	0.138	0.008	0.143	0.009
Hypertrophy						
pm7-pm10	0.354	<0.001	0.355	<0.001	0.359	<0.001
pm7-pm13	0.132	0.007	0.134	0.01	0.137	0.006
pm7-pm14	0.169	0.003	0.166	0.002	0.172	0.002
pm7-pm21	0.414	<0.001	0.418	<0.001	0.417	<0.001
pm10-pm14	0.124	0.008	0.115	0.008	0.131	0.007
pm16-pm17	0.155	0.002	0.149	0.006	0.143	0.007

Results of two-distribution perturbation analysis for apoptosis and hypertrophy. The synergistic effect was calculated as the difference between the effect of simultaneous perturbation of marginal distributions of two parameters on the phenotype and the sum of that obtained from perturbing either individual marginal distribution. The data represent average synergistic effect of simulation analysis using each response function separately. Higher values indicate stronger synergistic effect. Parameter pairs exhibiting synergistic effect for apoptosis or hypertrophy with significance (p < 0.05) are shown. The mathematical analysis was all repeated for 10 times using different random seeds of 1, 10, or 100 million parameter sets for each case. p-values were determined using Student’s *t* test. See Supplementary Data Sets for full data.

In the analysis results for hypertrophy, a synergistic effect with significance (p < 0.05) was observed in seven parameter combinations (Table 3). pm7 and pm14 appeared in six combinations, indicating a close involvement of αAR/RGS4 → Gq and IP3/activated PKA → Ca in inducing hypertrophy synergistically. In particular, although a marginal distribution of pm14 did not show an inducing relationship with hypertrophy in one distribution perturbation analysis, it acquired the relationship when the marginal distribution of pm7 or pm10 was applied simultaneously. These results were also consistent when four different response functions were applied in a combined manner (Supplementary Table S7).

## Discussion

Molecular targets for the treatment of apoptosis and hypertrophy have been suggested by experimental studies (e.g., β-AR, AngII-R1, PDE5, PDE3, HDAC, MEK12, MEK4, MEK36, and TAK1). However, since these suggestions were based on biochemical experiments performed under specific experimental conditions, their validity in other various cellular contexts is questionable. Furthermore, a plethora of possible combinations of experimental conditions makes it inconceivable to conduct experiments under each combination.

In this study, we identified five essential regulatory interactions: two interactions (i.e., αAR/RGS → Gq (pm7) and IP3/PKA → calcium (pm10)) for both apoptosis and hypertrophy; one interaction (i.e., pm12: (CaM& activated epac)/CaM → CaMK) for apoptosis; and two interactions (i.e., pm16: JNK/(CaN, p38) → NFATnuc and pm17: p38/(CaN, JNK) → NFATnuc) for hypertrophy (Fig. S1). Marginal distributions of these interactions significantly increased apoptosis or hypertrophy over numerous randomly sampled parameter sets (representing the diversity of experimental conditions or cellular contexts), whereas their reverse marginal distributions decreased it. This indicates that the five regulatory interactions may be responsible for inducing apoptosis or hypertrophy in a context-independent manner. Thus, these essential interactions may have the potential to be promising therapeutic targets that can overcome drug resistance caused by heterogeneous physiological environment and therefore can be effective across a broad spectrum of heart failure patients.

A non-inverse association of a phenotype with parameters does not necessarily imply an inducing relationship between them. For instance, the marginal distribution of pm29 (CaN/(MEK4, MEK7) → JNK) had no such relationship with apoptosis although they exhibited a non-inverse association. In order to ascertain an inducing relationship, we performed a distribution perturbation analysis to verify that a phenotype is generated more often in marginal distributions than in uniform distributions.

Because a suppressing relationship is not always derived from an inducing relationship, a reverse distribution perturbation analysis should be performed to verify the suppressing relationship. For instance, the marginal distribution of pm15 (CaN/(JNK, p38) → NFATnuc), despite its inducing relationship with hypertrophy, had no suppressing relationship with the phenotype. This was corroborated by the result that a weak effect of CaN on NFATnuc can induce hypertrophy, whereas its effect, irrespective of the intensity, cannot suppress hypertrophy. In contrast, pm16 (JNK/(CaN, p38) → NFATnuc) and pm17 (p38/(JNK, p38) → NFATnuc) showed both inducing and suppressing relationships, suggesting them a more efficacious target for hypertrophy than pm15.

Although a non-inverse association does not necessarily indicate an inducing relationship and an inducing relationship does not always lead to a suppressing relationship, the three relationships overlap to a large extent. Eighty percent of parameters that had an inducing relationship with apoptosis and 71% of parameters with hypertrophy showed a non-inverse association. Sixty-seven percent of parameters that had a suppressing relationship with apoptosis and 80% of parameters with hypertrophy showed an inducing relationship between them.

To date, several studies have investigated the cardiac signaling pathway through systems biological approaches^19, 23^. Despite their usefulness in analyzing cardiac signaling network, key interactions that consistently maintain a regulatory role under different cellular contexts have remained unexplored since most previous studies focused on the interpretation of experimental results under a specific cellular context.

To investigate this untrodden area of research, we developed a method using normalized equation modeling and distribution perturbation analyses. In the normalized equation model, the required number of parameters is smaller than the number required in conventional modeling. Since a parameter in the normalized equation modeling directly denotes a link in the network, the results are easily interpreted. In addition, the use of four function types (i.e., Lin, Hill, Sat, and Acc) with random parameter sets for the formulation of the models leads to a result that is not biased by a particular model setting.

A model ensemble instead of a one-parameter-specified model was established for a network topology by allowing a parameter in the normalized equation to take multiple values sampled from a distribution. Since these results in a sensitivity analysis being inapplicable to the normalized equation model, we developed a distribution perturbation analysis in which one or two selected parameters are sampled from marginal distributions related with a phenotype and remaining parameters are sampled from uniform distributions. Based on the results of the distribution perturbation analysis, we can determine an inducing relationship between the parameters and phenotype distributions.

Furthermore, the two-distribution perturbation analysis enabled the exploration of a synergistic effect from the network. Calcium regulation is known to play an important role in the development of apoptosis because calcium acts upon apoptosis-related pathways. Our analysis demonstrated that an interaction between the regulation of calcium and that of other molecules generates a synergistic effect in the process of apoptosis of cardiomyocytes, providing a fresh perspective on the underlying mechanism of apoptosis.

In summary, we identified essential interactions involved in the regulation of apoptosis and hypertrophy of cardiomyocytes through a novel computational method based on systems biology. Our analyses will help to estimate the potential usefulness of therapeutic targets suggested in experimental research. The applicability of the methods can be extended to analyses of other general large-scale networks.

## Methods

### Normalized equation modeling

**Figure 5:**
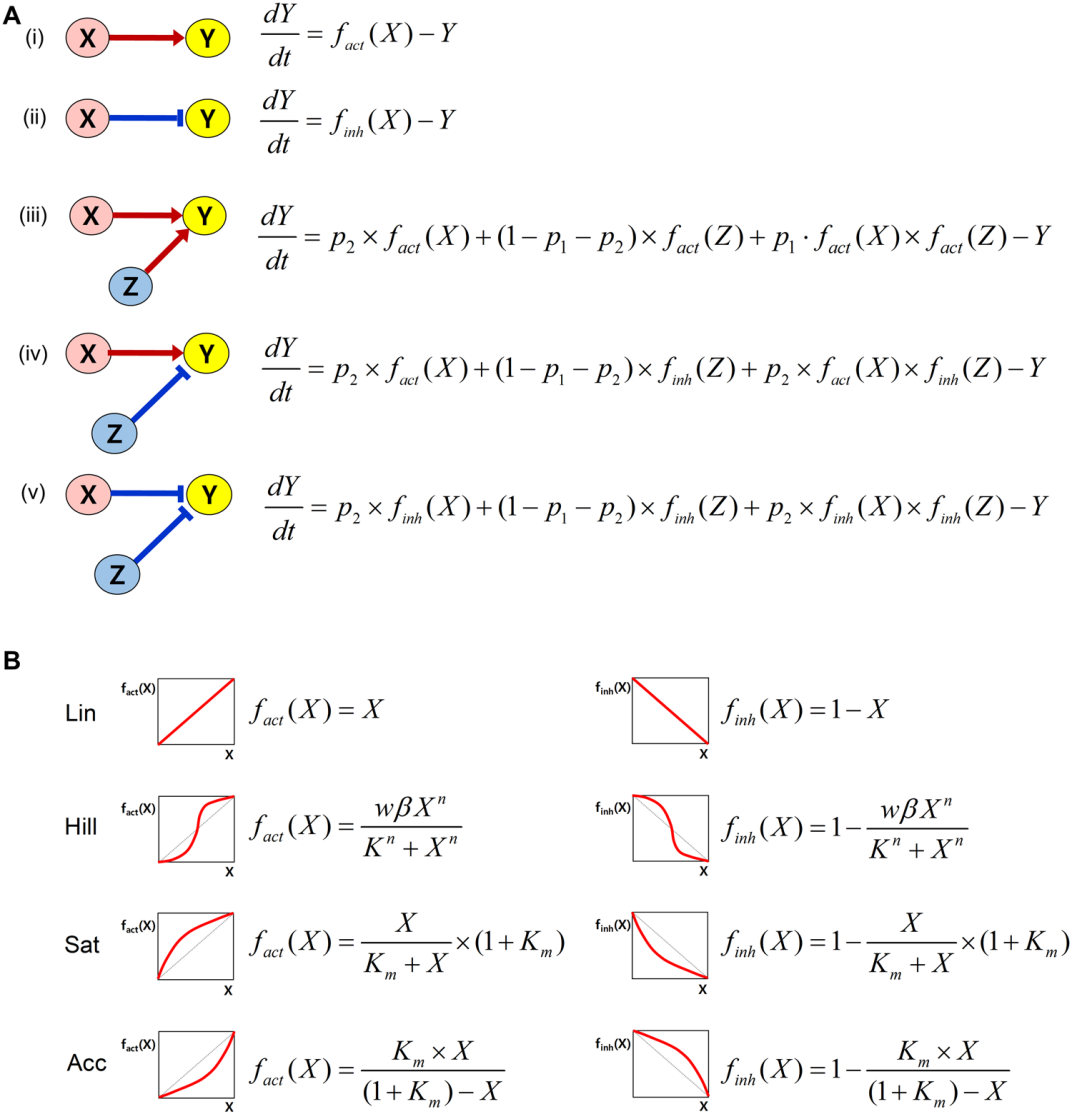
Normalized equation modeling. (**A**) Different forms of the differential equations according to the network structure are presented. (**B**) Four function types for the activation and the inhibition are presented.

Based on the network topology, a normalized equation model describes the dynamics of each network component, the activity of which is constrained between 0 (minimum activity) and 1 (maximum activity). The configuration of differential equations differs depending on the number of incoming links to the node and the nature of the links (i.e., activation or inhibition) (Fig. 5A). If one node (Y) is regulated by another node (X), the instantaneous rate of change in Y (dY/dt) is determined by X (Fig. 5A(i),(ii)). When one node (Y) is regulated by the other two nodes (X and Z), the instantaneous rate of change in Y (dY/dt) is determined by the sum of the three influences: the individual influence of X on Y, the individual influence of Z on Y, and the combined influence of X and Z on Y (Fig. 5A(iii),(iv),(v)). The equation includes two free parameters, p1 and p2: p1 represents the combined influence of X and Z on Y and p2 represents the individual influence of X on Y. The individual influence of Z on Y is then determined by 1 minus p1 minus p2. For example, if p1 is 0.2 and p2 is 0.3, the value of the individual influence of Z on Y is automatically determined as 0.5 (i.e., 1–0.2–0.3). Therefore, in this parameter set (i.e., p1 and p2), Y is influenced in the following descending order of strength: the individual influence of Z on Y (i.e., 0.5), the individual influence of X on Y (i.e., 0.3), and the combined influence of Z and X on Y (i.e., 0.2). When one node is regulated by the other three nodes, the formula for the instantaneous rate of the change in Y (dY/dt) becomes more complicated, requiring three free parameters (see Supplementary Text S1–S3 for details and exact formulation of equations).

We adopted four function types to describe activation or inhibition in a biologically plausible manner (Fig. 5B): the function type Lin represents a linear response of activation or inhibition; the function type Hill represents a sigmoidal response in a cooperative system; the function type Sat represents a response curve reacting to a low level of stimulus; and the function type Acc represents a response curve reacting to a high level of stimulus. Accordingly, four mathematical models with the four function types are established for one network topology.

### Distribution perturbation analysis

**Figure 6:**
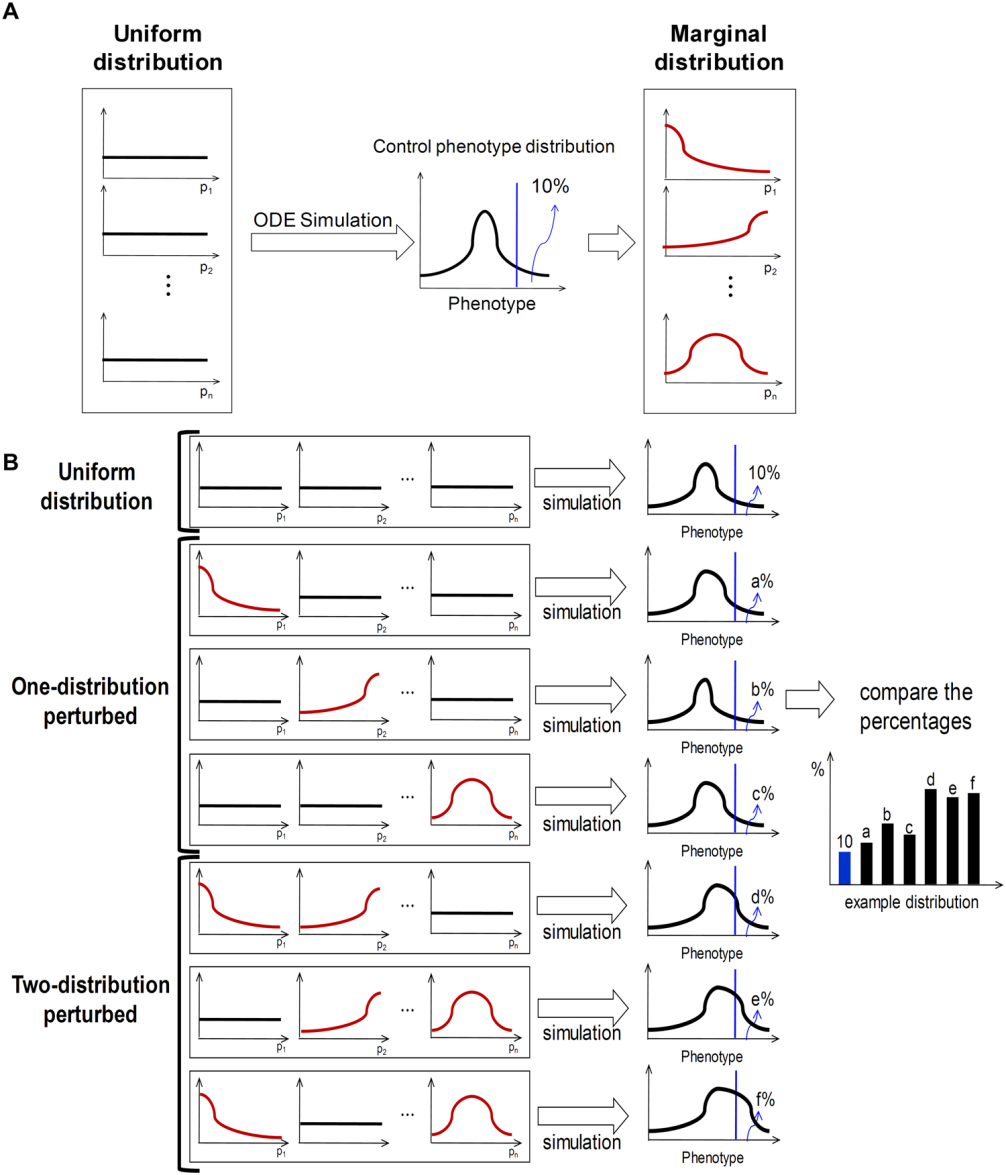
Distribution perturbation analysis. (**A**) After an initial simulation with random parameters sampled from a uniform distribution, the marginal distribution associated with a specific phenotype is calculated. (**B**) After one-distribution and two-distribution perturbation analyses, the resulting phenotype distributions are compared to the control phenotype distributions.

A distribution perturbation analysis was performed to investigate the causal relationship between marginal distributions of parameters and phenotype distributions. First, all parameters were randomly sampled from standard uniform distributions and a numerical simulation was conducted for each parameter set. Based on the results, distributions of phenotypes (control phenotype distributions) and the marginal distribution of each parameter were calculated (Fig. 6A). One-distribution perturbation analyses were performed for each of 37 parameters and two-distribution perturbation analyses were performed for each of 666 pairs of 37 parameters (_37_C_2_ = 37 × 36 ÷ 2 = 666). When each of the total 703 distribution perturbation analyses was conducted, the parameter or parameter pairs to be perturbed were sampled from marginal distributions and the remaining parameters were sampled from standard uniform distributions until one million random parameter sets were generated. These samplings were repeated for every phenotype, and for every function type. After a numerical simulation was performed for each parameter set, the distributions of the phenotypes were observed. These large-scale numerical simulations were performed using the parallel computing toolbox of MATLAB R2009a.

The resultant phenotype distributions were compared with the control phenotype distributions to determine which parameter or parameter pairs have an inducing or suppressing relationship with the phenotypes. In addition, phenotype distributions from two distribution perturbation analyses were compared with those from the one distribution perturbation analysis to identify which combination of parameters has a synergistic effect on generating either of the phenotypes (Fig. 6B).

The level of the synergistic effect for a phenotype was defined as the difference between (1) the degree of how much more frequently the phenotype appears in a two-distribution perturbation analysis and (2) the sum of the degrees of how much more frequently the phenotype appears in each one-distribution perturbation analysis with significance (p < 0.05). For instance, for apoptosis, if the degree for parameter 1 combined with parameter 2 is 0.5 in the two-distribution perturbation analysis and the degrees for parameters 1 and 2 are 0.1 and 0.15, respectively, in the one-distribution perturbation analyses, then the synergistic effect for apoptosis is expressed as 0.25 (i.e. 0.5–0.1–0.15).

### Validation of the developed method

To validate whether the normalized equation modeling can properly represent network dynamics, we constructed normalized equation models for the 16 network motifs (Supplementary Fig. S3). From the simulation analysis of those models, we found that well-known dynamical features of the network motifs could be successfully reproduced (Supplementary Fig. S4)^24, 25^. Next, to validate whether the distribution perturbation analyses are effective in identifying essential regulatory processes for the phenotypes, we applied the methods to the EGFR signaling network (Supplementary Fig. S5). As a result, the regulation of GAB1 was found to be an essential process for generating resistance to MEK inhibitors, which is consistent with published experimental results^26^. Detailed explanations of the validation processes are provided in Supplementary Text S1 and Supplementary Text S2.

### Cell culture

HL-1 (mouse cardiomyocytes) and H9C2 (rat cardiac cells) cell lines are commonly used as *in vitro* models of cardiomyocyte biology because they exhibit similar hypertrophic and apoptotic properties as those seen in primary adult and neonatal cardiomyocytes^27–29^. HL-1 and H9C2 cells were cultured in Dulbecco’s modified Eagle’s medium (WelGENE Inc.) with 10% fetal bovine serum (FBS) and antibiotics (1% penicillin/streptomycin/Fungizone) (Life Technologies Corp.) at 37 °C in a humidified atmosphere containing 5% CO_2_.

### Reagents

Isoproterenol, KN93, and ESI-09 were purchased from Sigma.

### CaMKII activity assay

CaMKII activity was determined by using a CaMKII assay kit (Upstate) according to the manufacturer’s instructions, which is based on the phosphorylation of a specific substrate peptide autocamtide-3 (KKALRRQETVDAL) by the CAMKII-induced transfer of [γ-32P] from [γ-32P]ATP.

### Analysis of cell death by ELISA

Cell apoptotic death was evaluated by using a Cell Death Detection ELISA PLUS kit (Roche Diagnostics) according to the manufacturer’s instructions, which measures cytoplasmic accumulation of histone-associated DNA fragments (mono- and oligonucleosomes) of apoptotic cells.

### Cell viability assay

Cell viability was measured using a cell counting kit-8 (CCK-8, Dojindo) by following the manufacturer’s instructions. In brief, CCK-8 solution was added to cells grown in 96 well plates for one to two hours and absorbance at 450 nm was measured using a VICTORTMX3 Multilabel Plate Reader (PerkinElmer Inc.).

## Electronic supplementary material

Supplementary Information
