# Supplementary material for: Context-independent essential regulatory interactions for apoptosis and hypertrophy in the cardiac signaling network
Source: Sci Rep. 2017 Feb 24;7:34. doi: 10.1038/s41598-017-00086-y (PMC5428364; doi:10.1038/s41598-017-00086-y)
Supplement: Supplementary file 1 — Supplementary Information [file 41598_2017_86_MOESM1_ESM.pdf]

Context-independent essential regulatory interactions for apoptosis and hypertrophy in the cardiac signaling network

Jun Hyuk Kang[1,*], Ho-Sung Lee[1,2,*], Daebeom Park[2], Yun-Won Kang[2], Seon Myeong Kim[2], Jeong-Ryeol Gong[2], and Kwang-Hyun Cho[1,2,†]

[1]Graduate School of Medical Science and Engineering, Korea Advanced Institute of Science and Technology (KAIST), Daejeon, 34141, Republic of Korea

[2]Laboratory for Systems Biology and Bio-inspired Engineering, Department of Bio and Brain Engineering, Korea Advanced Institute of Science and Technology (KAIST), Daejeon, 34141, Republic of Korea

Supplementary Information

*These authors contributed equally to this study.
†Corresponding author. E-mail: ckh@kaist.ac.kr, Phone: +82-42-350-4325, Fax: +82-42-350-4310, Web: http://sbie.kaist.ac.kr/.

Table of Contents

I. Supplementary Figures

Supplementray Figure S1. Therapeutic targets for treatment of apoptosis (*) and hypertrophy (#).

Supplementray Figure S2. Effect of inhibition of epac or CaMKII on the cellular responses of cardiomyocytes under catecholamine stimulation.

Supplementray Figure S3. Analyses of 16 network motifs.

Supplementray Figure S4. Marginal distributions of parameters and the results of the distribution perturbation analysis.

Supplementray Figure S5. Analyses of EGFR network.

Supplementray Figure S6. Constitution of the normalized equation model when one node (i.e., Y) is activated by three different nodes (i.e., A, B, and C).

II. Supplementary Tables

Supplementray Table S1. Differential equations of the cardiac signaling network model.

Supplementray Table S2. Represented regulatory process by parameters in the cardiac signaling network.

Supplementray Table S3. Type of equation and constraint of parameters in the cardiac signaling network model.

Supplementray Table S4. Verification data in the cardiac signaling network.

Supplementray Table S5. Verification results in the cardiac signaling network.

Supplementray Table S6. Results of the one-distribution perturbation analyses in the cardiac signaling network.

Supplementray Table S7. Results of two-distribution perturbation analysis when four

different response function types were applied in a combined manner.

Supplementray Table S8. Differential equations of the mathematical model of 16 network motifs.

Supplementray Table S9. Association of the network motifs and the dynamical features.

Supplementray Table S10. Dynamical features of the 16 network motifs.

Supplementray Table S11. Results of the distribution perturbation analysis of the 16 network motifs.

Supplementray Table S12. Differential equations of the EGFR model.

Supplementray Table S13. Relationship between interactions and parameters in the EGFR model.

Supplementray Table S14. Results of the distribution perturbation analysis of the EGFR network.

III. Supplementary Texts

Supplementray Text S1. Analysis of network motifs.

Supplementray Text S2. Analysis of the EGFR network.

Supplementray Text S3. Normalized equation modeling when one node is regulated by more than two nodes.

IV. Supplementary Data Sets

Supplementray Data Set S1. Experimental database for the cardiac signaling pathways.

Supplementray Data Set S2. Experimental evidence of links in the cardiac signaling network.

Supplementray Data Set S3. Results of the distribution perturbation analyses for apoptosis in the cardiac signaling network.

Supplementray Data Set S4. Results of the distribution perturbation analyses for

hypertrophy in the cardiac signaling network.

I. Supplementary Figures

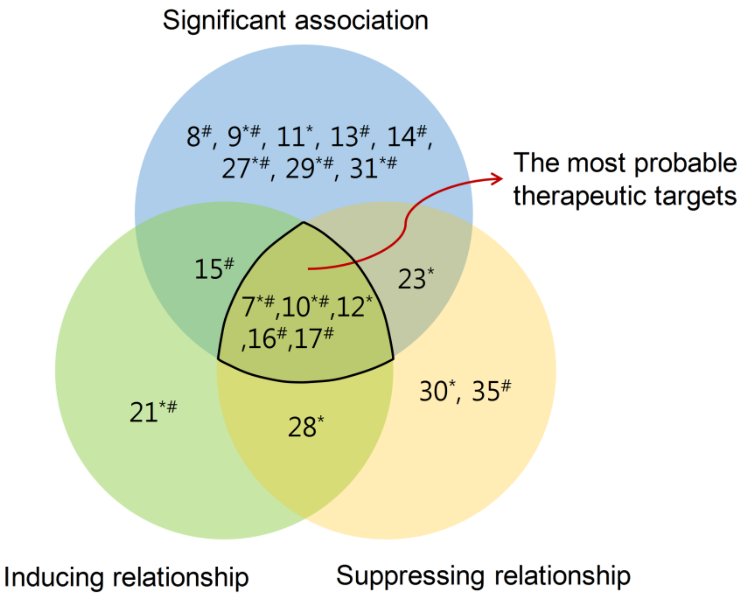

Supplementray Figure S1. Therapeutic targets for treatment of apoptosis (*) and hypertrophy (#).

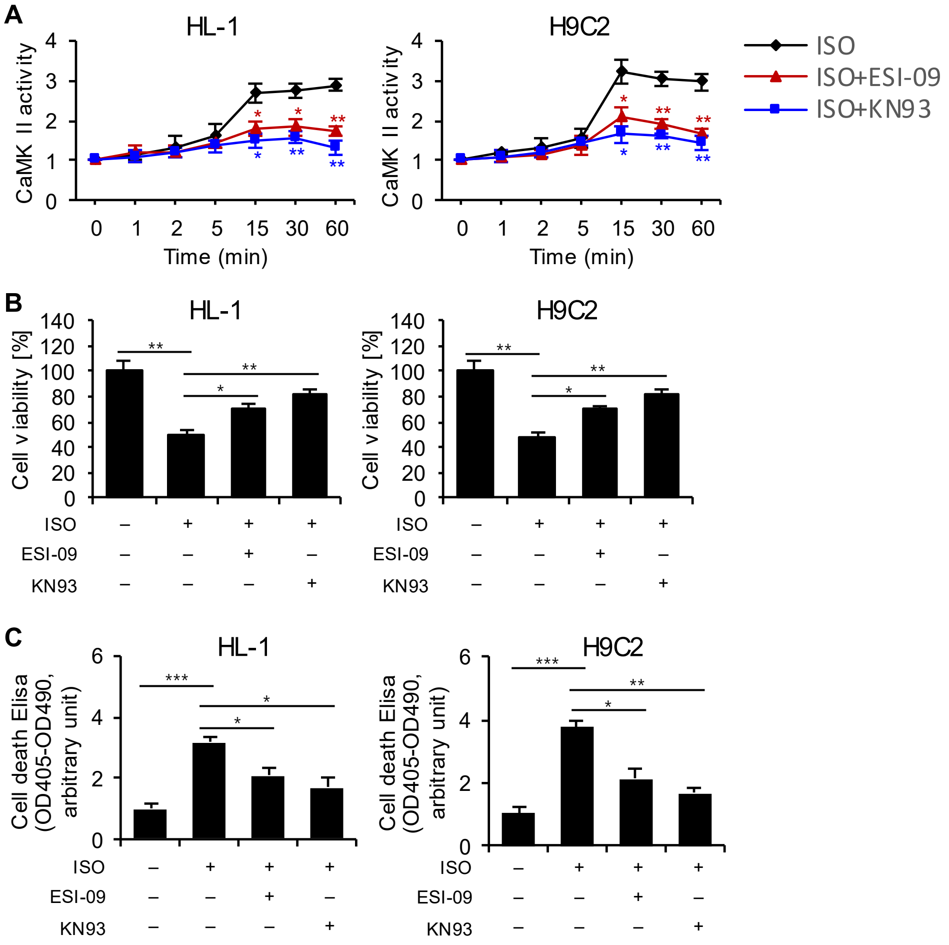

Supplementray Figure S2. Effect of inhibition of epac or CaMKII on the cellular responses of cardiomyocytes under catecholamine stimulation. (A) Graphs of the CaMKII activity in HL-1 and H9C2 cells treated with isoproterenol (ISO, a synthetic catecholamine that stimulates beta-adrenergic receptor signaling, 10 μM) for indicated time in the precence or absence of ESI-09 (an epac inhibitor, 10 μM) or KN93 (a CaMKII inhibitor, 5 μM) determined using a CaMKII assay. Red asterisks, significant differences between ISO-treated and ISO-ESI-09 co-treated cells; blue asterisks, significant differences between ISO-treated and ISO-KN93 co-treated cells. (B) Graphs of the percent of viable cells treated with ISO (10 μM) for 24 hours after the one-hour pre-treatment with ESI-09 (10 μM) or KN93 (5 μM) determined using a CCK-8 assay. (C) Graphs of the percent of apoptotic cells treated with ISO (10 μM) for 24 hours after the one-hour pre-treatment with ESI-09 (10 μM) or KN93 (5 μM) determined using a cell death ELISA assay. All data represent the mean ± S.E.M. of three biological replicates. *, $p < 0.05$; **, $p < 0.01$; ***, $p < 0.001$; ****, $p < 0.0001$; Student's *t* test.

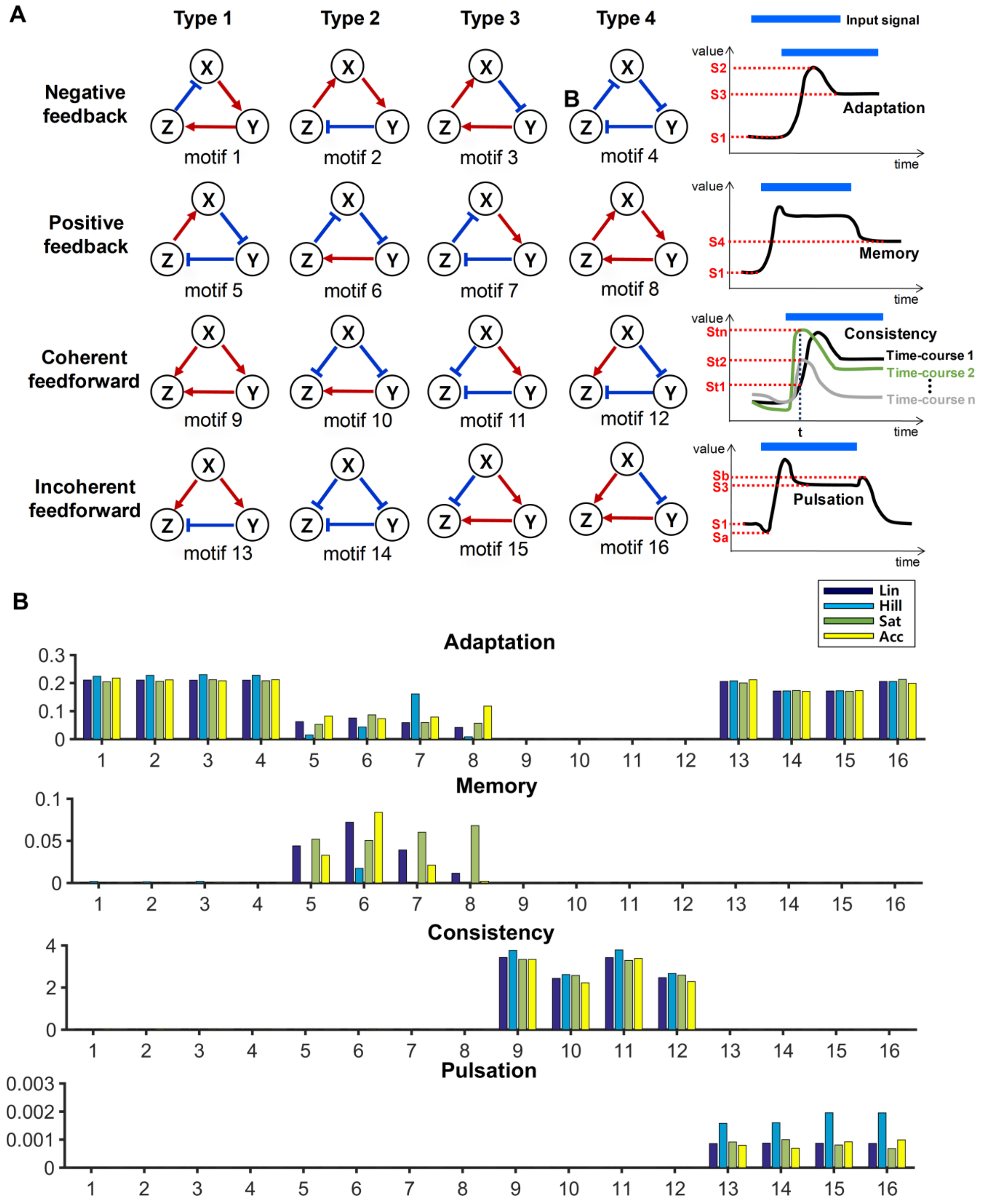

Supplementray Figure S3. Analyses of 16 network motifs. (A) Network structure of 16 network motifs. Dynamical features to be analyzed are demonstrated in the right side. Blue bars represents the duration of the given input signal. (B) Dynamical features of the 16 network motifs. The numbers marked on x-axis is the motif number. The value of the y-axis means relative portion of parameters that generate the dynamics of interest. For a detailed explanation, see Supplementary Texts.

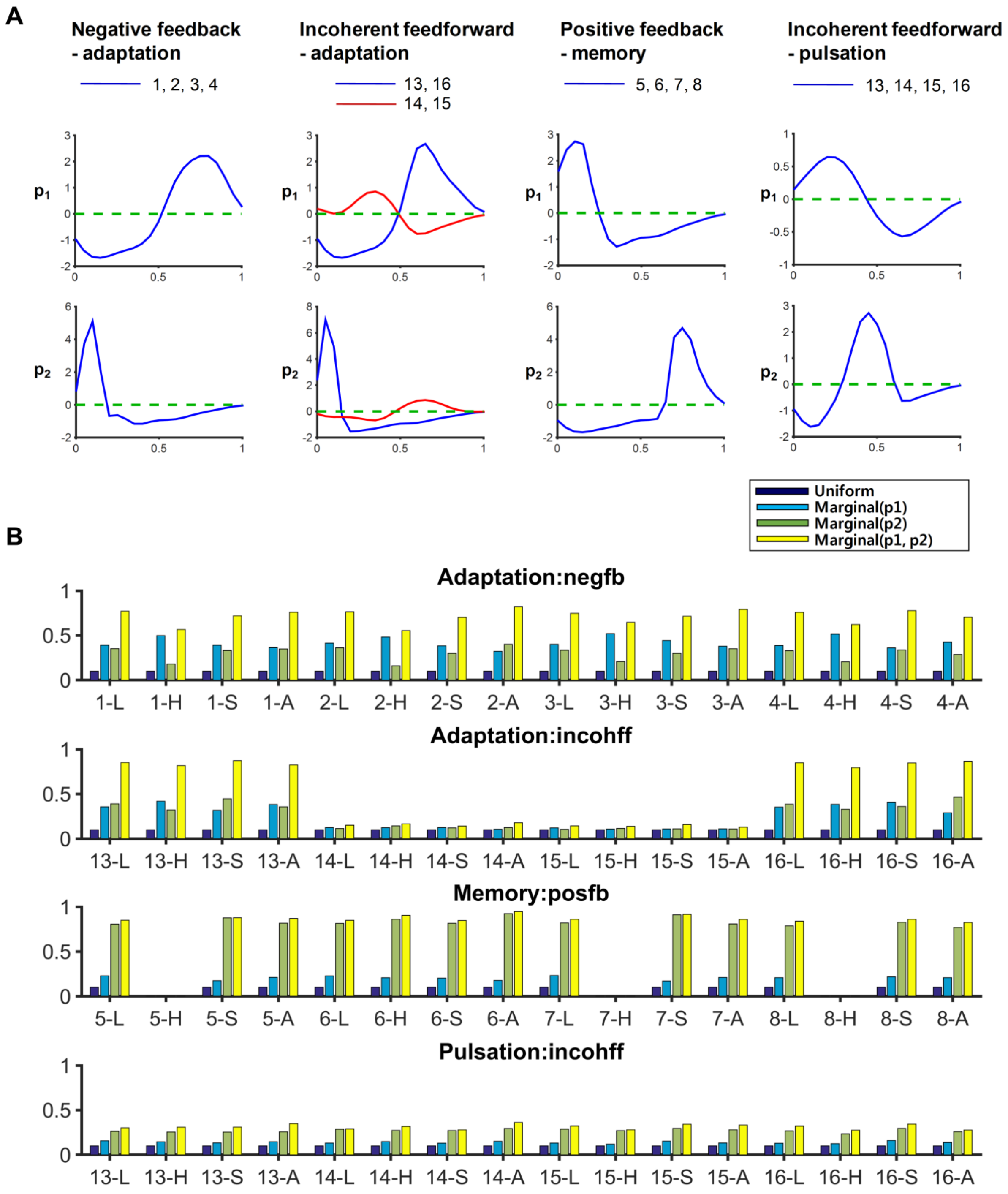

Supplementray Figure S4. Marginal distributions of parameters and the results of the distribution perturbation analysis. (A) Shape of marginal distribution for four associations of the network structure and dynamics (B) The results of distribution perturbation analysis. The label (A-B) on the x-axis means type A with function B used. B is one among L(Lin), H(Hill), S(Sat), A(Acc). For a detailed explanation, see Supplementary Texts.

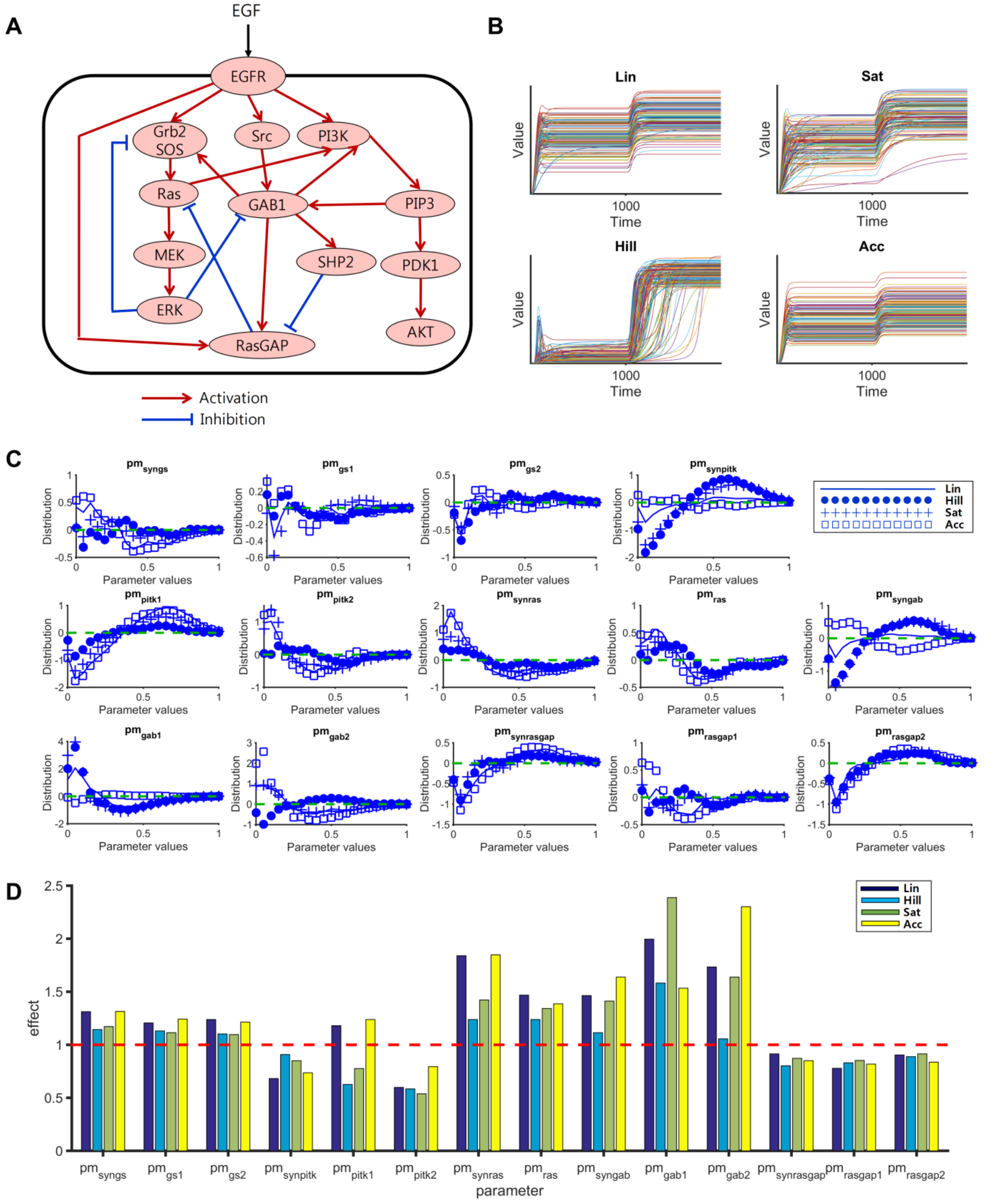

Supplementray Figure S5. Analyses of EGFR network. (A) Representation of EGFR network. (B) Resistance to the MEK inhibitor was identified. (C) Marginal distributions of the parameters that are associated with MEK inhibitor resistance. (D) The results of one distribution perturbation analysis. For detailed a explanation, see Supplementary Texts.

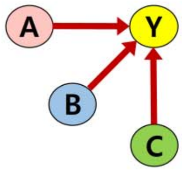

$$\frac{dY}{dt} = p_2 \times f_{act}(A) + p_3 \times f_{act}(B) + (1 - p_1 - p_2 - p_3) \times f_{act}(C)$$
$$+ p_{ab} \times f_{act}(A) \times f_{act}(B) + p_{bc} \times f_{act}(B) \times f_{act}(C) + p_{ca} \times f_{act}(C) \times f_{act}(A)$$
$$+ p_{abc} \times f_{act}(A) \times f_{act}(B) \times f_{act}(C) - Y$$

$$p_{ab} = p_2 \times p_3,\ \ p_{bc} = p_3 \times (1 - p_1 - p_2 - p_3),\ \ p_{ca} = (1 - p_1 - p_2 - p_3) \times p_2$$

$$p_4 = \frac{p_{ab}}{p_{ab} + p_{bc} + p_{ca}} \times (1 - p_1) \times p_1, p_5 = \frac{p_{bc}}{p_{ab} + p_{bc} + p_{ca}} \times (1 - p_1) \times p_1$$

$$p_6 = \frac{p_{ca}}{p_{ab} + p_{bc} + p_{ca}} \times (1 - p_1) \times p_1, p_7 = p_1 \times p_1$$

Supplementray Figure S6. Constitution of the normalized equation model when one node (i.e., Y) is activated by three different nodes (i.e., A, B, C). For a detailed explanation, see Supplementary Texts.

Supplementary Tables

Supplementtray Table S1. Differential equations of the cardiac signaling network model.

Eq. no.	Differential equation
(1)	$\frac{d[BAR]}{dt} = ISO \times (1 - BARp - BARd) - BAR$
(2)	$\frac{d[BARp]}{dt} = pm_1 \times f_{act}(BAR) \times f_{act}(PKA) - BARp$
(3)	$\frac{d[BARd]}{dt} = pm_2 \times f_{act}(BAR + BARp) \times f_{act}(bARKnPI3K) - BARd$
(4)	$\frac{d[bARK]}{dt} = f_{act}(Gbg) - bARK$
(5)	$\frac{d[Gs]}{dt} = f_{act}(BAR) - Gs$
(6)	$\frac{d[Gi]}{dt} = f_{act}(BARp) - Gi$
(7)	$\frac{d[AC]}{dt} = pm_3 \times f_{act}(Gs) - (ADD - pm_3) \times f_{inh}(Gi) + SYN \times f_{act}(Gs) \times f_{inh}(Gi) - AC$
(8)	$\frac{d[cAMP]}{dt} = pm_4 \times f_{act}(AC) + (ADD - pm_4) \times f_{inh}(PDE34) + SYN \times f_{act}(AC) \times f_{inh}(PDE34) - cAMP$
(9)	$\frac{d[PKA]}{dt} = f_{act}(cAMP) - PKA$

(10)	$\frac{d[epac]}{dt} = f_{act}(cAMP) - epac$
(11)	$\frac{d[PDE34]}{dt} = pm_5 \times f_{act}(PKA) - (ADD - pm_5) \times f_{act}(PKC) + SYN \times f_{act}(PKA) \times f_{act}(PKC) - PDE34$
(12)	$\frac{d[CREB]}{dt} = pm_6 \times f_{act}(PKA) - (ADD - pm_6) \times f_{act}(MSK1) + SYN \times f_{act}(PKA) \times f_{act}(MSK1) - CREB$
(13)	$\frac{d[aAR]}{dt} = PE - aAR$
(14)	$\frac{d[Gq]}{dt} = pm_7 \times f_{act}(aAR) - (ADD - pm_7) \times f_{inh}(RGS4) + SYN \times f_{act}(aAR) \times f_{inh}(RGS4) - Gq$
(15)	$\frac{d[Gbg]}{dt} = \frac{(f_{act}(Gi) + f_{act}(Gq))}{2} - Gbg$
(16)	$\frac{d[PLC]}{dt} = f_{act}(Gq) - PLC$
(17)	$\frac{d[IP3]}{dt} = f_{act}(PLC) - IP3$
(18)	$\frac{d[DAG]}{dt} = f_{act}(PLC) - DAG$
(19)	$\frac{d[PKC]}{dt} = pm_8 \times f_{act}(DAG) \times f_{act}(Ca) + (1 - pm_8) \times f_{act}(DAG) \times f_{act}(Ca) \times f_{act}(CaN) - PKC$
(20)	$\frac{d[TAK1]}{dt} = f_{act}(PKC) - TAK1$

(21)	$PKAPI3K = pm_9 \times f_{act}(PKA) \times f_{inh}(PI3Krct) + (1 - pm_9) \times f_{act}(PKA)$ $\frac{d[Ca]}{dt} = pm_{10} \times f_{act}(IP3) + (ADD - pm_{10}) \times PKAPI3K + SYN \times f_{act}(IP3) \times PKAPI3K - Ca$
(22)	$\frac{d[CaM]}{dt} = f_{act}(Ca) - CaM$
(23)	$epacPKC = pm_{11} \times f_{act}(epac) \times f_{act}(PKC) + (1 - pm_{11}) \times f_{act}(epac)$ $\frac{d[CaMK]}{dt} = pm_{12} \times f_{act}(CaM) \times epacPKC + (1 - pm_{12}) \times f_{act}(CaM) - CaMK$
(24)	$\frac{d[CaN]}{dt} = f_{act}(CaM) - CaN$
(25)	$\frac{d[HDAC]}{dt} = pm_{13} \times f_{inh}(CaMK) + pm_{14} \times f_{inh}(PKC) + (ADD - pm_{13} - pm_{14}) \times f_{act}(PKA) +$ $pm_{hdac12} \times f_{inh}(CaMK) \times f_{inh}(PKC) + pm_{hdac23} \times f_{inh}(PKC) \times f_{act}(PKA) +$ $pm_{hdac31} \times f_{act}(PKA) \times f_{inh}(CaMK) + SYN_3 \times f_{inh}(CaMK) \times f_{inh}(PKC) \times f_{act}(PKA) - HDAC$

(26)	$\frac{d[NFATnuc]}{dt} = pm_{15} \times f_{act}(CaN) + pm_{16} \times f_{inh}(JNK) + pm_{17} \times f_{inh}(p38)$ $+ (ADD - pm_{15} - pm_{16} - pm_{17}) \times f_{inh}(GSK3B) + pm_{nfatnuc12} \times f_{act}(CaN) \times f_{inh}(JNK) +$ $pm_{nfatnuc13} \times f_{act}(CaN) \times f_{inh}(p38) + pm_{nfatnuc14} \times f_{act}(CaN) \times f_{inh}(GSK3B) +$ $pm_{nfatnuc23} \times f_{inh}(JNK) \times f_{inh}(p38) + pm_{nfatnuc24} \times f_{inh}(JNK) \times f_{inh}(GSK3B) +$ $pm_{nfatnuc34} \times f_{inh}(p38) \times f_{inh}(GSK3B) + pm_{nfatnuc123} \times f_{act}(CaN) \times f_{inh}(JNK) \times f_{inh}(p38)$ $+ pm_{nfatnuc124} \times f_{act}(CaN) \times f_{inh}(JNK) \times f_{inh}(GSK3B)$ $+ pm_{nfatnuc134} \times f_{act}(CaN) \times f_{inh}(p38) \times f_{inh}(GSK3B)$ $+ pm_{nfatnuc234} \times f_{inh}(JNK) \times f_{inh}(p38) \times f_{inh}(GSK3B)$ $+ SYN_4 \times f_{act}(CaN) \times f_{inh}(JNK) \times f_{inh}(p38) \times f_{inh}(GSK3B) - NFATnuc$
(27)	$AP1 = 0.5 \times cFos \times cJun + 0.5 \times cJun \times cJun$ $cFoscJunGSK3B = pm_{18} \times f_{act}(AP1) + (ADD - pm_{18}) \times f_{inh}(GSK3B) + SYN \times f_{act}(AP1) \times f_{inh}(GSK3B)$ $\frac{d[NFATact]}{dt} = pm_{19} \times f_{act}(NFATnuc) \times cFoscJunGSK3B + (1 - pm_{19}) \times f_{act}(NFATnuc) - NFATact$
(28)	$\frac{d[Src]}{dt} = pm_{20} \times f_{act}(Gbg) \times f_{act}(CaN) + (1 - pm_{20}) \times f_{act}(Gbg) - Src$
(29)	$\frac{d[Shc]}{dt} = f_{act}(Src) - Shc$
(30)	$\frac{d[Grb2Sos]}{dt} = pm_{21} \times f_{inh}(ERK12) - (ADD - pm_{21}) \times f_{act}(Shc) + SYN \times f_{inh}(ERK12) \times f_{act}(Shc) - Grb2Sos$
(31)	$\frac{d[Ras]}{dt} = f_{act}(Grb2Sos) - Ras$

(32)	$\frac{d[Rac]}{dt} = f_{act}(Ras) - Rac$
(33)	$\frac{d[Raf1]}{dt} = pm_{22} \times f_{act}(Ras) + pm_{23} \times f_{act}(PKC) + (ADD - pm_{22} - pm_{23}) \times f_{inh}(PKA) +$ $pm_{raf12} \times f_{act}(Ras) \times f_{act}(PKC) + pm_{raf23} \times f_{act}(PKC) \times f_{inh}(PKA) + pm_{raf31} \times f_{inh}(PKA) \times f_{act}(Ras) +$ $SYN_3 \times f_{act}(Ras) \times f_{act}(PKC) \times f_{inh}(PKA) - Raf1$
(34)	$\frac{d[MEKK1]}{dt} = f_{act}(Rac) - MEKK1$
(35)	$\frac{d[MEKK234]}{dt} = f_{act}(Rac) - MEKK234$
(36)	$\frac{d[MEKK11]}{dt} = f_{act}(Rac) - MEKK11$
(37)	$\frac{d[MEK12]}{dt} = pm_{24} \times f_{act}(Raf1) - (ADD - pm_{24}) \times f_{act}(MEKK1)$ $+ SYN \times f_{act}(Raf1) \times f_{act}(MEKK1) - MEK12$
(38)	$\frac{d[MEK4]}{dt} = pm_{25} \times f_{act}(MEKK1) - (ADD - pm_{25}) \times f_{act}(MEKK234)$ $+ SYN \times f_{act}(MEKK1) \times f_{act}(MEKK234) - MEK4$
(39)	$\frac{d[MEK7]}{dt} = pm_{26} \times f_{act}(MEKK234) - (ADD - pm_{26}) \times f_{act}(MEKK11)$ $+ SYN \times f_{act}(MEKK234) \times f_{act}(MEKK11) - MEK7$

(40)	$\frac{d[MEK36]}{dt} = pm_{27} \times f_{act}(TAK1) - (ADD - pm_{27}) \times f_{act}(MEKK11)$ $+ SYN \times f_{act}(TAK1) \times f_{act}(MEKK11) - MEK36$
(41)	$\frac{d[ERK12]}{dt} = pm_{28} \times f_{act}(Gbg) - (ADD - pm_{28}) \times f_{act}(MEK12) + SYN \times f_{act}(Gbg) \times f_{act}(MEK12) - ERK12$
(42)	$\frac{d[JNK]}{dt} = pm_{29} \times f_{act}(CaN) + pm_{30} \times f_{act}(MEK4) + (ADD - pm_{29} - pm_{30}) \times f_{act}(MEK7) +$ $pm_{jnk12} \times f_{act}(CaN) \times f_{act}(MEK4) + pm_{jnk23} \times f_{act}(MEK4) \times f_{act}(MEK7) +$ $pm_{jnk31} \times f_{act}(MEK7) \times f_{act}(CaN) + SYN_3 \times f_{act}(CaN) \times f_{act}(MEK4) \times f_{act}(MEK7) - JNK$
(43)	$\frac{d[p38]}{dt} = pm_{31} \times f_{act}(MEK36) - (ADD - pm_{31}) \times f_{act}(MEK4) + SYN \times f_{act}(MEK36) \times f_{act}(MEK4) - p38$
(44)	$\frac{d[PI3Krct]}{dt} = bARKnPI3K + Grb2nPI3K - PI3Krct$ $PI3Knrct = 1 - PI3Krct$
(45)	$\frac{d[Grb2nPI3K]}{dt} = f_{act}(Grb2Sos) \times PI3Knrct - Grb2nPI3K$
(46)	$\frac{d[bARKnPI3K]}{dt} = f_{act}(bARK) \times PI3Knrct - bARKnPI3K$
(47)	$\frac{d[Akt]}{dt} = f_{act}(PI3Krct) - Akt$
(48)	$\frac{d[GSK3B]}{dt} = f_{inh}(Akt) - GSK3B$

(49)	$\frac{d[cGMP]}{dt} = pm_{32} \times f_{inh}(PDE5) - (ADD - pm_{32}) \times f_{act}(sGC) + SYN \times f_{inh}(PDE5) \times f_{act}(sGC) - cGMP$
(50)	$\frac{d[MEF2]}{dt} = pm_{33} \times f_{act}(p38) - (ADD - pm_{33}) \times f_{inh}(HDAC) + SYN \times f_{act}(p38) \times f_{inh}(HDAC) - MEF2$
(51)	$\frac{d[GATA4]}{dt} = pm_{34} \times f_{act}(ERK12) + pm_{35} \times f_{act}(p38) + (ADD - pm_{34} - pm_{35}) \times f_{inh}(GSK3B) +$ $pm_{gata12} \times f_{act}(ERK12) \times f_{act}(p38) + pm_{gata23} \times f_{act}(p38) \times f_{inh}(GSK3B) +$ $pm_{gata31} \times f_{inh}(GSK3B) \times f_{act}(ERK12) + SYN_3 \times f_{act}(ERK12) \times f_{act}(p38) \times f_{inh}(GSK3B) - GATA4$
(52)	$\frac{d[cFos]}{dt} = f_{act}(ERK12) - cFos$
(53)	$\frac{d[cJun]}{dt} = pm_{36} \times f_{act}(JNK) - (ADD - pm_{36}) \times f_{act}(p38) + SYN \times f_{act}(JNK) \times f_{act}(p38) - cJun$
(54)	$\frac{d[MSK1]}{dt} = pm_{37} \times f_{act}(ERK12) - (ADD - pm_{37}) \times f_{act}(p38) + SYN \times f_{act}(ERK12) \times f_{act}(p38) - MSK1$
(55)	$\frac{d[NOS]}{dt} = f_{act}(Akt) - NOS$
(56)	$\frac{d[sGC]}{dt} = f_{act}(NOS) - sGC$
(57)	$\frac{d[PKG]}{dt} = f_{act}(cGMP) - PKG$
(58)	$\frac{d[PDE5]}{dt} = f_{act}(PKG) - PDE5$

(59)	$\frac{d[RGS4]}{dt} = f_{act}(PKG) - RGS4$
(50)	$Apoptosis = \frac{[CaMK]+[JNK]+[p38]-[CREB]-[ERK12]}{5}$
(51)	$Hypertrophy = \frac{[NFATact]+[MEF2]+[GATA4]}{5}$

ADD is set to be 0.8 and SYN is 0.2. SYN_3=SYN×SYN, SYN_4=SYN×SYN×SYN

Supplementray Table S2. Represented regulatory process by parameters in the cardiac signaling network.

Parameter no.	Represented link or biological process by the parameter
pm_1	maximal degree of BAR phosphorylation
pm_2	maximal degree of BAR desensitization
pm_3	Gs/Gi → AC
pm_4	AC/PDE34 → cAMP
pm_5	PKA/PKC → PDE34
pm_6	PKA/MSK1 → CREB
pm_7	αAR/RGS4 → Gq
pm_8	(DAG & Ca)/(DAG & Ca & CaN) → PKC
pm_9	(PKA & PI3K)/PKA → activated PKA for Ca
pm_{10}	IP3/activated PKA → Ca
pm_{11}	(epac & PKC)/epac → activated epac for CaMK
pm_{12}	(CaM & activated epac)/CaM → CaMK
pm_{13}	CaMK/(PKC, PKA) → HDAC
pm_{14}	PKC/(CaMK, PKA) → HDAC
pm_{15}	CaN/(JNK, p38) → NFATnuc
pm_{16}	JNK/(CaN, p38) → NFATnuc
pm_{17}	p38/(CaN, JNK) → NFATnuc
pm_{18}	AP1/GSK3B → activated AP1
pm_{19}	(NFATnuc & activated AP1)/NFATnuc → NFATact
pm_{20}	(Gbg & CaN)/Gbg → Src
pm_{21}	ERK12/Shc → Grb2Sos
pm_{22}	Ras/(PKC, PKA) → Raf1
pm_{23}	PKC/(Ras, PKA) → Raf1
pm_{24}	Raf1/MEKK1 → MEK12
pm_{25}	MEKK1/MEKK234 → MEK4
pm_{26}	MEKK234/MEKK11 → cJun
pm_{27}	TAK1/MEKK11 → MEK36
pm_{28}	Gbg/MEK12 → ERK12
pm_{29}	CaN/(MEK4, MEK7) → JNK
pm_{30}	MEK4/(CaN, MEK7) → JNK
pm_{31}	MEK36/MEK4 → p38

pm_{32}	PDE5/sGC → cGMP
pm_{33}	p38/HDAC → MEF2
pm_{34}	ERK12/(p38, GSK3B) → GATA4
pm_{35}	p38/(ERK12, GSK3B) → GATA4
pm_{36}	JNK/p38 → cJun
pm_{37}	ERK12/p38 → MSK1

Note that the mark ' → ' represents influence that is either activation or inhibition.

Supplementray Table S3. Type of equation and constraint of parameters in the cardiac signaling network model.

Eq no.	Dependent variable	Related parameter	Equation type[a]	Constraints of parameter
(2)	BARp[b]	pm_1	sub	$0 \le pm_1 \le 1$
(3)	BARd[b]	pm_2	sub	$0 \le pm_2 \le 1$
(7)	AC	pm_3	add	$0 \le pm_3 \le 1-SYN$
(8)	cAMP	pm_4	add	$0 \le pm_4 \le 1-SYN$
(11)	PDE34	pm_5	add	$0 \le pm_5 \le 1-SYN$
(12)	CREB	pm_6	add	$0 \le pm_6 \le 1-SYN$
(14)	Gq	pm_7	add	$0 \le pm_7 \le 1-SYN$
(19)	PKC[c]	pm_8	sub	$0 \le pm_8 \le 1$
(21)	Ca[d]	pm_9	sub	$0 \le pm_9 \le 1$
(21)	Ca[d]	pm_{10}	add	$0 \le pm_{10} \le 1-SYN$
(23)	CaMK	pm_{11}	sub	$0 \le pm_{11} \le 1$
(23)	CaMK	pm_{12}	sub	$0 \le pm_{12} \le 1$
(25)	HDAC	pm_{13}, pm_{14}	add	$0 \le pm_{13}, pm_{14} \le 1-SYN$<br>$pm_{13} + pm_{14} \le 1-SYN$<br>$pm_{rem} = 1-SYN-pm_{13}-pm_{14}$<br>$sum_{hdac} = pm_{13} \times pm_{14} + pm_{14} \times pm_{rem} + pm_{rem} \times pm_{13}$<br>$pm_{hdac12} = (pm_{13} \times pm_{14} / sum_{hdac}) \times (SYN-SYN3)$<br>$pm_{hdac23} = (pm_{14} \times pm_{rem} / sum_{hdac}) \times (SYN-SYN3)$<br>$pm_{hdac31} = (pm_{rem} \times pm_{13} / sum_{hdac}) \times (SYN-SYN3)$

(26)	NFATnuc	pm_{15}, pm_{16}, pm_{17}	add	$0\leq pm_{15}, pm_{16}, pm_{17}\leq 1\text{-}SYN$<br>$pm_{15}+pm_{16}+pm_{17}\leq 1\text{-}SYN$<br>$pm_{rem}=1-pm_{15}-pm_{16}-pm_{17}$<br>$SYN_{4\_2}=SYN\times(1-SYN)$<br>$SYN_{4\_3}=SYN\times SYN\times (1-SYN)$<br>$sum_2=pm_{15}\times pm_{16}+ pm_{15}\times pm_{17} + pm_{15}\times pm_{rem}+ pm_{16}\times pm_{17} + pm_{16}\times pm_{rem}+ pm_{17}\times pm_{rem}$<br>$sum_3=pm_{15}\times pm_{16}\times pm_{17}+ pm_{15}\times pm_{16}\times pm_{rem} + pm_{15}\times pm_{17}\times pm_{rem}+ pm_{16}\times pm_{17}\times pm_{rem}$<br>$pm_{nfatnuc12}=((pm_{15}\times pm_{16})/sum_2)\times SYN_{4\_2}$<br>$pm_{nfatnuc13}=((pm_{15}\times pm_{17})/sum_2)\times SYN_{4\_2}$<br>$pm_{nfatnuc14}=((pm_{15}\times pm_{rem})/sum_2)\times SYN_{4\_2}$<br>$pm_{nfatnuc23}=((pm_{16}\times pm_{17})/sum_2)\times SYN_{4\_2}$<br>$pm_{nfatnuc24}=((pm_{16}\times pm_{rem})/sum_2)\times SYN_{4\_2}$<br>$pm_{nfatnuc34}=((pm_{17}\times pm_{rem})/sum_2)\times SYN_{4\_2}$<br>$pm_{nfatnuc123}=((pm_{15}\times pm_{16}\times pm_{17})/sum_3)\times SYN_{4\_3}$<br>$pm_{nfatnuc124}=((pm_{15}\times pm_{16}\times pm_{rem})/sum_3)\times SYN_{4\_3}$<br>$pm_{nfatnuc134}=((pm_{15}\times pm_{17}\times pm_{rem})/sum_3)\times SYN_{4\_3}$<br>$pm_{nfatnuc234}=((pm_{16}\times pm_{17}\times pm_{rem})/sum_3)\times SYN_{4\_3}$
(27)	NFATact	pm_{18}	add	$0\leq pm_{18}\leq 1\text{-}SYN$
(27)	NFATact	pm_{19}	sub	$0\leq pm_{19}\leq 1$
(28)	Src	pm_{20}	sub	$0\leq pm_{20}\leq 1$
(29)	Grb2Sos	pm_{21}	add	$0\leq pm_{21}\leq 1\text{-}SYN$

(33)	Raf1	pm_{22}, pm_{23}	add	$0\leq pm_{22}, pm_{23} \leq 1\text{-SYN}$<br>$pm_{22}+ pm_{23} \leq 1\text{-SYN}$<br>$pm_{rem}=1\text{-SYN-}pm_{22}\text{-}pm_{23}$<br>$sum_{raf}=pm_{22} \times pm_{23}+ pm_{23} \times pm_{rem} + pm_{rem} \times pm_{22}$<br>$pm_{raf12}=(pm_{22} \times pm_{23}/ sum_{raf})\times(SYN\text{-}SYN3)$<br>$pm_{raf23}=(pm_{23} \times pm_{rem}/ sum_{raf})\times(SYN\text{-}SYN3)$<br>$pm_{raf31}=(pm_{rem} \times pm_{22}/ sum_{raf})\times(SYN\text{-}SYN3)$
(37)	MEK12	pm_{24}	add	$0\leq pm_{24} \leq 1\text{-SYN}$
(38)	MEK4	pm_{25}	add	$0\leq pm_{25} \leq 1\text{-SYN}$
(39)	MEK7	pm_{26}	add	$0\leq pm_{26} \leq 1\text{-SYN}$
(40)	MEK36	pm_{27}	add	$0\leq pm_{27} \leq 1\text{-SYN}$
(41)	ERK12	pm_{28}	add	$0\leq pm_{28} \leq 1\text{-SYN}$
(42)	JNK	pm_{29}, pm_{30}	add	$0\leq pm_{29}, pm_{30} \leq 1\text{-SYN}$<br>$pm_{29}+ pm_{30} \leq 1\text{-SYN}$<br>$pm_{rem}=1\text{-SYN-}pm_{29}\text{-}pm_{30}$<br>$sum_{jnk}=pm_{29} \times pm_{30}+ pm_{30} \times pm_{rem} + pm_{rem} \times pm_{29}$<br>$pm_{jnk12}=(pm_{29} \times pm_{30}/ sum_{jnk})\times(SYN\text{-}SYN3)$<br>$pm_{jnk23}=(pm_{30} \times pm_{rem}/ sum_{jnk})\times(SYN\text{-}SYN3)$<br>$pm_{jnk31}=(pm_{rem} \times pm_{29}/ sum_{jnk})\times(SYN\text{-}SYN3)$
(43)	p38	pm_{31}	add	$0\leq pm_{31} \leq 1\text{-SYN}$
(49)	cGMP	pm_{32}	add	$0\leq pm_{32} \leq 1\text{-SYN}$
(50)	MEF2	pm_{33}	add	$0\leq pm_{33} \leq 1\text{-SYN}$
(51)	GATA4	pm_{34}, pm_{35}	add	$0\leq pm_{34}, pm_{35} \leq 1\text{-SYN}$

				$pm_{34}+pm_{35} \leq 1\text{-SYN}$<br>$pm_{rem}=1\text{-SYN}-pm_{34}-pm_{35}$<br>$sum_{gata}=pm_{34} \times pm_{35}+pm_{35} \times pm_{rem}+pm_{rem} \times pm_{34}$<br>$pm_{gata12}=(pm_{34} \times pm_{35}/sum_{gata}) \times (\text{SYN-SYN3})$<br>$pm_{gata23}=(pm_{35} \times pm_{rem}/sum_{gata}) \times (\text{SYN-SYN3})$<br>$pm_{gata31}=(pm_{rem} \times pm_{34}/sum_{gata}) \times (\text{SYN-SYN3})$
(53)	cJun	pm_{36}	add	$0 \leq pm_{36} \leq 1\text{-SYN}$
(54)	MSK1	pm_{37}	add	$0 \leq pm_{37} \leq 1\text{-SYN}$

[a]Constitution of differential equation is classified to two classes: type add and type sub. Type add is used when different links converging to one node can take independent roles in regulating the node. In contrast, type sub is used when one link takes a subsidiary role in regulating the node in the same situation.

[b]Because these equations represent process of transformation, equation type should be 'sub'

[c]Both Ca and DAG is required to activate PKC and CaN takes subsidiary role in such activation.

[d]Since PI3K only inhibits PKA-induced calcium activation, the equation takes the form of the stepwise activation rather than direct convergence of three different links

Supplementray Table S4. Verification data in the cardiac signaling network.

Reference (PMID)	Experimental setting	Input	Output	Effect	Time
11585926	cardiomyocytes + Ad GATA4 TF	PE(10uM)	p-GATA4/GATA4	3.5	0hr
11585926	cardiomyocytes + Ad GATA4 TF	PE(10uM)	p-GATA4/GATA4	5	1hr
11585926	cardiomyocytes + Ad GATA4 TF	PE(10uM)	p-GATA4/GATA4	8	3hr
11585926	cardiomyocytes + Ad GATA4 TF	PE(10uM)	p-GATA4/GATA4	10	24hr
11585926	NRVM	Saline	p-GATA4/GATA4	1	3hr
11585926	NRVM	PE	p-GATA4/GATA4	3	3hr
10737771	NRCM	none	MEF2	1	NA
10737771	NRCM	PE	MEF2	4.5	NA
15522277	NRVM	PE	CREB	0.2	0min
15522277	NRVM	PE	CREB	0.3	1min
15522277	NRVM	PE	CREB	0.5	2min
15522277	NRVM	PE	CREB	1.5	5min
15522277	NRVM	PE	CREB	1.4	10min
15522277	NRVM	PE	CREB	1.5	15min
15522277	NRVM	PE	CREB	0.6	30min
15522277	NRVM	none	MSK1	50	10min
15522277	NRVM	PE	MSK1	200	10min
11356841	NRVM+AdGATA4	none	GATA4	1	3hr
11356841	NRVM+AdGATA4	PE	GATA4	2.1	3hr
24152730	NRCM	none	HDAC4 mRNA expression	5	24hr
24152730	NRCM	Ang II	HDAC4 mRNA expression	1.5	24hr
24152730	NRCM	none	HDAC5 mRNA expression	7	24hr
24152730	NRCM	Ang II	HDAC5 mRNA expression	2	24hr
24152730	NRCM	none	HDAC4 protein expression	12	24hr
24152730	NRCM	Ang II	HDAC4 protein expression	4	24hr
24152730	NRCM	none	HDAC5 protein expression	13	24hr

24152730	NRCM	Ang II	HDAC5 protein expression	3	24hr
24152730	NRCM	none	GATA4 protein expression	100	24hr
24152730	NRCM	Ang II	GATA4 protein expression	330*	24hr
24152730	NRCM	none	GATA4 mRNA expression	1	24hr
24152730	NRCM	Ang II	GATA4 mRNA expression	3.3*	24hr
24152730	NRCM	none	GATA4 transcriptional activity	1	24hr
24152730	NRCM	Ang II	GATA4 transcriptional activity	2.8*	24hr
12177418	Ventricular cardiomyocyte	none	nuclear localization of NFAT	23	48hr
12177418	Ventricular cardiomyocyte	PE	nuclear localization of NFAT	50	48hr
12494267	Neonatal rat cardiomyocytes	100nM Ang2	ERK12	100	5min
12494267	Neonatal rat cardiomyocytes	100nM Ang2	ERK12	45	30min
12494267	Neonatal rat cardiomyocytes	100nM Ang2	ERK12	27.5	60min
12494267	Neonatal rat cardiomyocytes	100nM Ang2	ERK12	7.5	120min
9751683	ARVM(adult rat ventricular myocytes)	control	Apoptosis	10	24h
9751683	ARVM	NE(10microM/L)	Apoptosis	36	24h
9751683	ARVM	control	Apoptosis	7	24h
9751683	ARVM	ISO(10mciroM/L)	Apoptosis	14.6	24h
8921810	Cardiomyocytes	NE (1*10^-5 mol/L)	MAPK	1	0min
8921810	Cardiomyocytes	NE (1*10^-5 mol/L)	MAPK	5	2min
8921810	Cardiomyocytes	NE (1*10^-5 mol/L)	MAPK	11.4	5min
8921810	Cardiomyocytes	NE (1*10^-5 mol/L)	MAPK	8	15min
8921810	Cardiomyocytes	NE (1*10^-5 mol/L)	MAPK	5.75	30min
8921810	Cardiomyocytes	NE (1*10^-5 mol/L)	MAPK	4.8	60min
9363896	HEK293	none	pMAPK	1	5min
9363896	HEK293	Isoprenaline(10^-10)	pMAPK	1,2	5min
9363896	HEK293	Isoprenaline(10^-9)	pMAPK	2	5min

9363896	HEK293	Isoprenaline(10^-8)	pMAPK	4	5min
9363896	HEK293	Isoprenaline(10^-7)	pMAPK	5.5	5min
9363896	HEK293	Isoprenaline(10^-6)	pMAPK	6	5min
9363896	HEK293	Isoprenaline(10^-5)	pMAPK	5.8	5min
15964981	Adult cardiomyocyte(Rat)	control	CaMKII activation	100	15min
15964981	Adult cardiomyocyte(Rat)	phe(0.1uM)	CaMKII activation	130	15min
15964981	Adult cardiomyocyte(Rat)	phe(1uM)	CaMKII activation	170	15min
15964981	Adult cardiomyocyte(Rat)	phe(10uM)	CaMKII activation	190	15min
15964981	Adult cardiomyocyte(Rat)	phe(100uM)	CaMKII activation	185	15min
15572667	control mouse	none	p38 kinase activity	1	0min
15572667	control	phenylephrine	p38 kinase activity	1.6	30min
15572667	p38aCKO(10wk)	none	p38 kinase activity	0.25	0min
15572667	p38aCKO(10wk)	phenylephrine	p38 kinase activity	0.4	30min
15572667	control	saline	TUNEL(+) myocytes	23	NA
15572667	control	isoproterenol(7.5mg/kg/day for 2days)	TUNEL(+) myocytes	26	NA
15572667	p38aCKO(10wk)	saline	TUNEL(+) myocytes	25	NA
15572667	p38aCKO(10wk)	isoproterenol(7.5mg/kg/day for 2days)	TUNEL(+) myocytes	50	NA
12177418	Ventricular cardiomyocyte	none	cell size	800	48hr
12177418	Ventricular cardiomyocyte	PE	cell size	1200	48hr
11435346	NRVM	none	ERK1/2	0.1	NA
11435346	NRVM+thapsigargin/nifedipine/EGTA pretx	none	ERK1/2	0.1/0.1/0.1	NA
11435346	NRVM	Iso	ERK1/2	1	NA
11435346	NRVM+thapsigargin/nifedipine/EGTA pretx	Iso	ERK1/2	0.5/0.2/0.2	NA
11435346	NRVM	none	ERK1/2	0.1	8min
11435346	NRVM	AngII	ERK1/2	0.4	8min
11435346	NRVM	PHE	ERK1/2	2	8min
11435346	NRVM	Iso(10uM)	calcineurin	100	0min

11435346	NRVM	Iso(10uM)	calcineurin	120	1min
11435346	NRVM	Iso(10uM)	calcineurin	180	2min
11435346	NRVM	Iso(10uM)	calcineurin	260	5min
11435346	NRVM	Iso(10uM)	calcineurin	150	15min
11435346	NRVM	Iso(10uM)	calcineurin	130	30min
11435346	NRVM	Iso(0uM)	calcineurin	100	5min
11435346	NRVM	Iso(0.01uM)	calcineurin	180	5min
11435346	NRVM	Iso(0.1uM)	calcineurin	190	5min
11435346	NRVM	Iso(1uM)	calcineurin	200	5min
11435346	NRVM	Iso(10uM)	calcineurin	310	5min
11435346	NRVM	Iso(100uM)	calcineurin	300	5min
11435346	NRVM	Iso	Raf-1	0.1	0min
11435346	NRVM	Iso	Raf-1	0.5	1min
11435346	NRVM	Iso	Raf-1	1	2min
11435346	NRVM	Iso	Raf-1	1	5min
11435346	NRVM	Iso	Raf-1	1	10min
11435346	NRVM	Iso	Raf-1	0.5	15min
24248367	NRVM	control	PDE3A1	0.1	NA
24248367	NRVM	Iso	PDE3A1	1	NA
23933582	ARVM	control	calcium(Fura2 ratio)	20	NA
23933582	ARVM	Iso	calcium(Fura2 ratio)	100	NA
11799083	NRVM	PE	AP-1 DNA binding activity	1	0hr
11799083	NRVM	PE	AP-1 DNA binding activity	4.8	3hr
11799083	NRVM	PE	AP-1 DNA binding activity	4	6hr
11799083	NRVM	PE	AP-1 DNA binding activity	3.8	12hr
11799083	NRVM	PE	AP-1 DNA binding activity	2.1	24hr
9584192	myocyte+GST-ATF2	none	p38 activity	0	0min
9584192	myocyte+GST-ATF2	PE	p38 activity	7	15min
9584192	myocyte+GST-ATF2	PE	p38 activity	10	30min
9584192	myocyte+GST-ATF2	PE	p38 activity	3	60min

9584192	myocyte+GST-ATF2	PE	p38 activity	2	120min
9584192	myocyte+GST-c-Jun	none	JNK activity	0	0min
9584192	myocyte+GST-c-Jun	PE	JNK activity	10	15min
9584192	myocyte+GST-c-Jun	PE	JNK activity	13	30min
9584192	myocyte+GST-c-Jun	PE	JNK activity	5	60min
9584192	myocyte+GST-c-Jun	PE	JNK activity	3	120min
9584192	myocyte+MBP	none	ERK activity	0	0min
9584192	myocyte+MBP	PE	ERK activity	4	15min
9584192	myocyte+MBP	PE	ERK activity	2	30min
9584192	myocyte+MBP	PE	ERK activity	1	60min
9584192	myocyte+MBP	PE	ERK activity	1	120min
15367659	NRVM+Ad GFP-HDAC5	none	HDAC5 nuclear localization	750	2hr
15367659	NRVM+Ad GFP-HDAC5	PE	HDAC5 nuclear localization	150	2hr
20362664	NRVM serum-starved (24h)	ISO	RGS expression/18s rRNA	4.5	0.5h
20362664	NRVM serum-starved (24h)	ISO	RGS expression/18s rRNA	8	1h
20362664	NRVM serum-starved (24h)	ISO	RGS expression/18s rRNA	4	2h
20362664	NRVM serum-starved (24h)	ISO	RGS expression/18s rRNA	2.5	6h
20362664	NRVM serum-starved (24h)	ISO	RGS expression/18s rRNA	1	24h

Supplementray Table S5. Verification results in the cardiac signaling network.

	Lin	Hill	Sat	Acc	Combination*
Increase of RGS4	0.999999	0.618143	0.999986	0.999995	0.849005
Increase of Ca	0.975967	0.60222	0.944983	0.994678	0.841005
Increase of PDE34	0.992181	0.622474	0.984437	0.997869	0.811249
Increase of Raf1	0.798534	0.511632	0.85876	0.700121	0.691984
Increase of CaN	0.975967	0.602289	0.944971	0.994657	0.842722
Increase of JNK	0.903222	0.521664	0.881672	0.921375	0.801478
Increase of p38	0.891231	0.504119	0.912041	0.862816	0.816612
Increase of CaMK	0.978696	0.606074	0.95267	0.995022	0.835570
Increase of ERK12	0.994433	0.616307	0.997128	0.990608	0.833709
Increase of NFATnuc	0.746256	0.525368	0.595141	0.9129	0.676622
Decrease of HDAC	0.872288	0.945868	0.915132	0.793033	0.864994
Increase of MSK1	0.988983	0.588751	0.996149	0.984066	0.831351
Increase of CREB	0.926242	0.48057	0.921248	0.929137	0.810174
Increase of MEF2	0.921411	0.559428	0.956791	0.840387	0.829389
Increase of GATA4	0.99415	0.606909	0.997398	0.993794	0.868812
Increase of cJun	0.911455	0.520087	0.914718	0.911178	0.827229

Effect of catecholamine on the distribution of the 16 network components evaluated using each function type separately or using different types of response functions in a combined manner.

* Results of the mathematical simulation analysis performed using the combination of the four different response functions

Supplementray Table S6. Results of the one-distribution perturbation analyses in the cardiac signaling network.

1) Result of one-distribution perturbation analysis for apoptosis or hypertrophy when each response function was separately applied

			Lin		Hill		Sat		Acc	
			Effect	p-value	Effect	p-value	Effect	p-value	Effect	p-value
Threshold: top 5%	Apoptosis	pm7	2.069	<0.001	1.646	<0.001	2.483	<0.001	1.871	<0.001
		pm10	2.140	<0.001	1.905	<0.001	1.933	<0.001	2.221	<0.001
		pm12	1.154	0.024	1.236	<0.001	1.169	0.021	1.254	<0.001
		pm21	1.163	0.023	1.273	<0.001	1.484	<0.001	1.167	0.021
		pm28	1.335	<0.001	1.162	0.027	1.162	0.021	1.450	<0.001
			Lin		Hill		Sat		Acc	
			Effect	p-value	Effect	p-value	Effect	p-value	Effect	p-value
	Hypertrophy	pm7	3.115	<0.001	2.285	<0.001	3.306	<0.001	2.322	<0.001
		pm10	1.557	<0.001	1.542	<0.001	1.367	<0.001	1.692	<0.001
		pm15	1.151	0.021	1.267	<0.001	1.162	0.024	1.169	0.020
		pm16	1.148	0.022	1.193	0.008	1.218	<0.001	1.178	0.010
		pm17	1.148	0.022	1.170	0.009	1.196	0.007	1.154	0.020
		pm21	1.626	<0.001	1.143	0.033	1.873	<0.001	1.207	<0.001
			Lin		Hill		Sat		Acc	
			Effect	p-value	Effect	p-value	Effect	p-value	Effect	p-value
Threshold: top 20%	Apoptosis	pm7	1.907	<0.001	1.494	<0.001	2.074	<0.001	1.583	<0.001
		pm10	2.002	<0.001	1.639	<0.001	1.901	<0.001	2.127	<0.001
		pm12	1.122	0.033	1.161	0.014	1.125	0.038	1.227	<0.001

		pm21	1.117	0.038	1.251	<0.001	1.338	<0.001	1.147	0.035
		pm28	1.249	<0.001	1.117	0.042	1.130	0.034	1.372	<0.001
	Hypertrophy		Lin		Hill		Sat		Acc	
			Effect	p-value	Effect	p-value	Effect	p-value	Effect	p-value
		pm7	2.654	<0.001	1.920	<0.001	2.509	<0.001	2.203	<0.001
		pm10	1.411	<0.001	1.505	<0.001	1.252	<0.001	1.500	<0.001
		pm15	1.134	0.040	1.223	<0.001	1.135	0.035	1.139	0.031
		pm16	1.148	0.022	1.137	0.028	1.166	0.015	1.151	0.020
		pm17	1.127	0.041	1.156	0.030	1.173	0.011	1.130	0.037
		pm21	1.475	<0.001	1.122	0.042	1.748	<0.001	1.176	0.013

Results of one-distribution perturbation analysis for apoptosis and hypertrophy when the threshold for determining the marginal distributions was set to top 5% or 20%. The effect is represented as the ratio between the degree of appearance of phenotypes in the one-distribution perturbation analysis and that in the control distributions. Parameters of which the marginal distributions significantly ($p<0.05$) increased apoptosis or hypertrophy in all response function types are shown. The mathematical analysis was all repeated for 10 times using different random seeds of 1 million parameter sets for each case. P-values were determined by comparison with the control distributions using Student's *t* test. See Supplementary Data Sets for full data.

2) Result of reverse one-distribution perturbation analysis for apoptosis or hypertrophy when each response function was separately applied

Threshold	Phenotype		Lin		Hill		Sat		Acc	
			Effect	p-value	Effect	p-value	Effect	p-value	Effect	p-value
Threshold: top 5%	Apoptosis	pm7	0.012	<0.001	0.214	<0.001	0.024	<0.001	0.130	<0.001
		pm10	0.158	<0.001	0.376	<0.001	0.180	<0.001	0.050	<0.001
		pm12	0.816	0.009	0.743	<0.001	0.761	<0.001	0.694	<0.001
		pm23	0.816	0.009	0.614	<0.001	0.714	<0.001	0.784	<0.001
		pm28	0.671	<0.001	0.728	<0.001	0.789	<0.001	0.529	<0.001
		pm30	0.714	<0.001	0.470	<0.001	0.826	0.017	0.633	<0.001
	Hypertrophy		Lin		Hill		Sat		Acc	
			Effect	p-value	Effect	p-value	Effect	p-value	Effect	p-value
		pm7	0.005	<0.001	0.119	<0.001	0.001	<0.001	0.047	<0.001
		pm10	0.509	<0.001	0.443	<0.001	0.647	<0.001	0.322	<0.001
		pm16	0.498	<0.001	0.417	<0.001	0.481	<0.001	0.585	<0.001
		pm17	0.464	<0.001	0.385	<0.001	0.419	<0.001	0.699	<0.001
		pm35	0.873	0.030	0.917	0.043	0.897	0.045	0.638	<0.001
Threshold: top 20%	Apoptosis		Lin		Hill		Sat		Acc	
			Effect	p-value	Effect	p-value	Effect	p-value	Effect	p-value
		pm7	0.302	<0.001	0.536	<0.001	0.255	<0.001	0.387	<0.001
		pm10	0.274	<0.001	0.431	<0.001	0.394	<0.001	0.290	<0.001
		pm12	0.888	0.032	0.829	0.011	0.897	0.038	0.754	<0.001
		pm23	0.890	0.039	0.729	<0.001	0.863	0.011	0.897	0.042

		pm28	0.788	<0.001	0.895	0.040	0.847	0.011	0.700	<0.001
		pm30	0.790	<0.001	0.680	<0.001	0.887	0.041	0.715	<0.001
	Hypertrophy		Lin		Hill		Sat		Acc	
			Effect	p-value	Effect	p-value	Effect	p-value	Effect	p-value
		pm7	0.100	<0.001	0.299	<0.001	0.191	<0.001	0.195	<0.001
		pm10	0.669	<0.001	0.604	<0.001	0.781	<0.001	0.551	<0.001
		pm16	0.588	<0.001	0.446	<0.001	0.620	<0.001	0.670	<0.001
		pm17	0.648	<0.001	0.499	<0.001	0.616	<0.001	0.754	<0.001
		pm35	0.901	0.040	0.884	0.041	0.898	0.031	0.741	<0.001

Results of reverse one-distribution perturbation analysis for apoptosis and hypertrophy when the threshold for determining the marginal distributions was set to top 5% or 20%. The effect is represented as the ratio between the degree of appearance of phenotypes in the one-distribution perturbation analysis and that in the control distributions. Parameters of which the marginal distributions significantly ($p<0.05$) decreased apoptosis or hypertrophy in all response function types are shown. The mathematical analysis was all repeated for 10 times using different random seeds of 1 million parameter sets for each case. P-values were determined by comparison with the control distributions using Student's *t* test. See Supplementary Data Sets for full data.

3) Result of one-distribution perturbation analysis when four different response function types were applied in a combined manner

Analysis	Phenotype		Top 5%		Top 10%		Top 20%	
			Effect	p-value	Effect	p-value	Effect	p-value
One-distribution perturbation analysis	Apoptosis	pm7	1.888	<0.001	1.700	<0.001	1.385	<0.001
		pm10	1.876	<0.001	1.667	<0.001	1.597	<0.001
		pm12	1.349	<0.001	1.182	0.012	1.176	0.012
		pm21	1.397	<0.001	1.214	<0.001	1.174	0.013
		pm28	1.332	<0.001	1.188	0.012	1.166	0.028
	Hypertrophy		Top 5%		Top 10%		Top 20%	
			Effect	p-value	Effect	p-value	Effect	p-value
		pm7	2.412	<0.001	2.350	<0.001	1.955	<0.001
		pm10	1.397	<0.001	1.334	<0.001	1.247	<0.001
		pm15	1.291	<0.001	1.175	0.010	1.165	0.012
		pm16	1.259	<0.001	1.220	<0.001	1.146	0.022
		pm17	1.191	0.006	1.153	0.018	1.142	0.025
		pm21	1.438	<0.001	1.396	<0.001	1.292	<0.001
Reverse one-distribution perturbation analysis	Apoptosis		Top 5%		Top 10%		Top 20%	
			Effect	p-value	Effect	p-value	Effect	p-value
		pm7	0.192	<0.001	0.413	<0.001	0.497	<0.001
		pm10	0.338	<0.001	0.391	<0.001	0.518	<0.001
		pm12	0.775	<0.001	0.853	0.028	0.891	0.040
		pm23	0.797	<0.001	0.823	0.016	0.880	0.018
		pm28	0.778	<0.001	0.812	0.010	0.849	0.023
		pm30	0.782	<0.001	0.822	0.011	0.848	0.022

			Top 5%		Top 10%		Top 20%	
			Effect	p-value	Effect	p-value	Effect	p-value
	Hypertrophy	pm7	0.177	<0.001	0.269	<0.001	0.503	<0.001
		pm10	0.685	<0.001	0.759	<0.001	0.795	<0.001
		pm16	0.634	<0.001	0.693	<0.001	0.747	<0.001
		pm17	0.699	<0.001	0.733	<0.001	0.778	<0.001
		pm35	0.718	<0.001	0.827	0.007	0.896	0.034

Results of one-distribution perturbation analysis for apoptosis and hypertrophy when four different response function types were applied in a combined manner. The effect is represented as the ratio between the degree of appearance of phenotypes in one-distribution perturbation analysis and that in the control distributions. Threshold for determining the marginal distributions was set to top 5%, 10%, or 20%. Parameters of which the marginal distributions significantly ($p<0.05$) increased apoptosis or hypertrophy are shown. The mathematical analysis was all repeated for 10 times using different random seeds of 1 million parameter sets for each case. P-values were determined by comparison with the control distributions using Student's *t* test. See Supplementary Data Sets for full data.

Supplementray Table S7. Results of two-distribution perturbation analysis when four different response function types were applied in a combined manner

	Pair of perturbed parameter distributions	1 million parameter sets		10 million parameter sets		100 million parameter sets	
		Synergistic effect	p-value	Synergistic effect	p-value	Synergistic effect	p-value
Apoptosis	pm1-pm10	0.11	0.006	0.125	0.008	0.108	0.007
	pm2-pm10	0.113	0.009	0.096	0.017	0.117	0.010
	pm3-pm10	0.101	0.006	0.103	0.008	0.111	0.009
	pm6-pm10	0.196	0.004	0.211	<0.001	0.185	0.002
	pm7-pm10	0.482	<0.001	0.463	<0.001	0.454	<0.001
	pm7-pm30	0.09	0.019	0.078	0.015	0.079	0.011
	pm8-pm10	0.097	0.015	0.108	0.010	0.12	0.009
	pm9-pm10	0.113	0.009	0.098	0.018	0.115	0.007
	pm10-pm11	0.149	0.005	0.163	0.002	0.169	0.004
	pm10-pm13	0.119	0.007	0.107	0.005	0.128	0.009
	pm10-pm14	0.091	0.013	0.093	0.011	0.108	0.009
	pm10-pm15	0.065	0.026	0.071	0.013	0.07	0.032
	pm10-pm17	0.091	0.014	0.084	0.010	0.082	0.018
	pm10-pm19	0.09	0.019	0.086	0.014	0.104	0.010
	pm10-pm20	0.092	0.012	0.091	0.014	0.105	0.006
	pm10-pm26	0.066	0.031	0.068	0.031	0.073	0.011
	pm10-pm30	0.118	0.008	0.112	0.006	0.138	0.008
	pm10-pm32	0.08	0.014	0.09	0.015	0.081	0.017
	pm10-pm33	0.057	0.029	0.066	0.026	0.058	0.029

	Pair of perturbed parameter distributions	1 million parameter sets		10 million parameter sets		100 million parameter sets	
		Synergistic effect	p-value	Synergistic effect	p-value	Synergistic effect	p-value
	pm10-pm34	0.171	0.003	0.165	0.004	0.185	0.004
	pm10-pm35	0.109	0.009	0.109	0.007	0.094	0.015
	pm10-pm36	0.054	0.039	0.054	0.037	0.068	0.039
	pm10-pm37	0.142	0.008	0.128	0.008	0.134	0.008
	pm22-pm23	0.164	0.003	0.153	0.004	0.163	0.004
Hypertrophy	Pair of perturbed parameter distributions	1 million parameter sets		10 million parameter sets		100 million parameter sets	
		Synergistic effect	p-value	Synergistic effect	p-value	Synergistic effect	p-value
	pm7-pm10	0.379	<0.001	0.361	<0.001	0.333	<0.001
	pm7-pm13	0.195	0.005	0.167	0.007	0.175	0.003
	pm7-pm14	0.147	0.007	0.141	0.007	0.127	0.007
	pm7-pm21	0.405	<0.001	0.434	<0.001	0.399	<0.001
	pm10-pm14	0.144	0.009	0.166	0.003	0.175	0.017
	pm16-pm17	0.235	<0.001	0.256	<0.001	0.222	0.001

Results of two-distribution perturbation analysis for apoptosis and hypertrophy when four different response function types were applied in a combined manner. The synergistic effect was calculated as the difference between the effect of simultaneous perturbation of marginal distributions of two parameters on the phenotype and the sum of that obtained from perturbing either individual marginal distribution. Higher values indicate stronger synergistic effect. Parameter pairs exhibiting synergistic effect for apoptosis or hypertrophy with significance ($p<0.05$) are shown. The mathematical analysis was all repeated for 10 times using different random seeds of 1, 10, or 100 million parameter sets for each case. P-values were determined using Student's *t* test. See Supplementary Data Sets for full data.

Table S8. Differential equations of the mathematical model of 16 network motifs.

Network motif	Differential equations
Type 1 negative feedback (motif 1)	$\frac{dX}{dt} = p_2 \times f_{inh}(Z) + (1 - p_1 - p_2) \times f_{act}(Input) + p_1 \times f_{inh}(Z) \times f_{act}(Input) - X$<br>$\frac{dY}{dt} = f_{act}(X) - Y$<br>$\frac{dZ}{dt} = f_{act}(Y) - Z$
Type 2 negative feedback (motif 2)	$\frac{dX}{dt} = p_2 \times f_{act}(Z) + (1 - p_1 - p_2) \times f_{act}(Input) + p_1 \times f_{act}(Z) \times f_{act}(Input) - X$<br>$\frac{dY}{dt} = f_{act}(X) - Y$<br>$\frac{dZ}{dt} = f_{inh}(Y) - Z$
Type 3 negative feedback (motif 3)	$\frac{dX}{dt} = p_2 \times f_{act}(Z) + (1 - p_1 - p_2) \times f_{act}(Input) + p_1 \times f_{act}(Z) \times f_{act}(Input) - X$<br>$\frac{dY}{dt} = f_{inh}(X) - Y$<br>$\frac{dZ}{dt} = f_{act}(Y) - Z$

Type 4 negative feedback (motif 4)	$\frac{dX}{dt} = p_2 \times f_{inh}(Z) + (1 - p_1 - p_2) \times f_{act}(Input) + p_1 \times f_{inh}(Z) \times f_{act}(Input) - X$<br>$\frac{dY}{dt} = f_{inh}(X) - Y$<br>$\frac{dZ}{dt} = f_{inh}(Y) - Z$
Type 1 positive feedback (motif 5)	$\frac{dX}{dt} = p_2 \times f_{act}(Z) + (1 - p_1 - p_2) \times f_{act}(Input) + p_1 \times f_{act}(Z) \times f_{act}(Input) - X$<br>$\frac{dY}{dt} = f_{inh}(X) - Y$<br>$\frac{dZ}{dt} = f_{inh}(Y) - Z$
Type 2 positive feedback (motif 6)	$\frac{dX}{dt} = p_2 \times f_{inh}(Z) + (1 - p_1 - p_2) \times f_{act}(Input) + p_1 \times f_{inh}(Z) \times f_{act}(Input) - X$<br>$\frac{dY}{dt} = f_{inh}(X) - Y$<br>$\frac{dZ}{dt} = f_{act}(Y) - Z$
Type 3 positive feedback (motif 7)	$\frac{dX}{dt} = p_2 \times f_{inh}(Z) + (1 - p_1 - p_2) \times f_{act}(Input) + p_1 \times f_{inh}(Z) \times f_{act}(Input) - X$<br>$\frac{dY}{dt} = f_{act}(X) - Y$<br>$\frac{dZ}{dt} = f_{inh}(Y) - Z$

Type 4 positive feedback (motif 8)	$\frac{dX}{dt} = p_2 \times f_{act}(Z) + (1 - p_1 - p_2) \times f_{act}(Input) + p_1 \times f_{act}(Z) \times f_{act}(Input) - X$<br>$\frac{dY}{dt} = f_{act}(X) - Y$<br>$\frac{dZ}{dt} = f_{act}(Y) - Z$
Type 1 coherent feedforward (motif 9)	$\frac{dX}{dt} = f_{act}(Input) - X$<br>$\frac{dY}{dt} = f_{act}(X) - Y$<br>$\frac{dZ}{dt} = p_2 \times f_{act}(Y) + (1 - p_1 - p_2) \times f_{act}(X) + p_1 \times f_{act}(Y) \times f_{act}(X) - Z$
Type 2 coherent feedforward (motif 10)	$\frac{dX}{dt} = f_{act}(Input) - X$<br>$\frac{dY}{dt} = f_{inh}(X) - Y$<br>$\frac{dZ}{dt} = p_2 \times f_{act}(Y) + (1 - p_1 - p_2) \times f_{inh}(X) + p_1 \times f_{act}(Y) \times f_{inh}(X) - Z$
Type 3 coherent feedforward (motif 11)	$\frac{dX}{dt} = f_{act}(Input) - X$<br>$\frac{dY}{dt} = f_{act}(X) - Y$<br>$\frac{dZ}{dt} = p_2 \times f_{inh}(Y) + (1 - p_1 - p_2) \times f_{inh}(X) + p_1 \times f_{inh}(Y) \times f_{inh}(X) - Z$

Type 4 coherent feedforward (motif 12)	$\frac{dX}{dt} = f_{act}(Input) - X$<br>$\frac{dY}{dt} = f_{inh}(X) - Y$<br>$\frac{dZ}{dt} = p_2 \times f_{inh}(Y) + (1 - p_1 - p_2) \times f_{act}(X) + p_1 \times f_{inh}(Y) \times f_{act}(X) - Z$
Type 1 incoherent feedforward (motif 13)	$\frac{dX}{dt} = f_{act}(Input) - X$<br>$\frac{dY}{dt} = f_{act}(X) - Y$<br>$\frac{dZ}{dt} = p_2 \times f_{inh}(Y) + (1 - p_1 - p_2) \times f_{act}(X) + p_1 \times f_{inh}(Y) \times f_{act}(X) - Z$
Type 2 incoherent feedforward (motif 14)	$\frac{dX}{dt} = f_{act}(Input) - X$<br>$\frac{dY}{dt} = f_{inh}(X) - Y$<br>$\frac{dZ}{dt} = p_2 \times f_{inh}(Y) + (1 - p_1 - p_2) \times f_{inh}(X) + p_1 \times f_{inh}(Y) \times f_{inh}(X) - Z$
Type 3 incoherent feedforward (motif 15)	$\frac{dX}{dt} = f_{act}(Input) - X$<br>$\frac{dY}{dt} = f_{act}(X) - Y$<br>$\frac{dZ}{dt} = p_2 \times f_{act}(Y) + (1 - p_1 - p_2) \times f_{inh}(X) + p_1 \times f_{act}(Y) \times f_{inh}(X) - Z$

Type 4 incoherent feedforward (motif 16)	$\frac{dX}{dt} = f_{act}(Input) - X$<br>$\frac{dY}{dt} = f_{inh}(X) - Y$<br>$\frac{dZ}{dt} = p_2 \times f_{act}(Y) + (1 - p_1 - p_2) \times f_{act}(X) + p_1 \times f_{act}(Y) \times f_{act}(X) - Z$

Supplementray Table S9. Association of the network motifs and the dynamical features.

	Negative feedback	Positive feedback	Coherent feedforward	Incoherent feedforward
Adaptation	+++	+	-	+++
Memory	-	++	-	-
Consistency	-	-	+++	-
Pulsation	-	-	-	+++

+++: dynamics are observed significantly in all types of motifs regardless of the form of response function, ++: dynamics are observed significantly in some types of motifs, +: dynamics are observed weakly in all types of motifs, - : no such dynamics observed

Supplementray Table S10. Dynamical features of the 16 network motifs.

1) Dynamical features of network motif: adaptation

	Lin	Hill	Sat	Acc
Motif1	0.211±0.061	0.224±0.079	0.205±0.062	0.218±0.059
Motif2	0.211±0.061	0.228±0.076	0.206±0.062	0.211±0.061
Motif3	0.210±0.062	0.230±0.080	0.212±0.061	0.208±0.062
Motif4	0.210±0.062	0.228±0.083	0.208±0.062	0.212±0.061
Motif5	0.062±0.073	0.015±0.030	0.053±0.061	0.082±0.088
Motif6	0.075±0.079	0.043±0.153	0.087±0.087	0.073±0.077
Motif7	0.059±0.073	0.161±0.359	0.059±0.069	0.079±0.093
Motif8	0.042±0.068	0.009±0.021	0.057±0.068	0.118±0.094
Motif9	0.001±0.000	0.001±0.000	0.001±0.000	0.001±0.000
Motif10	0.001±0.000	0.001±0.000	0.001±0.000	0.001±0.000
Motif11	0.001±0.000	0.001±0.000	0.001±0.000	0.001±0.000
Motif12	0.001±0.000	0.001±0.001	0.001±0.000	0.001±0.000
Motif13	0.206±0.074	0.208±0.076	0.201±0.071	0.212±0.079
Motif14	0.172±0.069	0.172±0.069	0.174±0.069	0.171±0.070
Motif15	0.172±0.069	0.173±0.069	0.171±0.070	0.173±0.069
Motif16	0.206±0.074	0.206±0.074	0.213±0.080	0.199±0.070

2) Dynamical features of network motif: pulsation

	Lin	Hill	Sat	Acc
Motif1	0.0000±0.0000	0.0000±0.0000	0.0000±0.0000	0.0000±0.0000
Motif2	0.0000±0.0000	0.0000±0.0000	0.0000±0.0000	0.0000±0.0000
Motif3	0.0000±0.0000	0.0000±0.0000	0.0000±0.0000	0.0000±0.0000
Motif4	0.0000±0.0000	0.0000±0.0000	0.0000±0.0000	0.0000±0.0000

Motif5	0.0000±0.0000	0.0000±0.0000	0.0000±0.0000	0.0000±0.0000
Motif6	0.0000±0.0000	0.0000±0.0000	0.0000±0.0000	0.0000±0.0000
Motif7	0.0000±0.0000	0.0000±0.0000	0.0000±0.0000	0.0000±0.0000
Motif8	0.0000±0.0000	0.0000±0.0000	0.0000±0.0000	0.0000±0.0000
Motif9	0.0000±0.0000	0.0000±0.0000	0.0000±0.0000	0.0000±0.0000
Motif10	0.0000±0.0000	0.0000±0.0000	0.0000±0.0000	0.0000±0.0000
Motif11	0.0000±0.0000	0.0000±0.0000	0.0000±0.0000	0.0000±0.0000
Motif12	0.0000±0.0000	0.0000±0.0000	0.0000±0.0000	0.0000±0.0000
Motif13	0.0009±0.0026	0.0016±0.0041	0.0009±0.0027	0.0008±0.0024
Motif14	0.0009±0.0026	0.0016±0.0041	0.0010±0.0028	0.0007±0.0022
Motif15	0.0009±0.0026	0.0020±0.0044	0.0008±0.0024	0.0009±0.0027
Motif16	0.0009±0.0026	0.0020±0.0044	0.0007±0.0022	0.0010±0.0028

3) Dynamical features of network motif: memory

	Lin	Hill	Sat	Acc
Motif1	0.000±0.001	0.002±0.004	0.000±0.001	0.000±0.001
Motif2	0.001±0.001	0.001±0.002	0.001±0.001	0.001±0.001
Motif3	0.001±0.001	0.002±0.003	0.001±0.001	0.001±0.001
Motif4	0.000±0.000	0.001±0.001	0.000±0.000	0.000±0.000
Motif5	0.044±0.057	0.000±0.000	0.052±0.063	0.033±0.043
Motif6	0.072±0.098	0.017±0.070	0.051±0.072	0.084±0.099
Motif7	0.039±0.054	0.000±0.000	0.060±0.073	0.021±0.031
Motif8	0.012±0.015	0.000±0.000	0.068±0.111	0.002±0.003
Motif9	0.000±0.000	0.000±0.000	0.000±0.000	0.000±0.000
Motif10	0.000±0.000	0.001±0.000	0.000±0.000	0.000±0.000
Motif11	0.000±0.001	0.000±0.000	0.000±0.000	0.000±0.000
Motif12	0.000±0.000	0.000±0.000	0.000±0.000	0.000±0.000
Motif13	0.000±0.000	0.000±0.000	0.000±0.000	0.000±0.000

Motif14	0.000±0.000	0.000±0.000	0.000±0.000	0.001±0.001
Motif15	0.000±0.000	0.000±0.000	0.000±0.000	0.000±0.000
Motif16	0.000±0.000	0.000±0.000	0.000±0.000	0.000±0.000

4) Dynamical features of network motif: consistency

	Lin	Hill	Sat	Acc
Motif1	0.036	0.026	0.033	0.041
Motif2	0.037	0.027	0.031	0.044
Motif3	0.037	0.027	0.036	0.038
Motif4	0.037	0.026	0.038	0.035
Motif5	0.022	0.005	0.038	0.012
Motif6	0.023	0.005	0.012	0.038
Motif7	0.022	0.005	0.034	0.011
Motif8	0.020	0.005	0.019	0.010
Motif9	3.429	3.767	3.341	3.339
Motif10	2.436	2.619	2.577	2.226
Motif11	3.424	3.789	3.291	3.384
Motif12	2.474	2.669	2.588	2.285
Motif13	0.009	0.009	0.009	0.009
Motif14	0.009	0.009	0.009	0.009
Motif15	0.009	0.009	0.009	0.009
Motif16	0.009	0.009	0.009	0.009

Supplementray Table S11. Results of the distribution perturbation analysis of the 16 network motifs.

1) Result of distribution perturbation analysis: association 1 (adaptation-negative feedback)

	Uniform	1 marginal (pm1)	1 marginal (pm2)	2 marginal (pm1, pm2)
Type 1 - Lin	0.1	0.392	0.353	0.772
Type 1 - Hill	0.1	0.415	0.362	0.765
Type 1 - Sat	0.1	0.401	0.335	0.748
Type 1 - Acc	0.1	0.388	0.329	0.76
Type 2 - Lin	0.1	0.498	0.18	0.567
Type 2 - Hill	0.1	0.483	0.16	0.554
Type 2 - Sat	0.1	0.52	0.207	0.647
Type 2 - Acc	0.1	0.516	0.205	0.623
Type 3 - Lin	0.1	0.392	0.332	0.721
Type 3 - Hill	0.1	0.385	0.3	0.703
Type 3 - Sat	0.1	0.444	0.3	0.715
Type 3 - Acc	0.1	0.361	0.337	0.778
Type 4 - Lin	0.1	0.364	0.349	0.761
Type 4 - Hill	0.1	0.323	0.401	0.824
Type 4 - Sat	0.1	0.38	0.352	0.793
Type 4 - Acc	0.1	0.425	0.287	0.704

2) Result of distribution perturbation analysis: association 1 (memory-positive feedback)

	Uniform	1 marginal (pm1)	1 marginal (pm2)	2 marginal (pm1, pm2)
Type 1 - Lin	0.1	0.228	0.808	0.851
Type 1 - Hill	0.1	0.227	0.816	0.85
Type 1 - Sat	0.1	0.232	0.821	0.862
Type 1 - Acc	0.1	0.209	0.788	0.841

Type 2 - Lin	NA*	NA*	NA*	NA*
Type 2 - Hill	0.1	0.208	0.863	0.906
Type 2 - Sat	NA*	NA*	NA*	NA*
Type 2 - Acc	NA*	NA*	NA*	NA*
Type 3 - Lin	0.1	0.175	0.878	0.879
Type 3 - Hill	0.1	0.203	0.817	0.848
Type 3 - Sat	0.1	0.171	0.912	0.917
Type 3 - Acc	0.1	0.218	0.829	0.862
Type 4 - Lin	0.1	0.212	0.818	0.871
Type 4 - Hill	0.1	0.178	0.926	0.948
Type 4 - Sat	0.1	0.211	0.81	0.861
Type 4 - Acc	0.1	0.209	0.771	0.826

*NA denotes 'not available'

3) Result of distribution perturbation analysis: association 1 (adaptation-incoherent feedforward)

	Uniform	1 marginal (pm1)	1 marginal (pm2)	2 marginal (pm1, pm2)
Type 1 - Lin	0.1	0.357	0.391	0.853
Type 1 - Hill	0.1	0.124	0.114	0.151
Type 1 - Sat	0.1	0.121	0.105	0.144
Type 1 - Acc	0.1	0.354	0.386	0.85
Type 2 - Lin	0.1	0.42	0.322	0.818
Type 2 - Hill	0.1	0.123	0.144	0.166
Type 2 - Sat	0.1	0.108	0.115	0.139
Type 2 - Acc	0.1	0.384	0.33	0.796
Type 3 - Lin	0.1	0.318	0.447	0.874
Type 3 - Hill	0.1	0.124	0.121	0.142
Type 3 - Sat	0.1	0.109	0.11	0.158

Type 3 - Acc	0.1	0.406	0.361	0.848
Type 4 - Lin	0.1	0.383	0.357	0.826
Type 4 - Hill	0.1	0.106	0.124	0.179
Type 4 - Sat	0.1	0.11	0.109	0.129
Type 4 - Acc	0.1	0.289	0.466	0.867

4) Result of distribution perturbation analysis: association 1 (adaptation-negative feedback)

	Uniform	1 marginal (pm1)	1 marginal (pm2)	2 marginal (pm1, pm2)
Type 1 - Lin	0.1	0.158	0.263	0.302
Type 1 - Hill	0.1	0.132	0.287	0.29
Type 1 - Sat	0.1	0.132	0.288	0.323
Type 1 - Acc	0.1	0.13	0.267	0.322
Type 2 - Lin	0.1	0.145	0.256	0.31
Type 2 - Hill	0.1	0.148	0.273	0.318
Type 2 - Sat	0.1	0.119	0.27	0.281
Type 2 - Acc	0.1	0.125	0.234	0.275
Type 3 - Lin	0.1	0.134	0.255	0.311
Type 3 - Hill	0.1	0.131	0.271	0.28
Type 3 - Sat	0.1	0.153	0.295	0.343
Type 3 - Acc	0.1	0.161	0.295	0.345
Type 4 - Lin	0.1	0.146	0.258	0.35
Type 4 - Hill	0.1	0.151	0.294	0.362
Type 4 - Sat	0.1	0.133	0.281	0.333
Type 4 - Acc	0.1	0.138	0.259	0.277

Supplementray Table S12. Differential equations of the EGFR model.

Eq. no.	Differential equation
(1)	$\frac{d[EGFR]}{dt} = Inputs - EGFR$
(2)	$\frac{d[Grb2SoS]}{dt} = pm_{gs1} \times f_{act}(GAB1) + pm_{gs2} \times f_{inh}(ERK) + (add_{gs} - pm_{gs1} - pm_{gs2}) \times f_{act}(EGFR) + pm_{gs12} \times f_{act}(GAB1) \times f_{inh}(ERK) + pm_{gs23} \times f_{inh}(ERK) \times f_{act}(EGFR) + pm_{gs31} \times f_{act}(EGFR) \times f_{act}(GAB1) + pm_{syngs123} \times f_{act}(GAB1) \times f_{inh}(ERK) \times f_{act}(EGFR) - Grb2Sos$
(3)	$\frac{d[Src]}{dt} = f_{act}(EGFR) - Src$
(4)	$\frac{d[PI3K]}{dt} = pm_{pitk1} \times f_{act}(GAB1) + pm_{pitk2} \times f_{act}(Ras) + (add_{pitk} - pm_{pitk1} - pm_{pitk2}) \times f_{act}(EGFR) + pm_{pitk12} \times f_{act}(GAB1) \times f_{act}(Ras) + pm_{pitk23} \times f_{act}(Ras) \times f_{act}(EGFR) + pm_{pitk31} \times f_{act}(EGFR) \times f_{act}(GAB1) + pm_{synpitk123} \times f_{act}(GAB1) \times f_{act}(Ras) \times f_{act}(EGFR) - PI3K$
(5)	$\frac{d[Ras]}{dt} = pm_{ras} \times f_{inh}(RasGAP) + (add_{ras} - pm_{ras}) \times f_{act}(Grb2Sos) + pm_{synras} \times f_{inh}(RasGAP) \times f_{act}(Grb2Sos) - Ras$
(6)	$\frac{d[GAB1]}{dt} = pm_{gab1} \times f_{act}(Src) + pm_{gab2} \times f_{act}(PIP3) + (add_{gab} - pm_{gab1} - pm_{gab2}) \times f_{inh}(ERK) + pm_{gab12} \times f_{act}(Src) \times f_{act}(PIP3) + pm_{gab23} \times f_{act}(PIP3) \times f_{inh}(ERK) + pm_{gab31} \times f_{inh}(ERK) \times f_{act}(Src) + pm_{syngab123} \times f_{act}(Src) \times f_{act}(PIP3) \times f_{inh}(ERK) - GAB1$
(7)	$\frac{d[MEK]}{dt} = f_{act}(Ras) - k_i \times MEK$ *

(8)	$\frac{d[ERK]}{dt} = f_{act}(MEK) - ERK$
(9)	$\frac{d[RasGAP]}{dt} = pm_{rasgap1} \times f_{act}(GAB1) + pm_{rasgap2} \times f_{inh}(SHP2) + (add_{rasgap} - pm_{rasgap1} - pm_{rasgap2}) \times f_{act}(EGFR) + pm_{rasgap12} \times f_{act}(GAB1) \times f_{inh}(SHP2) + pm_{rasgap23} \times f_{inh}(SHP2) \times f_{act}(EGFR) + pm_{rasgap31} \times f_{act}(EGFR) \times f_{act}(GAB1) + pm_{synrasgap123} \times f_{act}(GAB1) \times f_{inh}(SHP2) \times f_{act}(EGFR) - RasGAP$
(10)	$\frac{d[PIP3]}{dt} = f_{act}(PI3K) - PIP3$
(11)	$\frac{d[SHP2]}{dt} = f_{act}(GAB1) - SHP2$
(12)	$\frac{d[PDK1]}{dt} = f_{act}(PIP3) - PDK1$
(13)	$\frac{d[AKT]}{dt} = f_{act}(PDK1) - AKT$

* The effect of the MEK inhibitor is represented by the parameter ki

Supplementray Table S13. Relationship between interactions and parameters in the EGFR model.

Link	Related parameter	Constraints of parameter
Effect of GAB1 on Grb2SoS	pm_{gs1}	pm_{syngs}*+pm_{gs1}*+pm_{gs2}*≤1<br>$0 \le pm_{syngs}, pm_{gs1}, pm_{gs2} \le 1$<br>$add_{syngs}=1-pm_{syngs}$<br>$pm_{gs3}=add_{syngs}-pm_{gs1}-pm_{gs2}$
Effect of ERK on Grb2SoS	pm_{gs2}	
Effect of EGFR on Grb2SoS	pm_{gs3}	
Synergistic effect of GAB1 and ERK on Grb2SoS	pm_{gs12}	$sum_{gs}=pm_{gs1} \times pm_{gs2}+ pm_{gs2} \times pm_{gs3} + pm_{gs3} \times pm_{gs1}$<br>$pm_{syngs123}= pm_{syngs}\times pm_{syngs}$<br>$pm_{gs12}=(pm_{gs1} \times pm_{gs2}/ sum_{gs})\times(pm_{syngs}-pm_{syngs123})$<br>$pm_{gs23}=(pm_{gs2} \times pm_{gs3}/ sum_{gs})\times(pm_{syngs}-pm_{syngs123})$<br>$pm_{gs31}=(pm_{gs3} \times pm_{gs1}/ sum_{gs})\times(pm_{syngs}-pm_{syngs123})$
Synergistic effect of ERK and EGFR on Grb2SoS	pm_{gs23}	
Synergistic effect of EGFR and GAB1 on Grb2SoS	pm_{gs31}	
Synergistic effect of GAB1, ERK, EGFR on Grb2SoS	$pm_{syngs123}$	
Effect of GAB1 on PI3K	pm_{pitk1}	$pm_{synpitk}$*+pm_{pitk1}*+pm_{pitk2}*≤1<br>$0 \le pm_{synpitk}, pm_{pitk1}, pm_{pitk2} \le 1$<br>$add_{synpitk}=1- pm_{synpitk}$<br>$pm_{pitk3}=add_{synpitk}-pm_{pitk1}-pm_{pitk2}$
Effect of Ras on PI3K	pm_{pitk2}	
Effect of EGFR on PI3K	pm_{pitk3}	
Synergistic effect of GAB1 and Ras on PI3K	pm_{pitk12}	$sum_{pitk}=pm_{pitk1} \times pm_{pitk2}+ pm_{pitk2} \times pm_{pitk3} + pm_{pitk3} \times pm_{pitk1}$<br>$pm_{synpitk123}= pm_{synpitk}\times pm_{synpitk}$<br>$pm_{pitk12}=(pm_{pitk1} \times pm_{pitk2}/ sum_{pitk})\times(pm_{synpitk}-pm_{synpitk123})$<br>$pm_{pitk23}=(pm_{pitk2} \times pm_{pitk3}/ sum_{pitk})\times(pm_{synpitk}-pm_{synpitk123})$<br>$pm_{pitk31}=(pm_{pitk3} \times pm_{pitk1}/ sum_{pitk})\times(pm_{synpiyk}-pm_{synpiyk123})$
Synergistic effect of Ras and EGFR on PI3K	pm_{pitk23}	
Synergistic effect of EGFR and GAB1 on PI3K	pm_{pitk31}	
Synergistic effect of GAB1, Ras, EGFR on PI3K	$pm_{synpitk123}$	
Effect of RasGAP on Ras	pm_{ras}	pm_{ras}*+pm_{synras}*≤1<br>$0 \le pm_{ras}, pm_{synras} \le 1$
Effect of Grb2SoS on Ras	$1-pm_{ras}-pm_{synras}$	
Synergistic effect of RasGAP and Grb2SoS on Ras	pm_{synras}	

Effect of Src on GAB1	pm_{gab1}	pm_{syngab}*+ pm_{gab1}*+ pm_{gab2}*≤ 1<br>$0\leq pm_{syngab}, pm_{gab1}, pm_{gab2}\leq 1$<br>$add_{syngab}=1-pm_{syngab}$<br>$pm_{gab3}=add_{syngab}-pm_{gab1}-pm_{gab2}$
Effect of PIP3 on GAB1	pm_{gab2}	
Effect of ERK on GAB1	pm_{gab3}	
Synergistic effect of Src and PIP3	pm_{gab12}	$sum_{gab}=pm_{gab1}\times pm_{gab2}+pm_{gab2}\times pm_{gab3}+pm_{gab3}\times pm_{gab1}$<br>$pm_{syngab123}=pm_{syngab}\times pm_{syngab}$<br>$pm_{gab12}=(pm_{gab1}\times pm_{gab2}/sum_{gab})\times(pm_{syngab}-pm_{syngab123})$<br>$pm_{gab23}=(pm_{gab2}\times pm_{gab3}/sum_{gab})\times(pm_{syngab}-pm_{syngab123})$<br>$pm_{gab31}=(pm_{gab3}\times pm_{gab1}/sum_{gab})\times(pm_{syngab}-pm_{syngab123})$
Synergistic effect of PIP3 and ERK	pm_{gab23}	
Synergistic effect of ERK and Src	pm_{gab31}	
Synergistic effect of Src, PIP3, ERK	$pm_{syngab123}$	
Effect of GAB1 on RasGAP	$pm_{rasgap1}$	$pm_{synrasgap}$*+ $pm_{rasgap1}$*+ $pm_{rasgap2}$*≤ 1<br>$0\leq pm_{synrasgap}, pm_{rasgap1}, pm_{rasgap2}\leq 1$<br>$add_{synrasgap}=1-pm_{synrasgap}$<br>$pm_{rasgap3}=add_{synrasgap}-pm_{rasgap1}-pm_{rasgap2}$
Effect of SHP2 on RasGAP	$pm_{rasgap2}$	
Effect of EGFR on RasGAP	$pm_{rasgap3}$	
Synergistic effect of GAB1 and SHP2	$pm_{rasgap12}$	$sum_{rasgap}=pm_{rasgap1}\times pm_{rasgap1}+pm_{rasgap2}\times pm_{rasgap3}+pm_{rasgap3}\times pm_{rasgap1}$<br>$pm_{synrasgp123}=pm_{synrasgap}\times pm_{synrasgap}$<br>$pm_{rasgap12}=(pm_{rasgap1}\times pm_{rasgap2}/sum_{rasgap})\times(pm_{synrasgap}-pm_{synrasgap123})$<br>$pm_{rasgap23}=(pm_{rasgap2}\times pm_{rasgap3}/sum_{rasgap})\times(pm_{synrasgap}-pm_{synrasgap123})$<br>$pm_{rasgap31}=(pm_{rasgap3}\times pm_{rasgap1}/sum_{rasgap})\times(pm_{synrasgap}-pm_{synrasgap123})$
Synergistic effect of SHP2 and EGFR	$pm_{rasgap23}$	
Synergistic effect of EGFR and GAB1	$pm_{rasgap31}$	
Synergistic effect of GAB1, SHP2, EGFR	$pm_{synrasgap123}$	

*The fourteen parameters (i.e. pm_{syngs}, pm_{gs1}, pm_{gs2}, $pm_{synpitk}$, pm_{pitk1}, pm_{pitk2}, pm_{ras}, pm_{synras}, pm_{syngab}, pm_{gab1}, pm_{gab2}, $pm_{synrasgap}$, $pm_{rasgap1}$, $pm_{rasgap2}$) are sampled from uniform distribution ranging from 0 to 1.

Supplementray Table S14. Results of the distribution perturbation analysis of the EGFR network.

A perturbed parameter distribution	Lin	Hill	Sat	Acc
pm_{syngs}	0.511	0.561	0.343	0.369
pm_{gs1}	0.435	0.547	0.311	0.323
pm_{gs2}	0.458	0.519	0.302	0.305
$pm_{synpitk}$	0.060	0.325	0.169	0.000
pm_{pitk1}	0.416	0.042	0.129	0.321
pm_{pitk2}	0.000	0.000	0.000	0.037
pm_{ras}	0.888	0.655	0.478	0.710
pm_{synras}	0.622	0.655	0.435	0.415
pm_{syngab}	0.619	0.531	0.472	0.576
pm_{gab1}	1.000	1.000	1.000	0.510
pm_{gab2}	0.813	0.473	0.595	1.000
$pm_{synrasgap}$	0.226	0.218	0.181	0.073
$pm_{rasgap1}$	0.129	0.246	0.170	0.052
$pm_{rasgap2}$	0.219	0.305	0.203	0.064

Supplementary Texts

Supplementray Text S1. Analysis of network motifs

I. Construction of the 16 network motifs

We constructed 16 network motifs (i.e. 4 negative feedbacks (motif 1, 2, 3, and 4), 4 positive feedbacks (motif 5, 6, 7, and 8), 4 coherent feedforward loops (motif 9, 10, 11, and 12), 4 incoherent feedforward loops (motif 13, 14, 15, and 16)) (Fig. S3A). Input signal was given to X and the time course of Z was observed. From the simulation data of Z in each network motif, we evaluated the presence of the dynamical features (i.e., adaptation, memory, pulsation, and consistency) (Fig. S3A). Adaptation means how the variables return to original value after the response to the input signal. It is calculated as the product of sensitivity and 1-precision. Memory means if the variable remember the input signal after it vanishes. Pulsation is the ability to generate pulse at the moment of input signal is given or is removed. Consistency measures similarity between the values of Z at each time point between those evaluated at different parameter sets.

II. Normalized equation modeling of the 16 network motifs

Differential equations of each model are made according to the principles described in METHODS (differential equations for all models are provided in Table S8). Each model has two parameters: p1 represents synergistic effect of two links while p2 represents dominant effect of one link over that of the other. p2 means dominant effect from Z to X over Input to X in negative feedback and positive feedback motifs while p2 means dominant effect from Y to Z over X to Z in coherent feedforward and incoherent feedforward motifs. Parameter p1 and p2 were sampled from uniform distribution between 0 and 1 with constraint that the sum of p1 and p2 should be less than or equal to 1. Total 1,000 parameter sets were generated. For each model and for each form of the response function, for each parameter set, numerical simulation was performed using ode15s function in the MATLAB R2009a (i.e. total 64,000 (16×4×1,000) simulations were conducted). Input signal is given from t=10 to t=100. Dynamical features are evaluated for every simulation.

III. Observed dynamical characteristics in the 16 network motifs

Adaptation was mainly observed in negative feedback (motif 1, 2, 3, and 4) and incoherent feedforward motifs (motif 13, 14, 15, and 16). The result was consistent regardless of the form of the response function. Memory was only observed in positive feedback motifs (motif 5, 6, 7, and 8) except for motif 5 with Hill, motif 7 with Hill, and motif 8 with Hill. Consistency was observed in all coherent feedforward motifs (motif 9, 10, 11, and 12) regardless of the form of the response function. Pulsation was observed in incoherent feedforward motifs (mainly with the function 'Hill'). From the results, we can conclude that the specific dynamical features are associated with the specific network motifs. These features may be determined by network structure of the motifs rather than the detailed arrangement of the links inside the motifs or form of the response functions. Therefore, dynamics of the network was successfully represented by normalized equation modeling. In Table S9, we summarized the association of network motifs and dynamical features. These results were consistent with previous studies (Table S10) (1, 2).

IV. Calculation of marginal distributions of the parameters

Then, which link is associated with the dynamical features? For example, which link is related with the property of adaption in type 1 negative feedback motif? To investigate the question, we observed the shape of the marginal distributions for the parameters in four such associations: association 1 - adaptation with negative feedback motifs, association 2 - adaptation with incoherent feedforward motifs, association 3 - memory with positive feedback motifs, association 4 - pulsation with incoherent feedforward motifs. Marginal distribution was calculated for the parameters that show upper 10% of the dynamics of interest for each association (Fig. S3A).

IV-1. Association 1: adaptation in negative feedback motifs

High p1 and low p2 were associated with adaptation in all types of negative feedback motifs. That is, the higher the synergistic effect of Input and Z, the lower the lower the dominant effect of Z to X in inhibition of X, adaptation can be observed more frequently. Therefore, coordination of two links in regulation of X may be important to generate the

dynamical feature, "adaptation". The distributions were similar regardless of the form of the response functions.

IV-2. Association 2: adaptation in incoherent feedforward motifs

For adaptation, different distributions of parameters were observed depending on the type of incoherent feedforward motifs (in motif 13 and 16, high p1 and low p2 was associated with adaptation while low p1 and high p2 was associated with adaptation in motif 14 and motif 15). However, the marginal distributions were not different according to the form of response functions.

IV-3. Association 3: memory in positive feedback motifs

Calculation of marginal distributions for type 1 positive feedback with Hill, type 3 positive feedback with Hill, and type 4 positive feedback with Hill was impossible because of zero or small number of cases. Except for these, low p1 and high p2 are associated with memory. That is, the lower the synergistic effect of Input and Z, the higher the dominant effect of Z to X in activation of X, adaptation can be observed more frequently. Therefore, positive feedback link may take a role to generate the dynamical feature, "memory".

IV-4. Association 4: pulsation in incoherent feedforward motifs

Low p1 and medium p2 were associated with pulsation in all types of incoherent feedforward motifs. That is, pulsation was observed more frequently when the synergistic effect of Input and Z was low and the effect of Y on Z (indirect effect for the activation of Z) was neither high nor low. The latter is an interesting point. Therefore, coordination of direct effect (i.e. X on Z) and indirect effect (i.e. Y on Z) may be required to generate the dynamical feature, "pulsation". The distributions were similar regardless of the form of the response functions.

V. Distribution perturbation analysis

Although distinct distributions of parameters were observed for specific dynamics in all previously investigated network-dynamics associations, it does not always imply that these distributions generate the dynamics. To further investigate this issue, we performed "distribution perturbation analysis". After the one or two parameters were sampled from marginal distribution while the others were sampled from uniform distribution, numerical simulation was performed and change of phenotypes (or dynamics) was evaluated. If the results showed certain dynamics more frequently, it will suggest that the distribution generates the dynamics. For one distribution perturbation analysis, we sampled the value of one parameter (p1 or p2) from marginal distribution whereas the other (p2 or p1) was sampled from uniform distribution. For two distribution perturbation analysis, we sampled both parameters (p1 and p2) from marginal distribution. One constraint required to be satisfied during the process of random sampling is that the sum of p1 and p2 is less 1. The results of distribution perturbation analyses were demonstrated Fig. S4 and Table S11.

V-1. Association 1: adaptation in negative feedback motifs

For all types of negative feedback motifs and all forms of response functions, perturbation of any one distribution and two distributions generated more degree of adaptation. The perturbation effect of the two distributions was always larger than that of one marginal distribution. This results implies that high p1 and low p2 (i.e., strong synergistic effect with dominant role of input) actively generates the dynamical feature, adaptation.

V-2. Association 2: adaptation in incoherent feedforward motifs

Distribution perturbation has more impacts in type 1 and type 4 than in type 2 and type 3. That is, marginal distributions of p1 and p2 in motif 14 and motif 15 do not have significant causal relationship with adaptation.

V-3. Association 3: memory in positive feedback motifs

For all types of positive feedback motifs and all forms of response functions, distribution

perturbation of p2 generates memory more effectively. In addition, differences are very slight between the results of distribution perturbtion of p2 and those of distribution perturbation of both p1 and p2. These results imply that high p2 have more significant causal relationship with memory than low p1.

V-4. Association 4: pulsation in incoherent feedforward motifs

For all types and all forms of response functions, perturbation of any one distribution and two distribution generated more degree of adaptation. The perturbation effect of two distributions was always larger than that of one distribution. This results implies that low p1 and medium p2 (i.e., strong synergistic effect with dominant role of input) actively generates the dynamical feature, pulsation.

VI. Summary

Normalized equation modeling revealed well-known dynamical features of network motifs: negative feedback and incoherent feedforward motifs showed adaptation; positive feedback motifs showed memory; coherent feedforward motif showed consistency. Furthermore, the conditions for the dynamic features can be identified through distribution perturbation analysis. Most of the results were consistent regardless of the form of the response function which strengthen the reliability of the developed method.

Supplementray Text S2. Analysis of the EGFR network

I. Construction of the EGFR network

Network structure is the simplified form of the previously constructed EGFR network (Fig. S5A).

II. Normalized equation modeling of the EGFR network.

Differential equations of each model are made according to the principles described in METHODS (differential equations for all models are provided in Table S12). The model requires 14 parameters (i.e. pm_{syngs}, pm_{gs1}, pm_{gs2}, $pm_{synpitk}$, pm_{pitk1}, pm_{pitk2}, pm_{ras}, pm_{synras}, pm_{syngab}, pm_{gab1}, pm_{gab2}, $pm_{synrasgap}$, $pm_{rasgap1}$, $pm_{rasgap2}$) which are sampled from standard uniform distribution. The remaining parameter can be calculated from the sampled parameters. In this manner, 100,000 parameter sets were randomly generated. The detailed information of the parameters is provided in Table S13.

III. Measurement of the MEK inhibitor resistance

Numerical simulation was performed using ode15s function in the MATLAB R2009a. MEK inhibitor is treated at t=100 (Fig. S5B). The resistance of the MEK inhibitor was calculated as the increased amount of AKT after MEK inhibitor is given.

IV. Marginal distribution of parameters

To find out which parameter is associated with MEK inhibitor resistance, we investigated the marginal distributions of all 14 parameters. Marginal distribution was calculated for the parameter sets that showed top 10% of the MEK inhibitor resistance. Among 14 parameters, six parameters (pm_{gs2}, pm_{pitk1}, pm_{synras}, pm_{ras}, $pm_{synrasgap}$, pm_{rasgap}) represented non-uniform, coherent marginal distributions (Fig. S5C).

V. One-distribution perturbation analyses

The presence of the association between MEK inhibitor resistance and marginal

distribution of parameters do not always imply the inducing relationship between them. To evaluate the inducing relationship, we performed one-distribution perturbation analyses. As a result, the marginal distributions of eight parameters (i.e., pm_{syngs}, pm_{gs1}, pm_{gs2}, pm_{synras}, pm_{ras}, pm_{syngab}, pm_{gab1}, pm_{gab2}) were shown to have the inducing relationship with the MEK inhibitor resistance (Table S14). These parameters represent the regulation of Grb2Sos (i.e., pm_{syngs}, pm_{gs1}, pm_{gs2}), Ras (i.e., pm_{synras}, pm_{ras}), and GAB1(i.e., pm_{syngab}, pm_{gab1}, pm_{gab2}). Among them, the importance of GAB1 regulation was supported by published experimental results (3).

VI. Summary

We investigated the underlying mechanism of MEK inhibitor resistance in the EGFR network using normalized equation modeling and one-distribution perturbation analysis. The regulation of Grb2Sos, Ras, and GAB1 were identified as essential regulatory processes to generate MEK inhibitor resistance, among which GAB1 regulation had been also identified as a key process in previously published results.

Supplementray Text S3. Normalized equation modeling when one node is regulated by more than two nodes

Let us suppose that Y is regulated by A, B, and C (Fig. S6). Then the instantaneous rate of change in Y (dY/dt) is determined in consideration of these 7 influences: individual influence of A on Y (shortly, A on Y), individual influence of B on Y (B on Y), individual influence of C and Y (C on Y), combined influence of A and B on Y (AB on Y), combined influence of B and C on Y (BC on Y), combined influence of C and A on Y (CA on Y), combined influence of A, B, and C on Y (ABC on Y).

The seven influences can be divided by individual influences (i.e., A on Y, B on Y, C on Y) and combined influences (i.e., AB on Y, BC on Y, CA on Y, ABC on Y). The sum of combined influences (i.e., AB on Y + BC on Y + CA on Y + ABC on Y) is parameterized as p1. Then the sum of individual influences becomes 1-p1. p2 represents A on Y, and p3 represents B on Y. Then, C on Y is determined as 1-p1-p2-p3.

The four combined influences are determined using p1, p2, and p3. Since ABC on Y has double combinations, ABC on Y is determined as $p1^2$. Then, the sum of remaining three combined influences becomes $p1-p1^2$. AB on Y, BC on Y, and CA on Y are determined using A on Y, B on Y, and C on Y. Specifically, the ratio between AB on Y, BC on Y, CA on Y is determined to be equal to the ration between A on Y × B on Y, B on Y × C on Y, C on Y × A on Y. Since the sum is already known as $p1-p1^2$, these three influences can be determined using a proportional expression. For example, if p1, p2, and p3 are 0.2, 0.4, and 0.25 respectively, seven influences are determined as follows: A on Y is 0.4; B on Y is 0.25; C on Y is 0.15 (i.e., 1-0.2-0.4-0.25); ABC on Y is 0.04 (i.e., 0.2 × 0.2); AB on Y is 0.081 (i.e., $\frac{0.1\times 0.16}{0.1+0.0375+0.06}$); BC on Y is 0.0304 (i.e., $\frac{0.0375\times 0.16}{0.1+0.0375+0.06}$); CA on Y is 0.0486 (i.e., $\frac{0.06\times 0.16}{0.1+0.0375+0.06}$).

When one node is regulated by more than three nodes, the formulation of dY/dt can be constituted in the same manner.

Supplementary Data Sets

Supplementray Data Set S1. Experimental database for the cardiac signaling pathways.

Reference (PMID)	Experimental Setting	Input	Output	Effect	Time
15781459	The FOXO3a transcription factor regulates cardiac myocyte size downstream of AKT signaling				
		none	pFOXO1/3a/4/pAkt	1	10min
	NRVM	IGF-1	pFOXO1	2	10min
	NRVM	IGF-1	pFOXO4	3	10min
	NRVM	IGF-1	pFOXO3a	1.5	10min
	NRVM	IGF-1	pAkt	2	10min
	NRVM	Insulin+LY294002	pFOXO1/3a/4	1	10min
	NRVM	insulin+LY294002	pAkt	1.5	10min
	NRVM	none	pAkt	1	12hr
	NRVM+TM-FOXO3a	none	pAkt	2	12hr
	NRVM+WT-FOXO3a	none	pAkt	2	12hr
	TAC on mouse	none	pAkt/pFOXO3a	4/2	TAC 1wk
	NRVM	IGF-1	cell size	1.4	48hr
	NRVM+WT-FOXO3a	IGF-1	cell size	1.3	24hr(IGF)+24 after TD
	NRVM+TM-FOXO3a	IGF-1	cell size	0.85	24hr(IGF)+24 after TD
	NRVM	stretch	cell size	1.3	48hr
	NRVM+WT-FOXO3a	stretch	cell size	1.15	24hr(stretch)+24 after TD
	NRVM+TM-FOXO3a	stretch	cell size	0.9	24hr(stretch)+24 after TD
9363896	Switching of the coupling of the beta2-adrenergic receptor to different G proteins by protein kinase A				
	HEK293	none	pMAPK	1	5min
	HEK293	Isoprenaline(10^-10/-9/-8/-7/-6/-5)	pMAPK	1.2/2/4/5.5/6/5.8	5min
	HEK293	Isoprenaline+Propranolol	pMAPK	1	5min
	HEK293	Isoprenaline	pMAPK	6.5	5min

	HEK293+bARKct TF	Isoprenaline	pMAPK	3	5min
	HEK293	Isoprenaline+PTX	pMAPK	1.7	5min
	HEK293+Csk TF	Isoprenaline	pMAPK	3	5min
	HEK293+Sos-Pro TF	Isoprenaline	pMAPK	2.6	5min
	HEK293	Isoprenaline	pMAPK	7	5min
	HEK293	Isoprenaline+H-89	pMAPK	1.5	5min
	HEK293	LPA	pMAPK	8	5min
	HEK293	LPA+H-89	pMAPK	8	5min
	HEK293	isoprenaline	pMAPK	5	5min
	HEK293+ERK1 TF+b2ARWT TF	isoprenaline	pMAPK	7	5min
	HEK293+ERK1 TF+b2ARmut TF	isoprenaline	pMAPK	3	5min
15964981	alpha1-Adrenoceptor stimulation potentiates L-type Ca2+ current through Ca2+/calmodulin-dependent PK II(CaMKII) activation in rat ventricular myocytes				
	Adult cardiomyocyte(Rat)	phenylephrine(10uM)	normalized LTCC current	1.0/0.95/0.9/0.92/0.98/1/1.15/1.18	0/1/2/3/4/5/10/15min
	Adult cardiomyocyte(Rat)+BAPTA pretx	phenylephrine(10uM)	normalized LTCC current	1/1/1/1/1/1/1/1	0/1/2/3/4/5/10/15min
	Adult cardiomyocyte(Rat)+KN-93(0.5uM) pretx	phenylephrine(10uM)	normalized LTCC current	1.0/0.9/0.8/0.8/0.79/0.76/0.7/0.65	0/1/2/3/4/5/10/15min
	Adult cardiomyocyte(Rat)+KN-92(0.5uM) pretx	phenylephrine(10uM)	normalized LTCC current	1.0/0.98/0.95/1.0/1.03/1.05/1.15/1.17	0/1/2/3/4/5/10/15min
	Adult cardiomyocyte(Rat)+chelerythrine(10uM) pretx	phenylephrine(10uM)	normalized LTCC current	1.0/0.9/0.8/0.77/0.75/0.7/0.55/0.	0/1/2/3/4/5/10/15min

				45	
	Adult cardiomyocyte(Rat)	control/phe(0.1uM)/1/10/100	CaMKII activation	100/130/170/190/185	15min
	Adult cardiomyocyte(Rat)	control/prazocin/pra+phe	CaMKII activation	100/100/105	15min
	Adult cardiomyocyte(Rat)	control/KN-93/KN-93+phe	CaMKII activation	100/70/75	15min
	Adult cardiomyocyte(Rat)	control/che/che+phe	CaMKII activation	100/85/99	15min
15572667	p38alpha Mitogen-Activated Protein Kinase Plays a Critical Role in Cardiomyocytes Survival but Not in Cardiac Hypertrophic Growth in Response to Pressure Overload				
	control mouse	none	p38 kinase activity	1	
	control	phenylephrine	p38 kinase activity	1.6	30min
	p38aCKO(10wk)	none	p38 kinase activity	0.25	
	p38aCKO(10wk)	phenylephrine	p38 kinase activity	0.4	30min
	control	none	FS	37	
	p38aCKO(10wk)	none	FS	37	
	p38aCKO(10wk)	TAC(1wk)	FS	15	
	p38aCKO(10wk)	sham(1wk)	FS	37	
	control	TAC(1wk)	FS	37	
	control	sham(1wk)	FS	37	
	control	sham(1wk)	TUNEL(+) myocytes	37	
	control	TAC(1wk)	TUNEL(+) myocytes	37	
	p38aCKO(10wk)	sham(1wk)	TUNEL(+) myocytes	37	
	p38aCKO(10wk)	TAC(1wk)	TUNEL(+)	115	

			myocytes		
	control	sham(1wk)	cytochrome c in cytosol	1	
	control	TAC(1wk)	cytochrome c in cytosol	1.5	
	p38aCKO(10wk)	sham(1wk)	cytochrome c in cytosol	1.3	
	p38aCKO(10wk)	TAC(1wk)	cytochrome c in cytosol	3.7	
	control	TAC(1wk)	Bax/Bcl	1	
	p38aCKO(10wk)	TAC(1wk)	Bax/Bcl	1.7	
	control	sham(1wk)	pJNK/totalJNK	1	
	control	TAC(1wk)	pJNK/totalJNK	2.2	
	p38aCKO(10wk)	sham(1wk)	pJNK/totalJNK	0.7	
	p38aCKO(10wk)	TAC(1wk)	pJNK/totalJNK	3.5	
	control	sham(1wk)	pERK/totalERK	1	
	control	TAC(1wk)	pERK/totalERK	2	
	p38aCKO(10wk)	sham(1wk)	pERK/totalERK	1	
	p38aCKO(10wk)	TAC(1wk)	pERK/totalERK	2	
	control	sham(1wk)	pMKK3/6 / totalMKK3/6	1	
	control	TAC(1wk)	pMKK3/6 / totalMKK3/6	5	
	p38aCKO(10wk)	sham(1wk)	pMKK3/6 / totalMKK3/6	5	
	p38aCKO(10wk)	TAC(1wk)	pMKK3/6 / totalMKK3/6	40	
	control	saline	LVDd	3.5	
	control	isoproterenol(7.5mg/kg /day for 2days)	LVDd	3.5	
	p38aCKO(10wk)	saline	LVDd	3.5	
	p38aCKO(10wk)	isoproterenol(7.5mg/kg /day for 2days)	LVDd	4.5	

	control	saline	FS	40	
	control	isoproterenol(7.5mg/kg /day for 2days)	FS	48	
	p38aCKO(10wk)	saline	FS	38	
	p38aCKO(10wk)	isoproterenol(7.5mg/kg /day for 2days)	FS	15	
	control	saline	TUNEL(+) myocytes	23	
	control	isoproterenol(7.5mg/kg /day for 2days)	TUNEL(+) myocytes	26	
	p38aCKO(10wk)	saline	TUNEL(+) myocytes	25	
	p38aCKO(10wk)	isoproterenol(7.5mg/kg /day for 2days)	TUNEL(+) myocytes	50	
	control cell	isoproterenol(0uM)	survival	100	
	p38aCKO(10wk) cell	isoproterenol(0uM)	survival	100	
	control cell	isoproterenol(0.31uM)	survival	120	
	p38aCKO(10wk) cell	isoproterenol(0.31uM)	survival	110	
	control cell	isoproterenol(0.63uM)	survival	110	
	p38aCKO(10wk) cell	isoproterenol(0.63uM)	survival	105	
	control cell	isoproterenol(1.25uM)	survival	120	
	p38aCKO(10wk) cell	isoproterenol(1.25uM)	survival	102	
	control cell	isoproterenol(2.5uM)	survival	120	
	p38aCKO(10wk) cell	isoproterenol(2.5uM)	survival	50	
	control cell	isoproterenol(5uM)	survival	120	
	p38aCKO(10wk) cell	isoproterenol(5uM)	survival	30	
	control cell	isoproterenol(10uM)	survival	50	
	p38aCKO(10wk) cell	isoproterenol(10uM)	survival	25	
	control cell	isoproterenol(20uM)	survival	28	
	p38aCKO(10wk) cell	isoproterenol(20uM)	survival	28	
	control cell	isoproterenol(40uM)	survival	28	
	p38aCKO(10wk) cell	isoproterenol(40uM)	survival	28	

22042619	Selective repression of MEF2 activity by PKA-dependent proteolysis of HDAC4				
	H9C2 myocyte	none	MEF2	1	
	H9C2 myocyte	dbcAMP(1mM)	MEF2	0.1	
	H9C2 myocyte	dbcAMP(1mM) + KT5720(2uM)	MEF2	0.8	
	COS cells	Myc-PKA:none	MEF2	0.05	
	COS cells+MEF2C	Myc-PKA:none	MEF2	1	
	COS cells+MEF2C+HDAC4	Myc-PKA:none	MEF2	0.1	
	COS cells+MEF2C+HDAC4+CaMKII	Myc-PKA:none	MEF2	1.2	
	COS cells	Myc-PKA:low	MEF2	0.05	
	COS cells+MEF2C	Myc-PKA:low	MEF2	1	
	COS cells+MEF2C+HDAC4	Myc-PKA:low	MEF2	0.15	
	COS cells+MEF2C+HDAC4+CaMKII	Myc-PKA:low	MEF2	0.5	
	COS cells	Myc-PKA:high	MEF2	0.05	
	COS cells+MEF2C	Myc-PKA:high	MEF2	1	
	COS cells+MEF2C+HDAC4	Myc-PKA:high	MEF2	0.13	
	COS cells+MEF2C+HDAC4+CaMKII	Myc-PKA:high	MEF2	0.3	
11435346	Isoproterenol activates extracellular signal-regulated protein kinases in cardiomyocytes through calcineurin				
	NRVM	none	ERK1/2	0.1	
	NRVM+thapsigargin/nifedipine/EGTA pretx	none	ERK1/2	0.1/0.1/0.1	
	NRVM	Iso	ERK1/2	1	
	NRVM+thapsigargin/nifedipine/EGTA pretx	Iso	ERK1/2	0.5/0.2/0.2	
	NRVM	none	ERK1/2	0.1	8min
	NRVM	Iso	ERK1/2	1	8min

	NRVM+KN93/W7/CsA pretx	Iso	ERK1/2	1.3/0.4/0.4	8min
	NRVM	none	ERK1/2	0.1	8min
	NRVM	AngII	ERK1/2	0.4	8min
	NRVM	PHE	ERK1/2	2	8min
	NRVM+CsA pretx	none	ERK1/2	0.1	8min
	NRVM+CsA pretx	AngII	ERK1/2	0.4	8min
	NRVM+CsA pretx	PHE	ERK1/2	1.5	8min
	NRVM	Iso(10uM)	calcineurin	100/120/180/260/150/130	0/1/2/5/15/30min
	NRVM	Iso(0/0.01/0.1/1/10/100uM)	calcineurin	100/180/190/200/310/300	5min
	NRVM	Iso	Raf-1	0.1/0.5/1/1/1/0.5	0/1/2/5/10/15min
	NRVM+CsA pretx	Iso	Raf-1	0.1/0.1/0.1/0.1/0.1/0.1	0/1/2/5/10/15min
18323524	Epac mediates beta-adrenergic receptor-induced cardiomyocyte hypertrophy				
	Rat myocardium	Sham/TAC	Epac1 expression	1/1.6	5day
	ARVM	Sham/Sham+8-CPT/TAC/TAC+8-CPT	Cell area	100/110/110/140	24hr(8-CPT)
	ARVM	Ad.GFP/Ad.GFP+8-CPT/Ad.Epac/Ad.Epac+8-CPT	Cell area	100/115/105/130	36hr(Ad.Epac or 8-CPT)
	ARVM	Ad.GFP/Ad.GFP+8-CPT/Ad.Epac/Ad.Epac+8-CPT	Ras-GTP/total Ras	0.1/0.7/0.3/1	36hr(Ad.Epac)+5min(8-CPT)
	ARVM	Ad.GFP/Ad.GFP+Ad.RasS17N/Ad.Epac/Ad.Epac+8-CPT	[3H]-Leucine uptake	100/105/120/85	1day(8-CPT)
	ARVM	Ad.GFP/Ad.GFP+Ad.RasS17N/Ad.Epac/Ad.E	Calcineurin activity	100/150/220/130	30min(8-CPT)

		pac+8-CPT			
	ARVM	Ad.GFP/Ad.Epac1/Ad.Epac1+8-CPT/Ad.Epac1+8-CPT+KN-93	P-CaMKII/total CaMKII	1/1.2/3/1.2	30min(8-CPT)
	ARVM	none/CsA/KN93/8-CPT/8-CPT+CsA/8-CPT+KN-93	[3H]-Leucine uptake	100/95/96/150/115/98	24hr
	NRVM	shCT/shCT+ISO/shEpac1/shEpac1+ISO	Cell area	100/150/104/130	48hr(ISO)
	NRVM	shCT/shCT+ISO/shEpac1/shEpac1+ISO	ANF	25/225/60/140	48hr(ISO)
	ARVM	none/Ad.PKI/ISO/Ad.PKI+ISO	[3H]-Leucine uptake	110/95/150/140	24hr(ISO)
	ARVM	none/Ad.PKI/ISO/Ad.PKI+ISO	PKA activity	80/70/180/90	10min(ISO)
	ARVM	Ad.Epac1/Ad.Epac1+ISO/Ad.Epac1R279K/Ad.Epac1R279K+ISO	[3H]-Leucine uptake	100/135/115/115	24hr
	Human heart(NonFailing(NF)/Heart Failure(HF))	none	Epac1 expression	1.0/2.0	
22056318	Epac enhances excitation-transcription coupling in cardiac myocytes				
	ARVM	8-CPT(10uM)	nuclear HDAC5	1.0/1.0/0.87/0.72/0.66/0.63	0/10/20/30/40/50/60min
	ARVM	endothelin(100nM)	nuclear HDAC5	1.0/0.95/0.87/0.82/0.78/0.67/0.55	0/10/20/30/40/50/60min
	NRVM	control/epac1WT TF	MEF-2	100/300	
	NRVM	8-CPT/KN93+8-CPT	nuclear HDAC5	0.7/1.0	
10931827	beta-Adrenergic Pathway Induces Apoptosis through Calcineurin Activation in Cardiac Myocytes				
	NRVM	control/Iso(10^-	% of TUNEL	4/5/10/15/	48hr

		7)/Iso(10^-6)/Iso(10^-5)/Iso(5*10^-5)/Ionomycin(10^-6)	positive cells	21/18	
	NRVM	control/Iso(50uM)/CsA pretx(1hr)+Iso/FK506(1hr)+Iso/nifedipine(1hr)+Iso	% of TUNEL positive cells	5/18/5/5/9	48hr
	NRVM	Iso(50uM)	pBad	1/0.5/0.5/1	0/2/4/6hr
	NRVM	Iso(50uM)+FK506	pBad	1/1/1/1	0/2/4/6hr
	NRVM	Iso(50uM)+nifedipine	pBad	1/1/1/1	0/2/4/6hr
	NRVM	Iso(50uM)	Bcl-2	1/1/1/1/1	0/6/12/24/48hr
	NRVM	Iso(50uM)	cytosolic cyt c	1/2/3/3	0/8/18/24hr
17307839	Cardiomyocyte-specific inactivation of transcription factor CREB in mice				
	control	none	survival	1.0/1.0/1.0/0.95/0.95	0/100/200/300/400
	Creb1 deficient mice	none	survival	1.0/0.98/0.95/0.9/0.9	0/100/200/300/400
	control/creb1 deficient mice	none	bcl-2 expression	100/100	
9346240	Akt phosphorylates BAD in vitro				
	3T3 cells	Vector/Active AKT TF/WT AKT TF/Inactive AKT TF	pBAD	0/1/0/0	
	3T3 cells	none/PDGF/Wort+PDGF/LY294002+PDGF	pBAD	0.1/1/0.5/0.5	
165545	Induction of bcl-2 expression by phosphorylated CREB proteins during B-cell activation and rescue from apoptosis				
	DHL-9 cells	Basal Promoter/WtUREDRE/MutCRE	bcl-2	1.0/3.0/1.3	
17296607	Activation of CaMKIIdeltaC is a common intermediate of diverse death stimuli-induced heart muscle cell apoptosis				
	ARCM	none	TUNEL positive cells(%)	2/4/5/8/9	0/1/2/3/4day
	ARCM	CA-CaMKIIdc	TUNEL positive cells(%)	3/9/18/20/22	0/1/2/3/4day

	ARCM	CA-CaMKIIdc+DN-CaMKIIdc	TUNEL positive cells(%)	3/4/12/13/14	0/1/2/3/4day
	ARCM	CA-CaMKIIdc+AIP	TUNEL positive cells(%)	3/7/8/12/13	0/1/2/3/4day
	ARCM	CA-CaMKIIdc+Bcl-XL	TUNEL positive cells(%)	3/5/10/12/14	0/1/2/3/4day
	ARCM	CaMKII activity(1/2/3/5/7)	TUNEL positive cells(%)	4/6/14/16/17	24hr
19667065	Identification of Novel in Vivo Phosphorylation Sites of the Human Proapoptotic Protein BAD: PORE-FORMING ACTIVITY OF BAD IS REGULATED BY PHOSPHORYLATION				
	HEK293+BAD TF+B-RAF TF	DMSO/BAY43-9006 0.1uM/1uM/10uM	pBAD(S75)	0.35/0.45/0.25	16hr TF + 22hr BAY
	HEK293+BAD TF+B-RAF TF	DMSO/BAY43-9006 0.1uM/1uM/10uM	pBAD(S99)	0.4/0.4/0.05/0	16hr TF + 22hr BAY
	HEK293+BAD TF+B-RAF TF	DMSO/BAY43-9006 0.1uM/1uM/10uM	pBAD(S118)	0.15/0.3/0.01/0.01	16hr TF + 22hr BAY
	HEK293+BAD	none	Apoptotic cells(%)	25	16hr TF + 30hr culture
	HEK293+BAD+B-RAF-KD	none	Apoptotic cells(%)	24	16hr TF + 30hr culture
	HEK293+BAD+B-RAF	none	Apoptotic cells(%)	10	16hr TF + 30hr culture
	HEK293+B-RAF	none	Apoptotic cells(%)	8	16hr TF + 30hr culture
24248367	Selective regulation of cyclic nucleotide phosphodiesterase PDE3A isoforms				
	HEK293+PDE3A2	Ctrl/ISO/PMA	PDE3A2-pS428	0.1/0.1/1.0	ISO(1uM) 90s, PMA(10ng/ml) for 15min
	HEK293+PDE3A1	Ctrl/ISO/PMA	PDE3A1-pS312	0.1/1.0/0.1	ISO(1uM) 90s, PMA(10ng/ml) for 15min
	HEK293+PDE3A2	Ctrl/ISO/IBMX/IBMX+ISO	PDE3A1-pS312	0.2/0.2/0.2/1.0	ISO(1uM) 90s, PMA(10ng/ml) for 15min
	HEK293+PDE3A2	Ctrl/ISO/IBMX/IBMX+ISO	PDE3A1-pS312	0.1/0.2/0.2/1.0	ISO(1uM) 90s, PMA(10ng/ml) for 15min
	HEK293+PDE3A2	PKA 0nM/10nM/100nM	PDE3A2-pS312	0.1/0.5/1.0	20min
	HEK293+PDE3A1	PKA	PDE3A1-pS312	0.1/0.2/1.0	20min

		0nM/10nM/100nM			
	HEK293+PDE3A2	Ctrl/PMA/dBcAMP	PDE3 activity	100/120/125	PMA 15min, dBcAMP 1h
	HEK293+PDE3A1	Ctrl/ISO/PMA/dBcAMP	PDE3 activity	100/100/100/145	ISO 90s, PMA 15min, dBcAMP 1h
23933582	Differential regulation of cardiac excitation-contraction coupling by cAMP phoshodiesterase subtypes				
	ARVM	Ctrl/Bay/Iso/Iso+Bay	Fura2 ratio(% of diastolic ratio)	20/40/100/130	
	ARVM	Ctrl/Bay/Iso/Iso+Bay	t1/2off relaxation(s) of calcium transient	0.45/0.4/0.25/0.25	
	ARVM	Ctrl/Bay/Iso/Iso+Bay	Sarcomere shortening	0.003333333	
	ARVM	Ctrl/Bay/Iso/Iso+Bay	t1/2off relaxation(s) of sarcomere shortening	0.5/0.35/0.15/0.15	
	ARVM	Ctrl/Cil/Iso/Iso+Cil	Fura2 ratio(% of diastolic ratio)	20/45/100/140	
	ARVM	Ctrl/Cil/Iso/Iso+Cil	t1/2off relaxation(s) of calcium transient	0.4/0.3/0.25/0.2	
	ARVM	Ctrl/Cil/Iso/Iso+Cil	Sarcomere shortening	0.001960784	
	ARVM	Ctrl/Cil/Iso/Iso+Cil	t1/2off relaxation(s) of sarcomere shortening	0.5/0.25/0.15/0.1	
	ARVM	Ctrl/Ro/Iso/Iso+Ro	Fura2 ratio(% of diastolic ratio)	20/20/80/140	
	ARVM	Ctrl/Ro/Iso/Iso+Ro	t1/2off relaxation(s) of calcium transient	0.4/0.35/0.25/0.25	
	ARVM	Ctrl/Ro/Iso/Iso+Ro	Sarcomere	0.0046296	

			shortening	3	
	ARVM	Ctrl/Ro/Iso/Iso+Ro	t1/2off relaxation(s) of sarcomere shortening	0.5/0.45/0.15/0.1	
15367659	Protein Kinases C and D Mediate Agonist-Dependent Cardiac Hypertrophy through Nuclear Export of Histone Deacetylase 5				
	NRVM+Ad GFP-HDAC5	none	HDAC5 nuclear localization	750	2hr
	NRVM+Ad GFP-HDAC5	PE	HDAC5 nuclear localization	150	2hr
	NRVM+Ad GFP-HDAC5	PE+KN93	HDAC5 nuclear localization	100	2hr
	NRVM+Ad GFP-HDAC5	PE+U1026	HDAC5 nuclear localization	150	2hr
	NRVM+Ad GFP-HDAC5	PE+HA1077	HDAC5 nuclear localization	300	2hr
	NRVM+Ad GFP-HDAC5	PE+Y27632	HDAC5 nuclear localization	200	2hr
	NRVM+Ad GFP-HDAC5	PE+DAGK Inhibitor	HDAC5 nuclear localization	250	2hr
	NRVM+Ad GFP-HDAC5	PE+Wortmannin	HDAC5 nuclear localization	20	2hr
	NRVM+Ad GFP-HDAC5	PE+Rapamycin	HDAC5 nuclear localization	200	2hr
	NRVM+Ad GFP-HDAC5	PE+SB216763	HDAC5 nuclear localization	10	2hr
	NRVM+Ad GFP-HDAC5	PE+Bis1	HDAC5 nuclear localization	600	2hr
	NRVM+Ad GFP-HDAC5	PE+Staurosporine	HDAC5 nuclear localization	1000	2hr
	NRVM+Ad GFP-HDAC5	none	HDAC5 nuclear localization	330	2hr
	NRVM+Ad GFP-HDAC5	PE	HDAC5 nuclear localization	100	2hr

	NRVM+Ad GFP-HDAC5	Bis1 pretx 30min+PE	HDAC5 nuclear localization	430	2hr
	NRVM+Ad GFP-HDAC5	ET-1	HDAC5 nuclear localization	110	2hr
	NRVM+Ad GFP-HDAC5	Bis1 pretx 30min+ET-1	HDAC5 nuclear localization	115	2hr
	NRVM+Ad GFP-HDAC5	FBS	HDAC5 nuclear localization	130	2hr
	NRVM+Ad GFP-HDAC5	Bis1 pretx 30min+FBS	HDAC5 nuclear localization	80	2hr
11715023	Akt/mTOR pathway is a crucial regulator of skeletal muscle hypertrophy and can prevent muscle atrophy in vivo				
	Rat heart	clenbuterol	Heart weight	21	14days
	Rat heart	clenbuterol+CsA	Heart weight	1	14days
	plantaris muscle lysates from control rats	none	calcineurin activity	47	14days
	plantaris muscle lysates from control rats	CsA	calcineurin activity	33	14days
	CH	none	calcineurin activity	36	14days
	CH	CsA	calcineurin activity	33	14days
	CH	none	Muscle weight	37	14days
	CH	CsA	Muscle weight	36	14days
	CH	none	Muscle weight	58	30days
	CH	CsA	Muscle weight	89	30days
	Rat plantaris muscle of control	none	Fibre cross-sectional area	2100	14days
	CH	none	Fibre cross-sectional area	3300	14days
	CH	CsA	Fibre cross-sectional area	3200	14days
	Rat plantaris muscle of control	none	Fibre cross-sectional area	1400	14days

	CH	none	Fibre cross-sectional area	2800	14days
	CH	CsA	Fibre cross-sectional area	2600	14days
	muscle fibres expressing slow MyHC in plantaris muscle of control	none	% of total fibre	12	14days
	CH	none	% of total fibre	25	14days
	CH	CsA	% of total fibre	24	14days
	CH	FK506	Muscle weight	70	7days
	plantaris of control	none	GSK-3β activity	100	14days
	CH	none	GSK-3β activity	75	14days
	CH	Rap	GSK-3β activity	75	14days
	plantaris of control	none	p70S6K activity	100	14days
	CH	none	p70S6K activity	140	14days
	CH	Rap	p70S6K activity	110	14days
	CH	none	Muscle weight	23	7days
	CH	Rap	Muscle weight	7	7days
	CH	none	Muscle weight	40	14days
	CH	Rap	Muscle weight	5	14days
	muscle fibres in rat plantaris muscle of control	none	Fibre cross-sectional area	2100	14days
	CH	none	Fibre cross-sectional area	3300	14days
	CH	Rap	Fibre cross-sectional area	2400	14days
	muscle fibres in rat plantaris muscle of control	none	Fibre cross-sectional area	1300	14days
	CH	none	Fibre cross-sectional area	2800	14days
	CH	Rap	Fibre cross-sectional area	1800	14days

8631897	Serum Response Factor Mediates AP-1-dependent Induction of the Skeletal α-Actin Promoter in Ventricular Myocytes				
	cardiac myocyte+Δ56Fos	none	Skeletal α-Actin activity	1	24hr
	cardiac myocyte+Δ56Fos+Fos 1ug	none	Skeletal α-Actin activity	1.3	24hr
	cardiac myocyte+Δ56Fos+Fos 5ug	none	Skeletal α-Actin activity	1.2	24hr
	cardiac myocyte+Δ56Fos+Fos 10ug	none	Skeletal α-Actin activity	0.5	24hr
	cardiac myocyte+Δ56Fos+Jun 1ug	none	Skeletal α-Actin activity	1	24hr
	cardiac myocyte+Δ56Fos+Jun 5ug	none	Skeletal α-Actin activity	1.1	24hr
	cardiac myocyte+Δ56Fos+Jun 10ug	none	Skeletal α-Actin activity	1.3	24hr
	cardiac myocyte+Δ56Fos+JunB 1ug	none	Skeletal α-Actin activity	0.4	24hr
	cardiac myocyte+Δ56Fos+JunB 5ug	none	Skeletal α-Actin activity	0.3	24hr
	cardiac myocyte+Δ56Fos+JunB 10ug	none	Skeletal α-Actin activity	0.2	24hr
	cardiac myocyte+Jun	none	Gal4 activity	0.8	24hr
	cardiac myocyte+Gal4-SRF(256-508)	none	Gal4 activity	8	24hr
	cardiac myocyte+Gal4-SRF(256-508)+Jun	none	Gal4 activity	9	24hr
24152730	Estrogen regulates histone deacetylases to prevent cardiac hypertrophy				
	NRCM	none	HDAC4 mRNA expression	5	24hr
	NRCM	Ang II	HDAC4 mRNA expression	1.5	24hr
	NRCM	ET-1	HDAC4 mRNA expression	2.5	24hr
	NRCM	none	HDAC5 mRNA	7	24hr

			expression		
	NRCM	Ang II	HDAC5 mRNA expression	2	24hr
	NRCM	ET-1	HDAC5 mRNA expression	3	24hr
	NRCM	none	HDAC4 protein expression	12	24hr
	NRCM	Ang II	HDAC4 protein expression	4	24hr
	NRCM	ET-1	HDAC4 protein expression	3.8	24hr
	NRCM	none	HDAC5 protein expression	13	24hr
	NRCM	Ang II	HDAC5 protein expression	3	24hr
	NRCM	ET-1	HDAC5 protein expression	3	24hr
	NRCM	none	HDAC4 mRNA expression	12	24hr
	NRCM	Ang II	HDAC4 mRNA expression	4	24hr
	NRCM	none	HDAC5 mRNA expression	8	24hr
	NRCM	Ang II	HDAC5 mRNA expression	3	24hr
	NRCM	none	pHDAC4 S632	1000	24hr
	NRCM	Ang II	pHDAC4 S632	3700	24hr
	NRCM	Ang II+BAPTA	pHDAC4 S632	1200	24hr
	NRCM	BAPTA 5mM	pHDAC4 S632	1400	24hr
	NRCM	Ang II+EGTA	pHDAC4 S632	1300	24hr
	NRCM	EGTA 3mM	pHDAC4 S632	1500	24hr
	NRCM	none	GAPDH	2.3	24hr
	NRCM	Ang II	GAPDH	2.3	24hr

	NRCM	Ang II+E2	GAPDH	2.4	24hr
	NRCM	Ang II+DPN	GAPDH	2	24hr
	NRCM	E2	GAPDH	2.3	24hr
	NRCM	DPN	GAPDH	2.3	24hr
	NRCM	none	HDAC2 mRNA expression	1	24hr
	NRCM	Ang II	GATA4 transcriptional activity	2.8*	24hr
18757826	Shp2 Negatively Regulates Growth in Cardiomyocytes by Controlling Focal Adhesion Kinase/Src and mTOR Pathways				
	NRVM	no stretch	pFAK	100	0min
	NRVM	stretch 10min	pFAK	150*	10min
	NRVM	stretch 30min	pFAK	180*	30min
	NRVM	stretch 60min	pFAK	190*	60min
	NRVM	no stretch	SHP2	100	0min
	NRVM	stretch 10min	SHP2	15*	10min
	NRVM	stretch 30min	SHP2	50*	30min
	NRVM	stretch 60min	SHP2	35*	60min
	NRVM+anti-SHP2	no stretch	SHP2	100	60min
	NRVM+anti-FAK	no stretch	SHP2	25*	60min
	NRVM+anti-FAK	stretch 60min	SHP2	15*	60min
	NRVM	no stretch	pFAK	100	
	NRVM+PTP-SHP2	no stretch	pFAK	10*	
	NRVM	stretch	pFAK	160*	
	NRVM+PTP-SHP2	stretch	pFAK	25*	
	NRVM	no stretch	pSHP2	100	0min
	NRVM	stretch 10min	pSHP2	30*	10min
	NRVM	stretch 30min	pSHP2	60*	30min
	NRVM	stretch 60min	pSHP2	50*	60min
	NRVM	no stretch	pFAK	100	0min
	NRVM	stretch 10min	pFAK	45*	10min

	NRVM	stretch 30min	pFAK	65*	30min
	NRVM	stretch 60min	pFAK	62*	60min
	NRVM	no stretch	pSHP2 Tyr542	100	0min
	NRVM	stretch 10min	pSHP2 Tyr542	130*	10min
	NRVM	stretch 60min	pSHP2 Tyr542	180	60min
	NRVM	none	SHP2	100	
	NRVM	Lipofectamine	SHP2	120	
	NRVM+si-GFP	none	SHP2	100	
	NRVM+si-SHP2	none	SHP2	10*	
	NRVM	none	p Anti-SHP2	100	
	NRVM	Lipofectamine	p Anti-SHP2	85	
10747897	Stress-induced activation of protein kinase CK2 by direct interaction with p38 mitogen-activated protein kinase				
	HeLa cell+ CK2	none	p38α-stimulated casein phosphotransferase activity of CK2	480	30min
	HeLa cell+ CK2+GST-p38	none	p38α-stimulated casein phosphotransferase activity of CK2	1600	30min
	HeLa cell+ CK2+GST-p38	SB203580	p38α-stimulated casein phosphotransferase activity of CK2	490	30min
	HeLa cell+ CK2+GST-p38	DRB	p38α-stimulated casein phosphotransferase activity of CK2	500	30min

	HeLa cell+ CK2+GST-p38	Heparin	p38α-stimulated casein phosphotransferase activity of CK2	220	30min
	HeLa cell+GST-p38	none	p38α-stimulated casein phosphotransferase activity of CK2	10	30min
9814969	Regulation of angiotensin II-induced JAK2 tyrosine phosphorylation: roles of SHP-1 and SHP-2				
	VSMC+Rabbit IgG	Ang II 0/5/10/30/60min	pJAK2	0.4/2.6/3.1/2.0/0.4	0/5/10/30/60min
	VSMC+Anti SHP2 Ab	Ang II 0/5/10/30/60min	pJAK2	0.4/0.4/0.5/0.4/0.4	0/5/10/30/60min
	VSMC	none	Cell proliferation	0.24/0.28/0.29/0.30/0.28	0/7/12/24/48hr
	VSMC	Ang II	Cell proliferation	0.23/0.28/0.50/0.60/0.63	0/7/12/24/48hr
	VSMC+anti SHP2 Ab	Ang II	Cell proliferation	0.20/0.25/0.30/0.31/0.29	0/7/12/24/48hr
11818507	Requirement of Ca(2+) and PKCdelta for Janus kinase 2 activation by angiotensin II: involvement of PYK2				
	VSMC	none	pJAK2	1	3min
	VSMC	Ang II	pJAK2	4	3min
	VSMC	none	pJAK2	0.2	3min
	VSMC	Ang II+GF109203X pretx 30min	pJAK2	0.7	3min
	VSMC	none	pJAK2	1	3min
	VSMC	Ang II	pJAK2	5	3min
	VSMC	none	pJAK2	0.8	3min

	VSMC	Ang II+Rottle pretx 30min	pJAK2	1.2	3min
	VSMC	none	pJAK2	1	3min
	VSMC	Ang II	pJAK2	5	3min
	VSMC	none	pJAK2	2	3min
	VSMC	Ang II+Go6976 pretx 30min	pJAK2	4	3min
16687399	Scaffolding Protein Grb2-associated Binder 1 Sustains Epidermal Growth Factor-induced Mitogenic and Survival Signaling by Multiple Positive Feedback Loops				
	HEK293	none	pAkt	1	0min
	HEK293	EGF	pAkt	3	1min
	HEK293	EGF	pAkt	2	5min
	HEK293	EGF	pAkt	1.5	10min
	HEK293	wortmannin	pAkt	0	0min
	HEK293	EGF+wortmannin	pAkt	0	1min
	HEK293	EGF+wortmannin	pAkt	0	5min
	HEK293	EGF+wortmannin	pAkt	0	10min
	HEK293	EGF	ppERK	0/1.5/7.0/6.0/4.5/2.0	0/1/3/5/10/30min
	HEK293	EGF+wortmannin	ppERK	0/0/0.8/1.5/1.2/0.3	0/1/3/5/10/30min
	HEK293	none	pEGFR	1	0min
	HEK293	EGF	pEGFR	3	1min
	HEK293	EGF	pEGFR	2	5min
	HEK293	EGF	pEGFR	1.5	10min
	HEK293	wortmannin	pEGFR	1	0min
	HEK293	EGF+wortmannin	pEGFR	3.5	1min
	HEK293	EGF+wortmannin	pEGFR	2	5min
	HEK293	EGF+wortmannin	pEGFR	1.5	10min
	HEK293	EGF	ppERK	0/0/0.45/0.6/0.75/1.2/0.9/ 0.28	0/1/3/4/5/7/10/15/30min

				/0.23	
	HEK293	EGF+wortmannin	ppERK	0/0/0/0/0/0.05/0.3/0.05/0.05	0/1/3/4/5/7/10/15/30min
	HEK293	none	pAkt	1	0min
	HEK293	EGF	pAkt	2	5min
	HEK293	U0126	pAkt	3	0min
	HEK293	U0126+EGF	pAkt	4	5min
	HEK293	IT-5	pAkt	2	0min
	HEK293	IT-5+EGF	pAkt	3.5	5min
	HEK293	EGF	pAkt	1.0/1.3/2.5/2.2/1/0.8	0/1/3/5/10/30min
	HEK293	EGF+IT-5	pAkt	2.0/3.0/5.0/5.0/4.5/3.0	0/1/3/5/10/30min
	HEK293	none	pGab1	0.1	0min
	HEK293	EGF	pGab1	5	5min
	HEK293	IT-5	pGab1	0.1	0min
	HEK293	IT-5+EGF	pGab1	8	5min
	HEK293	U0126	pGab1	0.1	0min
	HEK293	U0126+EGF	pGab1	10	5min
	HEK293	none	pGab1	0/280/170/120/90/70/50/25	0/2/5/10/15/20/25/30min
	HEK293	wortmannin	pGab1	0/150/30/25/25/25/25/25	0/2/5/10/15/20/25/30min
	HEK293	none	Gab-SHP2 complex	0/43/23/10/8/5/3/1	0/2/5/10/15/20/25/30min
	HEK293	wortmannin	Gab-SHP2 complex	0/18/2/1/1/1/1/1	0/2/5/10/15/20/25/30min

	HEK293	none	ppERK	0/70/75/50/42/40/35/20	0/2/5/10/15/20/25/30min
	HEK293+Gab1ΔSHP2	none	ppERK	0/60/45/20/20/20/20/20	0/2/5/10/15/20/25/30min
19713680	Angiotensin II induces cardiomyocyte hypertrophy probably through histone deacetylases				
	NRCM	none	HDAC2 mRNA	0.12	12hr
	NRCM	Ang II	HDAC2 mRNA	0.35	12hr
	NRCM	Ang II+VPA	HDAC2 mRNA	0.23	12hr
	NRCM	none	β-MHC mRNA	0.16	12hr
	NRCM	Ang II	β-MHC mRNA	0.36	12hr
	NRCM	Ang II+VPA	β-MHC mRNA	0.24	12hr
	NRCM	none	% of HDAC2 positive CM	1.25	12hr
	NRCM	Ang II	% of HDAC2 positive CM	2.2	12hr
	NRCM	Ang II+VPA	% of HDAC2 positive CM	1.6	12hr
	NRCM	none	% of c-fos positive CM	1.2	12hr
	NRCM	Ang II	% of c-fos positive CM	2.4	12hr
	NRCM	Ang II+VPA	% of c-fos positive CM	1.6	12hr
	NRCM	none	Surface area of CM	40	12hr
	NRCM	Ang II	Surface area of CM	110	12hr
	NRCM	Ang II+VPA	Surface area of CM	85	12hr
8526802	Protein tyrosine phosphorylation induced by epidermal growth factor and insulin-like growth factor-1 in a rat clonal dental pulp-cell line				

	RDP 4-1cells	none	proliferation	0.45	48h
	RDP 4-1cells	EGF 0.1ng/ml	proliferation	0.485	48h
	RDP 4-1cells	EGF 1ng/ml	proliferation	0.53	48h
	RDP 4-1cells	EGF 10ng/ml	proliferation	0.565	48h
	RDP 4-1cells	EGF 100ng/ml	proliferation	0.575	48h
	RDP 4-1cells	none	proliferation	0.45	48h
	RDP 4-1cells	IGF-1 0.1ng/ml	proliferation	0.452	48h
	RDP 4-1cells	IGF-1 1ng/ml	proliferation	0.465	48h
	RDP 4-1cells	IGF-1 10ng/ml	proliferation	0.515	48h
	RDP 4-1cells	IGF-1 100ng/ml	proliferation	0.527	48h
2472219	PLC-gamma is a substrate for the PDGF and EGF receptor protein-tyrosine kinases in vivo and in vitro				
	NIH 3T3 cells	none	pPLBbeta1	1	5min
	NIH 3T3 cells	PDGF(2nM)	pPLBbeta1	1.1	5min
	NIH 3T3 cells	none	pPLCgamma	1	5min
	NIH 3T3 cells	PDGF(2nM)	pPLCgamma	9	5min
	NIH 3T3 cells	none	pPLCdelta	1	5min
	NIH 3T3 cells	PDGF(2nM)	pPLCdelta	1	5min
	NIH 3T3 cells	none	pPLCgamma	1	15min
	NIH 3T3 cells	EGF(rp-saturating amount)	pPLCgamma	1.45	15min
	NIH 3T3 cells	PDGF(2nM)	pPLCgamma	10/20/30/42/58/100/80/50	0/2/4/6/8/10/30/60
	Swiss 3T3 cells	PDGFR	pPLCgamma	positive	
	A431 cells	EGFR	pPLCgamma	positive	
10559227	ErbB2 and ErbB3 receptors mediate inhibiton of calcium-dependent chloride secretion in colonic epithelial cells				
	T84 cells+CCh	none	Isc(microA/cm2)	33	20min
	T84 cells+CCh	EGF	Isc(microA/cm2)	15	20min
	T84 cells	EGF	p-EGFR	12.5	control
	T84 cells	EGF	p-EGFR	190	1min
	T84 cells	EGF	p-EGFR	195	5min

	T84 cells	EGF	p-EGFR	208	15min
	T84 cells	EGF	p-ErbB2	10	control
	T84 cells	EGF	p-ErbB2	108	1min
	T84 cells	EGF	p-ErbB2	106	5min
	T84 cells	EGF	p-ErbB2	114	15min
	T84 cells	EGF	p-ErbB3	3	control
	T84 cells	EGF	p-ErbB3	9	1min
	T84 cells	EGF	p-ErbB3	3	5min
	T84 cells	EGF	p-ErbB3	7	15min
	T84 cells	EGF(100ng/ml)	ErbB2+EGFR	0/1/0.7/0.7	0/1/5/15min
	T84 cells	EGF	PI3K+EGFR	0.1/1/0.8/0.6	0/1/5/15min
	T84 cells	EGF	PI3K+ErbB2	0/0/0/0	0/1/5/15min
17082637	A JNK-independent signaling pathway ragulates TNF alpha-stimulated, c-Jun-Driven FRA-1 protooncogene transcription in pulmonary epithelial cells				
	pulmonary epithelial cells	TNFa	p-JNK1(activation)	0	0min
	pulmonary epithelial cells	TNFa	p-JNK1(activation)	1	15min
	pulmonary epithelial cells	TNFa	p-JNK1(activation)	1.5	30min
	pulmonary epithelial cells	TNFa	p-JNK1(activation)	1.7	60min
	pulmonary epithelial cells	TNFa	p-c-JUN/c-JUN	0	0min
	pulmonary epithelial cells	TNFa	p-c-JUN/c-JUN	0.3	15min
	pulmonary epithelial cells	TNFa	p-c-JUN/c-JUN	0.4	30min
	pulmonary epithelial cells	TNFa	p-c-JUN/c-JUN	0.5	60min
	pulmonary epithelial cells	TNFa	p-c-JUN/c-JUN	1.2	120min
	pulmonary epithelial cells	TNFa	p-CREB	1.5	15min
	pulmonary epithelial cells	TNFa	p-CREB	2.7	30min
	pulmonary epithelial cells	TNFa	p-ATF1	1	0min
	pulmonary epithelial cells	TNFa	p-ATF1	2.7*	15min

	pulmonary epithelial cells	TNFa	p-ATF1	1.2	30min
	pulmonary epithelial cells + DMSO	TNFa	p-Elk-1/Elk-1	1	30min
	pulmonary epithelial cells + PD98059	TNFa	p-Elk-1/Elk-1	0.5	30min
	pulmonary epithelial cells + DMSO	TNFa	p-CREB	1.7	30min
	pulmonary epithelial cells + PD98059	TNFa	p-CREB	0.9	30min
	pulmonary epithelial cells + DMSO	TNFa	p-ATF1	1.1	30min
	pulmonary epithelial cells + PD98059	TNFa	p-ATF1	0.5	30min
11585926	The transcription factor GATA-4 is activated by extracellular signal-regulated kinase1- and 2-Mediated phosphorylation of serine 105 in cardiomyocytes				
	cardiomyocytes + Ad beta gal TF	PE(10uM)	p-GATA4/GATA4	1	0hr
	cardiomyocytes + Ad beta gal TF	PE(10uM)	p-GATA4/GATA4	2	1hr
	cardiomyocytes + Ad beta gal TF	PE(10uM)	p-GATA4/GATA4	2	3hr
	cardiomyocytes + Ad beta gal TF	PE(10uM)	p-GATA4/GATA4	2.5	24hr
	cardiomyocytes + Ad GATA4 TF	PE(10uM)	p-GATA4/GATA4	3.5	0hr
	cardiomyocytes + Ad GATA4 TF	PE(10uM)	p-GATA4/GATA4	5	1hr
	cardiomyocytes + Ad GATA4 TF	PE(10uM)	p-GATA4/GATA4	8	3hr
	cardiomyocytes + Ad GATA4 TF	PE(10uM)	p-GATA4/GATA4	10	24hr
	NRVM	Saline	p-GATA4/GATA4	1	3hr
	NRVM	PE	p-	3	3hr

			GATA4/GATA4		
	NRVM + Ad beta-gal TF	none	p-GATA4/GATA4	1	24h
	NRVM + Ad MEK1 TF	none	p-GATA4/GATA4	5	24h
	NRVM + Ad MKK7 TF	none	p-GATA4/GATA4	2	24h
	NRVM + Ad MKK6 TF	none	p-GATA4/GATA4	3	24h
	NRVM	beta-gal	p-GATA4 fold increase	1	0hr
	NRVM	beta-gal +PE	p-GATA4 fold increase	3.3	3hr
	NRVM	dnMEK1+PE	p-GATA4 fold increase	0.75	3hr
	NRVM	dnMKK4+PE	p-GATA4 fold increase	2.78	3hr
	NRVM	dnMKK3+PE	p-GATA4 fold increase	3.5	3hr
	NRVM+MBP	none	p-GATA4	0	20min
	NRVM	beta-gal	ANF	17	2h TF + 24h medium
	NRVM	MEK1	ANF	64	2h TF + 24h medium
	NRVM	MEK1+Engr	ANF	62	2h TF + 24h medium
	NRVM	MEK1+G4-Engr	ANF	30.5	2h TF + 24h medium
	NRVM	MKK7	ANF	67	2h TF + 24h medium
	NRVM	MKK7+Engr	ANF	62.5	2h TF + 24h medium
	NRVM	MKK7+G4-Engr	ANF	64	2h TF + 24h medium
10788473 Ad CnA : A replication-deficient adenovirus	Calcineurin Promotes PKC and c-JUN NH2-terminal Kinase Activation in the heart				
	None TG mice	none	p-JNK(p54)	1	14 day mice
	Calcineurin TG mice	none	p-JNK(p54)	1.7	14 day mice
	None TG mice	none	p-JNK(p54)	1	25 day mice
	Calcineurin TG mice	none	p-JNK(p54)	1.7	25 day mice

expressing a constitutively activated form of CaN	None TG mice	none	p-p38	1	14 day mice
	Calcineurin TG mice	none	p-p38	0.8	14 day mice
	None TG mice	none	p-p38	1	25 day mice
	Calcineurin TG mice	none	p-p38	0.9	25 day mice
	None TG mice	none	p-ERK1/2	1	14 day mice
	Calcineurin TG mice	none	p-ERK1/2	2	14 day mice
	None TG mice	none	p-ERK1/2	1	25 day mice
	Calcineurin TG mice	none	p-ERK1/2	2	25 day mice
	None TG mice	none	JNK activity	1	24hr
	Calcineurin TG mice	none	JNK activity	1.7	24hr
	None TG mice	none	ERK 1/2 activity	1	24hr
	Calcineurin TG mice	none	ERK 1/2 activity	1.9	24hr
	Calcineurin TG mice / None TG mice	none	PKC activity	1.4	4 day
	Calcineurin TG mice / None TG mice	none	PKC activity	2	8 day
	Calcineurin TG mice / None TG mice	none	PKC activity	2.4	14 day
	Calcineurin TG mice / None TG mice	none	PKC activity	1.4	25 day
	None TG mice	none	PKC alpha particulate	0.451612903	24hr
	Calcineurin TG mice	none	PKC alpha particulate	0.694444444	24hr
	None TG mice	none	PKC beta-1 particulate	0.751879699	24hr
	Calcineurin TG mice	none	PKC beta-1 particulate	0.862068966	24hr
	None TG mice	none	PKC epsilon cytosol	1.173913043	24hr
	Calcineurin TG mice	none	PKC epsilon cytosol	0.811111111	24hr
	None TG mice	none	PKC theta	0.75	24hr

			cytosol		
	Calcineurin TG mice	none	PKC theta cytosol	1.6	24hr
	None TG mice	none	PKC beta-2 cytosol	0.8666666 67	24hr
	Calcineurin TG mice	none	PKC beta-2 cytosol	2	24hr
	None TG mice	none	PKC lambda cytosol	4	24hr
	Calcineurin TG mice	none	PKC lambda cytosol	0.9230769 23	24hr
	Cardiomyocyte + Ad beta gal TF	none	CellArea(um2)	1250	24hr
	Cardiomyocyte + Ad CnA TF	none	CellArea(um2)	2200	24hr
	Cardiomyocyte + Ad CnA TF	Chelerythrine 2.5uM	CellArea(um2)	1300	24hr
	Cardiomyocyte + Ad CnA TF	PMA 1uM	CellArea(um2)	1400	24hr
	Cardiomyocyte + Ad CnA TF	PD098059 25uM	CellArea(um2)	2000	24hr
	Cardiomyocyte + Ad CnA TF	SB202190 20uM	CellArea(um2)	2100	24hr
	Cardiomyocyte + Ad CnA TF	BDM 7mM	CellArea(um2)	2000	24hr
	Cardiomyocyte + Ad CnA TF	BAPTA 2.5uM	CellArea(um2)	1080	24hr
10849446	Big mitogen-activated kinase regulates multiple members tof the mef2 protein family				
	in vitro protein kinase assay	BMK1	pMEF2A	positive	1hr
	in vitro protein kinase assay	BMK1	pMEF2B	neutral	1hr
	in vitro protein kinase assay	BMK1	pMEF2C	positive	1hr
	in vitro protein kinase assay	BMK1	pMEF2D	positive	1hr
	in vitro protein kinase assay	BMK1	MBP	neutral	1hr
	in vitro protein kinase assay	BMK1	c-jun	neutral	1hr
	HeLa	None	control	1	48hr
	HeLa	MEK5D+BMK1	control	0.8	48hr
	HeLa	None	MEF2A	1.5	48hr
	HeLa	MEK5D+BMK1	MEF2A	21.5	48hr
	HeLa	None	MEF2B	0.5	48hr

	HeLa	MEK5D+BMK1	MEF2B	0.3	48hr
	HeLa	None	MEF2C	1	48hr
	HeLa	MEK5D+BMK1	MEF2C	19	48hr
	HeLa	None	MEF2D	1.5	48hr
	HeLa	MEK5D+BMK1	MEF2D	26.5	48hr
	Chinese hamster ovary	None	control	1	48hr
	Chinese hamster ovary	MEK5D+BMK1	control	0.8	48hr
	Chinese hamster ovary	None	MEF2A	1.5	48hr
	Chinese hamster ovary	MEK5D+BMK1	MEF2A	25	48hr
	Chinese hamster ovary	None	MEF2B	0.7	48hr
	Chinese hamster ovary	MEK5D+BMK1	MEF2B	0.8	48hr
	Chinese hamster ovary	None	MEF2C	1.5	48hr
	Chinese hamster ovary	MEK5D+BMK1	MEF2C	32	48hr
	Chinese hamster ovary	None	MEF2D	0.9	48hr
	Chinese hamster ovary	MEK5D+BMK1	MEF2D	38	48hr
	293	None	control	1	48hr
	293	MEK5D+BMK1	control	1	48hr
	293	None	MEF2A	1	48hr
	293	MEK5D+BMK1	MEF2A	190	48hr
	293	None	MEF2B	1	48hr
	293	MEK5D+BMK1	MEF2B	5	48hr
	293	None	MEF2C	1	48hr
	293	MEK5D+BMK1	MEF2C	175	48hr
	293	None	MEF2D	1	48hr
	293	MEK5D+BMK1	MEF2D	120	48hr
	PC12	None	control	1	48hr
	PC12	MEK5D+BMK1	control	0.8	48hr
	PC12	None	MEF2A	1.2	48hr
	PC12	MEK5D+BMK1	MEF2A	4.4	48hr
	PC12	None	MEF2B	1.2	48hr

	PC12	MEK5D+BMK1	MEF2B	0.6	48hr
	PC12	None	MEF2C	1.3	48hr
	PC12	MEK5D+BMK1	MEF2C	6	48hr
	PC12	None	MEF2D	1.5	48hr
	PC12	MEK5D+BMK1	MEF2D	4.8	48hr
	HeLa cells	none	control	1	6hr
	HeLa cells	EGF	control	1.15	6hr
	HeLa cells	none	MEF2A	1.2	6hr
	HeLa cells	EGF	MEF2A	3.7	6hr
	HeLa cells + ERK2 (0.2ug) inactive TF	none	MEF2D	100	20min
	HeLa cells + ERK2 (0.3ug) inactive TF	none	MEF2D	80	20min
	HeLa cells	none	MEF2D	100	20min
	HeLa cells + p38 (0.1ug) inactive TF	none	MEF2D	115	20min
	HeLa cells + p38 (0.2ug) inactive TF	none	MEF2D	110	20min
	HeLa cells + p38 (0.3ug) inactive TF	none	MEF2D	97	20min
	BMK1 phosphrylates MEF2A at amino acids Ser-355, Thr-312, Thr-319				
	HeLa+pG5ElbLuc+pcDNA3	None	MEF2A	1	24h
	HeLa+pG5ElbLuc+pcDNA3	MEK5D+BMK1	MEF2A	0.8	24h
	HeLa+pG5ElbLuc+wt	None	MEF2A	1.2	24h
	HeLa+pG5ElbLuc+wt	MEK5D+BMK1	MEF2A	21	24h
	HeLa+pG5ElbLuc+MEF2A(S355A)	None	MEF2A	0.5	24h
	HeLa+pG5ElbLuc+MEF2A(S355A)	MEK5D+BMK1	MEF2A	11	24h
	HeLa+pG5ElbLuc+MEF2A(T312A,T319A)	None	MEF2A	0.8	24h
	HeLa+pG5ElbLuc+MEF2A(	MEK5D+BMK1	MEF2A	8.5	24h

	T312A,T319A)				
	HeLa+pG5ElbLuc+MEF2A(S355A/T312A,T319A)	None	MEF2A	1	24h
	HeLa+pG5ElbLuc+MEF2A(S355A/T312A,T319A)	MEK5D+BMK1	MEF2A	1.8	24h
	HeLa+pG5ElbLuc+pcDNA3	None	MEF2A	1	20min
	HeLa+pG5ElbLuc+pcDNA3	EGF	MEF2A	1	20min
	HeLa+pG5ElbLuc+wt	None	MEF2A	0.8	20min
	HeLa+pG5ElbLuc+wt	EGF	MEF2A	2.6	20min
	HeLa+pG5ElbLuc+MEF2A(S355A)	None	MEF2A	0.75	20min
	HeLa+pG5ElbLuc+MEF2A(S355A)	EGF	MEF2A	1.3	20min
	HeLa+pG5ElbLuc+MEF2A(T312A,T319A)	None	MEF2A	0.9	20min
	HeLa+pG5ElbLuc+MEF2A(T312A,T319A)	EGF	MEF2A	1.4	20min
	HeLa+pG5ElbLuc+MEF2A(S355A/T312A,T319A)	None	MEF2A	0.6	20min
	HeLa+pG5ElbLuc+MEF2A(S355A/T312A,T319A)	EGF	MEF2A	0.5	20min
	HeLa+pG5ElbLuc+pcDNA3	None	MEF2D	1	24h
	HeLa+pG5ElbLuc+pcDNA3	MEK5D+BMK1	MEF2D	1.2	24h
	HeLa+pG5ElbLuc+wt	None	MEF2D	1.1	24h
	HeLa+pG5ElbLuc+wt	MEK5D+BMK1	MEF2D	26.5	24h
	HeLa+pG5ElbLuc+MEF2D(S179A)	None	MEF2D	0.9	24h
	HeLa+pG5ElbLuc+MEF2D(S179A)	MEK5D+BMK1	MEF2D	0.5	24h
	HeLa+pG5ElbLuc+MEF2D(S430A)	None	MEF2D	1.5	24h
	HeLa+pG5ElbLuc+MEF2D(S430A)	MEK5D+BMK1	MEF2D	28.5	24h

	HeLa+pG5ElbLuc+pcDNA3	None	MEF2D	1	20min
	HeLa+pG5ElbLuc+pcDNA3	EGF	MEF2D	0.8	20min
9075635	Endothelin-1 is a potent regulator of human bone cell metabolism				
	osteoblastic cells+ETRAnt(10ng/ml)	None	Cell number(%)	75	24h
	osteoblastic cells+ETRAnt(10ng/ml)	Endothelin-1 1pg/ml	Cell number(%)	87.5	24h
	osteoblastic cells+ETRAnt(10ng/ml)	Endothelin-1 10pg/ml	Cell number(%)	120	24h
	osteoblastic cells+ETRAnt(10ng/ml)	Endothelin-1 100pg/ml	Cell number(%)	100	24h
	osteoblastic cells+ETRAnt(10ng/ml)	Endothelin-1 1000pg/ml	Cell number(%)	130	24h
	osteoblastic cells+ETRAnt(10ng/ml)	Endothelin-1 10000pg/ml	Cell number(%)	165	24h
	osteoblastic cells	Endothelin-1 1pg/ml	Cell number(%)	125	24h
	osteoblastic cells	Endothelin-1 10pg/ml	Cell number(%)	167	24h
	osteoblastic cells	Endothelin-1 100pg/ml	Cell number(%)	243	24h
	osteoblastic cells	Endothelin-1 1000pg/ml	Cell number(%)	267	24h
	osteoblastic cells	Endothelin-1 10000pg/ml	Cell number(%)	320	24h
9223382	A paracrine Role for myoepithelial cell-derived FGF2 in the normal human breast				
	Breast epithelial cells(BCM)	none	prolif of Breast ep cell	0.08	6 day
	Breast epithelial cells(BCM)+1%FCS	none	prolif of Breast ep cell	0.06	6 day
	Breast epithelial cells(BCM)+2%FCS	none	prolif of Breast ep cell	0.07	6 day
	Breast epithelial cells(BCM)+5%FCS	none	prolif of Breast ep cell	0.09	6 day
	Breast epithelial cells(BCM)+10%FCS	none	prolif of Breast myoep cell	0.45	6 day

	Breast epithelial cells(BCM)	FGF2(10ng/ml)	prolif of Breast myoep cell	1.7	6 day
	Breast epithelia cells(BCM)+10%FCS	FGF2(10ng/ml)	prolif of Breast myoep cell	0.47	6 day
	Breast epithelial cells(BCM)	none	prolif of Breast myoep cell	0.25	6 day
	Breast epithelial cells(BCM)	none	prolif of Breast myoep cell	1.65	6 day
	Breast epithelial cells(BCM)+0.5%FCS	none	prolif of Breast myoep cell	1.55	6 day
	Breast epithelial cells(BCM)+10%FCS	none	prolif of Breast myoep cell	1.5	6 day
	Breast epithelial cells(BCM)	FGF2(10ng/ml)	prolif of Breast myoep cell	1.5	6 day
	Breast epithelia cells(BCM)+10%FCS	FGF2(10ng/ml)	prolif of Breast myoep cell	1.65	6 day
10362356	Contribution of Src and Ras pathways in FGF-2 induced endothelial celldifferentiation				
	IBEC	none	cell Number(*10000)	4.6	3days
	IBEC	FGF	cell Number(*10000)	11.4	3days
	IBEC	FGF + Manumycin	cell Number(*10000)	5	3days
	IBEC	FGF	ERK12	4	120min
	IBEC	FGF	ERK12	6	180min
	IBEC	FGF	ERK12	8	300min
	IBEC	FGF + PD98059	ERK12	0	0min
	IBEC	FGF + PD98059	ERK12	2	10min
	IBEC	FGF + PD98059	ERK12	2	60min
	IBEC	FGF + PD98059	ERK12	3	120min
	IBEC	FGF + PD98059	ERK12	3	180min
	IBEC	FGF + PD98059	ERK12	3	300min
	IBEC	none	Cell Number	5	3 days

	IBEC	FGF	Cell Number	9.3	3 days
	IBEC	FGF + PD98059	Cell Number	5.7	3 days
	IBEC	FGF + PD98059	Cell Number	3.5	3 days
	IBEC + FN	FGF	Raf1	1	0min
	IBEC + FN	FGF	Raf1	4.2	1min
	IBEC + FN	FGF	Raf1	3.82	3min
	IBEC + FN	FGF	Raf1	2.4	5min
	IBEC + FN	FGF	Raf1	1.25	10min
	IBEC + FN	FGF	Raf1	1.25	60min
	IBEC + Coll	FGF	Raf1	1	0min
	IBEC + Coll	FGF	Raf1	2.2	1min
	IBEC + Coll	FGF	Raf1	2.5	3min
	IBEC + Coll	FGF	Raf1	1.27	5min
	IBEC + Coll	FGF	Raf1	1	10min
	IBEC + Coll	FGF	Raf1	1	60min
19564249	Gq-initiated cardiomyocyte hypertrophy is mediated by phospholipase Cbeta1b				
	NRVM+Ad-GFP	none	PLC	0.75	48h
	NRVM+Ad-PLCbeta1a	none	PLC	0.7	48h
	NRVM+Ad-PLCbeta1b	none	PLC	2	48h
	NRVM+Ad-Galpha q wild type	none	PLC	1.5	48h
	NRVM+Ad-G alpha q active(Q208L)	none	PLC	4.25	48h
	NRVM	none	Cell Area(um2)	600	48h
	NRVM+Ad-PLCbeta1a	none	Cell Area(um2)	800	48h
	NRVM+Ad-PLCbeta1b	none	Cell Area(um2)	1200	48h
	NRVM+Ad-Galpha q	none	Cell Area(um2)	1800	48h
	NRVM	none	ANP mRNA	0.06	48h
	NRVM+Ad-PLCbeta1a	none	ANP mRNA	0.055	48h
	NRVM+Ad-PLCbeta1b	none	ANP mRNA	0.105	48h
	NRVM+Ad-Galpha q	none	ANP mRNA	0.205	48h

	NRVM	None	PLC	2.5	24h
	NRVM+Ad-PLCbeta1a-CT	None	PLC	2.25	24h
	NRVM+Ad-PLCbeta1b-CT	None	PLC	3.75	24h
	NRVM	NE	PLC	7	24h
	NRVM+Ad-PLCbeta1a-CT	NE	PLC	6.8	24h
	NRVM+Ad-PLCbeta1b-CT	NE	PLC	3.75	24h
	NRVM	none	PLC	5	24h
	NRVM+Ad-Galpha q	none	PLC	25	24h
	NRVM+Ad-G alpha q + Ad-PLCbeta1a-CT	none	PLC	25.5	24h
	NRVM+Ad-G alpha q +Ad-PLCbeta1b-CT	none	PLC	4.5	24h
	NRVM	none	Cell Area	800	24h
	NRVM	PE + Propranolol	Cell Area	1900	24h
	NRVM+Ad-PLCbeta1a-CT	none	Cell Area	1000	24h
	NRVM+Ad-PLCbeta1a-CT	PE + Propranolol	Cell Area	2050	24h
	NRVM+Ad-PLCbeta1b-CT	none	Cell Area	1200	24h
	NRVM+Ad-PLCbeta1b-CT	PE + Propranolol	Cell Area	1000	24h
	NRVM	none	Cell Area	760	24h
	NRVM+Ad-PLCbeta1b-CT	none	Cell Area	820	24h
	NRVM+Ad-G alpha q	none	Cell Area	1220	24h
	NRVM+Ad-G alpha q+Ad-PLCbeta1b-CT	none	Cell Area	830	24h
	NRVM	none	ANP mRNA	0.016	24h
	NRVM+Ad-PLCbeta1b-CT	none	ANP mRNA	0.01	24h
	NRVM+Ad-G alpha q	none	ANP mRNA	0.07	24h

	NRVM+Ad-G alpha q+Ad-PLCbeta1b-CT	none	ANP mRNA	0.01	24h
12507995	Roles of Gbetagamma in membrane recruitment and activation of p110gamma/p101 PI3Kgamma				
	p110gamma~~p101 : dimerization of p110gamma and p101				
	HEK cells	none	Akt	1	
	HEK cells	FCS	Akt	2.5	
	HEK cells+fMLP-R TF + p110gamma	none	Akt	1	1day
	HEK cells+fMLP-R TF + p110gamma	fMLP	Akt	1.5	1day
	HEK cells+fMLP-R TF + p110gamma+p101 (PI3K)	none	Akt	1.5	1day
	HEK cells+fMLP-R TF + p110gamma+p101 (PI3K)	fMLP	Akt	2	1day
	HEK cells+p110gamma-CAAX+fMLP-R	none	ERK12	1	1day
	HEK cells+p110gamma-CAAX+fMLP-R+dn Ras	none	ERK12	0.5	1day
	HEK cells	none	Akt	1	1day
	HEK cells	FCS	Akt	2.5	1day
	HEK cells+p110gamma	none	Akt	0.2	1day
	HEK cells+p110gamma	fMLP	Akt	0.3	1day
	HEK cells+p110gamma +p101	none	Akt	0.4	1day
	HEK cells+p110gamma +p101	fMLP	Akt	1.3	1day
	HEK cells+p110gamma-CAAX	none	Akt	0.4	1day
	HEK cells+p110gamma-CAAX	fMLP	Akt	1	1day
	HEK cells+p110gamma-	none	Akt	0.3	1day

	CAAX+p101				
	HEK cells+p110gamma-CAAX+p101	fMLP	Akt	1.2	1day
14563709	Structure, Regulation, and Function of Mammalian Membrane Guanylyl Cyclase Receptors, with a Focus on Guanlylyl cyclase-A				
	cardiomyocyte(floxed GC-A mice)	none	ANP(pg/ml plasma)	220	
	cardiomyocyte(CM GC-A KO mice)	none	ANP(pg/ml plasma)	510	
	cardiomyocyte(floxed GC-A mice)	none	cGMP	20.8	
	cardiomyocyte(CM GC-A KO mice)	none	cGMP(pmol/ ml plasma)	45	
12727932	Pressure-independent cardiac hypertrophy in mice with cardiomyocyte-restricted inactivation of the atrial natriuretic peptide receptor guanylyl cyclase-A				
	cardiomyocyte(floxed GC-A mice)	none	cGMP	65	10min
	cardiomyocyte(CM GC-A KO mice)	none	cGMP	50	10min
	cardiomyocyte(floxed GC-A mice)	ANPi	cGMP	200	10min
	cardiomyocyte(CM GC-A KO mice)	ANPi	cGMP	140	10min
	cardiomyocyte(floxed GC-A mice)	ANPi	cGMP	325	10min
	cardiomyocyte(CM GC-A KO mice)	ANPi	cGMP	240	10min
	cardiomyocyte(floxed GC-A mice)	none	ANP mRNA	1	
	cardiomyocyte(CM GC-A KO mice)	none	ANP mRNA	4.9	
	cardiomyocyte(floxed GC-A mice)	none	sACT	1	
	cardiomyocyte(CM GC-A KO mice)	none	sACT	1.7	

	cardiomyocyte(floxed GC-A mice)	none	betaMHC	1	
	cardiomyocyte(CM GC-A KO mice)	none	betaMHC	2	
8921810	Activation of JAK-STAT and MAP kinases by leukemia inhibitory factor through gp130 in cardiac myocytes.				
	Cardiomyocytes	none	gp130LIFR	0	0min
	Cardiomyocytes	LIF	gp130LIFR	1	2min
	Cardiomyocytes	LIF	gp130LIFR	4	5min
	Cardiomyocytes	LIF	gp130LIFR	3	15min
	Cardiomyocytes	none	JAK1	0	0min
	Cardiomyocytes	LIF	JAK1	1	5min
	Cardiomyocytes	LIF	JAK1	2.5	15min
	Cardiomyocytes	none	STAT3	0	0min
	Cardiomyocytes	LIF	STAT3	30	5min
	Cardiomyocytes	LIF	STAT3	20	15min
	Cardiomyocytes	LIF	STAT3	10	30min
	Cardiomyocytes	LIF	STAT3	1	60min
	non-myocardial cells	none	STAT3	0	0min
	non-myocardial cells	LIF	STAT3	20	5min
	non-myocardial cells	LIF	STAT3	30	15min
	non-myocardial cells	LIF	STAT3	10	30min
	non-myocardial cells	LIF	STAT3	1	60min
	Cardiomyocytes	none	STAT3	0.01	15min
	Cardiomyocytes	LIF	STAT3	0.01	15min
	Cardiomyocytes	LIF	STAT3	1	15min
	Cardiomyocytes	LIF	STAT3	10	15min
	Neonatal murine CM + LIF (1&10^3 U/ml) 5min	none	JAK1	1	2hr
	Neonatal murine CM + LIF (1&10^3 U/ml) 5min	anti-gp130 blocking Ab	JAK1	0.2	2hr

	Neonatal murine CM + LIF (1&10^3 U/ml) 5min	none	STAT3	1	2hr
	Neonatal murine CM + LIF (1&10^3 U/ml) 5min	anti-gp130 blocking Ab	STAT3	0.05	2hr
	Cardiomyocytes	LIF	MAPK	1	0min
	Cardiomyocytes	LIF	MAPK	3	2min
	Cardiomyocytes	LIF	MAPK	4.8	5min
	Cardiomyocytes	LIF	MAPK	2.8	15min
	Cardiomyocytes	LIF	MAPK	1	30min
	Cardiomyocytes	LIF	MAPK	1.2	60min
	Cardiomyocytes	NE	MAPK	1	0min
	Cardiomyocytes	NE	MAPK	5	2min
	Cardiomyocytes	NE	MAPK	11.4	5min
	Cardiomyocytes	NE	MAPK	8	15min
	Cardiomyocytes	NE	MAPK	5.75	30min
	Cardiomyocytes	NE	MAPK	4.8	60min
	Cardiomyocytes	LIF	ERK1	1	0min
	Cardiomyocytes	LIF	ERK1	7	5min
	Cardiomyocytes	LIF	ERK1	2.5	15min
	Cardiomyocytes	LIF	ERK1	0.8	30min
	Cardiomyocytes	LIF	ERK1	0.7	60min
	Cardiomyocytes	LIF	ERK2	0	0min
	Cardiomyocytes	LIF	ERK2	6	5min
	Cardiomyocytes	LIF	ERK2	1	15min
	Cardiomyocytes	LIF	ERK2	0.5	30min
	Cardiomyocytes	LIF	ERK2	0.8	60min
	Adult Heart	LIF	gp130LIFR	1	0min
	Adult Heart	LIF	gp130LIFR	4	5min
	Adult Heart	LIF	gp130LIFR	0.5	15min
	Adult Heart	LIF	STAT3	1	0min

	Adult Heart	LIF	STAT3	4	5min
	Adult Heart	LIF	STAT3	6	15min
	Adult Heart	LIF	STAT3	2	30min
	Adult Heart	LIF	STAT3	0.5	60min
	Liver	LIF	STAT3	0	0min
	Liver	LIF	STAT3	1	5min
	Liver	LIF	STAT3	1.5	15min
	Liver	LIF	STAT3	0.02	30min
	Liver	LIF	STAT3	0	60min
	Adult Heart	IL6	STAT3	0	0min
	Adult Heart	IL6	STAT3	0	5min
	Adult Heart	IL6	STAT3	0	15min
	Adult Heart	IL6	STAT3	0	30min
	Adult Heart	NE	STAT3	0	0min
	Adult Heart	NE	STAT3	0	5min
	Adult Heart	NE	STAT3	0	15min
	Adult Heart	NE	STAT3	0	30min
	Adult Heart	LIF	MAPK	1	0min
	Adult Heart	LIF	MAPK	2.7	5min
	Adult Heart	LIF	MAPK	1.05	15min
	Adult Heart	LIF	MAPK	1	30min
12855672	Activation of gp130 Transduces Hypertrophic signal through interaction of scaffolding/docking protein Gab1 with tyrpsine phosphatase SHP2 in cardiomyocytes				
	NRVM	control	Gab1	1	5min
	NRVM	LIF	Gab1	8	5min
	NRVM	NE	Gab1	1	5min
	NRVM	ET1	Gab1	1.1	5min
	NRVM	AngII	Gab1	1.5	5min
	NRVM	control	SHP2	1	5min
	NRVM	LIF	SHP2	9	5min

	NRVM	NE	SHP2	0.5	5min
	NRVM	ET1	SHP2	0.8	5min
	NRVM	AngII	SHP2	0.8	5min
	NRVM	LIF	Gab1	0	0min
	NRVM	LIF	Gab1	1	2min
	NRVM	LIF	Gab1	10	5min
	NRVM	LIF	Gab1	5	10min
	NRVM	LIF	Gab1	0.2	30min
	NRVM	LIF	Gab1	0	60min
	NRVM	none	Gab1	1	5min
	NRVM	LIF	Gab1	2	5min
	NRVM	LIF	Gab1	4.5	5min
	NRVM	LIF	Gab1	6	5min
	NRVM	LIF	SHP2	0	0min
	NRVM	LIF	SHP2	1	2min
	NRVM	LIF	SHP2	3	5min
	NRVM	LIF	SHP2	5	10min
	NRVM	LIF	SHP2	1.5	30min
	NRVM	LIF	SHP2	0.3	60min
	NRVM	none	SHP2	1	5min
	NRVM	LIF	SHP2	1	5min
	NRVM	LIF	SHP2	3	5min
	NRVM	LIF	SHP2	5	5min
	NRVM	none	Tyrosine phospho- Gab1	1	5min
	NRVM	LIF	Tyrosine phospho- Gab1	10	5min
	NRVM	none	Tyrosine phospho- SHP2	1	5min
	NRVM	LIF	Tyrosine phospho- SHP2	50	5min

	Ad Gab1F627/659 -|(LIF->longitudinal elongation)				
	NRVM+Ad beta-gal	none	CellArea	1	24h
	NRVM+Ad beta-gal	LIF	CellArea	1.7	24h
	NRVM+Ad Gab1WT	none	CellArea	1	24h
	NRVM+Ad beta-gal	LIF	Akt	2.25	10min
	NRVM+Ad beta-gal	LIF	Akt	1.5	30min
	NRVM+Ad Gab1WT	LIF	Akt	1	0min
	NRVM+Ad Gab1WT	LIF	Akt	1.8	2min
	NRVM+Ad Gab1WT	LIF	Akt	2.1	5min
	NRVM+Ad Gab1WT	LIF	Akt	3.8	10min
	NRVM+Ad Gab1WT	LIF	Akt	1.78	30min
	NRVM+Ad Gab1F627/659	LIF	Akt	1	0min
	NRVM+Ad Gab1F627/659	LIF	Akt	1.8	2min
	NRVM+Ad Gab1F627/659	LIF	Akt	2.75	5min
	NRVM+Ad Gab1F627/659	LIF	Akt	4.1	10min
	NRVM+Ad Gab1F627/659	LIF	Akt	2.75	30min
	NRVM+Ad Gab1WT+Ad beta-gal	none	CellArea	1	24h
	NRVM+Ad Gab1WT+Ad beta-gal	LIF	CellArea	1.6	24h
	NRVM+Ad Gab1WT+Ad ERK5AEF	none	CellArea	1	24h
	NRVM+Ad Gab1WT+Ad ERK5AEF	LIF	CellArea	1.07	24h
8557975	MEK1 and the extracellular signal-regulated kinases are required for the stimulation of IL-2 gene transcription in T cells.				
	Jurkat T cells	anti-CD3 mAb	MEK1	0.6/2.0/1.6/1.4/1.0	0/5/10/15/60/90min
	Jurkat T cells	anti-CD3 mAb	ERK2	0.4/1.3/2.4/1.3/1.1/0.45	0/5/10/15/60/90min
11387209	Activated MEK5 induces serial assembly of sarcomeres and eccentric cardiac hypertrophy				
	NR cardiomyocytes	100 microM PE	ERK5	1	0min

	NR cardiomyocytes	100 microM PE	ERK5	2.75	5min
	NR cardiomyocytes	100 microM PE	ERK5	3.75	10min
	NR cardiomyocytes	100 microM PE	ERK5	2.5	20min
	NR cardiomyocytes	100 microM PE	ERK5	1.5	60min
	NR cardiomyocytes	1000U/ml LIF	ERK5	1	0min
	NR cardiomyocytes	1000U/ml LIF	ERK5	2.2	5min
	NR cardiomyocytes	1000U/ml LIF	ERK5	3	10min
	NR cardiomyocytes	1000U/ml LIF	ERK5	2	20min
	NR cardiomyocytes	1000U/ml LIF	ERK5	0.85	60min
	NR cardiomyocytes				
	Cardiomyocytes + MEK5WT TF	PE(50uM)	sACT	8	24h
	Cardiomyocytes + dnMEK5 TF	PE(50uM)	sACT	2.8	24h
	Cardiomyocytes + MEK5WT TF	LIF(1000U/ml)	sACT	4.8	24h
	Cardiomyocytes + dnMEK5 TF	LIF(1000U/ml)	sACT	1	24h
	Cardiomyocytes + beta-galactosidase TF	PE(50uM)	sACT	5	24h
	Cardiomyocytes + beta-galactosidase TF	LIF(1000U/ml)	sACT	4	24h
	Cardiomyocytes + MEK5WT TF	PE(50uM)	ANF	5.75	24h
	Cardiomyocytes + dnMEK5 TF	PE(50uM)	ANF	2	24h
9751683	Norepinephrine stimulates apoptosis in adult rat ventricular myoctes by activation of the beta-AR pathway				
	ARVM(adult rat ventricular myocytes)	control	Apoptosis(%of t=0)	10	24h
	ARVM	NE(10microM/L)	Apoptosis(%of t=0)	36	24h
	ARVM	PRO(2microM/L)+NE	Apoptosis(%of t=0)	14	30minPRO 24hNE

	ARVM	PZ(0.1microM/L)+NE	Apoptosis(%of t=0)	42	30minPZ 24hNE
	ARVM	control	Apoptosis(%)	5.7	30min
	ARVM	NE(10microM/L)	Apoptosis(%)	17	30min
	ARVM	NE+PRO(2microM/L)	Apoptosis(%)	7	30min
	ARVM	NE+PZ(0.1microM/L)	Apoptosis(%)	14	30min
	ARVM	control	Apoptosis(%)	7	24h
	ARVM	ISO(10mciroM/L)	Apoptosis(%)	14.6	24h
	ARVM	FSK(10microM/L)	Apoptosis(%)	14.375	24h
	ARVM	control	Apoptosis(%)	7	Time Course
	ARVM	NE(10microM/L)	Apoptosis(%)	10.625	
	ARVM	H-89(20microM/L)	Apoptosis(%)	4.28	
	ARVM	NE+H-89(20microM/L)	Apoptosis(%)	5.3	
	ARVM	control	Apoptosis(%)	8.75	
	ARVM	NE(10microM/L)	Apoptosis(%)	15.7	
	ARVM	DLTZ(diltiazem:1microM/L)	Apoptosis(%)	9.4	
	ARVM	NE+DLTZ(diltiazem:1microM/L)	Apoptosis(%)	8.8	
9688607	p38 MAPK expression and activation in smooth muscle				
	Colonic muscle strips	Ach	p38	1	0min
	Colonic muscle strips	Ach	p38	3.75	1min
	Colonic muscle strips	Ach	p38	4.75	5min
	Colonic muscle strips	Ach	p38	7.6	10min
	Colonic muscle strips	Ach	p38	6.8	20min
	Colonic muscle strips	Ach	p38	3.6	30min
	colonic smooth muscle	none	ATF2	0	0min
	colonic smooth muscle	none	ATF2	2500	30min
	colonic smooth muscle	none	ATF2	7000	180min
	colonic smooth muscle	SB202190	ATF2	0	0min

	colonic smooth muscle	SB202190	ATF2	0	30min
	colonic smooth muscle	SB202190	ATF2	0	180min
8622669	MKK3 and MKK6 regulated gene expression is mediated by the p38 MAPK signal transduction pathway.				
	CHO cell+MKK3 TF	none	p38	positive	48hr
	CHO cell+MKK3 TF	none	ERK	1	48hr
	CHO cell+MKK3 TF	none	JNK	1	48hr
	CHO cell+MEK1 TF	none	p38	1	48hr
	CHO cell+MEK1 TF	none	ERK	positive	48hr
	CHO cell+MEK1 TF	none	JNK	1	48hr
	CHO cell	none	cJun	1	48hr
	CHO cell+MKK3 TF + p38 TF	none	cJun	6	48hr
	CHO cell+ERK TF	none	cJun	28	48hr
	CHO cell	none	cFos	1	48hr
	CHO cell+MKK3 TF + p38 TF	none	cFos	7	48hr
	CHO cell+ERK TF	none	cFos	5	48hr
	CHO cell	none	ATF2	1	48hr
	CHO cell+MKK3 TF + p38 TF	none	ATF2	5	48hr
	CHO cell+ERK TF	none	ATF2	1.5	48hr
	CHO cell	none	cJun	1	48hr
	CHO cell+MKK3 TF + p38 TF	none	cJun	1.5	48hr
	CHO cell+ERK TF	none	cJun	3	48hr
	CHO cell	none	ELK1	1	48hr
	CHO cell+MKK3 TF + p38 TF	none	ELK1	6.5	48hr
	CHO cell+ERK TF	none	ELK1	6.5	48hr
10330143 From here	Targeting of p38 MAPK to MEF2 transcriptional factors				
	COS	p38	MEF2A	20	15min

	COS	p38	MEF2A	30	30min
	COS	p38	MEF2A	60	60min
	COS	p38	MEF2A	100	120min
	COS	p38	MEF2C	10	15min
	COS	p38	MEF2C	30	30min
	COS	p38	MEF2C	60	60min
	COS	p38	MEF2C	100	120min
	COS	none	MEF2A	2.5	
	COS + CA MKK6/p38	none	MEF2A	53	
	COS	none	MEF2C	2	
	COS + CA MKK6/p38	none	MEF2C	32.5	
18390926	The extreme C-terminal region of phospholipase Cbeta1 determines subcellular localizaiton and function; the "b" splice variant mediates alpha1-ar responses in cardiomyocytes				
	NRVM+Lac Z	none	PLC	1.3	
	NRVM+G alpha q-WT	none	PLC	2.8	
	NRVM+G alpha q(Q209L)	none	PLC	5.8	
	NRVM+G alpha q(Q209L,D243A,N244A,E245A)	none	PLC	2.2	
9247274	c-AMP-dependent regulation of cardiac L-type Ca2+ channels requires membrane targeting of PKA and phosphorylation of channel subunits.				
	Cardiac Myocytes	isoproterenol	Relative increase in Ica	1.75	
	Cardiac Myocytes	isoproterenol +PKI	Relative increase in Ica	1.1	
	Cardiac Myocytes	isoproterenol + HT31	Relative increase in Ica	1.2	
	Cardiac Myocytes	isoproterenol + HT31P	Relative increase in Ica	1.7	
10347087 HOE642(Na+/H+ exhanger	Mechanical stretch activates the JAK/STAT pathway in rat cardiomyocytes.				
	Cardiomyocytes	Stretch	JAK1	0	0min
	Cardiomyocytes	Stretch	JAK1	1	2min

inhibitor)<br>BAPTA-<br>AM(intracellular<br>calcium chelator)<br>gadolinium<br>(stretch-activated<br>ion channel<br>inhibitor)<br>EGTA<br>(extracellular ca2+<br>chelator)<br>KN62(Ca2+/calm<br>odulin kinase2<br>inhibitor)<br>chelrythrin (PKC<br>inhibitor)<br>CV11974(AT1<br>blocker)<br>TAK044(endothel<br>iln-1-type A/B-<br>receptor blocker)<br>RX435(anti-<br>glycoprotein 130<br>antibody)	Cardiomyocytes	Stretch	JAK1	4	5min
	Cardiomyocytes	Stretch	JAK1	3	15min
	Cardiomyocytes	Stretch	JAK1	2	30min
	Cardiomyocytes	Stretch	JAK2	1	0min
	Cardiomyocytes	Stretch	JAK2	2.5	2min
	Cardiomyocytes	Stretch	JAK2	3	5min
	Cardiomyocytes	Stretch	JAK2	2.5	15min
	Cardiomyocytes	Stretch	JAK2	1	30min
	Cardiomyocytes	Stretch	STAT1	1	0min
	Cardiomyocytes	Stretch	STAT1	5	2min
	Cardiomyocytes	Stretch	STAT1	5.5	5min
	Cardiomyocytes	Stretch	STAT1	3.5	15min
	Cardiomyocytes	Stretch	STAT1	3.5	30min
	Cardiomyocytes	Stretch	STAT3	1	0min
	Cardiomyocytes	Stretch	STAT3	2	2min
	Cardiomyocytes	Stretch	STAT3	3.5	5min
	Cardiomyocytes	Stretch	STAT3	4	15min
	Cardiomyocytes	Stretch	STAT3	2	30min
	Cardiomyocytes	none	STAT1	1	30min Treat 6min 20% stre
	Cardiomyocytes	Stretch	STAT1	2.5	30min Treat 6min 20% stre
	Cardiomyocytes	AG490 + Stretch	STAT1	0.5	30min Treat 6min 20% stre
	Cardiomyocytes	none	STAT3	1	30min Treat 6min 20% stre
	Cardiomyocytes	Stretch	STAT3	2.5	30min Treat 6min 20% stre
	Cardiomyocytes	AG490 + Stretch	STAT3	1	30min Treat 6min 20% stre
	Cardiomyocytes	none	STAT3		30min Treat 6min 20% stre
	Cardiomyocytes	LIF	STAT3		30min Treat 6min 20% stre
	Cardiomyocytes	AG490 + LIF	STAT3		30min Treat 6min 20% stre
	Cardiomyocytes	none	STAT1	1	30min Treat 6min 20% stre
	Cardiomyocytes	LIF	STAT1	5	30min Treat 6min 20% stre
	Cardiomyocytes	AG490 + LIF	STAT1	4	30min Treat 6min 20% stre

	Mouse cardiomyocytes	none	STAT3	1	30min Treat 6min 20% stre
	Mouse cardiomyocytes	Stretch	STAT3	6	30min Treat 6min 20% stre
	Mouse cardiomyocytes	Stretch + anti-gp130 blocking Ab	STAT3	2	30min Treat 6min 20% stre
	Mouse cardiomyocytes	LIF	STAT3	7.5	30min Treat 6min 20% stre
	Mouse cardiomyocytes	LIF + anti-gp130 blocking Ab	STAT3	3	30min Treat 6min 20% stre
	Mouse cardiomyocytes	none	STAT1	1	
	Mouse cardiomyocytes	Stretch	STAT1	5	
	Mouse cardiomyocytes	Stretch + anti-gp130 blocking Ab	STAT1	4	
	Mouse cardiomyocytes	none	STAT3	1	
	Mouse cardiomyocytes	Stretch	STAT3	5	
	Mouse cardiomyocytes	Stretch + anti-gp130 blocking Ab	STAT3	2	
	Rat cardiomyocytes	Stretch	gp130LIFR	1	0min
	Rat cardiomyocytes	Stretch	gp130LIFR	6	2min
	Rat cardiomyocytes	Stretch	gp130LIFR	4	5min
	Rat cardiomyocytes	Stretch	gp130LIFR	4	15min
	Cardiomyocytes	Stretch + BAPTA-AM	STAT3	6	6min 20% stretch 60min tre
	Cardiomyocytes	Stretch + BAPTA-AM	STAT3	6	6min 20% stretch 60min tre
	Cardiomyocytes	Stretch + BAPTA-AM	STAT3	0.5	6min 20% stretch 60min tre
	Cardiomyocytes	none	STAT3	1	6min
	Cardiomyocytes	Stretch	STAT3	2.5	6min
	Cardiomyocytes	Stretch + chelerythrine	STAT3	0.7	6min 20% stretch 30min tre
	Cardiomyocytes	PMA	STAT3	5	6min 20% stretch 120min tre
	Cardiomyocytes	none	STAT1	1	6min
	Cardiomyocytes	chelerythrine	STAT1	1	6min 20% stretch 30min tre
	Cardiomyocytes	Stretch	STAT1	3	6min
	Cardiomyocytes	Stretch + chelerythrine	STAT1	2	6min 20% stretch 30min tre
	Cardiomyocytes	PMA	STAT1	4	6min 20% stretch 120min tre

9468195	Biphasic activation of the jak/stat pathway by angiotensin2 in rat cardiomyocytes				
	Cardiomyocytes	AngII	JAK2	0.067	0min
	Cardiomyocytes	AngII	JAK2	0.067	2min
	Cardiomyocytes	AngII	JAK2	0.067	5min
	Cardiomyocytes	AngII	JAK2	0.5	15min
	Cardiomyocytes	AngII	JAK2	1	30min
	Cardiomyocytes	LIF	JAK2	0.03	0min
	Cardiomyocytes	LIF	JAK2	0.6	2min
	Cardiomyocytes	LIF	JAK2	1	5min
	Cardiomyocytes	LIF	JAK2	0.475	15min
	Cardiomyocytes	LIF	JAK2	0.067	30min
	Cardiomyocytes	AngII	STAT1	0.15	0min
	Cardiomyocytes	AngII	STAT1	0.15	2min
	Cardiomyocytes	AngII	STAT1	0.05	5min
	Cardiomyocytes	AngII	STAT1	1	15min
	Cardiomyocytes	AngII	STAT1	0.95	30min
	Cardiomyocytes	LIF	STAT1	0	0min
	Cardiomyocytes	LIF	STAT1	0.2	2min
	Cardiomyocytes	LIF	STAT1	1	5min
	Cardiomyocytes	LIF	STAT1	1	15min
	Cardiomyocytes	LIF	STAT1	0.1	30min
	Cardiomyocytes	AngII	STAT2	0.1	0min
	Cardiomyocytes	AngII	STAT2	0.1	2min
	Cardiomyocytes	AngII	STAT2	0.3	5min
	Cardiomyocytes	AngII	STAT2	0.6	15min
	Cardiomyocytes	AngII	STAT2	1	30min
	Cardiomyocytes	LIF	STAT2	0	0min
	Cardiomyocytes	LIF	STAT2	0	2min
	Cardiomyocytes	LIF	STAT2	0	5min
	Cardiomyocytes	LIF	STAT2	0	15min

	Cardiomyocytes	LIF	STAT2	0	30min
	Cardiomyocytes	AngII	STAT3	0	0min
	Cardiomyocytes	AngII	STAT3	0	2min
	Cardiomyocytes	AngII	STAT3	0	5min
	Cardiomyocytes	AngII	STAT3	0	15min
	Cardiomyocytes	AngII	STAT3	0	30min
	Cardiomyocytes	LIF	STAT3	0	0min
	Cardiomyocytes	LIF	STAT3	0.9	2min
	Cardiomyocytes	LIF	STAT3	1	5min
	Cardiomyocytes	LIF	STAT3	0.7	15min
	Cardiomyocytes	LIF	STAT3	0.2	30min
	Cardiomyocytes	AngII	STAT1	0	0min
	Cardiomyocytes	AngII	STAT1	0	5min
	Cardiomyocytes	AngII	STAT1	1	15min
	Cardiomyocytes	AngII	STAT1	0	60min
	Cardiomyocytes	AngII	STAT1	0.9	120min
	Cardiomyocytes	AngII	STAT3	0	0min
	Cardiomyocytes	AngII	STAT3	1	5min
	Cardiomyocytes	AngII	STAT3	0	15min
	Cardiomyocytes	AngII	STAT3	0.5	30min
	Cardiomyocytes	AngII	STAT3	0.5	60min
	Cardiomyocytes	AngII	STAT3	2.5	120min
	Cardiomyocytes	Ang2 (10^-7mol/L)	Tyr-STAT1	0	0min
	Cardiomyocytes	Ang2 (10^-7mol/L)	Tyr-STAT1	0	5min
	Cardiomyocytes	Ang2 (10^-7mol/L)	Tyr-STAT1	1	15min
	Cardiomyocytes	Ang2 (10^-7mol/L)	Tyr-STAT1	0.9	30min
	Cardiomyocytes	Ang2 (10^-7mol/L)	Tyr-STAT1	0	60min
	Cardiomyocytes	Ang2 (10^-7mol/L)	Tyr-STAT1	0.85	120min
	Cardiomyocytes	Ang2 (10^-7mol/L)	Tyr-STAT3	0.1	0min
	Cardiomyocytes	Ang2 (10^-7mol/L)	Tyr-STAT3	0.15	5min

	Cardiomyocytes	Ang2 (10^-7mol/L)	Tyr-STAT3	0.05	15min
	Cardiomyocytes	Ang2 (10^-7mol/L)	Tyr-STAT3	0.1	30min
	Cardiomyocytes	Ang2 (10^-7mol/L)	Tyr-STAT3	0.13	60min
	Cardiomyocytes	Ang2 (10^-7mol/L)	Tyr-STAT3	1	120min
	Cardiomyocytes	none	STAT1	1	
	Cardiomyocytes	Ang2	STAT1	5	
	Cardiomyocytes	Ang2 + CV11974	STAT1	1.3	
	Cardiomyocytes	none	STAT1	1	
	Cardiomyocytes	Ang2	STAT1	6	
	Cardiomyocytes	Ang2 + CV11974	STAT1	5.7	30min
	Cardiomyocytes	Ang2 + CV11974	STAT1	6	60min
10559134	Characterization of IGF-1-induced activation of the JAK/STAT Pathway in Rat Cardiomyocytes.				
	Cardiomyocytes	IGF-1 (10^-8mol/L)	JAK2	1	0min
	Cardiomyocytes	IGF-1 (10^-8mol/L)	JAK2	1	5min
	Cardiomyocytes	IGF-1 (10^-8mol/L)	JAK2	1	15min
	Cardiomyocytes	IGF-1 (10^-8mol/L)	JAK2	1	30min
	Cardiomyocytes	LIF	JAK2	5	5min
	Cardiomyocytes	IGF-1 (10^-8mol/L)	Raf1	1	0min
	Cardiomyocytes	IGF-1 (10^-8mol/L)	Raf1	2.35	2min
	Cardiomyocytes	IGF-1 (10^-8mol/L)	Raf1	2.3	5min
	Cardiomyocytes	IGF-1 (10^-8mol/L)	Raf1	1.18	15min
	Cardiomyocytes	IGF-1 (10^-8mol/L)	Raf1	1	30min
	Cardiomyocytes	IGF-1 (10^-8mol/L)	ERK12	1	0min
	Cardiomyocytes	IGF-1 (10^-8mol/L)	ERK12	2	1min
	Cardiomyocytes	IGF-1 (10^-8mol/L)	ERK12	3	2min
	Cardiomyocytes	IGF-1 (10^-8mol/L)	ERK12	5	5min
	Cardiomyocytes	IGF-1 (10^-8mol/L)	ERK12	4	15min
	Cardiomyocytes	IGF-1 (10^-8mol/L)	ERK12	1.5	30min
	Cardiomyocytes	IGF-1 (10^-8mol/L)	ERK12	1.7	60min
	Cardiomyocytes	IGF-1 (10^-8mol/L)	STAT1	0	0min

	Cardiomyocytes	IGF-1 (10^-8mol/L)	STAT1	1	2min
	Cardiomyocytes	IGF-1 (10^-8mol/L)	STAT1	2.5	5min
	Cardiomyocytes	IGF-1 (10^-8mol/L)	STAT1	4	15min
	Cardiomyocytes	IGF-1 (10^-8mol/L)	STAT1	0	30min
	Cardiomyocytes	IGF-1 (10^-8mol/L)	STAT1	0	60min
	Cardiomyocytes	LIF	STAT1	1	0min
	Cardiomyocytes	LIF	STAT1	1	2min
	Cardiomyocytes	LIF	STAT1	2	5min
	Cardiomyocytes	LIF	STAT1	2.3	15min
	Cardiomyocytes	LIF	STAT1	1	30min
	Cardiomyocytes	IGF-1 (10^-8mol/L)	STAT3	0.2	0min
	Cardiomyocytes	IGF-1 (10^-8mol/L)	STAT3	1	2min
	Cardiomyocytes	IGF-1 (10^-8mol/L)	STAT3	1	5min
	Cardiomyocytes	IGF-1 (10^-8mol/L)	STAT3	1	15min
	Cardiomyocytes	IGF-1 (10^-8mol/L)	STAT3	1	30min
	Cardiomyocytes	IGF-1 (10^-8mol/L)	STAT3	2	60min
	Cardiomyocytes	IGF-1 (10^-8mol/L)	STAT3	2	90min
	Cardiomyocytes	IGF-1 (10^-8mol/L)	STAT3	2	120min
	Cardiomyocytes	LIF	STAT3	0	0min
	Cardiomyocytes	LIF	STAT3	1	2min
	Cardiomyocytes	LIF	STAT3	1.3	5min
	Cardiomyocytes	LIF	STAT3	0.5	15min
	Cardiomyocytes	LIF	STAT3	0.05	30min
	Cardiomyocytes	none	STAT3	0	5min
	Cardiomyocytes	LIF (1 U/ml)	STAT3	4	5min
	Cardiomyocytes	LIF (10 U/ml)	STAT3	8	5min
	Cardiomyocytes	LIF (100 U/ml)	STAT3	90	5min
	Cardiomyocytes	LIF (1000 U/ml)	STAT3	100	5min
	Cardiomyocytes	IGF-1 (10^-9mol/L)	STAT3	9	60min
	Cardiomyocytes	IGF-1 (10^-8mol/L)	STAT3	14	60min

	Cardiomyocytes	IGF-1 (10^-8mol/L)	STAT1	0	0min
	Cardiomyocytes	IGF-1 (10^-8mol/L)	STAT1	1	5min
	Cardiomyocytes	IGF-1 (10^-8mol/L)	STAT1	1	15min
	Cardiomyocytes	IGF-1 (10^-8mol/L)	STAT1	2	30min
	Cardiomyocytes	IGF-1 (10^-8mol/L)	STAT1	1	60min
	Cardiomyocytes	IGF-1 (10^-8mol/L)	STAT3	1	0min
	Cardiomyocytes	IGF-1 (10^-8mol/L)	STAT3	3.3	5min
	Cardiomyocytes	IGF-1 (10^-8mol/L)	STAT3	0.6	15min
	Cardiomyocytes	IGF-1 (10^-8mol/L)	STAT3	0.7	30min
	Cardiomyocytes	IGF-1 (10^-8mol/L)	STAT3	4	60min
	Cardiomyocytes + IGF-1 (10^-8mol/L)	none	STAT3	1	0min
	Cardiomyocytes + IGF-1 (10^-8mol/L)	none	STAT3	1.3	60min
	Cardiomyocytes + IGF-1 (10^-8mol/L)	TAK044	STAT3	1.5	60min
	Cardiomyocytes + IGF-1 (10^-8mol/L)	CV11974	STAT3	1.3	60min
	Murine Cardiomyocytes	none	STAT3	0	60min
	Murine Cardiomyocytes	IGF-1 (10^-8mol/L)	STAT3	1	60min
	Murine Cardiomyocytes	IGF-1 (10^-8mol/L) + RX435	STAT3	1	60min
	Murine Cardiomyocytes	none	STAT3	0	5min
	Murine Cardiomyocytes	LIF (1000U/mL)	STAT3	1	5min
	Murine Cardiomyocytes	LIF (1000U/mL) + RX435	STAT3	0.25	5min
	Cardiomyocytes	none	STAT3	0	30min preincu- in inhibitor
	Cardiomyocytes	IGF-1 (10^-8mol/L)	STAT3	1	30min preincu- in inhibitor
	Cardiomyocytes	IGF-1 (10^-8mol/L) +BAPTA-AM	STAT3	0.05	30min preincu- in inhibitor
	Cardiomyocytes	IGF-1 (10^-8mol/L) + EDTA	STAT3	0.96	30min preincu- in inhibitor

	Cardiomyocytes	IGF-1 (10^-8mol/L) + KN62	STAT3	1.04	30min preincu- in inhibitor
	Cardiomyocytes	IGF-1 (10^-8mol/L) + PD98059	STAT3	0.98	30min preincu- in inhibitor
	Cardiomyocytes	IGF-1 (10^-8mol/L) + wortmannin	STAT3	0.84	30min preincu- in inhibitor
	Cardiomyocytes	IGF-1 (10^-8mol/L) + Chelerythrine	STAT3	0.02	30min preincu- in inhibitor
	Cardiomyocytes	none	STAT1	1	30min preincu- in inhibitor
	Cardiomyocytes	IGF-1 (10^-8mol/L)	STAT1	6	30min preincu- in inhibitor
	Cardiomyocytes	IGF-1 (10^-8mol/L) +BAPTA-AM	STAT1	6	30min preincu- in inhibitor
	Cardiomyocytes	IGF-1 (10^-8mol/L) + EDTA	STAT1	6	30min preincu- in inhibitor
	Cardiomyocytes	IGF-1 (10^-8mol/L) + KN62	STAT1	6	30min preincu- in inhibitor
	Cardiomyocytes	none	STAT1	1	30min preincu- in inhibitor
	Cardiomyocytes	IGF-1 (10^-8mol/L)	STAT1	2.5	30min preincu- in inhibitor
	Cardiomyocytes	IGF-1 (10^-8mol/L) + wortmannin	STAT1	2.6	30min preincu- in inhibitor
	Cardiomyocytes	IGF-1 (10^-8mol/L) + PD98059	STAT1	2.5	30min preincu- in inhibitor
	Cardiomyocytes	none	STAT1	1	30min preincu- in inhibitor
	Cardiomyocytes	IGF-1 (10^-8mol/L)	STAT1	3	30min preincu- in inhibitor
	Cardiomyocytes	IGF-1 (10^-8mol/L) + Chelerythrine	STAT1	2.8	30min preincu- in inhibitor
16172266 ERK 1/2 AND STAT3 IS OR LOGIC	Role of the Protein Kinase C-–Raf-1–MEK-1/2–p44/42 MAPK Signaling Cascade in the Activation of Signal Transducers and Activators of Transcription 1 and 3 and Induction of Cyclooxygenase-2 After Ischemic Preconditioning				
	myocardium	none	p-Ser(338)-Raf1 / total Raf1	1	
	myocardium	ischemic PC	p-Ser(338)-Raf1 / total Raf1	1.87	
	myocardium	ischemic PC + Chelerythrine		1.19	

	myocardium	none	p-Ser-MEK12 / total MEK12	1	
	myocardium	ischemic PC	p-Ser-MEK12 / total MEK12	2.44	
	myocardium	ischemic PC + Chelerythrine		1.63	
	myocardium	none	p-Tyr(204)-ERK12 / total ERK12	1	
	myocardium	ischemic PC	p-Tyr(204)-ERK12 / total ERK12	4.05	
	myocardium	ischemic PC + Chelerythrine		1.53	
	myocardium	none	p-Ser(727)-STAT1/total STAT1	1	
	myocardium	ischemic PC	p-Ser(727)-STAT1/total STAT1	1.32	
	myocardium + PKC epsilon (KO)	ischemic PC	p-Ser(727)-STAT3	0.413436693	
	myocardium	none	p-Tyr(705)-STAT3	1	
	myocardium	ischemic PC	p-Tyr(705)-STAT3	0.988235294	
	myocardium + PKC epsilon (KO)	none	p-Tyr(705)-STAT3	0.72	
	myocardium + PKC epsilon (KO)	ischemic PC	p-Tyr(705)-STAT3	0.981912145	
	myocardium	none	STAT1/3-DNA binding activity	1	
	myocardium	ischemic PC	STAT1/3-DNA binding activity	5.5	
	myocardium + PKC epsilon (KO)	none	STAT1/3-DNA binding activity	1	
	myocardium + PKC epsilon	ischemic PC	STAT1/3-DNA	2.4	

	(KO)		binding activity		
	myocardium	none	COX-2	1	
	myocardium	ischemic PC	COX-2	4.5	
	myocardium + PKC epsilon (KO)	none	COX-2	1	
	myocardium + PKC epsilon (KO)	ischemic PC	COX-2	2.25	
12494267	Angiotensin II effects on STAT3 phosphorylation in cardiomyocytes: evidence for Erk-dependent Tyr705 dephosphorylation				
	Neonatal rat cardiomyocytes	AngII	p-Tyr705-STAT3	1	0min
	Neonatal rat cardiomyocytes	AngII	p-Tyr705-STAT3	0.83	2min
	Neonatal rat cardiomyocytes	AngII	p-Tyr705-STAT3	0.95	5min
	Neonatal rat cardiomyocytes	AngII	p-Tyr705-STAT3	0.625	15min
	Neonatal rat cardiomyocytes	AngII	p-Tyr705-STAT3	0.48	30min
	Neonatal rat cardiomyocytes	AngII	p-Tyr705-STAT3	0.8	60min
	Neonatal rat cardiomyocytes	AngII	p-Tyr705-STAT3	1.55	90min
	Neonatal rat cardiomyocytes	AngII	p-Tyr705-STAT3	1.18	120min
	Neonatal rat cardiomyocytes	AngII	p-Ser727-STAT3	1	0min
	Neonatal rat cardiomyocytes	AngII	p-Ser727-STAT3	1.44	2min
	Neonatal rat cardiomyocytes	AngII	p-Ser727-STAT3	1.75	5min
	Neonatal rat cardiomyocytes	AngII	p-Ser727-STAT3	1.17	15min
	Neonatal rat cardiomyocytes	AngII	p-Ser727-STAT3	1.6	30min
	Neonatal rat cardiomyocytes	AngII	p-Ser727-STAT3	1.375	60min
	Neonatal rat cardiomyocytes	AngII	p-Ser727-STAT3	1.3	90min
	Neonatal rat cardiomyocytes	AngII	p-Ser727-STAT3	1.27	120min
	Neonatal rat cardiomyocytes	none	p-Tyr705-	1	30min pret and 30min Ang2

			STAT3		
	Neonatal rat cardiomyocytes	AngII	p-Tyr705-STAT3	0.5	30min pret and 30min Ang2
	Neonatal rat cardiomyocytes + cycloheximide	AngII	ERK2	100	5min
	Neonatal rat cardiomyocytes + cycloheximide	AngII	ERK2	50	30min
	Neonatal rat cardiomyocytes + cycloheximide	AngII	ERK2	37.5	60min
	Neonatal rat cardiomyocytes + cycloheximide	AngII	ERK2	27.5	120min
	Neonatal rat cardiomyocytes + 100nM Ang2	none	p-Tyr705-STAT3	0.7	
	Neonatal rat cardiomyocytes + 100nM Ang2	PD98059 15uM	p-Tyr705-STAT3	1.1	
	Neonatal rat cardiomyocytes	none	p-Tyr705-STAT3	1	
	Neonatal rat cardiomyocytes	vanadate	p-Tyr705-STAT3	0.8	
	Neonatal rat cardiomyocytes + 100nM Ang2	none	p-Tyr705-STAT3	0.5	
	Neonatal rat cardiomyocytes + 100nM Ang2	vanadate	p-Tyr705-STAT3	0.9	
10856262 Here 2/19	Cardiotrophin-1 Increases Angiotensinogen mRNA in Rat Cardiac Myocytes Through STAT3 An Autocrine Loop for Hypertrophy				
	NRVM	CT1	STAT1	0	0min
	NRVM	CT1	STAT1	0.02	2min
	NRVM	CT1	STAT1	1	5min
	NRVM	CT1	STAT1	5	10min
	NRVM	CT1	STAT1	6	15min
	NRVM	CT1	STAT1	2.5	30min
	NRVM	CT1	STAT1	0	60min
	NRVM	CT1	STAT1	0	120min
	NRVM	CT1	STAT3	0	0min

	NRVM	CT1	STAT3	1.5	2min
	NRVM	CT1	STAT3	3	5min
	NRVM	CT1	STAT3	4.7	10min
	NRVM	CT1	STAT3	2.7	15min
	NRVM	CT1	STAT3	2.5	30min
	NRVM	CT1	STAT3	0.5	60min
	NRVM	CT1	STAT3	0.02	120min
	Cardiomyocytes	CT1	3H-Leucine incorp	16	losartan30min CT-1 treat
	Cardiomyocytes + Losartan	CT1	3H-Leucine incorp	6	losartan30min CT-1 treat
	Cardiomyocytes	CT1	Protein content	25	losartan30min CT-1 treat
	Cardiomyocytes + Losartan	CT1	Protein content	11.25	losartan30min CT-1 treat
	Cardiomyocytes	CT1	Protein / DNA	30	losartan30min CT-1 treat
	Cardiomyocytes + Losartan	CT1	Protein / DNA	15.7	losartan30min CT-1 treat
20518848	Rac1 activation induces tumour necrosis factor- expression and cardiac dysfunction in endotoxemia				
	Neonetal Cardiomyocytes	LPS	Rac1	0.15	0min
	Neonetal Cardiomyocytes	LPS	Rac1	0.225	5min
	Neonetal Cardiomyocytes	LPS	Rac1	0.31	15min
	Neonetal Cardiomyocytes	LPS	Rac1	0.25	30min
	Neonetal Cardiomyocytes	LPS	Rac1	0.23	60min
	Adult male Racf/f mice	none	Rac1	0.47	30min
	Adult male Racf/f mice	LPS	Rac1	0.95	30min
	Neonetal Cardiomyocytes	none	PIP3 production	2.1	30min
	Neonetal Cardiomyocytes	LPS	PIP3 production	3.3	30min
	Cardiomyocytes	LPS	Rac1	0.73	30min
	Cardiomyocytes	LPS + LY294002	Rac1	0.43	30min
	Neonetal Cardiomyocytes	none	ERK12	1.3	0min
	Neonetal Cardiomyocytes	LPS	ERK12	2.2	15min
	Neonetal Cardiomyocytes	LPS	ERK12	2.55	30min
	Neonetal Cardiomyocytes	LPS	ERK12	1.55	1h

	Neonetal Cardiomyocytes	LPS	ERK12	1.27	2h
	Cardiomyocytes	none	ERK12	0.67	30min
	Cardiomyocytes	LPS	ERK12	0.97	30min
	Cardiomyocytes	LPS + LY294002	ERK12	0.7	30min
	Cardiomyocytes + Ad-GFP	none	ERK12	0.6	30min
	Cardiomyocytes + Ad-GFP	LPS	ERK12	0.94	30min
	Cardiomyocytes + Ad-Rac1N17	LPS	ERK12	0.73	30min
9305638 Here 3/28	MEK kinases are regulated by EGF and selectively interact with Rac/Cdc42				
	COS cells	none	MEKK1	1	
	COS cells	EGF	MEKK1	2.1	
	COS cells	Wortmannin + EGF	MEKK1	2.1	
	COS cells	none	MEKK2	1	
	COS cells	EGF	MEKK2	3.9	
	COS cells	Wortmannin + EGF	MEKK2	4.1	
	COS cells	none	JNK	1	
	COS cells + Delta MEKK1 TF	none	JNK	5	
	COS cells + Delta MEKK2 TF	none	JNK	5	
	COS cells + Delta MEKK3 TF	none	JNK	5	
	COS cells + Delta MEKK4 TF	none	JNK	5	
	COS cells	none	ERK2	1	
	COS cells + Delta MEKK1 TF	none	ERK2	3	
	COS cells + Delta MEKK2 TF	none	ERK2	2.5	
	COS cells + Delta MEKK3 TF	none	ERK2	2.65	
	COS cells + Delta MEKK4	none	ERK2	1.6	

	TF				
	COS cells	none	JNK	1	
	COS cells	EGF	JNK	4.3	
	COS cells	Wortmannin + EGF	JNK	4.7	
	COS cells	none	ERK2	1	
	COS cells	EGF	ERK2	5.25	
	COS cells	Wortmannin + EGF	ERK2	5.35	
	COS cells + empty vector TF	none	ERK	1	
	COS cells + empty vector TF	EGF	ERK	4.9	
	COS cells + MEKK1KM expression	EGF	ERK	1.6	
	COS cells + MEKK2KM expression	EGF	ERK	4.5	
	COS cells + MEKK3KM expression	EGF	ERK	4.25	
	COS cells + MEKK4KM expression	EGF	ERK	4.75	
	COS cells + empty vector TF	none	JNK	1	
	COS cells + empty vector TF	Cdc42	JNK	11	
	COS cells + MEKK1KM expression	Cdc42	JNK	3.6	
	COS cells + MEKK2KM expression	Cdc42	JNK	11.5	
	COS cells + MEKK3KM expression	Cdc42	JNK	9.3	
	COS cells + MEKK4KM expression	Cdc42	JNK	5.5	
	COS cells + empty vector TF	none	JNK	1	
	COS cells + empty vector TF	Rac1	JNK	6.7	
	COS cells + MEKK1KM expression	Rac1	JNK	3	
	COS cells + MEKK2KM expression	Rac1	JNK	7.25	

	COS cells + MEKK3KM expression	Rac1	JNK	6.5	
	COS cells + MEKK4KM expression	Rac1	JNK	3	
	MEKK2,3 do not bind to the Rho family of GTP-binding proteins.				
	MEKK1 bound to Cdc42 in a GTP dependent manner and di not bind to Rho.				
	MEKK4 bound to Cdc42 and Rho in both the GTP and GDP states				
	COS cells + MBP + Cdc42 1ug	none	Rel lev of 32P-GTP labelled	1	
	COS cells + MBP - MEKK1 + Cdc42 1ug	none	Rel lev of 32P-GTP labelled	7.75	
	COS cells + MBP - MEKK3 + Cdc42 1ug	none	Rel lev of 32P-GTP labelled	1.25	
	COS cells + MBP - MEKK4 + Cdc42 1ug	none	Rel lev of 32P-GTP labelled	9.6	
	COS cells + MBP + Rac 1ug	none	Rel lev of 32P-GTP labelled	1	
	COS cells + MBP - MEKK1 + Rac 1ug	none	Rel lev of 32P-GTP labelled	6.3	
	COS cells + MBP - MEKK3 + Rac 1ug	none	Rel lev of 32P-GTP labelled	1.7	
	COS cells + MBP - MEKK4 + Rac 1ug	none	Rel lev of 32P-GTP labelled	5.27	
16934435	GSK-3β regulation in skeletal muscles by adrenaline and insulin: Evidence that PKA and PKB regulate different pools of GSK-3				
	Male wistar rats Soleus muscles	Insulin (10mU/ml)	pSer-GSK3beta : %of Insulin	100	30min
	Male wistar rats Soleus muscles	Insulin (10mU/ml)+ db-cAMP (5mM)	pSer-GSK3beta : %of Insulin	125	30min
	Male wistar rats Soleus muscles	none	pSer-GSK3beta : %of Insulin	8	30min

	Male wistar rats Soleus muscles	db-cAMP (5mM)	pSer-GSK3beta : %of Insulin	25	30min
	Male wistar rats Soleus muscles	Insulin	pSer-GSK3beta : %of Insulin	100	60min Epac 30min hormone
	Male wistar rats Soleus muscles	Insulin + EPAC activator (1mM)	pSer-GSK3beta : %of Insulin	104	60min Epac 30min hormone
	Male wistar rats Soleus muscles	none	pSer-GSK3beta : %of Insulin	8	60min Epac 30min hormone
	Male wistar rats Soleus muscles	EPAC activator (1mM)	pSer-GSK3beta : %of Insulin	12.5	60min Epac 30min hormone
	Male wistar rats Soleus muscles	Insulin	pSer-GSK3beta : %of Insulin	100	60min N6 30min Insulin
	Male wistar rats Soleus muscles	Insulin + N6-Benzoyl-cAMP (2mM)	pSer-GSK3beta : %of Insulin	138.5	60min N6 30min Insulin
	Male wistar rats Soleus muscles	none	pSer-GSK3beta : %of Insulin	5	60min N6 30min Insulin
	Male wistar rats Soleus muscles	N6-Benzoyl-cAMP (2mM)	pSer-GSK3beta : %of Insulin	25	60min N6 30min Insulin
	Male wistar rats Soleus muscles	none	pSer-GSK3beta : %of Insulin	2	60min H89 30min Hormone
	Male wistar rats Soleus muscles	H89 (50uM)	pSer-GSK3beta : %of Insulin	2	60min H89 30min Hormone
	Male wistar rats Soleus muscles	Adrenaline (10^-6M)	pSer-GSK3beta : %of	12.5	60min H89 30min Hormone

			Insulin		
	Male wistar rats Soleus muscles	Insulin (10mU/ml) + Adrenaline (10^-6M) + Wort (1uM)	pSer-GSK3beta : %of Insulin	12.5	10min Wort 30min Hormone
	Male wistar rats Soleus muscles	none	Glycogen synthase 3-site phosphorylation (%of control	100	30min
	Male wistar rats Soleus muscles	Adrenaline (10^-6M)	Glycogen synthase 3-site phosphorylation (%of control)	125	30min
	Male wistar rats Soleus muscles	Insulin (10mU/ml)		56	30min
	Male wistar rats Soleus muscles	Insulin (10mU/ml) + Adrenaline (10^-6M)		104	30min
1324914	Differential Regulation of Glycogen Synthase Kinase-3beta by Protein Kinase C Isotypes				
	Rabbit skeletal muscle	none	p-GSK3alpha/GSK3alpha	0.35	30min incubation with PKCa
	Rabbit skeletal muscle	PKC - alpha	p-GSK3alpha/GSK3alpha	0.35	30min incubation with PKCa
	Rabbit skeletal muscle	none	p-GSK3beta/GSK3beta	0.16	30min incubation with PKCa
	Rabbit skeletal muscle	PKC - alpha	p-GSK3beta/GSK3beta	1.75	30min incubation with PKCa
	Rabbit skeletal muscle	GSK3beta	p-c-Jun	1	PKC 25min c-Jun 2min
	Rabbit skeletal muscle	GSK3beta	p-c-Jun	2.5	PKC 25min c-Jun 5min
	Rabbit skeletal muscle	GSK3beta	p-c-Jun	5	PKC 25min c-Jun 10min
	Rabbit skeletal muscle	GSK3beta + PKC alpha 5units/ml	p-c-Jun	0.5	PKC 25min c-Jun 2min
	Rabbit skeletal muscle	GSK3beta + PKC alpha 5units/ml	p-c-Jun	1.25	PKC 25min c-Jun 5min

	Rabbit skeletal muscle	GSK3beta + PKC alpha 5units/ml	p-c-Jun	2.5	PKC 25min c-Jun 10min
11179396	G protein-mediated inhibitory effect of a nitric oxide donor on the L-type Ca2+ current in rat ventricular myocytes.				
	RVM	none	Zero-current Ca2+ level (nA)	1.13	0min
	RVM	none	Zero-current Ca2+ level (nA)	1	2.5min
	RVM	ISO 10nM	Zero-current Ca2+ level (nA)	1	4min
	RVM	ISO 10nM	Zero-current Ca2+ level (nA)	0.99	5min
	RVM	ISO 10nM	Zero-current Ca2+ level (nA)	2.1	7.5min
	RVM	ISO 10nM + DEANO 100uM	Zero-current Ca2+ level (nA)	2.2	10min
	RVM	ISO 10nM + DEANO 100uM	Zero-current Ca2+ level (nA)	2.2	12.5min
	RVM	ISO 10nM + DEANO 100uM	Zero-current Ca2+ level (nA)	1.9	15min
	COS1 cells + tag-ERK1(2ug)., MEK1(1ug). c-Raf TF(1ug)	CA PKC delta 1ug	Mobility shift of tag-ERK1	46	24hCulture
	COS1 cells + tag-ERK1(2ug)., MEK1(1ug). c-Raf TF(1ug)	CA PKC delta 3ug	Mobility shift of tag-ERK1	34	24hCulture
	COS1 cells + tag-ERK1(2ug)., MEK1(1ug). c-Raf TF(1ug)	none	Mobility shift of tag-ERK1	8	24hCulture
	COS1 cells + tag-ERK1(2ug)., MEK1(1ug). c-Raf TF(1ug)	CA PKC epsilon 0.1ug	Mobility shift of tag-ERK1	8	24hCulture
	COS1 cells + tag-ERK1(2ug)., MEK1(1ug). c-Raf TF(1ug)	CA PKC epsilon 0.3ug	Mobility shift of tag-ERK1	6	24hCulture

	COS1 cells + tag-ERK1(2ug)., MEK1(1ug). c-Raf TF(1ug)	CA PKC epsilon 1ug	Mobility shift of tag-ERK1	7	24hCulture
	COS1 cells + tag-ERK1(2ug)., MEK1(1ug). c-Raf TF(1ug)	CA PKC epsilon 3ug	Mobility shift of tag-ERK1	4	24hCulture
	COS1 cells + tag- MEK1 TF(5ug)	none	Relative MEK activity	0.15	24hCulture
	COS1 cells + tag- MEK1 TF(5ug)	CA PKC delta (1ug)	Relative MEK activity	0.17	24hCulture
	COS1 cells + tag- MEK1 TF(5ug)	c-Raf(5ug)	Relative MEK activity	1	24hCulture
	COS1 cells + tag- MEK1 TF(5ug)	CA PKC delta (1ug) + c-Raf(5ug)	Relative MEK activity	5.4	24hCulture
	COS1 cells + tag- MEK1 TF(5ug)	CA PKC delta (1ug) + c-Raf(5ug) + dn-Ras(5ug)	Relative MEK activity	5	24hCulture
	COS1 cells + tag-ERK1(2ug), MEK1(1ug)	none	ERK activity	1	24hCulture
	COS1 cells + tag-ERK1(2ug), MEK1(1ug)	PKC delta (1ug)	ERK activity	2	24hCulture
	COS1 cells + tag-ERK1(2ug), MEK1(1ug)	CA PKC delta (1ug)	ERK activity	3.5	24hCulture
	COS1 cells + tag-ERK1(2ug), MEK1(1ug)	CA PKC delta (1ug) + c-Raf (1ug)	ERK activity	5	24hCulture
	COS1 cells + tag-ERK1(2ug), MEK1(1ug)	CA PKC delta (1ug) + dn-Ras (2ug)	ERK activity	3	24hCulture

Supplementray Data Set S2. Experimental evidence of links in the cardiac signaling network.

Target of regulation (Abbreviation)	Full name	Related links	Explanation	References (PMID)
BAR	ß-adrenergic receptor	ISO → BAR	Isoproterenol (ISO) activates betaAR	ISBN-10: 0071764011<br>ISBN-13: 978-0071764018
BARp	Phosphorylated ß-adrenergic receptor	PKA → (BAR → BARp)	1) PKA-mediated phosphorylation of the beta1-adrenergic receptor promotes Gs/Gi switching (Figure 4b)<br><br>2) PKA-mediated phosphorylation of beta2-AR decreases the affinity of the receptor for Gs and increases its affinity for Gi	1) 15381255<br>2) 9363896, 12063255
BARd	Desensitized ß-adrenergic receptor	bARKnPI3K → (BAR → BARd)<br>bARKnPI3K → (BARp → BARd)	1) betaARK preferentially phosphorylates the agonist-coupled form of betaAR<br><br>2) betaARK phosphorylates and thereby inactivates agonist-occupied betaAR	1) 2165947<br>2) 9349395
bARK	ß-adrenergic receptor kinase	Gbg → bARK	1) Gbg mediates the membrane targeting of the bARK in response to receptor activation<br><br>2) Gbg recruit elevated levels of cytosolic GRK2(bARK1) to agonist-stimulated BARs in HF, leading to the chronic BAR desensitization, downregulation and pathological signaling that are hallmarks of HF	1) 8463335, 1325672<br>2) 20576935
Gs	stimulatory G protein	BAR → Gs	PKA-mediated phosphorylation of beta2-AR decreases the affinity of the receptor for Gs and increases its affinity for Gi	9363896, 12063255
Gi	inhibitory G protein	BARp → Gi	PKA-mediated phosphorylation of beta2-AR decreases the affinity of the receptor for Gs and increases its affinity for Gi	9363896, 12063255

AC	Adenylyl cyclase	Gs → AC<br>Gi --| AC	AC is regulated by G proteins (Gs stimulating activity and Gi inhibiting it)	ISBN: 0805366245
cAMP	Cyclic adenosine monophosphate	AC → cAMP<br>PDE34 --| cAMP	activation of Gs-coupled receptors leads to an increase in intracellular cAMP and PKA activation	12381255, 20530128
PKA	Protein kinase A	cAMP → PKA	cAMP activates PKA	20530128
epac	Exchange factor directly activated by cAMP	cAMP → epac	Epac1 is a guanine nucleotide exchange factor for Rap1 that is activated by direct binding of cAMP.	15550931
PDE34	Phosphodiesterase type 3 and type 4	PKA → PDE34<br>PKC → PDE34	1) PDE4 activity increased two- to threefold in a PKA-dependent manner<br><br>2) In addition to PKA phosphorylation, human PDE3A enzyme is phosphorylated by PKC	1) 11606735<br>2) 16153182
CREB	cAMP response element-binding protein	PKA → CREB<br>MSK1 → CREB	1) Phenylephrine induces activation of CREB in adult rat cardiac myocytes through MSK1 and PKA signaling pathways (Figure 3A, 3B)<br><br>2) ET and PE did not affect the transcriptional activation of CREB	1) 15522277<br>2) 11799083
αAR	α-adrenergic receptor	PE → αAR	PE activates αAR	ISBN-10 : 0071764011<br>ISBN-13 : 978-0071764018
Gq	Gq alpha subunit	αAR→Gq<br>RGS4 --| Gq	The inhibitory effect of ANP on ET-1–stimulated IP3 production was abolished in cells overexpressing RGS4DN (Figure 2A)	18443239
Gbg	Beta and gamma subunit of G protein	Gi → Gbg<br>Gq → Gbg	Gbg is generated simultaneously with activation of Gi or Gq	10819326
PLC	Phospholipase C	Gq → PLC	Gq stimulates the membrane-bound phospholipase C beta, which then cleaves PIP2 into two second messengers, IP3 and diacylglycerol (DAG)	21873996

IP3	Inositol triphosphate	PLC → IP3	Gq stimulates the membrane-bound phospholipase C beta, which then cleaves PIP2 into two second messengers, IP3 and diacylglycerol (DAG)	21873996
DAG	Diacylglycerol	PLC → DAG	Gq stimulates the membrane-bound phospholipase C beta, which then cleaves PIP2 into two second messengers, IP3 and diacylglycerol (DAG)	21873996
PKC	Protein kinase C	Ca → PKC<br>DAG → PKC<br>CaN→PKC	Inhibition of calcineurin with cyclosporine blocks PKCalpha and PKCtheta activation during load induced hypertrophy (Figure 8E)	10788473
TAK1	Transforming growth factor beta-activated kinase 1	PKC →TAK1	PKC acts by activating TAK1, which leads to ATF-2 activation	16125722
Ca	Calcium	PKA →Ca<br>IP3 → Ca<br>PI3K --| Ca	1) PKA induces calcium influx from extracellular space while IP3 induces SR calcium release<br><br>2) PI3K offsets cAMP-mediated positive inotropic effect via inhibiting calcium influx in cardiomyocytes	15539636
CaM	Calmodulin	Ca →CaM	Calmodulin (CaM) is a multifunctional intermediate messenger protein that transduces calcium signals by binding calcium ions and then modifying its interaction with various target proteins	6313166, 10884684
CaMK	Calcium/calmodulin-dependent protein kinase II	CaM → CaMK<br>epac → CaMK<br>PKC → CaMK	Epac activation increased CaMK Thr286 phosphorylation and enhanced phosphorylation at CaMK phosphorylation sites on the ryanodine receptor (RyR2) and phospholamban in a PKC-dependent manner (Figure 3B)	18957419
CaN	Calcineurin	CaM → CaN	Binding of Ca-saturated CaM to CaN activates the catalytic activity of CaN	16009337

HDAC	Histone deacetylase	PKA → HDAC<br>CaMK --| HDAC<br>PKC --| HDAC	1) CaMK signaling detach HDACs from MEF2 (Figure 4C)<br><br>2) PKA phosphorylates HDAC5 and prevents its nuclear export, leading to the inhibition of gene transcription and cardiomyocyte hypertrophy (Figure 3B)<br><br>3) PKA inhibits nuclear export of HDAC regardless of the status of phosphorylation by CaMK or PKD<br><br>4) The general serine/threonine protein kinase inhibitor staurosporine and the PKC inhibitor Bis I were effective in blocking the PE-dependent export of HDAC5 (Fig. 3C and D). In contrast, inhibitors of CaMK (KN93), MEK1 (U1026), Rho kinase (Y-27632), diacylglycerol kinase (diacylglycerol kinase inhibitor II), phosphatidylinositol 3-kinase (wortmannin), S6 kinase (rapamycin), glycogen synthase kinase (SB216763), or an inhibitor of protein kinase G, myosin light-chain kinase, and protein kinase A (HA1077) did not significantly affect PE-induced nuclear export of HDAC5.<br><br>5) PE does not activate PKA	1) 10737771<br>2),3) 20716686<br>4),5) 15367659

NFATnuc	Nuclear factor of activated T-cells (nucleus)	CaN → NFATnuc<br>JNK --| NFATnuc<br>p38 --| NFATnuc<br>GSK3B --| NFATnuc	1) endogenous p38 signaling normally attenuates the efficiency of basal NFATc1 nuclear occupancy (Figure 5c)<br><br>2) Inhibition of p38 signaling in cardiac myocytes enhances NFAT transcriptional activity<br><br>3) MKK7/JNK pathway negatively regulates NFATc3 activation (Figure 5B)<br><br>4) Coinfection of AdNFATc3 and AdCnA with wild-type AdMKK7, AdJNK1 or AdJNK2 significantly reduced the calcineurin-induced NFATc3 mobility shift, indicating partial blockade of nuclear translocation (Figure 5C).<br><br>5) GSK3beta phosphorylates conserved serines necessary for nuclear export, promotes nuclear exit.<br><br>6) MEK1 does not affect NFAT nuclear localization.<br><br>7) In the presence of Ang2 or PE, NFAT is up-regulated. This up-regulation was completely abolished in the presence of CsA or FK506, supporting the conclusion that AngII and PE activate NFAT through a calcineurin-dependent signal transduction pathway (Figure 4H).	1),2) 12750397<br>3),4) 14517246<br>5) 9072970<br>6) 15657416<br>7) 9568714

NFATact	Activated NFAT	cFos → NFATact<br>cJun → NFATact<br>GSK3B --| NFATact	Here, biological meaning of 'NFATact' is the transcriptional activity of NFAT.<br><br>1) MEK1-ERK1/2 signaling augments NFAT transcriptional activity independent of calcineurin, independent of changes in NFAT nuclear localization, and independent of alterations in NFAT transactivation potential. In contrast, MEK1-ERK1/2 signaling enhances NFAT-dependent gene expression through an indirect mechanism involving induction of cardiac AP-1 activity, which functions as a necessary NFAT interacting partner.<br><br>2) MEK1-ERK1/2 synergy with NFAT depends, in part, on AP-1 (Figure 5A).<br><br>3) MEK1-ERK1/2 directly regulates NFAT DNA binding activity.<br><br>4) MEK1-ERK1/2-induced synergy was blocked by the AP-1 inhibitory mutant protein TAM67. In cardiac myocytes, MEK1-ERK1/2 signaling significantly enhanced AP-1 transcriptional activity, which was blocked with TAM67.<br><br>5) GSK3B inhibits the DNA binding activity of NFATc	1), 2), 3), 4) 15657416<br>5) 11063740
EGFR	Epidermal growth factor receptor	EGF → EGFR	EGF induces EGFR dimerization and activation	14732694
Src	Proto-oncogene tyrosine-protein kinase Src	Gbg → Src<br>CaN → Src<br>FAK → Src	1) Calcineurin regulates the ISO-induced activation of ERKs possibly through the Src/Shc/Raf-1 kinase pathway (Fig 7).<br><br>2) FAK overexpression enhances ras-dependent integrin signaling to ERK2/MAPK through interactions with and activation of c-Src<br><br>3) LPA stimulation or expression of Gbg subunits resulted in c-	1) 11435346<br>2) 9148935<br>3) 8702633

			Src activation, as assessed by increased c-Src autophosphorylation.	
Shc	SH2 domain protein C	Src → Shc<br>EGFR → Shc	1) Phosphorylation of Tyr1148 in EGFR is required for Shc binding.<br><br>2) Ang2 causes tyrosine phosphorylation of Shc and its association with Grb2 and mSos-1.<br><br>3) Overexpression of wild-type or constitutively active mutant c-Src, but not kinase inactive mutant c-Src, lead to increased tyrosine kinase activity in Shc immunoprecipitates.	1) 16687399<br>2) 8631299<br>3) 8702633
Grb2act	Activated growth factor receptor-bound protein 2	EGFR → Grb2act<br>Shc → Grb2act	1) In beta-adrenergic cardiac hypertrophy, the Akt-GSK3b pathway, rather than the mitogen-activated protein kinase pathway, plays an essential role in transcription of atrial natriuretic factor (ANF).<br><br>2) Grb2(SH2 domain) associates with activated EGFR either directly or through tyrosyl-phosphorylatec Shc.	1) 11382772<br>2) 16687399, 11057895
Grb2Sos	growth factor receptor-bound protein 2(Grb2)- Son of Sevenless(Sos) complex	Grb2act → Grb2Sos<br>Shc → Grb2Sos<br>ERK12 --| Grb2Sos	1) Grb2(SH3 domian) associates with the cytoplasmic guanine nucleotide exchange factor SOS.<br><br>2) Inhibitory feedback phosphorylation of SOS by ERK provides a mechanism for the inhibition of Ras signaling.<br><br>3) SHP2 that binds to Gab1 was reported to be a positive regulator of the MAPK pathway.<br><br>4) Ang2 stimulates association of mSos-1 with Shc and translocates mSos-1 to the membrane fraction.<br><br>5) SHP2 contributes to the Ras activation in the same degree as EGF (Figure 5C)	1) 9865697, 7781603<br>2) 7829473, 7592690<br>3) 16687399<br>4) 8631299<br>5) 11134009

Grb2&Gab1	growth factor receptor-bound protein 2(Grb2)-Grb2-associated binding protein 1(Gab1) complex	Grb2act → Grb2nGab1<br>ERK12 --| Grb2nGab1	1) Gab(PH domain) binds to Grb2(SH3 domain), The association of Gab1 with EGFR is thought to occur predominantly via Grb2	16687399
Ras	rat sarcoma viral oncogene homolog	Grb2Sos → Ras	EGF-induced recruitment of the SOS-Grb2 complex to the plasma membrane is critical for the initiation of the MAPK/ERK pathway	8479536
Rac	Ras-Related C3 Botulinum Toxin Substrate	Ras → Rac<br>EGFR → Rac	The pattern of Ras-activation and Rac-activation is significantly different	9314533
Raf1	Raf-1 Proto-Oncogene, Serine/Threonine Kinase	Ras → Raf1<br>PKC → Raf1<br>PKA --| Raf1	1) Active Ras can induce MAP kinase activation in cardiac muscle cells. In addition, phenylephrine-induced activation of the MAP kinases requires Ras activity since a dominant negative Ras mutant (Ala15Ras) and a Ras-blocking, Raf mutant (C4B Raf) prevent activation of the MAP kinase Erk2 by phenylephrine.<br><br>2) Calcineurin activates PKC isoforms in vivo.<br><br>3) PKA can weaken the interaction of Raf-1 with Ras.<br><br>4) PKA can inhibit Raf1 function directly via phosphorylation of the Raf1 kinase domain.<br><br>5) PKA and PKC synergistically activate the Raf-1 in cardiomyocyte.	1) 7802678<br>2) 10788473<br>3), 4) 7935389<br>5) 9299372
MEKK1	Mitogen-Activated Protein Kinase Kinase Kinase 1	Rac → MEKK1	The pattern of MEKK-activation is significantly different from the pattern of Ras- or Rac- activation	9314533
MEKK234	Mitogen-Activated Protein Kinase	Rac → MEKK234	Rac is required as an interactive binding motifs	10799501

	Kinase Kinase 2, 3, 4			
MEKK11	Mitogen-Activated Protein Kinase Kinase Kinase 11	Rac → MEKK11	Rac is required as an interactive binding motifs	10799501
MEK12	Mitogen-Activated Protein Kinase Kinase 1, 2	Raf1→ MEK12	1) Raf1 serves primarily as an ERK activator<br><br>2) Activated Rac had no effect on ERK in the cardiac cells<br><br>3) Only expression of a kinase-inactive inhibitory mutant of MEKK1 and not the kinase-inactive mutants of MEKK2, 3, or 4 inhibit ERK activation by EGF	1),2) 9314533<br>3) 9305638
MEK4	Mitogen-Activated Protein Kinase Kinase 4	MEKK1 → MEK4<br>MEKK23 → MEK4	MEK4 is activated by MEKK1	9405400
MEK7	Mitogen-Activated Protein Kinase Kinase 7	MEKK23 → MEK7<br>MEKK11 → MEK7	MEKK23 directly activates MEK7	10347227
MEK36	Mitogen-Activated Protein Kinase Kinase 3, 6	MEKK11 → MEK36<br>TAK1 →MEK36	1) TAK1 activates MEK36<br><br>2) Activated Rac had no effect on p38	1) 14681216<br>2) 9314533
ERK12	Mitogen-activated protein kinase 3, 1	Gbg → ERK12<br>MEK12 →ERK12	1) MEK12 phosphorylate ERK12 at the conserved TEY motif (amino acids 183-185 in mouse Erk2) and thereby activate ERK12<br><br>2) ERK(Thr188) phoshorylation was shown to be triggered by the association of Gq-derived Gbetagamma subunits with activated ERK12 (Fig 4B)	1) 7601337, 1628739<br>2) 22843704

JNK	Mitogen-Activated Protein Kinase 8	MEK4→ JNK<br>MEK7 → JNK<br>CaN → JNK	1) Inhibition of calcineurin with cyclosporine prevents activation of JNK (p54) during load induced hypertrophy (Figure 7)<br><br>2) JNK is activated through MEK4	1) 10788473<br>2) 16125722
p38	Mitogen-Activated Protein Kinase 14	MEK4 → p38<br>MEK36 → p38<br>RhoA → p38	1) MKK6 stimulates MEF2 by activating p38 MAPK (Figure 1C)<br><br>2) MEK4 partially contributes to the activation of p38<br><br>3) RhoA was shown to be a potent activator of the p38 MAPKs<br><br>4) p38 is activated through MKK3 or MKK6	1) 10737771<br>2) 9314533<br>3) 11641276<br>4) 16125722
PI3Krct	Recruited phosphatidylinositol-4,5-bisphosphate 3-kinase	Grb2 → PI3K<br>bARK → PI3K	PI3K is recruited to receptor	16687399, 1325672
Grb2&Gab1&PI3K	growth factor receptor-bound protein 2(Grb2)-Grb2-associated binding protein 1(Gab1)-phosphatidylinositol-4,5-bisphosphate 3-kinase complex	Grb2 → PI3K	Gab1-mediated recruitment of p85 results in PI3K activation and the production of PIP3 in the plasma membrane	16687399
bARKnPI3K	ß-adrenergic receptor kinase-phosphatidylinositol-4,5-bisphosphate 3-kinase complex	bARK → PI3K	1) From Figure 1 of 15539636, we deduced the relative strength of recruitment of PI3K to each receptor<br><br>2) betaARK as well as Gbetagamma signaling is involved in beta1-AR-mediated PI3K activation	15539636
Akt	V-akt murine thymoma viral oncogene	PI3K → Akt	Akt is activated when binds to PIP3 via its PH domain.	15718470, 16962653

GSK3B	Glycogen synthase kinase 3 beta	Akt --| GSK3B	Akt reverses GSK3B-induced inhibition of GATA4 in cardiac myocytes	11382772
MEF2	Myocyte enhancer factor 2	p38 → MEF2<br>ERK5 → MEF2<br>HDAC --| MEF2	1) MEF2 mediates synergistic transcriptional responses to the CaMK and MAPK signaling pathways MAPKs, which activate MEF2 by phosphorylation of the transcriptional activation domain, maximally stimulate MEF2 activity only when repression by HDACs is relieved by CaMK signaling to the DNA-binding domain (Figure 4A)<br><br>2) BMK1/ERK5 regulates serum-induced early gene expression through transcriptional factor MEF2C (Figure 6B)	1) 10737771<br>2) 9384584
GATA4	GATA binding protein 4	ERK12 → GATA4<br>p38 → GATA4<br>PCK → GATA4<br>GSK3B --| GATA4	1) PKC was shown to enhance activity of GATA4 in cell culture Mutation of the PKC recognition motif (S419A S420A) in GATA4 reduced transciptional activity of GATA4 more than 50%<br><br>2) Only dominant-negative MEK1 blocked PE-induced GATA4 phosphorylation (Figure 2C)<br><br>3) GATA4 activity is positively regulated by p38 MAPK<br><br>4) GSK3B directly phosphorylates GATA4 and thereby decreases basal and betaAR-stimulated GATA4 expression in the nucleus by activating the nuclear export system (Figure 5B)	1) 16260600<br>2) 11585926<br>3) 11641276<br>4) 11382772
cFos	FBJ murine osteosarcoma viral oncogene homolog	ERK12 → cFos	MEK1-ERK1/2 signaling significantly enhanced AP-1 transciptional activity	15657416
cJun	Jun proto-oncogene	JNK → cJun<br>p38 → cJun	In contrast to c-Fos, c-Jun has a basal level of transcription that likely represents its function in basal cellular homeostasis.	15795322

AP1	Activator protein 1	cFos → AP1<br>cJun → AP1	1) AP-1 is homodimers or heterodimers of c-Fos and c-Jun<br><br>2) c-fos, c-fos dimer cannot function as an AP-1<br><br>3) DNJun inhibited the increase in the AP1 activation, 3H-Phe incorporation, cell size enhancement due to ET and PE	1) 15795322<br>2) 11799083
MSK1	Ribosomal protein S6 kinase, 90kDa, polypeptide 5	ERK12 → MSK1<br>p38 → MSK1	MSK1 is a substrate of both ERK12 and p38.	16806820
NOS	Nitric oxide synthase	Akt → NOS	PI3K/Akt-NO signal pathway is involved in cardiomyocyte hypertrophy	26045205
sGC	Sarcoglycan	NOS → sGC	NO activates sGC	17700722
cGMP	Cyclic guanosine monophosphate	sGC → cGMP<br>PDE5 --| cGMP	1) sGC generates cGMP<br><br>2) PDE5 degrades cGMP and regulates the intracellular level of cGMP	1) 17700722<br>2) 10385692
PKG	Protein kinase G	cGMP → PKG<br>PDE5 --| PKG	1) cGMP activates PKG<br><br>2) PKG is activated by PDE5 inhibition	1) 17700722<br>2) 20177073
PDE5	Phosphodiesterase 5	PKG → PDE5	activation of PKG lead to phosphorylation and activation of PDE5	11723116
RGS4	Regulator of G-protein signaling 4	PKG → RGS4	activation of PKG lead to phosphorylation of RGS4, and association of RGS4 and Gq	18443239

Supplementray Data Set S3. Results of the distribution perturbation analyses for apoptosis in the cardiac signaling network.

1a) Result of one-distribution perturbation analysis for apoptosis when each response function was separately applied

		Lin		Hill		Sat		Acc	
		Effect	p-value	Effect	p-value	Effect	p-value	Effect	p-value
Threshold: top 5%	pm1	1.029	0.370	0.999	0.214	1.020	0.462	1.021	0.968
	pm2	0.996	0.617	0.999	0.638	0.995	0.798	1.003	0.640
	pm3	0.989	0.756	1.024	0.627	1.002	0.210	1.009	0.355
	pm4	1.005	0.820	1.098	0.353	1.009	0.896	1.000	0.913
	pm5	0.998	0.972	0.995	0.530	0.998	0.337	1.001	0.739
	pm6	1.021	0.940	1.000	0.786	1.109	0.044	1.014	0.645
	pm7	2.069	<0.001	1.646	<0.001	2.483	<0.001	1.871	<0.001
	pm8	0.999	0.559	0.995	0.444	0.998	0.928	0.997	0.650
	pm9	1.031	0.775	1.053	0.593	1.020	0.985	1.031	0.734
	pm10	2.140	<0.001	1.905	<0.001	1.933	<0.001	2.221	<0.001
	pm11	1.001	0.476	1.010	0.846	0.994	0.105	1.020	0.729
	pm12	1.154	0.024	1.236	<0.001	1.169	0.021	1.254	<0.001
	pm13	1.007	0.549	1.015	0.609	1.003	0.158	1.009	0.727
	pm14	0.998	0.420	1.006	0.912	0.999	0.578	1.007	0.127
	pm15	1.002	0.210	1.007	0.587	1.005	0.750	1.007	0.296
	pm16	0.995	0.701	1.004	0.774	0.997	0.947	1.004	0.952
	pm17	1.001	0.931	1.004	0.885	0.996	0.417	1.004	0.118
	pm18	0.996	0.468	0.998	0.420	0.999	0.980	1.001	0.492
	pm19	0.997	0.532	0.999	0.322	0.999	0.661	1.001	0.395
	pm20	1.001	0.303	1.007	0.190	0.996	0.540	1.005	0.395

	pm21	1.163	0.023	1.273	<0.001	1.484	<0.001	1.167	0.021
	pm22	1.073	0.667	1.156	0.026	1.040	0.644	1.053	0.890
	pm23	1.087	0.726	1.331	<0.001	1.064	0.550	1.059	0.314
	pm24	0.997	0.290	1.018	0.748	0.998	0.810	1.000	0.645
	pm25	0.997	0.740	1.001	0.802	0.999	0.374	1.001	0.272
	pm26	0.997	0.844	1.001	0.447	0.999	0.989	1.001	0.933
	pm27	1.021	0.867	1.041	0.972	1.020	0.177	1.008	0.131
	pm28	1.335	<0.001	1.162	0.027	1.162	0.021	1.450	<0.001
	pm29	1.033	0.148	1.190	0.056	0.995	0.395	1.112	0.603
	pm30	0.918	0.492	0.851	0.023	0.945	0.521	0.873	0.028
	pm31	1.016	0.652	1.042	0.239	1.019	0.633	1.013	0.539
	pm32	0.998	0.439	1.124	0.033	1.000	0.733	1.051	0.749
	pm33	0.997	0.158	1.002	0.959	0.999	0.715	1.001	0.315
	pm34	1.003	0.383	1.007	0.311	1.002	0.375	1.004	0.488
	pm35	1.001	0.555	1.001	0.334	1.012	0.779	1.008	0.625
	pm36	0.996	0.559	0.998	0.927	0.999	0.462	1.001	0.438
	pm37	0.994	0.281	1.008	0.533	0.999	0.846	1.008	0.252
Threshold: top 10%		Lin		Hill		Sat		Acc	
		Effect	p-value	Effect	p-value	Effect	p-value	Effect	p-value
	pm1	1.025	0.752	0.999	0.153	1.018	0.471	1.021	0.934
	pm2	0.995	0.941	0.999	0.731	0.995	0.590	1.002	0.981
	pm3	0.991	0.409	1.022	0.148	1.002	0.565	1.008	0.480
	pm4	1.006	0.308	1.091	0.168	1.007	0.934	1.000	0.991
	pm5	0.998	0.375	0.996	0.899	0.998	0.146	1.001	0.418
	pm6	1.016	0.668	1.000	0.177	1.116	0.042	1.013	0.948

	pm7	2.034	<0.001	1.582	<0.001	2.310	<0.001	1.690	<0.001
	pm8	0.999	0.194	0.995	0.315	0.998	0.651	0.997	0.723
	pm9	1.034	0.154	1.055	0.769	1.022	0.529	1.027	0.434
	pm10	2.130	<0.001	1.769	<0.001	1.906	<0.001	2.271	<0.001
	pm11	1.001	0.368	1.009	0.250	0.994	0.967	1.017	0.756
	pm12	1.123	0.046	1.206	<0.001	1.122	0.045	1.282	<0.001
	pm13	1.007	0.767	1.014	0.239	1.003	0.944	1.008	0.897
	pm14	0.998	0.306	1.006	0.888	0.999	0.184	1.006	0.446
	pm15	1.002	0.537	1.008	0.814	1.004	0.376	1.008	0.809
	pm16	0.995	0.421	1.003	0.210	0.998	0.761	1.004	0.956
	pm17	1.001	0.202	1.004	0.804	0.996	0.342	1.003	0.198
	pm18	0.997	0.665	0.998	0.257	0.999	0.622	1.001	0.133
	pm19	0.997	0.633	0.999	0.189	0.999	0.744	1.001	0.602
	pm20	1.001	0.346	1.007	0.722	0.996	0.168	1.004	0.191
	pm21	1.147	0.022	1.315	<0.001	1.428	<0.001	1.151	0.024
	pm22	1.074	0.497	1.135	0.065	1.050	0.403	1.055	0.496
	pm23	1.092	0.737	1.285	<0.001	1.054	0.361	1.056	0.941
	pm24	0.997	0.859	1.017	0.114	0.998	0.756	1.000	0.906
	pm25	0.997	0.150	1.001	0.499	0.999	0.842	1.001	0.110
	pm26	0.997	0.127	1.001	0.230	0.999	0.533	1.001	0.737
	pm27	1.021	0.988	1.039	0.334	1.017	0.718	1.007	0.678
	pm28	1.319	<0.001	1.137	0.042	1.143	0.027	1.422	<0.001
	pm29	1.035	0.346	1.189	0.016	0.996	0.347	1.087	0.401
	pm30	0.921	0.655	0.875	0.021	0.957	0.231	0.898	0.038
	pm31	1.015	0.531	1.046	0.540	1.020	0.593	1.011	0.679
	pm32	0.998	0.612	1.103	0.205	1.000	0.823	1.049	0.694

	pm33	0.997	0.962	1.002	0.876	0.999	0.667	1.001	0.313
	pm34	1.003	0.461	1.006	0.535	1.002	0.923	1.003	0.239
	pm35	1.001	0.596	1.001	0.536	1.010	0.931	1.008	0.115
	pm36	0.997	0.838	0.998	0.744	0.999	0.397	1.001	0.427
	pm37	0.993	0.389	1.007	0.292	0.999	0.752	1.007	0.237
Threshold: top 20%		Lin		Hill		Sat		Acc	
		Effect	p-value	Effect	p-value	Effect	p-value	Effect	p-value
	pm1	1.021	0.319	0.999	0.288	1.014	0.134	1.019	0.253
	pm2	0.996	0.150	0.999	0.933	0.996	0.436	1.002	0.710
	pm3	0.992	0.245	1.019	0.130	1.002	0.459	1.006	0.934
	pm4	1.004	0.607	1.074	0.821	1.006	0.189	1.000	0.350
	pm5	0.998	0.352	0.997	0.119	0.998	0.283	1.001	0.737
	pm6	1.014	0.802	1.000	0.655	1.096	0.586	1.011	0.355
	pm7	1.907	<0.001	1.494	<0.001	2.074	<0.001	1.583	<0.001
	pm8	0.999	0.551	0.995	0.113	0.998	0.772	0.997	0.631
	pm9	1.029	0.814	1.045	0.992	1.018	0.826	1.021	0.370
	pm10	2.002	<0.001	1.639	<0.001	1.901	<0.001	2.127	<0.001
	pm11	1.001	0.291	1.007	0.109	0.995	0.189	1.015	0.633
	pm12	1.122	0.033	1.161	0.014	1.125	0.038	1.227	<0.001
	pm13	1.005	0.346	1.012	0.747	1.002	0.741	1.007	0.888
	pm14	0.998	0.671	1.005	0.361	0.999	0.601	1.005	0.754
	pm15	1.002	0.751	1.006	0.420	1.003	0.316	1.007	0.159
	pm16	0.996	0.484	1.003	0.544	0.998	0.355	1.004	0.509
	pm17	1.001	0.247	1.003	0.727	0.997	0.376	1.003	0.228
	pm18	0.998	0.928	0.998	0.913	0.999	0.623	1.000	0.321

	pm19	0.998	0.263	0.999	0.740	1.000	0.749	1.000	0.634
	pm20	1.001	0.807	1.006	0.522	0.997	0.961	1.004	0.669
	pm21	1.117	0.038	1.251	<0.001	1.338	<0.001	1.147	0.035
	pm22	1.066	0.122	1.119	0.051	1.035	0.873	1.048	0.932
	pm23	1.081	0.934	1.232	<0.001	1.039	0.854	1.053	0.483
	pm24	0.998	0.648	1.014	0.417	0.998	0.134	1.000	0.979
	pm25	0.997	0.588	1.001	0.277	0.999	0.665	1.000	0.872
	pm26	0.998	0.289	1.001	0.882	0.999	0.278	1.000	0.314
	pm27	1.017	0.107	1.035	0.402	1.014	0.363	1.006	0.990
	pm28	1.249	<0.001	1.117	0.042	1.130	0.034	1.372	<0.001
	pm29	1.029	0.380	1.165	0.016	0.996	0.359	1.079	0.918
	pm30	0.930	0.991	0.891	0.042	0.962	0.130	0.910	0.043
	pm31	1.013	0.840	1.041	0.769	1.018	0.247	1.010	0.479
	pm32	0.998	0.270	1.090	0.255	1.000	0.259	1.045	0.111
	pm33	0.997	0.304	1.002	0.359	0.999	0.134	1.000	0.671
	pm34	1.003	0.925	1.005	0.664	1.002	0.805	1.002	0.890
	pm35	1.001	0.782	1.001	0.392	1.009	0.944	1.007	0.819
	pm36	0.998	0.986	0.998	0.248	0.999	0.152	1.000	0.491
	pm37	0.994	0.144	1.006	0.250	0.999	0.855	1.006	0.276

1b) Result of one-distribution perturbation analysis for apoptosis when four different response function types were applied in a combined manner

	Top 5%		Top 10%		Top 20%	
	Effect	p-value	Effect	p-value	Effect	p-value
pm1	1.020	0.257	1.015	0.527	1.012	0.168
pm2	0.997	0.887	0.997	0.124	0.998	0.787
pm3	0.993	0.227	0.995	0.645	0.995	0.648
pm4	1.004	0.646	1.004	0.862	1.003	0.571
pm5	0.998	0.382	0.999	0.353	0.999	0.884
pm6	1.014	0.438	1.011	0.216	1.008	0.247
pm7	1.888	<0.001	1.700	<0.001	1.385	<0.001
pm8	0.999	0.605	1.000	0.310	1.000	0.128
pm9	1.024	0.226	1.018	0.821	1.017	0.557
pm10	1.876	<0.001	1.667	<0.001	1.597	<0.001
pm11	1.000	0.739	1.000	0.694	1.000	0.421
pm12	1.349	<0.001	1.182	0.012	1.176	0.012
pm13	1.004	0.144	1.003	0.486	1.003	0.708
pm14	0.999	0.118	0.999	0.467	0.999	0.830
pm15	1.002	0.715	1.001	0.334	1.001	0.308
pm16	0.996	0.306	0.997	0.914	0.998	0.346
pm17	1.001	0.279	1.000	0.872	1.000	0.101
pm18	0.998	0.266	0.998	0.101	0.999	0.447
pm19	0.998	0.834	0.998	0.347	0.999	0.230
pm20	1.001	0.630	1.000	0.194	1.000	0.839
pm21	1.397	<0.001	1.214	<0.001	1.174	0.013

pm22	1.055	0.676	1.040	0.739	1.039	0.206
pm23	1.065	0.666	1.060	0.970	1.043	0.122
pm24	0.998	0.799	0.998	0.636	0.999	0.992
pm25	0.998	0.971	0.998	0.644	0.999	0.743
pm26	0.998	0.121	0.998	0.963	0.999	0.893
pm27	1.015	0.811	1.012	0.321	1.011	0.361
pm28	1.332	<0.001	1.188	0.012	1.166	0.028
pm29	1.027	0.380	1.020	0.540	1.017	0.303
pm30	0.947	0.519	0.948	0.317	0.958	0.809
pm31	1.012	0.285	1.009	0.695	1.008	0.889
pm32	0.999	0.533	0.999	0.711	0.999	0.494
pm33	0.998	0.562	0.998	0.557	0.998	0.948
pm34	1.002	0.968	1.002	0.679	1.002	0.969
pm35	1.001	0.735	1.001	0.818	1.000	0.902
pm36	0.998	0.385	0.998	0.530	0.999	0.602
pm37	0.995	0.482	0.996	0.186	0.997	0.625

2a) Result of reverse one-distribution perturbation analysis for apoptosis when each response function was separately applied

		Lin		Hill		Sat		Acc	
		Effect	p-value	Effect	p-value	Effect	p-value	Effect	p-value
Threshold: top 5%	pm1	0.962	0.788	0.972	0.108	0.971	0.182	0.957	0.218
	pm2	0.997	0.474	0.993	0.267	0.996	0.520	1.002	0.542
	pm3	0.990	0.170	0.961	0.476	0.998	0.930	0.989	0.168
	pm4	0.996	0.460	0.900	0.909	0.989	0.985	1.003	0.398
	pm5	0.995	0.390	1.001	0.415	1.000	0.875	0.997	0.656
	pm6	0.987	0.616	0.976	0.915	0.887	0.019	0.992	0.820
	pm7	0.012	<0.001	0.214	<0.001	0.024	<0.001	0.130	<0.001
	pm8	0.995	0.472	0.999	0.999	0.997	0.509	0.996	0.798
	pm9	0.952	0.670	0.957	0.444	0.975	0.334	0.969	0.152
	pm10	0.158	<0.001	0.376	<0.001	0.180	<0.001	0.050	<0.001
	pm11	0.987	0.980	0.996	0.984	0.997	0.860	0.986	0.352
	pm12	0.816	0.009	0.743	<0.001	0.761	<0.001	0.694	<0.001
	pm13	1.010	0.625	1.031	0.133	0.987	0.676	0.995	0.607
	pm14	1.010	0.154	1.001	0.544	0.990	0.377	1.002	0.473
	pm15	0.981	0.702	0.992	0.324	0.983	0.202	0.978	0.469
	pm16	0.997	0.916	1.005	0.239	0.990	0.486	0.985	0.974
	pm17	0.981	0.941	0.998	0.783	0.976	0.987	0.978	0.667
	pm18	0.997	0.336	1.001	0.569	0.999	0.951	1.001	0.120
	pm19	0.997	0.892	0.998	0.484	0.999	0.143	1.001	0.783
	pm20	0.992	0.142	0.979	0.447	0.993	0.606	1.000	0.989
	pm21	0.981	0.611	1.008	0.872	0.643	<0.001	0.967	0.714

	pm22	1.042	0.997	1.078	0.853	1.043	0.194	1.035	0.225
	pm23	0.816	0.009	0.614	<0.001	0.714	<0.001	0.784	<0.001
	pm24	0.996	0.999	0.982	0.249	1.001	0.485	1.003	0.718
	pm25	0.997	0.780	1.001	0.500	0.999	0.675	1.001	0.805
	pm26	0.997	0.378	1.001	0.415	0.999	0.116	1.001	0.976
	pm27	0.982	0.315	0.957	0.363	0.970	0.353	0.990	0.943
	pm28	0.671	<0.001	0.728	<0.001	0.789	<0.001	0.529	<0.001
	pm29	1.248	0.190	1.266	0.985	1.156	0.669	1.318	0.650
	pm30	0.714	<0.001	0.470	<0.001	0.826	0.017	0.633	<0.001
	pm31	0.984	0.546	0.940	0.171	0.971	0.977	0.991	0.581
	pm32	1.001	0.723	0.912	0.037	0.999	0.201	0.932	0.985
	pm33	0.997	0.796	1.000	0.338	0.999	0.418	1.001	0.103
	pm34	0.991	0.475	1.015	0.256	0.994	0.208	0.994	0.261
	pm35	1.008	0.344	1.010	0.937	1.007	0.931	1.001	0.932
	pm36	0.997	0.961	0.997	0.732	0.999	0.574	1.001	0.227
	pm37	0.990	0.191	1.008	0.135	1.000	0.313	1.000	0.509
Threshold: top 10%		Lin		Hill		Sat		Acc	
		Effect	p-value	Effect	p-value	Effect	p-value	Effect	p-value
	pm1	0.969	0.841	0.975	0.566	0.969	0.495	0.964	0.302
	pm2	0.997	0.736	0.992	0.242	0.997	0.883	1.002	0.821
	pm3	0.990	0.464	0.969	0.327	0.998	0.940	0.989	0.127
	pm4	0.996	0.420	0.922	0.321	0.987	0.479	1.003	0.600
	pm5	0.995	0.103	1.001	0.980	1.000	0.194	0.997	0.466
	pm6	0.987	0.336	0.978	0.514	0.883	0.038	0.992	0.946
	pm7	0.116	<0.001	0.412	<0.001	0.139	<0.001	0.242	<0.001

	pm8	0.995	0.309	0.999	0.612	0.997	0.450	0.996	0.402
	pm9	0.962	0.156	0.963	0.898	0.974	0.610	0.965	0.238
	pm10	0.158	<0.001	0.376	<0.001	0.180	<0.001	0.150	<0.001
	pm11	0.990	0.181	0.996	0.445	0.997	0.232	0.988	0.225
	pm12	0.854	0.010	0.806	0.006	0.814	0.008	0.746	<0.001
	pm13	1.009	0.901	1.028	0.914	0.987	0.816	0.995	0.461
	pm14	1.011	0.302	1.001	0.493	0.989	0.826	1.003	0.939
	pm15	0.983	0.475	0.992	0.420	0.981	0.709	0.983	0.294
	pm16	0.998	0.573	1.005	0.218	0.992	0.856	0.986	0.783
	pm17	0.985	0.721	0.998	0.583	0.978	0.924	0.980	0.235
	pm18	0.997	0.371	1.001	0.797	0.999	0.718	1.001	0.751
	pm19	0.997	0.903	0.998	0.833	0.999	0.350	1.001	0.875
	pm20	0.991	0.259	0.981	0.926	0.994	0.348	1.000	0.228
	pm21	0.985	0.336	1.008	0.847	0.694	<0.001	0.974	0.739
	pm22	1.037	0.400	1.083	0.266	1.036	0.435	1.026	0.937
	pm23	0.876	0.032	0.670	<0.001	0.817	0.009	0.837	0.011
	pm24	0.997	0.348	0.985	0.163	1.001	0.540	1.003	0.859
	pm25	0.997	0.887	1.001	0.171	0.999	0.586	1.001	0.933
	pm26	0.997	0.637	1.001	0.186	0.999	0.864	1.001	0.932
	pm27	0.984	0.326	0.957	0.173	0.974	0.777	0.992	0.724
	pm28	0.728	<0.001	0.811	0.011	0.822	0.013	0.605	<0.001
	pm29	1.194	0.536	1.208	0.374	1.123	0.312	1.258	0.885
	pm30	0.749	<0.001	0.565	<0.001	0.886	0.038	0.661	<0.001
	pm31	0.987	0.405	0.947	0.650	0.975	0.336	0.992	0.336
	pm32	1.001	0.736	0.917	0.079	0.999	0.644	0.936	0.468
	pm33	0.997	0.116	1.000	0.702	0.999	0.510	1.001	0.768

	pm34	0.992	0.147	1.015	0.208	0.995	0.664	0.994	0.266
	pm35	1.007	0.467	1.010	0.536	1.005	0.666	1.001	0.546
	pm36	0.997	0.341	0.997	0.516	0.999	0.558	1.001	0.210
	pm37	0.990	0.549	1.009	0.473	1.000	0.202	1.000	0.124
		Lin		Hill		Sat		Acc	
		Effect	p-value	Effect	p-value	Effect	p-value	Effect	p-value
Threshold: top 20%	pm1	0.976	0.300	0.977	0.425	0.974	0.213	0.969	0.873
	pm2	0.998	0.697	0.995	0.603	0.997	0.225	1.001	0.866
	pm3	0.993	0.854	0.976	0.856	0.998	0.243	0.991	0.285
	pm4	0.997	0.910	0.928	0.937	0.989	0.583	1.002	0.795
	pm5	0.996	0.137	1.001	0.964	1.000	0.888	0.997	0.822
	pm6	0.990	0.523	0.983	0.671	0.904	0.080	0.994	0.123
	pm7	0.302	<0.001	0.536	<0.001	0.255	<0.001	0.387	<0.001
	pm8	0.996	0.760	0.999	0.147	0.998	0.778	0.997	0.882
	pm9	0.968	0.731	0.972	0.558	0.978	0.856	0.972	0.458
	pm10	0.274	<0.001	0.431	<0.001	0.394	<0.001	0.290	<0.001
	pm11	0.991	0.384	0.997	0.203	0.998	0.825	0.989	0.478
	pm12	0.888	0.032	0.829	0.011	0.897	0.038	0.754	<0.001
	pm13	1.007	0.616	1.025	0.105	0.990	0.370	0.996	0.822
	pm14	1.008	0.946	1.001	0.998	0.991	0.465	1.002	0.426
	pm15	0.986	0.426	0.995	0.479	0.984	0.362	0.986	0.152
	pm16	0.998	0.375	1.004	0.635	0.994	0.269	0.988	0.888
	pm17	0.988	0.597	0.998	0.415	0.982	0.282	0.982	0.158
	pm18	0.998	0.604	1.000	0.655	1.000	0.632	1.000	0.991
	pm19	0.998	0.347	0.998	0.908	0.999	0.138	1.000	0.835

	pm20	0.992	0.199	0.986	0.143	0.996	0.385	1.000	0.936
	pm21	0.986	0.322	1.006	0.235	0.720	<0.001	0.976	0.681
	pm22	1.033	0.811	1.071	0.253	1.030	0.962	1.024	0.336
	pm23	0.890	0.039	0.729	<0.001	0.863	0.011	0.897	0.042
	pm24	0.997	0.703	0.989	0.982	1.001	0.580	1.002	0.513
	pm25	0.998	0.749	1.001	0.721	0.999	0.832	1.000	0.611
	pm26	0.997	0.237	1.001	0.947	0.999	0.849	1.000	0.930
	pm27	0.988	0.603	0.960	0.181	0.980	0.283	0.994	0.848
	pm28	0.788	<0.001	0.895	0.040	0.847	0.011	0.700	<0.001
	pm29	1.177	0.541	1.175	0.800	1.115	0.529	1.207	0.717
	pm30	0.790	<0.001	0.680	<0.001	0.887	0.041	0.715	<0.001
	pm31	0.989	0.615	0.959	0.414	0.979	0.630	0.994	0.930
	pm32	1.001	0.339	0.920	0.041	1.000	0.701	0.947	0.917
	pm33	0.998	0.697	1.000	0.995	0.999	0.457	1.000	0.673
	pm34	0.994	0.935	1.012	0.899	0.996	0.909	0.995	0.162
	pm35	1.005	0.282	1.008	0.350	1.004	0.273	1.001	0.860
	pm36	0.998	0.889	0.998	0.394	0.999	0.566	1.000	0.285
	pm37	0.992	0.381	1.007	0.932	1.000	0.438	1.000	0.950

2b) Result of reverse one-distribution perturbation analysis for apoptosis in cardiac signaling network when four different response function types were applied in a combined manner

	Top 5%		Top 10%		Top 20%	
	Effect	p-value	Effect	p-value	Effect	p-value
pm1	1.024	0.580	1.022	0.108	1.018	0.160
pm2	0.997	0.515	0.998	0.412	0.998	0.111
pm3	0.993	0.616	0.994	0.947	0.995	0.853
pm4	0.997	0.493	0.998	0.770	0.998	0.465
pm5	0.996	0.741	0.997	0.908	0.997	0.714
pm6	0.991	0.719	0.993	0.474	0.994	0.141
pm7	0.192	<0.001	0.413	<0.001	0.497	<0.001
pm8	0.995	0.455	0.997	0.788	0.997	0.201
pm9	0.965	0.234	0.972	0.729	0.976	0.110
pm10	0.338	<0.001	0.391	<0.001	0.518	<0.001
pm11	0.992	0.914	0.993	0.719	0.995	0.273
pm12	0.775	<0.001	0.853	0.028	0.891	0.040
pm13	1.006	0.396	1.005	0.124	1.004	0.519
pm14	1.009	0.590	1.007	0.603	1.005	0.348
pm15	0.985	0.224	0.987	0.596	0.990	0.618
pm16	0.998	0.511	0.998	0.313	0.999	0.529
pm17	0.986	0.944	0.990	0.180	0.992	0.241
pm18	0.998	0.842	0.998	0.522	0.999	0.283
pm19	0.998	0.153	0.998	0.778	0.999	0.507
pm20	0.993	0.674	0.994	0.114	0.995	0.272
pm21	0.986	0.167	0.988	0.866	0.992	0.834

pm22	1.028	0.512	1.023	0.983	1.021	0.310
pm23	0.797	<0.001	0.823	0.016	0.880	0.018
pm24	0.997	0.360	0.998	0.529	0.998	0.976
pm25	0.998	0.478	0.998	0.995	0.998	0.660
pm26	0.997	0.649	0.998	0.256	0.998	0.137
pm27	0.988	0.566	0.990	0.380	0.992	0.118
pm28	0.778	<0.001	0.812	0.010	0.849	0.023
pm29	1.056	0.531	1.014	0.691	1.011	0.497
pm30	0.782	<0.001	0.822	0.011	0.848	0.022
pm31	0.987	0.123	0.991	0.277	0.992	0.718
pm32	1.001	0.719	1.001	0.885	1.001	0.809
pm33	0.998	0.922	0.998	0.141	0.998	0.681
pm34	0.994	0.236	0.995	0.251	0.996	0.466
pm35	1.006	0.648	1.004	0.455	1.004	0.662
pm36	0.997	0.966	0.998	0.657	0.998	0.520
pm37	0.992	0.408	0.994	0.489	0.995	0.196

3a) Result of two-distribution perturbation analysis for apoptosis when each response function was separately applied (1 million parameter sets)

No.	Pair of perturbed parameter distributions	Lin	Hill	Sat	Acc	Synergistic effect	p-value
1	pm1-pm2	1.036	1.061	1	1.024	0.016	0.35
2	pm1-pm3	1.016	1.086	1.038	1.041	0.024	0.28
3	pm1-pm4	1.038	1.039	1.043	1.016	-0.008	0.569
4	pm1-pm5	0.997	0.992	1.005	1.006	-0.014	0.387
5	pm1-pm6	1.059	1.042	1.134	1.052	0.02	0.369
6	pm1-pm7	1.951	1.598	2.073	1.591	-0.116	0.008
7	pm1-pm8	1.018	1.048	1	1.025	0.01	0.405
8	pm1-pm9	1.058	1.125	1.047	1.048	0.02	0.399
9	pm1-pm10	2.258	1.879	2.052	2.261	0.078	0.012
10	pm1-pm11	1.048	1.061	1.004	1.046	0.019	0.338
11	pm1-pm12	1.148	1.208	1.067	1.28	-0.023	0.213
12	pm1-pm13	1.008	1.055	1.02	0.999	-0.003	0.736
13	pm1-pm14	0.983	1.087	0.974	1.009	-0.005	0.656
14	pm1-pm15	1.03	1.058	1.062	1.045	0.027	0.259
15	pm1-pm16	0.988	1.061	1.055	1.027	0.017	0.331
16	pm1-pm17	1	1.024	1.011	1.001	-0.008	0.409
17	pm1-pm18	1.007	1.012	1.038	1.026	0.006	0.565
18	pm1-pm19	1.011	1.029	1.015	1.031	0.007	0.423
19	pm1-pm20	1.015	1.019	0.992	1.024	-0.005	0.507
20	pm1-pm21	1.158	1.313	1.41	1.037	-0.047	0.168
21	pm1-pm22	1.087	1.183	1.103	1.072	0.017	0.352
22	pm1-pm23	1.135	1.249	1.117	1.057	0.002	0.835
23	pm1-pm24	0.999	1.068	1.004	1.031	0.007	0.569
24	pm1-pm25	1.019	1.025	1.022	1.034	0.01	0.598

25	pm1-pm26	1.028	1.039	0.992	1.009	0.002	0.782
26	pm1-pm27	1.009	1.09	1.021	1.038	0.003	0.776
27	pm1-pm28	1.262	1.105	1.137	1.382	-0.049	0.161
28	pm1-pm29	0.992	1.211	1.032	1.088	-0.011	0.359
29	pm1-pm30	0.931	0.949	0.97	0.926	0.015	0.376
30	pm1-pm31	1.021	1.052	1.046	1.022	-0.004	0.84
31	pm1-pm32	1.022	1.187	1.05	1.053	0.025	0.226
32	pm1-pm33	0.992	1.033	1.012	1.019	-0.002	0.918
33	pm1-pm34	1.042	1.074	1.011	1.005	0.013	0.378
34	pm1-pm35	1.037	1.093	1.02	1.031	0.025	0.278
35	pm1-pm36	1.018	1.034	0.995	1.038	0.007	0.579
36	pm1-pm37	1.011	1.03	0.985	1.014	-0.008	0.566
37	pm2-pm3	0.986	1.023	1.031	0.996	0.005	0.553
38	pm2-pm4	0.997	1.066	1.041	0.979	-0.003	0.72
39	pm2-pm5	0.979	1	0.988	1.02	0.001	0.719
40	pm2-pm6	1.007	1.013	1.104	1.027	0.004	0.602
41	pm2-pm7	1.96	1.553	2.079	1.616	-0.1	0.008
42	pm2-pm8	0.995	1.018	0.992	0.979	0.001	0.93
43	pm2-pm9	1.037	1.055	1.011	1.028	0.001	0.998
44	pm2-pm10	2.284	1.898	2.183	2.302	0.15	0.009
45	pm2-pm11	1.001	1.011	0.972	1.01	-0.005	0.831
46	pm2-pm12	1.105	1.144	1.051	1.188	-0.059	0.031
47	pm2-pm13	0.983	1.053	0.991	0.989	-0.002	0.918
48	pm2-pm14	0.988	1.029	1.016	1.009	0.01	0.312
49	pm2-pm15	0.997	1.035	1.018	1.018	0.014	0.362
50	pm2-pm16	1.028	1.039	1.003	1.014	0.023	0.29
51	pm2-pm17	1	1.004	0.985	0.974	-0.008	0.531
52	pm2-pm18	0.991	0.999	0.981	1.018	0.001	0.804
53	pm2-pm19	1.001	1.022	0.993	0.995	0.006	0.434
54	pm2-pm20	1.015	1.033	0.996	0.993	0.009	0.546

55	pm2-pm21	1.093	1.302	1.39	1.007	-0.06	0.021
56	pm2-pm22	1.045	1.135	1.096	1.011	-0.005	0.98
57	pm2-pm23	1.064	1.239	1.116	1.051	-0.002	0.641
58	pm2-pm24	0.966	1.03	1.021	0.975	-0.003	0.623
59	pm2-pm25	0.991	1	0.997	1.017	0.004	0.634
60	pm2-pm26	0.981	0.988	0.987	1.014	-0.005	0.718
61	pm2-pm27	1.002	1.012	1.037	1.008	-0.004	0.773
62	pm2-pm28	1.216	1.066	1.108	1.344	-0.07	0.023
63	pm2-pm29	1.024	1.235	0.998	1.054	0.003	0.886
64	pm2-pm30	0.923	0.921	0.992	0.892	0.021	0.219
65	pm2-pm31	0.984	1.029	1.031	0.993	-0.012	0.327
66	pm2-pm32	0.988	1.136	1.026	1.038	0.012	0.349
67	pm2-pm33	0.969	0.982	0.997	0.995	-0.012	0.313
68	pm2-pm34	0.984	1.01	1.023	1.021	0.008	0.548
69	pm2-pm35	1.026	1.011	0.991	1.003	0.005	0.728
70	pm2-pm36	0.991	1.007	1.016	1.002	0.007	0.474
71	pm2-pm37	0.992	0.991	1.003	1.027	0.003	0.851
72	pm3-pm4	0.987	1.038	0.992	1.008	-0.025	0.279
73	pm3-pm5	0.976	1.031	1.017	1.025	0.008	0.446
74	pm3-pm6	1.046	1.027	1.081	1.018	0.001	0.818
75	pm3-pm7	1.951	1.58	2.084	1.62	-0.101	0.007
76	pm3-pm8	1.008	1.031	1.021	1	0.012	0.376
77	pm3-pm9	1.038	1.022	1.047	1.03	-0.006	0.405
78	pm3-pm10	2.282	1.942	2.455	2.038	0.155	0.002
79	pm3-pm11	0.999	1.007	1.01	1.004	-0.005	0.583
80	pm3-pm12	1.11	1.214	1.042	1.21	-0.045	0.16
81	pm3-pm13	0.972	1.058	0.994	0.97	-0.015	0.305
82	pm3-pm14	1.009	1.054	0.998	0.985	0.004	0.886
83	pm3-pm15	0.985	1.028	1.018	1.01	-0.001	0.652
84	pm3-pm16	0.997	1.017	1.022	1.014	0.007	0.555

85	pm3-pm17	1.005	1.02	1.035	1.004	0.009	0.527
86	pm3-pm18	1.022	1.026	1.014	1.023	0.017	0.304
87	pm3-pm19	0.99	1.015	1.001	1.001	-0.003	0.681
88	pm3-pm20	1.006	1.031	1.022	1.003	0.008	0.443
89	pm3-pm21	1.116	1.299	1.341	1.006	-0.076	0.012
90	pm3-pm22	1.091	1.178	1.075	1.006	0.003	0.884
91	pm3-pm23	1.096	1.231	1.157	1.066	0.01	0.503
92	pm3-pm24	1.001	1.036	0.995	1.012	0.002	0.972
93	pm3-pm25	1.016	1.003	1.01	0.984	-0.002	0.936
94	pm3-pm26	0.995	1.029	1.013	1.019	0.009	0.463
95	pm3-pm27	1.009	1.029	1.026	1.001	-0.01	0.329
96	pm3-pm28	1.218	1.084	1.141	1.327	-0.069	0.027
97	pm3-pm29	0.971	1.189	0.999	1.036	-0.034	0.298
98	pm3-pm30	0.911	0.908	0.946	0.892	-0.005	0.88
99	pm3-pm31	1.021	1.061	1.013	0.985	-0.009	0.498
100	pm3-pm32	0.991	1.139	1.04	1.046	0.011	0.336
101	pm3-pm33	0.986	0.997	0.993	0.978	-0.017	0.312
102	pm3-pm34	1.042	1.071	1.014	0.982	0.018	0.322
103	pm3-pm35	0.985	1.005	1.024	0.981	-0.012	0.35
104	pm3-pm36	0.979	0.963	0.999	1.018	-0.015	0.358
105	pm3-pm37	1.002	0.997	1.022	1.019	0.003	0.848
106	pm4-pm5	1.001	1.041	1.003	1.023	-0.007	0.522
107	pm4-pm6	1.009	1.032	1.102	0.997	-0.027	0.234
108	pm4-pm7	1.898	1.608	2.094	1.598	-0.13	0.006
109	pm4-pm8	1.017	1.073	1.004	1.015	0.004	0.757
110	pm4-pm9	1.041	1.077	1.038	1.021	-0.016	0.365
111	pm4-pm10	2.074	1.863	2.009	2.147	-0.022	0.265
112	pm4-pm11	0.986	1.049	1.017	1.029	-0.011	0.343
113	pm4-pm12	1.116	1.213	1.068	1.223	-0.054	0.028
114	pm4-pm13	0.98	1.023	1.037	0.963	-0.033	0.207

115	pm4-pm14	0.994	1.075	1.006	1.015	-0.006	0.58
116	pm4-pm15	1.003	1.085	1.048	0.996	0.002	0.991
117	pm4-pm16	1.034	1.073	1.019	0.973	-0.001	0.748
118	pm4-pm17	1.014	1.063	1.02	0.999	-0.003	0.657
119	pm4-pm18	0.994	1.024	1.018	0.983	-0.02	0.305
120	pm4-pm19	1.001	1.022	1	0.989	-0.022	0.269
121	pm4-pm20	1.01	1.078	1.031	1.002	0.002	0.871
122	pm4-pm21	1.155	1.311	1.425	1.039	-0.054	0.041
123	pm4-pm22	1.063	1.217	1.14	1.03	0.008	0.426
124	pm4-pm23	1.151	1.424	1.126	1.07	0.045	0.158
125	pm4-pm24	1.007	1.056	1.039	0.985	-0.008	0.518
126	pm4-pm25	0.995	1.077	0.992	1.005	-0.008	0.558
127	pm4-pm26	1.029	1.067	0.993	0.975	-0.009	0.414
128	pm4-pm27	1.031	1.045	1.014	1.002	-0.024	0.222
129	pm4-pm28	1.242	1.143	1.156	1.318	-0.066	0.041
130	pm4-pm29	1.033	1.344	1.001	1.089	0.014	0.326
131	pm4-pm30	0.929	0.958	0.966	0.88	-0.005	0.593
132	pm4-pm31	1.008	1.066	1.057	1.029	-0.009	0.483
133	pm4-pm32	1.001	1.16	1.025	1.031	-0.009	0.543
134	pm4-pm33	1.023	1.047	1.009	1.001	-0.006	0.495
135	pm4-pm34	0.99	1.112	1.05	0.977	0.003	0.99
136	pm4-pm35	1.028	1.042	1.006	0.994	-0.013	0.371
137	pm4-pm36	0.983	1.024	1.038	1.037	-0.004	0.842
138	pm4-pm37	1.02	1.049	1.016	0.988	-0.009	0.465
139	pm5-pm6	1.032	0.986	1.12	1.003	0.001	0.736
140	pm5-pm7	1.937	1.583	2.1	1.597	-0.098	0.015
141	pm5-pm8	0.994	1.008	0.994	0.973	-0.003	0.604
142	pm5-pm9	1.012	1.033	1.01	1.044	-0.008	0.446
143	pm5-pm10	2.133	1.728	1.96	2.2	-0.012	0.328
144	pm5-pm11	0.989	0.978	0.991	1.027	-0.007	0.487

145	pm5-pm12	1.132	1.167	1.007	1.246	-0.043	0.166
146	pm5-pm13	0.988	0.992	1.004	0.98	-0.015	0.323
147	pm5-pm14	0.989	1.031	1.009	1.003	0.007	0.514
148	pm5-pm15	0.987	1.014	0.99	1.002	-0.005	0.581
149	pm5-pm16	0.995	0.997	1.006	0.987	-0.002	0.684
150	pm5-pm17	1.002	0.974	0.994	0.991	-0.009	0.522
151	pm5-pm18	1.02	1.004	0.989	0.982	0.002	0.732
152	pm5-pm19	0.975	0.987	0.999	0.997	-0.008	0.465
153	pm5-pm20	0.975	1.001	0.976	0.992	-0.014	0.364
154	pm5-pm21	1.1	1.305	1.356	1.046	-0.057	0.044
155	pm5-pm22	1.057	1.131	1.073	1.035	-0.003	0.774
156	pm5-pm23	1.073	1.187	1.126	1.055	-0.01	0.447
157	pm5-pm24	1.007	0.988	1.007	0.999	-0.001	0.657
158	pm5-pm25	0.992	0.981	0.979	1.001	-0.009	0.439
159	pm5-pm26	0.982	0.98	0.982	0.985	-0.016	0.355
160	pm5-pm27	0.992	1.021	1.012	1.013	-0.01	0.46
161	pm5-pm28	1.2	1.056	1.135	1.385	-0.06	0.021
162	pm5-pm29	1.013	1.157	0.95	1.106	-0.018	0.399
163	pm5-pm30	0.918	0.871	1.008	0.881	0.009	0.546
164	pm5-pm31	1.021	1.04	1.041	1.043	0.015	0.347
165	pm5-pm32	1.01	1.141	1.013	1.033	0.014	0.331
166	pm5-pm33	1.023	0.989	0.994	1.007	0.005	0.475
167	pm5-pm34	0.997	1.015	1.013	0.972	-0.003	0.615
168	pm5-pm35	1.011	1.021	1.007	0.997	0.006	0.435
169	pm5-pm36	1.004	1.018	1.015	1.01	0.015	0.303
170	pm5-pm37	0.99	1.008	1.018	1.012	0.007	0.542
171	pm6-pm7	1.997	1.544	2.276	1.657	-0.072	0.019
172	pm6-pm8	1.016	1.052	1.113	1.028	0.018	0.333
173	pm6-pm9	1.055	1.034	1.13	1.045	-0.004	0.612
174	pm6-pm10	2.271	1.951	2.296	2.345	0.161	0.004

175	pm6-pm11	1.041	1.002	1.098	1.009	-0.004	0.999
176	pm6-pm12	1.115	1.19	1.124	1.25	-0.05	0.195
177	pm6-pm13	0.993	1.013	1.087	1.004	-0.02	0.37
178	pm6-pm14	1.023	1.083	1.087	1.018	0.014	0.389
179	pm6-pm15	1.046	1.02	1.128	1.04	0.016	0.315
180	pm6-pm16	1.045	1.008	1.084	1.033	0.006	0.525
181	pm6-pm17	1.042	1.014	1.093	0.991	-0.002	0.672
182	pm6-pm18	1.018	0.998	1.102	1.015	-0.002	0.903
183	pm6-pm19	1.013	1.027	1.112	1.033	0.011	0.335
184	pm6-pm20	1.037	1.02	1.11	1.016	0.008	0.525
185	pm6-pm21	1.131	1.323	1.527	1.043	-0.041	0.183
186	pm6-pm22	1.095	1.139	1.187	1.015	-0.006	0.451
187	pm6-pm23	1.068	1.25	1.2	1.06	-0.014	0.387
188	pm6-pm24	1.036	1.027	1.1	1.025	0.008	0.533
189	pm6-pm25	1.012	1.008	1.109	0.982	-0.008	0.533
190	pm6-pm26	1.033	0.991	1.093	1.031	0.001	0.646
191	pm6-pm27	1.053	1.06	1.145	1.025	0.013	0.323
192	pm6-pm28	1.299	1.095	1.209	1.312	-0.063	0.041
193	pm6-pm29	0.996	1.156	1.12	1.115	-0.016	0.362
194	pm6-pm30	0.931	0.945	1.056	0.917	0.013	0.375
195	pm6-pm31	1.044	1.038	1.074	1.023	-0.015	0.389
196	pm6-pm32	1.026	1.15	1.124	1.069	0.018	0.359
197	pm6-pm33	1.031	1.012	1.138	1.016	0.013	0.399
198	pm6-pm34	1.04	1.007	1.118	0.992	-0.001	0.908
199	pm6-pm35	1.026	1.006	1.081	1.012	-0.01	0.424
200	pm6-pm36	1.045	1.028	1.102	1.003	0.01	0.564
201	pm6-pm37	1.046	1.027	1.116	1.005	0.01	0.317
202	pm7-pm8	1.99	1.637	2.088	1.584	-0.077	0.011
203	pm7-pm9	1.994	1.588	2.136	1.638	-0.099	0.013
204	pm7-pm10	3.999	2.559	3.892	3.354	0.528	<0.001

205	pm7-pm11	1.953	1.633	2.11	1.632	-0.077	0.013
206	pm7-pm12	2.128	1.767	2.169	1.926	-0.09	0.011
207	pm7-pm13	1.942	1.559	2.048	1.578	-0.13	0.005
208	pm7-pm14	2.013	1.556	2.114	1.584	-0.09	0.01
209	pm7-pm15	1.976	1.589	2.047	1.564	-0.116	0.007
210	pm7-pm16	1.984	1.593	2.072	1.612	-0.089	0.016
211	pm7-pm17	1.995	1.535	2.121	1.601	-0.092	0.019
212	pm7-pm18	1.957	1.522	2.03	1.554	-0.137	0.006
213	pm7-pm19	1.96	1.54	2.127	1.57	-0.104	0.008
214	pm7-pm20	2.003	1.565	2.068	1.6	-0.097	0.02
215	pm7-pm21	2.141	1.931	2.79	1.616	-0.045	0.151
216	pm7-pm22	2.089	1.712	2.277	1.659	-0.048	0.163
217	pm7-pm23	2.075	1.827	2.22	1.657	-0.081	0.019
218	pm7-pm24	1.966	1.602	2.118	1.579	-0.091	0.014
219	pm7-pm25	1.952	1.523	2.093	1.596	-0.113	0.007
220	pm7-pm26	1.992	1.593	2.083	1.595	-0.088	0.018
221	pm7-pm27	1.976	1.544	2.097	1.641	-0.111	0.009
222	pm7-pm28	2.444	1.62	2.338	2.196	-0.01	0.435
223	pm7-pm29	2.073	1.85	2.041	1.698	-0.066	0.021
224	pm7-pm30	2.09	1.7	2.366	1.457	0.086	0.015
225	pm7-pm31	2.021	1.676	2.101	1.623	-0.072	0.016
226	pm7-pm32	1.939	1.609	2.035	1.639	-0.136	0.005
227	pm7-pm33	1.962	1.532	2.078	1.586	-0.114	0.006
228	pm7-pm34	1.962	1.593	2.066	1.596	-0.104	0.008
229	pm7-pm35	1.987	1.535	2.09	1.59	-0.108	0.007
230	pm7-pm36	1.953	1.547	2.108	1.58	-0.106	0.009
231	pm7-pm37	1.972	1.55	2.093	1.598	-0.103	0.007
232	pm8-pm9	0.99	1.08	1.025	1.021	-0.003	0.673
233	pm8-pm10	2.268	2	2.162	2.042	0.102	0.008
234	pm8-pm11	1.019	0.991	0.995	1.008	0.001	0.654

235	pm8-pm12	1.077	1.165	1.031	1.206	-0.061	0.026
236	pm8-pm13	0.977	0.998	0.977	0.966	-0.026	0.266
237	pm8-pm14	0.998	1.053	0.982	0.986	0.005	0.499
238	pm8-pm15	1.039	0.965	1.02	1.031	0.011	0.342
239	pm8-pm16	0.996	1.036	1.032	0.992	0.016	0.376
240	pm8-pm17	1.015	1.003	1.017	0.993	0.009	0.537
241	pm8-pm18	1.01	0.989	0.976	1.015	0.001	0.855
242	pm8-pm19	1.003	0.972	0.988	1.001	-0.006	0.476
243	pm8-pm20	0.982	1.029	0.994	1.016	0.006	0.522
244	pm8-pm21	1.125	1.276	1.372	1.016	-0.06	0.03
245	pm8-pm22	1.074	1.168	1.105	1.044	0.022	0.201
246	pm8-pm23	1.075	1.25	1.117	1.052	0.004	0.745
247	pm8-pm24	0.968	1.007	1.014	1.002	-0.003	0.986
248	pm8-pm25	0.983	1.021	0.981	1	-0.001	0.847
249	pm8-pm26	0.996	0.998	0.977	1.003	-0.003	0.853
250	pm8-pm27	1.003	1.031	1.02	1.003	-0.004	0.736
251	pm8-pm28	1.232	1.077	1.092	1.346	-0.066	0.029
252	pm8-pm29	1.019	1.161	0.998	1.077	-0.01	0.377
253	pm8-pm30	0.938	0.893	0.949	0.923	0.016	0.304
254	pm8-pm31	1.004	1.054	1.033	1.009	0.004	0.742
255	pm8-pm32	0.977	1.113	1.013	1.025	-0.003	0.935
256	pm8-pm33	0.991	0.996	0.985	0.995	-0.005	0.445
257	pm8-pm34	0.986	1.039	1.008	0.981	0.003	0.8
258	pm8-pm35	1.021	1.055	0.992	0.986	0.011	0.35
259	pm8-pm36	0.992	1.006	1.011	1.004	0.007	0.505
260	pm8-pm37	1.001	0.992	1.004	1.006	0.002	0.744
261	pm9-pm10	2.292	2.022	2.194	2.06	0.089	0.017
262	pm9-pm11	1.029	1.03	1.033	1.037	-0.007	0.599
263	pm9-pm12	1.162	1.182	1.083	1.259	-0.046	0.198
264	pm9-pm13	0.998	1.039	1.029	1.005	-0.024	0.271

265	pm9-pm14	1.016	1.076	1.057	1.035	0.009	0.529
266	pm9-pm15	1.022	1.038	1.075	1.019	-0.001	0.807
267	pm9-pm16	1.067	1.027	1.006	1.031	-0.001	0.766
268	pm9-pm17	1.019	1.059	1.06	0.99	-0.003	0.609
269	pm9-pm18	1.022	0.998	1.017	1.028	-0.017	0.34
270	pm9-pm19	1.028	1.029	1.02	1.045	-0.003	0.766
271	pm9-pm20	1.031	1.017	1.031	1.028	-0.01	0.568
272	pm9-pm21	1.151	1.349	1.413	1.042	-0.056	0.027
273	pm9-pm22	1.098	1.212	1.147	1.026	0.008	0.452
274	pm9-pm23	1.097	1.293	1.136	1.041	-0.015	0.37
275	pm9-pm24	1.047	1.05	1.006	1.003	-0.011	0.302
276	pm9-pm25	1.056	1.022	1.047	1.019	0.002	0.681
277	pm9-pm26	1.028	1.059	1.025	1.042	0.005	0.695
278	pm9-pm27	1.032	1.059	1.048	1.039	-0.011	0.354
279	pm9-pm28	1.262	1.104	1.16	1.366	-0.067	0.022
280	pm9-pm29	1.038	1.199	1.049	1.101	-0.014	0.312
281	pm9-pm30	0.962	0.952	1.016	0.908	0.012	0.312
282	pm9-pm31	1.046	1.102	1.058	1.019	-0.001	0.719
283	pm9-pm32	1.013	1.167	1.026	1.047	-0.009	0.595
284	pm9-pm33	1.03	1.067	1.038	1.006	0.001	0.699
285	pm9-pm34	1.044	1.062	1.015	1.002	-0.007	0.543
286	pm9-pm35	1.015	1.063	1.037	0.993	-0.012	0.389
287	pm9-pm36	1.009	1.057	1.023	1.004	-0.01	0.344
288	pm9-pm37	1.033	1.066	1.031	1.013	0	0.902
289	pm10-pm11	2.295	1.946	2.154	2.083	0.096	0.014
290	pm10-pm12	2.2	1.992	1.922	2.437	-0.065	0.044
291	pm10-pm13	2.283	2	2.132	2.014	0.08	0.016
292	pm10-pm14	2.262	1.861	2.064	2.222	0.081	0.018
293	pm10-pm15	2.207	1.881	2.168	2.112	0.067	0.03
294	pm10-pm16	2.005	1.756	1.854	2.016	-0.111	0.006

295	pm10-pm17	2.314	1.971	2.113	2.049	0.092	0.014
296	pm10-pm18	2.003	1.748	1.889	2.064	-0.092	0.017
297	pm10-pm19	2.166	1.89	2.071	2.278	0.083	0.013
298	pm10-pm20	2.307	2.005	2.149	2.053	0.107	0.009
299	pm10-pm21	2.214	2.142	2.587	2.074	-0.025	0.283
300	pm10-pm22	2.07	1.874	1.97	2.09	-0.097	0.019
301	pm10-pm23	2.107	1.96	2.037	2.129	-0.083	0.013
302	pm10-pm24	2.098	1.852	1.963	2.127	-0.012	0.341
303	pm10-pm25	2.109	1.754	1.996	2.149	-0.016	0.329
304	pm10-pm26	2.315	1.913	2.189	2.073	0.104	0.009
305	pm10-pm27	2.051	1.736	1.876	2.07	-0.107	0.005
306	pm10-pm28	2.401	1.714	2.069	2.725	-0.047	0.184
307	pm10-pm29	2.018	1.908	1.887	2.206	-0.091	0.017
308	pm10-pm30	2.138	1.982	2.073	1.838	0.076	0.013
309	pm10-pm31	2.029	1.712	1.89	2.098	-0.11	0.01
310	pm10-pm32	2.166	1.953	2.084	2.319	0.074	0.017
311	pm10-pm33	2.264	1.949	2.167	2.009	0.079	0.017
312	pm10-pm34	2.29	2.022	2.147	2.066	0.108	0.009
313	pm10-pm35	2.302	1.998	2.093	2.057	0.089	0.019
314	pm10-pm36	2.268	2.002	2.171	2.016	0.096	0.013
315	pm10-pm37	2.292	1.985	2.196	2.012	0.1	0.008
316	pm11-pm12	1.099	1.182	1.041	1.263	-0.042	0.162
317	pm11-pm13	0.987	1.05	1.008	0.977	-0.007	0.509
318	pm11-pm14	1.02	1.066	1.035	1.005	0.025	0.289
319	pm11-pm15	1.016	1.013	0.983	1.016	-0.004	0.986
320	pm11-pm16	0.993	1.045	1.004	1.043	0.016	0.312
321	pm11-pm17	0.982	0.995	0.981	0.971	-0.024	0.238
322	pm11-pm18	1.003	0.971	1.006	1.027	-0.002	0.871
323	pm11-pm19	0.965	0.985	1.009	0.982	-0.019	0.327
324	pm11-pm20	1	1.018	0.995	0.992	-0.006	0.558

325	pm11-pm21	1.096	1.277	1.351	1.011	-0.082	0.02
326	pm11-pm22	1.042	1.184	1.093	1.04	0.006	0.486
327	pm11-pm23	1.078	1.258	1.14	1.054	0.006	0.478
328	pm11-pm24	0.994	1.021	1.003	1.001	-0.004	0.875
329	pm11-pm25	0.997	1.017	1.008	1.015	0.005	0.532
330	pm11-pm26	0.991	0.987	1.01	1.02	-0.002	0.706
331	pm11-pm27	1.03	1.041	1.026	1.009	0	0.737
332	pm11-pm28	1.233	1.058	1.103	1.349	-0.075	0.019
333	pm11-pm29	1.005	1.187	0.98	1.068	-0.022	0.294
334	pm11-pm30	0.933	0.864	0.982	0.911	0.005	0.4
335	pm11-pm31	1.023	1.048	1.026	1.009	-0.002	0.785
336	pm11-pm32	0.981	1.128	1.007	1.047	-0.002	0.884
337	pm11-pm33	1.015	1.003	0.981	1.043	0.006	0.51
338	pm11-pm34	1.012	1.046	1.013	0.994	0.008	0.542
339	pm11-pm35	0.969	1.004	1.016	1	-0.013	0.348
340	pm11-pm36	0.977	0.964	1	1.027	-0.011	0.387
341	pm11-pm37	0.984	0.956	0.993	1.042	-0.013	0.334
342	pm12-pm13	1.098	1.182	1.003	1.234	-0.062	0.025
343	pm12-pm14	1.094	1.196	1.075	1.234	-0.036	0.249
344	pm12-pm15	1.11	1.139	1.033	1.28	-0.048	0.182
345	pm12-pm16	1.138	1.229	1.055	1.223	-0.022	0.281
346	pm12-pm17	1.113	1.237	1.031	1.225	-0.033	0.298
347	pm12-pm18	1.088	1.185	1.038	1.227	-0.047	0.186
348	pm12-pm19	1.087	1.163	1.027	1.205	-0.062	0.041
349	pm12-pm20	1.111	1.139	1.039	1.195	-0.064	0.041
350	pm12-pm21	1.267	1.525	1.414	1.249	-0.08	0.014
351	pm12-pm22	1.167	1.342	1.155	1.249	-0.034	0.254
352	pm12-pm23	1.193	1.42	1.137	1.304	-0.041	0.198
353	pm12-pm24	1.095	1.156	1.041	1.233	-0.055	0.028
354	pm12-pm25	1.119	1.163	1.051	1.224	-0.043	0.192

355	pm12-pm26	1.103	1.17	1.037	1.197	-0.056	0.029
356	pm12-pm27	1.118	1.184	1.061	1.257	-0.049	0.152
357	pm12-pm28	1.377	1.232	1.19	1.636	-0.08	0.011
358	pm12-pm29	1.126	1.411	1.06	1.297	-0.036	0.254
359	pm12-pm30	1.042	1.054	0.938	1.146	-0.051	0.033
360	pm12-pm31	1.117	1.206	1.089	1.238	-0.044	0.19
361	pm12-pm32	1.097	1.303	1.016	1.291	-0.044	0.184
362	pm12-pm33	1.08	1.125	1.051	1.217	-0.065	0.024
363	pm12-pm34	1.137	1.201	1.055	1.165	-0.047	0.177
364	pm12-pm35	1.077	1.24	1.05	1.191	-0.049	0.153
365	pm12-pm36	1.1	1.151	1.031	1.199	-0.062	0.035
366	pm12-pm37	1.093	1.15	1.014	1.226	-0.064	0.024
367	pm13-pm14	0.992	1.026	0.995	1.013	-0.004	0.937
368	pm13-pm15	0.993	0.976	1.009	1.001	-0.019	0.302
369	pm13-pm16	1.007	0.973	1.005	0.99	-0.014	0.327
370	pm13-pm17	0.959	1.002	0.979	0.971	-0.031	0.242
371	pm13-pm18	0.989	1.03	0.975	0.982	-0.013	0.382
372	pm13-pm19	1.027	1.019	1.008	0.959	-0.004	0.997
373	pm13-pm20	1.027	1.022	1.002	1.013	0.006	0.407
374	pm13-pm21	1.082	1.313	1.37	0.991	-0.079	0.015
375	pm13-pm22	1.03	1.139	1.093	0.989	-0.024	0.247
376	pm13-pm23	1.086	1.203	1.107	1.042	-0.02	0.228
377	pm13-pm24	0.986	1.027	1.001	1.017	-0.003	0.786
378	pm13-pm25	0.994	1.045	0.995	0.992	-0.001	0.623
379	pm13-pm26	0.989	1.03	0.99	0.963	-0.014	0.305
380	pm13-pm27	0.979	1.074	1.008	0.998	-0.015	0.373
381	pm13-pm28	1.248	1.137	1.132	1.346	-0.047	0.161
382	pm13-pm29	0.972	1.112	0.978	1.058	-0.055	0.023
383	pm13-pm30	0.902	0.893	0.931	0.898	-0.015	0.336
384	pm13-pm31	1.016	1.048	1.022	0.989	-0.012	0.347

385	pm13-pm32	0.974	1.15	1.01	1.016	-0.008	0.402
386	pm13-pm33	0.999	1.063	0.994	1.009	0.009	0.516
387	pm13-pm34	1.004	0.999	0.992	0.962	-0.023	0.27
388	pm13-pm35	0.956	1.005	0.996	0.942	-0.038	0.266
389	pm13-pm36	0.981	1.038	1.008	1.011	0.003	0.971
390	pm13-pm37	0.996	1.06	1.009	0.98	0.002	0.728
391	pm14-pm15	1.023	0.978	1.004	0.981	-0.011	0.387
392	pm14-pm16	0.971	0.99	1.002	0.962	-0.021	0.223
393	pm14-pm17	1.015	0.982	1.004	0.972	-0.01	0.44
394	pm14-pm18	1.021	1.038	1.017	0.977	0.012	0.334
395	pm14-pm19	1.021	1.044	1.007	0.967	0.008	0.507
396	pm14-pm20	0.981	1.036	0.999	0.989	-0.003	0.912
397	pm14-pm21	1.11	1.341	1.401	1.013	-0.047	0.177
398	pm14-pm22	1.075	1.144	1.088	1.024	0.002	0.637
399	pm14-pm23	1.053	1.197	1.117	1.016	-0.028	0.279
400	pm14-pm24	1	1.055	0.986	1.001	0.005	0.766
401	pm14-pm25	1.013	1.056	1.023	1.011	0.024	0.295
402	pm14-pm26	0.989	1.02	1.031	1.011	0.011	0.383
403	pm14-pm27	1.018	1.031	1.027	1.003	-0.004	0.763
404	pm14-pm28	1.257	1.138	1.108	1.33	-0.049	0.159
405	pm14-pm29	0.977	1.141	0.98	1.02	-0.05	0.164
406	pm14-pm30	0.921	0.867	0.941	0.896	-0.009	0.468
407	pm14-pm31	1.032	1.119	1.021	1	0.018	0.31
408	pm14-pm32	0.993	1.165	0.999	1.031	0.007	0.502
409	pm14-pm33	0.986	1.032	1.011	1.016	0.009	0.582
410	pm14-pm34	0.993	1.036	1.011	0.994	0.002	0.86
411	pm14-pm35	0.978	0.991	1.026	0.993	-0.01	0.328
412	pm14-pm36	1.004	1.019	1.019	1.006	0.011	0.385
413	pm14-pm37	0.995	1.023	0.997	0.994	-0.002	0.745
414	pm15-pm16	1.01	1.007	0.983	0.993	-0.007	0.489

415	pm15-pm17	0.987	1.01	1.024	0.977	-0.007	0.498
416	pm15-pm18	0.966	1.018	1.038	0.983	-0.003	0.787
417	pm15-pm19	0.997	0.998	1.012	1.013	0	0.733
418	pm15-pm20	1.001	1.049	1.029	0.968	0.004	0.818
419	pm15-pm21	1.117	1.267	1.385	1.042	-0.063	0.029
420	pm15-pm22	1.059	1.115	1.106	1.007	-0.012	0.37
421	pm15-pm23	1.058	1.259	1.096	1.004	-0.023	0.268
422	pm15-pm24	1.006	1.021	1.005	1.018	0.004	0.802
423	pm15-pm25	0.979	1.055	1.016	0.989	0.005	0.735
424	pm15-pm26	1.004	1.034	1.035	1.006	0.015	0.311
425	pm15-pm27	1	1.063	1.013	1.015	-0.004	0.962
426	pm15-pm28	1.235	1.108	1.187	1.313	-0.05	0.168
427	pm15-pm29	1.005	1.109	0.97	1.076	-0.042	0.15
428	pm15-pm30	0.9	0.894	1.011	0.886	0.004	0.778
429	pm15-pm31	1.033	1.061	1.074	1.024	0.019	0.38
430	pm15-pm32	0.989	1.103	1.028	1.023	-0.007	0.523
431	pm15-pm33	0.985	1.004	1.035	1.006	0.002	0.957
432	pm15-pm34	1.002	0.991	0.984	0.978	-0.021	0.291
433	pm15-pm35	0.974	0.978	1.001	0.975	-0.028	0.237
434	pm15-pm36	0.968	1.014	1.018	1.016	-0.001	0.836
435	pm15-pm37	0.998	1.022	1.012	1.011	0.003	0.622
436	pm16-pm17	0.968	1.011	1.002	0.979	-0.011	0.367
437	pm16-pm18	1.004	0.984	1.017	0.991	0	0.972
438	pm16-pm19	1.013	0.994	1.042	1.004	0.014	0.375
439	pm16-pm20	1.006	0.997	1.01	0.975	-0.005	0.483
440	pm16-pm21	1.105	1.325	1.371	1.053	-0.047	0.201
441	pm16-pm22	1.044	1.166	1.073	1.03	-0.001	0.677
442	pm16-pm23	1.089	1.216	1.113	1.009	-0.015	0.333
443	pm16-pm24	1.008	1.032	1.055	0.971	0.013	0.318
444	pm16-pm25	1.021	0.855	1.012	1.189	0.02	0.397

445	pm16-pm26	0.938	0.853	0.794	0.996	-0.105	0.009
446	pm16-pm27	1.022	1.073	1.025	0.992	0.006	0.582
447	pm16-pm28	1.228	1.129	1.138	1.357	-0.042	0.158
448	pm16-pm29	1.046	1.174	0.938	1.051	-0.025	0.297
449	pm16-pm30	0.896	0.812	0.964	0.923	-0.014	0.331
450	pm16-pm31	0.987	1.067	1.045	0.994	0	0.963
451	pm16-pm32	0.998	1.122	1.036	1.058	0.016	0.382
452	pm16-pm33	0.991	1.032	0.984	1.014	0.005	0.481
453	pm16-pm34	1.015	0.966	1.007	0.983	-0.011	0.304
454	pm16-pm35	0.978	0.985	1.015	0.982	-0.015	0.386
455	pm16-pm36	0.968	1.003	1.012	0.984	-0.007	0.541
456	pm16-pm37	1.002	1.013	1.01	1.023	0.01	0.383
457	pm17-pm18	1	0.981	1.007	0.974	-0.009	0.503
458	pm17-pm19	0.996	1.04	1.011	0.992	0.009	0.599
459	pm17-pm20	0.984	1.015	0.99	0.982	-0.01	0.346
460	pm17-pm21	1.109	1.257	1.382	0.986	-0.078	0.015
461	pm17-pm22	1.023	1.076	1.073	0.997	-0.037	0.231
462	pm17-pm23	1.072	1.174	1.126	1	-0.03	0.296
463	pm17-pm24	1.016	1.012	1.024	0.983	0.004	0.943
464	pm17-pm25	1.022	1.002	0.991	0.995	0.002	0.792
465	pm17-pm26	0.983	1.01	0.995	0.974	-0.01	0.387
466	pm17-pm27	0.996	0.992	1.032	0.979	-0.023	0.216
467	pm17-pm28	1.217	1.083	1.175	1.353	-0.049	0.163
468	pm17-pm29	0.971	1.206	0.971	1.014	-0.037	0.235
469	pm17-pm30	0.888	0.857	0.929	0.863	-0.03	0.2
470	pm17-pm31	0.996	1.029	1.03	0.983	-0.015	0.368
471	pm17-pm32	0.979	1.175	1.011	1.006	0.004	0.771
472	pm17-pm33	0.985	0.99	1.008	0.973	-0.012	0.372
473	pm17-pm34	0.991	0.949	0.998	0.969	-0.028	0.219
474	pm17-pm35	0.992	0.95	1.015	0.978	-0.022	0.256

475	pm17-pm36	0.996	0.978	1.018	0.95	-0.014	0.392
476	pm17-pm37	0.978	1.023	1.001	0.991	-0.004	0.832
477	pm18-pm19	0.984	1.009	0.994	0.991	-0.003	0.996
478	pm18-pm20	0.987	1.009	1.008	1.012	0.003	0.622
479	pm18-pm21	1.098	1.237	1.418	1.034	-0.062	0.04
480	pm18-pm22	1.039	1.181	1.083	1.004	-0.001	0.751
481	pm18-pm23	1.102	1.227	1.129	1.064	0.01	0.563
482	pm18-pm24	0.984	1.015	0.979	1.003	-0.007	0.43
483	pm18-pm25	0.996	1.015	0.986	0.981	-0.004	0.902
484	pm18-pm26	0.981	0.993	1	1.016	-0.001	0.721
485	pm18-pm27	0.997	1.035	1.014	1.011	-0.006	0.455
486	pm18-pm28	1.233	1.11	1.108	1.406	-0.039	0.204
487	pm18-pm29	1.017	1.226	0.965	1.041	-0.013	0.307
488	pm18-pm30	0.926	0.882	0.972	0.887	0.005	0.448
489	pm18-pm31	1.019	1.016	1.033	1.021	0	0.957
490	pm18-pm32	0.997	1.159	1.002	1.023	0.009	0.466
491	pm18-pm33	0.991	1.019	0.983	0.987	-0.003	0.737
492	pm18-pm34	1.005	1.044	0.984	0.991	0.004	0.672
493	pm18-pm35	0.985	1.013	0.987	0.972	-0.014	0.354
494	pm18-pm36	1.003	0.986	0.985	1.011	-0.001	0.991
495	pm18-pm37	1.015	1.014	1.015	1.026	0.017	0.391
496	pm19-pm20	1.006	0.996	1.012	1.014	0.006	0.509
497	pm19-pm21	1.107	1.287	1.371	1.007	-0.067	0.022
498	pm19-pm22	1.04	1.156	1.099	1.038	0.005	0.559
499	pm19-pm23	1.073	1.207	1.104	1.032	-0.017	0.307
500	pm19-pm24	0.979	1.045	1.007	1.014	0.009	0.477
501	pm19-pm25	1.001	0.984	0.992	0.987	-0.008	0.582
502	pm19-pm26	0.992	0.996	1.003	0.988	-0.004	0.616
503	pm19-pm27	1.03	1.007	1.042	1.027	0.006	0.529
504	pm19-pm28	1.208	1.094	1.127	1.32	-0.067	0.042

505	pm19-pm29	1.039	1.24	1.026	1.063	0.016	0.3
506	pm19-pm30	0.917	0.88	0.969	0.895	0.003	0.688
507	pm19-pm31	1.021	1.028	1.032	0.995	-0.003	0.794
508	pm19-pm32	0.978	1.137	0.988	1.038	-0.001	0.996
509	pm19-pm33	1.006	1.017	0.99	1.014	0.008	0.451
510	pm19-pm34	0.974	1.03	1.025	0.986	0.001	0.886
511	pm19-pm35	1.036	1.014	0.981	1.009	0.006	0.583
512	pm19-pm36	1.003	0.996	1.016	1.017	0.01	0.313
513	pm19-pm37	1.014	0.979	1.008	1.03	0.007	0.404
514	pm20-pm21	1.115	1.218	1.393	1.022	-0.075	0.014
515	pm20-pm22	1.082	1.153	1.089	1.01	0.003	0.717
516	pm20-pm23	1.076	1.244	1.094	1.05	-0.008	0.58
517	pm20-pm24	1.017	1.012	0.979	1	-0.003	0.869
518	pm20-pm25	1.003	1.024	0.993	0.979	-0.002	0.744
519	pm20-pm26	0.979	0.982	1.016	0.973	-0.014	0.392
520	pm20-pm27	1.028	1.064	1.03	1.041	0.018	0.335
521	pm20-pm28	1.228	1.062	1.151	1.363	-0.056	0.035
522	pm20-pm29	1.055	1.178	1.011	1.099	0.007	0.586
523	pm20-pm30	0.906	0.889	0.964	0.874	-0.007	0.547
524	pm20-pm31	1.025	1.019	1.032	1.028	0.001	0.983
525	pm20-pm32	1.007	1.133	0.983	1.032	-0.001	0.827
526	pm20-pm33	0.984	1.028	1.003	0.987	-0.001	0.737
527	pm20-pm34	0.989	1.06	1.005	1.005	0.009	0.529
528	pm20-pm35	1.005	1.018	0.977	0.987	-0.01	0.342
529	pm20-pm36	1.013	1.019	0.981	0.984	-0.002	0.822
530	pm20-pm37	1.019	1	0.993	1.01	0.002	0.843
531	pm21-pm22	1.192	1.397	1.468	1.043	-0.064	0.044
532	pm21-pm23	1.182	1.537	1.539	1.076	-0.049	0.201
533	pm21-pm24	1.131	1.305	1.392	1.021	-0.051	0.037
534	pm21-pm25	1.133	1.306	1.382	1.012	-0.052	0.039

535	pm21-pm26	1.083	1.267	1.383	1.023	-0.071	0.015
536	pm21-pm27	1.081	1.26	1.388	1.037	-0.09	0.015
537	pm21-pm28	1.382	1.337	1.517	1.38	-0.112	0.007
538	pm21-pm29	1.119	1.495	1.409	1.103	-0.056	0.022
539	pm21-pm30	1.036	1.169	1.344	0.91	-0.059	0.043
540	pm21-pm31	1.154	1.346	1.367	1.045	-0.056	0.039
541	pm21-pm32	1.104	1.437	1.361	1.051	-0.059	0.025
542	pm21-pm33	1.149	1.266	1.392	1.03	-0.051	0.033
543	pm21-pm34	1.112	1.295	1.348	0.996	-0.076	0.012
544	pm21-pm35	1.136	1.29	1.395	1.062	-0.044	0.163
545	pm21-pm36	1.1	1.28	1.378	1.019	-0.065	0.025
546	pm21-pm37	1.133	1.316	1.339	1.008	-0.063	0.035
547	pm22-pm23	1.384	1.546	1.414	1.037	0.145	0.009
548	pm22-pm24	1.051	1.229	1.102	1.019	0.018	0.373
549	pm22-pm25	1.045	1.103	1.133	1.011	-0.005	0.511
550	pm22-pm26	1.038	1.17	1.114	1.014	0.006	0.464
551	pm22-pm27	1.034	1.195	1.112	1.027	-0.008	0.474
552	pm22-pm28	1.304	1.246	1.287	1.42	-0.02	0.375
553	pm22-pm29	1.107	1.277	1.053	1.059	-0.031	0.233
554	pm22-pm30	0.939	1.012	1.042	0.934	-0.01	0.485
555	pm22-pm31	1.048	1.21	1.104	1.028	-0.004	0.689
556	pm22-pm32	1.054	1.271	1.068	1.066	-0.002	0.86
557	pm22-pm33	1.058	1.149	1.065	1.006	-0.009	0.486
558	pm22-pm34	1.061	1.168	1.074	1.03	0.001	0.654
559	pm22-pm35	1.039	1.097	1.083	1.027	-0.022	0.247
560	pm22-pm36	1.037	1.15	1.112	1.006	-0.001	0.624
561	pm22-pm37	1.052	1.126	1.129	1.017	0.001	0.997
562	pm23-pm24	1.125	1.271	1.092	1.036	0.006	0.481
563	pm23-pm25	1.066	1.272	1.089	1.009	-0.012	0.393
564	pm23-pm26	1.121	1.279	1.1	1.035	0.012	0.323

565	pm23-pm27	1.112	1.263	1.137	1.036	-0.006	0.495
566	pm23-pm28	1.349	1.398	1.244	1.39	-0.032	0.243
567	pm23-pm29	1.073	1.417	1.054	1.085	-0.041	0.157
568	pm23-pm30	0.961	1.114	1.051	0.917	-0.024	0.237
569	pm23-pm31	1.083	1.292	1.106	1.055	-0.011	0.39
570	pm23-pm32	1.09	1.382	1.106	1.09	0.008	0.485
571	pm23-pm33	1.06	1.257	1.117	1.009	-0.011	0.32
572	pm23-pm34	1.045	1.279	1.091	1.028	-0.015	0.362
573	pm23-pm35	1.061	1.231	1.098	1.017	-0.025	0.3
574	pm23-pm36	1.11	1.214	1.124	1.053	0.005	0.943
575	pm23-pm37	1.053	1.297	1.131	1.05	0.009	0.42
576	pm24-pm25	1.017	0.976	1.011	1.002	-0.001	0.753
577	pm24-pm26	0.997	1.062	0.987	1.011	0.011	0.349
578	pm24-pm27	0.988	1.078	1.037	0.981	-0.004	0.713
579	pm24-pm28	1.24	1.125	1.176	1.327	-0.042	0.181
580	pm24-pm29	1.01	1.158	1.031	1.035	-0.022	0.294
581	pm24-pm30	0.919	0.913	0.96	0.88	0.002	0.952
582	pm24-pm31	1.017	1.057	1.044	0.996	0.002	0.684
583	pm24-pm32	0.983	1.145	1.014	1.031	0.002	0.707
584	pm24-pm33	0.968	0.993	0.995	0.995	-0.016	0.389
585	pm24-pm34	1.01	1.048	0.989	1.009	0.007	0.555
586	pm24-pm35	1.021	1	1.015	0.972	-0.006	0.518
587	pm24-pm36	0.974	1	0.992	0.994	-0.012	0.36
588	pm24-pm37	1.005	1.009	1.021	1.003	0.004	0.841
589	pm25-pm26	1.001	0.98	1.016	1.015	0.004	0.653
590	pm25-pm27	1.011	1.007	1.04	0.998	-0.007	0.498
591	pm25-pm28	1.233	1.044	1.119	1.339	-0.071	0.013
592	pm25-pm29	0.996	1.179	1.026	1.032	-0.018	0.329
593	pm25-pm30	0.95	0.862	0.936	0.924	0.006	0.555
594	pm25-pm31	1.012	1.051	1.043	1.012	0.007	0.551

595	pm25-pm32	0.992	1.127	1.022	1.051	0.011	0.333
596	pm25-pm33	0.989	0.995	1.012	1.002	0	0.975
597	pm25-pm34	1.02	1.017	1.021	1.005	0.013	0.324
598	pm25-pm35	1.011	0.994	1.015	0.97	-0.007	0.448
599	pm25-pm36	0.982	0.98	0.98	1.011	-0.01	0.317
600	pm25-pm37	0.998	1	0.983	0.998	-0.006	0.584
601	pm26-pm27	1.014	1.035	1.045	1.004	0.004	0.783
602	pm26-pm28	1.242	1.071	1.11	1.383	-0.054	0.029
603	pm26-pm29	1.006	1.179	0.979	1.067	-0.018	0.361
604	pm26-pm30	0.928	0.914	0.945	0.9	0.009	0.567
605	pm26-pm31	1.029	1.029	1.046	1.024	0.009	0.514
606	pm26-pm32	0.989	1.094	1.024	1.054	0.003	0.952
607	pm26-pm33	0.992	1.004	1.003	1.02	0.005	0.528
608	pm26-pm34	1.006	1.028	0.996	1.02	0.009	0.415
609	pm26-pm35	0.997	1.04	1.002	0.995	0.004	0.9
610	pm26-pm36	1.002	1.013	1.007	1.006	0.009	0.556
611	pm26-pm37	0.98	1.016	0.992	1.016	0	0.817
612	pm27-pm28	1.231	1.151	1.148	1.377	-0.05	0.178
613	pm27-pm29	1.026	1.23	1.029	1.054	-0.013	0.335
614	pm27-pm30	0.956	0.928	0.984	0.907	0.01	0.595
615	pm27-pm31	1.022	1.119	1.057	1.028	0.012	0.305
616	pm27-pm32	0.994	1.166	1.019	1.036	-0.005	0.555
617	pm27-pm33	1.016	1.033	0.995	1.022	-0.005	0.821
618	pm27-pm34	1.036	1.052	1.052	1.004	0.011	0.375
619	pm27-pm35	1.011	1.085	1.043	0.998	0.008	0.444
620	pm27-pm36	1.003	1.069	1.014	0.998	0.001	0.944
621	pm27-pm37	1.001	1.066	1.026	1.036	0.009	0.467
622	pm28-pm29	1.332	1.228	1.122	1.48	-0.041	0.161
623	pm28-pm30	1.156	0.989	1.082	1.238	-0.052	0.032
624	pm28-pm31	1.264	1.086	1.162	1.39	-0.053	0.043

625	pm28-pm32	1.227	1.173	1.13	1.396	-0.061	0.039
626	pm28-pm33	1.204	1.016	1.136	1.347	-0.079	0.014
627	pm28-pm34	1.245	1.1	1.121	1.361	-0.052	0.038
628	pm28-pm35	1.263	1.107	1.153	1.328	-0.047	0.19
629	pm28-pm36	1.224	1.068	1.155	1.309	-0.065	0.035
630	pm28-pm37	1.209	1.061	1.14	1.343	-0.069	0.037
631	pm29-pm30	1.001	1.296	0.781	0.927	0.012	0.375
632	pm29-pm31	1.04	1.257	1.003	1.072	-0.007	0.405
633	pm29-pm32	1.054	1.343	0.983	1.08	0.001	0.671
634	pm29-pm33	0.967	1.15	0.99	1.027	-0.043	0.193
635	pm29-pm34	1.022	1.123	0.985	1.047	-0.036	0.26
636	pm29-pm35	1.012	1.093	0.971	1.079	-0.043	0.189
637	pm29-pm36	1.017	1.181	1	1.07	-0.008	0.591
638	pm29-pm37	1.011	1.193	1.008	1.039	-0.016	0.309
639	pm30-pm31	0.925	0.974	0.984	0.901	0.01	0.343
640	pm30-pm32	0.92	0.992	0.99	0.929	0.008	0.432
641	pm30-pm33	0.954	0.913	0.969	0.909	0.023	0.258
642	pm30-pm34	0.887	0.924	0.971	0.892	0.002	0.786
643	pm30-pm35	0.946	0.905	0.961	0.909	0.012	0.36
644	pm30-pm36	0.956	0.875	0.95	0.935	0.017	0.392
645	pm30-pm37	0.967	0.876	0.951	0.921	0.014	0.379
646	pm31-pm32	1.01	1.143	1.034	1.046	-0.002	0.816
647	pm31-pm33	1.01	0.999	1.04	1.018	-0.006	0.471
648	pm31-pm34	1.032	1.069	1.026	1.007	0.006	0.592
649	pm31-pm35	1.007	1.07	1.026	1.012	0	0.796
650	pm31-pm36	0.996	1.036	1.028	1.029	0	0.657
651	pm31-pm37	1.006	1.062	1.046	0.992	0.002	0.832
652	pm32-pm33	0.992	1.155	1.006	1.025	0.007	0.413
653	pm32-pm34	1.028	1.188	1.021	1.03	0.025	0.237
654	pm32-pm35	1.026	1.113	0.992	1.04	0	0.666

655	pm32-pm36	0.991	1.129	0.985	1.043	0.001	0.79
656	pm32-pm37	1.019	1.167	1.017	1.002	0.012	0.396
657	pm33-pm34	0.986	1.173	0.989	0.99	0.031	0.28
658	pm33-pm35	0.998	1.043	1.004	0.976	0.001	0.681
659	pm33-pm36	0.981	0.999	0.998	0.991	-0.006	0.527
660	pm33-pm37	1.012	0.979	0.997	0.999	-0.005	0.959
661	pm34-pm35	1.02	0.991	1.036	0.993	0.002	0.673
662	pm34-pm36	1.029	0.994	1.016	0.978	0.002	0.787
663	pm34-pm37	1.006	1.045	1.032	1.027	0.022	0.221
664	pm35-pm36	1.017	1.034	0.986	0.989	0.003	0.864
665	pm35-pm37	0.991	0.997	1.002	1.013	-0.006	0.592
666	pm36-pm37	1.019	0.993	0.981	0.988	-0.005	0.575

3b) Result of two-distribution perturbation analysis for apoptosis when each response function was separately applied (10 million parameter sets)

No.	Pair of perturbed parameter distributions	Lin	Hill	Sat	Acc	Synergistic effect	p-value
1	pm1-pm2	1.048	1.052	1.026	1.047	0.03	0.272
2	pm1-pm3	1.034	1.082	1.05	1.04	0.03	0.294
3	pm1-pm4	1.034	1.062	1.008	1.023	-0.01	0.356
4	pm1-pm5	1.011	0.992	1.013	0.997	-0.01	0.362
5	pm1-pm6	1.062	1.043	1.14	1.057	0.023	0.248
6	pm1-pm7	1.949	1.617	2.101	1.595	-0.104	0.008
7	pm1-pm8	1.033	1.045	1.024	1.019	0.017	0.369
8	pm1-pm9	1.073	1.108	1.057	1.059	0.024	0.287
9	pm1-pm10	2.249	1.902	2.079	2.235	0.082	0.012
10	pm1-pm11	1.022	1.053	0.993	1.039	0.006	0.596

11	pm1-pm12	1.125	1.235	1.063	1.244	-0.032	0.247
12	pm1-pm13	0.982	1.051	0.996	0.983	-0.021	0.21
13	pm1-pm14	1.008	1.085	0.994	1.022	0.009	0.493
14	pm1-pm15	1.033	1.066	1.044	1.015	0.018	0.314
15	pm1-pm16	0.994	1.08	1.028	1.033	0.018	0.354
16	pm1-pm17	0.988	1.025	1.013	0.995	-0.011	0.31
17	pm1-pm18	1.031	1	1.028	1.023	0.006	0.49
18	pm1-pm19	1.012	1.004	1.032	1.02	0.002	0.972
19	pm1-pm20	1.027	1.027	1.016	1.029	0.007	0.59
20	pm1-pm21	1.172	1.305	1.4	1.037	-0.048	0.161
21	pm1-pm22	1.089	1.201	1.109	1.064	0.021	0.212
22	pm1-pm23	1.135	1.241	1.145	1.067	0.009	0.58
23	pm1-pm24	1.019	1.082	1.001	1.018	0.011	0.373
24	pm1-pm25	1.028	1.036	1.02	1.019	0.011	0.353
25	pm1-pm26	0.999	1.045	0.981	1.002	-0.009	0.576
26	pm1-pm27	1.01	1.077	1.018	1.013	-0.007	0.527
27	pm1-pm28	1.248	1.11	1.132	1.372	-0.056	0.035
28	pm1-pm29	1.026	1.209	1.007	1.092	-0.009	0.447
29	pm1-pm30	0.946	0.954	0.974	0.912	0.018	0.391
30	pm1-pm31	1.035	1.064	1.034	1.033	0.003	0.799
31	pm1-pm32	1.021	1.186	1.051	1.04	0.022	0.287
32	pm1-pm33	1.002	1.065	1.019	1.009	0.008	0.505
33	pm1-pm34	1.039	1.102	1.009	0.995	0.017	0.309
34	pm1-pm35	1.024	1.097	1.002	1.035	0.019	0.361
35	pm1-pm36	1.003	1.055	1.029	1.053	0.02	0.23
36	pm1-pm37	1.006	1.049	0.994	1.02	0	0.817
37	pm2-pm3	1.015	1.022	1.02	0.999	0.01	0.318
38	pm2-pm4	1.007	1.054	1.04	0.984	-0.003	0.998
39	pm2-pm5	0.98	0.989	0.959	1.028	-0.007	0.401
40	pm2-pm6	1.013	0.982	1.093	1.015	-0.009	0.463

41	pm2-pm7	1.973	1.555	2.079	1.615	-0.096	0.012
42	pm2-pm8	0.996	1.022	0.987	0.985	0.002	0.941
43	pm2-pm9	1.015	1.044	1.024	1.001	-0.011	0.374
44	pm2-pm10	2.259	1.895	2.144	2.299	0.132	0.009
45	pm2-pm11	0.982	0.991	0.992	0.998	-0.012	0.31
46	pm2-pm12	1.109	1.154	1.027	1.172	-0.066	0.024
47	pm2-pm13	0.974	1.054	1.011	1.004	0.005	0.732
48	pm2-pm14	0.968	1.031	1.004	0.989	-0.003	0.979
49	pm2-pm15	1.004	1.042	0.991	1.002	0.006	0.44
50	pm2-pm16	1.029	1.025	0.993	1.027	0.021	0.252
51	pm2-pm17	0.988	1.03	1.018	0.98	0.005	0.582
52	pm2-pm18	0.998	1.017	0.979	0.998	0.001	0.908
53	pm2-pm19	0.994	1.004	1.016	0.988	0.004	0.908
54	pm2-pm20	1.011	1.043	1.01	0.98	0.011	0.33
55	pm2-pm21	1.123	1.275	1.383	1.015	-0.059	0.022
56	pm2-pm22	1.055	1.134	1.076	1.009	-0.008	0.449
57	pm2-pm23	1.064	1.231	1.119	1.068	0.001	0.909
58	pm2-pm24	1	1.04	0.997	0.998	0.007	0.457
59	pm2-pm25	0.973	0.964	0.996	1.003	-0.013	0.327
60	pm2-pm26	1.009	0.999	1	0.984	0.001	0.656
61	pm2-pm27	1.018	1.036	1.044	1.027	0.012	0.372
62	pm2-pm28	1.224	1.061	1.121	1.329	-0.069	0.024
63	pm2-pm29	1.016	1.215	1.002	1.072	0.002	0.811
64	pm2-pm30	0.937	0.921	0.973	0.876	0.016	0.352
65	pm2-pm31	0.978	1.018	1.018	0.992	-0.02	0.321
66	pm2-pm32	1.003	1.133	1.014	1.011	0.005	0.867
67	pm2-pm33	0.983	1.015	1.009	1.015	0.008	0.479
68	pm2-pm34	0.961	1.034	1.013	1.016	0.004	0.882
69	pm2-pm35	1.029	1.027	1.003	1.016	0.016	0.398
70	pm2-pm36	1.009	0.992	1.013	1.001	0.007	0.581

71	pm2-pm37	1.018	1.018	1.014	1.025	0.019	0.367
72	pm3-pm4	1.01	1.043	1	1.001	-0.018	0.39
73	pm3-pm5	0.994	1.008	1.045	1.026	0.014	0.378
74	pm3-pm6	1.031	1.02	1.073	1.029	-0.004	0.688
75	pm3-pm7	1.944	1.571	2.092	1.598	-0.109	0.009
76	pm3-pm8	1.005	1.032	1.024	0.997	0.012	0.358
77	pm3-pm9	1.033	1.016	1.063	0.999	-0.012	0.342
78	pm3-pm10	2.316	1.944	2.472	2.048	0.17	0.002
79	pm3-pm11	1.015	1.025	1.029	0.991	0.005	0.951
80	pm3-pm12	1.08	1.211	1.039	1.201	-0.056	0.034
81	pm3-pm13	1	1.058	1.002	0.99	-0.001	0.894
82	pm3-pm14	1.002	1.047	1.002	1.007	0.006	0.444
83	pm3-pm15	0.995	1.019	0.983	0.994	-0.013	0.321
84	pm3-pm16	0.988	1.022	1.012	0.993	-0.002	0.843
85	pm3-pm17	0.99	0.988	1.033	0.983	-0.008	0.447
86	pm3-pm18	1.017	1.018	0.99	1.016	0.006	0.407
87	pm3-pm19	0.999	1.017	1.004	0.988	-0.003	0.989
88	pm3-pm20	0.997	1.057	0.992	0.993	0.002	0.878
89	pm3-pm21	1.113	1.283	1.335	1.01	-0.081	0.017
90	pm3-pm22	1.085	1.19	1.062	1.03	0.007	0.409
91	pm3-pm23	1.074	1.215	1.122	1.038	-0.015	0.373
92	pm3-pm24	1.016	1.044	1	1.011	0.009	0.476
93	pm3-pm25	0.989	1.029	1.002	1.002	0	0.865
94	pm3-pm26	1.008	1.042	1.031	1.017	0.019	0.325
95	pm3-pm27	1.005	1.047	1.041	0.996	-0.005	0.716
96	pm3-pm28	1.201	1.054	1.132	1.346	-0.078	0.019
97	pm3-pm29	0.975	1.198	1.026	1.018	-0.028	0.228
98	pm3-pm30	0.899	0.882	0.963	0.893	-0.009	0.507
99	pm3-pm31	1.014	1.05	1.019	1.002	-0.008	0.434
100	pm3-pm32	0.997	1.136	1.032	1.035	0.006	0.484

101	pm3-pm33	0.984	1.001	0.995	0.995	-0.011	0.355
102	pm3-pm34	1.02	1.074	0.995	0.976	0.007	0.421
103	pm3-pm35	0.998	1.005	1.012	0.966	-0.015	0.355
104	pm3-pm36	0.989	0.965	1.006	0.997	-0.015	0.36
105	pm3-pm37	0.999	0.993	1.011	1.004	-0.006	0.548
106	pm4-pm5	1.002	1.045	0.977	1.024	-0.012	0.374
107	pm4-pm6	1.018	1.006	1.073	1.001	-0.038	0.294
108	pm4-pm7	1.915	1.605	2.076	1.619	-0.126	0.007
109	pm4-pm8	0.996	1.069	1.017	1.015	0.001	0.891
110	pm4-pm9	1.017	1.087	1.03	1.045	-0.015	0.319
111	pm4-pm10	2.1	1.875	1.999	2.151	-0.014	0.322
112	pm4-pm11	0.99	1.052	1.035	1.001	-0.012	0.357
113	pm4-pm12	1.095	1.23	1.066	1.219	-0.057	0.039
114	pm4-pm13	0.974	1.039	1.029	0.992	-0.025	0.217
115	pm4-pm14	1.032	1.079	1.043	0.991	0.008	0.514
116	pm4-pm15	1.002	1.081	1.06	1.013	0.008	0.505
117	pm4-pm16	1.037	1.084	1.033	0.999	0.012	0.319
118	pm4-pm17	0.99	1.079	1.009	0.971	-0.015	0.321
119	pm4-pm18	1.018	1.015	0.988	0.981	-0.024	0.265
120	pm4-pm19	1.02	1.002	1.016	0.987	-0.019	0.338
121	pm4-pm20	1.009	1.086	1.028	0.992	0.001	0.62
122	pm4-pm21	1.139	1.312	1.419	1.012	-0.066	0.025
123	pm4-pm22	1.057	1.237	1.13	1.034	0.01	0.304
124	pm4-pm23	1.141	1.41	1.141	1.032	0.033	0.294
125	pm4-pm24	0.986	1.051	1.03	0.973	-0.019	0.379
126	pm4-pm25	1.01	1.048	0.992	0.999	-0.013	0.348
127	pm4-pm26	1.022	1.075	1.006	0.969	-0.007	0.568
128	pm4-pm27	1.05	1.04	1.041	1.014	-0.011	0.331
129	pm4-pm28	1.252	1.167	1.14	1.313	-0.063	0.04
130	pm4-pm29	1.017	1.33	1	1.116	0.013	0.33

131	pm4-pm30	0.953	0.962	0.978	0.893	0.008	0.471
132	pm4-pm31	0.987	1.064	1.054	1.026	-0.017	0.351
133	pm4-pm32	1.006	1.131	1.013	1.036	-0.017	0.324
134	pm4-pm33	1.006	1.053	1.03	1.006	-0.002	0.716
135	pm4-pm34	1.006	1.106	1.04	0.977	0.002	0.872
136	pm4-pm35	0.996	1.044	0.973	0.97	-0.035	0.294
137	pm4-pm36	1.008	1.051	1.029	1.021	0.003	0.959
138	pm4-pm37	0.998	1.04	1.01	0.986	-0.019	0.36
139	pm5-pm6	1.059	1.001	1.098	1.007	0.007	0.459
140	pm5-pm7	1.946	1.573	2.09	1.57	-0.107	0.008
141	pm5-pm8	1.003	0.976	0.978	1	-0.006	0.42
142	pm5-pm9	0.991	1.032	1.014	1.046	-0.012	0.389
143	pm5-pm10	2.147	1.704	1.967	2.185	-0.016	0.359
144	pm5-pm11	1.013	0.99	0.975	1.017	-0.004	0.964
145	pm5-pm12	1.125	1.188	1.018	1.245	-0.038	0.277
146	pm5-pm13	0.976	1.008	0.996	0.965	-0.02	0.333
147	pm5-pm14	1.014	1.039	1.043	0.987	0.02	0.215
148	pm5-pm15	1.007	1.015	1.001	1	0.002	0.678
149	pm5-pm16	0.997	0.978	1.002	0.987	-0.007	0.455
150	pm5-pm17	1.039	0.993	0.977	0.989	0	0.677
151	pm5-pm18	1.005	0.974	0.977	0.981	-0.013	0.358
152	pm5-pm19	0.998	0.983	0.991	1.015	-0.001	0.847
153	pm5-pm20	1.002	0.987	0.986	1.007	-0.005	0.779
154	pm5-pm21	1.122	1.291	1.366	1.015	-0.06	0.022
155	pm5-pm22	1.07	1.103	1.095	1.017	-0.006	0.515
156	pm5-pm23	1.087	1.207	1.121	1.072	0.002	0.717
157	pm5-pm24	0.997	0.993	0.976	1.023	-0.004	0.844
158	pm5-pm25	0.983	0.979	0.975	0.991	-0.016	0.349
159	pm5-pm26	0.998	0.972	0.989	0.981	-0.013	0.314
160	pm5-pm27	1.001	0.988	1.042	0.998	-0.012	0.331

161	pm5-pm28	1.204	1.025	1.12	1.373	-0.073	0.015
162	pm5-pm29	0.982	1.174	0.958	1.098	-0.022	0.222
163	pm5-pm30	0.916	0.864	0.998	0.899	0.008	0.496
164	pm5-pm31	1.008	1.033	1.024	1.019	0	0.733
165	pm5-pm32	1.008	1.143	0.987	1.03	0.007	0.491
166	pm5-pm33	0.991	0.978	0.988	0.995	-0.01	0.439
167	pm5-pm34	1.013	1.03	1.007	0.968	0.003	0.78
168	pm5-pm35	0.986	1.027	0.98	1.009	-0.002	0.898
169	pm5-pm36	1.002	0.997	0.989	0.996	-0.001	0.913
170	pm5-pm37	0.984	0.996	1.012	1.03	0.006	0.476
171	pm6-pm7	1.985	1.567	2.266	1.627	-0.079	0.011
172	pm6-pm8	1.018	1.038	1.107	1.004	0.008	0.54
173	pm6-pm9	1.058	1.028	1.143	1.044	-0.002	0.836
174	pm6-pm10	2.281	1.967	2.291	2.352	0.167	0.002
175	pm6-pm11	1.036	1	1.095	1.012	-0.006	0.589
176	pm6-pm12	1.137	1.203	1.145	1.243	-0.038	0.217
177	pm6-pm13	1.017	1.026	1.076	1.021	-0.009	0.513
178	pm6-pm14	1.033	1.066	1.11	1.013	0.017	0.308
179	pm6-pm15	1.017	0.998	1.096	1.024	-0.008	0.428
180	pm6-pm16	1.033	1.011	1.116	1.009	0.006	0.521
181	pm6-pm17	1.021	1.01	1.093	1.006	-0.005	0.604
182	pm6-pm18	1.05	0.996	1.105	1.014	0.006	0.452
183	pm6-pm19	1.017	1.042	1.107	1.013	0.009	0.481
184	pm6-pm20	1.054	1.012	1.109	1.018	0.01	0.498
185	pm6-pm21	1.146	1.328	1.525	1.054	-0.033	0.298
186	pm6-pm22	1.092	1.129	1.169	1.016	-0.014	0.362
187	pm6-pm23	1.085	1.223	1.218	1.042	-0.016	0.342
188	pm6-pm24	1.032	1.025	1.075	1.014	-0.003	0.85
189	pm6-pm25	1.03	1.013	1.106	1.013	0.005	0.653
190	pm6-pm26	1.038	0.997	1.082	1.005	-0.005	0.518

191	pm6-pm27	1.061	1.054	1.142	1.028	0.014	0.327
192	pm6-pm28	1.297	1.117	1.205	1.304	-0.061	0.026
193	pm6-pm29	1.023	1.152	1.111	1.114	-0.013	0.372
194	pm6-pm30	0.93	0.931	1.058	0.895	0.004	0.737
195	pm6-pm31	1.056	1.049	1.106	1.041	0.003	0.948
196	pm6-pm32	1.033	1.148	1.098	1.078	0.015	0.316
197	pm6-pm33	1.022	1.032	1.128	1.024	0.015	0.356
198	pm6-pm34	1.041	1.044	1.13	0.996	0.013	0.38
199	pm6-pm35	1.03	1.017	1.08	1.028	-0.003	0.843
200	pm6-pm36	1.038	1.015	1.106	1.03	0.012	0.362
201	pm6-pm37	1.033	1.015	1.127	1.015	0.009	0.494
202	pm7-pm8	1.986	1.642	2.085	1.613	-0.07	0.016
203	pm7-pm9	2.015	1.578	2.133	1.663	-0.091	0.011
204	pm7-pm10	3.979	2.552	3.882	3.334	0.514	<0.001
205	pm7-pm11	1.941	1.636	2.086	1.652	-0.08	0.014
206	pm7-pm12	2.152	1.786	2.18	1.949	-0.071	0.018
207	pm7-pm13	1.953	1.579	2.079	1.585	-0.113	0.008
208	pm7-pm14	2.005	1.552	2.096	1.585	-0.097	0.018
209	pm7-pm15	1.968	1.608	2.049	1.584	-0.107	0.007
210	pm7-pm16	1.995	1.583	2.083	1.615	-0.085	0.01
211	pm7-pm17	1.986	1.521	2.114	1.606	-0.098	0.011
212	pm7-pm18	1.957	1.518	2.043	1.552	-0.136	0.008
213	pm7-pm19	1.945	1.54	2.13	1.596	-0.101	0.009
214	pm7-pm20	1.965	1.588	2.075	1.609	-0.097	0.01
215	pm7-pm21	2.177	1.948	2.795	1.619	-0.03	0.266
216	pm7-pm22	2.083	1.731	2.255	1.661	-0.05	0.041
217	pm7-pm23	2.049	1.832	2.231	1.672	-0.08	0.019
218	pm7-pm24	1.99	1.591	2.114	1.583	-0.088	0.016
219	pm7-pm25	1.954	1.522	2.102	1.62	-0.104	0.008
220	pm7-pm26	1.975	1.617	2.087	1.575	-0.09	0.016

221	pm7-pm27	1.98	1.546	2.129	1.612	-0.109	0.008
222	pm7-pm28	2.433	1.614	2.329	2.178	-0.021	0.201
223	pm7-pm29	2.08	1.849	2.054	1.683	-0.065	0.022
224	pm7-pm30	2.108	1.707	2.35	1.44	0.085	0.015
225	pm7-pm31	2.015	1.677	2.136	1.605	-0.069	0.041
226	pm7-pm32	1.939	1.626	2.045	1.649	-0.127	0.009
227	pm7-pm33	1.964	1.538	2.065	1.604	-0.111	0.009
228	pm7-pm34	1.965	1.593	2.099	1.594	-0.095	0.012
229	pm7-pm35	1.985	1.519	2.106	1.575	-0.113	0.007
230	pm7-pm36	1.956	1.547	2.084	1.581	-0.111	0.008
231	pm7-pm37	1.983	1.557	2.073	1.586	-0.106	0.006
232	pm8-pm9	1.001	1.065	1.015	1.001	-0.011	0.327
233	pm8-pm10	2.286	1.979	2.161	2.033	0.099	0.01
234	pm8-pm11	1.024	0.981	0.988	0.995	-0.005	0.558
235	pm8-pm12	1.104	1.155	1.05	1.177	-0.059	0.034
236	pm8-pm13	1.008	1.01	0.987	0.978	-0.009	0.494
237	pm8-pm14	0.985	1.055	1.016	1.015	0.018	0.319
238	pm8-pm15	1.026	0.994	1.002	1.027	0.009	0.469
239	pm8-pm16	1.002	1.014	1.024	0.99	0.01	0.326
240	pm8-pm17	1.013	1.015	0.992	0.984	0.003	0.706
241	pm8-pm18	0.991	0.991	0.981	0.983	-0.01	0.514
242	pm8-pm19	0.982	0.978	0.996	0.996	-0.008	0.451
243	pm8-pm20	0.991	1.001	0.988	0.983	-0.009	0.577
244	pm8-pm21	1.111	1.256	1.371	1.028	-0.066	0.027
245	pm8-pm22	1.076	1.14	1.073	1.024	0.002	0.688
246	pm8-pm23	1.072	1.252	1.105	1.041	-0.002	0.656
247	pm8-pm24	1.004	0.992	0.989	0.977	-0.01	0.312
248	pm8-pm25	1.007	1.019	1.003	1.005	0.012	0.327
249	pm8-pm26	1.004	0.99	0.993	1.012	0.003	0.677
250	pm8-pm27	1.003	1.044	1.017	1.007	-0.001	0.75

251	pm8-pm28	1.229	1.094	1.081	1.346	-0.065	0.045
252	pm8-pm29	1.027	1.161	0.995	1.109	-0.001	0.7
253	pm8-pm30	0.953	0.881	0.963	0.905	0.015	0.321
254	pm8-pm31	1.021	1.038	1.019	1.018	0.003	0.935
255	pm8-pm32	0.985	1.112	1.022	1.024	0.001	0.87
256	pm8-pm33	1.023	0.998	0.987	0.992	0.003	0.743
257	pm8-pm34	0.971	1.015	1	0.983	-0.009	0.535
258	pm8-pm35	1.02	1.034	0.99	1.009	0.011	0.358
259	pm8-pm36	0.983	1.017	0.993	1.003	0.003	0.732
260	pm8-pm37	0.993	0.988	1.003	0.989	-0.006	0.512
261	pm9-pm10	2.3	2.005	2.199	2.059	0.088	0.013
262	pm9-pm11	1.037	1.025	1.023	1.04	-0.008	0.525
263	pm9-pm12	1.173	1.182	1.045	1.269	-0.05	0.039
264	pm9-pm13	1.019	1.018	1.039	0.994	-0.025	0.283
265	pm9-pm14	1.023	1.078	1.032	1.029	0.004	0.842
266	pm9-pm15	1.026	1.045	1.081	1.017	0.002	0.862
267	pm9-pm16	1.042	1.04	1.009	1.044	-0.001	0.933
268	pm9-pm17	1.034	1.041	1.061	1.003	-0.001	0.616
269	pm9-pm18	1.009	1.002	1.009	0.996	-0.029	0.264
270	pm9-pm19	1.019	1.042	1.012	1.019	-0.01	0.335
271	pm9-pm20	1.01	1.037	1.036	1.035	-0.007	0.466
272	pm9-pm21	1.147	1.344	1.434	1.031	-0.056	0.021
273	pm9-pm22	1.105	1.201	1.127	1.037	0.005	0.627
274	pm9-pm23	1.083	1.269	1.107	1.055	-0.027	0.262
275	pm9-pm24	1.051	1.048	1.002	1.006	-0.011	0.33
276	pm9-pm25	1.043	1.043	1.037	1.046	0.009	0.455
277	pm9-pm26	1.018	1.062	1.004	1.019	-0.008	0.408
278	pm9-pm27	1.046	1.082	1.039	1.02	-0.009	0.474
279	pm9-pm28	1.272	1.123	1.171	1.383	-0.053	0.04
280	pm9-pm29	1.04	1.178	1.014	1.082	-0.032	0.269

281	pm9-pm30	0.951	0.957	1.004	0.904	0.007	0.552
282	pm9-pm31	1.046	1.1	1.07	1.054	0.01	0.381
283	pm9-pm32	1.019	1.153	1.044	1.065	-0.002	0.962
284	pm9-pm33	1.003	1.072	1.044	1.025	0.002	0.892
285	pm9-pm34	1.04	1.054	1	1.017	-0.01	0.325
286	pm9-pm35	1.023	1.089	1.016	0.996	-0.008	0.431
287	pm9-pm36	1.023	1.041	1.058	1.026	0.004	0.694
288	pm9-pm37	1.036	1.066	1.018	1.01	-0.003	0.817
289	pm10-pm11	2.331	1.929	2.154	2.105	0.106	0.007
290	pm10-pm12	2.193	1.975	1.925	2.436	-0.07	0.016
291	pm10-pm13	2.301	2.006	2.138	2.005	0.086	0.018
292	pm10-pm14	2.242	1.856	2.062	2.246	0.08	0.014
293	pm10-pm15	2.094	1.91	2.099	2.246	0.063	0.028
294	pm10-pm16	1.989	1.758	1.853	2.022	-0.113	0.006
295	pm10-pm17	2.297	1.969	2.121	2.042	0.087	0.019
296	pm10-pm18	1.988	1.717	1.888	2.073	-0.101	0.006
297	pm10-pm19	2.16	1.891	2.079	2.272	0.082	0.017
298	pm10-pm20	2.307	2.005	2.155	2.052	0.108	0.008
299	pm10-pm21	2.202	2.14	2.56	2.061	-0.039	0.297
300	pm10-pm22	2.041	1.902	1.964	2.104	-0.095	0.018
301	pm10-pm23	2.142	1.941	2.038	2.126	-0.079	0.018
302	pm10-pm24	2.098	1.831	1.965	2.108	-0.022	0.237
303	pm10-pm25	2.108	1.766	2.004	2.127	-0.017	0.378
304	pm10-pm26	2.306	1.94	2.177	2.049	0.099	0.018
305	pm10-pm27	2.053	1.747	1.88	2.065	-0.104	0.009
306	pm10-pm28	2.408	1.732	2.049	2.745	-0.041	0.19
307	pm10-pm29	2.01	1.939	1.889	2.204	-0.085	0.016
308	pm10-pm30	2.119	1.883	2.074	2.126	0.119	0.008
309	pm10-pm31	2.051	1.693	1.875	2.084	-0.116	0.006
310	pm10-pm32	2.201	1.953	2.084	2.325	0.085	0.011

311	pm10-pm33	2.273	1.943	2.193	2.021	0.089	0.015
312	pm10-pm34	2.303	2.025	2.127	2.066	0.108	0.01
313	pm10-pm35	2.279	1.984	2.128	2.042	0.084	0.01
314	pm10-pm36	2.243	1.987	2.148	2.027	0.083	0.016
315	pm10-pm37	2.295	2.001	2.178	2.007	0.1	0.02
316	pm11-pm12	1.107	1.156	1.029	1.254	-0.052	0.03
317	pm11-pm13	0.999	1.053	1.04	0.986	0.007	0.473
318	pm11-pm14	1.023	1.031	1.042	0.993	0.015	0.337
319	pm11-pm15	1.008	1.039	1.011	1.014	0.008	0.577
320	pm11-pm16	1.025	1.041	1.027	1.047	0.03	0.235
321	pm11-pm17	0.977	1.001	0.997	0.954	-0.024	0.26
322	pm11-pm18	0.982	0.963	0.991	1.019	-0.015	0.371
323	pm11-pm19	0.971	0.976	0.978	0.985	-0.027	0.264
324	pm11-pm20	0.982	1.038	0.995	0.986	-0.007	0.59
325	pm11-pm21	1.105	1.276	1.357	1.003	-0.08	0.014
326	pm11-pm22	1.05	1.179	1.084	1.041	0.005	0.755
327	pm11-pm23	1.065	1.247	1.115	1.064	-0.004	0.706
328	pm11-pm24	0.979	1.031	1.021	0.993	-0.003	0.833
329	pm11-pm25	1.02	1.02	1.001	1.014	0.009	0.498
330	pm11-pm26	0.969	1	1	0.983	-0.017	0.339
331	pm11-pm27	1.007	1.042	1.028	0.983	-0.011	0.346
332	pm11-pm28	1.259	1.037	1.104	1.337	-0.076	0.019
333	pm11-pm29	1.021	1.177	0.986	1.093	-0.012	0.396
334	pm11-pm30	0.917	0.894	0.955	0.918	0.003	0.731
335	pm11-pm31	1.025	1.026	1.016	1.007	-0.01	0.52
336	pm11-pm32	0.997	1.126	0.993	1.026	-0.007	0.568
337	pm11-pm33	1.01	1	0.973	1.039	0.001	0.933
338	pm11-pm34	0.986	1.046	1.001	0.971	-0.008	0.46
339	pm11-pm35	0.974	1.015	1.017	1.002	-0.008	0.581
340	pm11-pm36	1.011	0.998	0.985	1.019	0	0.911

341	pm11-pm37	1.004	0.989	0.987	1.046	0	0.65
342	pm12-pm13	1.075	1.172	0.994	1.243	-0.07	0.034
343	pm12-pm14	1.102	1.198	1.073	1.226	-0.036	0.222
344	pm12-pm15	1.087	1.12	1.017	1.252	-0.07	0.029
345	pm12-pm16	1.132	1.22	1.017	1.244	-0.03	0.232
346	pm12-pm17	1.105	1.215	1.015	1.233	-0.042	0.182
347	pm12-pm18	1.09	1.161	1.041	1.209	-0.057	0.033
348	pm12-pm19	1.095	1.163	1.036	1.209	-0.057	0.026
349	pm12-pm20	1.122	1.142	1.01	1.214	-0.063	0.038
350	pm12-pm21	1.272	1.536	1.428	1.255	-0.071	0.016
351	pm12-pm22	1.154	1.35	1.149	1.245	-0.037	0.297
352	pm12-pm23	1.178	1.426	1.134	1.312	-0.043	0.195
353	pm12-pm24	1.079	1.168	1.024	1.234	-0.06	0.023
354	pm12-pm25	1.099	1.168	1.069	1.224	-0.043	0.159
355	pm12-pm26	1.104	1.168	1.036	1.22	-0.051	0.023
356	pm12-pm27	1.115	1.193	1.072	1.26	-0.044	0.172
357	pm12-pm28	1.403	1.197	1.184	1.65	-0.08	0.013
358	pm12-pm29	1.122	1.448	1.056	1.3	-0.028	0.238
359	pm12-pm30	1.067	1.051	0.973	1.12	-0.043	0.156
360	pm12-pm31	1.112	1.215	1.062	1.239	-0.05	0.198
361	pm12-pm32	1.085	1.297	1.027	1.275	-0.05	0.191
362	pm12-pm33	1.071	1.125	1.046	1.201	-0.072	0.019
363	pm12-pm34	1.112	1.205	1.026	1.162	-0.061	0.021
364	pm12-pm35	1.088	1.226	1.037	1.169	-0.058	0.022
365	pm12-pm36	1.111	1.149	1.016	1.213	-0.06	0.041
366	pm12-pm37	1.098	1.162	1.028	1.206	-0.061	0.038
367	pm13-pm14	0.994	1.028	0.999	1	-0.005	0.863
368	pm13-pm15	1	0.964	1.011	0.977	-0.025	0.264
369	pm13-pm16	1	0.975	0.994	1.001	-0.016	0.33
370	pm13-pm17	0.957	0.968	1	0.952	-0.04	0.296

371	pm13-pm18	1.009	1.045	1.003	0.952	-0.004	0.817
372	pm13-pm19	1.002	1.027	0.992	0.965	-0.011	0.353
373	pm13-pm20	1.011	1.03	1.008	1.017	0.007	0.547
374	pm13-pm21	1.116	1.332	1.398	0.999	-0.057	0.021
375	pm13-pm22	1.017	1.166	1.095	1	-0.017	0.377
376	pm13-pm23	1.099	1.22	1.104	1.036	-0.015	0.363
377	pm13-pm24	0.984	1.062	1.006	0.998	0.001	0.921
378	pm13-pm25	0.979	1.035	1.002	0.984	-0.007	0.525
379	pm13-pm26	0.995	1.047	0.969	0.994	-0.006	0.424
380	pm13-pm27	0.971	1.067	0.999	0.998	-0.021	0.214
381	pm13-pm28	1.242	1.13	1.148	1.348	-0.046	0.19
382	pm13-pm29	0.968	1.129	0.964	1.052	-0.056	0.037
383	pm13-pm30	0.894	0.895	0.919	0.872	-0.026	0.236
384	pm13-pm31	1.011	1.025	1.005	1.007	-0.019	0.311
385	pm13-pm32	1.01	1.17	0.992	1.007	0	0.861
386	pm13-pm33	1.027	1.044	0.997	0.973	0.003	0.938
387	pm13-pm34	1.01	0.999	0.964	0.971	-0.025	0.216
388	pm13-pm35	0.961	1.017	0.972	0.962	-0.035	0.218
389	pm13-pm36	0.981	1.031	0.988	0.992	-0.009	0.416
390	pm13-pm37	0.99	1.034	1.009	0.983	-0.006	0.592
391	pm14-pm15	0.991	0.955	1.031	0.987	-0.017	0.348
392	pm14-pm16	0.993	0.978	1.007	0.952	-0.02	0.298
393	pm14-pm17	0.976	0.99	1.018	0.972	-0.015	0.319
394	pm14-pm18	1.015	1.058	1.004	0.987	0.015	0.328
395	pm14-pm19	0.993	1.058	1.006	0.971	0.006	0.6
396	pm14-pm20	0.99	1.04	1.004	0.981	-0.001	0.799
397	pm14-pm21	1.105	1.315	1.395	1.009	-0.057	0.027
398	pm14-pm22	1.068	1.161	1.107	1.007	0.005	0.728
399	pm14-pm23	1.063	1.19	1.11	1.028	-0.026	0.298
400	pm14-pm24	1.007	1.025	1.009	0.999	0.004	0.785

401	pm14-pm25	1.021	1.048	1.005	1.001	0.017	0.32
402	pm14-pm26	1.012	1.041	1.017	0.993	0.014	0.316
403	pm14-pm27	1.016	1.058	1.041	1.008	0.007	0.415
404	pm14-pm28	1.254	1.108	1.104	1.326	-0.06	0.035
405	pm14-pm29	0.972	1.128	0.985	1.028	-0.051	0.043
406	pm14-pm30	0.913	0.877	0.95	0.877	-0.011	0.306
407	pm14-pm31	1.042	1.106	1.011	1.004	0.015	0.385
408	pm14-pm32	1.009	1.147	1.021	1.021	0.009	0.579
409	pm14-pm33	1.012	1.023	1.018	0.991	0.009	0.578
410	pm14-pm34	0.983	1.021	0.982	0.976	-0.016	0.302
411	pm14-pm35	0.983	0.997	1.029	0.993	-0.007	0.458
412	pm14-pm36	0.985	1.006	1.018	1	0.001	0.766
413	pm14-pm37	1.008	1.028	0.99	1	0.002	0.836
414	pm15-pm16	1.014	1.012	1.017	1.019	0.01	0.508
415	pm15-pm17	1.014	0.986	1.022	0.999	-0.001	0.701
416	pm15-pm18	0.97	1.019	1.022	0.997	-0.002	0.898
417	pm15-pm19	1.009	0.999	1.013	1.032	0.009	0.455
418	pm15-pm20	1.009	1.031	1.043	0.971	0.006	0.432
419	pm15-pm21	1.104	1.269	1.383	1.05	-0.064	0.039
420	pm15-pm22	1.067	1.112	1.087	1.015	-0.014	0.306
421	pm15-pm23	1.068	1.236	1.069	1.003	-0.033	0.24
422	pm15-pm24	1.005	0.986	1.024	1.031	0.002	0.922
423	pm15-pm25	0.997	1.051	1.022	0.993	0.011	0.38
424	pm15-pm26	0.989	1.048	1.039	1.021	0.019	0.363
425	pm15-pm27	0.995	1.068	1.023	1.019	0	0.66
426	pm15-pm28	1.238	1.103	1.212	1.348	-0.036	0.209
427	pm15-pm29	1.015	1.095	0.995	1.1	-0.031	0.223
428	pm15-pm30	0.894	0.903	0.991	0.889	0.001	0.613
429	pm15-pm31	1.027	1.047	1.05	1.018	0.007	0.482
430	pm15-pm32	0.97	1.107	1.028	1.033	-0.009	0.509

431	pm15-pm33	0.959	1.003	1.034	1.002	-0.006	0.566
432	pm15-pm34	1.027	0.987	1.012	0.982	-0.007	0.566
433	pm15-pm35	0.967	0.972	0.992	1	-0.028	0.267
434	pm15-pm36	0.985	1.039	1.012	0.995	0.004	0.933
435	pm15-pm37	0.991	1.022	1.017	0.995	-0.001	0.991
436	pm16-pm17	0.963	1.012	1.011	0.991	-0.007	0.572
437	pm16-pm18	0.99	1.004	1.02	1.001	0.005	0.73
438	pm16-pm19	0.998	1.027	1.021	0.989	0.01	0.586
439	pm16-pm20	0.979	0.999	1.001	0.974	-0.014	0.396
440	pm16-pm21	1.135	1.327	1.36	1.025	-0.049	0.177
441	pm16-pm22	1.037	1.163	1.072	1.003	-0.01	0.538
442	pm16-pm23	1.077	1.216	1.101	1.012	-0.02	0.266
443	pm16-pm24	0.995	1.048	1.017	0.994	0.01	0.575
444	pm16-pm25	0.827	1.047	1.033	1.178	0.022	0.266
445	pm16-pm26	1.018	1.049	1.006	0.985	0.015	0.303
446	pm16-pm27	1.018	1.059	1.018	0.996	0.001	0.871
447	pm16-pm28	1.231	1.132	1.113	1.374	-0.043	0.179
448	pm16-pm29	1.043	1.18	0.955	1.053	-0.019	0.347
449	pm16-pm30	0.903	0.846	0.928	0.919	-0.014	0.357
450	pm16-pm31	1.004	1.067	1.049	0.996	0.006	0.415
451	pm16-pm32	0.983	1.107	1.027	1.064	0.008	0.485
452	pm16-pm33	1.021	1.038	0.982	0.987	0.007	0.548
453	pm16-pm34	1.018	0.961	1.025	0.96	-0.013	0.397
454	pm16-pm35	0.971	0.97	1.004	0.976	-0.024	0.288
455	pm16-pm36	0.996	0.984	1.008	1.004	-0.001	0.918
456	pm16-pm37	0.994	1.024	1.021	1.007	0.01	0.394
457	pm17-pm18	0.976	0.975	1.023	0.987	-0.01	0.53
458	pm17-pm19	1.007	1.003	1.044	1.005	0.015	0.382
459	pm17-pm20	0.961	1.027	1.004	0.98	-0.01	0.319
460	pm17-pm21	1.089	1.251	1.38	0.971	-0.089	0.012

461	pm17-pm22	1.045	1.096	1.084	0.975	-0.03	0.285
462	pm17-pm23	1.088	1.202	1.126	1.014	-0.016	0.371
463	pm17-pm24	1.023	1.025	1.041	0.994	0.016	0.334
464	pm17-pm25	1.007	1.035	1.015	0.991	0.011	0.33
465	pm17-pm26	0.984	1.038	0.989	1.003	0.003	0.741
466	pm17-pm27	1.016	0.97	1.013	0.997	-0.023	0.272
467	pm17-pm28	1.223	1.09	1.188	1.35	-0.043	0.189
468	pm17-pm29	0.998	1.173	0.956	1.024	-0.04	0.267
469	pm17-pm30	0.894	0.862	0.925	0.869	-0.026	0.254
470	pm17-pm31	0.981	1.035	1.049	0.986	-0.011	0.301
471	pm17-pm32	1.006	1.144	1.006	1.013	0.004	0.846
472	pm17-pm33	0.99	0.995	0.997	0.974	-0.012	0.308
473	pm17-pm34	0.969	0.947	0.984	0.957	-0.04	0.15
474	pm17-pm35	0.989	0.962	1.021	0.983	-0.017	0.35
475	pm17-pm36	1.001	1	1.016	0.964	-0.005	0.646
476	pm17-pm37	0.972	1.002	0.97	1.001	-0.017	0.356
477	pm18-pm19	0.998	0.999	0.98	1.009	-0.001	0.841
478	pm18-pm20	0.986	0.992	0.994	0.994	-0.009	0.547
479	pm18-pm21	1.076	1.24	1.407	1.034	-0.07	0.013
480	pm18-pm22	1.048	1.159	1.099	0.999	-0.001	0.724
481	pm18-pm23	1.119	1.247	1.111	1.053	0.012	0.384
482	pm18-pm24	0.992	1.026	1.007	1.019	0.009	0.549
483	pm18-pm25	1.016	0.988	1.015	1.012	0.009	0.474
484	pm18-pm26	0.989	0.99	1.015	0.997	0	0.629
485	pm18-pm27	1.007	1.023	1.016	1.032	0	0.751
486	pm18-pm28	1.252	1.116	1.13	1.409	-0.027	0.253
487	pm18-pm29	1.011	1.197	0.996	1.028	-0.017	0.383
488	pm18-pm30	0.945	0.875	0.954	0.88	0.002	0.842
489	pm18-pm31	1.035	1.012	1.053	1.002	0.004	0.91
490	pm18-pm32	0.986	1.145	1.008	1.037	0.008	0.527

491	pm18-pm33	1.006	1.004	0.986	1.013	0.004	0.684
492	pm18-pm34	1.022	1.048	1.008	0.976	0.011	0.314
493	pm18-pm35	0.999	1.008	0.992	0.995	-0.005	0.935
494	pm18-pm36	0.99	0.981	0.98	0.996	-0.011	0.319
495	pm18-pm37	1.021	0.989	1.015	1.009	0.008	0.468
496	pm19-pm20	0.993	1.014	0.982	0.982	-0.009	0.464
497	pm19-pm21	1.112	1.289	1.366	0.994	-0.069	0.026
498	pm19-pm22	1.047	1.175	1.105	1.049	0.016	0.361
499	pm19-pm23	1.07	1.216	1.102	1.049	-0.012	0.34
500	pm19-pm24	0.977	1.04	1.002	0.982	-0.002	0.827
501	pm19-pm25	0.987	0.999	0.991	1.002	-0.004	0.758
502	pm19-pm26	0.981	0.989	1.011	0.984	-0.007	0.497
503	pm19-pm27	1.034	0.985	1.042	1.028	0.002	0.986
504	pm19-pm28	1.229	1.065	1.119	1.32	-0.071	0.019
505	pm19-pm29	1.026	1.234	1.056	1.054	0.017	0.342
506	pm19-pm30	0.932	0.88	0.937	0.882	-0.004	0.962
507	pm19-pm31	1.017	1.033	1.059	1.005	0.006	0.438
508	pm19-pm32	0.978	1.117	0.992	1.021	-0.009	0.498
509	pm19-pm33	0.983	1.012	1	0.981	-0.005	0.966
510	pm19-pm34	0.988	1.04	1.014	0.986	0.004	0.696
511	pm19-pm35	1.016	1.043	0.988	0.99	0.005	0.424
512	pm19-pm36	0.988	0.981	1.014	0.996	-0.003	0.828
513	pm19-pm37	1.01	0.993	1.022	1.011	0.008	0.44
514	pm20-pm21	1.113	1.219	1.417	1.008	-0.073	0.017
515	pm20-pm22	1.063	1.156	1.077	0.996	-0.008	0.53
516	pm20-pm23	1.07	1.255	1.107	1.038	-0.007	0.495
517	pm20-pm24	1.006	1.017	0.976	0.99	-0.008	0.437
518	pm20-pm25	0.99	0.991	0.985	0.996	-0.011	0.335
519	pm20-pm26	1.013	0.995	1.016	0.993	0.003	0.672
520	pm20-pm27	1.019	1.07	1.016	1.021	0.008	0.593

521	pm20-pm28	1.214	1.078	1.136	1.367	-0.059	0.026
522	pm20-pm29	1.068	1.153	0.995	1.074	-0.006	0.419
523	pm20-pm30	0.939	0.893	0.952	0.866	-0.002	0.642
524	pm20-pm31	1.045	1.046	1.013	1.02	0.005	0.4
525	pm20-pm32	0.987	1.162	1.003	1.049	0.011	0.383
526	pm20-pm33	0.989	1.049	1.005	0.991	0.007	0.553
527	pm20-pm34	1.003	1.063	1.011	0.976	0.007	0.564
528	pm20-pm35	0.999	1.023	0.972	0.998	-0.009	0.571
529	pm20-pm36	0.984	1.003	1.009	1.009	0.001	0.699
530	pm20-pm37	0.992	0.999	1.01	0.989	-0.006	0.425
531	pm21-pm22	1.19	1.403	1.445	1.059	-0.065	0.025
532	pm21-pm23	1.209	1.533	1.539	1.063	-0.046	0.167
533	pm21-pm24	1.11	1.311	1.388	1.03	-0.054	0.028
534	pm21-pm25	1.111	1.282	1.381	1.027	-0.06	0.025
535	pm21-pm26	1.109	1.28	1.365	1.018	-0.067	0.036
536	pm21-pm27	1.083	1.272	1.357	1.062	-0.088	0.017
537	pm21-pm28	1.354	1.35	1.529	1.351	-0.12	0.008
538	pm21-pm29	1.105	1.513	1.397	1.08	-0.063	0.036
539	pm21-pm30	1.019	1.174	1.368	0.904	-0.057	0.033
540	pm21-pm31	1.141	1.343	1.35	1.05	-0.063	0.025
541	pm21-pm32	1.124	1.467	1.387	1.045	-0.042	0.193
542	pm21-pm33	1.146	1.278	1.381	1.021	-0.054	0.04
543	pm21-pm34	1.121	1.303	1.359	0.988	-0.072	0.019
544	pm21-pm35	1.125	1.267	1.382	1.046	-0.06	0.02
545	pm21-pm36	1.12	1.306	1.384	1.027	-0.05	0.156
546	pm21-pm37	1.142	1.306	1.355	1.02	-0.057	0.025
547	pm22-pm23	1.353	1.527	1.429	1.048	0.138	0.008
548	pm22-pm24	1.055	1.218	1.077	1.033	0.014	0.347
549	pm22-pm25	1.058	1.104	1.118	1.013	-0.005	0.962
550	pm22-pm26	1.036	1.178	1.093	1.039	0.008	0.537

551	pm22-pm27	1.066	1.196	1.142	1.005	0.002	0.872
552	pm22-pm28	1.305	1.273	1.295	1.432	-0.008	0.44
553	pm22-pm29	1.111	1.291	1.078	1.076	-0.016	0.39
554	pm22-pm30	0.928	1.014	1.052	0.93	-0.01	0.325
555	pm22-pm31	1.07	1.201	1.076	1.03	-0.008	0.431
556	pm22-pm32	1.072	1.279	1.066	1.068	0.005	0.564
557	pm22-pm33	1.025	1.134	1.094	1.018	-0.01	0.309
558	pm22-pm34	1.04	1.138	1.073	1.039	-0.01	0.527
559	pm22-pm35	1.05	1.099	1.088	1.015	-0.021	0.271
560	pm22-pm36	1.043	1.142	1.116	1.034	0.006	0.497
561	pm22-pm37	1.063	1.155	1.121	1.026	0.011	0.36
562	pm23-pm24	1.139	1.238	1.116	1.06	0.013	0.399
563	pm23-pm25	1.042	1.269	1.096	1.02	-0.015	0.357
564	pm23-pm26	1.093	1.262	1.107	1.036	0.003	0.672
565	pm23-pm27	1.122	1.272	1.122	1.024	-0.008	0.578
566	pm23-pm28	1.37	1.392	1.245	1.388	-0.028	0.262
567	pm23-pm29	1.072	1.419	1.06	1.088	-0.039	0.201
568	pm23-pm30	0.989	1.115	1.063	0.94	-0.008	0.524
569	pm23-pm31	1.107	1.29	1.123	1.044	-0.004	0.982
570	pm23-pm32	1.084	1.413	1.115	1.093	0.017	0.397
571	pm23-pm33	1.078	1.269	1.112	1.028	0	0.976
572	pm23-pm34	1.078	1.246	1.082	1.019	-0.019	0.322
573	pm23-pm35	1.059	1.242	1.106	1.013	-0.022	0.222
574	pm23-pm36	1.089	1.203	1.111	1.04	-0.01	0.498
575	pm23-pm37	1.062	1.281	1.104	1.048	0	0.955
576	pm24-pm25	0.994	0.978	1.005	1.017	-0.004	0.688
577	pm24-pm26	0.988	1.042	0.986	0.981	-0.004	0.614
578	pm24-pm27	1.004	1.113	1.041	1.014	0.018	0.339
579	pm24-pm28	1.268	1.12	1.181	1.349	-0.029	0.271
580	pm24-pm29	0.977	1.164	1.023	1.03	-0.032	0.252

581	pm24-pm30	0.885	0.922	0.94	0.915	-0.001	0.601
582	pm24-pm31	1.02	1.076	1.022	1.022	0.009	0.481
583	pm24-pm32	1.007	1.141	1.02	1.034	0.01	0.423
584	pm24-pm33	0.981	0.991	0.991	0.987	-0.015	0.33
585	pm24-pm34	1.018	1.042	0.991	0.981	0.001	0.871
586	pm24-pm35	1.002	0.979	1.02	0.996	-0.009	0.457
587	pm24-pm36	0.993	1.01	1.019	0.995	0.002	0.856
588	pm24-pm37	0.998	1.007	1.003	1.005	-0.002	0.768
589	pm25-pm26	1.01	0.997	1.005	0.982	0	0.998
590	pm25-pm27	1.018	1.03	1.016	1.027	0.002	0.873
591	pm25-pm28	1.246	1.044	1.142	1.342	-0.062	0.033
592	pm25-pm29	0.999	1.148	1.005	1.026	-0.032	0.253
593	pm25-pm30	0.931	0.896	0.968	0.907	0.013	0.306
594	pm25-pm31	1.028	1.045	1.047	0.985	0.003	0.765
595	pm25-pm32	0.992	1.108	1.007	1.039	-0.001	0.671
596	pm25-pm33	1.014	0.982	1.01	0.999	0.002	0.799
597	pm25-pm34	1.006	1.029	0.986	1.014	0.006	0.458
598	pm25-pm35	0.982	0.988	0.989	0.96	-0.025	0.228
599	pm25-pm36	0.993	1.016	1.014	1.017	0.012	0.303
600	pm25-pm37	1.014	0.99	1.008	1.024	0.008	0.597
601	pm26-pm27	1.02	1.033	1.032	0.987	-0.003	0.656
602	pm26-pm28	1.223	1.062	1.1	1.38	-0.063	0.03
603	pm26-pm29	0.99	1.146	0.983	1.086	-0.025	0.251
604	pm26-pm30	0.935	0.913	0.942	0.889	0.007	0.405
605	pm26-pm31	0.999	1.055	1.017	1.018	0	0.778
606	pm26-pm32	0.987	1.091	1.009	1.016	-0.011	0.341
607	pm26-pm33	0.981	1.006	0.995	0.987	-0.007	0.493
608	pm26-pm34	1.006	1.024	1.015	0.997	0.007	0.581
609	pm26-pm35	1.007	1.077	0.977	0.987	0.008	0.592
610	pm26-pm36	1.004	0.979	1.007	1.006	0.001	0.762

611	pm26-pm37	0.992	1.016	1.008	1.02	0.007	0.509
612	pm27-pm28	1.221	1.129	1.148	1.374	-0.058	0.043
613	pm27-pm29	1.04	1.239	0.998	1.036	-0.02	0.366
614	pm27-pm30	0.936	0.93	0.969	0.893	-0.002	0.708
615	pm27-pm31	1.004	1.107	1.073	0.991	-0.001	0.831
616	pm27-pm32	0.999	1.164	1.026	1.053	0.002	0.722
617	pm27-pm33	0.992	1.019	0.997	1.003	-0.018	0.322
618	pm27-pm34	1.046	1.058	1.049	0.984	0.01	0.442
619	pm27-pm35	1.014	1.092	1.022	1.02	0.011	0.324
620	pm27-pm36	1.03	1.072	1.012	1.009	0.011	0.343
621	pm27-pm37	1.029	1.034	1.012	1.026	0.003	0.65
622	pm28-pm29	1.332	1.212	1.095	1.479	-0.052	0.042
623	pm28-pm30	1.151	0.968	1.089	1.238	-0.057	0.024
624	pm28-pm31	1.278	1.108	1.152	1.379	-0.049	0.157
625	pm28-pm32	1.226	1.182	1.156	1.4	-0.052	0.042
626	pm28-pm33	1.206	1.018	1.11	1.354	-0.083	0.016
627	pm28-pm34	1.249	1.114	1.148	1.384	-0.035	0.259
628	pm28-pm35	1.238	1.083	1.13	1.321	-0.067	0.043
629	pm28-pm36	1.198	1.091	1.122	1.296	-0.077	0.014
630	pm28-pm37	1.21	1.049	1.121	1.348	-0.075	0.01
631	pm29-pm30	0.918	1.291	0.787	1.132	0.043	0.177
632	pm29-pm31	1.025	1.238	1.011	1.104	-0.005	0.452
633	pm29-pm32	1.039	1.31	0.991	1.085	-0.008	0.424
634	pm29-pm33	0.969	1.158	0.992	1.048	-0.035	0.281
635	pm29-pm34	1.011	1.154	0.981	1.01	-0.041	0.154
636	pm29-pm35	1.015	1.119	0.981	1.066	-0.036	0.258
637	pm29-pm36	1.002	1.163	1.021	1.082	-0.009	0.578
638	pm29-pm37	1.011	1.192	1.002	1.056	-0.013	0.309
639	pm30-pm31	0.935	0.971	0.997	0.877	0.009	0.519
640	pm30-pm32	0.93	0.975	0.984	0.936	0.006	0.586

641	pm30-pm33	0.941	0.91	0.969	0.93	0.025	0.288
642	pm30-pm34	0.895	0.894	0.975	0.886	-0.004	0.924
643	pm30-pm35	0.942	0.905	0.971	0.894	0.01	0.376
644	pm30-pm36	0.957	0.895	0.939	0.922	0.017	0.383
645	pm30-pm37	0.95	0.872	0.921	0.935	0.005	0.571
646	pm31-pm32	0.995	1.178	1.025	1.042	-0.001	0.802
647	pm31-pm33	1.031	0.998	1.034	1.016	-0.003	0.72
648	pm31-pm34	1.001	1.063	1.035	1.015	0.002	0.962
649	pm31-pm35	1.01	1.056	1.037	0.997	-0.003	0.879
650	pm31-pm36	1.008	1.047	1.05	1.027	0.011	0.361
651	pm31-pm37	1.015	1.066	1.035	1.03	0.012	0.366
652	pm32-pm33	0.982	1.151	1.032	1.015	0.008	0.494
653	pm32-pm34	1.013	1.187	1.005	1.01	0.013	0.386
654	pm32-pm35	1.021	1.101	0.993	1.046	-0.002	0.716
655	pm32-pm36	1.006	1.148	0.987	1.014	0.002	0.722
656	pm32-pm37	0.987	1.161	1.01	1.016	0.004	0.958
657	pm33-pm34	0.987	1.163	1.006	0.985	0.032	0.276
658	pm33-pm35	1.001	1.037	0.993	0.996	0.002	0.67
659	pm33-pm36	1.002	0.99	0.995	1.014	0.002	0.975
660	pm33-pm37	1.012	1.014	0.99	1.023	0.008	0.432
661	pm34-pm35	1.035	0.996	1.017	0.973	-0.003	0.646
662	pm34-pm36	1.026	1.026	1.034	1.003	0.019	0.337
663	pm34-pm37	1.018	1.031	1.002	1.014	0.011	0.383
664	pm35-pm36	1.025	1.054	0.968	0.996	0.007	0.503
665	pm35-pm37	0.988	0.999	1.002	0.997	-0.01	0.339
666	pm36-pm37	0.985	1.007	1.005	0.995	-0.003	0.89

3c) Result of two-distribution perturbation analysis for apoptosis when each response function was separately applied (100 million parameter sets)

No.	Pair of perturbed parameter distributions	Lin	Hill	Sat	Acc	Synergistic effect	p-value
1	pm1-pm2	1.03	1.057	1.034	1.045	0.028	0.23
2	pm1-pm3	1.034	1.069	1.058	1.055	0.032	0.273
3	pm1-pm4	1.031	1.062	1.011	1.005	-0.014	0.397
4	pm1-pm5	1.019	0.996	1.026	1.014	0	0.939
5	pm1-pm6	1.055	1.029	1.155	1.056	0.022	0.231
6	pm1-pm7	1.944	1.612	2.075	1.613	-0.109	0.008
7	pm1-pm8	1.013	1.033	1.022	1.002	0.005	0.835
8	pm1-pm9	1.06	1.124	1.066	1.059	0.027	0.208
9	pm1-pm10	2.265	1.896	2.043	2.238	0.076	0.018
10	pm1-pm11	1.025	1.087	0.99	1.029	0.012	0.371
11	pm1-pm12	1.152	1.215	1.056	1.267	-0.026	0.256
12	pm1-pm13	0.972	1.051	0.993	0.975	-0.026	0.282
13	pm1-pm14	1.012	1.071	0.993	1.046	0.012	0.361
14	pm1-pm15	1.052	1.078	1.067	1.013	0.031	0.23
15	pm1-pm16	0.996	1.077	1.048	1.019	0.019	0.335
16	pm1-pm17	1.002	1.045	1.028	0.988	-0.001	0.759
17	pm1-pm18	0.998	0.995	1.033	1.004	-0.007	0.483
18	pm1-pm19	1.001	1.031	1.014	1.037	0.006	0.535
19	pm1-pm20	1.012	1.032	1.01	1.033	0.004	0.634
20	pm1-pm21	1.163	1.285	1.412	1.066	-0.045	0.156
21	pm1-pm22	1.088	1.197	1.119	1.072	0.025	0.243
22	pm1-pm23	1.119	1.245	1.15	1.087	0.013	0.38
23	pm1-pm24	1.017	1.068	1.01	1.027	0.012	0.36
24	pm1-pm25	1.043	1.034	0.994	1.04	0.013	0.382

25	pm1-pm26	1.024	1.016	1.012	1.02	0.003	0.88
26	pm1-pm27	1.028	1.085	1.03	1.047	0.011	0.367
27	pm1-pm28	1.262	1.112	1.149	1.382	-0.045	0.156
28	pm1-pm29	0.988	1.233	1.012	1.097	-0.01	0.468
29	pm1-pm30	0.925	0.951	0.951	0.926	0.01	0.418
30	pm1-pm31	1.01	1.058	1.043	1.018	-0.007	0.529
31	pm1-pm32	1.022	1.187	1.033	1.066	0.024	0.23
32	pm1-pm33	1.02	1.059	1.033	1.031	0.02	0.24
33	pm1-pm34	1.038	1.109	1.03	0.992	0.023	0.211
34	pm1-pm35	1.019	1.107	1.02	1.019	0.021	0.23
35	pm1-pm36	1.028	1.041	1.003	1.032	0.012	0.369
36	pm1-pm37	1.009	1.058	1.021	1.013	0.008	0.417
37	pm2-pm3	1.01	1.039	1.01	0.999	0.011	0.367
38	pm2-pm4	0.991	1.061	1.026	0.975	-0.011	0.306
39	pm2-pm5	1.004	0.985	0.974	1.018	-0.001	0.877
40	pm2-pm6	1.014	1	1.109	1.027	0.003	0.907
41	pm2-pm7	1.967	1.565	2.081	1.61	-0.096	0.013
42	pm2-pm8	0.98	0.991	0.986	1.015	-0.002	0.72
43	pm2-pm9	1.05	1.077	1.01	1.011	0.005	0.975
44	pm2-pm10	2.281	1.887	2.153	2.303	0.139	0.009
45	pm2-pm11	0.983	1.003	0.995	1.015	-0.004	0.62
46	pm2-pm12	1.119	1.157	1.041	1.176	-0.058	0.04
47	pm2-pm13	0.975	1.069	1.005	1.002	0.007	0.418
48	pm2-pm14	0.964	1.034	1.026	0.982	0.001	0.923
49	pm2-pm15	1.023	1.03	1.023	0.99	0.013	0.384
50	pm2-pm16	1.03	1.026	1.001	1.02	0.021	0.266
51	pm2-pm17	1.004	1.009	0.99	0.954	-0.01	0.485
52	pm2-pm18	0.978	1	0.987	1.009	-0.003	0.853
53	pm2-pm19	1.006	1.014	1.004	1.017	0.013	0.36
54	pm2-pm20	0.993	1.035	0.994	0.985	0.002	0.717

55	pm2-pm21	1.097	1.275	1.378	1.013	-0.068	0.024
56	pm2-pm22	1.044	1.151	1.081	1.037	0.002	0.81
57	pm2-pm23	1.06	1.228	1.109	1.06	-0.005	0.534
58	pm2-pm24	0.964	1.039	1.006	0.986	-0.003	0.805
59	pm2-pm25	0.978	0.976	0.986	0.992	-0.014	0.368
60	pm2-pm26	0.976	0.99	1.012	0.989	-0.005	0.429
61	pm2-pm27	1.025	1.015	1.045	1.015	0.006	0.505
62	pm2-pm28	1.233	1.074	1.095	1.356	-0.064	0.04
63	pm2-pm29	1.033	1.237	1.009	1.048	0.007	0.501
64	pm2-pm30	0.92	0.919	0.971	0.895	0.016	0.379
65	pm2-pm31	1.01	1.029	1.016	0.994	-0.009	0.481
66	pm2-pm32	0.981	1.134	1.008	1.015	-0.001	0.716
67	pm2-pm33	1	0.995	0.993	1.007	0.001	0.604
68	pm2-pm34	0.959	1.022	1.028	0.999	0	0.959
69	pm2-pm35	0.996	1.012	0.993	1.001	-0.002	0.71
70	pm2-pm36	0.996	1	1.007	0.991	0.002	0.867
71	pm2-pm37	0.982	1.01	1.001	1.003	-0.001	0.963
72	pm3-pm4	0.988	1.036	0.999	1.019	-0.021	0.242
73	pm3-pm5	0.991	1.021	1.017	1.039	0.013	0.37
74	pm3-pm6	1.061	1.016	1.073	1.02	0.001	0.938
75	pm3-pm7	1.943	1.587	2.099	1.612	-0.099	0.016
76	pm3-pm8	1.005	1.025	1.029	1.001	0.012	0.361
77	pm3-pm9	1.035	1.024	1.061	1	-0.01	0.352
78	pm3-pm10	2.291	1.948	2.459	2.025	0.156	0.004
79	pm3-pm11	1.01	1.01	1.024	0.996	0	0.663
80	pm3-pm12	1.098	1.192	1.016	1.192	-0.064	0.04
81	pm3-pm13	0.995	1.05	0.993	0.967	-0.012	0.32
82	pm3-pm14	1.007	1.051	0.999	1.002	0.007	0.531
83	pm3-pm15	0.993	1.035	0.982	1.013	-0.006	0.403
84	pm3-pm16	0.97	0.997	1.014	0.997	-0.011	0.38

85	pm3-pm17	0.98	0.99	1.014	0.977	-0.017	0.353
86	pm3-pm18	1.021	0.995	1.008	1.012	0.004	0.753
87	pm3-pm19	0.982	1.019	1.015	1.008	0.001	0.645
88	pm3-pm20	0.994	1.031	1.016	0.999	0.002	0.817
89	pm3-pm21	1.124	1.287	1.337	1.005	-0.078	0.016
90	pm3-pm22	1.069	1.163	1.064	1.034	-0.002	0.713
91	pm3-pm23	1.073	1.221	1.146	1.054	-0.004	0.641
92	pm3-pm24	1.016	1.028	1.027	1.014	0.012	0.327
93	pm3-pm25	0.998	1	1.014	0.997	-0.003	0.92
94	pm3-pm26	0.992	1.016	1.027	1.017	0.008	0.522
95	pm3-pm27	1.008	1.04	1.038	1.031	0.003	0.67
96	pm3-pm28	1.232	1.053	1.146	1.357	-0.064	0.021
97	pm3-pm29	0.995	1.194	1.001	1.041	-0.025	0.239
98	pm3-pm30	0.899	0.892	0.972	0.895	-0.004	0.911
99	pm3-pm31	1.018	1.055	1.04	1.01	0.002	0.716
100	pm3-pm32	0.99	1.144	1.037	1.047	0.011	0.395
101	pm3-pm33	1.008	1.008	1.026	1.016	0.009	0.594
102	pm3-pm34	1.044	1.079	1	0.98	0.016	0.395
103	pm3-pm35	1.015	1.023	1.022	0.988	0.002	0.67
104	pm3-pm36	0.987	0.983	1.021	1.026	-0.001	0.825
105	pm3-pm37	1.026	0.984	1.015	1.031	0.007	0.523
106	pm4-pm5	1.015	1.053	0.994	1.026	-0.002	0.801
107	pm4-pm6	0.986	1.012	1.076	0.986	-0.047	0.152
108	pm4-pm7	1.921	1.625	2.073	1.588	-0.128	0.009
109	pm4-pm8	1.022	1.04	1	0.987	-0.011	0.347
110	pm4-pm9	1.027	1.108	1.056	1.04	-0.002	0.771
111	pm4-pm10	2.095	1.882	2.002	2.149	-0.013	0.34
112	pm4-pm11	1.02	1.05	1.019	0.999	-0.009	0.452
113	pm4-pm12	1.128	1.233	1.066	1.205	-0.051	0.03
114	pm4-pm13	0.983	1.053	1.024	0.97	-0.026	0.286

115	pm4-pm14	1.009	1.062	1.031	0.997	-0.003	0.815
116	pm4-pm15	1.008	1.077	1.041	0.979	-0.005	0.86
117	pm4-pm16	1.018	1.089	1.004	0.963	-0.007	0.545
118	pm4-pm17	1.016	1.082	1.016	0.996	0	0.675
119	pm4-pm18	1.014	1.021	1.01	0.974	-0.02	0.213
120	pm4-pm19	1.021	1.022	1.014	1.004	-0.01	0.589
121	pm4-pm20	1.021	1.075	1.005	1.002	-0.002	0.798
122	pm4-pm21	1.144	1.294	1.429	1.004	-0.069	0.045
123	pm4-pm22	1.066	1.243	1.16	1.058	0.027	0.25
124	pm4-pm23	1.132	1.404	1.125	1.065	0.034	0.23
125	pm4-pm24	1.014	1.049	1.04	0.999	-0.004	0.986
126	pm4-pm25	1.005	1.06	0.995	1.01	-0.008	0.494
127	pm4-pm26	1.028	1.072	0.994	0.976	-0.008	0.403
128	pm4-pm27	1.025	1.053	1.026	1.024	-0.015	0.339
129	pm4-pm28	1.235	1.16	1.17	1.334	-0.056	0.022
130	pm4-pm29	1.04	1.314	0.993	1.108	0.011	0.395
131	pm4-pm30	0.931	0.97	0.972	0.881	0	0.838
132	pm4-pm31	0.99	1.073	1.045	1.042	-0.011	0.305
133	pm4-pm32	0.993	1.145	1.025	1.007	-0.021	0.258
134	pm4-pm33	1.01	1.054	1.024	1.015	0	0.998
135	pm4-pm34	0.976	1.121	1.055	0.998	0.008	0.474
136	pm4-pm35	0.994	1.077	0.983	0.978	-0.023	0.213
137	pm4-pm36	1.016	1.053	1.02	1.03	0.005	0.852
138	pm4-pm37	1.022	1.062	1.01	1.013	-0.001	0.647
139	pm5-pm6	1.053	0.963	1.108	0.989	-0.006	0.437
140	pm5-pm7	1.968	1.591	2.08	1.585	-0.096	0.013
141	pm5-pm8	0.982	1.011	1.007	0.97	-0.003	0.882
142	pm5-pm9	0.996	1.026	1.023	1.038	-0.012	0.382
143	pm5-pm10	2.128	1.711	1.973	2.205	-0.013	0.339
144	pm5-pm11	1.016	0.975	1.002	0.989	-0.007	0.409

145	pm5-pm12	1.125	1.174	1.016	1.218	-0.048	0.171
146	pm5-pm13	0.977	0.989	0.972	0.97	-0.029	0.202
147	pm5-pm14	1.019	1.024	1.026	0.993	0.015	0.333
148	pm5-pm15	0.987	1.003	1.004	1.018	-0.001	0.965
149	pm5-pm16	1.013	0.998	1.016	0.976	0.003	0.792
150	pm5-pm17	1.015	0.988	1.002	0.978	-0.003	0.75
151	pm5-pm18	0.998	0.985	0.994	0.986	-0.006	0.406
152	pm5-pm19	0.986	1.003	1.007	1.024	0.007	0.448
153	pm5-pm20	0.983	0.973	0.983	0.99	-0.018	0.383
154	pm5-pm21	1.113	1.277	1.361	1.025	-0.065	0.038
155	pm5-pm22	1.083	1.136	1.069	1.009	-0.002	0.677
156	pm5-pm23	1.086	1.208	1.106	1.043	-0.009	0.426
157	pm5-pm24	0.999	0.993	0.988	0.995	-0.008	0.597
158	pm5-pm25	1.008	0.963	0.991	0.986	-0.011	0.342
159	pm5-pm26	1.011	0.981	1.016	0.993	0.003	0.989
160	pm5-pm27	0.995	0.996	1.042	1.012	-0.008	0.583
161	pm5-pm28	1.211	1.031	1.141	1.375	-0.064	0.037
162	pm5-pm29	0.99	1.147	0.953	1.102	-0.027	0.218
163	pm5-pm30	0.909	0.888	0.983	0.864	0	0.92
164	pm5-pm31	1.021	1.03	1.056	1.038	0.015	0.302
165	pm5-pm32	1.001	1.145	1.02	1.022	0.011	0.314
166	pm5-pm33	1.014	0.964	1.009	0.995	-0.003	0.61
167	pm5-pm34	1.024	1.025	1.026	1.005	0.018	0.378
168	pm5-pm35	0.989	0.992	1.016	1.004	-0.003	0.624
169	pm5-pm36	0.998	1.02	1.004	1.022	0.014	0.308
170	pm5-pm37	1.004	1.02	0.986	0.994	0.001	0.731
171	pm6-pm7	1.996	1.567	2.252	1.633	-0.078	0.018
172	pm6-pm8	1.048	1.02	1.092	1.017	0.01	0.328
173	pm6-pm9	1.039	1.03	1.146	1.011	-0.014	0.356
174	pm6-pm10	2.271	1.961	2.317	2.353	0.17	0.005

175	pm6-pm11	1.041	1.013	1.104	1.045	0.009	0.441
176	pm6-pm12	1.141	1.179	1.126	1.241	-0.048	0.158
177	pm6-pm13	1.006	1.035	1.087	1.012	-0.009	0.47
178	pm6-pm14	1.045	1.079	1.11	1.027	0.027	0.27
179	pm6-pm15	1.031	0.994	1.109	1.029	-0.001	0.717
180	pm6-pm16	1.022	1.03	1.105	1.022	0.008	0.533
181	pm6-pm17	1.04	1.014	1.098	0.999	0.001	0.994
182	pm6-pm18	1.026	1.014	1.116	1.009	0.006	0.557
183	pm6-pm19	1.014	1.036	1.09	1.046	0.011	0.331
184	pm6-pm20	1.04	1.024	1.13	0.997	0.01	0.461
185	pm6-pm21	1.107	1.318	1.532	1.042	-0.047	0.17
186	pm6-pm22	1.086	1.139	1.155	1.027	-0.013	0.37
187	pm6-pm23	1.084	1.238	1.235	1.054	-0.005	0.414
188	pm6-pm24	1.023	1.035	1.101	1.019	0.005	0.811
189	pm6-pm25	1.044	1.004	1.1	1.012	0.004	0.979
190	pm6-pm26	1.026	0.99	1.082	1.02	-0.006	0.498
191	pm6-pm27	1.048	1.06	1.139	1.016	0.008	0.548
192	pm6-pm28	1.293	1.1	1.217	1.31	-0.061	0.042
193	pm6-pm29	1.033	1.15	1.103	1.12	-0.012	0.343
194	pm6-pm30	0.92	0.947	1.064	0.914	0.012	0.331
195	pm6-pm31	1.059	1.054	1.084	1.03	-0.003	0.968
196	pm6-pm32	1.039	1.159	1.108	1.052	0.015	0.361
197	pm6-pm33	1.061	1.004	1.121	1.017	0.015	0.368
198	pm6-pm34	1.045	1.007	1.111	0.988	-0.002	0.933
199	pm6-pm35	1.003	1.016	1.102	1.009	-0.009	0.555
200	pm6-pm36	1.048	1.015	1.082	1.018	0.006	0.551
201	pm6-pm37	1.036	1.016	1.1	1.036	0.009	0.529
202	pm7-pm8	1.991	1.622	2.083	1.613	-0.074	0.017
203	pm7-pm9	2.022	1.568	2.121	1.65	-0.098	0.014
204	pm7-pm10	4.003	2.584	3.865	3.347	0.527	<0.001

205	pm7-pm11	1.919	1.627	2.097	1.656	-0.084	0.011
206	pm7-pm12	2.142	1.777	2.156	1.945	-0.082	0.018
207	pm7-pm13	1.928	1.578	2.067	1.581	-0.124	0.008
208	pm7-pm14	2.014	1.541	2.132	1.578	-0.09	0.015
209	pm7-pm15	1.952	1.607	2.067	1.584	-0.107	0.01
210	pm7-pm16	1.975	1.586	2.083	1.595	-0.094	0.017
211	pm7-pm17	2.018	1.536	2.117	1.625	-0.081	0.013
212	pm7-pm18	1.948	1.515	2.021	1.554	-0.143	0.008
213	pm7-pm19	1.947	1.51	2.107	1.587	-0.116	0.006
214	pm7-pm20	2.004	1.584	2.069	1.584	-0.096	0.011
215	pm7-pm21	2.153	1.921	2.781	1.63	-0.043	0.197
216	pm7-pm22	2.1	1.701	2.243	1.663	-0.056	0.023
217	pm7-pm23	2.042	1.813	2.22	1.677	-0.088	0.02
218	pm7-pm24	1.966	1.601	2.12	1.574	-0.092	0.016
219	pm7-pm25	1.974	1.529	2.12	1.59	-0.1	0.01
220	pm7-pm26	1.992	1.594	2.098	1.6	-0.083	0.019
221	pm7-pm27	1.983	1.562	2.125	1.628	-0.101	0.007
222	pm7-pm28	2.464	1.624	2.308	2.199	-0.011	0.387
223	pm7-pm29	2.08	1.834	2.031	1.686	-0.073	0.01
224	pm7-pm30	2.082	1.728	2.372	1.448	0.091	0.012
225	pm7-pm31	1.994	1.703	2.127	1.622	-0.066	0.042
226	pm7-pm32	1.934	1.597	2.054	1.636	-0.136	0.009
227	pm7-pm33	1.961	1.551	2.094	1.593	-0.105	0.006
228	pm7-pm34	1.957	1.576	2.068	1.62	-0.103	0.008
229	pm7-pm35	1.985	1.532	2.08	1.585	-0.114	0.006
230	pm7-pm36	1.959	1.534	2.077	1.583	-0.114	0.005
231	pm7-pm37	1.955	1.544	2.103	1.584	-0.109	0.009
232	pm8-pm9	1.012	1.069	1.036	1.023	0.003	0.647
233	pm8-pm10	2.273	1.981	2.143	2.024	0.089	0.014
234	pm8-pm11	1.018	0.997	0.994	1.012	0.003	0.889

235	pm8-pm12	1.078	1.149	1.034	1.185	-0.069	0.024
236	pm8-pm13	0.98	1.018	0.992	0.951	-0.02	0.394
237	pm8-pm14	0.995	1.065	0.986	0.987	0.008	0.458
238	pm8-pm15	1.023	0.983	1.012	1.017	0.006	0.493
239	pm8-pm16	0.99	1.036	1.005	0.989	0.008	0.4
240	pm8-pm17	0.99	0.994	0.996	0.995	-0.004	0.664
241	pm8-pm18	1.004	0.982	0.985	1.01	-0.001	0.997
242	pm8-pm19	1.004	0.973	0.995	0.99	-0.006	0.501
243	pm8-pm20	0.982	1.018	1.003	0.998	0.001	0.823
244	pm8-pm21	1.118	1.284	1.377	1.004	-0.062	0.023
245	pm8-pm22	1.096	1.138	1.09	1.009	0.007	0.458
246	pm8-pm23	1.054	1.265	1.094	1.053	-0.003	0.922
247	pm8-pm24	0.966	0.992	1.01	0.995	-0.01	0.335
248	pm8-pm25	1.005	0.992	1.001	0.98	-0.002	0.915
249	pm8-pm26	0.999	1.011	1.004	1.001	0.007	0.428
250	pm8-pm27	1.007	1.012	1.037	0.995	-0.006	0.45
251	pm8-pm28	1.221	1.072	1.112	1.338	-0.067	0.031
252	pm8-pm29	1.038	1.172	0.996	1.106	0.004	0.977
253	pm8-pm30	0.948	0.895	0.947	0.923	0.018	0.351
254	pm8-pm31	1.01	1.034	1.046	1.015	0.006	0.412
255	pm8-pm32	1.01	1.125	1.013	1.023	0.008	0.563
256	pm8-pm33	0.997	0.992	0.976	1.018	-0.001	0.983
257	pm8-pm34	0.991	1.03	1.017	0.995	0.007	0.596
258	pm8-pm35	0.998	1.058	0.974	1.012	0.008	0.548
259	pm8-pm36	0.994	1.013	0.99	0.969	-0.005	0.722
260	pm8-pm37	1.012	0.994	0.994	0.993	-0.001	0.844
261	pm9-pm10	2.297	2.032	2.213	2.069	0.1	0.016
262	pm9-pm11	1.012	1.023	1.028	1.023	-0.018	0.364
263	pm9-pm12	1.174	1.159	1.064	1.273	-0.05	0.175
264	pm9-pm13	1.014	1.038	1.031	0.998	-0.022	0.272

265	pm9-pm14	1.043	1.076	1.062	1.022	0.014	0.319
266	pm9-pm15	1.023	1.046	1.078	1.009	-0.001	0.841
267	pm9-pm16	1.069	1.056	1.012	1.042	0.01	0.34
268	pm9-pm17	1.022	1.054	1.066	1.018	0.005	0.978
269	pm9-pm18	1.031	0.996	1.026	1.024	-0.014	0.395
270	pm9-pm19	1.013	1.031	1.008	1.045	-0.009	0.422
271	pm9-pm20	1.007	1.023	1.055	1.051	-0.002	0.888
272	pm9-pm21	1.141	1.328	1.424	1.053	-0.058	0.027
273	pm9-pm22	1.121	1.221	1.125	1.028	0.011	0.312
274	pm9-pm23	1.104	1.288	1.142	1.066	-0.006	0.588
275	pm9-pm24	1.053	1.071	1.021	1.01	0.001	0.725
276	pm9-pm25	1.051	1.034	1.022	1.018	-0.003	0.705
277	pm9-pm26	1.008	1.055	1.029	1.041	0	0.813
278	pm9-pm27	1.02	1.057	1.053	1.019	-0.018	0.305
279	pm9-pm28	1.274	1.122	1.144	1.382	-0.059	0.029
280	pm9-pm29	1.038	1.17	1.014	1.083	-0.035	0.234
281	pm9-pm30	0.955	0.931	0.999	0.892	-0.003	0.723
282	pm9-pm31	1.04	1.121	1.08	1.025	0.009	0.494
283	pm9-pm32	1.027	1.163	1.025	1.063	-0.002	0.947
284	pm9-pm33	1.026	1.075	1.04	1.021	0.007	0.576
285	pm9-pm34	1.06	1.077	1.02	1.019	0.006	0.47
286	pm9-pm35	1.045	1.071	1.044	0.992	-0.001	0.732
287	pm9-pm36	1.039	1.04	1.035	1.02	0.001	0.925
288	pm9-pm37	1.01	1.067	1.023	1.031	-0.003	0.666
289	pm10-pm11	2.315	1.934	2.162	2.112	0.107	0.007
290	pm10-pm12	2.2	1.968	1.937	2.464	-0.06	0.02
291	pm10-pm13	2.293	2.014	2.133	2.04	0.093	0.013
292	pm10-pm14	2.243	1.852	2.046	2.221	0.069	0.03
293	pm10-pm15	2.108	1.905	2.100	2.246	0.065	0.040
294	pm10-pm16	2.022	1.781	1.859	2.023	-0.098	0.012

295	pm10-pm17	2.298	1.953	2.101	2.045	0.079	0.011
296	pm10-pm18	2.012	1.724	1.899	2.087	-0.087	0.014
297	pm10-pm19	2.151	1.914	2.082	2.266	0.085	0.011
298	pm10-pm20	2.323	1.993	2.175	2.055	0.115	0.005
299	pm10-pm21	2.201	2.144	2.567	2.067	-0.035	0.261
300	pm10-pm22	2.065	1.889	1.939	2.106	-0.098	0.019
301	pm10-pm23	2.127	1.941	2.043	2.117	-0.084	0.014
302	pm10-pm24	2.077	1.823	1.986	2.122	-0.021	0.26
303	pm10-pm25	2.119	1.741	2.001	2.133	-0.02	0.287
304	pm10-pm26	2.307	1.922	2.186	2.078	0.105	0.008
305	pm10-pm27	2.057	1.745	1.891	2.093	-0.094	0.016
306	pm10-pm28	2.399	1.709	2.073	2.727	-0.047	0.162
307	pm10-pm29	2.008	1.935	1.889	2.209	-0.085	0.016
308	pm10-pm30	2.115	1.846	2.079	2.062	0.094	0.018
309	pm10-pm31	2.046	1.724	1.889	2.11	-0.1	0.013
310	pm10-pm32	2.187	1.947	2.077	2.333	0.08	0.02
311	pm10-pm33	2.265	1.941	2.167	2	0.074	0.015
312	pm10-pm34	2.29	2.01	2.134	2.042	0.096	0.011
313	pm10-pm35	2.304	1.974	2.12	2.038	0.085	0.016
314	pm10-pm36	2.258	2.018	2.144	2.043	0.098	0.012
315	pm10-pm37	2.294	1.979	2.161	1.995	0.086	0.019
316	pm11-pm12	1.103	1.176	1.028	1.284	-0.04	0.188
317	pm11-pm13	0.99	1.039	1.013	0.985	-0.006	0.564
318	pm11-pm14	1.045	1.056	1.008	1.003	0.021	0.274
319	pm11-pm15	1.02	1.042	1.001	1.023	0.011	0.304
320	pm11-pm16	1.004	1.068	1.023	1.032	0.027	0.214
321	pm11-pm17	0.963	0.987	1.004	0.959	-0.028	0.285
322	pm11-pm18	0.982	0.968	0.999	1.018	-0.012	0.346
323	pm11-pm19	0.984	1.001	1.007	0.983	-0.01	0.389
324	pm11-pm20	0.998	1.029	0.996	0.995	-0.002	0.653

325	pm11-pm21	1.116	1.274	1.339	1.033	-0.075	0.019
326	pm11-pm22	1.045	1.191	1.072	1.04	0.003	0.824
327	pm11-pm23	1.066	1.242	1.115	1.09	0.002	0.985
328	pm11-pm24	0.997	1.045	1.007	0.99	0.001	0.731
329	pm11-pm25	0.992	1.028	1.003	1.015	0.005	0.592
330	pm11-pm26	0.966	1	0.991	1.009	-0.013	0.304
331	pm11-pm27	1.011	1.052	1.03	1.014	0.001	0.821
332	pm11-pm28	1.248	1.057	1.11	1.367	-0.065	0.042
333	pm11-pm29	0.997	1.196	0.976	1.065	-0.023	0.245
334	pm11-pm30	0.942	0.879	0.96	0.928	0.009	0.57
335	pm11-pm31	1.043	1.028	1.014	1.037	0.002	0.934
336	pm11-pm32	1.003	1.114	1.014	1.059	0.005	0.525
337	pm11-pm33	1.018	1.005	0.998	1.006	0.002	0.916
338	pm11-pm34	0.997	1.064	0.992	1	0.005	0.818
339	pm11-pm35	0.975	1.015	1.014	0.989	-0.011	0.309
340	pm11-pm36	1.005	0.975	1.004	1.005	-0.007	0.525
341	pm11-pm37	1.008	0.978	0.985	1.039	-0.005	0.71
342	pm12-pm13	1.081	1.177	0.997	1.25	-0.065	0.022
343	pm12-pm14	1.068	1.2	1.056	1.209	-0.052	0.032
344	pm12-pm15	1.115	1.135	1.031	1.274	-0.05	0.175
345	pm12-pm16	1.113	1.201	1.05	1.256	-0.028	0.287
346	pm12-pm17	1.083	1.229	1.019	1.209	-0.049	0.161
347	pm12-pm18	1.1	1.193	1.019	1.217	-0.05	0.188
348	pm12-pm19	1.078	1.164	1.015	1.214	-0.064	0.024
349	pm12-pm20	1.12	1.134	1.018	1.211	-0.064	0.032
350	pm12-pm21	1.267	1.538	1.424	1.262	-0.071	0.018
351	pm12-pm22	1.156	1.347	1.128	1.259	-0.039	0.204
352	pm12-pm23	1.203	1.423	1.147	1.296	-0.038	0.295
353	pm12-pm24	1.073	1.161	1.047	1.222	-0.061	0.023
354	pm12-pm25	1.086	1.179	1.056	1.225	-0.046	0.156

355	pm12-pm26	1.107	1.153	1.047	1.217	-0.052	0.04
356	pm12-pm27	1.112	1.18	1.058	1.252	-0.054	0.027
357	pm12-pm28	1.37	1.226	1.162	1.627	-0.092	0.019
358	pm12-pm29	1.131	1.428	1.076	1.303	-0.026	0.271
359	pm12-pm30	1.062	1.043	0.939	1.139	-0.05	0.036
360	pm12-pm31	1.135	1.218	1.062	1.241	-0.042	0.175
361	pm12-pm32	1.114	1.311	1.005	1.279	-0.043	0.197
362	pm12-pm33	1.098	1.13	1.029	1.2	-0.069	0.025
363	pm12-pm34	1.122	1.204	1.058	1.173	-0.048	0.174
364	pm12-pm35	1.097	1.213	1.032	1.193	-0.055	0.035
365	pm12-pm36	1.107	1.175	1.027	1.206	-0.053	0.039
366	pm12-pm37	1.093	1.175	1.022	1.224	-0.056	0.023
367	pm13-pm14	0.991	1.019	1.007	1.008	-0.004	0.953
368	pm13-pm15	1.01	0.982	0.999	0.977	-0.021	0.203
369	pm13-pm16	0.981	0.993	1.028	1.006	-0.006	0.476
370	pm13-pm17	0.968	0.998	1.002	0.972	-0.024	0.238
371	pm13-pm18	1.017	1.037	0.967	0.984	-0.005	0.467
372	pm13-pm19	0.996	1.037	1	0.952	-0.011	0.355
373	pm13-pm20	0.991	1.026	1.009	1.022	0.002	0.935
374	pm13-pm21	1.078	1.317	1.386	1.001	-0.073	0.018
375	pm13-pm22	1.035	1.157	1.07	0.997	-0.022	0.284
376	pm13-pm23	1.071	1.23	1.132	1.042	-0.011	0.393
377	pm13-pm24	0.973	1.035	0.989	0.988	-0.015	0.365
378	pm13-pm25	0.957	1.037	1.001	1.016	-0.005	0.686
379	pm13-pm26	0.987	1.037	0.996	0.966	-0.011	0.334
380	pm13-pm27	0.97	1.076	1.009	1.014	-0.012	0.365
381	pm13-pm28	1.241	1.118	1.157	1.358	-0.045	0.196
382	pm13-pm29	0.989	1.131	0.97	1.053	-0.049	0.157
383	pm13-pm30	0.903	0.877	0.924	0.894	-0.021	0.226
384	pm13-pm31	1.017	1.044	1.032	1.015	-0.004	0.946

385	pm13-pm32	0.975	1.14	1.012	1.02	-0.008	0.583
386	pm13-pm33	1.03	1.046	1	1.008	0.013	0.34
387	pm13-pm34	0.989	1.027	0.985	0.989	-0.014	0.338
388	pm13-pm35	0.943	1.04	0.989	0.944	-0.034	0.272
389	pm13-pm36	0.999	1.035	0.993	0.985	-0.004	0.889
390	pm13-pm37	0.991	1.059	1	0.979	-0.003	0.975
391	pm14-pm15	1.022	0.979	1	0.954	-0.019	0.392
392	pm14-pm16	0.987	0.998	0.999	0.975	-0.013	0.356
393	pm14-pm17	1.002	0.998	0.987	0.937	-0.022	0.212
394	pm14-pm18	1.014	1.06	1.032	0.992	0.023	0.296
395	pm14-pm19	1.01	1.028	1.018	0.975	0.006	0.468
396	pm14-pm20	0.994	1.036	1.009	0.979	0	0.681
397	pm14-pm21	1.086	1.315	1.398	1.03	-0.055	0.026
398	pm14-pm22	1.069	1.128	1.109	0.999	-0.005	0.838
399	pm14-pm23	1.075	1.176	1.093	1.019	-0.034	0.221
400	pm14-pm24	0.98	1.019	0.995	1	-0.007	0.512
401	pm14-pm25	1.021	1.027	0.995	0.998	0.008	0.585
402	pm14-pm26	1.011	1.007	1.023	1.011	0.011	0.353
403	pm14-pm27	1.034	1.025	1.044	1.018	0.007	0.439
404	pm14-pm28	1.257	1.138	1.106	1.314	-0.054	0.033
405	pm14-pm29	0.991	1.138	0.977	1.03	-0.045	0.194
406	pm14-pm30	0.897	0.889	0.946	0.898	-0.007	0.441
407	pm14-pm31	1.021	1.089	1.043	1.035	0.021	0.211
408	pm14-pm32	0.998	1.174	0.992	1.031	0.009	0.586
409	pm14-pm33	1.018	1.041	1.01	1	0.015	0.348
410	pm14-pm34	0.996	1.035	0.991	0.965	-0.009	0.403
411	pm14-pm35	1.008	1.006	1.015	0.985	-0.004	0.686
412	pm14-pm36	1.019	1.004	0.993	0.987	0	0.798
413	pm14-pm37	0.99	0.99	1.024	0.99	-0.005	0.401
414	pm15-pm16	1.037	1.006	1.005	1.009	0.009	0.59

415	pm15-pm17	0.978	0.991	1.019	0.986	-0.013	0.358
416	pm15-pm18	0.99	1.011	1.014	0.961	-0.01	0.321
417	pm15-pm19	0.983	1.017	1.044	1.015	0.01	0.39
418	pm15-pm20	1.004	1.045	1.046	0.961	0.006	0.459
419	pm15-pm21	1.114	1.295	1.389	1.036	-0.057	0.037
420	pm15-pm22	1.071	1.136	1.104	1.024	0	0.76
421	pm15-pm23	1.049	1.23	1.082	1.005	-0.036	0.233
422	pm15-pm24	0.979	1.003	1.019	1.005	-0.007	0.453
423	pm15-pm25	1.005	1.033	1.026	0.995	0.01	0.416
424	pm15-pm26	1.014	1.037	1.033	1.003	0.017	0.338
425	pm15-pm27	1.009	1.076	1.028	1.028	0.009	0.439
426	pm15-pm28	1.251	1.104	1.213	1.336	-0.035	0.247
427	pm15-pm29	1	1.116	0.992	1.074	-0.037	0.259
428	pm15-pm30	0.883	0.883	0.989	0.91	-0.002	0.782
429	pm15-pm31	1.031	1.064	1.062	1.022	0.016	0.32
430	pm15-pm32	0.98	1.138	1.024	1.025	-0.001	0.872
431	pm15-pm33	0.959	1.016	1.031	1.001	-0.004	0.885
432	pm15-pm34	1.01	1.01	1.002	0.981	-0.008	0.444
433	pm15-pm35	0.973	0.985	0.995	0.971	-0.03	0.233
434	pm15-pm36	1	1.032	1.01	0.991	0.004	1
435	pm15-pm37	1	1.048	1.017	0.992	0.007	0.591
436	pm16-pm17	0.971	1.021	0.996	0.989	-0.007	0.435
437	pm16-pm18	1.017	0.977	1.038	1.008	0.012	0.328
438	pm16-pm19	1.026	1.001	1.037	0.996	0.016	0.347
439	pm16-pm20	1.01	1.032	1.011	0.966	0.003	0.946
440	pm16-pm21	1.102	1.346	1.39	1.049	-0.039	0.283
441	pm16-pm22	1.038	1.159	1.068	1.024	-0.007	0.447
442	pm16-pm23	1.081	1.23	1.109	1.026	-0.01	0.337
443	pm16-pm24	1.022	1.058	1.027	0.973	0.017	0.351
444	pm16-pm25	1.011	1.063	0.799	0.967	-0.039	0.215

445	pm16-pm26	1.035	1.038	1.002	0.972	0.012	0.365
446	pm16-pm27	1.011	1.082	1.026	0.993	0.007	0.524
447	pm16-pm28	1.215	1.111	1.129	1.382	-0.046	0.184
448	pm16-pm29	1.038	1.161	0.972	1.06	-0.019	0.34
449	pm16-pm30	0.931	0.816	0.956	0.897	-0.013	0.369
450	pm16-pm31	1.001	1.093	1.049	0.996	0.012	0.311
451	pm16-pm32	0.977	1.119	1.031	1.065	0.01	0.311
452	pm16-pm33	1.009	1.037	0.978	0.979	0.001	0.735
453	pm16-pm34	1.024	0.96	1.004	0.984	-0.011	0.328
454	pm16-pm35	0.977	0.984	1.029	1.013	-0.004	0.867
455	pm16-pm36	0.999	0.984	1.015	1	0.001	0.76
456	pm16-pm37	1.008	1.019	1.01	1.024	0.013	0.357
457	pm17-pm18	0.976	0.959	0.984	1.004	-0.019	0.354
458	pm17-pm19	0.98	1.023	1.017	1.004	0.006	0.428
459	pm17-pm20	0.989	1.02	1.008	1.009	0.003	0.72
460	pm17-pm21	1.112	1.258	1.392	1.005	-0.07	0.029
461	pm17-pm22	1.037	1.099	1.07	0.991	-0.031	0.213
462	pm17-pm23	1.058	1.181	1.114	1.029	-0.027	0.242
463	pm17-pm24	1.004	1.021	1.042	0.959	0.002	0.797
464	pm17-pm25	1.008	1.031	0.992	0.998	0.007	0.56
465	pm17-pm26	0.977	1.013	0.983	0.999	-0.008	0.528
466	pm17-pm27	0.984	0.977	1.004	0.989	-0.034	0.294
467	pm17-pm28	1.23	1.101	1.186	1.353	-0.039	0.207
468	pm17-pm29	0.994	1.199	0.961	1.006	-0.038	0.232
469	pm17-pm30	0.915	0.835	0.917	0.881	-0.027	0.287
470	pm17-pm31	1.01	1.016	1.021	1.001	-0.012	0.348
471	pm17-pm32	0.984	1.158	1.023	1.033	0.011	0.324
472	pm17-pm33	1.006	0.993	1.01	0.967	-0.007	0.436
473	pm17-pm34	0.989	0.96	1.007	0.974	-0.022	0.208
474	pm17-pm35	0.986	0.974	1.02	1.003	-0.01	0.513

475	pm17-pm36	1.004	1.01	0.985	0.949	-0.013	0.305
476	pm17-pm37	0.996	1.019	0.991	0.996	-0.002	0.911
477	pm18-pm19	0.982	1.001	0.997	0.999	-0.003	0.865
478	pm18-pm20	1.007	0.977	0.995	1.009	-0.004	0.615
479	pm18-pm21	1.109	1.251	1.391	1.011	-0.069	0.035
480	pm18-pm22	1.067	1.193	1.1	0.993	0.011	0.371
481	pm18-pm23	1.111	1.241	1.12	1.053	0.011	0.358
482	pm18-pm24	0.992	1.038	0.988	1.009	0.005	0.664
483	pm18-pm25	0.988	1.008	1.005	1.004	0.003	0.698
484	pm18-pm26	1.004	0.994	0.987	1.017	0.002	0.639
485	pm18-pm27	1.003	1.054	1.003	1.015	-0.001	0.89
486	pm18-pm28	1.222	1.107	1.13	1.383	-0.044	0.169
487	pm18-pm29	1.006	1.219	0.999	1.04	-0.01	0.555
488	pm18-pm30	0.947	0.869	0.942	0.88	-0.002	0.646
489	pm18-pm31	1.019	1.019	1.026	1.014	-0.003	0.693
490	pm18-pm32	1.008	1.141	0.992	1.037	0.008	0.55
491	pm18-pm33	1.002	1.011	0.982	1	0	0.849
492	pm18-pm34	1.036	1.058	1.008	0.989	0.02	0.222
493	pm18-pm35	0.987	1.009	0.996	0.99	-0.008	0.54
494	pm18-pm36	1.004	1.017	1.004	0.98	0.004	0.838
495	pm18-pm37	1.011	1	1.019	1.029	0.014	0.316
496	pm19-pm20	0.993	1.01	1.005	0.984	-0.003	0.669
497	pm19-pm21	1.125	1.261	1.351	1.014	-0.072	0.015
498	pm19-pm22	1.058	1.182	1.088	1.029	0.012	0.305
499	pm19-pm23	1.067	1.196	1.087	1.056	-0.019	0.347
500	pm19-pm24	0.97	1.023	0.992	0.997	-0.007	0.539
501	pm19-pm25	0.983	0.987	0.996	0.998	-0.008	0.486
502	pm19-pm26	1.009	0.982	0.985	1.013	-0.001	0.746
503	pm19-pm27	1.008	1.007	1.023	1.01	-0.008	0.475
504	pm19-pm28	1.238	1.078	1.137	1.336	-0.057	0.043

505	pm19-pm29	1.044	1.208	1.055	1.055	0.015	0.33
506	pm19-pm30	0.914	0.888	0.954	0.885	-0.002	0.931
507	pm19-pm31	1.017	1.028	1.022	1.01	-0.003	0.989
508	pm19-pm32	0.991	1.128	0.989	1.04	0	0.975
509	pm19-pm33	1.012	0.984	1.002	0.99	-0.002	0.681
510	pm19-pm34	0.969	1.051	0.99	0.997	-0.001	0.612
511	pm19-pm35	1.036	1.026	0.983	0.985	0.004	0.842
512	pm19-pm36	1.006	0.984	1.006	1.009	0.003	0.873
513	pm19-pm37	1.017	0.996	0.993	1.034	0.009	0.513
514	pm20-pm21	1.118	1.222	1.389	1.011	-0.077	0.013
515	pm20-pm22	1.051	1.149	1.099	0.997	-0.006	0.411
516	pm20-pm23	1.089	1.254	1.099	1.054	0	0.938
517	pm20-pm24	1.034	1.014	0.985	0.982	-0.002	0.777
518	pm20-pm25	1.01	0.993	0.991	1.006	-0.002	0.673
519	pm20-pm26	1.006	0.987	0.983	0.991	-0.01	0.478
520	pm20-pm27	1.008	1.048	1.019	1.024	0.002	0.736
521	pm20-pm28	1.234	1.078	1.116	1.394	-0.052	0.041
522	pm20-pm29	1.061	1.149	1.007	1.088	-0.003	0.84
523	pm20-pm30	0.942	0.901	0.931	0.874	-0.003	0.833
524	pm20-pm31	1.014	1.046	1.009	1.015	-0.004	0.99
525	pm20-pm32	1.001	1.133	1.006	1.039	0.005	0.467
526	pm20-pm33	0.984	1.012	0.977	0.996	-0.01	0.596
527	pm20-pm34	1	1.055	1.026	0.993	0.013	0.344
528	pm20-pm35	0.992	1.044	0.989	0.977	-0.007	0.543
529	pm20-pm36	1.003	1.028	0.986	0.997	0.002	0.82
530	pm20-pm37	0.988	0.986	0.989	1.025	-0.007	0.417
531	pm21-pm22	1.194	1.416	1.443	1.061	-0.061	0.023
532	pm21-pm23	1.215	1.516	1.516	1.087	-0.049	0.158
533	pm21-pm24	1.125	1.317	1.382	1.013	-0.054	0.033
534	pm21-pm25	1.119	1.287	1.365	1.02	-0.062	0.036

535	pm21-pm26	1.11	1.3	1.379	1.015	-0.059	0.027
536	pm21-pm27	1.113	1.27	1.358	1.034	-0.088	0.011
537	pm21-pm28	1.347	1.361	1.501	1.37	-0.121	0.005
538	pm21-pm29	1.129	1.496	1.399	1.103	-0.055	0.04
539	pm21-pm30	1.014	1.164	1.342	0.9	-0.068	0.028
540	pm21-pm31	1.133	1.342	1.366	1.03	-0.066	0.043
541	pm21-pm32	1.106	1.459	1.353	1.08	-0.049	0.169
542	pm21-pm33	1.124	1.265	1.358	0.996	-0.074	0.019
543	pm21-pm34	1.137	1.294	1.348	1.027	-0.063	0.037
544	pm21-pm35	1.11	1.272	1.383	1.058	-0.06	0.036
545	pm21-pm36	1.134	1.274	1.392	1.042	-0.049	0.164
546	pm21-pm37	1.121	1.305	1.348	1.01	-0.066	0.045
547	pm22-pm23	1.376	1.555	1.402	1.041	0.143	0.009
548	pm22-pm24	1.073	1.238	1.104	1.017	0.026	0.262
549	pm22-pm25	1.04	1.107	1.102	1.016	-0.012	0.331
550	pm22-pm26	1.04	1.173	1.116	1.03	0.012	0.314
551	pm22-pm27	1.058	1.191	1.108	1.04	-0.001	0.799
552	pm22-pm28	1.291	1.238	1.269	1.414	-0.031	0.239
553	pm22-pm29	1.084	1.291	1.074	1.067	-0.027	0.235
554	pm22-pm30	0.916	1.007	1.036	0.953	-0.013	0.314
555	pm22-pm31	1.052	1.206	1.103	1.02	-0.007	0.583
556	pm22-pm32	1.047	1.282	1.063	1.053	-0.005	0.942
557	pm22-pm33	1.037	1.135	1.071	1.032	-0.009	0.413
558	pm22-pm34	1.043	1.158	1.059	1.047	-0.005	0.451
559	pm22-pm35	1.059	1.09	1.086	1.032	-0.017	0.4
560	pm22-pm36	1.06	1.144	1.079	1.006	-0.005	0.471
561	pm22-pm37	1.063	1.154	1.118	1.024	0.009	0.454
562	pm23-pm24	1.124	1.263	1.09	1.043	0.005	0.416
563	pm23-pm25	1.03	1.275	1.088	1.016	-0.019	0.377
564	pm23-pm26	1.115	1.259	1.074	1.056	0.005	0.827

565	pm23-pm27	1.111	1.277	1.129	1.034	-0.005	0.462
566	pm23-pm28	1.371	1.395	1.245	1.401	-0.024	0.245
567	pm23-pm29	1.061	1.418	1.074	1.086	-0.039	0.259
568	pm23-pm30	0.989	1.119	1.041	0.907	-0.021	0.246
569	pm23-pm31	1.089	1.293	1.117	1.034	-0.012	0.334
570	pm23-pm32	1.07	1.406	1.106	1.083	0.007	0.534
571	pm23-pm33	1.064	1.26	1.132	1.019	-0.003	0.891
572	pm23-pm34	1.048	1.255	1.097	1.032	-0.018	0.343
573	pm23-pm35	1.081	1.218	1.096	1.019	-0.023	0.3
574	pm23-pm36	1.08	1.203	1.13	1.059	-0.003	0.81
575	pm23-pm37	1.037	1.28	1.134	1.039	-0.001	0.979
576	pm24-pm25	0.997	0.986	1.012	0.984	-0.008	0.429
577	pm24-pm26	1	1.052	0.996	0.98	0.004	0.904
578	pm24-pm27	0.992	1.087	1.027	1.015	0.006	0.533
579	pm24-pm28	1.271	1.127	1.17	1.345	-0.03	0.242
580	pm24-pm29	0.992	1.169	0.997	1.052	-0.028	0.254
581	pm24-pm30	0.892	0.932	0.936	0.915	0.003	0.842
582	pm24-pm31	0.999	1.041	1.027	1.009	-0.007	0.445
583	pm24-pm32	1.001	1.173	1.01	1.046	0.016	0.304
584	pm24-pm33	0.978	1.015	1.021	0.991	-0.002	0.799
585	pm24-pm34	1.031	1.021	1.003	0.989	0.004	0.955
586	pm24-pm35	1.022	0.998	1.01	0.985	-0.005	0.839
587	pm24-pm36	0.98	1.028	1.022	1.001	0.006	0.506
588	pm24-pm37	0.992	1.009	0.988	0.993	-0.009	0.439
589	pm25-pm26	1.013	0.986	1.004	0.999	0.002	0.827
590	pm25-pm27	1.006	1.007	1.017	1.027	-0.007	0.467
591	pm25-pm28	1.258	1.036	1.108	1.34	-0.069	0.023
592	pm25-pm29	0.986	1.171	1.008	1.044	-0.024	0.212
593	pm25-pm30	0.913	0.884	0.965	0.925	0.01	0.487
594	pm25-pm31	1.004	1.046	1.029	1.012	0	0.822

595	pm25-pm32	1.014	1.116	0.991	1.038	0.003	0.631
596	pm25-pm33	1.011	0.991	0.981	0.997	-0.004	0.927
597	pm25-pm34	0.996	1.043	0.988	0.995	0.002	0.653
598	pm25-pm35	0.995	1.014	1.01	0.965	-0.008	0.409
599	pm25-pm36	1.005	1.01	1.014	1.014	0.013	0.346
600	pm25-pm37	1.024	0.99	1.003	1.021	0.008	0.588
601	pm26-pm27	1.012	1.06	1.048	1.007	0.011	0.342
602	pm26-pm28	1.219	1.06	1.082	1.382	-0.069	0.036
603	pm26-pm29	0.993	1.149	0.979	1.077	-0.027	0.27
604	pm26-pm30	0.94	0.893	0.954	0.92	0.015	0.31
605	pm26-pm31	1.025	1.052	1.041	1.03	0.014	0.343
606	pm26-pm32	1	1.11	1.013	1.048	0.006	0.592
607	pm26-pm33	1.002	0.983	0.99	1.001	-0.005	0.552
608	pm26-pm34	0.991	1.029	1.027	0.989	0.006	0.561
609	pm26-pm35	0.988	1.067	0.994	0.974	0.001	0.668
610	pm26-pm36	0.988	0.982	0.997	0.988	-0.01	0.589
611	pm26-pm37	0.995	1.003	1.005	0.987	-0.004	0.772
612	pm27-pm28	1.246	1.157	1.133	1.356	-0.053	0.021
613	pm27-pm29	1.011	1.226	0.991	1.074	-0.022	0.242
614	pm27-pm30	0.929	0.933	0.971	0.917	0.004	0.678
615	pm27-pm31	1.009	1.116	1.06	1.01	0.004	0.851
616	pm27-pm32	0.99	1.152	1.02	1.052	-0.005	0.451
617	pm27-pm33	1.003	1.025	1	1.015	-0.01	0.535
618	pm27-pm34	1.037	1.054	1.05	1.003	0.011	0.376
619	pm27-pm35	1.022	1.076	1.015	1.033	0.011	0.306
620	pm27-pm36	1.034	1.042	1.016	0.996	0.002	0.972
621	pm27-pm37	1.02	1.045	1.01	1.017	0	0.909
622	pm28-pm29	1.324	1.22	1.102	1.46	-0.055	0.03
623	pm28-pm30	1.153	0.982	1.098	1.257	-0.046	0.152
624	pm28-pm31	1.253	1.1	1.145	1.382	-0.059	0.043

625	pm28-pm32	1.224	1.188	1.157	1.406	-0.049	0.2
626	pm28-pm33	1.204	1.03	1.127	1.362	-0.074	0.015
627	pm28-pm34	1.238	1.114	1.155	1.359	-0.043	0.195
628	pm28-pm35	1.242	1.094	1.142	1.349	-0.054	0.042
629	pm28-pm36	1.223	1.081	1.148	1.292	-0.068	0.042
630	pm28-pm37	1.211	1.076	1.137	1.37	-0.059	0.021
631	pm29-pm30	1.013	1.277	0.784	0.917	0.008	0.5
632	pm29-pm31	1.019	1.238	1.034	1.075	-0.008	0.507
633	pm29-pm32	1.028	1.333	0.979	1.099	-0.004	0.798
634	pm29-pm33	0.973	1.158	0.984	1.038	-0.038	0.216
635	pm29-pm34	1.007	1.127	0.994	1.028	-0.042	0.169
636	pm29-pm35	0.99	1.09	1	1.052	-0.049	0.163
637	pm29-pm36	1.018	1.18	1.026	1.07	-0.002	0.745
638	pm29-pm37	1.014	1.227	1	1.048	-0.006	0.554
639	pm30-pm31	0.933	0.956	0.999	0.876	0.005	0.599
640	pm30-pm32	0.934	0.97	0.969	0.926	0	0.663
641	pm30-pm33	0.929	0.916	0.94	0.919	0.014	0.363
642	pm30-pm34	0.912	0.924	0.996	0.891	0.014	0.385
643	pm30-pm35	0.949	0.917	0.962	0.892	0.012	0.302
644	pm30-pm36	0.956	0.888	0.944	0.913	0.014	0.359
645	pm30-pm37	0.966	0.898	0.958	0.93	0.024	0.256
646	pm31-pm32	1.013	1.164	1.04	1.043	0.004	0.945
647	pm31-pm33	1.03	0.988	1.026	0.996	-0.013	0.328
648	pm31-pm34	1.002	1.052	1.046	1.003	-0.001	0.705
649	pm31-pm35	0.997	1.067	1.015	0.98	-0.013	0.398
650	pm31-pm36	1.013	1.044	1.057	1.033	0.015	0.359
651	pm31-pm37	1.006	1.056	1.064	1.002	0.007	0.422
652	pm32-pm33	0.983	1.124	1.005	1.008	-0.007	0.533
653	pm32-pm34	0.991	1.194	1.003	1.026	0.012	0.387
654	pm32-pm35	1.007	1.126	1.014	1.034	0.003	0.753

655	pm32-pm36	0.985	1.125	1.018	1.025	0.002	0.713
656	pm32-pm37	1.003	1.152	1.027	1.02	0.012	0.351
657	pm33-pm34	0.977	1.151	0.997	1.022	0.033	0.271
658	pm33-pm35	0.994	1.042	0.99	0.995	0.001	0.785
659	pm33-pm36	1	1.006	0.979	1.004	-0.001	0.748
660	pm33-pm37	1.016	0.981	1.021	1.016	0.007	0.442
661	pm34-pm35	1.009	1.004	1.021	0.979	-0.005	0.563
662	pm34-pm36	1	0.998	1.016	0.994	-0.001	0.971
663	pm34-pm37	1.011	1.041	1.026	0.997	0.013	0.387
664	pm35-pm36	0.986	1.037	1	0.969	-0.006	0.486
665	pm35-pm37	0.992	1.015	0.977	1.008	-0.009	0.474
666	pm36-pm37	1.022	0.994	0.995	1.002	0.003	0.99

3d) Result of two-distribution perturbation analysis for apoptosis in when four different response function types were applied in a combined manner

.		1 million parameter sets		10 million parameter sets		100 million parameter sets	
No	Pair of perturbed parameter distributions	Synergistic effect	p-value	Synergistic effect	p-value	Synergistic effect	p-value
1	pm1-pm2	0.01	0.396	0	0.83	0.022	0.287
2	pm1-pm3	-0.001	0.772	0.01	0.309	0.045	0.161
3	pm1-pm4	0.012	0.364	0.044	0.159	0.026	0.246
4	pm1-pm5	-0.002	0.781	-0.01	0.49	-0.029	0.274
5	pm1-pm6	-0.176	0.002	-0.18	0.003	-0.165	0.004
6	pm1-pm7	-0.158	0.002	-0.173	0.004	-0.183	0.003
7	pm1-pm8	0.013	0.334	0.01	0.325	-0.021	0.284

8	pm1-pm9	0.021	0.272	0.003	0.69	0.012	0.363
9	pm1-pm10	0.11	0.006	0.125	0.008	0.108	0.007
10	pm1-pm11	0.027	0.294	0.02	0.39	-0.005	0.959
11	pm1-pm12	-0.042	0.166	-0.07	0.016	-0.083	0.015
12	pm1-pm13	-0.008	0.509	-0.045	0.165	-0.022	0.287
13	pm1-pm14	0.003	0.785	-0.003	0.745	0	0.92
14	pm1-pm15	0.042	0.199	0.018	0.399	-0.004	0.732
15	pm1-pm16	-0.02	0.285	0.014	0.308	-0.003	0.826
16	pm1-pm17	-0.024	0.215	0.007	0.546	0.013	0.363
17	pm1-pm18	0.006	0.516	-0.017	0.393	0.023	0.238
18	pm1-pm19	-0.029	0.248	-0.016	0.368	-0.037	0.257
19	pm1-pm20	0.009	0.438	0.017	0.347	0.002	0.733
20	pm1-pm21	-0.059	0.021	-0.061	0.021	-0.052	0.023
21	pm1-pm22	-0.016	0.312	-0.006	0.486	0.016	0.331
22	pm1-pm23	-0.272	<0.001	-0.271	<0.001	-0.275	<0.001
23	pm1-pm24	0	0.954	-0.013	0.381	-0.023	0.249
24	pm1-pm25	0.044	0.193	0.039	0.258	0.003	0.601
25	pm1-pm26	0.021	0.214	-0.023	0.278	0.013	0.375
26	pm1-pm27	-0.027	0.245	0.012	0.357	-0.016	0.328
27	pm1-pm28	-0.256	<0.001	-0.259	<0.001	-0.264	<0.001
28	pm1-pm29	-0.014	0.351	-0.02	0.231	-0.029	0.209
29	pm1-pm30	-0.03	0.218	-0.051	0.039	-0.05	0.036
30	pm1-pm31	0.026	0.237	0.026	0.296	-0.001	0.881
31	pm1-pm32	0.007	0.562	-0.022	0.27	-0.022	0.262
32	pm1-pm33	-0.012	0.395	-0.013	0.34	-0.004	0.835
33	pm1-pm34	0.002	0.68	0.022	0.229	0.004	0.707
34	pm1-pm35	0.022	0.297	0.015	0.357	0.038	0.227
35	pm1-pm36	-0.019	0.384	-0.002	0.715	0.011	0.31
36	pm1-pm37	-0.018	0.397	0.005	0.654	-0.034	0.209
37	pm2-pm3	0.011	0.343	0.009	0.437	0.023	0.254

38	pm2-pm4	-0.002	0.686	-0.031	0.249	-0.027	0.208
39	pm2-pm5	-0.016	0.357	0.008	0.474	-0.024	0.219
40	pm2-pm6	0.013	0.351	0.01	0.403	0.01	0.302
41	pm2-pm7	-0.017	0.354	-0.048	0.192	-0.039	0.297
42	pm2-pm8	0.012	0.302	-0.026	0.258	0.015	0.308
43	pm2-pm9	0.039	0.223	0.041	0.175	0.043	0.176
44	pm2-pm10	0.113	0.009	0.096	0.017	0.117	0.010
45	pm2-pm11	0.007	0.577	-0.024	0.276	-0.019	0.348
46	pm2-pm12	-0.062	0.038	-0.058	0.038	-0.04	0.157
47	pm2-pm13	-0.035	0.221	-0.039	0.257	0.006	0.547
48	pm2-pm14	-0.001	0.659	0.007	0.523	-0.03	0.291
49	pm2-pm15	0.036	0.299	-0.003	0.929	0	0.933
50	pm2-pm16	0.009	0.492	0.008	0.436	0.04	0.247
51	pm2-pm17	-0.013	0.375	0.005	0.781	-0.004	0.845
52	pm2-pm18	-0.019	0.394	-0.013	0.31	-0.005	0.432
53	pm2-pm19	-0.019	0.308	0.008	0.569	0.019	0.391
54	pm2-pm20	-0.015	0.368	-0.024	0.277	0	0.729
55	pm2-pm21	-0.123	0.01	-0.081	0.019	-0.101	0.01
56	pm2-pm22	0.002	0.724	0.021	0.214	0.028	0.252
57	pm2-pm23	0.009	0.491	0.003	0.945	0.01	0.429
58	pm2-pm24	-0.03	0.252	-0.021	0.28	0.005	0.714
59	pm2-pm25	0.007	0.449	-0.012	0.349	-0.023	0.232
60	pm2-pm26	-0.015	0.38	0.021	0.22	0.012	0.314
61	pm2-pm27	0.022	0.251	-0.007	0.528	0.024	0.295
62	pm2-pm28	-0.494	<0.001	-0.448	<0.001	-0.474	<0.001
63	pm2-pm29	0.019	0.35	-0.017	0.373	0.003	0.891
64	pm2-pm30	-0.03	0.283	-0.039	0.298	-0.029	0.217
65	pm2-pm31	-0.024	0.283	0.009	0.567	-0.005	0.56
66	pm2-pm32	0.006	0.575	0.004	0.722	-0.012	0.325
67	pm2-pm33	-0.023	0.226	-0.025	0.209	-0.013	0.367

68	pm2-pm34	-0.032	0.296	-0.012	0.34	-0.039	0.275
69	pm2-pm35	-0.01	0.463	-0.008	0.489	-0.004	0.863
70	pm2-pm36	0.016	0.311	0.019	0.328	0.01	0.472
71	pm2-pm37	0.017	0.351	-0.015	0.339	0.021	0.289
72	pm3-pm4	-0.01	0.417	-0.003	0.787	0.023	0.26
73	pm3-pm5	0.022	0.246	-0.018	0.319	-0.004	0.962
74	pm3-pm6	-0.143	0.008	-0.152	0.005	-0.149	0.006
75	pm3-pm7	-0.128	0.009	-0.167	0.004	-0.152	0.003
76	pm3-pm8	0.004	0.843	0.01	0.508	0.026	0.236
77	pm3-pm9	-0.457	<0.001	-0.452	<0.001	-0.452	<0.001
78	pm3-pm10	0.101	0.006	0.103	0.008	0.111	0.009
79	pm3-pm11	0	0.958	0.03	0.229	0.029	0.233
80	pm3-pm12	-0.082	0.012	-0.073	0.012	-0.086	0.019
81	pm3-pm13	-0.001	0.723	0.006	0.434	-0.041	0.177
82	pm3-pm14	0	0.674	0.017	0.353	-0.022	0.254
83	pm3-pm15	-0.01	0.387	0.02	0.307	-0.013	0.315
84	pm3-pm16	-0.018	0.347	0	0.651	0.019	0.396
85	pm3-pm17	-0.025	0.26	-0.012	0.327	0.001	0.867
86	pm3-pm18	-0.009	0.438	-0.003	0.832	0.004	0.89
87	pm3-pm19	-0.007	0.426	0.013	0.348	-0.012	0.315
88	pm3-pm20	0.023	0.254	-0.003	0.656	-0.012	0.384
89	pm3-pm21	-0.085	0.018	-0.09	0.014	-0.077	0.016
90	pm3-pm22	-0.164	0.005	-0.136	0.006	-0.138	0.009
91	pm3-pm23	0.026	0.263	0.042	0.198	0.004	0.849
92	pm3-pm24	0.026	0.2	-0.012	0.329	-0.009	0.437
93	pm3-pm25	0.004	0.697	-0.01	0.501	0.02	0.32
94	pm3-pm26	0.039	0.285	-0.001	0.7	0.009	0.42
95	pm3-pm27	-0.009	0.508	0.013	0.315	-0.011	0.398
96	pm3-pm28	-0.48	<0.001	-0.49	<0.001	-0.485	<0.001
97	pm3-pm29	-0.054	0.035	-0.06	0.022	-0.039	0.258

98	pm3-pm30	-0.002	0.843	-0.042	0.185	-0.01	0.357
99	pm3-pm31	-0.473	<0.001	-0.497	<0.001	-0.491	<0.001
100	pm3-pm32	-0.003	0.711	0.004	0.655	-0.004	0.716
101	pm3-pm33	0.026	0.262	-0.005	0.643	0.034	0.256
102	pm3-pm34	-0.108	0.007	-0.076	0.016	-0.118	0.007
103	pm3-pm35	-0.003	0.849	0.005	0.665	-0.011	0.397
104	pm3-pm36	0.003	0.674	-0.002	0.761	0.001	0.669
105	pm3-pm37	-0.001	0.892	0.015	0.397	0.026	0.213
106	pm4-pm5	0.021	0.213	0.012	0.394	0.012	0.347
107	pm4-pm6	-0.018	0.309	-0.012	0.325	-0.017	0.305
108	pm4-pm7	-0.092	0.018	-0.103	0.009	-0.079	0.013
109	pm4-pm8	-0.019	0.335	-0.015	0.334	0.002	0.614
110	pm4-pm9	0	0.915	0.021	0.225	-0.009	0.486
111	pm4-pm10	0.043	0.177	-0.002	0.633	0.004	0.686
112	pm4-pm11	-0.001	0.905	0.009	0.441	0.013	0.307
113	pm4-pm12	-0.056	0.023	-0.095	0.012	-0.081	0.011
114	pm4-pm13	-0.009	0.491	0.001	0.968	-0.01	0.304
115	pm4-pm14	0.029	0.276	-0.012	0.363	0.007	0.453
116	pm4-pm15	0.028	0.283	-0.007	0.566	0.003	0.621
117	pm4-pm16	0.038	0.27	0.021	0.224	0.017	0.341
118	pm4-pm17	-0.023	0.29	0.003	0.917	-0.003	0.766
119	pm4-pm18	-0.004	0.937	0.005	0.658	0.002	0.755
120	pm4-pm19	0.013	0.306	0.001	0.845	-0.006	0.426
121	pm4-pm20	-0.017	0.343	0.018	0.348	0.025	0.285
122	pm4-pm21	-0.06	0.03	-0.101	0.005	-0.082	0.016
123	pm4-pm22	0.007	0.562	-0.016	0.336	0.012	0.339
124	pm4-pm23	-0.225	<0.001	-0.225	<0.001	-0.247	<0.001
125	pm4-pm24	0.014	0.339	-0.019	0.363	-0.002	0.817
126	pm4-pm25	-0.005	0.478	-0.005	0.679	-0.016	0.323
127	pm4-pm26	0.024	0.285	0.01	0.378	0.024	0.259

128	pm4-pm27	0.037	0.215	0.024	0.229	0.033	0.273
129	pm4-pm28	-0.149	0.005	-0.153	0.003	-0.117	0.008
130	pm4-pm29	0.025	0.269	0.023	0.255	0.026	0.276
131	pm4-pm30	-0.013	0.393	0.013	0.375	0.014	0.354
132	pm4-pm31	-0.001	0.712	-0.033	0.293	-0.012	0.332
133	pm4-pm32	0.019	0.365	-0.021	0.249	0.012	0.305
134	pm4-pm33	0.013	0.345	-0.007	0.574	0.016	0.367
135	pm4-pm34	-0.031	0.24	-0.009	0.552	0.011	0.363
136	pm4-pm35	-0.007	0.554	0.028	0.261	-0.003	0.83
137	pm4-pm36	0	0.823	-0.016	0.301	0.012	0.334
138	pm4-pm37	0.004	0.704	-0.011	0.316	-0.01	0.346
139	pm5-pm6	-0.155	0.003	-0.165	0.001	-0.18	0.003
140	pm5-pm7	-0.264	<0.001	-0.235	<0.001	-0.222	<0.001
141	pm5-pm8	0.003	0.93	0	0.839	0.023	0.21
142	pm5-pm9	-0.023	0.284	-0.005	0.632	-0.004	0.916
143	pm5-pm10	-0.119	0.009	-0.13	0.006	-0.12	0.009
144	pm5-pm11	0.012	0.347	-0.013	0.392	0.012	0.37
145	pm5-pm12	-0.051	0.02	-0.047	0.185	-0.044	0.161
146	pm5-pm13	-0.006	0.424	-0.008	0.55	-0.032	0.249
147	pm5-pm14	-0.012	0.317	0.033	0.23	0.029	0.235
148	pm5-pm15	-0.003	0.849	-0.012	0.369	-0.018	0.353
149	pm5-pm16	0.011	0.382	0.024	0.255	0.002	0.637
150	pm5-pm17	0.015	0.302	0.046	0.154	0.016	0.375
151	pm5-pm18	0.027	0.286	-0.012	0.347	0.004	0.84
152	pm5-pm19	-0.003	0.927	0.017	0.33	-0.001	0.772
153	pm5-pm20	-0.007	0.511	-0.004	0.827	-0.036	0.216
154	pm5-pm21	-0.112	0.009	-0.086	0.013	-0.109	0.007
155	pm5-pm22	-0.154	0.003	-0.158	0.004	-0.149	0.006
156	pm5-pm23	0.011	0.349	0.035	0.283	0.036	0.206
157	pm5-pm24	0.002	0.836	0.014	0.314	-0.025	0.229

158	pm5-pm25	-0.024	0.269	0.022	0.221	-0.013	0.357
159	pm5-pm26	0.001	0.77	-0.001	0.876	0.01	0.341
160	pm5-pm27	-0.015	0.306	-0.018	0.354	0.011	0.397
161	pm5-pm28	-0.168	0.005	-0.167	0.002	-0.194	0.003
162	pm5-pm29	-0.024	0.204	-0.003	0.951	-0.031	0.288
163	pm5-pm30	-0.042	0.167	-0.015	0.317	-0.028	0.208
164	pm5-pm31	0.018	0.317	0.033	0.256	0.025	0.225
165	pm5-pm32	-0.005	0.948	-0.022	0.24	-0.019	0.359
166	pm5-pm33	-0.014	0.307	0.002	0.712	0.028	0.226
167	pm5-pm34	0.031	0.222	0.017	0.37	0.023	0.28
168	pm5-pm35	-0.013	0.397	0.008	0.545	0.024	0.235
169	pm5-pm36	-0.018	0.37	-0.024	0.224	0.02	0.358
170	pm5-pm37	0.017	0.374	0	0.67	0.028	0.291
171	pm6-pm7	-0.014	0.317	-0.038	0.211	-0.025	0.282
172	pm6-pm8	0.016	0.373	0.014	0.393	0.018	0.378
173	pm6-pm9	0.018	0.332	0.025	0.287	0.005	0.607
174	pm6-pm10	0.196	0.004	0.211	<0.001	0.185	0.002
175	pm6-pm11	0.031	0.251	0.013	0.359	-0.004	0.867
176	pm6-pm12	-0.092	0.012	-0.052	0.04	-0.056	0.03
177	pm6-pm13	0.011	0.302	-0.02	0.3	0.001	0.617
178	pm6-pm14	0.007	0.484	0.009	0.486	0.039	0.299
179	pm6-pm15	0.004	0.986	0.036	0.21	-0.006	0.478
180	pm6-pm16	0.014	0.377	-0.487	<0.001	-0.473	<0.001
181	pm6-pm17	0.026	0.279	0.041	0.184	0.03	0.223
182	pm6-pm18	-0.46	<0.001	-0.486	<0.001	-0.464	<0.001
183	pm6-pm19	0.023	0.286	0.004	0.672	0.025	0.24
184	pm6-pm20	-0.268	<0.001	-0.253	<0.001	-0.274	<0.001
185	pm6-pm21	-0.083	0.012	-0.121	0.01	-0.105	0.008
186	pm6-pm22	-0.436	<0.001	-0.441	<0.001	-0.485	<0.001
187	pm6-pm23	0.01	0.32	0.028	0.202	0.003	0.653

188	pm6-pm24	0.028	0.241	0.03	0.217	0.009	0.55
189	pm6-pm25	0.01	0.506	-0.002	0.768	0.021	0.216
190	pm6-pm26	0.01	0.322	0.004	0.643	0.026	0.229
191	pm6-pm27	-0.226	<0.001	-0.214	<0.001	-0.194	0.001
192	pm6-pm28	-0.202	<0.001	-0.237	<0.001	-0.232	<0.001
193	pm6-pm29	-0.028	0.264	-0.014	0.358	-0.011	0.311
194	pm6-pm30	-0.027	0.29	-0.037	0.212	-0.003	0.61
195	pm6-pm31	-0.249	<0.001	-0.248	<0.001	-0.254	<0.001
196	pm6-pm32	-0.263	<0.001	-0.243	<0.001	-0.259	<0.001
197	pm6-pm33	-0.285	<0.001	-0.262	<0.001	-0.262	<0.001
198	pm6-pm34	0.014	0.338	-0.173	0.004	0.049	0.178
199	pm6-pm35	-0.02	0.287	0.017	0.341	-0.01	0.376
200	pm6-pm36	0.034	0.293	0.034	0.245	0.038	0.285
201	pm6-pm37	-0.035	0.281	-0.043	0.166	-0.044	0.198
202	pm7-pm8	-0.015	0.327	-0.03	0.238	-0.036	0.226
203	pm7-pm9	0.005	0.497	-0.022	0.274	0.007	0.515
204	pm7-pm10	0.482	<0.001	0.463	<0.001	0.454	<0.001
205	pm7-pm11	-0.082	0.019	-0.054	0.027	-0.066	0.025
206	pm7-pm12	-0.048	0.183	-0.057	0.023	-0.059	0.02
207	pm7-pm13	-0.056	0.043	-0.076	0.018	-0.077	0.018
208	pm7-pm14	0.004	0.958	0.007	0.452	-0.019	0.352
209	pm7-pm15	-0.023	0.203	-0.029	0.216	-0.033	0.292
210	pm7-pm16	0	0.717	-0.002	0.721	-0.03	0.264
211	pm7-pm17	0.021	0.262	-0.009	0.538	0.013	0.341
212	pm7-pm18	-0.041	0.165	-0.045	0.188	-0.043	0.158
213	pm7-pm19	-0.043	0.173	-0.067	0.022	-0.033	0.257
214	pm7-pm20	-0.019	0.376	0.009	0.437	-0.014	0.335
215	pm7-pm21	-0.074	0.014	-0.06	0.03	-0.072	0.015
216	pm7-pm22	-0.132	0.006	-0.162	0.004	-0.149	0.009
217	pm7-pm23	0.009	0.429	0.001	0.765	0.012	0.347

218	pm7-pm24	-0.031	0.27	-0.014	0.349	-0.014	0.341
219	pm7-pm25	-0.044	0.194	-0.057	0.035	-0.025	0.2
220	pm7-pm26	-0.004	0.667	-0.008	0.451	-0.009	0.588
221	pm7-pm27	0.001	0.924	-0.029	0.248	-0.007	0.598
222	pm7-pm28	-0.431	<0.001	-0.461	<0.001	-0.464	<0.001
223	pm7-pm29	-0.131	0.007	-0.137	0.008	-0.124	0.009
224	pm7-pm30	0.09	0.019	0.078	0.015	0.079	0.011
225	pm7-pm31	-0.02	0.323	-0.023	0.291	-0.031	0.295
226	pm7-pm32	-0.061	0.04	-0.035	0.29	-0.064	0.037
227	pm7-pm33	-0.054	0.021	-0.027	0.266	-0.069	0.029
228	pm7-pm34	-0.044	0.176	-0.078	0.01	-0.041	0.163
229	pm7-pm35	-0.042	0.169	-0.038	0.208	-0.004	0.836
230	pm7-pm36	-0.043	0.183	-0.022	0.244	-0.035	0.286
231	pm7-pm37	-0.034	0.221	-0.056	0.031	-0.05	0.043
232	pm8-pm9	-0.027	0.208	-0.015	0.324	-0.014	0.309
233	pm8-pm10	0.097	0.015	0.108	0.010	0.12	0.009
234	pm8-pm11	-0.018	0.307	0.021	0.298	-0.014	0.39
235	pm8-pm12	-0.069	0.022	-0.098	0.015	-0.069	0.025
236	pm8-pm13	-0.003	0.772	-0.028	0.2	-0.009	0.498
237	pm8-pm14	0.003	0.906	-0.029	0.257	0.018	0.384
238	pm8-pm15	0.03	0.25	0.012	0.378	0.033	0.255
239	pm8-pm16	-0.004	0.887	0.005	0.971	-0.011	0.392
240	pm8-pm17	0.019	0.392	-0.019	0.376	0.011	0.349
241	pm8-pm18	-0.015	0.374	-0.02	0.307	-0.008	0.583
242	pm8-pm19	0.026	0.228	0.026	0.296	-0.013	0.315
243	pm8-pm20	0.005	0.507	0.018	0.362	-0.002	0.687
244	pm8-pm21	-0.086	0.017	-0.092	0.017	-0.089	0.016
245	pm8-pm22	-0.372	<0.001	-0.362	<0.001	-0.373	<0.001
246	pm8-pm23	0.011	0.329	0.017	0.346	-0.017	0.334
247	pm8-pm24	-0.009	0.521	-0.029	0.254	0.009	0.438

248	pm8-pm25	-0.018	0.35	0.013	0.349	0.018	0.373
249	pm8-pm26	0.015	0.305	-0.001	0.873	-0.007	0.581
250	pm8-pm27	0.013	0.364	0.014	0.308	-0.025	0.258
251	pm8-pm28	-0.468	<0.001	-0.477	<0.001	-0.448	<0.001
252	pm8-pm29	0.005	0.671	0.016	0.305	-0.006	0.441
253	pm8-pm30	0.015	0.324	-0.03	0.222	-0.005	0.672
254	pm8-pm31	0.003	0.644	-0.022	0.211	0.01	0.37
255	pm8-pm32	-0.011	0.346	0.006	0.544	0.013	0.36
256	pm8-pm33	0.021	0.283	0.012	0.345	0.03	0.298
257	pm8-pm34	-0.011	0.336	-0.005	0.491	-0.008	0.428
258	pm8-pm35	-0.009	0.452	-0.005	0.535	0.006	0.545
259	pm8-pm36	-0.011	0.305	-0.001	0.775	0.013	0.388
260	pm8-pm37	-0.007	0.507	-0.014	0.335	0.024	0.218
261	pm9-pm10	0.113	0.009	0.098	0.018	0.115	0.007
262	pm9-pm11	0.021	0.293	0.01	0.358	-0.006	0.553
263	pm9-pm12	-0.041	0.161	-0.054	0.034	-0.02	0.343
264	pm9-pm13	-0.017	0.396	-0.002	0.898	-0.004	0.987
265	pm9-pm14	0.042	0.188	0.012	0.328	-0.003	0.937
266	pm9-pm15	0.012	0.392	0	0.967	0.007	0.555
267	pm9-pm16	-0.063	0.021	-0.076	0.018	-0.081	0.015
268	pm9-pm17	0.01	0.556	-0.013	0.371	-0.001	0.617
269	pm9-pm18	0.019	0.328	0.021	0.223	0.017	0.333
270	pm9-pm19	-0.022	0.23	0.014	0.361	0.006	0.578
271	pm9-pm20	-0.012	0.34	-0.022	0.299	0.022	0.282
272	pm9-pm21	-0.076	0.019	-0.119	0.005	-0.078	0.011
273	pm9-pm22	-0.041	0.176	-0.054	0.039	-0.058	0.023
274	pm9-pm23	0.004	0.626	0.027	0.213	0.041	0.163
275	pm9-pm24	-0.47	<0.001	-0.487	<0.001	-0.465	<0.001
276	pm9-pm25	0.022	0.274	0.021	0.274	0.035	0.204
277	pm9-pm26	-0.01	0.387	0.001	0.899	0.003	0.625

278	pm9-pm27	-0.021	0.239	-0.002	0.825	0.012	0.315
279	pm9-pm28	-0.039	0.224	-0.037	0.24	-0.045	0.17
280	pm9-pm29	-0.016	0.333	-0.035	0.261	-0.034	0.249
281	pm9-pm30	-0.03	0.261	-0.006	0.517	-0.031	0.239
282	pm9-pm31	0.029	0.282	0.021	0.284	0.021	0.209
283	pm9-pm32	0.006	0.477	0.03	0.218	0.016	0.397
284	pm9-pm33	0.016	0.366	-0.017	0.369	0.028	0.289
285	pm9-pm34	0.002	0.669	0.033	0.242	0.018	0.318
286	pm9-pm35	0.024	0.238	0.001	0.779	-0.003	0.73
287	pm9-pm36	0.012	0.343	0.028	0.218	0.011	0.36
288	pm9-pm37	0.009	0.464	-0.002	0.828	-0.012	0.359
289	pm10-pm11	0.149	0.005	0.163	0.002	0.169	0.004
290	pm10-pm12	-0.136	0.009	-0.157	0.003	-0.141	0.007
291	pm10-pm13	0.119	0.007	0.107	0.005	0.128	0.009
292	pm10-pm14	0.091	0.013	0.093	0.011	0.108	0.009
293	pm10-pm15	0.065	0.026	0.071	0.013	0.07	0.032
294	pm10-pm16	-0.143	0.006	-0.142	0.007	-0.149	0.01
295	pm10-pm17	0.091	0.014	0.084	0.010	0.082	0.018
296	pm10-pm18	-0.17	0.003	-0.176	0.001	-0.145	0.009
297	pm10-pm19	0.09	0.019	0.086	0.014	0.104	0.010
298	pm10-pm20	0.092	0.012	0.091	0.014	0.105	0.006
299	pm10-pm21	-0.182	0.004	-0.151	0.002	-0.158	0.003
300	pm10-pm22	-0.146	0.008	-0.157	0.003	-0.152	0.002
301	pm10-pm23	-0.082	0.02	-0.107	0.01	-0.093	0.015
302	pm10-pm24	0.048	0.2	0.029	0.214	0.016	0.339
303	pm10-pm25	-0.128	0.006	-0.118	0.006	-0.14	0.008
304	pm10-pm26	0.066	0.031	0.068	0.031	0.073	0.011
305	pm10-pm27	-0.099	0.014	-0.142	0.009	-0.128	0.005
306	pm10-pm28	-0.165	0.002	-0.159	0.001	-0.131	0.008
307	pm10-pm29	-0.258	<0.001	-0.18	0.005	-0.182	0.002

308	pm10-pm30	0.118	0.008	0.112	0.006	0.138	0.008
309	pm10-pm31	-0.11	0.007	-0.141	0.01	-0.114	0.006
310	pm10-pm32	0.08	0.014	0.09	0.015	0.081	0.017
311	pm10-pm33	0.057	0.029	0.066	0.026	0.058	0.029
312	pm10-pm34	0.171	0.003	0.165	0.004	0.185	0.004
313	pm10-pm35	0.109	0.009	0.109	0.007	0.094	0.015
314	pm10-pm36	0.054	0.039	0.054	0.037	0.068	0.039
315	pm10-pm37	0.319	<0.001	0.33	<0.001	0.331	<0.001
316	pm11-pm12	-0.071	0.014	-0.051	0.025	-0.093	0.012
317	pm11-pm13	-0.034	0.291	-0.024	0.29	-0.026	0.246
318	pm11-pm14	0.017	0.36	0.024	0.244	0.038	0.255
319	pm11-pm15	-0.021	0.21	-0.008	0.421	-0.004	0.826
320	pm11-pm16	0.018	0.324	0.005	0.677	0.02	0.371
321	pm11-pm17	-0.035	0.278	-0.009	0.584	-0.009	0.438
322	pm11-pm18	-0.002	0.966	0.004	0.925	-0.01	0.321
323	pm11-pm19	-0.031	0.247	-0.031	0.204	-0.012	0.375
324	pm11-pm20	-0.017	0.341	-0.023	0.239	-0.011	0.393
325	pm11-pm21	-0.133	0.006	-0.121	0.009	-0.129	0.006
326	pm11-pm22	-0.004	0.981	0.004	0.771	0.022	0.201
327	pm11-pm23	0.023	0.205	0.011	0.32	0.012	0.309
328	pm11-pm24	-0.018	0.34	0.02	0.216	-0.012	0.334
329	pm11-pm25	0.024	0.291	-0.001	0.682	-0.019	0.376
330	pm11-pm26	-0.011	0.306	-0.03	0.288	-0.015	0.354
331	pm11-pm27	0.013	0.366	0.001	0.63	0.003	0.616
332	pm11-pm28	-0.255	<0.001	-0.222	<0.001	-0.235	<0.001
333	pm11-pm29	0.004	0.995	-0.01	0.404	-0.018	0.377
334	pm11-pm30	-0.005	0.969	-0.038	0.236	-0.032	0.291
335	pm11-pm31	0.039	0.207	0.027	0.207	0.012	0.354
336	pm11-pm32	-0.01	0.443	-0.025	0.277	0.015	0.306
337	pm11-pm33	-0.016	0.39	0.028	0.24	0.011	0.312

338	pm11-pm34	0.015	0.334	-0.018	0.332	0.008	0.453
339	pm11-pm35	-0.001	0.695	-0.004	0.881	0.002	0.725
340	pm11-pm36	0.004	0.995	0.013	0.381	0.007	0.423
341	pm11-pm37	-0.02	0.217	0.009	0.473	-0.012	0.393
342	pm12-pm13	-0.081	0.015	-0.087	0.011	-0.079	0.013
343	pm12-pm14	-0.09	0.01	-0.076	0.014	-0.07	0.039
344	pm12-pm15	-0.094	0.013	-0.091	0.016	-0.068	0.02
345	pm12-pm16	-0.064	0.039	-0.066	0.038	-0.03	0.267
346	pm12-pm17	-0.082	0.018	-0.08	0.013	-0.105	0.01
347	pm12-pm18	-0.065	0.039	-0.069	0.028	-0.098	0.011
348	pm12-pm19	-0.105	0.007	-0.093	0.014	-0.068	0.031
349	pm12-pm20	-0.082	0.012	-0.032	0.296	-0.052	0.038
350	pm12-pm21	-0.154	0.003	-0.116	0.007	-0.132	0.009
351	pm12-pm22	-0.043	0.195	-0.046	0.169	-0.054	0.033
352	pm12-pm23	-0.049	0.162	-0.046	0.168	-0.052	0.039
353	pm12-pm24	-0.086	0.013	-0.105	0.006	-0.097	0.011
354	pm12-pm25	-0.059	0.04	-0.058	0.03	-0.092	0.011
355	pm12-pm26	-0.055	0.039	-0.051	0.044	-0.077	0.011
356	pm12-pm27	-0.091	0.012	-0.104	0.008	-0.067	0.043
357	pm12-pm28	0.006	0.413	0.019	0.334	0.021	0.262
358	pm12-pm29	-0.058	0.031	-0.069	0.027	-0.048	0.165
359	pm12-pm30	-0.061	0.026	-0.057	0.039	-0.096	0.019
360	pm12-pm31	-0.038	0.252	-0.043	0.166	-0.064	0.036
361	pm12-pm32	-0.055	0.024	-0.079	0.01	-0.083	0.019
362	pm12-pm33	-0.109	0.008	-0.072	0.011	-0.107	0.009
363	pm12-pm34	-0.029	0.248	-0.045	0.191	-0.065	0.032
364	pm12-pm35	-0.082	0.013	-0.096	0.019	-0.108	0.008
365	pm12-pm36	-0.065	0.035	-0.071	0.017	-0.062	0.033
366	pm12-pm37	-0.051	0.033	-0.079	0.016	-0.072	0.019
367	pm13-pm14	0.004	0.754	0.008	0.536	0.017	0.39

368	pm13-pm15	-0.03	0.256	-0.036	0.291	0	0.959
369	pm13-pm16	-0.022	0.246	-0.008	0.569	-0.015	0.399
370	pm13-pm17	-0.012	0.335	-0.04	0.153	-0.017	0.328
371	pm13-pm18	-0.022	0.218	-0.01	0.414	0.012	0.345
372	pm13-pm19	-0.012	0.33	-0.013	0.377	-0.017	0.304
373	pm13-pm20	0.022	0.202	0.016	0.353	-0.016	0.363
374	pm13-pm21	-0.106	0.008	-0.127	0.007	-0.142	0.008
375	pm13-pm22	-0.031	0.267	-0.032	0.247	-0.009	0.552
376	pm13-pm23	0.005	0.932	0.014	0.379	0.027	0.272
377	pm13-pm24	-0.028	0.252	-0.031	0.266	-0.023	0.278
378	pm13-pm25	-0.013	0.325	-0.03	0.282	-0.026	0.252
379	pm13-pm26	-0.005	0.775	-0.013	0.308	-0.007	0.59
380	pm13-pm27	-0.029	0.209	-0.03	0.279	-0.05	0.154
381	pm13-pm28	-0.25	<0.001	-0.251	<0.001	-0.281	<0.001
382	pm13-pm29	-0.047	0.159	-0.032	0.238	-0.051	0.029
383	pm13-pm30	-0.038	0.253	-0.029	0.252	-0.054	0.035
384	pm13-pm31	-0.023	0.268	-0.007	0.411	-0.021	0.204
385	pm13-pm32	-0.026	0.214	-0.026	0.25	-0.003	0.72
386	pm13-pm33	0.034	0.254	0.022	0.254	0.015	0.397
387	pm13-pm34	0.014	0.384	-0.008	0.466	-0.02	0.291
388	pm13-pm35	-0.038	0.28	-0.069	0.037	-0.038	0.249
389	pm13-pm36	-0.009	0.494	-0.011	0.352	-0.005	0.553
390	pm13-pm37	0.009	0.403	-0.006	0.503	0.005	0.858
391	pm14-pm15	0.002	0.671	0.003	0.981	-0.01	0.495
392	pm14-pm16	-0.031	0.286	-0.011	0.378	-0.001	0.628
393	pm14-pm17	-0.008	0.5	-0.026	0.215	0.003	0.703
394	pm14-pm18	0.034	0.222	-0.002	0.965	0.009	0.587
395	pm14-pm19	-0.003	0.86	0.027	0.222	0.032	0.264
396	pm14-pm20	0.013	0.356	0.011	0.389	-0.02	0.328
397	pm14-pm21	-0.127	0.006	-0.132	0.009	-0.123	0.01

398	pm14-pm22	-0.496	<0.001	-0.477	<0.001	-0.46	<0.001
399	pm14-pm23	0.014	0.345	0.033	0.223	0.002	0.699
400	pm14-pm24	-0.007	0.561	0.015	0.38	0.001	0.739
401	pm14-pm25	-0.015	0.311	0.021	0.211	0.01	0.334
402	pm14-pm26	0.029	0.226	-0.01	0.443	0.028	0.294
403	pm14-pm27	-0.013	0.324	-0.013	0.303	-0.006	0.46
404	pm14-pm28	-0.43	<0.001	-0.449	<0.001	-0.427	<0.001
405	pm14-pm29	-0.042	0.162	-0.063	0.037	-0.05	0.026
406	pm14-pm30	-0.051	0.026	-0.018	0.4	-0.052	0.035
407	pm14-pm31	0.001	0.998	0.037	0.233	0.027	0.297
408	pm14-pm32	0.01	0.491	-0.004	0.786	-0.01	0.54
409	pm14-pm33	-0.014	0.365	-0.014	0.348	-0.021	0.266
410	pm14-pm34	0.007	0.465	0.003	0.663	0.016	0.364
411	pm14-pm35	-0.016	0.358	-0.031	0.229	-0.006	0.569
412	pm14-pm36	0.029	0.28	-0.016	0.365	0.007	0.429
413	pm14-pm37	-0.015	0.318	0.019	0.362	-0.027	0.224
414	pm15-pm16	-0.283	<0.001	-0.258	<0.001	-0.266	<0.001
415	pm15-pm17	0.013	0.369	0.006	0.557	0.001	0.845
416	pm15-pm18	-0.003	0.763	-0.014	0.318	0.003	0.653
417	pm15-pm19	0.014	0.392	-0.025	0.271	0.005	0.544
418	pm15-pm20	-0.022	0.23	0.02	0.283	-0.007	0.444
419	pm15-pm21	-0.113	0.005	-0.079	0.012	-0.098	0.018
420	pm15-pm22	0.028	0.234	0.038	0.283	0.006	0.569
421	pm15-pm23	-0.008	0.591	0.016	0.311	0.004	0.844
422	pm15-pm24	-0.029	0.273	-0.002	0.741	0.005	0.599
423	pm15-pm25	-0.008	0.6	-0.006	0.584	0.012	0.348
424	pm15-pm26	0.015	0.345	-0.016	0.335	0.011	0.37
425	pm15-pm27	-0.009	0.488	-0.001	0.774	-0.023	0.296
426	pm15-pm28	-0.465	<0.001	-0.464	<0.001	-0.434	<0.001
427	pm15-pm29	-0.033	0.242	-0.009	0.464	0.012	0.364

428	pm15-pm30	-0.039	0.297	-0.049	0.176	-0.034	0.226
429	pm15-pm31	0.015	0.336	-0.015	0.311	0.008	0.513
430	pm15-pm32	-0.002	0.848	0.006	0.424	-0.026	0.213
431	pm15-pm33	-0.046	0.185	-0.01	0.37	-0.012	0.325
432	pm15-pm34	0.028	0.262	0.007	0.512	-0.013	0.316
433	pm15-pm35	-0.025	0.296	-0.022	0.225	-0.027	0.219
434	pm15-pm36	-0.012	0.353	-0.011	0.309	0.011	0.37
435	pm15-pm37	-0.014	0.379	-0.025	0.231	-0.015	0.318
436	pm16-pm17	-0.006	0.571	-0.015	0.345	0	0.666
437	pm16-pm18	0.013	0.343	-0.011	0.337	0.007	0.419
438	pm16-pm19	0.022	0.211	0.008	0.493	0.044	0.188
439	pm16-pm20	0.013	0.397	0.021	0.219	-0.001	0.628
440	pm16-pm21	-0.116	0.005	-0.094	0.016	-0.073	0.019
441	pm16-pm22	0.024	0.263	0.017	0.397	0.014	0.335
442	pm16-pm23	0.036	0.247	0.037	0.222	0.02	0.237
443	pm16-pm24	0.018	0.319	0.001	0.916	0.022	0.295
444	pm16-pm25	0.041	0.192	0.04	0.189	0.033	0.264
445	pm16-pm26	0.01	0.558	0.046	0.164	0.019	0.378
446	pm16-pm27	0.023	0.225	0.026	0.257	0.033	0.248
447	pm16-pm28	-0.479	<0.001	-0.447	<0.001	-0.442	<0.001
448	pm16-pm29	-0.47	<0.001	-0.444	<0.001	-0.452	<0.001
449	pm16-pm30	-0.021	0.219	-0.033	0.282	-0.019	0.395
450	pm16-pm31	-0.023	0.215	-0.012	0.3	-0.019	0.353
451	pm16-pm32	-0.011	0.317	0.003	0.763	0.012	0.374
452	pm16-pm33	-0.001	0.704	-0.007	0.433	-0.004	0.852
453	pm16-pm34	0.034	0.241	0.008	0.423	0.004	0.659
454	pm16-pm35	-0.028	0.202	-0.003	0.942	-0.034	0.212
455	pm16-pm36	-0.015	0.345	-0.017	0.359	-0.004	0.761
456	pm16-pm37	0.004	0.912	-0.004	0.727	-0.005	0.579
457	pm17-pm18	-0.021	0.218	-0.008	0.465	-0.007	0.48

458	pm17-pm19	0	0.785	0.019	0.349	-0.013	0.387
459	pm17-pm20	-0.013	0.36	-0.018	0.354	-0.014	0.346
460	pm17-pm21	-0.121	0.009	-0.106	0.009	-0.116	0.008
461	pm17-pm22	-0.007	0.478	-0.002	0.712	-0.032	0.3
462	pm17-pm23	0.011	0.381	0.006	0.465	-0.008	0.437
463	pm17-pm24	0.016	0.399	-0.002	0.851	0.023	0.205
464	pm17-pm25	-0.008	0.42	0.026	0.246	0	0.833
465	pm17-pm26	-0.015	0.388	0.011	0.374	-0.009	0.438
466	pm17-pm27	-0.022	0.292	-0.033	0.247	-0.006	0.54
467	pm17-pm28	-0.273	<0.001	-0.271	<0.001	-0.244	<0.001
468	pm17-pm29	-0.029	0.206	-0.032	0.297	-0.061	0.033
469	pm17-pm30	-0.068	0.032	-0.054	0.045	-0.051	0.037
470	pm17-pm31	-0.033	0.22	0.01	0.307	-0.001	0.892
471	pm17-pm32	-0.003	0.898	-0.021	0.287	0.012	0.334
472	pm17-pm33	-0.001	0.902	-0.004	0.946	-0.019	0.347
473	pm17-pm34	-0.05	0.171	-0.013	0.369	-0.041	0.155
474	pm17-pm35	-0.005	0.573	-0.019	0.368	0.013	0.304
475	pm17-pm36	-0.011	0.341	0.016	0.314	0.028	0.204
476	pm17-pm37	-0.02	0.316	-0.028	0.254	-0.008	0.527
477	pm18-pm19	0.013	0.304	-0.009	0.517	-0.023	0.202
478	pm18-pm20	0.021	0.229	-0.022	0.288	-0.011	0.329
479	pm18-pm21	-0.134	0.008	-0.103	0.007	-0.12	0.009
480	pm18-pm22	-0.01	0.321	0.034	0.288	-0.014	0.331
481	pm18-pm23	-0.26	<0.001	-0.221	<0.001	-0.222	<0.001
482	pm18-pm24	0.004	0.611	-0.011	0.33	-0.025	0.287
483	pm18-pm25	0.019	0.347	0.008	0.571	-0.022	0.224
484	pm18-pm26	-0.021	0.218	-0.01	0.565	0.024	0.29
485	pm18-pm27	-0.022	0.296	-0.015	0.374	-0.026	0.247
486	pm18-pm28	-0.466	<0.001	-0.429	<0.001	-0.465	<0.001
487	pm18-pm29	-0.018	0.343	0.008	0.556	-0.033	0.25

488	pm18-pm30	0.009	0.555	-0.038	0.283	-0.027	0.274
489	pm18-pm31	0.04	0.159	0.006	0.583	0.023	0.233
490	pm18-pm32	-0.009	0.495	0.019	0.345	-0.008	0.592
491	pm18-pm33	0.002	0.841	0.023	0.28	0.016	0.311
492	pm18-pm34	0.001	0.929	0	0.66	0.04	0.259
493	pm18-pm35	0.004	0.902	-0.021	0.277	-0.029	0.205
494	pm18-pm36	-0.02	0.32	-0.018	0.368	-0.012	0.363
495	pm18-pm37	0.005	0.514	0.015	0.364	-0.007	0.511
496	pm19-pm20	-0.006	0.464	0.017	0.363	-0.011	0.393
497	pm19-pm21	-0.125	0.008	-0.087	0.014	-0.123	0.01
498	pm19-pm22	0.003	0.721	-0.003	0.797	-0.003	0.995
499	pm19-pm23	-0.001	0.9	0.036	0.271	0.012	0.342
500	pm19-pm24	-0.014	0.314	0.005	0.468	-0.013	0.363
501	pm19-pm25	-0.023	0.286	0	0.945	0.011	0.315
502	pm19-pm26	0.01	0.525	-0.021	0.272	0.01	0.304
503	pm19-pm27	0.01	0.326	0.004	0.877	-0.001	0.72
504	pm19-pm28	-0.44	<0.001	-0.442	<0.001	-0.481	<0.001
505	pm19-pm29	-0.014	0.393	0.017	0.335	0.01	0.415
506	pm19-pm30	-0.016	0.35	-0.029	0.288	0.004	0.965
507	pm19-pm31	-0.005	0.903	0.024	0.259	0.032	0.205
508	pm19-pm32	0.002	0.677	-0.016	0.387	-0.031	0.216
509	pm19-pm33	-0.007	0.458	-0.004	0.877	0.013	0.378
510	pm19-pm34	-0.022	0.279	-0.02	0.231	-0.015	0.345
511	pm19-pm35	0.015	0.382	0.028	0.245	0.013	0.345
512	pm19-pm36	0.012	0.385	0.023	0.276	0.021	0.226
513	pm19-pm37	-0.011	0.366	0.035	0.24	0.034	0.214
514	pm20-pm21	-0.117	0.005	-0.091	0.012	-0.129	0.007
515	pm20-pm22	0.046	0.198	-0.252	<0.001	-0.26	<0.001
516	pm20-pm23	0.03	0.235	0.035	0.286	0.017	0.31
517	pm20-pm24	0.031	0.267	0.04	0.179	0.036	0.256

518	pm20-pm25	-0.019	0.374	-0.003	0.812	0.01	0.333
519	pm20-pm26	0.005	0.818	-0.023	0.298	0.009	0.546
520	pm20-pm27	0.008	0.58	-0.025	0.249	-0.024	0.217
521	pm20-pm28	-0.259	<0.001	-0.238	<0.001	-0.246	<0.001
522	pm20-pm29	-0.282	<0.001	-0.259	<0.001	-0.248	<0.001
523	pm20-pm30	-0.021	0.265	-0.039	0.266	-0.039	0.22
524	pm20-pm31	0.037	0.252	0.003	0.759	0.03	0.218
525	pm20-pm32	-0.021	0.289	-0.029	0.218	-0.025	0.288
526	pm20-pm33	0.013	0.321	-0.008	0.432	-0.014	0.394
527	pm20-pm34	-0.028	0.292	-0.001	0.912	-0.021	0.248
528	pm20-pm35	-0.018	0.394	-0.027	0.224	0.008	0.414
529	pm20-pm36	0.011	0.311	-0.001	0.901	0.022	0.278
530	pm20-pm37	-0.003	0.665	-0.016	0.362	0.008	0.515
531	pm21-pm22	-0.065	0.025	-0.064	0.03	-0.038	0.295
532	pm21-pm23	-0.071	0.014	-0.07	0.031	-0.101	0.006
533	pm21-pm24	-0.091	0.012	-0.101	0.008	-0.116	0.006
534	pm21-pm25	-0.084	0.018	-0.095	0.015	-0.118	0.006
535	pm21-pm26	-0.134	0.01	-0.121	0.006	-0.131	0.006
536	pm21-pm27	-0.133	0.009	-0.111	0.005	-0.121	0.006
537	pm21-pm28	-0.014	0.375	-0.043	0.189	-0.036	0.285
538	pm21-pm29	-0.126	0.01	-0.093	0.017	-0.095	0.016
539	pm21-pm30	-0.157	0.001	-0.126	0.007	-0.146	0.007
540	pm21-pm31	-0.072	0.015	-0.088	0.019	-0.068	0.044
541	pm21-pm32	-0.12	0.007	-0.095	0.016	-0.096	0.013
542	pm21-pm33	-0.1	0.015	-0.059	0.042	-0.095	0.019
543	pm21-pm34	-0.089	0.011	-0.06	0.038	-0.105	0.006
544	pm21-pm35	-0.084	0.016	-0.077	0.016	-0.075	0.013
545	pm21-pm36	-0.116	0.01	-0.072	0.013	-0.107	0.008
546	pm21-pm37	-0.083	0.018	-0.075	0.011	-0.081	0.012
547	pm22-pm23	0.164	0.003	0.153	0.004	0.163	0.004

548	pm22-pm24	-0.276	<0.001	-0.083	0.016	0.045	0.197
549	pm22-pm25	0.006	0.507	0.024	0.283	0.023	0.265
550	pm22-pm26	0.001	0.813	0.025	0.229	0.029	0.236
551	pm22-pm27	0.02	0.391	0.01	0.459	0.015	0.342
552	pm22-pm28	-0.22	<0.001	-0.211	<0.001	-0.227	<0.001
553	pm22-pm29	-0.385	<0.001	-0.348	<0.001	-0.367	<0.001
554	pm22-pm30	-0.067	0.03	-0.066	0.035	-0.065	0.026
555	pm22-pm31	0.02	0.23	0.024	0.213	0.039	0.277
556	pm22-pm32	0.025	0.229	0.038	0.268	0.004	0.938
557	pm22-pm33	0.02	0.227	-0.003	0.603	0.001	0.902
558	pm22-pm34	0.007	0.528	0.027	0.25	0.014	0.376
559	pm22-pm35	0.028	0.289	0.022	0.26	0.009	0.422
560	pm22-pm36	0.011	0.38	0.01	0.339	0.012	0.384
561	pm22-pm37	0.044	0.19	0.039	0.215	0.02	0.345
562	pm23-pm24	-0.215	<0.001	-0.255	<0.001	-0.25	<0.001
563	pm23-pm25	0.008	0.59	-0.007	0.568	-0.017	0.324
564	pm23-pm26	-0.277	<0.001	-0.243	<0.001	-0.24	<0.001
565	pm23-pm27	-0.26	<0.001	-0.252	<0.001	-0.24	<0.001
566	pm23-pm28	-0.215	<0.001	-0.219	<0.001	-0.206	<0.001
567	pm23-pm29	0.015	0.346	0.012	0.364	0.013	0.39
568	pm23-pm30	-0.032	0.228	-0.038	0.254	-0.028	0.213
569	pm23-pm31	0.023	0.228	0.013	0.321	0.005	0.806
570	pm23-pm32	0.021	0.214	0.003	0.69	0.038	0.212
571	pm23-pm33	0.004	0.651	0.009	0.474	-0.003	0.933
572	pm23-pm34	0.012	0.398	-0.023	0.282	0.004	0.813
573	pm23-pm35	0.011	0.383	0.007	0.5	-0.015	0.383
574	pm23-pm36	-0.475	<0.001	-0.444	<0.001	-0.472	<0.001
575	pm23-pm37	0.012	0.328	-0.008	0.514	-0.01	0.566
576	pm24-pm25	0.026	0.268	-0.002	0.63	0.007	0.588
577	pm24-pm26	-0.022	0.297	-0.002	0.918	0.001	0.641

578	pm24-pm27	0	0.727	-0.032	0.225	-0.015	0.391
579	pm24-pm28	-0.238	<0.001	-0.24	<0.001	-0.225	<0.001
580	pm24-pm29	-0.031	0.232	-0.043	0.153	-0.013	0.398
581	pm24-pm30	-0.045	0.199	-0.023	0.211	-0.021	0.207
582	pm24-pm31	0.024	0.274	-0.013	0.312	-0.003	0.759
583	pm24-pm32	-0.021	0.291	0.012	0.385	-0.02	0.325
584	pm24-pm33	-0.018	0.36	-0.032	0.209	-0.015	0.348
585	pm24-pm34	0.017	0.341	0.018	0.353	-0.003	0.622
586	pm24-pm35	0.032	0.292	0.034	0.266	0	0.739
587	pm24-pm36	-0.012	0.383	-0.032	0.212	0	0.873
588	pm24-pm37	0.035	0.249	0.019	0.391	0.019	0.32
589	pm25-pm26	0.016	0.348	-0.009	0.402	0.026	0.247
590	pm25-pm27	0.009	0.418	-0.032	0.243	-0.016	0.393
591	pm25-pm28	-0.26	<0.001	-0.24	<0.001	-0.226	<0.001
592	pm25-pm29	-0.02	0.352	-0.031	0.275	-0.045	0.164
593	pm25-pm30	-0.025	0.272	-0.007	0.412	-0.026	0.238
594	pm25-pm31	0.021	0.279	0.021	0.252	0.001	0.605
595	pm25-pm32	-0.004	0.65	-0.017	0.389	0.029	0.241
596	pm25-pm33	-0.014	0.375	0.009	0.575	-0.011	0.346
597	pm25-pm34	0.004	0.981	-0.008	0.482	-0.012	0.307
598	pm25-pm35	-0.027	0.24	0.008	0.526	-0.015	0.372
599	pm25-pm36	0.006	0.532	0.004	0.979	-0.02	0.315
600	pm25-pm37	0.024	0.261	0.023	0.233	0.034	0.247
601	pm26-pm27	-0.014	0.359	0.003	0.719	0.009	0.55
602	pm26-pm28	-0.284	<0.001	-0.267	<0.001	-0.244	<0.001
603	pm26-pm29	-0.006	0.453	0.003	0.987	-0.032	0.229
604	pm26-pm30	-0.017	0.34	-0.014	0.396	-0.034	0.238
605	pm26-pm31	-0.008	0.538	-0.009	0.577	0.021	0.227
606	pm26-pm32	0.01	0.314	0.024	0.282	-0.019	0.306
607	pm26-pm33	-0.004	0.623	-0.005	0.56	0.025	0.247

608	pm26-pm34	0.004	0.866	-0.03	0.223	-0.017	0.347
609	pm26-pm35	-0.03	0.205	-0.009	0.418	-0.031	0.24
610	pm26-pm36	0.017	0.392	0.001	0.739	0.008	0.543
611	pm26-pm37	0.022	0.231	0.006	0.41	0.007	0.559
612	pm27-pm28	-0.284	<0.001	-0.271	<0.001	-0.272	<0.001
613	pm27-pm29	-0.009	0.567	0.009	0.442	-0.011	0.304
614	pm27-pm30	-0.026	0.226	-0.037	0.263	-0.013	0.372
615	pm27-pm31	-0.021	0.291	-0.02	0.371	-0.027	0.246
616	pm27-pm32	-0.016	0.326	0.006	0.534	0.004	0.62
617	pm27-pm33	-0.023	0.208	-0.028	0.212	-0.028	0.247
618	pm27-pm34	0.033	0.212	0.013	0.34	0.016	0.376
619	pm27-pm35	-0.01	0.464	0.021	0.289	0.027	0.289
620	pm27-pm36	0.029	0.243	-0.005	0.785	-0.007	0.579
621	pm27-pm37	0.031	0.272	-0.007	0.449	-0.009	0.466
622	pm28-pm29	-0.204	<0.001	-0.189	0.002	-0.188	0.003
623	pm28-pm30	0.024	0.222	0.006	0.503	0.031	0.261
624	pm28-pm31	-0.233	<0.001	-0.223	<0.001	-0.235	<0.001
625	pm28-pm32	-0.263	<0.001	-0.279	<0.001	-0.267	<0.001
626	pm28-pm33	-0.248	<0.001	-0.247	<0.001	-0.29	<0.001
627	pm28-pm34	-0.213	<0.001	-0.222	<0.001	-0.229	<0.001
628	pm28-pm35	-0.239	<0.001	-0.249	<0.001	-0.214	<0.001
629	pm28-pm36	0.022	0.282	0.018	0.302	0.003	0.745
630	pm28-pm37	-0.462	<0.001	-0.492	<0.001	-0.495	<0.001
631	pm29-pm30	-0.258	<0.001	-0.243	<0.001	-0.277	<0.001
632	pm29-pm31	0.016	0.37	0.015	0.347	0.001	0.882
633	pm29-pm32	0.001	0.932	0.038	0.23	0.009	0.444
634	pm29-pm33	-0.039	0.276	-0.054	0.045	-0.046	0.183
635	pm29-pm34	-0.026	0.271	-0.011	0.389	0.012	0.361
636	pm29-pm35	-0.043	0.168	-0.017	0.328	0.002	0.728
637	pm29-pm36	0	0.696	-0.012	0.39	0.019	0.362

638	pm29-pm37	0.027	0.222	0.03	0.229	0	0.744
639	pm30-pm31	-0.029	0.252	-0.025	0.216	0.007	0.594
640	pm30-pm32	0.003	0.661	-0.041	0.198	-0.015	0.341
641	pm30-pm33	0.004	0.746	0	0.65	-0.018	0.376
642	pm30-pm34	-0.065	0.045	-0.039	0.221	-0.079	0.013
643	pm30-pm35	-0.014	0.354	-0.021	0.255	0.024	0.258
644	pm30-pm36	-0.008	0.465	0.021	0.287	0.017	0.386
645	pm30-pm37	0.01	0.586	0.026	0.247	0.016	0.358
646	pm31-pm32	-0.022	0.273	0.014	0.322	-0.014	0.315
647	pm31-pm33	-0.004	0.996	0.023	0.212	0.033	0.265
648	pm31-pm34	0.014	0.324	-0.014	0.321	0.027	0.243
649	pm31-pm35	0.019	0.371	0.01	0.592	-0.001	0.847
650	pm31-pm36	-0.003	0.763	-0.013	0.367	-0.011	0.369
651	pm31-pm37	-0.016	0.35	0.017	0.319	-0.004	0.728
652	pm32-pm33	0.027	0.205	-0.017	0.394	-0.01	0.36
653	pm32-pm34	-0.011	0.305	-0.011	0.372	-0.015	0.367
654	pm32-pm35	0.016	0.399	0.035	0.291	0.028	0.297
655	pm32-pm36	0	0.772	-0.01	0.426	0	0.868
656	pm32-pm37	0.011	0.356	0.017	0.341	0.016	0.365
657	pm33-pm34	-0.008	0.593	0.013	0.374	-0.025	0.21
658	pm33-pm35	-0.003	0.82	0.009	0.413	0.003	0.79
659	pm33-pm36	0.023	0.214	-0.019	0.367	0.005	0.448
660	pm33-pm37	0.012	0.333	0.024	0.289	0.01	0.555
661	pm34-pm35	0.005	0.852	0.029	0.248	0.006	0.515
662	pm34-pm36	0.032	0.267	0.024	0.201	0.012	0.306
663	pm34-pm37	0.021	0.232	-0.017	0.399	0.012	0.334
664	pm35-pm36	-0.009	0.597	-0.002	0.824	0.011	0.329
665	pm35-pm37	-0.007	0.518	-0.024	0.279	-0.021	0.251
666	pm36-pm37	0.007	0.44	0.004	0.852	0.003	0.672

Supplementray Data Set S4. Results of the distribution perturbation analyses for hypertrophy in the cardiac signaling network.

1a) Result of one-distribution perturbation analysis for hypertrophy when each response function was separately applied

		Lin		Hill		Sat		Acc	
		Effect	p-value	Effect	p-value	Effect	p-value	Effect	p-value
Threshold: top 5%	pm1	1.019	0.818	1.025	0.806	1.020	0.132	0.998	0.328
	pm2	0.999	0.917	1.003	0.774	0.998	0.451	0.999	0.607
	pm3	1.002	0.679	1.034	0.908	0.994	0.421	1.019	0.670
	pm4	1.005	0.859	1.092	0.536	1.000	0.986	1.005	0.115
	pm5	1.008	0.486	1.000	0.870	0.998	0.693	1.003	0.585
	pm6	0.998	0.411	0.994	0.435	0.997	0.545	0.998	0.642
	pm7	3.115	<0.001	2.285	<0.001	3.306	<0.001	2.322	<0.001
	pm8	1.029	0.401	1.004	0.169	1.002	0.594	1.047	0.322
	pm9	1.040	0.377	1.066	0.493	1.027	0.349	1.024	0.754
	pm10	1.557	<0.001	1.542	<0.001	1.367	<0.001	1.692	<0.001
	pm11	0.998	0.616	0.996	0.422	0.996	0.593	0.996	0.548
	pm12	1.005	0.609	1.038	0.763	0.998	0.562	1.017	0.804
	pm13	0.864	0.012	0.841	0.009	0.897	0.184	0.765	<0.001
	pm14	0.913	0.209	0.894	0.039	0.927	0.961	0.879	0.027
	pm15	1.151	0.021	1.267	<0.001	1.162	0.024	1.169	0.020
	pm16	1.148	0.022	1.193	0.008	1.218	<0.001	1.178	0.010
	pm17	1.148	0.022	1.170	0.009	1.196	0.007	1.154	0.020
	pm18	1.014	0.303	1.023	0.105	0.998	0.439	1.005	0.541
	pm19	1.002	0.625	1.077	0.645	1.042	0.492	1.051	0.681
	pm20	1.019	0.209	1.032	0.480	0.996	0.279	1.017	0.642

	pm21	1.626	<0.001	1.143	0.033	1.873	<0.001	1.207	<0.001
	pm22	0.984	0.277	1.045	0.356	0.928	0.751	0.992	0.262
	pm23	0.986	0.690	1.038	0.829	0.929	0.949	0.992	0.524
	pm24	0.998	0.278	1.029	0.591	0.999	0.638	0.995	0.793
	pm25	0.998	0.582	0.996	0.916	0.997	0.329	0.998	0.567
	pm26	0.997	0.572	0.995	0.407	0.997	0.502	0.998	0.403
	pm27	1.018	0.769	1.080	0.818	1.019	0.290	1.005	0.455
	pm28	1.010	0.149	1.028	0.562	1.002	0.357	1.026	0.216
	pm29	1.007	0.956	1.024	0.876	0.984	0.468	1.010	0.344
	pm30	1.011	0.362	1.023	0.357	0.988	0.718	1.026	0.774
	pm31	1.023	0.537	1.096	0.394	1.026	0.417	1.012	0.566
	pm32	1.001	0.985	1.020	0.375	1.003	0.292	1.072	0.920
	pm33	1.022	0.214	1.136	0.025	1.004	0.140	1.201	0.016
	pm34	1.016	0.806	1.036	0.871	0.921	0.660	1.072	0.380
	pm35	1.018	0.504	1.042	0.289	0.933	0.819	1.125	0.319
	pm36	1.000	0.654	0.998	0.622	0.996	0.608	0.998	0.532
	pm37	0.997	0.976	0.992	0.385	0.997	0.976	0.998	0.742
Threshold: top 10%		Lin		Hill		Sat		Acc	
		Effect	p-value	Effect	p-value	Effect	p-value	Effect	p-value
	pm1	1.016	0.508	1.024	0.780	1.017	0.301	0.998	0.834
	pm2	0.999	0.314	1.003	0.975	0.998	0.446	0.999	0.215
	pm3	1.002	0.140	1.030	0.454	0.994	0.836	1.017	0.203
	pm4	1.004	0.901	1.074	0.160	1.000	0.951	1.004	0.204
	pm5	1.007	0.629	1.000	0.628	0.998	0.547	1.003	0.792
	pm6	0.998	0.574	0.994	0.338	0.997	0.212	0.998	0.194

	pm7	2.920	<0.001	2.081	<0.001	2.798	<0.001	2.387	<0.001
	pm8	1.023	0.567	1.004	0.158	1.002	0.604	1.044	0.364
	pm9	1.041	0.166	1.067	0.974	1.023	0.880	1.023	0.479
	pm10	1.448	<0.001	1.565	<0.001	1.286	<0.001	1.532	<0.001
	pm11	0.998	0.967	0.996	0.968	0.997	0.842	0.996	0.155
	pm12	1.004	0.948	1.031	0.378	0.999	0.195	1.014	0.437
	pm13	0.860	0.013	0.856	0.012	0.896	0.224	0.822	<0.001
	pm14	0.915	0.720	0.885	0.468	0.925	0.241	0.871	0.012
	pm15	1.126	0.033	1.260	<0.001	1.125	0.033	1.144	0.021
	pm16	1.151	0.020	1.171	0.011	1.207	<0.001	1.101	0.043
	pm17	1.156	0.021	1.185	<0.001	1.221	<0.001	1.142	0.022
	pm18	1.012	0.440	1.021	0.569	0.998	0.833	1.005	0.298
	pm19	1.002	0.249	1.069	0.983	1.040	0.574	1.043	0.430
	pm20	1.020	0.491	1.032	0.901	0.996	0.612	1.014	0.639
	pm21	1.574	<0.001	1.129	0.036	1.831	<0.001	1.183	0.012
	pm22	0.985	0.558	1.048	0.818	0.931	0.284	0.992	0.408
	pm23	0.987	0.110	1.029	0.756	0.934	0.145	0.991	0.634
	pm24	0.997	0.979	1.029	0.126	0.999	0.193	0.995	0.773
	pm25	0.998	0.446	0.996	0.431	0.997	0.978	0.998	0.522
	pm26	0.998	0.754	0.996	0.383	0.997	0.963	0.998	0.404
	pm27	1.014	0.656	1.083	0.972	1.022	0.276	1.005	0.683
	pm28	1.008	0.949	1.024	0.809	1.002	0.658	1.020	0.958
	pm29	1.006	0.323	1.018	0.400	0.987	0.485	1.010	0.369
	pm30	1.012	0.168	1.019	0.701	0.987	0.192	1.020	0.671
	pm31	1.019	0.880	1.084	0.359	1.023	0.567	1.014	0.632
	pm32	1.001	0.508	1.018	0.758	1.004	0.636	1.058	0.265

	pm33	1.019	0.733	1.121	0.038	1.004	0.885	1.184	0.011
	pm34	1.013	0.156	1.031	0.311	0.929	0.284	1.062	0.103
	pm35	1.015	0.834	1.036	0.565	0.933	0.467	1.112	0.944
	pm36	1.000	0.337	0.997	0.170	0.997	0.608	0.998	0.799
	pm37	0.998	0.348	0.994	0.872	0.997	0.179	0.998	0.520

		Lin		Hill		Sat		Acc	
		Effect	p-value	Effect	p-value	Effect	p-value	Effect	p-value
Threshold: top 20%	pm1	1.015	0.113	1.018	0.686	1.015	0.168	0.998	0.315
	pm2	0.999	0.515	1.003	0.332	0.998	0.787	0.999	0.916
	pm3	1.002	0.188	1.026	0.631	0.995	0.648	1.015	0.433
	pm4	1.003	0.144	1.066	0.790	1.000	0.571	1.004	0.152
	pm5	1.006	0.438	1.000	0.341	0.999	0.884	1.002	0.698
	pm6	0.998	0.809	0.995	0.874	0.998	0.247	0.998	0.147
	pm7	2.654	<0.001	1.920	<0.001	2.509	<0.001	2.203	<0.001
	pm8	1.021	0.957	1.003	0.486	1.002	0.128	1.038	0.432
	pm9	1.036	0.657	1.056	0.944	1.020	0.557	1.020	0.459
	pm10	1.411	<0.001	1.505	<0.001	1.252	<0.001	1.500	<0.001
	pm11	0.998	0.291	0.996	0.977	0.997	0.421	0.996	0.896
	pm12	1.003	0.785	1.026	0.975	0.999	0.701	1.013	0.523
	pm13	0.893	0.038	0.869	0.024	0.905	0.708	0.837	<0.001
	pm14	0.927	0.880	0.906	0.901	0.940	0.830	0.898	0.228
	pm15	1.134	0.040	1.223	<0.001	1.135	0.035	1.139	0.031
	pm16	1.148	0.022	1.137	0.028	1.166	0.015	1.151	0.020
	pm17	1.127	0.041	1.156	0.030	1.173	0.011	1.130	0.037
	pm18	1.010	0.951	1.017	0.602	0.998	0.447	1.004	0.281

	pm19	1.002	1.000	1.061	0.129	1.036	0.230	1.037	0.170
	pm20	1.016	0.182	1.026	0.169	0.996	0.839	1.012	0.530
	pm21	1.475	<0.001	1.122	0.042	1.748	<0.001	1.176	0.013
	pm22	0.987	0.687	1.039	0.252	0.933	0.206	0.993	0.432
	pm23	0.990	0.377	1.028	0.979	0.935	0.122	0.993	0.750
	pm24	0.998	0.511	1.024	0.350	0.999	0.992	0.996	0.276
	pm25	0.998	0.755	0.996	0.777	0.998	0.743	0.998	0.612
	pm26	0.998	0.534	0.996	0.137	0.998	0.893	0.998	0.927
	pm27	1.013	0.341	1.068	0.224	1.017	0.361	1.005	0.966
	pm28	1.007	0.405	1.021	0.156	1.002	0.783	1.019	0.442
	pm29	1.005	0.799	1.016	0.880	0.989	0.303	1.009	0.402
	pm30	1.009	0.191	1.017	0.220	0.989	0.809	1.017	0.289
	pm31	1.018	0.250	1.077	0.395	1.019	0.889	1.011	0.370
	pm32	1.001	0.423	1.015	0.646	1.003	0.494	1.049	0.327
	pm33	1.016	0.961	1.106	0.079	1.003	0.948	1.158	0.036
	pm34	1.011	0.390	1.029	0.688	0.942	0.969	1.057	0.515
	pm35	1.014	0.782	1.033	0.411	0.944	0.902	1.090	0.217
	pm36	1.000	0.219	0.998	0.792	0.997	0.602	0.998	0.403
	pm37	0.998	0.857	0.994	0.915	0.998	0.625	0.998	0.590

1b) Result of one-distribution perturbation analysis for hypertrophy when four different response function types were applied in a combined manner

	Top 5%		Top 10%		Top 20%	
	Effect	p-value	Effect	p-value	Effect	p-value
pm1	1.014	0.956	1.012	0.965	1.009	0.889
pm2	0.999	0.662	0.999	0.785	1.000	0.983
pm3	1.002	0.405	1.001	0.860	1.001	0.290
pm4	1.003	0.623	1.002	0.693	1.002	0.307
pm5	1.007	0.274	1.005	0.950	1.005	0.831
pm6	0.998	0.860	0.998	0.221	0.999	0.442
pm7	2.412	<0.001	2.350	<0.001	1.955	<0.001
pm8	1.019	0.462	1.017	0.575	1.013	0.398
pm9	1.037	0.122	1.030	0.709	1.025	0.933
pm10	1.397	<0.001	1.334	<0.001	1.247	<0.001
pm11	0.998	0.673	0.998	0.269	0.999	0.828
pm12	1.003	0.462	1.003	0.178	1.002	0.842
pm13	0.879	0.018	0.913	0.037	0.919	0.031
pm14	0.925	0.176	0.934	0.563	0.946	0.912
pm15	1.291	<0.001	1.175	0.010	1.165	0.012
pm16	1.259	<0.001	1.220	<0.001	1.146	0.022
pm17	1.191	0.006	1.153	0.018	1.142	0.025
pm18	1.008	0.799	1.008	0.464	1.006	0.162
pm19	1.002	0.816	1.001	0.713	1.001	0.458
pm20	1.017	0.925	1.014	0.245	1.011	0.719
pm21	1.438	<0.001	1.396	<0.001	1.292	<0.001

pm22	0.986	0.147	0.989	0.482	0.991	0.983
pm23	0.990	0.574	0.991	0.766	0.993	0.413
pm24	0.998	0.884	0.998	0.748	0.999	0.644
pm25	0.998	0.868	0.999	0.808	0.999	0.560
pm26	0.998	0.719	0.998	0.959	0.999	0.432
pm27	1.013	0.434	1.010	0.192	1.009	0.565
pm28	1.008	0.619	1.006	0.770	1.004	0.778
pm29	1.005	0.745	1.004	0.440	1.003	0.975
pm30	1.009	0.238	1.008	0.951	1.006	0.275
pm31	1.016	0.373	1.015	0.190	1.012	0.156
pm32	1.001	0.553	1.001	0.766	1.001	0.381
pm33	1.016	0.940	1.011	0.959	1.011	0.737
pm34	1.010	0.947	1.008	0.988	1.007	0.694
pm35	1.012	0.944	1.010	0.143	1.008	0.551
pm36	1.000	0.914	1.000	0.542	1.000	0.784
pm37	0.998	0.601	0.999	0.270	0.999	0.521

2) Result of reverse one-distribution perturbation analysis for hypertrophy in cardiac signaling network

2a) Result of reverse one-distribution perturbation analysis for hypertrophy when each response function was separately applied

		Lin		Hill		Sat		Acc	
		Effect	p-value	Effect	p-value	Effect	p-value	Effect	p-value
Threshold: top 5%	pm1	0.992	0.127	0.968	0.451	0.985	0.556	0.989	0.517
	pm2	0.998	0.134	0.990	0.868	0.997	0.434	0.995	0.733
	pm3	0.999	0.756	0.970	0.869	0.990	0.108	0.961	0.742
	pm4	0.996	0.570	0.932	0.189	0.992	0.366	0.972	0.915
	pm5	0.996	0.777	0.999	0.571	0.999	0.260	0.978	0.992
	pm6	0.997	0.258	0.994	0.451	0.997	0.587	0.998	0.184
	pm7	0.005	<0.001	0.119	<0.001	0.001	<0.001	0.047	<0.001
	pm8	0.983	0.737	0.996	0.814	0.992	0.311	0.954	0.868
	pm9	0.972	0.811	0.922	0.530	0.977	0.274	0.972	0.539
	pm10	0.509	<0.001	0.443	<0.001	0.647	<0.001	0.322	<0.001
	pm11	0.997	0.115	0.993	0.830	0.997	0.173	0.991	0.606
	pm12	0.992	0.461	0.982	0.709	0.999	0.167	0.977	0.851
	pm13	1.012	0.917	1.025	0.134	0.979	0.417	1.029	0.705
	pm14	1.120	0.879	1.082	0.411	1.092	0.766	1.102	0.843
	pm15	1.604	<0.001	1.740	<0.001	1.620	<0.001	1.498	<0.001
	pm16	0.498	<0.001	0.417	<0.001	0.481	<0.001	0.585	<0.001
	pm17	0.464	<0.001	0.385	<0.001	0.419	<0.001	0.699	<0.001
	pm18	0.993	0.320	0.983	0.628	0.998	0.881	0.978	0.533
	pm19	1.005	0.948	0.924	0.464	0.957	0.780	0.935	0.780
	pm20	0.989	0.693	0.977	0.712	0.997	0.141	0.989	0.411

	pm21	0.489	<0.001	1.130	0.582	0.276	<0.001	0.777	<0.001
	pm22	0.993	0.126	0.980	0.820	0.987	0.251	0.983	0.632
	pm23	1.011	0.471	0.942	0.612	0.998	0.408	0.995	0.944
	pm24	0.999	0.721	0.976	0.446	0.999	0.447	0.997	0.231
	pm25	0.998	0.432	0.995	0.443	0.997	0.247	0.998	0.707
	pm26	0.998	0.649	0.996	0.274	0.997	0.341	0.998	0.807
	pm27	0.979	0.110	0.935	0.296	0.963	0.933	0.987	0.402
	pm28	1.002	0.141	0.987	0.541	0.991	0.582	0.970	0.838
	pm29	0.945	0.483	0.851	0.021	0.965	0.959	0.884	0.033
	pm30	1.025	0.742	1.074	0.571	1.007	0.114	1.041	0.183
	pm31	0.985	0.751	0.908	0.042	0.968	0.496	0.985	0.568
	pm32	0.996	0.871	0.996	0.508	0.987	0.540	0.928	0.302
	pm33	0.984	0.618	0.903	0.046	0.979	0.449	0.785	<0.001
	pm34	1.116	0.293	0.980	0.231	1.057	0.900	1.124	0.304
	pm35	0.873	0.030	0.917	0.043	0.897	0.045	0.638	<0.001
	pm36	0.999	0.180	0.996	0.264	0.998	0.919	0.998	0.177
	pm37	0.998	0.761	0.995	0.485	0.997	0.524	0.998	0.462
Threshold: top 10%		Lin		Hill		Sat		Acc	
		Effect	p-value	Effect	p-value	Effect	p-value	Effect	p-value
	pm1	0.994	0.565	0.976	0.220	0.985	0.120	0.991	0.866
	pm2	0.998	0.391	0.990	0.190	0.997	0.334	0.996	0.205
	pm3	0.999	0.582	0.974	0.811	0.991	0.168	0.971	0.279
	pm4	0.996	0.610	0.935	0.567	0.994	0.822	0.977	0.542
	pm5	0.997	0.564	0.999	0.433	0.999	0.759	0.981	0.237
	pm6	0.998	0.962	0.995	0.845	0.997	0.356	0.998	0.486

	pm7	0.048	p<0.001	0.156	p<0.001	0.178	p<0.001	0.149	p<0.001
	pm8	0.983	0.641	0.997	0.572	0.993	0.684	0.958	0.407
	pm9	0.971	0.934	0.936	0.622	0.974	0.330	0.973	0.170
	pm10	0.610	p<0.001	0.466	p<0.001	0.737	p<0.001	0.477	p<0.001
	pm11	0.997	0.168	0.994	0.164	0.998	0.806	0.992	0.966
	pm12	0.992	0.502	0.983	0.159	0.999	0.353	0.980	0.440
	pm13	1.009	0.516	1.025	0.426	0.980	0.952	1.030	0.322
	pm14	1.097	0.576	1.083	0.468	1.102	0.674	1.099	0.183
	pm15	1.558	p<0.001	1.782	p<0.001	1.534	p<0.001	1.514	p<0.001
	pm16	0.526	p<0.001	0.385	p<0.001	0.543	p<0.001	0.551	p<0.001
	pm17	0.613	p<0.001	0.395	p<0.001	0.500	p<0.001	0.714	p<0.001
	pm18	0.992	0.441	0.987	0.121	0.998	0.661	0.981	0.298
	pm19	1.005	0.225	0.941	0.889	0.966	0.801	0.944	0.727
	pm20	0.992	0.910	0.979	0.303	0.997	0.667	0.991	0.283
	pm21	0.572	p<0.001	1.109	0.824	0.339	p<0.001	0.795	p<0.001
	pm22	0.994	0.508	0.984	1.000	0.987	0.665	0.984	0.847
	pm23	1.012	0.408	0.952	0.521	0.998	0.172	0.996	0.823
	pm24	0.999	0.290	0.976	0.268	0.999	0.850	0.997	0.482
	pm25	0.998	0.119	0.996	0.373	0.997	0.206	0.998	0.204
	pm26	0.998	0.933	0.996	0.865	0.997	0.918	0.998	0.614
	pm27	0.977	0.432	0.924	0.800	0.963	0.282	0.986	0.902
	pm28	1.002	0.600	0.990	0.534	0.992	0.841	0.970	0.575
	pm29	0.958	0.445	0.856	0.020	0.966	0.256	0.901	0.588
	pm30	1.024	0.212	1.075	0.885	1.006	0.696	1.040	0.294
	pm31	0.986	0.721	0.918	0.201	0.973	0.226	0.985	0.683
	pm32	0.997	0.784	0.996	0.933	0.989	0.105	0.937	0.385

	pm33	0.986	0.347	0.906	0.633	0.981	0.769	0.806	0.011
	pm34	1.101	0.853	0.982	0.544	1.049	0.313	1.133	0.918
	pm35	0.872	0.016	0.918	0.041	0.898	0.031	0.726	p<0.001
	pm36	0.999	0.865	0.997	0.529	0.998	0.127	0.997	0.263
	pm37	0.998	0.639	0.996	0.463	0.997	0.559	0.998	0.508
		Lin		Hill		Sat		Acc	
		Effect	p-value	Effect	p-value	Effect	p-value	Effect	p-value
Threshold: top 20%	pm1	0.995	0.583	0.980	0.742	0.986	0.625	0.992	0.801
	pm2	0.998	0.625	0.991	0.871	0.998	0.983	0.996	0.108
	pm3	0.999	0.989	0.979	0.808	0.992	0.823	0.976	0.694
	pm4	0.997	0.680	0.947	0.359	0.994	0.410	0.981	0.932
	pm5	0.997	0.687	0.999	0.413	0.999	0.200	0.987	0.730
	pm6	0.998	0.966	0.997	0.290	0.998	0.320	0.999	0.206
	pm7	0.100	<0.001	0.299	<0.001	0.191	<0.001	0.195	<0.001
	pm8	0.987	0.795	0.998	0.585	0.994	0.169	0.968	0.752
	pm9	0.976	0.174	0.948	0.261	0.978	0.884	0.980	0.511
	pm10	0.669	<0.001	0.604	<0.001	0.781	<0.001	0.551	<0.001
	pm11	0.998	0.439	0.995	0.815	0.998	0.814	0.994	0.509
	pm12	0.994	0.484	0.985	0.776	0.999	0.335	0.982	0.836
	pm13	1.008	0.834	1.022	0.908	0.984	0.472	1.023	0.154
	pm14	1.077	0.836	1.060	0.735	1.082	0.803	1.092	0.130
	pm15	1.456	<0.001	1.607	<0.001	1.412	<0.001	1.438	<0.001
	pm16	0.588	<0.001	0.446	<0.001	0.620	<0.001	0.670	<0.001
	pm17	0.648	<0.001	0.499	<0.001	0.616	<0.001	0.754	<0.001
	pm18	0.993	0.882	0.989	0.544	0.998	0.233	0.985	0.721

	pm19	1.004	0.954	0.944	0.579	0.971	0.569	0.951	0.443
	pm20	0.993	0.376	0.982	0.102	0.997	0.475	0.993	0.482
	pm21	0.672	<0.001	1.056	0.799	0.486	<0.001	0.854	0.014
	pm22	0.995	0.142	0.985	0.171	0.990	0.253	0.986	0.411
	pm23	1.009	0.416	0.964	0.637	0.998	0.881	0.997	0.349
	pm24	1.000	0.167	0.981	0.168	0.999	0.869	0.998	0.295
	pm25	0.998	0.329	0.996	0.806	0.998	0.251	0.999	0.341
	pm26	0.998	0.573	0.997	0.530	0.998	0.493	0.999	0.140
	pm27	0.980	0.193	0.940	0.816	0.973	0.790	0.988	0.709
	pm28	1.001	0.278	0.990	0.486	0.994	0.781	0.971	0.234
	pm29	0.966	0.147	0.876	0.021	0.969	0.717	0.922	0.328
	pm30	1.018	0.898	1.055	0.325	1.005	0.185	1.035	0.394
	pm31	0.989	0.581	0.936	0.492	0.980	0.308	0.989	0.420
	pm32	0.997	0.556	0.997	0.745	0.992	0.170	0.950	0.414
	pm33	0.988	0.253	0.930	0.836	0.986	0.595	0.859	0.011
	pm34	1.080	0.589	0.983	0.748	1.042	0.896	1.099	0.530
	pm35	0.901	0.040	0.884	0.041	0.898	0.031	0.741	<0.001
	pm36	1.000	0.243	0.997	0.774	0.999	0.503	0.998	0.498
	pm37	0.998	0.436	0.997	0.340	0.998	0.246	0.998	0.133

2b) Result of reverse one-distribution perturbation analysis for hypertrophy when four different response function types were applied in a combined manner

	Top 5%		Top 10%		Top 20%	
	Effect	p-value	Effect	p-value	Effect	p-value
pm1	1.005	0.325	1.004	0.126	1.003	0.720
pm2	0.998	0.198	0.999	0.689	0.999	0.586
pm3	0.999	0.726	0.999	0.104	0.999	0.752
pm4	0.997	0.980	0.997	0.963	0.998	0.588
pm5	0.998	0.444	0.998	0.997	0.998	0.568
pm6	0.998	0.580	0.999	0.665	0.999	0.318
pm7	0.177	<0.001	0.269	<0.001	0.503	<0.001
pm8	0.986	0.415	0.989	0.524	0.991	0.720
pm9	0.979	0.343	0.983	0.933	0.987	0.272
pm10	0.685	<0.001	0.759	<0.001	0.795	<0.001
pm11	0.998	0.265	0.998	0.261	0.998	0.230
pm12	0.994	0.404	0.995	0.608	0.996	0.182
pm13	1.008	0.692	1.006	0.655	1.005	0.548
pm14	1.081	0.557	1.068	0.302	1.051	0.344
pm15	1.376	<0.001	1.346	<0.001	1.286	<0.001
pm16	0.634	<0.001	0.693	<0.001	0.747	<0.001
pm17	0.699	<0.001	0.733	<0.001	0.778	<0.001
pm18	0.994	0.427	0.996	0.711	0.996	0.544
pm19	1.003	0.768	1.003	0.165	1.002	0.738
pm20	0.994	0.573	0.995	0.118	0.996	0.365
pm21	1.051	0.735	1.040	0.585	1.030	0.298

pm22	0.996	0.411	0.997	0.769	0.997	0.213
pm23	1.008	0.571	1.006	0.779	1.006	0.695
pm24	1.000	0.980	1.000	0.347	1.000	0.415
pm25	0.998	0.965	0.999	0.302	0.999	0.375
pm26	0.998	0.147	0.999	0.571	0.999	0.196
pm27	0.984	0.781	0.986	0.613	0.989	0.417
pm28	1.001	0.264	1.001	0.561	1.001	0.507
pm29	0.969	0.200	0.972	0.558	0.977	0.567
pm30	1.018	0.762	1.014	0.529	1.013	0.766
pm31	0.989	0.114	0.991	0.164	0.993	0.603
pm32	0.998	0.864	0.998	0.334	0.998	0.381
pm33	0.990	0.240	0.991	0.981	0.994	0.997
pm34	1.079	0.102	1.053	0.177	1.048	0.868
pm35	0.718	<0.001	0.827	0.007	0.896	0.034
pm36	1.000	0.199	1.000	0.512	1.000	0.448
pm37	0.998	0.707	0.999	0.920	0.999	0.778

3a) Result of two-distribution perturbation analysis for hypertrophy when each response function was separately applied (1 million parameter sets)

No.	Pair of perturbed parameter distributions	Lin	Hill	Sat	Acc	Synergistic effect	p-value
1	pm1-pm2	1.001	0.984	1.017	0.995	-0.014	0.31
2	pm1-pm3	1.031	1.036	0.999	1.03	-0.001	0.782
3	pm1-pm4	1.011	1.099	1.025	1.01	0.002	0.895

4	pm1-pm5	1.013	1.001	1.015	0.978	-0.014	0.351
5	pm1-pm6	0.988	0.995	1.001	0.979	-0.02	0.314
6	pm1-pm7	2.826	2.074	2.686	2.258	-0.099	0.016
7	pm1-pm8	1.043	1.022	0.998	1.084	0.005	0.874
8	pm1-pm9	1.038	1.067	1.047	1.037	-0.005	0.72
9	pm1-pm10	1.466	1.557	1.262	1.473	-0.032	0.234
10	pm1-pm11	1.017	0.96	0.993	1.002	-0.017	0.353
11	pm1-pm12	1.01	1.054	0.997	1.014	-0.007	0.49
12	pm1-pm13	0.943	0.924	0.905	0.864	0.037	0.233
13	pm1-pm14	0.922	0.907	0.953	0.91	0.01	0.315
14	pm1-pm15	1.083	1.222	1.127	0.989	-0.072	0.015
15	pm1-pm16	1.227	1.227	1.181	1.053	0.001	0.749
16	pm1-pm17	1.19	1.183	1.188	1.029	-0.042	0.183
17	pm1-pm18	1.028	1.032	1.033	1.025	0.007	0.408
18	pm1-pm19	0.987	1.065	1.027	1.028	-0.025	0.276
19	pm1-pm20	1.029	1.058	1.019	1.022	0.003	0.848
20	pm1-pm21	1.528	1.099	1.857	1.228	-0.015	0.381
21	pm1-pm22	1.033	1.055	1.037	0.966	0.02	0.219
22	pm1-pm23	1.012	1.045	1.024	1.002	0.022	0.205
23	pm1-pm24	1.013	1.064	0.998	1.008	0.002	0.662
24	pm1-pm25	1.024	1.05	1.015	1.016	0.015	0.39
25	pm1-pm26	0.994	1.031	1.017	0.988	-0.003	0.612
26	pm1-pm27	1.039	1.049	1.019	0.996	-0.019	0.331
27	pm1-pm28	1.025	1.064	1.006	1.011	0	0.648
28	pm1-pm29	1.013	1.068	1.012	1.041	0.015	0.394
29	pm1-pm30	1.05	1.016	1.021	0.983	-0.006	0.497
30	pm1-pm31	1.019	1.101	1.04	0.992	-0.011	0.306
31	pm1-pm32	1.01	1.039	0.995	1.083	-0.002	0.818
32	pm1-pm33	1.027	1.15	1.006	1.13	-0.018	0.334
33	pm1-pm34	0.982	1.043	0.956	1.108	-0.001	0.759

34	pm1-pm35	1.013	1.077	0.955	1.106	0	0.84
35	pm1-pm36	1.042	1.033	1.015	0.979	0.006	0.548
36	pm1-pm37	1.033	1.002	1.009	0.997	0	0.728
37	pm2-pm3	0.972	0.973	0.991	1.033	-0.018	0.39
38	pm2-pm4	0.975	1.07	1.013	1.001	-0.005	0.544
39	pm2-pm5	1.017	0.97	0.997	1.029	0.001	0.949
40	pm2-pm6	1.019	0.971	0.988	0.985	-0.006	0.455
41	pm2-pm7	2.838	1.959	2.588	2.249	-0.137	0.008
42	pm2-pm8	1.008	1.004	0.994	1.043	-0.005	0.583
43	pm2-pm9	1.032	1.063	1.005	1.056	0.001	0.827
44	pm2-pm10	1.426	1.511	1.276	1.484	-0.033	0.268
45	pm2-pm11	1.004	0.986	0.981	1.021	0.002	0.994
46	pm2-pm12	0.997	1.04	0.993	1.003	-0.003	0.643
47	pm2-pm13	0.886	0.841	0.929	0.864	0.022	0.249
48	pm2-pm14	0.898	0.85	0.939	0.895	-0.003	0.661
49	pm2-pm15	1.069	1.19	1.133	1.034	-0.057	0.044
50	pm2-pm16	1.124	1.154	1.147	1.063	-0.036	0.296
51	pm2-pm17	1.14	1.171	1.187	1.035	-0.042	0.176
52	pm2-pm18	1.013	1.01	0.984	1.016	-0.003	0.719
53	pm2-pm19	0.992	1.049	1.03	1.033	-0.012	0.389
54	pm2-pm20	1.018	1.009	1.006	1.053	0.007	0.43
55	pm2-pm21	1.561	1.074	1.874	1.222	0.004	0.83
56	pm2-pm22	0.996	1.035	0.993	0.989	0.015	0.379
57	pm2-pm23	0.991	1.043	0.98	1.005	0.019	0.383
58	pm2-pm24	1.01	1.042	0.989	0.984	0.001	0.604
59	pm2-pm25	0.989	1.005	0.995	1.011	0.003	0.965
60	pm2-pm26	1.004	0.997	1.003	1.019	0.009	0.448
61	pm2-pm27	1.011	1.07	1.007	0.988	-0.012	0.335
62	pm2-pm28	1.016	1.019	1.013	1.027	0.006	0.516
63	pm2-pm29	1	1.021	0.973	0.992	-0.008	0.437

64	pm2-pm30	1.034	1.055	0.991	0.999	0.011	0.372
65	pm2-pm31	1.042	1.113	1.047	1.014	0.019	0.365
66	pm2-pm32	1	1.015	1.025	1.066	0.007	0.42
67	pm2-pm33	1.024	1.162	1.005	1.136	0	0.735
68	pm2-pm34	1.002	1.047	0.952	1.06	0.006	0.506
69	pm2-pm35	1	1.025	0.968	1.121	0.005	0.904
70	pm2-pm36	1.009	1.032	1	1.017	0.017	0.333
71	pm2-pm37	1.025	1.017	0.995	1.02	0.018	0.367
72	pm3-pm4	1.001	1.104	1	1.025	0.001	0.997
73	pm3-pm5	0.994	1.021	1.003	1.018	-0.004	0.989
74	pm3-pm6	1.027	0.99	0.993	1.033	0.003	0.883
75	pm3-pm7	2.869	1.965	2.668	2.247	-0.12	0.01
76	pm3-pm8	1.013	1.012	0.991	1.078	-0.006	0.595
77	pm3-pm9	1.055	1.076	1.064	1.051	0.012	0.399
78	pm3-pm10	1.466	1.487	1.216	1.544	-0.04	0.186
79	pm3-pm11	0.99	1.014	0.975	1.013	-0.009	0.433
80	pm3-pm12	1.027	1.045	1.022	1.025	0.007	0.439
81	pm3-pm13	0.948	0.873	0.904	0.849	0.024	0.274
82	pm3-pm14	0.911	0.957	0.901	0.888	0.004	0.931
83	pm3-pm15	1.106	1.203	1.097	1.062	-0.057	0.039
84	pm3-pm16	1.176	1.174	1.146	1.104	-0.019	0.333
85	pm3-pm17	1.16	1.203	1.173	1.098	-0.029	0.267
86	pm3-pm18	1.019	1.01	1	1.025	-0.006	0.506
87	pm3-pm19	1.008	1.054	1.047	1.026	-0.016	0.334
88	pm3-pm20	1.022	0.987	1.013	1.03	-0.013	0.345
89	pm3-pm21	1.512	1.155	1.821	1.201	-0.018	0.34
90	pm3-pm22	0.975	1.064	0.96	0.993	-0.002	0.969
91	pm3-pm23	0.987	1.071	0.987	1.026	0.021	0.257
92	pm3-pm24	0.972	1.05	1.009	1.041	0.002	0.687
93	pm3-pm25	1.017	0.99	0.975	0.998	-0.013	0.371

94	pm3-pm26	1.016	1.012	0.996	1.029	0.005	0.468
95	pm3-pm27	1	1.066	0.998	1.035	-0.017	0.368
96	pm3-pm28	0.996	1.02	1.006	1.031	-0.011	0.358
97	pm3-pm29	1.02	1.038	1.031	1.04	0.016	0.346
98	pm3-pm30	1.043	1.055	0.986	1.022	0.006	0.465
99	pm3-pm31	1.024	1.056	1.004	1.027	-0.018	0.32
100	pm3-pm32	1.008	1.018	0.965	1.103	-0.007	0.567
101	pm3-pm33	0.981	1.106	0.993	1.202	-0.022	0.285
102	pm3-pm34	1.035	1.04	0.936	1.103	0.008	0.524
103	pm3-pm35	1.041	1.025	0.937	1.117	-0.005	0.425
104	pm3-pm36	1.016	1.002	0.992	1.011	-0.003	0.817
105	pm3-pm37	0.998	1.007	0.981	1.004	-0.01	0.371
106	pm4-pm5	1.014	1.11	0.999	1.023	0.014	0.388
107	pm4-pm6	1.006	1.095	1.007	1.013	0.013	0.308
108	pm4-pm7	2.81	2.098	2.643	2.296	-0.105	0.006
109	pm4-pm8	1.019	1.092	1.006	1.031	-0.002	0.853
110	pm4-pm9	1.055	1.15	1.036	1.051	0.014	0.324
111	pm4-pm10	1.44	1.561	1.263	1.541	-0.027	0.266
112	pm4-pm11	1.014	1.073	0.997	1.015	0.008	0.58
113	pm4-pm12	1.008	1.107	1.013	1.023	0.006	0.517
114	pm4-pm13	0.888	0.938	0.942	0.879	0.033	0.299
115	pm4-pm14	0.917	0.936	0.938	0.908	0.005	0.484
116	pm4-pm15	1.098	1.267	1.128	1.022	-0.055	0.04
117	pm4-pm16	1.161	1.267	1.153	1.086	-0.011	0.341
118	pm4-pm17	1.166	1.23	1.221	1.079	-0.023	0.224
119	pm4-pm18	1.015	1.091	1.01	1.041	0.01	0.578
120	pm4-pm19	0.988	1.107	1.047	1.047	-0.012	0.362
121	pm4-pm20	1.033	1.109	1.003	1.015	0.004	0.613
122	pm4-pm21	1.511	1.164	1.818	1.188	-0.03	0.224
123	pm4-pm22	1.002	1.131	0.991	1	0.021	0.2

124	pm4-pm23	1.015	1.139	1.017	1.025	0.043	0.165
125	pm4-pm24	1.005	1.14	1.021	1.004	0.017	0.334
126	pm4-pm25	0.994	1.104	1.011	0.992	0.008	0.489
127	pm4-pm26	0.993	1.115	0.985	1.013	0.009	0.43
128	pm4-pm27	1.004	1.13	1.022	1.027	-0.006	0.6
129	pm4-pm28	1.012	1.102	1.001	1.025	0.001	0.748
130	pm4-pm29	1.014	1.16	1.002	1.042	0.029	0.244
131	pm4-pm30	1.016	1.092	1.01	1.022	0.005	0.731
132	pm4-pm31	1.034	1.183	1.018	1.033	0.011	0.321
133	pm4-pm32	0.988	1.094	0.998	1.038	-0.011	0.342
134	pm4-pm33	0.996	1.158	0.969	1.203	-0.021	0.291
135	pm4-pm34	0.985	1.141	0.926	1.079	0.003	0.833
136	pm4-pm35	1.018	1.102	0.933	1.131	0.001	0.793
137	pm4-pm36	0.996	1.042	1.008	0.98	-0.012	0.308
138	pm4-pm37	1.009	1.075	1.007	1.015	0.009	0.472
139	pm5-pm6	0.981	0.984	1.003	0.998	-0.007	0.479
140	pm5-pm7	2.845	2.009	2.656	2.267	-0.104	0.009
141	pm5-pm8	1.039	1.003	0.994	1.058	0.003	0.878
142	pm5-pm9	1.076	1.084	1.035	1.013	0.011	0.341
143	pm5-pm10	1.431	1.486	1.212	1.488	-0.056	0.021
144	pm5-pm11	1.014	0.99	0.99	1.019	0.005	0.693
145	pm5-pm12	1.017	1.027	0.986	1.014	-0.003	0.942
146	pm5-pm13	0.923	0.848	0.907	0.836	0.018	0.377
147	pm5-pm14	0.948	0.92	0.956	0.913	0.034	0.282
148	pm5-pm15	1.122	1.145	1.121	1.011	-0.066	0.035
149	pm5-pm16	1.136	1.167	1.122	1.071	-0.035	0.25
150	pm5-pm17	1.16	1.145	1.21	1.044	-0.038	0.243
151	pm5-pm18	1.049	0.987	0.995	1.016	0.001	0.609
152	pm5-pm19	1.005	1.009	1.008	1.059	-0.02	0.278
153	pm5-pm20	1.021	1.036	1.007	1.016	0.003	0.965

154	pm5-pm21	1.534	1.093	1.86	1.208	-0.008	0.523
155	pm5-pm22	1.01	1.017	1.02	1.025	0.027	0.28
156	pm5-pm23	1.03	1.075	1.005	1.016	0.044	0.179
157	pm5-pm24	0.974	1.046	0.986	1.013	-0.002	0.842
158	pm5-pm25	1.001	0.966	1.018	0.998	-0.003	0.893
159	pm5-pm26	0.99	0.994	1.006	1.005	0	0.69
160	pm5-pm27	1.018	1.081	1.025	1.024	0.004	0.828
161	pm5-pm28	1.009	0.992	1.012	1.033	-0.004	0.836
162	pm5-pm29	1.005	1.037	0.982	1.015	0.003	0.722
163	pm5-pm30	1.063	1.028	1.009	1.027	0.02	0.21
164	pm5-pm31	1.031	1.059	1.034	1.015	-0.002	0.967
165	pm5-pm32	1.01	0.988	1.002	1.082	-0.001	0.759
166	pm5-pm33	1.022	1.151	0.996	1.137	-0.007	0.501
167	pm5-pm34	0.994	1.015	0.95	1.069	-0.004	0.633
168	pm5-pm35	1.009	1.035	0.939	1.117	-0.001	0.78
169	pm5-pm36	1.002	0.989	0.98	0.994	-0.009	0.538
170	pm5-pm37	1.012	0.975	1.004	1.031	0.007	0.433
171	pm6-pm7	2.818	2.029	2.689	2.235	-0.1	0.01
172	pm6-pm8	1.035	1.012	1.009	1.043	0.01	0.582
173	pm6-pm9	1.068	1.065	1.007	1.025	0.006	0.475
174	pm6-pm10	1.402	1.488	1.262	1.508	-0.04	0.22
175	pm6-pm11	0.977	1.009	1.015	0.998	0.006	0.505
176	pm6-pm12	0.998	1.029	1.001	1.006	0	0.663
177	pm6-pm13	0.85	0.865	0.927	0.831	0.013	0.333
178	pm6-pm14	0.93	0.899	0.899	0.872	0.004	0.975
179	pm6-pm15	1.101	1.208	1.118	0.999	-0.054	0.04
180	pm6-pm16	1.151	1.156	1.182	1.058	-0.018	0.319
181	pm6-pm17	1.14	1.204	1.194	1.035	-0.03	0.243
182	pm6-pm18	1.042	1.013	0.999	1.045	0.019	0.342
183	pm6-pm19	0.996	1.108	1.029	1.032	0.006	0.48

184	pm6-pm20	1.04	1.018	1.011	1.016	0.009	0.428
185	pm6-pm21	1.51	1.173	1.877	1.225	0.02	0.209
186	pm6-pm22	0.983	1.058	1.011	0.977	0.022	0.272
187	pm6-pm23	0.987	1.045	0.993	0.968	0.016	0.352
188	pm6-pm24	1.007	1.003	0.985	0.985	-0.007	0.545
189	pm6-pm25	1.015	1.01	1.011	0.99	0.013	0.37
190	pm6-pm26	1.01	0.989	1.02	1.015	0.015	0.377
191	pm6-pm27	1.025	1.045	1.035	0.998	-0.002	0.659
192	pm6-pm28	1.033	1.019	1.011	1.019	0.01	0.322
193	pm6-pm29	0.982	1.035	0.989	1.01	0.002	0.85
194	pm6-pm30	1.04	1.044	1.021	1.013	0.023	0.274
195	pm6-pm31	1.039	1.089	1.034	1.021	0.014	0.359
196	pm6-pm32	0.994	1.026	1.007	1.078	0.01	0.581
197	pm6-pm33	1.015	1.115	0.984	1.179	-0.006	0.536
198	pm6-pm34	0.997	1.001	0.921	1.09	-0.004	0.833
199	pm6-pm35	0.999	1.062	0.935	1.134	0.012	0.375
200	pm6-pm36	1.004	0.987	0.983	0.986	-0.005	0.697
201	pm6-pm37	0.986	0.976	1	0.99	-0.006	0.571
202	pm7-pm8	2.855	2.001	2.605	2.303	-0.124	0.01
203	pm7-pm9	2.923	2.136	2.703	2.264	-0.079	0.015
204	pm7-pm10	3.856	3.011	3.241	3.323	0.354	<0.001
205	pm7-pm11	2.832	1.996	2.606	2.252	-0.121	0.008
206	pm7-pm12	2.805	2.032	2.617	2.279	-0.125	0.005
207	pm7-pm13	2.873	2.13	2.812	2.331	0.132	0.007
208	pm7-pm14	2.983	2.183	2.909	2.382	0.169	0.003
209	pm7-pm15	3.058	2.416	2.904	2.343	-0.03	0.278
210	pm7-pm16	3.168	2.317	3.092	2.388	0.037	0.285
211	pm7-pm17	3.203	2.306	3.132	2.354	0.026	0.251
212	pm7-pm18	2.813	2.024	2.645	2.303	-0.109	0.007
213	pm7-pm19	2.803	2.011	2.739	2.271	-0.129	0.007

214	pm7-pm20	2.855	2.017	2.654	2.252	-0.117	0.007
215	pm7-pm21	4.082	2.207	4.566	2.705	0.414	<0.001
216	pm7-pm22	2.757	2.069	2.671	2.285	-0.09	0.015
217	pm7-pm23	2.804	2.103	2.634	2.263	-0.081	0.015
218	pm7-pm24	2.84	2.027	2.625	2.211	-0.126	0.009
219	pm7-pm25	2.792	2.044	2.633	2.233	-0.118	0.01
220	pm7-pm26	2.734	2.019	2.64	2.266	-0.129	0.005
221	pm7-pm27	2.83	2.096	2.699	2.222	-0.116	0.007
222	pm7-pm28	2.876	2.045	2.652	2.257	-0.102	0.009
223	pm7-pm29	2.852	2.015	2.646	2.266	-0.107	0.005
224	pm7-pm30	2.81	2.065	2.617	2.275	-0.114	0.009
225	pm7-pm31	2.897	2.169	2.685	2.295	-0.07	0.012
226	pm7-pm32	2.845	2.014	2.641	2.364	-0.1	0.008
227	pm7-pm33	2.807	2.247	2.737	2.508	-0.054	0.043
228	pm7-pm34	2.882	2.065	2.537	2.304	-0.109	0.007
229	pm7-pm35	2.902	2.128	2.528	2.485	-0.06	0.021
230	pm7-pm36	2.859	1.908	2.673	2.244	-0.123	0.01
231	pm7-pm37	2.824	1.944	2.672	2.212	-0.13	0.01
232	pm8-pm9	1.043	1.122	1.029	1.106	0.018	0.332
233	pm8-pm10	1.439	1.456	1.268	1.554	-0.047	0.18
234	pm8-pm11	1.03	0.994	0.998	1.065	0.007	0.512
235	pm8-pm12	1.042	1.036	0.998	1.03	-0.003	0.887
236	pm8-pm13	0.954	0.874	0.923	0.865	0.027	0.28
237	pm8-pm14	0.927	0.855	0.945	0.914	-0.007	0.489
238	pm8-pm15	1.097	1.194	1.101	1.066	-0.067	0.032
239	pm8-pm16	1.181	1.186	1.132	1.133	-0.018	0.349
240	pm8-pm17	1.176	1.175	1.175	1.087	-0.041	0.16
241	pm8-pm18	1.044	1.025	1.019	1.064	0.011	0.361
242	pm8-pm19	0.989	1.062	1.037	1.099	-0.01	0.588
243	pm8-pm20	1.053	1.056	1.011	1.047	0.008	0.415

244	pm8-pm21	1.536	1.115	1.842	1.24	-0.015	0.363
245	pm8-pm22	1.051	1.079	0.999	1.029	0.032	0.237
246	pm8-pm23	0.988	1.076	0.983	1.016	0.012	0.346
247	pm8-pm24	1.018	1.037	1.006	1.026	-0.001	0.885
248	pm8-pm25	1.029	1.009	1.002	1.051	0.007	0.569
249	pm8-pm26	1.009	1.017	1.013	1.065	0.011	0.334
250	pm8-pm27	1.039	1.043	1.019	1.032	-0.016	0.397
251	pm8-pm28	1.014	1.005	0.994	1.059	-0.014	0.323
252	pm8-pm29	1.039	1.092	1.029	1.062	0.032	0.279
253	pm8-pm30	1.062	1.025	1.004	1.038	0.004	0.909
254	pm8-pm31	1.077	1.122	1.066	1.031	0.021	0.214
255	pm8-pm32	0.998	1.065	0.976	1.146	0.008	0.405
256	pm8-pm33	0.987	1.134	1.018	1.203	-0.015	0.324
257	pm8-pm34	1.005	1.015	0.917	1.101	-0.018	0.391
258	pm8-pm35	1.036	1.059	0.976	1.183	0.021	0.202
259	pm8-pm36	1.021	0.982	1.026	1.034	-0.001	0.81
260	pm8-pm37	1.025	1.02	0.995	1.079	0.015	0.38
261	pm9-pm10	1.455	1.572	1.296	1.512	-0.038	0.245
262	pm9-pm11	1.089	1.064	1.014	1.024	0.013	0.392
263	pm9-pm12	1.084	1.116	1.029	1.028	0.014	0.327
264	pm9-pm13	0.937	0.916	0.912	0.894	0.018	0.343
265	pm9-pm14	0.958	0.905	0.943	0.911	-0.008	0.583
266	pm9-pm15	1.174	1.268	1.174	1.083	-0.027	0.255
267	pm9-pm16	1.232	1.252	1.184	1.097	-0.005	0.959
268	pm9-pm17	1.224	1.207	1.256	1.088	-0.021	0.249
269	pm9-pm18	1.069	1.121	1.016	1.07	0.021	0.282
270	pm9-pm19	1.045	1.12	1.043	1.093	-0.002	0.996
271	pm9-pm20	1.073	1.076	1.001	1.043	-0.006	0.547
272	pm9-pm21	1.627	1.177	1.892	1.291	0.029	0.298
273	pm9-pm22	1.062	1.147	1.003	1.008	0.027	0.225

274	pm9-pm23	1.065	1.122	1.008	1.048	0.037	0.216
275	pm9-pm24	1.038	1.143	1.023	1.019	0.012	0.343
276	pm9-pm25	1.069	1.094	1.042	1.032	0.023	0.235
277	pm9-pm26	1.041	1.064	1.025	1.039	0.006	0.589
278	pm9-pm27	1.07	1.182	1.072	1.056	0.025	0.265
279	pm9-pm28	1.06	1.117	0.998	1.044	0.003	0.631
280	pm9-pm29	1.062	1.157	1.041	1.018	0.026	0.287
281	pm9-pm30	1.097	1.105	1.021	1.063	0.023	0.259
282	pm9-pm31	1.081	1.182	1.042	1.042	0.013	0.373
283	pm9-pm32	1.065	1.113	1.011	1.08	0.009	0.478
284	pm9-pm33	1.024	1.16	0.982	1.147	-0.043	0.185
285	pm9-pm34	1.012	1.116	0.981	1.109	0.007	0.541
286	pm9-pm35	1.069	1.141	0.995	1.107	0.015	0.302
287	pm9-pm36	1.046	1.08	1.046	1.036	0.015	0.357
288	pm9-pm37	1.052	1.061	1.014	1.031	0.004	0.841
289	pm10-pm11	1.479	1.527	1.257	1.488	-0.017	0.338
290	pm10-pm12	1.454	1.59	1.258	1.502	-0.019	0.365
291	pm10-pm13	1.298	1.312	1.201	1.243	-0.053	0.041
292	pm10-pm14	1.529	1.501	1.384	1.51	0.124	0.008
293	pm10-pm15	1.569	1.806	1.379	1.527	-0.051	0.043
294	pm10-pm16	1.571	1.723	1.441	1.545	-0.046	0.151
295	pm10-pm17	1.624	1.708	1.477	1.543	-0.046	0.181
296	pm10-pm18	1.412	1.483	1.276	1.557	-0.035	0.272
297	pm10-pm19	1.437	1.606	1.317	1.541	-0.021	0.228
298	pm10-pm20	1.441	1.516	1.26	1.475	-0.05	0.029
299	pm10-pm21	1.989	1.577	2.148	1.71	-0.032	0.261
300	pm10-pm22	1.35	1.613	1.249	1.456	-0.03	0.285
301	pm10-pm23	1.417	1.497	1.252	1.46	-0.037	0.283
302	pm10-pm24	1.418	1.531	1.288	1.469	-0.037	0.293
303	pm10-pm25	1.459	1.474	1.252	1.512	-0.031	0.231

304	pm10-pm26	1.383	1.485	1.255	1.444	-0.063	0.036
305	pm10-pm27	1.429	1.602	1.285	1.525	-0.029	0.261
306	pm10-pm28	1.466	1.548	1.267	1.513	-0.023	0.278
307	pm10-pm29	1.411	1.547	1.261	1.465	-0.042	0.156
308	pm10-pm30	1.406	1.553	1.216	1.516	-0.045	0.186
309	pm10-pm31	1.468	1.643	1.301	1.513	-0.012	0.361
310	pm10-pm32	1.435	1.568	1.254	1.611	-0.011	0.315
311	pm10-pm33	1.45	1.61	1.269	1.721	-0.028	0.264
312	pm10-pm34	1.451	1.547	1.188	1.556	-0.032	0.232
313	pm10-pm35	1.442	1.554	1.208	1.594	-0.033	0.299
314	pm10-pm36	1.416	1.488	1.269	1.479	-0.043	0.169
315	pm10-pm37	1.429	1.432	1.275	1.475	-0.052	0.035
316	pm11-pm12	1.019	1.008	1.024	1.011	0.007	0.442
317	pm11-pm13	0.909	0.836	0.916	0.815	0.014	0.362
318	pm11-pm14	0.908	0.828	0.93	0.9	-0.004	0.937
319	pm11-pm15	1.112	1.182	1.115	1.058	-0.044	0.187
320	pm11-pm16	1.181	1.165	1.145	1.066	-0.015	0.36
321	pm11-pm17	1.174	1.155	1.202	1.032	-0.032	0.207
322	pm11-pm18	1.034	1.01	1.008	1.037	0.017	0.371
323	pm11-pm19	1.004	1.004	0.988	1.027	-0.029	0.227
324	pm11-pm20	1.028	1.028	0.997	1.005	0.003	0.694
325	pm11-pm21	1.51	1.098	1.843	1.185	-0.017	0.332
326	pm11-pm22	1.009	1.029	0.993	0.99	0.02	0.398
327	pm11-pm23	1.031	1.051	0.982	1.036	0.043	0.186
328	pm11-pm24	1.005	1.035	1.021	0.983	0.009	0.518
329	pm11-pm25	0.977	0.983	0.993	0.998	-0.006	0.57
330	pm11-pm26	1.016	1.01	0.987	1.019	0.014	0.372
331	pm11-pm27	1.028	1.04	1.028	1.009	-0.001	0.852
332	pm11-pm28	1.002	1.057	0.989	1.041	0.012	0.3
333	pm11-pm29	0.995	1.025	0.994	0.977	-0.004	0.785

334	pm11-pm30	1.044	1.003	0.986	1.003	0.003	0.68
335	pm11-pm31	1.041	1.061	1.023	1.021	0.005	0.886
336	pm11-pm32	0.994	1.031	0.997	1.05	0.001	0.816
337	pm11-pm33	1.008	1.103	1.008	1.202	0.001	0.655
338	pm11-pm34	0.988	1.057	0.904	1.043	-0.007	0.422
339	pm11-pm35	1.041	1.06	0.946	1.124	0.022	0.276
340	pm11-pm36	0.996	0.983	0.992	1.016	0.002	0.799
341	pm11-pm37	1.011	1.009	0.977	1	0.006	0.592
342	pm12-pm13	0.872	0.934	0.903	0.831	0.015	0.33
343	pm12-pm14	0.948	0.917	0.938	0.888	0.012	0.359
344	pm12-pm15	1.08	1.176	1.14	1.038	-0.067	0.038
345	pm12-pm16	1.174	1.214	1.162	1.089	-0.01	0.498
346	pm12-pm17	1.189	1.14	1.201	1.065	-0.039	0.266
347	pm12-pm18	1.046	1.047	1.015	1.024	0.012	0.35
348	pm12-pm19	1.012	1.037	1.006	1.032	-0.028	0.205
349	pm12-pm20	1.034	1.039	1.008	0.992	-0.009	0.446
350	pm12-pm21	1.523	1.167	1.803	1.234	-0.01	0.41
351	pm12-pm22	1.01	1.06	1.017	0.992	0.019	0.368
352	pm12-pm23	1.004	1.091	1.016	1.006	0.032	0.201
353	pm12-pm24	0.995	1.035	0.999	1.018	-0.005	0.523
354	pm12-pm25	0.996	1.005	1.013	1.017	-0.001	0.727
355	pm12-pm26	1.023	1.003	0.998	1.028	0.004	0.659
356	pm12-pm27	1.033	1.11	1.05	1.014	0.009	0.561
357	pm12-pm28	1.025	1.03	0.99	1.021	-0.009	0.593
358	pm12-pm29	1.017	1.07	0.998	1.019	0.009	0.468
359	pm12-pm30	1.083	1.029	1.02	1.068	0.029	0.223
360	pm12-pm31	1.018	1.131	1.037	1.024	0.006	0.495
361	pm12-pm32	1.024	1.05	0.996	1.08	0.006	0.439
362	pm12-pm33	0.99	1.149	1.025	1.145	-0.017	0.335
363	pm12-pm34	0.975	1.063	0.911	1.04	-0.024	0.202

364	pm12-pm35	1.038	1.053	0.94	1.134	0.005	0.407
365	pm12-pm36	0.999	1.053	1.005	1.004	0.006	0.554
366	pm12-pm37	1.012	1.012	1.009	1.024	0.005	0.558
367	pm13-pm14	0.858	0.786	0.841	0.856	0.078	0.065
368	pm13-pm15	0.991	1.043	0.982	0.852	-0.055	0.032
369	pm13-pm16	1.058	0.944	1.04	0.902	-0.03	0.299
370	pm13-pm17	0.955	0.994	1.068	0.892	-0.057	0.021
371	pm13-pm18	0.906	0.937	0.897	0.838	0.027	0.255
372	pm13-pm19	0.905	0.908	0.973	0.865	0.016	0.391
373	pm13-pm20	0.907	0.889	0.881	0.847	0.007	0.429
374	pm13-pm21	1.436	0.959	1.661	1.043	-0.013	0.351
375	pm13-pm22	0.908	0.854	0.894	0.831	0.024	0.25
376	pm13-pm23	0.89	0.828	0.891	0.845	0.02	0.348
377	pm13-pm24	0.877	0.896	0.903	0.844	0.016	0.356
378	pm13-pm25	0.857	0.832	0.93	0.834	0.007	0.511
379	pm13-pm26	0.887	0.881	0.9	0.831	0.019	0.362
380	pm13-pm27	0.881	0.888	0.904	0.835	-0.013	0.317
381	pm13-pm28	0.919	0.874	0.89	0.873	0.017	0.3
382	pm13-pm29	0.856	0.92	0.927	0.872	0.03	0.276
383	pm13-pm30	0.878	0.855	0.894	0.875	0.007	0.431
384	pm13-pm31	0.933	0.947	0.945	0.848	0.024	0.252
385	pm13-pm32	0.92	0.867	0.873	0.919	0.016	0.304
386	pm13-pm33	0.906	0.905	0.877	0.948	-0.032	0.216
387	pm13-pm34	0.88	0.909	0.824	0.889	0.008	0.509
388	pm13-pm35	0.887	0.891	0.837	0.969	0.013	0.392
389	pm13-pm36	0.874	0.847	0.88	0.843	0.004	0.883
390	pm13-pm37	0.905	0.839	0.876	0.831	0.007	0.437
391	pm14-pm15	1.026	1.08	1.031	0.907	-0.052	0.038
392	pm14-pm16	1.044	1.006	1.065	0.921	-0.048	0.168
393	pm14-pm17	1.042	0.974	1.112	0.92	-0.063	0.037

394	pm14-pm18	0.913	0.896	0.912	0.869	-0.01	0.358
395	pm14-pm19	0.918	0.95	0.955	0.906	-0.005	0.588
396	pm14-pm20	0.909	0.941	0.93	0.905	0.007	0.511
397	pm14-pm21	1.416	0.983	1.718	1.104	-0.023	0.258
398	pm14-pm22	0.892	0.942	0.929	0.876	0.022	0.247
399	pm14-pm23	0.879	0.938	0.899	0.884	0.016	0.372
400	pm14-pm24	0.93	0.898	0.911	0.872	-0.001	0.994
401	pm14-pm25	0.924	0.862	0.93	0.869	0	0.777
402	pm14-pm26	0.931	0.926	0.9	0.886	0.015	0.391
403	pm14-pm27	0.939	0.982	0.958	0.919	0.019	0.356
404	pm14-pm28	0.942	0.826	0.933	0.888	-0.015	0.312
405	pm14-pm29	0.911	0.88	0.89	0.878	-0.014	0.349
406	pm14-pm30	0.939	0.883	0.894	0.866	-0.013	0.397
407	pm14-pm31	0.915	0.957	0.999	0.921	0.014	0.392
408	pm14-pm32	0.887	0.937	0.945	0.957	0.012	0.37
409	pm14-pm33	0.902	0.989	0.922	0.954	-0.039	0.288
410	pm14-pm34	0.928	0.892	0.873	0.912	-0.007	0.595
411	pm14-pm35	0.927	0.867	0.919	0.964	-0.004	0.8
412	pm14-pm36	0.934	0.852	0.91	0.894	0	0.867
413	pm14-pm37	0.928	0.892	0.944	0.858	0.01	0.464
414	pm15-pm16	1.272	1.398	1.223	1.121	-0.068	0.043
415	pm15-pm17	1.233	1.425	1.296	1.092	-0.078	0.014
416	pm15-pm18	1.136	1.165	1.104	1.018	-0.067	0.037
417	pm15-pm19	1.113	1.256	1.168	1.078	-0.048	0.195
418	pm15-pm20	1.129	1.229	1.126	1.04	-0.048	0.151
419	pm15-pm21	1.697	1.319	2.116	1.269	0.007	0.426
420	pm15-pm22	1.113	1.226	1.125	1.037	-0.027	0.219
421	pm15-pm23	1.066	1.273	1.13	1.008	-0.029	0.259
422	pm15-pm24	1.099	1.239	1.128	1.011	-0.05	0.185
423	pm15-pm25	1.136	1.217	1.127	1.026	-0.034	0.274

424	pm15-pm26	1.133	1.196	1.124	1.07	-0.03	0.232
425	pm15-pm27	1.145	1.283	1.137	1.017	-0.049	0.17
426	pm15-pm28	1.092	1.223	1.125	1.073	-0.049	0.19
427	pm15-pm29	1.093	1.158	1.08	1.034	-0.077	0.017
428	pm15-pm30	1.138	1.235	1.125	1.096	-0.024	0.248
429	pm15-pm31	1.103	1.28	1.155	1.051	-0.051	0.028
430	pm15-pm32	1.078	1.252	1.093	1.104	-0.052	0.025
431	pm15-pm33	1.114	1.362	1.135	1.158	-0.053	0.021
432	pm15-pm34	1.137	1.232	1.047	1.066	-0.052	0.023
433	pm15-pm35	1.102	1.216	1.051	1.187	-0.049	0.156
434	pm15-pm36	1.11	1.227	1.113	0.989	-0.051	0.029
435	pm15-pm37	1.115	1.193	1.138	1.034	-0.041	0.179
436	pm16-pm17	1.5	1.527	1.555	1.373	0.155	0.002
437	pm16-pm18	1.182	1.215	1.152	1.047	-0.018	0.38
438	pm16-pm19	1.148	1.27	1.183	1.094	-0.022	0.279
439	pm16-pm20	1.166	1.189	1.122	1.077	-0.034	0.22
440	pm16-pm21	1.762	1.277	2.15	1.257	0.024	0.272
441	pm16-pm22	1.172	1.227	1.164	1.017	-0.001	0.736
442	pm16-pm23	1.145	1.207	1.15	1.071	0	0.904
443	pm16-pm24	1.116	1.248	1.162	1.072	-0.013	0.373
444	pm16-pm25	1.163	1.146	1.162	1.056	-0.023	0.216
445	pm16-pm26	1.156	1.148	1.133	1.057	-0.031	0.265
446	pm16-pm27	1.142	1.195	1.186	1.038	-0.048	0.167
447	pm16-pm28	1.169	1.216	1.15	1.112	-0.009	0.454
448	pm16-pm29	1.163	1.163	1.174	1.086	-0.016	0.353
449	pm16-pm30	1.139	1.141	1.126	1.059	-0.051	0.042
450	pm16-pm31	1.215	1.278	1.191	1.07	-0.004	0.916
451	pm16-pm32	1.127	1.163	1.153	1.168	-0.025	0.259
452	pm16-pm33	1.209	1.299	1.19	1.213	-0.012	0.399
453	pm16-pm34	1.157	1.229	1.127	1.127	-0.007	0.559

454	pm16-pm35	1.159	1.253	1.119	1.218	0.005	0.516
455	pm16-pm36	1.138	1.16	1.149	1.062	-0.028	0.207
456	pm16-pm37	1.143	1.151	1.141	1.041	-0.035	0.215
457	pm17-pm18	1.178	1.175	1.182	1.06	-0.036	0.248
458	pm17-pm19	1.154	1.235	1.223	1.097	-0.037	0.234
459	pm17-pm20	1.157	1.172	1.205	1.075	-0.039	0.237
460	pm17-pm21	1.816	1.32	2.212	1.17	0.024	0.254
461	pm17-pm22	1.162	1.216	1.208	1.005	-0.017	0.391
462	pm17-pm23	1.151	1.248	1.198	0.995	-0.013	0.324
463	pm17-pm24	1.168	1.16	1.178	1.015	-0.051	0.031
464	pm17-pm25	1.159	1.147	1.177	1.038	-0.043	0.189
465	pm17-pm26	1.128	1.212	1.19	1.019	-0.036	0.237
466	pm17-pm27	1.141	1.19	1.253	1.069	-0.044	0.178
467	pm17-pm28	1.161	1.201	1.187	1.044	-0.041	0.182
468	pm17-pm29	1.158	1.189	1.239	1.034	-0.026	0.298
469	pm17-pm30	1.163	1.151	1.211	1.032	-0.046	0.157
470	pm17-pm31	1.154	1.255	1.217	1.064	-0.039	0.262
471	pm17-pm32	1.117	1.15	1.202	1.114	-0.05	0.024
472	pm17-pm33	1.175	1.29	1.193	1.204	-0.043	0.162
473	pm17-pm34	1.155	1.186	1.146	1.101	-0.038	0.23
474	pm17-pm35	1.168	1.206	1.138	1.165	-0.031	0.22
475	pm17-pm36	1.129	1.15	1.23	1.021	-0.041	0.178
476	pm17-pm37	1.175	1.131	1.227	1.06	-0.025	0.291
477	pm18-pm19	1.005	1.019	1.051	1.055	-0.015	0.346
478	pm18-pm20	1.044	1.009	1.024	1.027	0.002	0.692
479	pm18-pm21	1.517	1.117	1.844	1.273	-0.001	0.823
480	pm18-pm22	1.037	1.033	0.986	1.027	0.023	0.269
481	pm18-pm23	1.006	1.079	1.005	1.01	0.031	0.29
482	pm18-pm24	1.041	1.062	0.983	1.002	0.008	0.531
483	pm18-pm25	1.015	1.009	1.02	1.03	0.012	0.313

484	pm18-pm26	1.017	0.986	0.986	1.054	0.004	0.836
485	pm18-pm27	1.024	1.067	1.012	1.009	-0.012	0.352
486	pm18-pm28	1.03	1.065	0.996	1.018	0.005	0.822
487	pm18-pm29	1.024	1.065	1.017	1.01	0.015	0.372
488	pm18-pm30	1.051	1.017	1.038	1.016	0.012	0.313
489	pm18-pm31	1.042	1.093	0.995	1.044	0	0.963
490	pm18-pm32	1.027	1.081	0.999	1.091	0.021	0.265
491	pm18-pm33	1.03	1.137	0.996	1.185	-0.004	0.782
492	pm18-pm34	1.018	1.035	0.896	1.127	0.001	0.671
493	pm18-pm35	1.061	1.05	0.941	1.16	0.02	0.342
494	pm18-pm36	1.01	1.036	0.987	1.043	0.012	0.388
495	pm18-pm37	1.026	1.012	1.009	1.022	0.011	0.375
496	pm19-pm20	0.988	1.109	1.01	1.037	-0.017	0.358
497	pm19-pm21	1.516	1.146	1.874	1.242	-0.023	0.244
498	pm19-pm22	1.028	1.024	1.019	1.04	0	0.635
499	pm19-pm23	0.995	1.085	0.995	1.064	0.011	0.381
500	pm19-pm24	1.001	1.047	1.049	1.024	-0.013	0.374
501	pm19-pm25	1.014	1.069	1.036	1.043	0.005	0.754
502	pm19-pm26	0.989	1.065	1.048	1.061	0.005	0.57
503	pm19-pm27	1.016	1.111	1.071	1.075	-0.001	0.645
504	pm19-pm28	0.999	1.055	1.035	1.086	-0.008	0.419
505	pm19-pm29	1.017	1.095	1.01	1.074	0.006	0.474
506	pm19-pm30	0.983	1.055	1.031	1.054	-0.017	0.368
507	pm19-pm31	1.014	1.112	1.047	1.027	-0.023	0.254
508	pm19-pm32	1.028	1.041	1.037	1.099	-0.007	0.428
509	pm19-pm33	1.007	1.188	1.013	1.164	-0.027	0.228
510	pm19-pm34	0.986	1.081	0.934	1.102	-0.022	0.248
511	pm19-pm35	1.032	1.145	0.972	1.123	0.005	0.488
512	pm19-pm36	0.99	1.018	1.025	1.034	-0.02	0.37
513	pm19-pm37	1.008	1.101	1.029	1.032	0.007	0.476

514	pm20-pm21	1.563	1.155	1.819	1.217	-0.006	0.487
515	pm20-pm22	1.006	1.089	0.988	0.998	0.016	0.355
516	pm20-pm23	1.012	1.087	1.004	1.037	0.034	0.248
517	pm20-pm24	0.998	1.044	0.998	1.006	-0.009	0.527
518	pm20-pm25	1.012	1.059	1.001	1.035	0.014	0.399
519	pm20-pm26	1.008	1.06	0.98	1.009	0.002	0.958
520	pm20-pm27	1.021	1.017	1.03	1.033	-0.021	0.212
521	pm20-pm28	1.022	1.064	1.002	1.052	0.006	0.437
522	pm20-pm29	1.003	1.049	0.973	1.019	-0.009	0.473
523	pm20-pm30	1.047	1.032	0.993	1.041	0.003	0.813
524	pm20-pm31	1.031	1.124	1.058	1.039	0.013	0.321
525	pm20-pm32	1.023	1.032	1.007	1.104	0.006	0.433
526	pm20-pm33	1.061	1.086	1.001	1.156	-0.021	0.264
527	pm20-pm34	1.019	1.034	0.911	1.064	-0.017	0.384
528	pm20-pm35	1.006	1.062	0.962	1.141	0.003	0.691
529	pm20-pm36	1.005	0.997	1.007	1.026	-0.004	0.981
530	pm20-pm37	0.992	1.04	1.012	0.998	-0.002	0.943
531	pm21-pm22	1.521	1.149	1.824	1.162	-0.005	0.831
532	pm21-pm23	1.532	1.151	1.841	1.191	0.014	0.331
533	pm21-pm24	1.574	1.131	1.816	1.203	-0.004	0.864
534	pm21-pm25	1.535	1.086	1.804	1.218	-0.016	0.378
535	pm21-pm26	1.515	1.124	1.814	1.206	-0.012	0.327
536	pm21-pm27	1.529	1.202	1.823	1.235	-0.013	0.307
537	pm21-pm28	1.566	1.126	1.864	1.243	0.007	0.417
538	pm21-pm29	1.545	1.095	1.793	1.186	-0.029	0.235
539	pm21-pm30	1.502	1.07	1.832	1.2	-0.038	0.214
540	pm21-pm31	1.537	1.159	1.844	1.24	-0.02	0.327
541	pm21-pm32	1.519	1.165	1.785	1.249	-0.02	0.303
542	pm21-pm33	1.55	1.21	1.883	1.39	-0.003	0.951
543	pm21-pm34	1.549	1.131	1.762	1.23	-0.021	0.266

544	pm21-pm35	1.524	1.173	1.778	1.365	0.007	0.433
545	pm21-pm36	1.502	1.086	1.823	1.213	-0.022	0.266
546	pm21-pm37	1.524	1.124	1.867	1.182	-0.002	0.641
547	pm22-pm23	1	1.061	0.988	0.985	0.034	0.211
548	pm22-pm24	0.981	1.095	1.007	0.986	0.023	0.236
549	pm22-pm25	0.998	1.033	1.026	0.991	0.026	0.258
550	pm22-pm26	1.031	1.033	1.001	0.986	0.027	0.23
551	pm22-pm27	1.009	1.092	1.007	1.03	0.014	0.328
552	pm22-pm28	1.015	1.065	1.005	1.033	0.027	0.281
553	pm22-pm29	1.002	1.08	0.977	1.017	0.025	0.288
554	pm22-pm30	0.99	1.088	0.967	1.007	0.015	0.367
555	pm22-pm31	1.062	1.124	1.01	0.987	0.022	0.257
556	pm22-pm32	1.008	1.081	0.988	1.039	0.02	0.245
557	pm22-pm33	1.003	1.145	0.989	1.172	0.007	0.511
558	pm22-pm34	0.984	1.061	0.952	1.057	0.016	0.338
559	pm22-pm35	1.019	1.075	0.982	1.113	0.034	0.224
560	pm22-pm36	1.003	1.029	0.98	0.973	0.009	0.464
561	pm22-pm37	0.98	1.049	1.001	1.001	0.022	0.241
562	pm23-pm24	1.028	1.152	0.987	0.923	0.032	0.281
563	pm23-pm25	0.989	1.044	0.996	1.012	0.028	0.206
564	pm23-pm26	1.009	1.031	1.016	1.003	0.032	0.291
565	pm23-pm27	1.012	1.159	1.036	0.97	0.028	0.247
566	pm23-pm28	1.019	1.069	0.997	1.031	0.03	0.203
567	pm23-pm29	1.014	1.049	0.98	0.991	0.018	0.312
568	pm23-pm30	0.996	1.068	0.976	0.983	0.011	0.378
569	pm23-pm31	1.043	1.168	1.035	1.003	0.042	0.153
570	pm23-pm32	0.987	1.075	0.997	1.093	0.033	0.239
571	pm23-pm33	1.01	1.186	1.002	1.134	0.015	0.332
572	pm23-pm34	0.947	1.094	0.939	1.023	0.006	0.556
573	pm23-pm35	1.008	1.087	0.933	1.119	0.027	0.269

574	pm23-pm36	1.006	1.067	1.004	1.014	0.039	0.283
575	pm23-pm37	0.981	1.083	1.023	0.979	0.034	0.233
576	pm24-pm25	1.008	1.057	1	0.994	0.012	0.334
577	pm24-pm26	1.004	1.042	1.012	1.002	0.013	0.356
578	pm24-pm27	0.984	1.077	1.047	0.98	-0.014	0.364
579	pm24-pm28	0.987	1.017	0.991	1.029	-0.013	0.368
580	pm24-pm29	0.985	1.074	0.993	0.974	-0.004	0.841
581	pm24-pm30	1.029	1.064	0.98	1.007	0.005	0.49
582	pm24-pm31	1.019	1.121	1.005	1.022	0.001	0.726
583	pm24-pm32	0.994	1.07	0.999	1.073	0.009	0.484
584	pm24-pm33	0.996	1.151	0.994	1.154	-0.013	0.303
585	pm24-pm34	1.016	1.045	0.945	1.056	0.001	0.999
586	pm24-pm35	1.021	1.114	0.904	1.118	0.01	0.556
587	pm24-pm36	0.976	1.027	1.014	1.014	0.005	0.833
588	pm24-pm37	1.019	1.014	1.003	1.01	0.01	0.526
589	pm25-pm26	0.984	1	0.996	1.01	0.003	0.89
590	pm25-pm27	0.987	1.039	1.015	1.003	-0.017	0.313
591	pm25-pm28	1.002	1.046	0.995	1.033	0.008	0.4
592	pm25-pm29	1.02	1.058	1.002	0.982	0.013	0.326
593	pm25-pm30	1.035	1.038	1.011	1.023	0.02	0.207
594	pm25-pm31	1.027	1.119	0.996	0.991	0.001	0.879
595	pm25-pm32	0.985	1.018	1.007	1.053	-0.001	0.683
596	pm25-pm33	1	1.115	0.991	1.118	-0.023	0.207
597	pm25-pm34	1.003	1.02	0.931	1.066	-0.001	0.733
598	pm25-pm35	1.034	1.077	0.96	1.135	0.03	0.26
599	pm25-pm36	0.997	1.002	1.015	0.987	0.005	0.502
600	pm25-pm37	0.996	1.006	1.006	1.021	0.013	0.392
601	pm26-pm27	1.022	1.045	1.041	1.013	0.002	0.698
602	pm26-pm28	0.992	0.988	0.991	1.041	-0.008	0.462
603	pm26-pm29	1	1.053	0.98	0.981	0.001	0.924

604	pm26-pm30	1.042	0.996	0.988	1.018	0.004	0.986
605	pm26-pm31	1.045	1.094	1.046	1.018	0.019	0.38
606	pm26-pm32	1.003	1.01	0.969	1.058	-0.007	0.402
607	pm26-pm33	1.021	1.139	0.967	1.155	-0.009	0.485
608	pm26-pm34	0.991	1.037	0.919	1.048	-0.008	0.498
609	pm26-pm35	1.029	1.017	0.939	1.137	0.009	0.516
610	pm26-pm36	0.986	1.016	1.014	0.991	0.006	0.476
611	pm26-pm37	0.991	0.996	1.006	0.992	0.002	0.804
612	pm27-pm28	0.999	1.098	1.046	1.025	-0.003	0.926
613	pm27-pm29	0.996	1.104	1.008	1.019	-0.005	0.769
614	pm27-pm30	1.013	1.062	1.016	1.021	-0.013	0.311
615	pm27-pm31	1.062	1.171	1.057	1.025	0.012	0.37
616	pm27-pm32	1.008	1.143	1.014	1.071	0.008	0.58
617	pm27-pm33	1.019	1.169	1.042	1.189	-0.009	0.421
618	pm27-pm34	1.015	1.11	0.977	1.057	-0.001	0.644
619	pm27-pm35	1.033	1.091	0.965	1.131	-0.001	0.997
620	pm27-pm36	1.022	1.023	1.036	1	-0.009	0.533
621	pm27-pm37	0.997	1.05	1.026	1	-0.01	0.51
622	pm28-pm29	1.015	1.039	1.014	1.028	0.005	0.57
623	pm28-pm30	1.053	1.048	1.006	1.058	0.018	0.398
624	pm28-pm31	1.039	1.114	1.031	1.029	0.005	0.756
625	pm28-pm32	1.015	1.083	1.003	1.099	0.016	0.385
626	pm28-pm33	1.033	1.191	0.99	1.183	0.004	0.806
627	pm28-pm34	1	1.061	0.935	1.057	-0.009	0.465
628	pm28-pm35	1.006	1.076	0.952	1.136	0.005	0.82
629	pm28-pm36	1.01	1.018	1.026	1.009	0.004	0.971
630	pm28-pm37	1.012	1.019	0.987	1.045	0.006	0.541
631	pm29-pm30	1.022	1.047	0.991	1.012	0.003	0.618
632	pm29-pm31	1.024	1.173	1.058	1.014	0.027	0.258
633	pm29-pm32	0.994	1.046	0.997	1.084	0.005	0.58

634	pm29-pm33	1.018	1.136	1.012	1.167	-0.004	0.867
635	pm29-pm34	0.967	1.097	0.904	1.097	0.002	0.657
636	pm29-pm35	1.04	1.039	0.946	1.142	0.012	0.311
637	pm29-pm36	0.991	1.021	1.009	0.979	-0.003	0.85
638	pm29-pm37	1.01	1.061	0.971	1.013	0.012	0.335
639	pm30-pm31	1.043	1.113	1.048	1.019	0.011	0.39
640	pm30-pm32	1.016	1.062	0.992	1.066	0.004	0.932
641	pm30-pm33	1.056	1.138	1.01	1.178	0.004	0.958
642	pm30-pm34	1.008	1.047	0.914	1.043	-0.016	0.352
643	pm30-pm35	1.045	1.062	0.96	1.167	0.025	0.21
644	pm30-pm36	1.04	1.018	1.021	1.004	0.013	0.324
645	pm30-pm37	1.021	1.018	1.009	1.052	0.019	0.396
646	pm31-pm32	1.031	1.183	1.007	1.059	0.015	0.356
647	pm31-pm33	1.041	1.165	1.019	1.195	-0.012	0.385
648	pm31-pm34	1.034	1.15	0.966	1.068	0.01	0.349
649	pm31-pm35	1.036	1.135	0.953	1.134	0.005	0.501
650	pm31-pm36	1.023	1.081	1.01	0.994	-0.006	0.453
651	pm31-pm37	1.056	1.106	1.048	1.019	0.026	0.232
652	pm32-pm33	1.035	1.143	1.013	1.238	0.005	0.574
653	pm32-pm34	1.01	1.06	0.932	1.136	0.006	0.483
654	pm32-pm35	1.036	1.126	0.947	1.207	0.035	0.207
655	pm32-pm36	1.008	1.055	0.982	1.063	0.009	0.472
656	pm32-pm37	1.001	1.055	0.98	1.077	0.011	0.388
657	pm33-pm34	1.037	1.114	0.978	1.24	0.001	0.681
658	pm33-pm35	1.047	1.176	0.891	1.285	-0.006	0.582
659	pm33-pm36	0.993	1.101	0.994	1.175	-0.014	0.368
660	pm33-pm37	1.004	1.164	1.007	1.152	0.003	0.678
661	pm34-pm35	1.034	1.058	0.957	1.141	0.014	0.349
662	pm34-pm36	0.996	1.028	0.975	1.075	0.011	0.369
663	pm34-pm37	1.002	1.027	0.943	1.054	0	0.634

664	pm35-pm36	1.015	1.066	0.947	1.086	0.006	0.579
665	pm35-pm37	1	1.038	0.925	1.138	0.004	0.607
666	pm36-pm37	1.019	1.011	1.019	0.998	0.017	0.38

3b) Result of two-distribution perturbation analysis for hypertrophy when each response function was separately applied (10 million parameter sets)

No.	Pair of perturbed parameter distributions	Lin	Hill	Sat	Acc	Synergistic effect	p-value
1	pm1-pm2	0.995	0.984	1	1.004	-0.018	0.387
2	pm1-pm3	1.009	1.043	0.998	1.046	-0.001	0.822
3	pm1-pm4	0.992	1.114	1.001	1.007	-0.006	0.435
4	pm1-pm5	1.037	0.994	1.027	0.99	-0.003	0.753
5	pm1-pm6	1.004	1.006	0.997	1.002	-0.008	0.592
6	pm1-pm7	2.802	2.076	2.705	2.249	-0.102	0.006
7	pm1-pm8	1.05	1.05	1.028	1.085	0.021	0.217
8	pm1-pm9	1.055	1.078	1.038	1.03	-0.002	0.912
9	pm1-pm10	1.434	1.539	1.259	1.485	-0.042	0.196
10	pm1-pm11	1	0.998	1.01	1.012	-0.005	0.574
11	pm1-pm12	1.039	1.044	1.012	0.988	-0.005	0.712
12	pm1-pm13	0.93	0.921	0.917	0.84	0.03	0.218
13	pm1-pm14	0.935	0.891	0.94	0.911	0.007	0.488
14	pm1-pm15	1.107	1.217	1.129	1.005	-0.063	0.036
15	pm1-pm16	1.197	1.225	1.2	1.037	-0.007	0.559
16	pm1-pm17	1.165	1.179	1.181	1.052	-0.046	0.163
17	pm1-pm18	1.058	1.01	1.005	1.026	0.002	0.88
18	pm1-pm19	1.012	1.08	1.057	1.029	-0.007	0.483
19	pm1-pm20	1.033	1.086	1.028	1.003	0.009	0.442

20	pm1-pm21	1.516	1.117	1.842	1.204	-0.024	0.269
21	pm1-pm22	1.01	1.058	1.005	0.984	0.012	0.335
22	pm1-pm23	1.047	1.069	1.01	0.996	0.032	0.215
23	pm1-pm24	0.993	1.081	1.009	1.015	0.006	0.411
24	pm1-pm25	1.025	1.024	1.006	0.988	0	0.692
25	pm1-pm26	1.012	1.056	1.016	0.987	0.007	0.574
26	pm1-pm27	1.011	1.082	1.04	1.012	-0.008	0.543
27	pm1-pm28	1.009	1.037	1.023	1.008	-0.008	0.531
28	pm1-pm29	1.038	1.077	1.003	1.003	0.012	0.392
29	pm1-pm30	1.047	1.009	1.027	1.015	0.001	0.644
30	pm1-pm31	1.029	1.097	1.069	1.022	0.006	0.506
31	pm1-pm32	1.019	1.011	1.013	1.089	0	0.723
32	pm1-pm33	1.026	1.135	1.001	1.16	-0.015	0.368
33	pm1-pm34	0.971	1.039	0.941	1.084	-0.014	0.38
34	pm1-pm35	1.006	1.095	0.972	1.112	0.009	0.42
35	pm1-pm36	1.029	1.037	1.007	1.005	0.008	0.444
36	pm1-pm37	1.036	1.005	1.02	1.018	0.009	0.466
37	pm2-pm3	0.995	0.996	0.968	1.031	-0.013	0.373
38	pm2-pm4	0.966	1.058	1.005	1.016	-0.009	0.483
39	pm2-pm5	1.01	0.995	1.026	1.016	0.01	0.378
40	pm2-pm6	0.992	0.968	1.012	1.012	0	0.67
41	pm2-pm7	2.862	1.995	2.617	2.246	-0.116	0.006
42	pm2-pm8	1.001	1.019	1.012	1.029	-0.003	0.772
43	pm2-pm9	1.059	1.059	1.032	1.024	0.005	0.545
44	pm2-pm10	1.43	1.5	1.289	1.482	-0.032	0.222
45	pm2-pm11	1.008	0.985	0.993	1.009	0.002	0.845
46	pm2-pm12	1.009	1.023	1.003	0.973	-0.009	0.51
47	pm2-pm13	0.889	0.85	0.933	0.838	0.019	0.352
48	pm2-pm14	0.912	0.859	0.931	0.87	-0.006	0.543
49	pm2-pm15	1.082	1.175	1.134	1.034	-0.057	0.027

50	pm2-pm16	1.132	1.152	1.167	1.051	-0.032	0.254
51	pm2-pm17	1.169	1.182	1.198	1.034	-0.03	0.227
52	pm2-pm18	1.013	1.007	1.002	1.014	0	0.667
53	pm2-pm19	0.972	1.059	1.037	1.034	-0.013	0.337
54	pm2-pm20	1.012	1.023	0.992	1.057	0.006	0.55
55	pm2-pm21	1.545	1.099	1.882	1.235	0.011	0.395
56	pm2-pm22	0.996	1.024	0.984	0.986	0.009	0.557
57	pm2-pm23	1.022	1.063	0.976	0.986	0.027	0.202
58	pm2-pm24	0.979	1.016	0.998	0.988	-0.009	0.438
59	pm2-pm25	0.99	1.012	1.016	0.992	0.006	0.519
60	pm2-pm26	1.02	0.975	0.992	0.985	-0.004	0.6
61	pm2-pm27	1.027	1.056	1.042	1.022	0.006	0.508
62	pm2-pm28	1.025	1.028	1.022	1.038	0.015	0.31
63	pm2-pm29	0.989	1.021	0.98	1.004	-0.006	0.533
64	pm2-pm30	1.025	1.031	0.992	1.001	0.003	0.664
65	pm2-pm31	1.043	1.114	1.046	1.008	0.018	0.337
66	pm2-pm32	0.992	1.029	0.998	1.077	0.005	0.739
67	pm2-pm33	1.002	1.16	0.99	1.136	-0.01	0.498
68	pm2-pm34	0.977	1.015	0.958	1.069	-0.004	0.664
69	pm2-pm35	0.999	1.032	0.961	1.127	0.006	0.537
70	pm2-pm36	1.01	1.036	0.978	1.016	0.013	0.301
71	pm2-pm37	1.026	0.995	1.001	1.012	0.012	0.31
72	pm3-pm4	0.996	1.093	0.998	1.025	-0.003	0.835
73	pm3-pm5	1.029	1.012	0.984	1.02	-0.001	0.743
74	pm3-pm6	1.011	1.008	0.986	1.01	-0.004	0.845
75	pm3-pm7	2.893	1.954	2.658	2.247	-0.119	0.009
76	pm3-pm8	1.002	1.008	0.976	1.051	-0.02	0.386
77	pm3-pm9	1.079	1.066	1.043	1.053	0.01	0.372
78	pm3-pm10	1.458	1.498	1.233	1.564	-0.03	0.235
79	pm3-pm11	0.988	1.029	0.997	1.026	0.003	0.974

80	pm3-pm12	0.999	1.053	1.025	1.02	0.002	0.78
81	pm3-pm13	0.936	0.89	0.9	0.861	0.028	0.2
82	pm3-pm14	0.927	0.952	0.921	0.889	0.012	0.36
83	pm3-pm15	1.098	1.204	1.093	1.071	-0.058	0.033
84	pm3-pm16	1.156	1.152	1.174	1.115	-0.019	0.373
85	pm3-pm17	1.165	1.199	1.141	1.061	-0.045	0.168
86	pm3-pm18	1.051	1.01	0.968	1.042	-0.002	0.719
87	pm3-pm19	0.993	1.074	1.008	1.033	-0.022	0.247
88	pm3-pm20	1.032	1.013	0.985	1.051	-0.006	0.489
89	pm3-pm21	1.507	1.149	1.819	1.2	-0.022	0.272
90	pm3-pm22	0.984	1.045	0.995	1.014	0.01	0.478
91	pm3-pm23	1.002	1.069	0.995	1.029	0.028	0.218
92	pm3-pm24	0.995	1.05	1.007	1.018	0.001	0.91
93	pm3-pm25	1.012	0.982	0.978	0.992	-0.017	0.339
94	pm3-pm26	0.996	0.99	1.011	1.027	-0.002	0.98
95	pm3-pm27	1.011	1.041	1.001	1.033	-0.02	0.207
96	pm3-pm28	1.024	1.009	1.005	1.062	0	0.691
97	pm3-pm29	1.008	1.056	1.002	1.028	0.008	0.518
98	pm3-pm30	1.038	1.024	0.987	1.03	0	0.766
99	pm3-pm31	1.023	1.092	1.019	1.018	-0.008	0.454
100	pm3-pm32	1.002	1.039	0.983	1.105	0.001	0.968
101	pm3-pm33	1.01	1.112	0.994	1.182	-0.019	0.344
102	pm3-pm34	1.022	1.016	0.945	1.095	-0.001	0.958
103	pm3-pm35	1.065	1.033	0.909	1.109	-0.006	0.489
104	pm3-pm36	0.994	0.998	0.989	1.004	-0.013	0.309
105	pm3-pm37	1.006	1.041	0.985	1.026	0.007	0.582
106	pm4-pm5	1.03	1.114	0.972	1.016	0.011	0.363
107	pm4-pm6	0.994	1.104	1	1.036	0.016	0.309
108	pm4-pm7	2.835	2.119	2.628	2.33	-0.089	0.018
109	pm4-pm8	1.008	1.073	0.978	1.061	-0.009	0.598

110	pm4-pm9	1.076	1.142	1.052	1.046	0.02	0.291
111	pm4-pm10	1.448	1.57	1.258	1.518	-0.03	0.265
112	pm4-pm11	1.017	1.066	0.995	1.001	0.003	0.775
113	pm4-pm12	1.004	1.101	0.996	1.011	-0.004	0.68
114	pm4-pm13	0.888	0.94	0.91	0.865	0.021	0.257
115	pm4-pm14	0.936	0.925	0.966	0.874	0.006	0.593
116	pm4-pm15	1.105	1.288	1.116	1.036	-0.048	0.158
117	pm4-pm16	1.173	1.245	1.157	1.11	-0.007	0.548
118	pm4-pm17	1.145	1.23	1.203	1.069	-0.035	0.202
119	pm4-pm18	1.006	1.092	1.001	1.033	0.004	0.65
120	pm4-pm19	0.958	1.138	1.026	1.036	-0.019	0.332
121	pm4-pm20	1.029	1.1	1.006	1.045	0.009	0.445
122	pm4-pm21	1.524	1.168	1.818	1.183	-0.027	0.283
123	pm4-pm22	1.016	1.142	0.998	0.995	0.028	0.266
124	pm4-pm23	1.025	1.147	0.988	1.043	0.045	0.183
125	pm4-pm24	0.986	1.123	0.995	1.01	0.003	0.8
126	pm4-pm25	0.996	1.106	0.987	1.01	0.007	0.481
127	pm4-pm26	1.018	1.092	0.999	1.02	0.014	0.354
128	pm4-pm27	1.011	1.111	1.024	1.025	-0.009	0.503
129	pm4-pm28	1.003	1.103	1.003	1.034	0.002	0.633
130	pm4-pm29	1.02	1.171	0.972	1.044	0.026	0.214
131	pm4-pm30	1.02	1.092	0.998	1.025	0.004	0.657
132	pm4-pm31	1.044	1.184	1.046	1.045	0.024	0.29
133	pm4-pm32	0.999	1.081	1.005	1.054	-0.005	0.474
134	pm4-pm33	1.008	1.171	0.971	1.2	-0.015	0.316
135	pm4-pm34	1.012	1.164	0.959	1.065	0.02	0.228
136	pm4-pm35	1.02	1.125	0.957	1.12	0.011	0.359
137	pm4-pm36	1.01	1.06	1.015	0.993	0.001	0.984
138	pm4-pm37	1.026	1.074	0.994	1.029	0.013	0.394
139	pm5-pm6	0.982	1.014	1.02	1.011	0.008	0.455

140	pm5-pm7	2.827	1.983	2.656	2.272	-0.114	0.009
141	pm5-pm8	1.036	0.989	1.004	1.055	0.001	0.975
142	pm5-pm9	1.071	1.061	1.015	1.017	0	0.779
143	pm5-pm10	1.459	1.487	1.21	1.493	-0.048	0.182
144	pm5-pm11	0.996	0.981	1.005	1.006	-0.001	0.827
145	pm5-pm12	1.001	1.024	1.015	1.014	0	0.924
146	pm5-pm13	0.911	0.832	0.899	0.838	0.01	0.505
147	pm5-pm14	0.919	0.898	0.957	0.9	0.017	0.307
148	pm5-pm15	1.145	1.154	1.121	1.027	-0.054	0.025
149	pm5-pm16	1.128	1.139	1.125	1.1	-0.037	0.271
150	pm5-pm17	1.18	1.149	1.213	1.041	-0.032	0.244
151	pm5-pm18	1.036	0.987	0.985	1.019	-0.004	0.905
152	pm5-pm19	0.995	1.012	1.016	1.052	-0.022	0.269
153	pm5-pm20	1.01	1.043	1.018	1.026	0.007	0.587
154	pm5-pm21	1.55	1.106	1.835	1.222	-0.003	0.898
155	pm5-pm22	1.015	1.03	1.018	1.001	0.025	0.293
156	pm5-pm23	1.015	1.08	1.028	1.01	0.046	0.184
157	pm5-pm24	0.975	1.042	0.991	0.998	-0.006	0.595
158	pm5-pm25	0.989	0.983	0.998	1.006	-0.005	0.836
159	pm5-pm26	1.008	1	1.02	1.004	0.008	0.591
160	pm5-pm27	1.02	1.084	1.065	1.034	0.018	0.396
161	pm5-pm28	1.002	0.997	1.01	1.034	-0.005	0.985
162	pm5-pm29	1.023	1.036	0.984	1.032	0.012	0.388
163	pm5-pm30	1.05	1.058	0.988	0.996	0.011	0.325
164	pm5-pm31	1.028	1.092	1.051	1.009	0.008	0.459
165	pm5-pm32	0.993	1.018	1.019	1.086	0.007	0.464
166	pm5-pm33	1.016	1.165	0.965	1.142	-0.012	0.369
167	pm5-pm34	0.994	1.009	0.918	1.086	-0.009	0.566
168	pm5-pm35	1.038	1.036	0.924	1.153	0.012	0.366
169	pm5-pm36	1.017	1.009	0.992	0.995	0.003	0.912

170	pm5-pm37	1.011	0.966	1.007	1.006	-0.001	0.946
171	pm6-pm7	2.804	2.013	2.675	2.235	-0.111	0.006
172	pm6-pm8	1.032	1.014	0.996	1.068	0.012	0.389
173	pm6-pm9	1.068	1.094	1.019	1.034	0.018	0.362
174	pm6-pm10	1.432	1.467	1.281	1.527	-0.028	0.298
175	pm6-pm11	0.989	0.997	0.995	1.023	0.008	0.528
176	pm6-pm12	1.014	1.024	1.011	1.012	0.007	0.496
177	pm6-pm13	0.845	0.84	0.916	0.834	0.003	0.813
178	pm6-pm14	0.931	0.894	0.925	0.871	0.009	0.544
179	pm6-pm15	1.104	1.2	1.106	1.001	-0.057	0.033
180	pm6-pm16	1.16	1.169	1.194	1.056	-0.01	0.431
181	pm6-pm17	1.154	1.175	1.209	1.045	-0.027	0.299
182	pm6-pm18	1.032	1.023	0.988	1.023	0.011	0.369
183	pm6-pm19	0.999	1.106	1.033	1.004	0	0.841
184	pm6-pm20	1.037	1.054	1	1.005	0.012	0.372
185	pm6-pm21	1.54	1.157	1.885	1.239	0.029	0.271
186	pm6-pm22	0.982	1.036	0.974	1.002	0.013	0.333
187	pm6-pm23	0.994	1.067	0.984	0.974	0.023	0.212
188	pm6-pm24	0.976	1.012	0.983	1.004	-0.008	0.457
189	pm6-pm25	0.98	1.016	0.999	1.007	0.007	0.492
190	pm6-pm26	0.998	0.994	0.986	0.985	-0.003	0.898
191	pm6-pm27	1.037	1.071	1.026	1.02	0.01	0.346
192	pm6-pm28	1.013	1.012	0.991	1.042	0.004	0.916
193	pm6-pm29	0.989	1.039	0.988	1.015	0.006	0.585
194	pm6-pm30	1.033	1.036	1.009	1.032	0.021	0.27
195	pm6-pm31	1.06	1.078	1.028	1.003	0.01	0.349
196	pm6-pm32	0.99	1.036	1.003	1.047	0.002	0.752
197	pm6-pm33	0.998	1.114	1.01	1.186	-0.002	0.802
198	pm6-pm34	0.977	1.021	0.911	1.069	-0.011	0.332
199	pm6-pm35	1.023	1.061	0.965	1.111	0.019	0.337

200	pm6-pm36	0.995	1.017	0.978	1.014	0.006	0.483
201	pm6-pm37	0.989	1.005	1	0.999	0.005	0.616
202	pm7-pm8	2.857	2.013	2.619	2.304	-0.116	0.005
203	pm7-pm9	2.949	2.144	2.698	2.272	-0.069	0.031
204	pm7-pm10	3.888	3.013	3.25	3.287	0.355	<0.001
205	pm7-pm11	2.859	2.011	2.603	2.25	-0.112	0.006
206	pm7-pm12	2.812	2	2.61	2.296	-0.128	0.005
207	pm7-pm13	2.884	2.126	2.819	2.328	0.134	0.01
208	pm7-pm14	2.988	2.187	2.887	2.383	0.166	0.002
209	pm7-pm15	3.044	2.395	2.928	2.355	-0.03	0.269
210	pm7-pm16	3.16	2.335	3.106	2.392	0.044	0.165
211	pm7-pm17	3.174	2.29	3.16	2.328	0.016	0.38
212	pm7-pm18	2.828	2.051	2.658	2.294	-0.097	0.016
213	pm7-pm19	2.81	2.006	2.737	2.265	-0.13	0.006
214	pm7-pm20	2.862	2.035	2.631	2.253	-0.116	0.01
215	pm7-pm21	4.102	2.215	4.559	2.699	0.418	<0.001
216	pm7-pm22	2.775	2.062	2.635	2.297	-0.093	0.014
217	pm7-pm23	2.822	2.102	2.622	2.265	-0.079	0.014
218	pm7-pm24	2.845	2.01	2.637	2.226	-0.122	0.007
219	pm7-pm25	2.784	2.042	2.664	2.249	-0.109	0.009
220	pm7-pm26	2.739	2.01	2.633	2.246	-0.137	0.01
221	pm7-pm27	2.807	2.102	2.67	2.213	-0.13	0.006
222	pm7-pm28	2.887	2.027	2.638	2.272	-0.104	0.007
223	pm7-pm29	2.865	2.007	2.652	2.273	-0.102	0.008
224	pm7-pm30	2.833	2.055	2.61	2.246	-0.12	0.009
225	pm7-pm31	2.873	2.148	2.683	2.302	-0.08	0.011
226	pm7-pm32	2.833	2.051	2.645	2.357	-0.095	0.014
227	pm7-pm33	2.773	2.216	2.731	2.484	-0.077	0.013
228	pm7-pm34	2.882	2.077	2.553	2.31	-0.1	0.013
229	pm7-pm35	2.925	2.101	2.492	2.451	-0.078	0.011

230	pm7-pm36	2.86	1.923	2.675	2.22	-0.125	0.007
231	pm7-pm37	2.789	1.968	2.646	2.206	-0.141	0.008
232	pm8-pm9	1.077	1.099	1.037	1.073	0.014	0.344
233	pm8-pm10	1.437	1.467	1.279	1.541	-0.045	0.186
234	pm8-pm11	1.023	1.012	1.015	1.073	0.016	0.36
235	pm8-pm12	1.047	1.04	0.989	1.03	-0.003	0.605
236	pm8-pm13	0.926	0.876	0.931	0.878	0.026	0.246
237	pm8-pm14	0.927	0.877	0.944	0.912	-0.002	0.736
238	pm8-pm15	1.11	1.184	1.125	1.077	-0.058	0.044
239	pm8-pm16	1.186	1.17	1.144	1.127	-0.019	0.326
240	pm8-pm17	1.166	1.191	1.196	1.102	-0.03	0.244
241	pm8-pm18	1.054	1.003	1.008	1.069	0.006	0.524
242	pm8-pm19	1.012	1.079	1.051	1.1	0.004	0.757
243	pm8-pm20	1.044	1.02	1.004	1.038	-0.007	0.424
244	pm8-pm21	1.556	1.09	1.844	1.247	-0.013	0.333
245	pm8-pm22	1.07	1.06	0.982	1.022	0.026	0.201
246	pm8-pm23	1.025	1.057	1.007	1.042	0.029	0.223
247	pm8-pm24	0.999	1.042	0.991	1.021	-0.01	0.38
248	pm8-pm25	1.04	1.004	0.992	1.026	0	0.714
249	pm8-pm26	1.029	1.014	1.009	1.06	0.012	0.324
250	pm8-pm27	1.043	1.063	1.012	1.027	-0.013	0.336
251	pm8-pm28	1.004	1.026	1.016	1.071	-0.002	0.623
252	pm8-pm29	1.033	1.085	1.003	1.066	0.023	0.2
253	pm8-pm30	1.037	1.004	1.009	1.018	-0.011	0.318
254	pm8-pm31	1.051	1.102	1.036	1.049	0.006	0.442
255	pm8-pm32	1.032	1.064	1.013	1.151	0.027	0.299
256	pm8-pm33	1.008	1.129	1.004	1.206	-0.014	0.312
257	pm8-pm34	1.011	0.992	0.93	1.1	-0.019	0.321
258	pm8-pm35	1.042	1.05	0.973	1.171	0.016	0.353
259	pm8-pm36	1.037	0.992	1.02	1.035	0.005	0.925

260	pm8-pm37	1.039	0.993	1.008	1.058	0.009	0.56
261	pm9-pm10	1.492	1.575	1.306	1.492	-0.03	0.254
262	pm9-pm11	1.065	1.068	0.995	1.053	0.01	0.427
263	pm9-pm12	1.088	1.114	1.047	1.043	0.022	0.259
264	pm9-pm13	0.936	0.915	0.912	0.887	0.015	0.365
265	pm9-pm14	0.936	0.93	0.927	0.92	-0.009	0.462
266	pm9-pm15	1.147	1.266	1.172	1.064	-0.04	0.195
267	pm9-pm16	1.232	1.252	1.181	1.088	-0.008	0.524
268	pm9-pm17	1.202	1.207	1.265	1.11	-0.019	0.369
269	pm9-pm18	1.072	1.091	1.036	1.066	0.019	0.345
270	pm9-pm19	1.067	1.106	1.038	1.096	0	0.794
271	pm9-pm20	1.058	1.095	0.998	1.048	-0.004	0.806
272	pm9-pm21	1.623	1.205	1.888	1.263	0.026	0.243
273	pm9-pm22	1.044	1.155	1.024	1.018	0.032	0.232
274	pm9-pm23	1.078	1.13	1.029	1.029	0.042	0.193
275	pm9-pm24	1.052	1.136	1.002	1.027	0.01	0.386
276	pm9-pm25	1.072	1.09	1.043	1.012	0.018	0.373
277	pm9-pm26	1.043	1.079	1.019	1.039	0.009	0.526
278	pm9-pm27	1.081	1.145	1.058	1.042	0.012	0.394
279	pm9-pm28	1.081	1.107	1.005	1.034	0.005	0.799
280	pm9-pm29	1.066	1.146	1.053	1.021	0.028	0.2
281	pm9-pm30	1.088	1.129	1.022	1.047	0.023	0.297
282	pm9-pm31	1.057	1.194	1.06	1.046	0.016	0.311
283	pm9-pm32	1.063	1.098	1.011	1.116	0.013	0.329
284	pm9-pm33	1.022	1.164	1.005	1.165	-0.032	0.206
285	pm9-pm34	1.029	1.125	1.007	1.082	0.013	0.355
286	pm9-pm35	1.062	1.152	0.966	1.107	0.009	0.409
287	pm9-pm36	1.051	1.079	1.039	1.031	0.013	0.303
288	pm9-pm37	1.049	1.061	1.019	1.034	0.005	0.448
289	pm10-pm11	1.481	1.517	1.272	1.501	-0.012	0.307

290	pm10-pm12	1.43	1.596	1.275	1.531	-0.012	0.334
291	pm10-pm13	1.298	1.318	1.187	1.273	-0.048	0.186
292	pm10-pm14	1.518	1.489	1.39	1.49	0.115	0.008
293	pm10-pm15	1.573	1.81	1.389	1.544	-0.043	0.163
294	pm10-pm16	1.594	1.709	1.435	1.576	-0.037	0.224
295	pm10-pm17	1.64	1.68	1.488	1.54	-0.047	0.173
296	pm10-pm18	1.398	1.493	1.247	1.53	-0.05	0.029
297	pm10-pm19	1.435	1.61	1.303	1.536	-0.025	0.216
298	pm10-pm20	1.434	1.52	1.264	1.46	-0.054	0.037
299	pm10-pm21	1.997	1.582	2.133	1.677	-0.04	0.193
300	pm10-pm22	1.368	1.63	1.281	1.447	-0.016	0.39
301	pm10-pm23	1.435	1.475	1.281	1.455	-0.032	0.294
302	pm10-pm24	1.43	1.54	1.278	1.494	-0.027	0.247
303	pm10-pm25	1.461	1.488	1.251	1.505	-0.029	0.218
304	pm10-pm26	1.394	1.505	1.245	1.454	-0.056	0.039
305	pm10-pm27	1.462	1.604	1.299	1.509	-0.02	0.281
306	pm10-pm28	1.464	1.569	1.293	1.546	-0.004	0.681
307	pm10-pm29	1.435	1.541	1.289	1.47	-0.029	0.245
308	pm10-pm30	1.382	1.555	1.239	1.517	-0.044	0.153
309	pm10-pm31	1.476	1.632	1.284	1.514	-0.017	0.313
310	pm10-pm32	1.406	1.565	1.235	1.608	-0.024	0.243
311	pm10-pm33	1.438	1.603	1.266	1.718	-0.034	0.204
312	pm10-pm34	1.446	1.521	1.213	1.572	-0.029	0.28
313	pm10-pm35	1.447	1.537	1.179	1.62	-0.036	0.264
314	pm10-pm36	1.398	1.477	1.267	1.484	-0.049	0.166
315	pm10-pm37	1.419	1.454	1.267	1.456	-0.056	0.033
316	pm11-pm12	0.998	1.002	0.987	0.983	-0.016	0.344
317	pm11-pm13	0.91	0.858	0.903	0.819	0.018	0.366
318	pm11-pm14	0.932	0.834	0.907	0.879	-0.007	0.476
319	pm11-pm15	1.095	1.198	1.141	1.051	-0.039	0.285

320	pm11-pm16	1.195	1.159	1.177	1.088	0.001	0.666
321	pm11-pm17	1.167	1.175	1.24	1.033	-0.019	0.395
322	pm11-pm18	1.011	1.009	1.013	1.015	0.006	0.591
323	pm11-pm19	0.996	1.035	1.004	1.031	-0.018	0.382
324	pm11-pm20	1.025	1.024	1.009	1.03	0.01	0.373
325	pm11-pm21	1.518	1.124	1.808	1.204	-0.012	0.384
326	pm11-pm22	1.023	1.046	0.989	0.992	0.027	0.262
327	pm11-pm23	1.024	1.058	1.003	1.026	0.046	0.161
328	pm11-pm24	0.997	1.051	1.008	0.989	0.01	0.523
329	pm11-pm25	0.997	0.991	1.001	0.994	0.002	0.725
330	pm11-pm26	1.004	0.99	1.017	1	0.009	0.51
331	pm11-pm27	1.006	1.022	1.002	1.005	-0.019	0.343
332	pm11-pm28	1.031	1.055	0.981	1.029	0.014	0.35
333	pm11-pm29	0.988	1.02	1.008	0.979	-0.003	0.632
334	pm11-pm30	1.026	1.023	0.996	1.013	0.008	0.42
335	pm11-pm31	1.005	1.062	1.04	1.008	-0.003	0.961
336	pm11-pm32	1.005	1.02	0.98	1.061	0	0.771
337	pm11-pm33	0.994	1.102	0.986	1.205	-0.007	0.586
338	pm11-pm34	0.986	1.063	0.907	1.06	-0.002	0.69
339	pm11-pm35	1.023	1.051	0.932	1.114	0.009	0.41
340	pm11-pm36	0.985	1.006	0.998	1.008	0.005	0.626
341	pm11-pm37	0.979	0.998	0.978	1.002	-0.004	0.798
342	pm12-pm13	0.879	0.916	0.915	0.831	0.015	0.359
343	pm12-pm14	0.924	0.939	0.926	0.864	0.003	0.648
344	pm12-pm15	1.082	1.177	1.124	1.041	-0.07	0.04
345	pm12-pm16	1.171	1.192	1.137	1.099	-0.02	0.371
346	pm12-pm17	1.185	1.105	1.209	1.043	-0.052	0.023
347	pm12-pm18	1.037	1.023	1.004	1.006	-0.003	0.794
348	pm12-pm19	0.995	1.032	1.029	1.049	-0.024	0.291
349	pm12-pm20	1.038	1.036	1.004	1.025	-0.001	0.87

350	pm12-pm21	1.524	1.144	1.781	1.21	-0.027	0.28
351	pm12-pm22	1.008	1.026	0.985	1.016	0.008	0.51
352	pm12-pm23	1.031	1.069	0.996	1.026	0.033	0.286
353	pm12-pm24	0.996	1.048	1.004	1.003	-0.004	0.933
354	pm12-pm25	1.008	0.998	0.992	1.006	-0.008	0.504
355	pm12-pm26	1.016	1.016	1.017	1.024	0.009	0.478
356	pm12-pm27	1.054	1.088	1.047	1.028	0.011	0.342
357	pm12-pm28	1.024	1.049	0.97	1.016	-0.011	0.366
358	pm12-pm29	1.027	1.035	0.996	1.01	0	0.718
359	pm12-pm30	1.077	1.052	0.992	1.04	0.019	0.341
360	pm12-pm31	1.02	1.101	1.045	1.001	-0.005	0.596
361	pm12-pm32	1.01	1.058	0.982	1.096	0.005	0.659
362	pm12-pm33	1.007	1.153	1.035	1.155	-0.006	0.557
363	pm12-pm34	0.987	1.032	0.927	1.061	-0.019	0.356
364	pm12-pm35	1.04	1.07	0.941	1.147	0.013	0.318
365	pm12-pm36	1.021	1.031	0.986	1.004	0.001	0.674
366	pm12-pm37	0.997	1.004	1.004	1.013	-0.004	0.821
367	pm13-pm14	0.831	0.816	0.834	0842	0.073	0.063
368	pm13-pm15	0.973	1.041	1.009	0.849	-0.054	0.044
369	pm13-pm16	1.047	0.947	1.067	0.877	-0.031	0.288
370	pm13-pm17	0.947	1.03	1.073	0.863	-0.056	0.041
371	pm13-pm18	0.873	0.913	0.922	0.848	0.021	0.229
372	pm13-pm19	0.9	0.888	0.964	0.853	0.004	0.885
373	pm13-pm20	0.888	0.902	0.906	0.851	0.013	0.327
374	pm13-pm21	1.405	0.925	1.654	1.011	-0.039	0.254
375	pm13-pm22	0.875	0.853	0.882	0.816	0.009	0.458
376	pm13-pm23	0.887	0.859	0.88	0.816	0.017	0.381
377	pm13-pm24	0.888	0.889	0.903	0.876	0.026	0.257
378	pm13-pm25	0.879	0.853	0.913	0.864	0.022	0.258
379	pm13-pm26	0.885	0.872	0.904	0.83	0.017	0.345

380	pm13-pm27	0.907	0.89	0.906	0.857	0	0.859
381	pm13-pm28	0.931	0.882	0.895	0.843	0.016	0.384
382	pm13-pm29	0.839	0.89	0.903	0.864	0.011	0.361
383	pm13-pm30	0.881	0.881	0.893	0.859	0.011	0.337
384	pm13-pm31	0.899	0.932	0.962	0.879	0.025	0.232
385	pm13-pm32	0.921	0.855	0.873	0.893	0.007	0.55
386	pm13-pm33	0.935	0.923	0.9	0.927	-0.019	0.368
387	pm13-pm34	0.868	0.926	0.816	0.891	0.007	0.597
388	pm13-pm35	0.881	0.888	0.853	0.948	0.01	0.523
389	pm13-pm36	0.88	0.85	0.888	0.845	0.009	0.586
390	pm13-pm37	0.902	0.859	0.878	0.832	0.013	0.369
391	pm14-pm15	0.994	1.114	1.042	0.915	-0.046	0.188
392	pm14-pm16	1.044	1	1.035	0.922	-0.056	0.02
393	pm14-pm17	1.021	0.993	1.106	0.925	-0.064	0.039
394	pm14-pm18	0.912	0.921	0.908	0.878	-0.003	0.733
395	pm14-pm19	0.891	0.947	0.92	0.908	-0.021	0.292
396	pm14-pm20	0.937	0.908	0.938	0.884	0.002	0.872
397	pm14-pm21	1.43	0.971	1.716	1.101	-0.024	0.255
398	pm14-pm22	0.919	0.911	0.951	0.856	0.022	0.273
399	pm14-pm23	0.895	0.954	0.9	0.89	0.025	0.288
400	pm14-pm24	0.938	0.88	0.923	0.878	0.001	0.798
401	pm14-pm25	0.928	0.849	0.915	0.875	-0.005	0.984
402	pm14-pm26	0.918	0.932	0.912	0.903	0.02	0.345
403	pm14-pm27	0.911	0.968	0.963	0.91	0.008	0.466
404	pm14-pm28	0.927	0.837	0.899	0.889	-0.024	0.295
405	pm14-pm29	0.908	0.892	0.909	0.85	-0.014	0.365
406	pm14-pm30	0.925	0.879	0.903	0.89	-0.009	0.577
407	pm14-pm31	0.946	0.924	0.992	0.926	0.013	0.382
408	pm14-pm32	0.905	0.945	0.958	0.943	0.019	0.322
409	pm14-pm33	0.909	0.968	0.936	0.958	-0.038	0.223

410	pm14-pm34	0.947	0.894	0.869	0.906	-0.004	0.891
411	pm14-pm35	0.902	0.885	0.923	0.952	-0.008	0.582
412	pm14-pm36	0.925	0.858	0.922	0.877	-0.001	0.915
413	pm14-pm37	0.944	0.876	0.905	0.869	0.003	0.657
414	pm15-pm16	1.278	1.381	1.206	1.114	-0.077	0.016
415	pm15-pm17	1.219	1.426	1.287	1.075	-0.088	0.013
416	pm15-pm18	1.16	1.191	1.115	1.042	-0.045	0.171
417	pm15-pm19	1.118	1.262	1.186	1.079	-0.041	0.167
418	pm15-pm20	1.112	1.245	1.135	1.008	-0.054	0.036
419	pm15-pm21	1.694	1.307	2.104	1.292	0.006	0.434
420	pm15-pm22	1.092	1.224	1.098	1.03	-0.041	0.185
421	pm15-pm23	1.065	1.256	1.146	1.006	-0.031	0.213
422	pm15-pm24	1.11	1.255	1.118	1.017	-0.044	0.174
423	pm15-pm25	1.134	1.24	1.128	1.056	-0.021	0.288
424	pm15-pm26	1.133	1.19	1.107	1.062	-0.038	0.21
425	pm15-pm27	1.148	1.298	1.126	1.011	-0.049	0.159
426	pm15-pm28	1.1	1.208	1.124	1.077	-0.05	0.172
427	pm15-pm29	1.101	1.178	1.077	1.057	-0.065	0.035
428	pm15-pm30	1.136	1.251	1.157	1.081	-0.017	0.351
429	pm15-pm31	1.138	1.302	1.155	1.048	-0.038	0.224
430	pm15-pm32	1.097	1.288	1.095	1.091	-0.041	0.174
431	pm15-pm33	1.089	1.389	1.136	1.152	-0.054	0.032
432	pm15-pm34	1.144	1.255	1.038	1.076	-0.044	0.199
433	pm15-pm35	1.112	1.222	1.051	1.154	-0.053	0.042
434	pm15-pm36	1.094	1.212	1.133	1.027	-0.045	0.176
435	pm15-pm37	1.103	1.197	1.118	1.034	-0.047	0.176
436	pm16-pm17	1.502	1.518	1.551	1.361	0.149	0.006
437	pm16-pm18	1.161	1.211	1.169	1.051	-0.018	0.347
438	pm16-pm19	1.162	1.258	1.183	1.112	-0.017	0.371
439	pm16-pm20	1.169	1.173	1.16	1.067	-0.031	0.253

440	pm16-pm21	1.77	1.241	2.145	1.295	0.026	0.276
441	pm16-pm22	1.15	1.237	1.138	1.021	-0.01	0.457
442	pm16-pm23	1.108	1.228	1.143	1.054	-0.01	0.398
443	pm16-pm24	1.121	1.216	1.159	1.079	-0.019	0.359
444	pm16-pm25	1.169	1.125	1.132	1.039	-0.039	0.291
445	pm16-pm26	1.163	1.162	1.141	1.076	-0.019	0.306
446	pm16-pm27	1.145	1.165	1.189	1.05	-0.052	0.038
447	pm16-pm28	1.168	1.184	1.156	1.122	-0.014	0.353
448	pm16-pm29	1.178	1.183	1.176	1.087	-0.007	0.537
449	pm16-pm30	1.158	1.171	1.138	1.044	-0.039	0.236
450	pm16-pm31	1.197	1.285	1.166	1.05	-0.018	0.392
451	pm16-pm32	1.147	1.146	1.169	1.155	-0.023	0.23
452	pm16-pm33	1.22	1.283	1.174	1.228	-0.013	0.365
453	pm16-pm34	1.172	1.249	1.129	1.135	0.004	0.89
454	pm16-pm35	1.162	1.244	1.119	1.224	0.005	0.488
455	pm16-pm36	1.141	1.145	1.159	1.092	-0.022	0.241
456	pm16-pm37	1.169	1.16	1.138	1.055	-0.024	0.264
457	pm17-pm18	1.204	1.153	1.199	1.034	-0.038	0.253
458	pm17-pm19	1.139	1.231	1.244	1.115	-0.032	0.228
459	pm17-pm20	1.151	1.157	1.205	1.067	-0.046	0.197
460	pm17-pm21	1.584	1.351	2.236	1.265	0.004	0.96
461	pm17-pm22	1.177	1.188	1.187	1.008	-0.025	0.259
462	pm17-pm23	1.149	1.245	1.194	0.988	-0.018	0.309
463	pm17-pm24	1.152	1.177	1.188	1.004	-0.051	0.026
464	pm17-pm25	1.151	1.151	1.185	1.027	-0.045	0.158
465	pm17-pm26	1.129	1.184	1.179	1.035	-0.041	0.184
466	pm17-pm27	1.141	1.215	1.252	1.046	-0.044	0.174
467	pm17-pm28	1.181	1.181	1.174	1.055	-0.042	0.171
468	pm17-pm29	1.164	1.172	1.249	1.021	-0.03	0.291
469	pm17-pm30	1.149	1.167	1.206	1.047	-0.043	0.17

470	pm17-pm31	1.166	1.262	1.229	1.076	-0.028	0.298
471	pm17-pm32	1.124	1.157	1.198	1.131	-0.043	0.164
472	pm17-pm33	1.146	1.296	1.215	1.2	-0.044	0.169
473	pm17-pm34	1.145	1.206	1.142	1.091	-0.039	0.252
474	pm17-pm35	1.158	1.193	1.133	1.168	-0.037	0.247
475	pm17-pm36	1.131	1.174	1.217	1.022	-0.038	0.228
476	pm17-pm37	1.159	1.12	1.221	1.079	-0.028	0.282
477	pm18-pm19	1.004	1.012	1.016	1.06	-0.024	0.297
478	pm18-pm20	1.05	1.015	0.991	1.025	-0.004	0.705
479	pm18-pm21	1.534	1.128	1.808	1.251	-0.008	0.473
480	pm18-pm22	1.043	1.038	0.991	1.024	0.026	0.249
481	pm18-pm23	1.014	1.07	0.978	1.043	0.032	0.296
482	pm18-pm24	1.024	1.04	0.995	1.028	0.007	0.564
483	pm18-pm25	1.039	1.007	1.006	1.02	0.012	0.315
484	pm18-pm26	1.013	0.996	1.007	1.044	0.009	0.406
485	pm18-pm27	1.024	1.082	1.001	1.009	-0.011	0.381
486	pm18-pm28	1.045	1.048	0.98	1.022	0.001	0.632
487	pm18-pm29	1.052	1.036	0.993	1.022	0.012	0.342
488	pm18-pm30	1.051	1.043	1.008	1.042	0.017	0.385
489	pm18-pm31	1.029	1.119	1.031	1.044	0.012	0.333
490	pm18-pm32	1.042	1.071	0.985	1.091	0.018	0.352
491	pm18-pm33	1.036	1.137	0.992	1.192	-0.002	0.709
492	pm18-pm34	1.029	1.007	0.894	1.127	-0.004	0.87
493	pm18-pm35	1.066	1.056	0.939	1.166	0.023	0.208
494	pm18-pm36	1.008	1.015	0.982	1.022	0	0.79
495	pm18-pm37	1.033	1.007	0.992	1.024	0.008	0.42
496	pm19-pm20	0.979	1.079	1.013	1.029	-0.028	0.221
497	pm19-pm21	1.544	1.143	1.9	1.237	-0.012	0.389
498	pm19-pm22	1.009	1.019	1.046	1.047	0.003	0.902
499	pm19-pm23	1.001	1.052	0.997	1.05	0.001	0.937

500	pm19-pm24	0.993	1.046	1.059	1.012	-0.016	0.379
501	pm19-pm25	0.987	1.068	1.014	1.03	-0.011	0.307
502	pm19-pm26	0.986	1.083	1.016	1.047	-0.003	0.947
503	pm19-pm27	0.991	1.119	1.055	1.082	-0.008	0.508
504	pm19-pm28	1.019	1.079	1.047	1.092	0.007	0.549
505	pm19-pm29	0.986	1.082	1.019	1.051	-0.009	0.547
506	pm19-pm30	0.989	1.045	1.001	1.07	-0.022	0.246
507	pm19-pm31	1.033	1.132	1.059	1.044	-0.006	0.508
508	pm19-pm32	1.003	1.044	1.041	1.116	-0.007	0.538
509	pm19-pm33	0.983	1.179	1.049	1.171	-0.025	0.293
510	pm19-pm34	0.992	1.051	0.935	1.09	-0.031	0.286
511	pm19-pm35	1.036	1.116	0.968	1.124	-0.001	0.668
512	pm19-pm36	1.013	1.023	1.022	1.062	-0.006	0.482
513	pm19-pm37	1.002	1.081	1.024	0.999	-0.009	0.545
514	pm20-pm21	1.564	1.154	1.846	1.199	-0.004	0.731
515	pm20-pm22	1.032	1.101	1.019	1.006	0.035	0.207
516	pm20-pm23	1.029	1.09	0.988	1.035	0.035	0.276
517	pm20-pm24	1.006	1.055	0.991	1.019	-0.003	0.952
518	pm20-pm25	1.01	1.051	0.995	1.016	0.006	0.503
519	pm20-pm26	1.03	1.075	0.98	0.998	0.008	0.559
520	pm20-pm27	1.021	1.042	1.037	1.019	-0.017	0.39
521	pm20-pm28	1.045	1.072	1.024	1.021	0.012	0.314
522	pm20-pm29	0.978	1.015	0.975	1.016	-0.024	0.222
523	pm20-pm30	1.041	1.069	0.978	1.055	0.011	0.343
524	pm20-pm31	1.016	1.126	1.028	1.039	0.002	0.614
525	pm20-pm32	1.033	1.016	1.012	1.085	0.001	0.795
526	pm20-pm33	1.046	1.102	1.025	1.15	-0.017	0.37
527	pm20-pm34	1.004	1.072	0.934	1.084	-0.001	0.68
528	pm20-pm35	1.019	1.048	0.944	1.117	-0.008	0.488
529	pm20-pm36	1.027	0.986	0.978	1.025	-0.009	0.531

530	pm20-pm37	1.023	1.044	0.996	1.021	0.009	0.581
531	pm21-pm22	1.49	1.126	1.819	1.182	-0.014	0.393
532	pm21-pm23	1.568	1.148	1.849	1.183	0.022	0.251
533	pm21-pm24	1.551	1.147	1.821	1.205	-0.004	0.665
534	pm21-pm25	1.559	1.088	1.819	1.212	-0.007	0.511
535	pm21-pm26	1.506	1.125	1.825	1.22	-0.008	0.436
536	pm21-pm27	1.525	1.199	1.835	1.23	-0.013	0.331
537	pm21-pm28	1.549	1.141	1.837	1.234	-0.003	0.841
538	pm21-pm29	1.557	1.094	1.802	1.184	-0.025	0.295
539	pm21-pm30	1.514	1.092	1.834	1.187	-0.032	0.269
540	pm21-pm31	1.532	1.164	1.817	1.23	-0.029	0.242
541	pm21-pm32	1.501	1.146	1.783	1.249	-0.03	0.226
542	pm21-pm33	1.542	1.201	1.907	1.369	-0.007	0.599
543	pm21-pm34	1.566	1.154	1.765	1.238	-0.008	0.572
544	pm21-pm35	1.524	1.185	1.779	1.363	0.009	0.578
545	pm21-pm36	1.505	1.123	1.832	1.195	-0.014	0.347
546	pm21-pm37	1.497	1.139	1.851	1.2	-0.005	0.835
547	pm22-pm23	0.971	1.05	0.999	1.004	0.032	0.284
548	pm22-pm24	0.991	1.073	0.993	0.971	0.013	0.394
549	pm22-pm25	0.987	1.036	1.017	1.006	0.025	0.291
550	pm22-pm26	1.003	1.018	0.975	0.992	0.011	0.367
551	pm22-pm27	1.027	1.082	1.02	1.031	0.02	0.344
552	pm22-pm28	0.996	1.056	1.032	1.006	0.02	0.244
553	pm22-pm29	0.989	1.1	1.002	0.999	0.028	0.247
554	pm22-pm30	0.996	1.105	0.99	0.976	0.018	0.327
555	pm22-pm31	1.035	1.104	1.025	0.983	0.012	0.363
556	pm22-pm32	0.994	1.072	0.984	1.056	0.018	0.36
557	pm22-pm33	1.014	1.154	0.985	1.161	0.008	0.415
558	pm22-pm34	0.975	1.051	0.925	1.054	0.003	0.993
559	pm22-pm35	0.989	1.081	0.947	1.114	0.02	0.399

560	pm22-pm36	1.002	1.005	0.989	0.972	0.005	0.51
561	pm22-pm37	0.98	1.05	0.976	1.019	0.021	0.218
562	pm23-pm24	1.025	1.027	0.991	1.009	0.022	0.227
563	pm23-pm25	1.001	1.049	0.99	1.013	0.031	0.249
564	pm23-pm26	1.003	1.05	1.005	0.993	0.03	0.235
565	pm23-pm27	1.016	1.163	1.036	0.974	0.031	0.25
566	pm23-pm28	1.022	1.087	1.003	1.017	0.033	0.225
567	pm23-pm29	1.003	1.039	1.004	0.987	0.018	0.332
568	pm23-pm30	1.002	1.052	0.957	1.011	0.01	0.32
569	pm23-pm31	1.033	1.166	1.023	1.014	0.039	0.267
570	pm23-pm32	1.002	1.074	0.973	1.07	0.024	0.254
571	pm23-pm33	1.002	1.161	0.98	1.138	0.003	0.812
572	pm23-pm34	0.95	1.096	0.959	1.038	0.016	0.326
573	pm23-pm35	1.034	1.074	0.9	1.094	0.016	0.354
574	pm23-pm36	0.993	1.077	0.986	0.999	0.03	0.297
575	pm23-pm37	0.998	1.049	1.024	0.997	0.035	0.294
576	pm24-pm25	1.002	1.064	1.012	0.99	0.015	0.39
577	pm24-pm26	1.01	1.043	1.016	1.005	0.016	0.305
578	pm24-pm27	0.976	1.115	1.053	0.994	-0.002	0.713
579	pm24-pm28	1.006	1.025	0.993	1.033	-0.004	0.948
580	pm24-pm29	0.981	1.083	1.005	0.983	0.003	0.98
581	pm24-pm30	1.024	1.04	1.014	1.002	0.005	0.588
582	pm24-pm31	1.019	1.149	1.019	1.025	0.013	0.348
583	pm24-pm32	0.999	1.086	1.002	1.093	0.02	0.399
584	pm24-pm33	1.014	1.148	1.003	1.146	-0.009	0.52
585	pm24-pm34	0.992	1.06	0.906	1.091	-0.002	0.676
586	pm24-pm35	1.022	1.105	0.909	1.096	0.004	0.624
587	pm24-pm36	0.993	1.021	0.991	1.002	-0.001	0.732
588	pm24-pm37	0.999	1.015	0.976	1.008	-0.002	0.939
589	pm25-pm26	0.993	1.001	1.02	1.007	0.011	0.317

590	pm25-pm27	0.997	1.037	1.018	0.986	-0.019	0.36
591	pm25-pm28	1.016	1.042	1.007	1.027	0.013	0.399
592	pm25-pm29	1.009	1.048	0.996	0.991	0.009	0.488
593	pm25-pm30	1.039	1.064	0.998	1.005	0.02	0.214
594	pm25-pm31	1.04	1.11	1.003	1.015	0.01	0.519
595	pm25-pm32	0.997	1.04	0.99	1.082	0.01	0.379
596	pm25-pm33	0.997	1.126	1.007	1.119	-0.017	0.356
597	pm25-pm34	0.992	1.034	0.921	1.054	-0.006	0.457
598	pm25-pm35	1.032	1.097	0.936	1.151	0.033	0.278
599	pm25-pm36	1.005	1.023	0.991	0.987	0.006	0.469
600	pm25-pm37	1.018	0.982	0.998	0.988	0.002	0.663
601	pm26-pm27	0.998	1.031	1.04	0.99	-0.013	0.387
602	pm26-pm28	0.996	0.965	1.01	1.04	-0.008	0.401
603	pm26-pm29	0.991	1.075	1.015	1.008	0.02	0.304
604	pm26-pm30	1.039	0.999	1.004	1.021	0.009	0.418
605	pm26-pm31	1.046	1.099	1.031	0.994	0.01	0.467
606	pm26-pm32	1.006	1.005	0.982	1.09	0.003	0.761
607	pm26-pm33	1.048	1.125	0.988	1.148	-0.002	0.774
608	pm26-pm34	1.01	1.036	0.922	1.065	0.002	0.774
609	pm26-pm35	1.013	1.005	0.929	1.124	-0.004	0.761
610	pm26-pm36	0.978	1.006	0.988	1.008	0	0.882
611	pm26-pm37	1	1.001	1.015	0.991	0.008	0.495
612	pm27-pm28	1.03	1.081	1.034	0.996	-0.01	0.55
613	pm27-pm29	0.99	1.111	1.008	1.031	-0.001	0.628
614	pm27-pm30	1.003	1.061	1.013	1.017	-0.017	0.313
615	pm27-pm31	1.056	1.166	1.084	1.03	0.018	0.389
616	pm27-pm32	1.01	1.118	1.015	1.096	0.009	0.497
617	pm27-pm33	1.023	1.162	1.043	1.185	-0.01	0.48
618	pm27-pm34	1.006	1.102	0.974	1.068	-0.003	0.902
619	pm27-pm35	1.031	1.094	0.989	1.108	0	0.616

620	pm27-pm36	0.993	1.034	1.046	1.012	-0.008	0.575
621	pm27-pm37	0.99	1.047	1.039	1.017	-0.005	0.652
622	pm28-pm29	1.025	1.034	1.005	1.05	0.01	0.568
623	pm28-pm30	1.019	1.057	0.997	1.062	0.011	0.397
624	pm28-pm31	1.054	1.112	1.028	1.031	0.007	0.513
625	pm28-pm32	1.004	1.061	0.989	1.109	0.008	0.498
626	pm28-pm33	1.02	1.174	0.96	1.178	-0.013	0.395
627	pm28-pm34	1	1.042	0.916	1.043	-0.022	0.285
628	pm28-pm35	1.023	1.072	0.959	1.138	0.01	0.358
629	pm28-pm36	1.022	1.044	0.997	1.016	0.008	0.482
630	pm28-pm37	1.025	1.025	0.99	1.036	0.009	0.597
631	pm29-pm30	1.006	1.033	0.99	1.027	-0.001	0.899
632	pm29-pm31	1.034	1.153	1.041	1.026	0.023	0.234
633	pm29-pm32	1.012	1.02	0.985	1.066	-0.004	0.773
634	pm29-pm33	1.012	1.154	1.014	1.188	0.005	0.899
635	pm29-pm34	0.971	1.103	0.907	1.073	-0.001	0.843
636	pm29-pm35	1.028	1.057	0.922	1.144	0.008	0.505
637	pm29-pm36	1.01	1.028	1.011	0.984	0.005	0.444
638	pm29-pm37	1.005	1.053	0.989	0.988	0.007	0.507
639	pm30-pm31	1.068	1.107	1.037	1.005	0.01	0.433
640	pm30-pm32	1.03	1.068	0.986	1.077	0.011	0.335
641	pm30-pm33	1.063	1.134	1.009	1.167	0.002	0.82
642	pm30-pm34	0.977	1.021	0.91	1.078	-0.022	0.289
643	pm30-pm35	1.041	1.045	0.947	1.181	0.02	0.325
644	pm30-pm36	1.039	1.033	1.027	0.993	0.015	0.385
645	pm30-pm37	1.006	1.004	1.02	1.031	0.009	0.426
646	pm31-pm32	1.013	1.175	0.999	1.079	0.012	0.374
647	pm31-pm33	1.041	1.189	1.03	1.158	-0.013	0.388
648	pm31-pm34	1.032	1.162	0.979	1.078	0.018	0.358
649	pm31-pm35	1.039	1.167	0.956	1.166	0.023	0.207

650	pm31-pm36	1.033	1.075	1.02	1.03	0.006	0.542
651	pm31-pm37	1.038	1.132	1.031	1.008	0.02	0.28
652	pm32-pm33	1.051	1.151	0.981	1.225	0	0.875
653	pm32-pm34	1.006	1.082	0.931	1.14	0.011	0.302
654	pm32-pm35	1.061	1.119	0.933	1.186	0.031	0.241
655	pm32-pm36	0.996	1.044	0.989	1.064	0.006	0.562
656	pm32-pm37	0.986	1.056	0.995	1.08	0.012	0.375
657	pm33-pm34	1.047	1.114	0.959	1.241	-0.001	0.614
658	pm33-pm35	1.015	1.169	0.904	1.268	-0.017	0.375
659	pm33-pm36	0.973	1.115	1	1.171	-0.015	0.35
660	pm33-pm37	1.012	1.156	0.973	1.166	-0.002	0.905
661	pm34-pm35	1.004	1.028	0.94	1.146	-0.004	0.982
662	pm34-pm36	0.984	1.031	0.951	1.067	0.001	0.954
663	pm34-pm37	1.016	1.051	0.938	1.031	0.003	0.915
664	pm35-pm36	1.014	1.099	0.934	1.081	0.01	0.489
665	pm35-pm37	1.031	1.052	0.901	1.133	0.008	0.536
666	pm36-pm37	0.987	1	0.999	1.004	0.002	0.797

3c) Result of two-distribution perturbation analysis for hypertrophy when each response function was separately applied (100 million parameter sets)

No.	Pair of perturbed parameter distributions	Lin	Hill	Sat	Acc	Synergistic effect	p-value
1	pm1-pm2	1.01	1.01	1.003	0.981	-0.012	0.334
2	pm1-pm3	1.036	1.044	0.984	1.019	-0.004	0.895
3	pm1-pm4	0.997	1.082	1.006	1.01	-0.01	0.342
4	pm1-pm5	1.009	1.025	1.028	0.985	-0.004	0.883
5	pm1-pm6	1.001	1.001	0.998	1.009	-0.008	0.484

6	pm1-pm7	2.814	2.048	2.691	2.244	-0.111	0.006
7	pm1-pm8	1.053	1.024	1.025	1.073	0.012	0.328
8	pm1-pm9	1.071	1.068	1.013	1.054	-0.001	0.963
9	pm1-pm10	1.456	1.542	1.267	1.452	-0.042	0.186
10	pm1-pm11	1.014	0.98	0.99	0.997	-0.015	0.389
11	pm1-pm12	1.005	1.061	0.985	1.016	-0.008	0.405
12	pm1-pm13	0.924	0.906	0.893	0.836	0.018	0.343
13	pm1-pm14	0.942	0.908	0.966	0.883	0.012	0.35
14	pm1-pm15	1.083	1.225	1.121	0.987	-0.073	0.012
15	pm1-pm16	1.202	1.205	1.187	1.039	-0.013	0.336
16	pm1-pm17	1.189	1.214	1.183	1.063	-0.027	0.245
17	pm1-pm18	1.044	1.003	1.002	1.002	-0.01	0.306
18	pm1-pm19	0.989	1.077	1.048	1.022	-0.018	0.341
19	pm1-pm20	1.015	1.074	1.011	1.024	0.002	0.701
20	pm1-pm21	1.532	1.1	1.834	1.229	-0.019	0.388
21	pm1-pm22	1.016	1.041	1.031	0.955	0.008	0.522
22	pm1-pm23	1.012	1.047	1.038	0.978	0.019	0.306
23	pm1-pm24	1.021	1.068	0.995	0.99	0	0.644
24	pm1-pm25	1.008	1.022	1.003	1.012	0	0.782
25	pm1-pm26	0.988	1.028	0.987	1.01	-0.007	0.592
26	pm1-pm27	1.008	1.056	1.038	0.997	-0.02	0.229
27	pm1-pm28	1.028	1.042	1.035	1.02	0.004	0.824
28	pm1-pm29	1.035	1.059	1.003	1.007	0.007	0.525
29	pm1-pm30	1.062	1.01	1.017	0.994	-0.002	0.934
30	pm1-pm31	1.02	1.103	1.062	0.995	-0.004	0.634
31	pm1-pm32	1.027	1.04	1.029	1.055	0.004	0.975
32	pm1-pm33	1.02	1.144	1	1.152	-0.016	0.394
33	pm1-pm34	0.977	1.058	0.961	1.097	0	0.87
34	pm1-pm35	1.014	1.072	0.972	1.083	-0.003	0.603
35	pm1-pm36	1.014	1.02	1.022	0.979	-0.003	0.868

36	pm1-pm37	1.014	1.025	1.021	0.989	0.002	0.845
37	pm2-pm3	0.983	0.972	0.997	1.036	-0.014	0.304
38	pm2-pm4	0.963	1.077	1.01	1.023	-0.002	0.61
39	pm2-pm5	1.018	0.978	1.024	1.019	0.008	0.545
40	pm2-pm6	1.025	1.004	0.987	0.995	0.006	0.566
41	pm2-pm7	2.865	1.992	2.614	2.238	-0.119	0.008
42	pm2-pm8	0.997	0.991	0.998	1.031	-0.014	0.336
43	pm2-pm9	1.035	1.082	1.02	1.047	0.008	0.504
44	pm2-pm10	1.432	1.495	1.296	1.495	-0.028	0.294
45	pm2-pm11	0.982	0.994	0.976	1.007	-0.006	0.552
46	pm2-pm12	0.986	1.011	0.982	0.972	-0.024	0.293
47	pm2-pm13	0.881	0.834	0.905	0.871	0.015	0.396
48	pm2-pm14	0.879	0.86	0.933	0.862	-0.015	0.315
49	pm2-pm15	1.07	1.193	1.114	1.032	-0.061	0.026
50	pm2-pm16	1.14	1.151	1.174	1.053	-0.028	0.203
51	pm2-pm17	1.148	1.171	1.19	1.032	-0.04	0.158
52	pm2-pm18	1.025	1.015	1.015	1.008	0.007	0.496
53	pm2-pm19	0.981	1.053	1.01	1.026	-0.021	0.219
54	pm2-pm20	1.023	1.027	1.006	1.027	0.006	0.536
55	pm2-pm21	1.548	1.106	1.862	1.235	0.009	0.51
56	pm2-pm22	1.016	1.048	1.017	0.977	0.026	0.29
57	pm2-pm23	0.995	1.071	1.004	0.999	0.032	0.291
58	pm2-pm24	1.003	1.016	1.015	0.995	0.002	0.897
59	pm2-pm25	1.02	1.022	0.991	0.998	0.011	0.385
60	pm2-pm26	1.02	0.975	1.018	1.011	0.009	0.437
61	pm2-pm27	1.004	1.069	1.034	0.989	-0.007	0.441
62	pm2-pm28	1.019	1.036	0.998	1.017	0.004	0.668
63	pm2-pm29	1.014	1.013	0.997	1.008	0.003	0.793
64	pm2-pm30	1.039	1.032	1.022	1.024	0.02	0.234
65	pm2-pm31	1.052	1.088	1.044	1.02	0.016	0.352

66	pm2-pm32	1.014	1.032	1.002	1.09	0.015	0.399
67	pm2-pm33	1.01	1.156	1	1.147	-0.004	0.814
68	pm2-pm34	1	1.026	0.97	1.089	0.012	0.37
69	pm2-pm35	1.025	1.015	0.937	1.143	0.006	0.495
70	pm2-pm36	0.988	1.035	1.007	0.991	0.008	0.407
71	pm2-pm37	1.013	1.001	0.982	0.999	0.002	0.715
72	pm3-pm4	1.029	1.098	1.017	1.014	0.008	0.415
73	pm3-pm5	0.998	0.997	0.997	1.045	-0.004	0.748
74	pm3-pm6	1.013	0.976	1.009	1.029	-0.001	0.988
75	pm3-pm7	2.876	1.964	2.669	2.258	-0.115	0.008
76	pm3-pm8	1.002	1.042	0.983	1.068	-0.005	0.43
77	pm3-pm9	1.043	1.055	1.035	1.048	-0.004	0.981
78	pm3-pm10	1.471	1.517	1.252	1.565	-0.018	0.335
79	pm3-pm11	1.017	1.03	1.007	1.02	0.011	0.388
80	pm3-pm12	1.028	1.03	1.018	1.011	-0.001	0.788
81	pm3-pm13	0.932	0.899	0.894	0.851	0.024	0.212
82	pm3-pm14	0.915	0.961	0.912	0.889	0.01	0.523
83	pm3-pm15	1.108	1.217	1.096	1.071	-0.052	0.039
84	pm3-pm16	1.164	1.156	1.149	1.111	-0.024	0.26
85	pm3-pm17	1.175	1.199	1.14	1.067	-0.042	0.165
86	pm3-pm18	1.022	1.025	0.973	1.022	-0.009	0.534
87	pm3-pm19	0.98	1.048	1.041	1.049	-0.02	0.333
88	pm3-pm20	1.022	0.988	1.009	1.03	-0.014	0.305
89	pm3-pm21	1.53	1.16	1.827	1.194	-0.013	0.37
90	pm3-pm22	0.998	1.059	0.978	0.992	0.007	0.555
91	pm3-pm23	1.022	1.089	1.004	1.029	0.04	0.241
92	pm3-pm24	0.998	1.049	1.009	1.033	0.006	0.513
93	pm3-pm25	1.013	0.972	1.001	1.023	-0.006	0.584
94	pm3-pm26	1.025	1.01	1.009	1.039	0.013	0.381
95	pm3-pm27	1.023	1.075	1.032	1.03	-0.002	0.782

96	pm3-pm28	1.024	1.03	0.97	1.047	-0.007	0.54
97	pm3-pm29	1.003	1.065	1.013	1.048	0.016	0.307
98	pm3-pm30	1.016	1.055	0.992	1.022	0.001	0.92
99	pm3-pm31	1.057	1.06	1.035	1.033	0	0.94
100	pm3-pm32	0.987	1.03	0.987	1.118	0	0.982
101	pm3-pm33	1.015	1.086	1.021	1.197	-0.013	0.335
102	pm3-pm34	1.029	1.025	0.941	1.087	0.001	0.88
103	pm3-pm35	1.043	1.022	0.934	1.118	-0.006	0.599
104	pm3-pm36	0.986	1.018	0.99	1.017	-0.006	0.541
105	pm3-pm37	1.002	1.031	0.98	1.013	-0.001	0.981
106	pm4-pm5	1.044	1.119	0.983	1.036	0.023	0.27
107	pm4-pm6	1.013	1.082	0.994	1.009	0.007	0.45
108	pm4-pm7	2.82	2.111	2.629	2.299	-0.102	0.006
109	pm4-pm8	1.016	1.095	1.006	1.056	0.004	0.621
110	pm4-pm9	1.05	1.152	1.029	1.051	0.011	0.355
111	pm4-pm10	1.474	1.551	1.267	1.522	-0.025	0.272
112	pm4-pm11	1.027	1.049	1.014	1.023	0.011	0.314
113	pm4-pm12	1.012	1.101	0.997	1.028	0.002	0.958
114	pm4-pm13	0.87	0.953	0.922	0.891	0.03	0.282
115	pm4-pm14	0.938	0.903	0.95	0.875	-0.003	0.917
116	pm4-pm15	1.1	1.298	1.116	1.024	-0.05	0.15
117	pm4-pm16	1.147	1.264	1.158	1.099	-0.011	0.329
118	pm4-pm17	1.163	1.238	1.221	1.058	-0.026	0.248
119	pm4-pm18	1.015	1.108	1.028	1.025	0.014	0.357
120	pm4-pm19	0.973	1.122	1.011	1.053	-0.019	0.312
121	pm4-pm20	1.025	1.094	0.991	1.04	0.002	0.892
122	pm4-pm21	1.503	1.19	1.805	1.205	-0.024	0.281
123	pm4-pm22	1.035	1.143	0.997	1.017	0.038	0.231
124	pm4-pm23	1.009	1.158	1.001	1.012	0.039	0.252
125	pm4-pm24	0.995	1.121	1.015	1.017	0.011	0.31

126	pm4-pm25	1.012	1.089	1.007	1.003	0.01	0.515
127	pm4-pm26	0.984	1.107	0.996	1.004	0.005	0.776
128	pm4-pm27	1.01	1.147	1.038	1.025	0.003	0.798
129	pm4-pm28	1.033	1.111	0.996	1.047	0.013	0.377
130	pm4-pm29	0.984	1.174	1.002	1.037	0.024	0.232
131	pm4-pm30	1.047	1.079	1.024	1.061	0.023	0.291
132	pm4-pm31	1.035	1.205	1.04	1.026	0.021	0.207
133	pm4-pm32	0.997	1.091	1.006	1.039	-0.007	0.492
134	pm4-pm33	1.03	1.161	0.972	1.179	-0.017	0.355
135	pm4-pm34	1.009	1.153	0.93	1.086	0.015	0.359
136	pm4-pm35	1.028	1.118	0.936	1.116	0.005	0.909
137	pm4-pm36	1.014	1.065	1.006	1.001	0.003	0.875
138	pm4-pm37	1.016	1.083	1.014	1.015	0.015	0.398
139	pm5-pm6	0.987	1.011	0.999	0.989	-0.002	0.68
140	pm5-pm7	2.848	1.983	2.677	2.281	-0.101	0.009
141	pm5-pm8	1.027	0.993	1.007	1.079	0.006	0.598
142	pm5-pm9	1.069	1.077	1.012	1.017	0.003	0.787
143	pm5-pm10	1.424	1.475	1.238	1.496	-0.052	0.021
144	pm5-pm11	1.011	1.01	0.989	1.012	0.007	0.595
145	pm5-pm12	1.035	1.024	1.017	1.046	0.017	0.352
146	pm5-pm13	0.899	0.824	0.909	0.831	0.005	0.501
147	pm5-pm14	0.944	0.898	0.948	0.915	0.025	0.232
148	pm5-pm15	1.138	1.15	1.13	1.003	-0.06	0.039
149	pm5-pm16	1.149	1.163	1.119	1.068	-0.035	0.203
150	pm5-pm17	1.187	1.169	1.235	1.032	-0.022	0.218
151	pm5-pm18	1.032	1.011	1.013	1.025	0.009	0.536
152	pm5-pm19	1.009	1.001	1.018	1.045	-0.022	0.298
153	pm5-pm20	1.034	1.013	1.019	1.029	0.007	0.465
154	pm5-pm21	1.547	1.13	1.84	1.232	0.006	0.532
155	pm5-pm22	1.009	1.004	0.999	1.003	0.013	0.381

156	pm5-pm23	1.005	1.062	1.012	1.028	0.039	0.208
157	pm5-pm24	0.994	1.046	0.993	1.009	0.004	0.97
158	pm5-pm25	1.005	0.983	1	1.006	-0.001	0.978
159	pm5-pm26	0.99	1.003	1.02	1.028	0.011	0.369
160	pm5-pm27	0.996	1.074	1.043	1.015	-0.001	0.909
161	pm5-pm28	1.022	1.013	1.003	1.037	0.003	0.831
162	pm5-pm29	1.012	1.034	1.004	1.049	0.018	0.359
163	pm5-pm30	1.053	1.024	1.009	1	0.01	0.374
164	pm5-pm31	1.044	1.087	1.019	1.015	0.004	0.777
165	pm5-pm32	0.999	1.01	0.999	1.1	0.005	0.507
166	pm5-pm33	1.013	1.154	0.978	1.175	-0.004	0.701
167	pm5-pm34	1	1.025	0.925	1.086	-0.002	0.685
168	pm5-pm35	1.017	1.039	0.923	1.132	0.002	0.8
169	pm5-pm36	1.014	1.005	1.02	0.995	0.008	0.44
170	pm5-pm37	1.007	0.988	0.997	1.03	0.007	0.486
171	pm6-pm7	2.809	2.011	2.659	2.239	-0.114	0.01
172	pm6-pm8	1.033	1.012	0.992	1.038	0.004	0.91
173	pm6-pm9	1.066	1.085	1.024	1.024	0.014	0.397
174	pm6-pm10	1.43	1.45	1.255	1.527	-0.039	0.271
175	pm6-pm11	1.008	0.993	1.004	1.017	0.012	0.327
176	pm6-pm12	1.025	1.023	0.987	1.026	0.006	0.54
177	pm6-pm13	0.875	0.841	0.913	0.84	0.012	0.389
178	pm6-pm14	0.918	0.877	0.919	0.855	-0.003	0.72
179	pm6-pm15	1.108	1.216	1.134	1.03	-0.038	0.235
180	pm6-pm16	1.183	1.176	1.185	1.046	-0.007	0.58
181	pm6-pm17	1.153	1.177	1.194	1.021	-0.036	0.271
182	pm6-pm18	1.024	1.009	0.986	1.015	0.003	0.787
183	pm6-pm19	1.004	1.102	1.027	1.021	0.003	0.965
184	pm6-pm20	1.033	1.043	0.986	1.004	0.004	0.85
185	pm6-pm21	1.526	1.176	1.884	1.239	0.03	0.238

186	pm6-pm22	1.003	1.054	0.979	0.971	0.016	0.37
187	pm6-pm23	1.014	1.062	0.98	0.984	0.028	0.293
188	pm6-pm24	0.985	1.004	1.008	0.988	-0.006	0.581
189	pm6-pm25	0.986	0.985	1.008	1.015	0.005	0.635
190	pm6-pm26	0.98	0.995	1.015	0.998	0.003	0.826
191	pm6-pm27	1.016	1.064	1.035	1	0.001	0.804
192	pm6-pm28	0.997	0.982	1.014	1.016	-0.008	0.518
193	pm6-pm29	0.984	1.031	0.985	1.001	-0.001	0.7
194	pm6-pm30	1.008	1.03	0.994	1.023	0.008	0.468
195	pm6-pm31	1.036	1.077	1.009	1.003	0	0.885
196	pm6-pm32	1.004	1.023	1.001	1.082	0.011	0.322
197	pm6-pm33	0.998	1.102	0.974	1.185	-0.014	0.343
198	pm6-pm34	0.988	1.022	0.92	1.088	-0.001	0.883
199	pm6-pm35	1.022	1.059	0.966	1.118	0.02	0.201
200	pm6-pm36	0.981	0.994	0.987	1.015	-0.001	0.81
201	pm6-pm37	0.992	0.971	0.996	1.006	-0.002	0.782
202	pm7-pm8	2.876	2.031	2.635	2.288	-0.107	0.007
203	pm7-pm9	2.954	2.149	2.695	2.264	-0.07	0.039
204	pm7-pm10	3.863	3.026	3.279	3.287	0.359	<0.001
205	pm7-pm11	2.85	2.009	2.607	2.266	-0.11	0.006
206	pm7-pm12	2.801	2.003	2.621	2.288	-0.13	0.008
207	pm7-pm13	2.905	2.125	2.823	2.314	0.137	0.006
208	pm7-pm14	2.996	2.173	2.892	2.408	0.172	0.002
209	pm7-pm15	3.055	2.412	2.894	2.345	-0.033	0.295
210	pm7-pm16	3.193	2.313	3.076	2.412	0.045	0.19
211	pm7-pm17	3.196	2.305	3.134	2.356	0.025	0.268
212	pm7-pm18	2.813	2.043	2.632	2.286	-0.112	0.006
213	pm7-pm19	2.818	2.035	2.715	2.257	-0.129	0.006
214	pm7-pm20	2.887	2.023	2.665	2.272	-0.1	0.008
215	pm7-pm21	4.075	2.224	4.54	2.731	0.417	<0.001

216	pm7-pm22	2.752	2.065	2.663	2.289	-0.093	0.011
217	pm7-pm23	2.826	2.102	2.643	2.255	-0.076	0.016
218	pm7-pm24	2.873	2.011	2.63	2.23	-0.115	0.008
219	pm7-pm25	2.796	2.057	2.626	2.237	-0.115	0.005
220	pm7-pm26	2.736	1.991	2.619	2.267	-0.141	0.006
221	pm7-pm27	2.829	2.072	2.689	2.234	-0.122	0.01
222	pm7-pm28	2.863	2.053	2.659	2.292	-0.093	0.01
223	pm7-pm29	2.875	1.982	2.639	2.274	-0.109	0.005
224	pm7-pm30	2.822	2.035	2.634	2.273	-0.115	0.005
225	pm7-pm31	2.87	2.16	2.704	2.302	-0.072	0.017
226	pm7-pm32	2.84	2.044	2.626	2.359	-0.099	0.017
227	pm7-pm33	2.785	2.239	2.734	2.489	-0.067	0.042
228	pm7-pm34	2.877	2.055	2.533	2.326	-0.108	0.006
229	pm7-pm35	2.912	2.104	2.53	2.483	-0.063	0.043
230	pm7-pm36	2.832	1.91	2.682	2.254	-0.125	0.009
231	pm7-pm37	2.808	1.98	2.683	2.203	-0.125	0.005
232	pm8-pm9	1.06	1.085	1.028	1.1	0.011	0.309
233	pm8-pm10	1.461	1.482	1.264	1.543	-0.039	0.212
234	pm8-pm11	1.018	0.99	1.021	1.04	0.002	0.684
235	pm8-pm12	1.025	1.037	0.987	1.015	-0.014	0.302
236	pm8-pm13	0.917	0.899	0.93	0.883	0.031	0.298
237	pm8-pm14	0.916	0.853	0.942	0.923	-0.009	0.43
238	pm8-pm15	1.102	1.164	1.089	1.1	-0.068	0.027
239	pm8-pm16	1.167	1.16	1.16	1.129	-0.022	0.3
240	pm8-pm17	1.197	1.203	1.203	1.093	-0.02	0.228
241	pm8-pm18	1.026	1.033	1.01	1.054	0.003	0.956
242	pm8-pm19	0.989	1.074	1.036	1.1	-0.007	0.422
243	pm8-pm20	1.05	1.042	1	1.039	-0.001	0.966
244	pm8-pm21	1.544	1.092	1.837	1.251	-0.017	0.392
245	pm8-pm22	1.049	1.073	1.004	1.036	0.033	0.273

246	pm8-pm23	1.004	1.071	0.981	1.019	0.015	0.349
247	pm8-pm24	1.004	1.028	0.993	1.03	-0.01	0.532
248	pm8-pm25	1.031	1.022	0.993	1.049	0.009	0.565
249	pm8-pm26	1.005	1.005	1.01	1.054	0.003	0.972
250	pm8-pm27	1.042	1.069	1	1.046	-0.01	0.307
251	pm8-pm28	1.007	1.031	0.994	1.082	-0.003	0.745
252	pm8-pm29	1.02	1.098	1.018	1.054	0.024	0.203
253	pm8-pm30	1.057	1.007	1.015	1.043	0.003	0.851
254	pm8-pm31	1.054	1.104	1.056	1.048	0.012	0.314
255	pm8-pm32	1.008	1.06	1.005	1.136	0.014	0.397
256	pm8-pm33	0.994	1.144	0.997	1.218	-0.012	0.398
257	pm8-pm34	1.002	1	0.919	1.113	-0.019	0.355
258	pm8-pm35	1.008	1.044	0.966	1.178	0.007	0.54
259	pm8-pm36	1.011	1.014	1.007	1.047	0.003	0.84
260	pm8-pm37	1.013	1.003	1.015	1.075	0.011	0.386
261	pm9-pm10	1.459	1.552	1.289	1.528	-0.04	0.234
262	pm9-pm11	1.087	1.063	1.007	1.047	0.016	0.344
263	pm9-pm12	1.059	1.118	1.05	1.032	0.014	0.35
264	pm9-pm13	0.937	0.918	0.95	0.904	0.03	0.297
265	pm9-pm14	0.975	0.921	0.918	0.922	-0.004	0.653
266	pm9-pm15	1.168	1.293	1.167	1.064	-0.029	0.275
267	pm9-pm16	1.224	1.248	1.193	1.08	-0.01	0.325
268	pm9-pm17	1.222	1.227	1.244	1.104	-0.016	0.354
269	pm9-pm18	1.058	1.111	1.014	1.044	0.009	0.53
270	pm9-pm19	1.069	1.094	1.035	1.09	-0.005	0.414
271	pm9-pm20	1.067	1.079	1.015	1.038	-0.004	0.77
272	pm9-pm21	1.633	1.18	1.869	1.285	0.024	0.284
273	pm9-pm22	1.06	1.138	1.04	1.027	0.039	0.224
274	pm9-pm23	1.075	1.121	1.005	1.031	0.034	0.287
275	pm9-pm24	1.074	1.139	1.03	1.036	0.026	0.215

276	pm9-pm25	1.084	1.083	1.015	1.022	0.015	0.322
277	pm9-pm26	1.059	1.063	1.023	1.058	0.015	0.331
278	pm9-pm27	1.091	1.167	1.051	1.074	0.026	0.279
279	pm9-pm28	1.067	1.119	1.03	1.035	0.01	0.353
280	pm9-pm29	1.043	1.14	1.037	1.032	0.019	0.4
281	pm9-pm30	1.099	1.097	1.037	1.048	0.022	0.249
282	pm9-pm31	1.058	1.169	1.042	1.038	0.003	0.799
283	pm9-pm32	1.036	1.12	1.005	1.102	0.007	0.554
284	pm9-pm33	1.037	1.152	1	1.176	-0.029	0.249
285	pm9-pm34	1.028	1.11	0.997	1.092	0.009	0.576
286	pm9-pm35	1.053	1.153	0.997	1.124	0.019	0.319
287	pm9-pm36	1.077	1.076	1.031	1.015	0.013	0.346
288	pm9-pm37	1.057	1.081	1.025	1.002	0.006	0.549
289	pm10-pm11	1.445	1.546	1.255	1.472	-0.025	0.226
290	pm10-pm12	1.457	1.576	1.29	1.518	-0.009	0.572
291	pm10-pm13	1.283	1.298	1.199	1.25	-0.059	0.023
292	pm10-pm14	1.513	1.524	1.419	1.498	0.131	0.007
293	pm10-pm15	1.587	1.833	1.382	1.556	-0.032	0.211
294	pm10-pm16	1.577	1.704	1.422	1.563	-0.049	0.154
295	pm10-pm17	1.656	1.718	1.48	1.518	-0.041	0.188
296	pm10-pm18	1.43	1.511	1.262	1.538	-0.032	0.247
297	pm10-pm19	1.427	1.609	1.325	1.546	-0.02	0.347
298	pm10-pm20	1.448	1.547	1.274	1.451	-0.043	0.18
299	pm10-pm21	2.018	1.598	2.144	1.705	-0.021	0.243
300	pm10-pm22	1.369	1.618	1.28	1.454	-0.017	0.307
301	pm10-pm23	1.406	1.509	1.256	1.463	-0.035	0.278
302	pm10-pm24	1.417	1.511	1.29	1.498	-0.034	0.281
303	pm10-pm25	1.431	1.502	1.255	1.487	-0.037	0.294
304	pm10-pm26	1.394	1.493	1.234	1.435	-0.066	0.04
305	pm10-pm27	1.429	1.585	1.303	1.522	-0.029	0.209

306	pm10-pm28	1.453	1.56	1.288	1.549	-0.009	0.446
307	pm10-pm29	1.423	1.55	1.287	1.444	-0.037	0.236
308	pm10-pm30	1.399	1.547	1.233	1.528	-0.041	0.173
309	pm10-pm31	1.462	1.637	1.294	1.515	-0.016	0.376
310	pm10-pm32	1.414	1.562	1.233	1.613	-0.022	0.206
311	pm10-pm33	1.459	1.614	1.251	1.705	-0.033	0.284
312	pm10-pm34	1.424	1.544	1.201	1.579	-0.03	0.229
313	pm10-pm35	1.433	1.533	1.186	1.595	-0.045	0.194
314	pm10-pm36	1.385	1.462	1.259	1.5	-0.055	0.04
315	pm10-pm37	1.42	1.462	1.287	1.491	-0.04	0.246
316	pm11-pm12	0.999	1.024	1.019	1.01	0.005	0.699
317	pm11-pm13	0.899	0.864	0.897	0.829	0.017	0.314
318	pm11-pm14	0.912	0.848	0.909	0.895	-0.005	0.851
319	pm11-pm15	1.094	1.203	1.113	1.027	-0.051	0.025
320	pm11-pm16	1.187	1.142	1.153	1.071	-0.016	0.318
321	pm11-pm17	1.162	1.183	1.221	1.031	-0.023	0.291
322	pm11-pm18	1.028	0.991	0.979	1.022	-0.001	0.883
323	pm11-pm19	1.028	1.023	1.011	1.054	-0.006	0.599
324	pm11-pm20	1.023	1.031	0.987	1.005	0	0.89
325	pm11-pm21	1.529	1.104	1.811	1.199	-0.015	0.314
326	pm11-pm22	1	1.031	1.001	0.999	0.022	0.28
327	pm11-pm23	0.994	1.076	1.009	1.009	0.04	0.192
328	pm11-pm24	0.981	1.026	1.006	0.987	-0.002	0.854
329	pm11-pm25	1.001	0.983	1.015	1.01	0.009	0.484
330	pm11-pm26	1.017	1.012	1.015	0.998	0.017	0.361
331	pm11-pm27	1.002	1.036	1.005	1.02	-0.012	0.393
332	pm11-pm28	0.995	1.057	1.005	1.003	0.005	0.699
333	pm11-pm29	0.973	1.041	1.012	0.972	-0.002	0.666
334	pm11-pm30	1.013	1.028	1.007	1.011	0.009	0.526
335	pm11-pm31	1.026	1.062	1.04	0.995	-0.001	0.931

336	pm11-pm32	0.986	1.05	0.977	1.067	0.003	0.732
337	pm11-pm33	1.031	1.117	0.994	1.206	0.008	0.486
338	pm11-pm34	0.957	1.038	0.92	1.042	-0.016	0.311
339	pm11-pm35	1.007	1.056	0.941	1.123	0.011	0.338
340	pm11-pm36	0.984	1.015	0.98	0.99	-0.002	0.701
341	pm11-pm37	1.017	0.993	0.99	1.016	0.011	0.35
342	pm12-pm13	0.879	0.899	0.917	0.803	0.004	0.638
343	pm12-pm14	0.949	0.91	0.934	0.865	0.004	0.676
344	pm12-pm15	1.106	1.211	1.115	1.052	-0.054	0.028
345	pm12-pm16	1.167	1.217	1.147	1.081	-0.016	0.373
346	pm12-pm17	1.164	1.12	1.186	1.056	-0.056	0.043
347	pm12-pm18	1.056	1.025	0.989	1.013	0	0.978
348	pm12-pm19	1.006	1.056	1.001	1.034	-0.026	0.218
349	pm12-pm20	1.046	1.017	1	0.989	-0.014	0.342
350	pm12-pm21	1.529	1.142	1.815	1.236	-0.011	0.314
351	pm12-pm22	1.006	1.052	1.012	0.992	0.015	0.368
352	pm12-pm23	1	1.077	1.022	1.004	0.029	0.292
353	pm12-pm24	0.999	1.034	1.02	1.013	0	0.996
354	pm12-pm25	1.005	1.013	0.989	1.014	-0.004	0.741
355	pm12-pm26	1.003	1.022	1.014	1.012	0.004	0.75
356	pm12-pm27	1.046	1.109	1.062	1.028	0.018	0.356
357	pm12-pm28	1.019	1.03	0.993	1.027	-0.008	0.453
358	pm12-pm29	0.994	1.032	1.013	1.023	-0.002	0.988
359	pm12-pm30	1.076	1.025	1.021	1.043	0.02	0.368
360	pm12-pm31	1.023	1.112	1.03	0.992	-0.007	0.505
361	pm12-pm32	1.033	1.037	0.978	1.1	0.005	0.439
362	pm12-pm33	1.012	1.181	1.026	1.177	0.005	0.561
363	pm12-pm34	0.989	1.031	0.921	1.025	-0.029	0.257
364	pm12-pm35	1.037	1.052	0.95	1.118	0.003	0.83
365	pm12-pm36	0.997	1.042	0.983	1.002	-0.004	0.992

366	pm12-pm37	1.02	0.997	1.009	1.013	0.001	0.644
367	pm13-pm14	0.858	0.797	0.848	0.851	0.081	0.063
368	pm13-pm15	0.989	1.034	0.987	0.872	-0.052	0.026
369	pm13-pm16	1.078	0.972	1.055	0.894	-0.016	0.359
370	pm13-pm17	0.951	0.994	1.09	0.876	-0.057	0.024
371	pm13-pm18	0.895	0.929	0.899	0.867	0.03	0.247
372	pm13-pm19	0.924	0.894	0.941	0.851	0.006	0.58
373	pm13-pm20	0.919	0.913	0.897	0.835	0.017	0.393
374	pm13-pm21	1.411	0.938	1.66	1.049	-0.024	0.278
375	pm13-pm22	0.911	0.833	0.9	0.813	0.017	0.313
376	pm13-pm23	0.887	0.835	0.898	0.844	0.022	0.203
377	pm13-pm24	0.872	0.891	0.932	0.858	0.025	0.295
378	pm13-pm25	0.883	0.83	0.924	0.862	0.019	0.367
379	pm13-pm26	0.911	0.884	0.906	0.827	0.026	0.239
380	pm13-pm27	0.876	0.919	0.932	0.855	0.006	0.5
381	pm13-pm28	0.937	0.863	0.917	0.837	0.016	0.311
382	pm13-pm29	0.868	0.888	0.919	0.863	0.021	0.234
383	pm13-pm30	0.896	0.871	0.894	0.843	0.008	0.591
384	pm13-pm31	0.908	0.956	0.956	0.869	0.028	0.217
385	pm13-pm32	0.936	0.861	0.87	0.9	0.013	0.33
386	pm13-pm33	0.914	0.923	0.882	0.931	-0.028	0.27
387	pm13-pm34	0.877	0.897	0.826	0.872	0	0.881
388	pm13-pm35	0.899	0.865	0.824	0.947	0.001	0.687
389	pm13-pm36	0.898	0.833	0.882	0.818	0.001	0.997
390	pm13-pm37	0.917	0.843	0.877	0.812	0.007	0.545
391	pm14-pm15	0.999	1.096	1.054	0.904	-0.049	0.186
392	pm14-pm16	1.019	1.029	1.057	0.927	-0.049	0.2
393	pm14-pm17	1.024	0.984	1.111	0.923	-0.064	0.037
394	pm14-pm18	0.918	0.931	0.902	0.874	-0.002	0.726
395	pm14-pm19	0.912	0.947	0.927	0.891	-0.018	0.316

396	pm14-pm20	0.914	0.908	0.93	0.911	0.001	0.925
397	pm14-pm21	1.428	0.955	1.749	1.093	-0.022	0.24
398	pm14-pm22	0.923	0.927	0.953	0.868	0.03	0.25
399	pm14-pm23	0.908	0.943	0.902	0.872	0.022	0.207
400	pm14-pm24	0.916	0.872	0.925	0.889	-0.003	0.711
401	pm14-pm25	0.934	0.858	0.948	0.857	0.003	0.959
402	pm14-pm26	0.935	0.934	0.917	0.885	0.022	0.23
403	pm14-pm27	0.907	0.977	0.957	0.918	0.01	0.44
404	pm14-pm28	0.939	0.86	0.904	0.921	-0.007	0.468
405	pm14-pm29	0.927	0.9	0.895	0.874	-0.005	0.527
406	pm14-pm30	0.933	0.874	0.913	0.866	-0.012	0.303
407	pm14-pm31	0.939	0.946	0.996	0.927	0.018	0.305
408	pm14-pm32	0.912	0.92	0.939	0.965	0.015	0.362
409	pm14-pm33	0.934	0.968	0.925	0.983	-0.029	0.201
410	pm14-pm34	0.942	0.899	0.895	0.921	0.006	0.591
411	pm14-pm35	0.912	0.885	0.905	0.988	-0.001	0.874
412	pm14-pm36	0.919	0.878	0.911	0.892	0.003	0.891
413	pm14-pm37	0.946	0.877	0.938	0.887	0.016	0.378
414	pm15-pm16	1.29	1.401	1.22	1.13	-0.061	0.029
415	pm15-pm17	1.246	1.402	1.281	1.094	-0.084	0.012
416	pm15-pm18	1.17	1.195	1.097	1.019	-0.052	0.034
417	pm15-pm19	1.132	1.289	1.168	1.095	-0.031	0.267
418	pm15-pm20	1.108	1.223	1.152	1.04	-0.048	0.166
419	pm15-pm21	1.705	1.3	2.1	1.267	0	0.837
420	pm15-pm22	1.092	1.245	1.118	1.041	-0.029	0.293
421	pm15-pm23	1.079	1.246	1.134	1.027	-0.028	0.203
422	pm15-pm24	1.114	1.244	1.1	1.014	-0.051	0.03
423	pm15-pm25	1.126	1.25	1.123	1.052	-0.023	0.239
424	pm15-pm26	1.103	1.179	1.132	1.059	-0.043	0.159
425	pm15-pm27	1.112	1.287	1.117	1.018	-0.061	0.043

426	pm15-pm28	1.1	1.234	1.139	1.044	-0.048	0.151
427	pm15-pm29	1.081	1.175	1.111	1.057	-0.063	0.021
428	pm15-pm30	1.145	1.217	1.123	1.086	-0.031	0.217
429	pm15-pm31	1.106	1.278	1.159	1.04	-0.053	0.038
430	pm15-pm32	1.101	1.256	1.091	1.094	-0.048	0.17
431	pm15-pm33	1.107	1.378	1.127	1.177	-0.049	0.166
432	pm15-pm34	1.139	1.238	1.039	1.084	-0.048	0.187
433	pm15-pm35	1.097	1.245	1.052	1.178	-0.045	0.172
434	pm15-pm36	1.113	1.235	1.136	1.007	-0.039	0.249
435	pm15-pm37	1.106	1.165	1.109	1.026	-0.059	0.025
436	pm16-pm17	1.485	1.516	1.531	1.374	0.143	0.007
437	pm16-pm18	1.17	1.206	1.162	1.054	-0.019	0.37
438	pm16-pm19	1.126	1.266	1.204	1.11	-0.02	0.319
439	pm16-pm20	1.173	1.178	1.131	1.066	-0.036	0.209
440	pm16-pm21	1.739	1.253	2.167	1.293	0.026	0.284
441	pm16-pm22	1.163	1.224	1.152	1.055	0.002	0.739
442	pm16-pm23	1.129	1.193	1.143	1.068	-0.01	0.445
443	pm16-pm24	1.113	1.211	1.183	1.092	-0.013	0.324
444	pm16-pm25	1.159	1.155	1.152	1.065	-0.022	0.213
445	pm16-pm26	1.173	1.176	1.122	1.071	-0.019	0.391
446	pm16-pm27	1.157	1.171	1.181	1.064	-0.045	0.166
447	pm16-pm28	1.157	1.211	1.134	1.089	-0.023	0.26
448	pm16-pm29	1.155	1.172	1.163	1.066	-0.024	0.284
449	pm16-pm30	1.157	1.135	1.146	1.063	-0.042	0.16
450	pm16-pm31	1.199	1.257	1.179	1.079	-0.014	0.349
451	pm16-pm32	1.14	1.149	1.163	1.159	-0.025	0.206
452	pm16-pm33	1.221	1.309	1.155	1.212	-0.016	0.311
453	pm16-pm34	1.164	1.209	1.117	1.124	-0.013	0.307
454	pm16-pm35	1.193	1.249	1.122	1.223	0.015	0.357
455	pm16-pm36	1.15	1.161	1.161	1.087	-0.016	0.366

456	pm16-pm37	1.168	1.16	1.16	1.059	-0.018	0.393
457	pm17-pm18	1.186	1.166	1.185	1.032	-0.043	0.178
458	pm17-pm19	1.137	1.241	1.212	1.12	-0.037	0.222
459	pm17-pm20	1.138	1.153	1.21	1.043	-0.055	0.038
460	pm17-pm21	1.596	1.34	2.23	1.273	0.004	0.836
461	pm17-pm22	1.149	1.221	1.189	1	-0.025	0.275
462	pm17-pm23	1.141	1.234	1.215	0.98	-0.019	0.31
463	pm17-pm24	1.166	1.188	1.199	1.028	-0.036	0.286
464	pm17-pm25	1.169	1.163	1.198	1.037	-0.031	0.222
465	pm17-pm26	1.102	1.199	1.177	1.042	-0.043	0.172
466	pm17-pm27	1.138	1.212	1.263	1.048	-0.042	0.186
467	pm17-pm28	1.182	1.202	1.189	1.053	-0.033	0.222
468	pm17-pm29	1.162	1.186	1.253	1.021	-0.026	0.204
469	pm17-pm30	1.16	1.156	1.194	1.06	-0.043	0.161
470	pm17-pm31	1.183	1.289	1.233	1.077	-0.016	0.348
471	pm17-pm32	1.138	1.155	1.202	1.094	-0.049	0.184
472	pm17-pm33	1.146	1.299	1.214	1.194	-0.045	0.15
473	pm17-pm34	1.168	1.19	1.13	1.096	-0.039	0.218
474	pm17-pm35	1.18	1.224	1.113	1.187	-0.024	0.232
475	pm17-pm36	1.154	1.153	1.227	1.056	-0.027	0.268
476	pm17-pm37	1.166	1.137	1.189	1.042	-0.039	0.276
477	pm18-pm19	1.038	1.035	1.017	1.04	-0.015	0.371
478	pm18-pm20	1.027	1.009	0.993	1.03	-0.009	0.537
479	pm18-pm21	1.531	1.136	1.817	1.237	-0.008	0.504
480	pm18-pm22	1.038	1.04	0.987	1.013	0.022	0.207
481	pm18-pm23	1.013	1.05	0.996	1.01	0.023	0.245
482	pm18-pm24	1.035	1.047	0.977	1.025	0.007	0.441
483	pm18-pm25	1.02	1.014	1.018	1.026	0.013	0.38
484	pm18-pm26	1.04	0.977	1.004	1.046	0.011	0.35
485	pm18-pm27	1.007	1.08	1.02	1.037	-0.004	0.623

486	pm18-pm28	1.05	1.037	1.008	1.042	0.012	0.358
487	pm18-pm29	1.019	1.047	0.984	0.993	-0.003	0.615
488	pm18-pm30	1.075	1.023	1.025	1.01	0.014	0.303
489	pm18-pm31	1.02	1.092	0.997	1.054	-0.003	0.712
490	pm18-pm32	1.026	1.055	0.999	1.099	0.016	0.345
491	pm18-pm33	1.027	1.152	1	1.185	0	0.893
492	pm18-pm34	1.047	1.031	0.915	1.1	0.005	0.92
493	pm18-pm35	1.069	1.08	0.939	1.165	0.03	0.298
494	pm18-pm36	1.037	1.007	1	1.045	0.015	0.359
495	pm18-pm37	1.035	1.018	0.988	1.032	0.012	0.322
496	pm19-pm20	0.988	1.099	1.02	1.028	-0.02	0.237
497	pm19-pm21	1.536	1.135	1.886	1.26	-0.014	0.395
498	pm19-pm22	0.993	1.04	1.014	1.022	-0.01	0.385
499	pm19-pm23	1.002	1.078	1.012	1.033	0.008	0.591
500	pm19-pm24	1.007	1.037	1.022	1.028	-0.02	0.256
501	pm19-pm25	1.005	1.056	1.024	1.051	-0.001	0.663
502	pm19-pm26	1.002	1.07	1.012	1.041	-0.004	0.615
503	pm19-pm27	1.002	1.117	1.051	1.086	-0.005	0.535
504	pm19-pm28	0.998	1.058	1.04	1.084	-0.007	0.559
505	pm19-pm29	1.001	1.085	1.037	1.05	0	0.802
506	pm19-pm30	1.005	1.057	1.027	1.038	-0.016	0.367
507	pm19-pm31	1.028	1.133	1.035	1.025	-0.018	0.339
508	pm19-pm32	1.025	1.033	1.031	1.116	-0.007	0.584
509	pm19-pm33	0.98	1.184	1.012	1.16	-0.036	0.26
510	pm19-pm34	0.991	1.059	0.929	1.102	-0.027	0.202
511	pm19-pm35	1.014	1.135	0.985	1.146	0.007	0.58
512	pm19-pm36	1.017	1.033	1.022	1.046	-0.007	0.541
513	pm19-pm37	0.984	1.068	1.044	1.003	-0.01	0.353
514	pm20-pm21	1.542	1.15	1.836	1.22	-0.008	0.451
515	pm20-pm22	1.039	1.116	0.992	1.021	0.038	0.286

516	pm20-pm23	1.036	1.085	1.005	1.019	0.036	0.231
517	pm20-pm24	1.03	1.07	1.017	0.995	0.008	0.586
518	pm20-pm25	1.02	1.054	1.018	1.039	0.021	0.273
519	pm20-pm26	1.033	1.067	0.993	1.004	0.012	0.339
520	pm20-pm27	1.048	1.019	1.013	1.025	-0.02	0.26
521	pm20-pm28	1.029	1.042	1.009	1.045	0.002	0.836
522	pm20-pm29	0.992	1.047	0.941	1.034	-0.017	0.367
523	pm20-pm30	1.06	1.054	0.995	1.024	0.008	0.428
524	pm20-pm31	1.023	1.128	1.058	1.022	0.007	0.585
525	pm20-pm32	1.015	1.052	1.004	1.079	0.003	0.636
526	pm20-pm33	1.05	1.108	1.012	1.135	-0.021	0.259
527	pm20-pm34	0.996	1.066	0.937	1.089	-0.002	0.825
528	pm20-pm35	1.005	1.041	0.952	1.145	-0.004	0.697
529	pm20-pm36	1.033	1.014	0.994	0.987	-0.006	0.574
530	pm20-pm37	0.999	1.023	0.984	1.021	-0.005	0.424
531	pm21-pm22	1.501	1.145	1.82	1.179	-0.007	0.499
532	pm21-pm23	1.534	1.158	1.845	1.179	0.014	0.369
533	pm21-pm24	1.578	1.157	1.798	1.19	-0.004	0.999
534	pm21-pm25	1.532	1.104	1.822	1.232	-0.004	0.912
535	pm21-pm26	1.524	1.103	1.828	1.199	-0.013	0.376
536	pm21-pm27	1.526	1.184	1.841	1.22	-0.018	0.388
537	pm21-pm28	1.54	1.138	1.846	1.258	0.002	0.775
538	pm21-pm29	1.556	1.101	1.777	1.172	-0.033	0.262
539	pm21-pm30	1.517	1.091	1.82	1.206	-0.03	0.256
540	pm21-pm31	1.531	1.145	1.846	1.214	-0.031	0.202
541	pm21-pm32	1.511	1.17	1.807	1.271	-0.009	0.559
542	pm21-pm33	1.541	1.205	1.88	1.385	-0.009	0.498
543	pm21-pm34	1.554	1.148	1.78	1.248	-0.006	0.405
544	pm21-pm35	1.523	1.201	1.752	1.361	0.006	0.522
545	pm21-pm36	1.499	1.104	1.833	1.189	-0.021	0.205

546	pm21-pm37	1.511	1.122	1.871	1.176	-0.006	0.558
547	pm22-pm23	0.996	1.037	1.011	0.993	0.035	0.216
548	pm22-pm24	0.968	1.071	1.002	0.992	0.014	0.377
549	pm22-pm25	0.985	1.016	1.002	1.008	0.017	0.382
550	pm22-pm26	1.001	1.05	1.004	0.984	0.024	0.226
551	pm22-pm27	1.033	1.107	1.002	1.03	0.023	0.222
552	pm22-pm28	1.027	1.045	1.024	1.039	0.031	0.209
553	pm22-pm29	0.99	1.063	1.008	0.991	0.019	0.327
554	pm22-pm30	0.987	1.092	0.978	0.974	0.009	0.48
555	pm22-pm31	1.041	1.116	1.009	0.974	0.011	0.328
556	pm22-pm32	1.001	1.062	1.013	1.052	0.023	0.228
557	pm22-pm33	1.012	1.169	1	1.142	0.01	0.518
558	pm22-pm34	0.977	1.044	0.922	1.048	0	0.618
559	pm22-pm35	1.02	1.08	0.965	1.098	0.028	0.234
560	pm22-pm36	1.01	1.023	0.979	0.976	0.01	0.492
561	pm22-pm37	0.973	1.03	0.969	1.01	0.01	0.382
562	pm23-pm24	1.033	1.127	0.981	0.99	0.042	0.159
563	pm23-pm25	0.992	1.058	0.996	1.036	0.038	0.211
564	pm23-pm26	1.026	1.053	0.993	1.02	0.04	0.167
565	pm23-pm27	1.033	1.134	1.031	1.004	0.034	0.248
566	pm23-pm28	1.018	1.058	0.969	1.031	0.02	0.209
567	pm23-pm29	1.003	1.036	0.981	0.98	0.009	0.458
568	pm23-pm30	0.999	1.059	0.972	0.978	0.007	0.523
569	pm23-pm31	1.025	1.168	1.012	1.019	0.036	0.292
570	pm23-pm32	0.992	1.092	0.971	1.079	0.028	0.233
571	pm23-pm33	1.021	1.185	0.973	1.138	0.012	0.324
572	pm23-pm34	0.971	1.096	0.965	1.006	0.015	0.348
573	pm23-pm35	1.033	1.077	0.914	1.099	0.021	0.295
574	pm23-pm36	0.995	1.056	0.978	1.005	0.025	0.267
575	pm23-pm37	0.972	1.073	1.03	1.005	0.038	0.236

576	pm24-pm25	0.991	1.039	0.986	0.991	0	0.973
577	pm24-pm26	0.982	1.034	1.014	0.997	0.004	0.667
578	pm24-pm27	0.985	1.082	1.053	0.989	-0.009	0.445
579	pm24-pm28	0.975	1.043	1.002	1.014	-0.01	0.325
580	pm24-pm29	0.965	1.073	1.013	0.987	-0.001	0.752
581	pm24-pm30	1.003	1.063	0.992	1.022	0.005	0.48
582	pm24-pm31	1.013	1.127	1.021	0.996	-0.001	0.687
583	pm24-pm32	0.988	1.059	1	1.073	0.005	0.533
584	pm24-pm33	1.023	1.169	0.993	1.171	0.002	0.928
585	pm24-pm34	1.017	1.082	0.932	1.065	0.009	0.465
586	pm24-pm35	1.024	1.102	0.927	1.106	0.01	0.336
587	pm24-pm36	0.987	1.018	0.988	0.997	-0.006	0.599
588	pm24-pm37	0.992	1	0.985	0.996	-0.009	0.505
589	pm25-pm26	0.989	1.002	0.982	0.989	-0.004	0.939
590	pm25-pm27	0.998	1.065	1.023	0.982	-0.011	0.339
591	pm25-pm28	1.002	1.042	1.006	1.032	0.01	0.55
592	pm25-pm29	1.019	1.047	1.008	0.985	0.012	0.307
593	pm25-pm30	1.022	1.04	1.03	1.003	0.017	0.351
594	pm25-pm31	1.019	1.125	1.002	0.988	0.001	0.803
595	pm25-pm32	1.001	1.046	1.002	1.045	0.007	0.511
596	pm25-pm33	0.998	1.1	1.017	1.145	-0.014	0.341
597	pm25-pm34	0.989	1.039	0.915	1.071	-0.003	0.962
598	pm25-pm35	1.03	1.095	0.944	1.147	0.033	0.201
599	pm25-pm36	0.989	0.987	1.001	1.01	0.002	0.629
600	pm25-pm37	0.984	0.973	0.987	1.007	-0.006	0.569
601	pm26-pm27	0.986	1.013	1.033	0.99	-0.023	0.253
602	pm26-pm28	1.009	0.992	1.029	1.039	0.007	0.413
603	pm26-pm29	0.994	1.089	1.011	0.993	0.019	0.323
604	pm26-pm30	1.02	1.027	1.008	1.003	0.008	0.436
605	pm26-pm31	1.016	1.093	1.028	1.009	0.004	0.919

606	pm26-pm32	0.987	1.025	0.974	1.061	-0.005	0.45
607	pm26-pm33	1.021	1.129	0.996	1.173	0	0.694
608	pm26-pm34	0.998	1.026	0.928	1.068	-0.002	0.957
609	pm26-pm35	1.01	1.015	0.962	1.141	0.011	0.376
610	pm26-pm36	1.005	0.993	0.982	0.99	-0.003	0.844
611	pm26-pm37	1.018	1.018	1.009	0.984	0.013	0.395
612	pm27-pm28	1.015	1.094	1.04	1.015	-0.004	0.937
613	pm27-pm29	1.002	1.085	1.023	1.024	-0.003	0.89
614	pm27-pm30	1.035	1.047	1.026	1.01	-0.011	0.36
615	pm27-pm31	1.051	1.194	1.057	1.027	0.016	0.313
616	pm27-pm32	1.025	1.128	1.006	1.095	0.013	0.331
617	pm27-pm33	1.017	1.16	1.048	1.183	-0.011	0.313
618	pm27-pm34	1.017	1.116	0.956	1.065	-0.002	0.745
619	pm27-pm35	1.006	1.086	0.979	1.118	-0.008	0.405
620	pm27-pm36	1.028	1.024	1.042	0.997	-0.006	0.524
621	pm27-pm37	1.021	1.056	1.043	1.022	0.007	0.516
622	pm28-pm29	1.026	1.038	0.998	1.027	0.004	0.741
623	pm28-pm30	1.042	1.078	0.999	1.061	0.022	0.289
624	pm28-pm31	1.047	1.106	1.027	1.031	0.004	0.885
625	pm28-pm32	1.025	1.064	1.007	1.093	0.014	0.381
626	pm28-pm33	1.018	1.201	0.96	1.197	-0.002	0.702
627	pm28-pm34	0.998	1.061	0.899	1.063	-0.017	0.399
628	pm28-pm35	1.031	1.069	0.954	1.111	0.004	0.773
629	pm28-pm36	0.995	1.035	1.018	1.031	0.008	0.431
630	pm28-pm37	1.015	1.047	1.018	1.011	0.012	0.381
631	pm29-pm30	1.033	1.02	1.015	1.042	0.013	0.338
632	pm29-pm31	1.046	1.158	1.06	1.015	0.03	0.263
633	pm29-pm32	0.99	1.051	1.003	1.066	0.003	0.806
634	pm29-pm33	1.018	1.149	1.022	1.173	0.003	0.622
635	pm29-pm34	1	1.073	0.904	1.081	0	0.69

636	pm29-pm35	1.05	1.039	0.96	1.156	0.022	0.285
637	pm29-pm36	0.993	1.056	0.988	0.985	0.002	0.68
638	pm29-pm37	0.973	1.033	0.962	0.995	-0.011	0.319
639	pm30-pm31	1.063	1.138	1.045	1.023	0.022	0.211
640	pm30-pm32	1.038	1.049	0.99	1.073	0.008	0.434
641	pm30-pm33	1.064	1.161	1.001	1.166	0.006	0.434
642	pm30-pm34	0.99	1.037	0.91	1.064	-0.018	0.399
643	pm30-pm35	1.048	1.049	0.937	1.175	0.019	0.383
644	pm30-pm36	1.026	1.022	1.004	1.008	0.007	0.585
645	pm30-pm37	1.024	0.99	1.011	1.024	0.006	0.517
646	pm31-pm32	1.038	1.171	1.005	1.061	0.014	0.35
647	pm31-pm33	1.061	1.174	1.03	1.193	-0.003	0.865
648	pm31-pm34	1.042	1.164	0.973	1.067	0.017	0.317
649	pm31-pm35	1.035	1.17	0.983	1.152	0.026	0.202
650	pm31-pm36	1.028	1.07	1.019	1.008	-0.002	0.734
651	pm31-pm37	1.049	1.123	1.04	1.004	0.022	0.295
652	pm32-pm33	1.014	1.181	1.015	1.218	0.005	0.508
653	pm32-pm34	1.016	1.078	0.931	1.129	0.009	0.427
654	pm32-pm35	1.071	1.104	0.915	1.19	0.026	0.24
655	pm32-pm36	1.015	1.034	0.973	1.049	0	0.921
656	pm32-pm37	0.988	1.037	0.97	1.057	-0.004	0.755
657	pm33-pm34	1.014	1.109	0.985	1.22	-0.009	0.5
658	pm33-pm35	1.046	1.187	0.91	1.288	0.001	0.668
659	pm33-pm36	0.987	1.121	1.003	1.17	-0.01	0.574
660	pm33-pm37	1.018	1.141	1.007	1.167	0.004	0.9
661	pm34-pm35	1.018	1.05	0.918	1.14	-0.002	0.704
662	pm34-pm36	0.988	1.031	0.942	1.069	0	0.998
663	pm34-pm37	0.99	1.039	0.93	1.044	-0.005	0.476
664	pm35-pm36	1.016	1.093	0.948	1.1	0.017	0.35
665	pm35-pm37	1.021	1.048	0.903	1.145	0.008	0.59

666	pm36-pm37	1.007	1.004	0.997	1.016	0.011	0.301

3d) Result of two-distribution perturbation analysis for hypertrophy when four different response function types were applied in a combined manner (combination of response functions)

.		1 million parameter sets		10 million parameter sets		100 million parameter sets	
No	Pair of perturbed parameter distributions	Synergistic effect	p-value	Synergistic effect	p-value	Synergistic effect	p-value
1	pm1-pm2	0	0.968	0.004	0.792	-0.012	0.324
2	pm1-pm3	0.032	0.29	0.019	0.346	0.003	0.69
3	pm1-pm4	0	0.606	0.009	0.573	0.004	0.952
4	pm1-pm5	0.025	0.257	0.02	0.329	0.016	0.346
5	pm1-pm6	-0.029	0.23	-0.012	0.365	-0.023	0.203
6	pm1-pm7	-0.032	0.245	-0.053	0.029	-0.032	0.216
7	pm1-pm8	0.016	0.33	0.024	0.25	0.008	0.476
8	pm1-pm9	0.035	0.274	0.016	0.397	-0.006	0.41
9	pm1-pm10	-0.379	<0.001	-0.399	<0.001	-0.417	<0.001
10	pm1-pm11	-0.02	0.266	-0.006	0.429	0.023	0.261
11	pm1-pm12	0.017	0.318	0.023	0.249	0.009	0.55
12	pm1-pm13	0.002	0.976	0.021	0.264	0.013	0.372
13	pm1-pm14	-0.037	0.248	-0.025	0.212	-0.012	0.307
14	pm1-pm15	-0.063	0.03	-0.085	0.013	-0.088	0.011
15	pm1-pm16	0.006	0.465	-0.001	0.832	-0.028	0.256
16	pm1-pm17	0.025	0.202	0.019	0.395	0.016	0.329
17	pm1-pm18	0.003	0.928	0.001	0.833	0.02	0.302
18	pm1-pm19	0.008	0.531	-0.011	0.311	-0.021	0.209

19	pm1-pm20	0	0.818	0.031	0.218	0.01	0.397
20	pm1-pm21	-0.402	<0.001	-0.408	<0.001	-0.377	<0.001
21	pm1-pm22	0.039	0.253	-0.003	0.692	0.01	0.476
22	pm1-pm23	-0.187	0.003	-0.169	0.001	-0.185	0.001
23	pm1-pm24	0	0.915	0.011	0.388	0.008	0.509
24	pm1-pm25	-0.006	0.463	0.033	0.206	0.037	0.281
25	pm1-pm26	-0.022	0.239	0.002	0.601	-0.005	0.663
26	pm1-pm27	-0.01	0.551	-0.003	0.968	0.016	0.385
27	pm1-pm28	0.007	0.559	0.018	0.335	-0.015	0.307
28	pm1-pm29	0.03	0.261	0.023	0.243	-0.002	0.902
29	pm1-pm30	0.008	0.495	0.016	0.332	0.038	0.223
30	pm1-pm31	0.007	0.492	-0.012	0.392	0.024	0.254
31	pm1-pm32	0.025	0.26	-0.015	0.39	-0.008	0.504
32	pm1-pm33	-0.009	0.577	0.016	0.369	0.027	0.264
33	pm1-pm34	-0.049	0.171	-0.033	0.212	-0.027	0.267
34	pm1-pm35	0.016	0.341	-0.02	0.205	-0.008	0.502
35	pm1-pm36	0.004	0.962	0.002	0.968	0.033	0.292
36	pm1-pm37	0.012	0.37	0.006	0.404	0.033	0.238
37	pm2-pm3	-0.003	0.697	-0.037	0.263	-0.002	0.724
38	pm2-pm4	-0.003	0.861	-0.017	0.381	-0.023	0.212
39	pm2-pm5	-0.005	0.986	-0.017	0.363	-0.011	0.322
40	pm2-pm6	0.022	0.202	0.015	0.305	-0.002	0.648
41	pm2-pm7	0.015	0.357	0.006	0.456	0.012	0.395
42	pm2-pm8	0	0.938	-0.027	0.238	-0.025	0.223
43	pm2-pm9	0.003	0.73	0.039	0.258	0.031	0.261
44	pm2-pm10	-0.427	<0.001	-0.389	<0.001	-0.427	<0.001
45	pm2-pm11	-0.017	0.365	-0.026	0.224	-0.027	0.255
46	pm2-pm12	0.019	0.353	0.01	0.307	-0.022	0.279
47	pm2-pm13	-0.03	0.214	-0.031	0.296	-0.017	0.307
48	pm2-pm14	-0.016	0.341	-0.037	0.237	-0.023	0.213

49	pm2-pm15	-0.119	0.009	-0.108	0.01	-0.095	0.014
50	pm2-pm16	-0.093	0.014	-0.097	0.02	-0.077	0.017
51	pm2-pm17	-0.01	0.36	0.001	0.632	-0.023	0.235
52	pm2-pm18	0.045	0.184	0.016	0.35	0.039	0.222
53	pm2-pm19	-0.003	0.825	-0.004	0.726	-0.016	0.376
54	pm2-pm20	0.008	0.477	-0.008	0.416	0.016	0.36
55	pm2-pm21	-0.327	<0.001	-0.366	<0.001	-0.342	<0.001
56	pm2-pm22	0.025	0.235	-0.002	0.783	0.022	0.233
57	pm2-pm23	0.007	0.451	0.018	0.363	0.003	0.796
58	pm2-pm24	0.006	0.476	-0.006	0.506	-0.026	0.227
59	pm2-pm25	0.006	0.544	0.002	0.851	-0.013	0.302
60	pm2-pm26	-0.007	0.576	0.013	0.324	0.028	0.212
61	pm2-pm27	-0.001	0.616	0.015	0.384	0.021	0.217
62	pm2-pm28	-0.023	0.257	-0.023	0.263	0.01	0.338
63	pm2-pm29	-0.001	0.77	-0.019	0.326	-0.003	0.768
64	pm2-pm30	0.003	0.743	0.022	0.239	-0.012	0.389
65	pm2-pm31	-0.466	<0.001	-0.48	<0.001	-0.473	<0.001
66	pm2-pm32	0.021	0.211	0.022	0.299	-0.011	0.387
67	pm2-pm33	-0.016	0.313	-0.001	0.897	0.012	0.314
68	pm2-pm34	-0.026	0.254	-0.012	0.39	-0.026	0.222
69	pm2-pm35	0.012	0.364	-0.004	0.953	-0.005	0.679
70	pm2-pm36	0.024	0.235	-0.012	0.335	0.008	0.546
71	pm2-pm37	0.005	0.492	0.013	0.384	0.024	0.256
72	pm3-pm4	0.015	0.317	0.003	0.817	-0.012	0.368
73	pm3-pm5	0.017	0.341	0.016	0.305	0.021	0.252
74	pm3-pm6	0.027	0.204	0.018	0.365	-0.009	0.504
75	pm3-pm7	0.03	0.245	0.046	0.168	0.001	0.825
76	pm3-pm8	0.012	0.368	0.006	0.442	-0.01	0.532
77	pm3-pm9	0.016	0.38	0.042	0.188	0.031	0.257
78	pm3-pm10	-0.394	<0.001	-0.394	<0.001	-0.37	<0.001

79	pm3-pm11	-0.02	0.304	-0.007	0.541	0.016	0.377
80	pm3-pm12	0.016	0.372	0.025	0.251	0.004	0.807
81	pm3-pm13	0.02	0.298	0.022	0.237	0.03	0.286
82	pm3-pm14	-0.025	0.268	-0.028	0.269	-0.026	0.237
83	pm3-pm15	-0.066	0.028	-0.063	0.044	-0.067	0.031
84	pm3-pm16	-0.029	0.29	-0.038	0.216	-0.065	0.032
85	pm3-pm17	0.005	0.618	0.007	0.416	-0.001	0.982
86	pm3-pm18	0	0.973	0.044	0.201	0	0.694
87	pm3-pm19	-0.018	0.328	0.004	0.946	-0.006	0.477
88	pm3-pm20	-0.008	0.419	0.022	0.268	0.033	0.296
89	pm3-pm21	-0.388	<0.001	-0.404	<0.001	-0.378	<0.001
90	pm3-pm22	-0.007	0.516	-0.027	0.269	-0.012	0.37
91	pm3-pm23	-0.01	0.546	0.009	0.414	0.034	0.225
92	pm3-pm24	-0.016	0.366	0.008	0.528	-0.019	0.302
93	pm3-pm25	0.002	0.736	-0.001	0.934	0.005	0.508
94	pm3-pm26	0.02	0.225	0.008	0.443	0.024	0.255
95	pm3-pm27	-0.011	0.332	-0.007	0.571	0.013	0.335
96	pm3-pm28	0.017	0.342	0.02	0.325	-0.007	0.586
97	pm3-pm29	0.02	0.342	0.02	0.355	0.02	0.347
98	pm3-pm30	0.005	0.433	0.018	0.332	0.032	0.273
99	pm3-pm31	0.015	0.378	0.05	0.17	0.048	0.18
100	pm3-pm32	-0.002	0.61	-0.002	0.744	-0.017	0.356
101	pm3-pm33	-0.009	0.507	-0.039	0.254	-0.03	0.284
102	pm3-pm34	-0.01	0.494	0.024	0.223	0.031	0.275
103	pm3-pm35	-0.478	<0.001	-0.443	<0.001	-0.472	<0.001
104	pm3-pm36	-0.009	0.566	0.003	0.973	-0.011	0.376
105	pm3-pm37	0.029	0.294	0.019	0.303	-0.004	0.903
106	pm4-pm5	0.008	0.462	0.012	0.331	0.008	0.579
107	pm4-pm6	-0.017	0.324	-0.016	0.386	-0.007	0.44
108	pm4-pm7	-0.007	0.559	-0.039	0.294	-0.012	0.371

109	pm4-pm8	-0.032	0.209	-0.019	0.388	-0.017	0.304
110	pm4-pm9	0.032	0.285	0.049	0.189	0.03	0.205
111	pm4-pm10	-0.38	<0.001	-0.36	<0.001	-0.365	<0.001
112	pm4-pm11	0.029	0.262	0.011	0.318	0.031	0.293
113	pm4-pm12	0	0.996	0.003	0.869	-0.016	0.375
114	pm4-pm13	-0.047	0.174	-0.061	0.039	-0.029	0.24
115	pm4-pm14	-0.031	0.21	-0.023	0.258	-0.019	0.378
116	pm4-pm15	-0.097	0.015	-0.083	0.019	-0.085	0.018
117	pm4-pm16	-0.048	0.163	-0.04	0.189	-0.054	0.038
118	pm4-pm17	0.008	0.514	0.008	0.571	-0.013	0.304
119	pm4-pm18	0.015	0.324	-0.013	0.346	0.03	0.261
120	pm4-pm19	-0.042	0.194	-0.033	0.207	-0.037	0.266
121	pm4-pm20	0.009	0.528	-0.016	0.339	-0.005	0.53
122	pm4-pm21	-0.387	<0.001	-0.403	<0.001	-0.393	<0.001
123	pm4-pm22	0.018	0.326	0.033	0.21	0.045	0.167
124	pm4-pm23	0.048	0.169	0.009	0.582	0.016	0.38
125	pm4-pm24	-0.006	0.427	-0.007	0.52	0.011	0.361
126	pm4-pm25	0.011	0.323	-0.007	0.565	-0.001	0.979
127	pm4-pm26	-0.009	0.482	-0.005	0.532	-0.018	0.304
128	pm4-pm27	-0.025	0.286	0.018	0.384	-0.016	0.313
129	pm4-pm28	0.006	0.431	0.016	0.385	0.028	0.292
130	pm4-pm29	0	0.844	-0.009	0.466	-0.011	0.398
131	pm4-pm30	0.042	0.181	0.001	0.791	0.033	0.272
132	pm4-pm31	0.026	0.222	0.007	0.557	0.028	0.287
133	pm4-pm32	0.008	0.428	-0.022	0.237	0.012	0.396
134	pm4-pm33	0	0.839	-0.008	0.585	-0.011	0.338
135	pm4-pm34	-0.008	0.423	-0.022	0.292	-0.03	0.271
136	pm4-pm35	-0.018	0.308	-0.022	0.299	0.016	0.304
137	pm4-pm36	0.005	0.826	-0.007	0.432	0.001	0.662
138	pm4-pm37	0.009	0.408	0.017	0.355	0.001	0.984

139	pm5-pm6	-0.025	0.283	-0.012	0.356	0.015	0.317
140	pm5-pm7	-0.003	0.692	-0.003	0.635	-0.003	0.972
141	pm5-pm8	0.033	0.2	0.006	0.459	0.008	0.463
142	pm5-pm9	-0.083	0.017	-0.06	0.034	-0.071	0.013
143	pm5-pm10	-0.383	<0.001	-0.381	<0.001	-0.382	<0.001
144	pm5-pm11	0.018	0.374	0.027	0.281	0.019	0.307
145	pm5-pm12	0.023	0.274	0.017	0.4	0.007	0.554
146	pm5-pm13	0.011	0.314	-0.013	0.303	0.021	0.244
147	pm5-pm14	-0.029	0.21	0.003	0.953	0	0.827
148	pm5-pm15	-0.065	0.032	-0.029	0.253	-0.05	0.192
149	pm5-pm16	-0.1	0.006	-0.075	0.011	-0.079	0.015
150	pm5-pm17	-0.001	0.603	0.016	0.358	0.039	0.273
151	pm5-pm18	-0.059	0.038	-0.077	0.013	-0.059	0.034
152	pm5-pm19	0.027	0.239	-0.007	0.5	-0.007	0.594
153	pm5-pm20	-0.018	0.337	0.004	0.693	-0.016	0.375
154	pm5-pm21	-0.369	<0.001	-0.358	<0.001	-0.33	<0.001
155	pm5-pm22	-0.006	0.482	0.018	0.3	-0.001	0.795
156	pm5-pm23	0.005	0.509	0.024	0.214	0.02	0.32
157	pm5-pm24	-0.029	0.224	-0.022	0.249	0.002	0.983
158	pm5-pm25	-0.014	0.31	0.011	0.395	-0.02	0.256
159	pm5-pm26	-0.02	0.269	-0.01	0.44	0.014	0.319
160	pm5-pm27	0.014	0.371	-0.024	0.23	-0.021	0.229
161	pm5-pm28	0.005	0.843	0.017	0.379	0.001	0.976
162	pm5-pm29	0.002	0.692	-0.008	0.56	-0.002	0.614
163	pm5-pm30	-0.041	0.151	0.006	0.503	-0.021	0.274
164	pm5-pm31	0.034	0.21	0.032	0.214	-0.004	0.913
165	pm5-pm32	0.005	0.588	0.025	0.274	0.023	0.297
166	pm5-pm33	0.005	0.687	-0.019	0.37	0.004	0.661
167	pm5-pm34	-0.007	0.483	-0.022	0.266	-0.049	0.153
168	pm5-pm35	-0.004	0.885	0.005	0.711	0.016	0.397

169	pm5-pm36	-0.004	0.918	0	0.875	-0.024	0.211
170	pm5-pm37	-0.002	0.815	0.006	0.512	-0.012	0.38
171	pm6-pm7	-0.05	0.031	-0.018	0.335	-0.022	0.245
172	pm6-pm8	0.033	0.28	0.026	0.286	0.002	0.865
173	pm6-pm9	-0.443	<0.001	-0.444	<0.001	-0.461	<0.001
174	pm6-pm10	-0.393	<0.001	-0.415	<0.001	-0.416	<0.001
175	pm6-pm11	-0.021	0.256	0.007	0.518	0.001	0.794
176	pm6-pm12	0.006	0.579	0.015	0.388	-0.001	0.683
177	pm6-pm13	-0.06	0.045	-0.058	0.023	-0.049	0.173
178	pm6-pm14	0.008	0.445	-0.004	0.842	0.012	0.387
179	pm6-pm15	-0.081	0.013	-0.084	0.013	-0.043	0.198
180	pm6-pm16	-0.032	0.211	-0.058	0.045	-0.052	0.022
181	pm6-pm17	0.014	0.308	-0.009	0.559	-0.015	0.387
182	pm6-pm18	0.025	0.25	0.03	0.206	0.01	0.431
183	pm6-pm19	0.012	0.318	0.009	0.451	0.019	0.327
184	pm6-pm20	0.01	0.317	0.012	0.376	0.017	0.318
185	pm6-pm21	-0.373	<0.001	-0.368	<0.001	-0.354	<0.001
186	pm6-pm22	0.002	0.714	0.009	0.591	-0.02	0.293
187	pm6-pm23	0.011	0.321	-0.013	0.39	-0.01	0.51
188	pm6-pm24	0.01	0.38	-0.028	0.284	0.001	0.866
189	pm6-pm25	0.019	0.322	0.007	0.444	0.022	0.235
190	pm6-pm26	-0.017	0.398	-0.013	0.323	-0.015	0.323
191	pm6-pm27	0.037	0.264	0.013	0.321	0.025	0.276
192	pm6-pm28	-0.004	0.859	0.031	0.25	0.028	0.261
193	pm6-pm29	0.013	0.331	-0.024	0.239	0.017	0.394
194	pm6-pm30	0.028	0.282	-0.007	0.528	0.029	0.288
195	pm6-pm31	0.041	0.175	0.045	0.186	0.033	0.216
196	pm6-pm32	-0.015	0.329	0.021	0.275	-0.012	0.366
197	pm6-pm33	-0.019	0.31	-0.025	0.206	0.017	0.357
198	pm6-pm34	-0.018	0.356	-0.006	0.538	-0.019	0.394

199	pm6-pm35	-0.017	0.379	0.001	0.943	0.015	0.395
200	pm6-pm36	-0.021	0.261	-0.014	0.33	0.013	0.336
201	pm6-pm37	0.017	0.334	0.019	0.348	0.004	0.645
202	pm7-pm8	0.007	0.459	-0.017	0.376	-0.008	0.488
203	pm7-pm9	-0.425	<0.001	-0.432	<0.001	-0.462	<0.001
204	pm7-pm10	0.379	<0.001	0.361	<0.001	0.333	<0.001
205	pm7-pm11	0.004	0.749	-0.01	0.488	-0.004	0.967
206	pm7-pm12	-0.024	0.218	-0.035	0.2	-0.035	0.298
207	pm7-pm13	0.195	0.005	0.147	0.007	0.155	0.003
208	pm7-pm14	0.147	0.007	0.141	0.007	0.127	0.007
209	pm7-pm15	0.045	0.17	0.035	0.247	0.028	0.253
210	pm7-pm16	-0.115	0.007	-0.119	0.006	-0.091	0.016
211	pm7-pm17	-0.006	0.592	0.002	0.929	-0.006	0.597
212	pm7-pm18	-0.009	0.428	-0.024	0.281	-0.023	0.204
213	pm7-pm19	-0.066	0.037	-0.034	0.22	-0.063	0.036
214	pm7-pm20	-0.013	0.346	0.006	0.532	-0.013	0.39
215	pm7-pm21	0.405	<0.001	0.434	<0.001	0.359	<0.001
216	pm7-pm22	-0.252	<0.001	-0.292	<0.001	-0.293	<0.001
217	pm7-pm23	-0.248	<0.001	-0.253	<0.001	-0.23	<0.001
218	pm7-pm24	-0.182	0.002	-0.196	0.003	-0.202	<0.001
219	pm7-pm25	-0.25	<0.001	-0.275	<0.001	-0.27	<0.001
220	pm7-pm26	-0.314	<0.001	-0.314	<0.001	-0.274	<0.001
221	pm7-pm27	-0.222	<0.001	-0.236	<0.001	-0.213	<0.001
222	pm7-pm28	-0.2	0.003	-0.21	<0.001	-0.196	0.002
223	pm7-pm29	-0.207	<0.001	-0.216	<0.001	-0.207	<0.001
224	pm7-pm30	-0.249	<0.001	-0.222	<0.001	-0.254	<0.001
225	pm7-pm31	-0.177	0.002	-0.204	<0.001	-0.2	0.002
226	pm7-pm32	-0.21	<0.001	-0.19	0.004	-0.215	<0.001
227	pm7-pm33	-0.252	<0.001	-0.287	<0.001	-0.248	<0.001
228	pm7-pm34	-0.209	<0.001	-0.179	0.005	-0.182	0.002

229	pm7-pm35	-0.14	0.01	-0.144	0.009	-0.167	0.003
230	pm7-pm36	-0.19	0.004	-0.198	0.004	-0.203	<0.001
231	pm7-pm37	-0.257	<0.001	-0.24	<0.001	-0.263	<0.001
232	pm8-pm9	0.017	0.358	0.011	0.379	-0.008	0.421
233	pm8-pm10	-0.603	<0.001	-0.591	<0.001	-0.626	<0.001
234	pm8-pm11	0.003	0.836	0.008	0.483	0.006	0.436
235	pm8-pm12	-0.012	0.337	-0.003	0.814	0.027	0.247
236	pm8-pm13	-0.016	0.395	0.007	0.489	-0.018	0.366
237	pm8-pm14	-0.021	0.252	-0.014	0.326	-0.043	0.198
238	pm8-pm15	-0.076	0.016	-0.098	0.019	-0.061	0.029
239	pm8-pm16	-0.062	0.023	-0.038	0.28	-0.05	0.176
240	pm8-pm17	0.022	0.287	0.003	0.798	0.039	0.233
241	pm8-pm18	0.015	0.341	0.015	0.346	0.001	0.913
242	pm8-pm19	-0.021	0.257	0.007	0.472	-0.033	0.209
243	pm8-pm20	0.002	0.856	0.004	0.75	0.001	0.736
244	pm8-pm21	-0.576	<0.001	-0.536	<0.001	-0.562	<0.001
245	pm8-pm22	-0.463	<0.001	-0.463	<0.001	-0.443	<0.001
246	pm8-pm23	0.001	0.652	0.008	0.518	-0.017	0.394
247	pm8-pm24	0	0.808	0.024	0.255	-0.002	0.766
248	pm8-pm25	0.003	0.71	-0.016	0.329	0.016	0.337
249	pm8-pm26	-0.009	0.504	-0.018	0.33	-0.025	0.266
250	pm8-pm27	0.001	0.99	0.047	0.201	0.005	0.481
251	pm8-pm28	-0.014	0.352	0.008	0.422	0.012	0.382
252	pm8-pm29	0.019	0.399	0.009	0.569	0.025	0.275
253	pm8-pm30	0.026	0.282	0.005	0.502	0.022	0.219
254	pm8-pm31	-0.446	<0.001	-0.473	<0.001	-0.447	<0.001
255	pm8-pm32	0.024	0.222	-0.013	0.397	0.022	0.232
256	pm8-pm33	-0.036	0.232	-0.007	0.405	-0.029	0.265
257	pm8-pm34	-0.022	0.252	-0.004	0.764	0.012	0.396
258	pm8-pm35	-0.025	0.2	0.003	0.706	-0.002	1

259	pm8-pm36	-0.015	0.391	0.016	0.327	0.027	0.245
260	pm8-pm37	0.032	0.291	0.017	0.398	0.029	0.244
261	pm9-pm10	-0.605	<0.001	-0.612	<0.001	-0.585	<0.001
262	pm9-pm11	-0.449	<0.001	-0.442	<0.001	-0.459	<0.001
263	pm9-pm12	-0.453	<0.001	-0.46	<0.001	-0.473	<0.001
264	pm9-pm13	-0.001	0.745	-0.01	0.444	0.016	0.34
265	pm9-pm14	-0.012	0.312	-0.002	0.985	-0.011	0.352
266	pm9-pm15	-0.03	0.222	-0.055	0.033	-0.029	0.245
267	pm9-pm16	-0.044	0.167	-0.011	0.385	-0.025	0.263
268	pm9-pm17	0.039	0.234	0.025	0.221	0.028	0.295
269	pm9-pm18	-0.141	0.006	-0.181	0.003	-0.165	0.004
270	pm9-pm19	-0.177	0.004	-0.169	0.005	-0.146	0.007
271	pm9-pm20	0.013	0.381	0.011	0.301	0.017	0.32
272	pm9-pm21	-0.497	<0.001	-0.493	<0.001	-0.493	<0.001
273	pm9-pm22	0.014	0.304	0.016	0.318	0.043	0.192
274	pm9-pm23	-0.021	0.205	-0.013	0.327	-0.021	0.252
275	pm9-pm24	0.042	0.199	0.028	0.266	0.031	0.258
276	pm9-pm25	-0.463	<0.001	-0.471	<0.001	-0.472	<0.001
277	pm9-pm26	0.023	0.254	0.03	0.242	0.046	0.166
278	pm9-pm27	-0.459	<0.001	-0.466	<0.001	-0.462	<0.001
279	pm9-pm28	-0.076	0.011	-0.076	0.019	-0.06	0.034
280	pm9-pm29	0.006	0.562	-0.007	0.506	0.019	0.323
281	pm9-pm30	-0.444	<0.001	-0.43	<0.001	-0.435	<0.001
282	pm9-pm31	0.029	0.211	0.002	0.792	0.035	0.258
283	pm9-pm32	0.022	0.245	0.012	0.371	0.041	0.16
284	pm9-pm33	-0.017	0.378	0.013	0.311	-0.024	0.286
285	pm9-pm34	-0.042	0.196	-0.005	0.919	0	0.668
286	pm9-pm35	0.046	0.2	0.043	0.164	0.017	0.358
287	pm9-pm36	-0.479	<0.001	-0.473	<0.001	-0.45	<0.001
288	pm9-pm37	0.021	0.228	0.021	0.217	0.006	0.424

289	pm10-pm11	-0.365	<0.001	-0.353	<0.001	-0.362	<0.001
290	pm10-pm12	-0.392	<0.001	-0.391	<0.001	-0.372	<0.001
291	pm10-pm13	-0.442	<0.001	-0.435	<0.001	-0.429	<0.001
292	pm10-pm14	0.144	0.009	0.166	0.003	0.095	0.017
293	pm10-pm15	-0.424	<0.001	-0.422	<0.001	-0.447	<0.001
294	pm10-pm16	0.01	0.368	0.032	0.218	0.012	0.326
295	pm10-pm17	-0.324	<0.001	-0.332	<0.001	-0.337	<0.001
296	pm10-pm18	-0.421	<0.001	-0.444	<0.001	-0.415	<0.001
297	pm10-pm19	-0.418	<0.001	-0.434	<0.001	-0.416	<0.001
298	pm10-pm20	-0.38	<0.001	-0.386	<0.001	-0.404	<0.001
299	pm10-pm21	-0.213	<0.001	-0.213	<0.001	-0.232	<0.001
300	pm10-pm22	-0.178	0.003	-0.144	0.009	-0.148	0.005
301	pm10-pm23	-0.425	<0.001	-0.392	<0.001	-0.378	<0.001
302	pm10-pm24	-0.437	<0.001	-0.434	<0.001	-0.405	<0.001
303	pm10-pm25	-0.38	<0.001	-0.401	<0.001	-0.372	<0.001
304	pm10-pm26	-0.128	0.008	-0.151	0.001	-0.141	0.009
305	pm10-pm27	-0.405	<0.001	-0.381	<0.001	-0.382	<0.001
306	pm10-pm28	-0.387	<0.001	-0.378	<0.001	-0.365	<0.001
307	pm10-pm29	-0.408	<0.001	-0.438	<0.001	-0.402	<0.001
308	pm10-pm30	-0.433	<0.001	-0.437	<0.001	-0.463	<0.001
309	pm10-pm31	-0.378	<0.001	-0.375	<0.001	-0.406	<0.001
310	pm10-pm32	-0.415	<0.001	-0.44	<0.001	-0.394	<0.001
311	pm10-pm33	-0.42	<0.001	-0.393	<0.001	-0.402	<0.001
312	pm10-pm34	-0.381	<0.001	-0.412	<0.001	-0.399	<0.001
313	pm10-pm35	-0.394	<0.001	-0.394	<0.001	-0.428	<0.001
314	pm10-pm36	-0.44	<0.001	-0.444	<0.001	-0.455	<0.001
315	pm10-pm37	-0.39	<0.001	-0.405	<0.001	-0.434	<0.001
316	pm11-pm12	-0.02	0.241	0.014	0.346	0.015	0.358
317	pm11-pm13	0.015	0.376	-0.016	0.361	-0.005	0.664
318	pm11-pm14	-0.038	0.246	0.006	0.49	-0.04	0.232

319	pm11-pm15	-0.076	0.018	-0.073	0.02	-0.078	0.015
320	pm11-pm16	-0.053	0.036	-0.009	0.522	-0.042	0.158
321	pm11-pm17	0.029	0.218	0.015	0.386	-0.008	0.45
322	pm11-pm18	0.017	0.355	0.024	0.206	0.01	0.335
323	pm11-pm19	-0.003	0.85	0.014	0.345	-0.005	0.652
324	pm11-pm20	0.015	0.305	0.007	0.593	0.001	0.6
325	pm11-pm21	-0.387	<0.001	-0.368	<0.001	-0.371	<0.001
326	pm11-pm22	0.048	0.2	0.034	0.257	0.032	0.247
327	pm11-pm23	0.044	0.191	0	0.973	0.006	0.576
328	pm11-pm24	0.003	0.933	-0.021	0.214	-0.001	0.781
329	pm11-pm25	-0.015	0.37	0.015	0.305	-0.004	0.638
330	pm11-pm26	-0.011	0.393	-0.017	0.376	-0.013	0.359
331	pm11-pm27	0.024	0.284	0.028	0.258	0.007	0.534
332	pm11-pm28	0.027	0.216	0.001	0.852	-0.001	0.696
333	pm11-pm29	-0.022	0.3	-0.033	0.235	-0.007	0.459
334	pm11-pm30	0.023	0.214	0.008	0.521	0.022	0.259
335	pm11-pm31	-0.002	0.988	0.027	0.229	0.016	0.306
336	pm11-pm32	0.004	0.795	-0.007	0.556	-0.003	0.808
337	pm11-pm33	-0.019	0.308	0.017	0.3	0.025	0.233
338	pm11-pm34	-0.046	0.164	-0.031	0.28	-0.053	0.036
339	pm11-pm35	-0.003	0.651	0.019	0.331	0.032	0.233
340	pm11-pm36	0.016	0.309	-0.013	0.358	-0.015	0.316
341	pm11-pm37	-0.011	0.363	0.004	0.948	0.005	0.963
342	pm12-pm13	-0.017	0.348	-0.047	0.165	-0.036	0.201
343	pm12-pm14	0.019	0.368	0.001	0.736	0.014	0.367
344	pm12-pm15	-0.065	0.021	-0.08	0.012	-0.093	0.012
345	pm12-pm16	-0.065	0.028	-0.041	0.197	-0.054	0.024
346	pm12-pm17	-0.004	0.838	0.008	0.479	0.018	0.365
347	pm12-pm18	-0.004	0.675	0.002	0.651	-0.024	0.252
348	pm12-pm19	-0.008	0.53	0.002	0.709	0.022	0.298

349	pm12-pm20	0.023	0.272	0.02	0.295	0.021	0.265
350	pm12-pm21	-0.386	<0.001	-0.4	<0.001	-0.375	<0.001
351	pm12-pm22	0.029	0.231	0.019	0.364	-0.007	0.479
352	pm12-pm23	0.007	0.503	0.027	0.272	0.034	0.244
353	pm12-pm24	0.003	0.688	0.02	0.377	0.02	0.336
354	pm12-pm25	-0.01	0.524	0.002	0.683	0.021	0.247
355	pm12-pm26	-0.013	0.398	-0.01	0.419	0.009	0.592
356	pm12-pm27	0.019	0.34	0.008	0.405	0.018	0.305
357	pm12-pm28	0.009	0.465	0.022	0.234	-0.016	0.343
358	pm12-pm29	-0.004	0.976	0.03	0.259	0.021	0.224
359	pm12-pm30	-0.251	<0.001	-0.27	<0.001	-0.255	<0.001
360	pm12-pm31	-0.01	0.361	-0.005	0.446	-0.01	0.434
361	pm12-pm32	0.027	0.229	0.016	0.349	-0.002	0.895
362	pm12-pm33	0.001	0.689	0.002	0.662	-0.028	0.238
363	pm12-pm34	-0.031	0.274	-0.016	0.31	-0.046	0.161
364	pm12-pm35	-0.002	0.955	-0.007	0.475	-0.028	0.295
365	pm12-pm36	0.033	0.231	0.017	0.363	0.027	0.232
366	pm12-pm37	-0.012	0.357	0.017	0.346	0.021	0.24
367	pm13-pm14	0.022	0.154	0.019	0.252	0.0180	0.341
368	pm13-pm15	-0.101	0.01	-0.137	0.01	-0.129	0.009
369	pm13-pm16	-0.098	0.015	-0.05	0.184	-0.071	0.015
370	pm13-pm17	-0.119	0.007	-0.077	0.016	-0.082	0.017
371	pm13-pm18	-0.012	0.348	-0.016	0.376	-0.056	0.043
372	pm13-pm19	-0.009	0.478	0.016	0.399	-0.025	0.268
373	pm13-pm20	-0.048	0.2	-0.013	0.326	-0.009	0.594
374	pm13-pm21	-0.407	<0.001	-0.388	<0.001	-0.378	<0.001
375	pm13-pm22	0.007	0.572	-0.034	0.252	-0.023	0.292
376	pm13-pm23	-0.037	0.23	-0.046	0.167	-0.015	0.386
377	pm13-pm24	-0.011	0.313	-0.004	0.97	-0.047	0.167
378	pm13-pm25	-0.029	0.208	-0.028	0.284	-0.03	0.272

379	pm13-pm26	-0.024	0.214	-0.013	0.33	-0.037	0.251
380	pm13-pm27	-0.01	0.4	-0.048	0.159	-0.043	0.178
381	pm13-pm28	-0.026	0.283	-0.022	0.226	-0.017	0.309
382	pm13-pm29	-0.054	0.026	-0.071	0.012	-0.076	0.013
383	pm13-pm30	-0.034	0.253	-0.057	0.023	-0.036	0.246
384	pm13-pm31	-0.029	0.289	-0.032	0.243	-0.003	0.725
385	pm13-pm32	0.015	0.351	0.008	0.442	0.008	0.477
386	pm13-pm33	0	0.901	-0.019	0.333	0.005	0.729
387	pm13-pm34	-0.045	0.161	-0.042	0.2	-0.029	0.232
388	pm13-pm35	-0.034	0.298	-0.014	0.335	-0.032	0.23
389	pm13-pm36	-0.038	0.217	-0.003	0.747	-0.046	0.177
390	pm13-pm37	-0.032	0.259	-0.03	0.224	-0.016	0.328
391	pm14-pm15	-0.118	0.008	-0.08	0.011	-0.085	0.019
392	pm14-pm16	-0.129	0.006	-0.127	0.01	-0.138	0.005
393	pm14-pm17	-0.054	0.035	-0.022	0.276	-0.022	0.264
394	pm14-pm18	-0.026	0.263	-0.035	0.29	-0.032	0.278
395	pm14-pm19	-0.041	0.194	-0.012	0.342	-0.011	0.362
396	pm14-pm20	-0.014	0.358	-0.002	0.914	-0.003	0.7
397	pm14-pm21	-0.421	<0.001	-0.422	<0.001	-0.415	<0.001
398	pm14-pm22	-0.014	0.313	-0.014	0.333	0.007	0.478
399	pm14-pm23	-0.006	0.427	-0.01	0.325	-0.014	0.357
400	pm14-pm24	0.012	0.305	-0.021	0.266	0.002	0.693
401	pm14-pm25	0.013	0.388	0.022	0.24	0.024	0.299
402	pm14-pm26	-0.024	0.283	-0.032	0.287	-0.033	0.21
403	pm14-pm27	-0.016	0.314	-0.042	0.163	-0.027	0.233
404	pm14-pm28	0.004	0.622	0.005	0.785	0.028	0.238
405	pm14-pm29	-0.016	0.32	-0.023	0.245	-0.041	0.198
406	pm14-pm30	0.002	0.866	-0.017	0.368	-0.012	0.38
407	pm14-pm31	-0.019	0.308	-0.002	0.825	-0.036	0.208
408	pm14-pm32	-0.034	0.223	-0.018	0.306	-0.052	0.039

409	pm14-pm33	-0.002	0.926	-0.004	0.754	-0.017	0.323
410	pm14-pm34	0.009	0.56	-0.007	0.531	-0.025	0.292
411	pm14-pm35	-0.035	0.205	-0.024	0.286	-0.015	0.381
412	pm14-pm36	0.002	0.812	0.012	0.357	-0.01	0.599
413	pm14-pm37	0.037	0.241	0.012	0.34	0.006	0.535
414	pm15-pm16	-0.146	0.005	-0.143	0.009	-0.108	0.009
415	pm15-pm17	-0.084	0.012	-0.111	0.009	-0.084	0.014
416	pm15-pm18	-0.044	0.176	-0.044	0.167	-0.035	0.222
417	pm15-pm19	-0.042	0.191	-0.072	0.012	-0.054	0.023
418	pm15-pm20	-0.052	0.043	-0.067	0.024	-0.057	0.042
419	pm15-pm21	-0.381	<0.001	-0.365	<0.001	-0.357	<0.001
420	pm15-pm22	-0.065	0.021	-0.076	0.019	-0.053	0.022
421	pm15-pm23	-0.111	0.008	-0.085	0.014	-0.116	0.009
422	pm15-pm24	-0.044	0.188	-0.044	0.161	-0.087	0.01
423	pm15-pm25	-0.064	0.042	-0.053	0.041	-0.045	0.151
424	pm15-pm26	-0.038	0.242	-0.055	0.034	-0.075	0.013
425	pm15-pm27	-0.075	0.018	-0.04	0.157	-0.059	0.037
426	pm15-pm28	-0.087	0.017	-0.085	0.014	-0.111	0.007
427	pm15-pm29	-0.092	0.015	-0.103	0.006	-0.096	0.019
428	pm15-pm30	-0.034	0.218	-0.042	0.199	-0.026	0.263
429	pm15-pm31	-0.049	0.154	-0.071	0.019	-0.054	0.033
430	pm15-pm32	-0.102	0.006	-0.067	0.028	-0.061	0.034
431	pm15-pm33	-0.094	0.018	-0.067	0.043	-0.058	0.03
432	pm15-pm34	-0.064	0.045	-0.058	0.034	-0.045	0.173
433	pm15-pm35	-0.095	0.011	-0.082	0.012	-0.064	0.021
434	pm15-pm36	-0.04	0.163	-0.061	0.027	-0.063	0.02
435	pm15-pm37	-0.064	0.036	-0.054	0.038	-0.07	0.027
436	pm16-pm17	0.235	<0.001	0.256	<0.001	0.192	0.001
437	pm16-pm18	-0.048	0.161	-0.057	0.044	-0.048	0.186
438	pm16-pm19	-0.096	0.012	-0.068	0.038	-0.075	0.014

439	pm16-pm20	-0.098	0.019	-0.064	0.033	-0.075	0.016
440	pm16-pm21	-0.38	<0.001	-0.357	<0.001	-0.371	<0.001
441	pm16-pm22	-0.023	0.267	-0.036	0.217	-0.056	0.03
442	pm16-pm23	-0.083	0.01	-0.061	0.036	-0.081	0.015
443	pm16-pm24	-0.093	0.02	-0.082	0.01	-0.103	0.008
444	pm16-pm25	-0.065	0.035	-0.072	0.016	-0.028	0.255
445	pm16-pm26	-0.062	0.031	-0.033	0.281	-0.053	0.045
446	pm16-pm27	-0.062	0.043	-0.051	0.029	-0.081	0.015
447	pm16-pm28	-0.058	0.042	-0.068	0.021	-0.088	0.019
448	pm16-pm29	-0.072	0.018	-0.051	0.03	-0.07	0.015
449	pm16-pm30	-0.09	0.017	-0.091	0.015	-0.098	0.014
450	pm16-pm31	-0.024	0.257	-0.011	0.345	-0.011	0.38
451	pm16-pm32	-0.1	0.008	-0.072	0.011	-0.085	0.018
452	pm16-pm33	-0.021	0.221	-0.03	0.294	-0.017	0.333
453	pm16-pm34	-0.061	0.022	-0.077	0.013	-0.088	0.018
454	pm16-pm35	-0.052	0.042	-0.071	0.017	-0.056	0.028
455	pm16-pm36	-0.05	0.032	-0.095	0.015	-0.084	0.014
456	pm16-pm37	-0.05	0.18	-0.093	0.018	-0.08	0.018
457	pm17-pm18	0.041	0.164	0.03	0.226	0.028	0.258
458	pm17-pm19	-0.007	0.452	-0.001	0.991	-0.002	0.889
459	pm17-pm20	-0.046	0.163	-0.023	0.289	-0.001	0.918
460	pm17-pm21	-0.273	<0.001	-0.255	<0.001	-0.244	<0.001
461	pm17-pm22	0.016	0.366	0.022	0.223	0.031	0.287
462	pm17-pm23	-0.012	0.327	-0.018	0.32	0.007	0.43
463	pm17-pm24	-0.019	0.358	0.006	0.529	-0.016	0.356
464	pm17-pm25	0.038	0.263	0.033	0.234	0.015	0.39
465	pm17-pm26	-0.033	0.291	-0.035	0.271	-0.034	0.274
466	pm17-pm27	-0.043	0.2	-0.048	0.18	-0.029	0.22
467	pm17-pm28	-0.011	0.347	0.027	0.269	0.002	0.957
468	pm17-pm29	0.034	0.253	0.003	0.825	0.008	0.519

469	pm17-pm30	-0.025	0.259	-0.018	0.365	0.014	0.369
470	pm17-pm31	-0.025	0.282	-0.023	0.246	-0.013	0.328
471	pm17-pm32	-0.045	0.154	-0.041	0.176	-0.008	0.517
472	pm17-pm33	-0.012	0.381	0.01	0.429	-0.011	0.32
473	pm17-pm34	-0.018	0.309	-0.003	0.657	-0.031	0.297
474	pm17-pm35	0.026	0.227	0.02	0.318	0.026	0.26
475	pm17-pm36	-0.036	0.209	-0.037	0.292	0.007	0.504
476	pm17-pm37	0.026	0.245	0.014	0.35	0.001	0.683
477	pm18-pm19	0.035	0.239	0.004	0.969	0.021	0.226
478	pm18-pm20	0	0.803	0.005	0.438	-0.004	0.934
479	pm18-pm21	-0.353	<0.001	-0.379	<0.001	-0.354	<0.001
480	pm18-pm22	-0.443	<0.001	-0.465	<0.001	-0.462	<0.001
481	pm18-pm23	0.04	0.163	0.032	0.231	0.031	0.26
482	pm18-pm24	-0.473	<0.001	-0.49	<0.001	-0.454	<0.001
483	pm18-pm25	0.045	0.196	0.022	0.21	0.028	0.209
484	pm18-pm26	0.033	0.268	0.026	0.294	0.016	0.362
485	pm18-pm27	0.026	0.292	0.022	0.223	0.03	0.207
486	pm18-pm28	0.028	0.225	0.01	0.516	0.006	0.559
487	pm18-pm29	0	0.678	0.022	0.204	0.034	0.217
488	pm18-pm30	-0.444	<0.001	-0.453	<0.001	-0.438	<0.001
489	pm18-pm31	0.013	0.343	0	0.913	0.036	0.291
490	pm18-pm32	0.026	0.288	0.019	0.39	0.019	0.317
491	pm18-pm33	0.018	0.312	-0.011	0.379	0.02	0.4
492	pm18-pm34	0.017	0.341	0.022	0.295	0.031	0.225
493	pm18-pm35	-0.484	<0.001	-0.487	<0.001	-0.46	<0.001
494	pm18-pm36	0.014	0.345	0.034	0.22	0.04	0.194
495	pm18-pm37	0.031	0.254	0.02	0.208	0.032	0.283
496	pm19-pm20	-0.027	0.272	-0.017	0.359	-0.038	0.211
497	pm19-pm21	-0.362	<0.001	-0.375	<0.001	-0.346	<0.001
498	pm19-pm22	0.03	0.249	0.043	0.193	0.033	0.299

499	pm19-pm23	0.018	0.374	-0.015	0.378	0	0.669
500	pm19-pm24	0	0.865	-0.008	0.509	0.019	0.331
501	pm19-pm25	-0.001	0.66	0.015	0.384	0.011	0.331
502	pm19-pm26	0.002	0.618	0.004	0.873	0.022	0.296
503	pm19-pm27	0.017	0.36	0.021	0.233	0.012	0.359
504	pm19-pm28	0.002	0.791	0.014	0.33	0.014	0.399
505	pm19-pm29	-0.019	0.355	-0.006	0.434	0.001	0.605
506	pm19-pm30	-0.013	0.312	-0.027	0.221	-0.027	0.249
507	pm19-pm31	-0.012	0.314	0.002	0.756	-0.009	0.449
508	pm19-pm32	0.009	0.436	-0.011	0.304	-0.011	0.398
509	pm19-pm33	-0.01	0.346	-0.032	0.262	0.007	0.456
510	pm19-pm34	-0.551	<0.001	-0.536	<0.001	-0.552	<0.001
511	pm19-pm35	0.021	0.263	0.011	0.328	0.024	0.26
512	pm19-pm36	0.007	0.441	0.006	0.474	-0.019	0.365
513	pm19-pm37	-0.003	0.704	0.023	0.264	0.013	0.353
514	pm20-pm21	-0.349	<0.001	-0.355	<0.001	-0.385	<0.001
515	pm20-pm22	0.024	0.234	0.025	0.212	0.005	0.586
516	pm20-pm23	0.014	0.345	0.035	0.299	0.04	0.219
517	pm20-pm24	0.012	0.348	0.014	0.397	0.022	0.288
518	pm20-pm25	0.02	0.22	0.015	0.368	-0.008	0.415
519	pm20-pm26	0.031	0.274	-0.003	0.674	0.038	0.221
520	pm20-pm27	0.022	0.231	-0.003	0.669	0.033	0.238
521	pm20-pm28	0.012	0.361	0.024	0.295	-0.008	0.503
522	pm20-pm29	0.004	0.739	-0.044	0.178	-0.013	0.324
523	pm20-pm30	-0.057	0.027	-0.092	0.018	-0.071	0.017
524	pm20-pm31	0.008	0.422	0.031	0.277	0.026	0.292
525	pm20-pm32	0.004	0.663	-0.009	0.466	-0.006	0.535
526	pm20-pm33	0.037	0.259	0.013	0.375	0.007	0.427
527	pm20-pm34	-0.034	0.28	-0.01	0.321	-0.027	0.287
528	pm20-pm35	-0.016	0.353	-0.026	0.293	-0.022	0.22

529	pm20-pm36	-0.009	0.487	0.017	0.379	0.01	0.367
530	pm20-pm37	-0.005	0.75	-0.01	0.302	0.006	0.528
531	pm21-pm22	-0.067	0.045	-0.067	0.033	-0.086	0.015
532	pm21-pm23	-0.056	0.038	-0.015	0.35	-0.031	0.248
533	pm21-pm24	-0.041	0.193	-0.053	0.034	-0.022	0.213
534	pm21-pm25	-0.046	0.168	-0.031	0.288	-0.043	0.161
535	pm21-pm26	-0.079	0.011	-0.058	0.027	-0.086	0.016
536	pm21-pm27	-0.062	0.039	-0.091	0.017	-0.059	0.045
537	pm21-pm28	-0.032	0.293	-0.056	0.044	-0.042	0.161
538	pm21-pm29	-0.065	0.04	-0.062	0.025	-0.06	0.029
539	pm21-pm30	-0.077	0.012	-0.099	0.011	-0.086	0.019
540	pm21-pm31	-0.062	0.026	-0.084	0.016	-0.08	0.014
541	pm21-pm32	-0.062	0.039	-0.095	0.013	-0.083	0.015
542	pm21-pm33	-0.057	0.03	-0.088	0.018	-0.073	0.013
543	pm21-pm34	-0.039	0.268	-0.075	0.013	-0.077	0.012
544	pm21-pm35	-0.073	0.016	-0.078	0.02	-0.114	0.007
545	pm21-pm36	-0.098	0.013	-0.083	0.017	-0.072	0.019
546	pm21-pm37	-0.074	0.018	-0.097	0.018	-0.056	0.038
547	pm22-pm23	0.004	0.689	0.015	0.379	-0.001	0.895
548	pm22-pm24	-0.008	0.449	-0.02	0.212	0.002	0.932
549	pm22-pm25	-0.012	0.359	0.018	0.365	-0.01	0.525
550	pm22-pm26	0.016	0.38	0.047	0.181	0.032	0.222
551	pm22-pm27	0.045	0.173	0.037	0.208	0.018	0.328
552	pm22-pm28	0.019	0.324	0.017	0.324	0.021	0.21
553	pm22-pm29	-0.019	0.32	-0.001	0.781	-0.019	0.301
554	pm22-pm30	-0.001	0.775	-0.008	0.411	-0.006	0.582
555	pm22-pm31	-0.453	<0.001	-0.467	<0.001	-0.435	<0.001
556	pm22-pm32	-0.015	0.351	-0.013	0.315	-0.017	0.371
557	pm22-pm33	0.007	0.413	0.006	0.509	0.021	0.239
558	pm22-pm34	0.011	0.33	-0.01	0.397	0.004	0.947

559	pm22-pm35	0.028	0.257	0.024	0.249	-0.001	0.932
560	pm22-pm36	-0.004	0.843	0.021	0.217	0.005	0.693
561	pm22-pm37	-0.022	0.249	0.012	0.3	-0.023	0.265
562	pm23-pm24	-0.481	<0.001	-0.451	<0.001	-0.473	<0.001
563	pm23-pm25	-0.02	0.276	-0.023	0.281	-0.018	0.325
564	pm23-pm26	0.016	0.313	0.024	0.223	0.02	0.372
565	pm23-pm27	0.007	0.404	0.017	0.302	0.025	0.205
566	pm23-pm28	0.01	0.366	-0.005	0.446	0.035	0.235
567	pm23-pm29	0.002	0.672	0.027	0.291	0.016	0.353
568	pm23-pm30	0.029	0.225	0.034	0.219	0.019	0.335
569	pm23-pm31	0.022	0.277	0.025	0.21	0.017	0.342
570	pm23-pm32	0.008	0.51	-0.011	0.32	0.004	0.819
571	pm23-pm33	-0.003	0.896	-0.006	0.521	0.038	0.282
572	pm23-pm34	-0.024	0.21	-0.035	0.233	-0.017	0.319
573	pm23-pm35	0.047	0.199	0.013	0.336	0.027	0.276
574	pm23-pm36	-0.011	0.377	0.026	0.265	-0.01	0.35
575	pm23-pm37	0.007	0.506	0.011	0.349	0.004	0.736
576	pm24-pm25	0.01	0.431	-0.01	0.56	0.002	0.984
577	pm24-pm26	-0.015	0.385	0.015	0.304	-0.02	0.34
578	pm24-pm27	-0.019	0.337	-0.023	0.234	-0.012	0.347
579	pm24-pm28	-0.025	0.247	-0.026	0.299	-0.021	0.227
580	pm24-pm29	0.004	0.92	-0.006	0.405	-0.011	0.389
581	pm24-pm30	0.034	0.287	0.025	0.202	0.004	0.982
582	pm24-pm31	0.035	0.252	0.037	0.222	0.031	0.248
583	pm24-pm32	-0.027	0.27	0.01	0.492	0.019	0.335
584	pm24-pm33	0.001	0.78	0.007	0.422	0.027	0.284
585	pm24-pm34	-0.02	0.351	0.024	0.21	-0.001	0.699
586	pm24-pm35	0.024	0.293	0.017	0.304	0.023	0.288
587	pm24-pm36	-0.001	0.879	-0.029	0.215	0.004	0.907
588	pm24-pm37	0.014	0.383	-0.007	0.454	0.007	0.577

589	pm25-pm26	0.022	0.201	0.021	0.288	0.018	0.376
590	pm25-pm27	-0.019	0.373	-0.031	0.225	0.001	0.682
591	pm25-pm28	-0.015	0.339	-0.029	0.255	0	0.679
592	pm25-pm29	-0.016	0.372	0.002	0.9	0.003	0.929
593	pm25-pm30	0.026	0.215	0.033	0.252	0.019	0.354
594	pm25-pm31	0.021	0.224	-0.01	0.489	0.021	0.229
595	pm25-pm32	0.008	0.547	-0.007	0.465	0.021	0.221
596	pm25-pm33	-0.013	0.371	0.001	0.936	0.032	0.261
597	pm25-pm34	-0.026	0.288	-0.012	0.305	-0.029	0.264
598	pm25-pm35	0.015	0.388	-0.008	0.523	0.01	0.329
599	pm25-pm36	-0.011	0.365	-0.019	0.382	-0.017	0.376
600	pm25-pm37	-0.01	0.329	0.004	0.877	-0.017	0.339
601	pm26-pm27	0.011	0.367	0	0.777	-0.005	0.905
602	pm26-pm28	0.013	0.354	-0.008	0.469	0.028	0.241
603	pm26-pm29	0.03	0.262	0.014	0.306	-0.013	0.354
604	pm26-pm30	0.026	0.207	0.019	0.342	0.03	0.216
605	pm26-pm31	0.014	0.31	0.043	0.15	0.004	0.895
606	pm26-pm32	0.006	0.487	-0.003	0.955	-0.015	0.361
607	pm26-pm33	0.007	0.494	0.033	0.208	0.042	0.167
608	pm26-pm34	-0.004	0.751	0.002	0.619	0.018	0.361
609	pm26-pm35	0.005	0.533	0.033	0.3	0.011	0.352
610	pm26-pm36	-0.004	0.945	-0.001	0.987	-0.01	0.547
611	pm26-pm37	-0.019	0.396	-0.014	0.374	0.009	0.545
612	pm27-pm28	0.002	0.867	0.018	0.342	0.014	0.361
613	pm27-pm29	-0.008	0.587	-0.031	0.253	-0.041	0.171
614	pm27-pm30	0.018	0.341	0.006	0.55	0.011	0.365
615	pm27-pm31	-0.026	0.216	-0.041	0.191	-0.04	0.192
616	pm27-pm32	-0.012	0.342	-0.016	0.39	-0.024	0.272
617	pm27-pm33	0.011	0.363	0.012	0.324	0.012	0.386
618	pm27-pm34	0.026	0.246	0.027	0.231	0.02	0.391

619	pm27-pm35	-0.007	0.548	0.007	0.575	0.017	0.387
620	pm27-pm36	-0.002	0.893	0.013	0.365	0.01	0.461
621	pm27-pm37	-0.025	0.247	0.004	0.933	-0.002	0.618
622	pm28-pm29	0.018	0.377	-0.023	0.223	0.02	0.265
623	pm28-pm30	0.028	0.261	0.02	0.288	0	0.944
624	pm28-pm31	0.037	0.221	0.029	0.296	0.039	0.219
625	pm28-pm32	0.026	0.267	-0.015	0.319	0.025	0.254
626	pm28-pm33	0.02	0.237	-0.006	0.499	0.006	0.463
627	pm28-pm34	-0.011	0.315	-0.036	0.291	-0.045	0.174
628	pm28-pm35	0.016	0.4	0.025	0.286	0.011	0.365
629	pm28-pm36	0.001	0.771	-0.019	0.3	0.01	0.52
630	pm28-pm37	0.032	0.26	0.027	0.296	0.028	0.207
631	pm29-pm30	0.027	0.234	0.005	0.413	0.008	0.548
632	pm29-pm31	0.023	0.284	0.033	0.298	-0.005	0.535
633	pm29-pm32	0.008	0.482	0.01	0.45	0.015	0.335
634	pm29-pm33	-0.001	0.854	0.014	0.386	-0.003	0.967
635	pm29-pm34	-0.046	0.175	-0.029	0.291	-0.042	0.194
636	pm29-pm35	0.017	0.336	0.045	0.156	0.023	0.202
637	pm29-pm36	0.008	0.568	-0.001	0.837	0.025	0.202
638	pm29-pm37	-0.025	0.207	0.011	0.361	0.014	0.301
639	pm30-pm31	0.006	0.465	0.002	0.804	-0.029	0.279
640	pm30-pm32	0.011	0.377	-0.003	0.686	0.019	0.362
641	pm30-pm33	-0.042	0.159	-0.013	0.335	-0.023	0.229
642	pm30-pm34	-0.009	0.56	-0.032	0.29	-0.038	0.203
643	pm30-pm35	0.007	0.478	0.047	0.155	0.012	0.34
644	pm30-pm36	0.027	0.208	0.003	0.816	0.001	0.942
645	pm30-pm37	0.017	0.379	-0.013	0.349	-0.012	0.37
646	pm31-pm32	-0.004	0.752	0.009	0.465	-0.001	0.691
647	pm31-pm33	0.016	0.304	0.002	0.601	0.023	0.216
648	pm31-pm34	0.003	0.781	0.01	0.361	-0.001	0.923

649	pm31-pm35	0.048	0.166	0.009	0.539	0.036	0.2
650	pm31-pm36	0.024	0.212	-0.01	0.444	-0.002	0.691
651	pm31-pm37	-0.171	0.001	-0.159	0.003	-0.151	0.005
652	pm32-pm33	0.033	0.227	0.012	0.397	0.006	0.432
653	pm32-pm34	-0.003	0.873	-0.025	0.206	-0.006	0.442
654	pm32-pm35	-0.134	0.009	-0.164	0.004	-0.182	0.001
655	pm32-pm36	0.009	0.522	0.014	0.379	-0.01	0.595
656	pm32-pm37	0	0.766	-0.015	0.332	0.001	0.606
657	pm33-pm34	0.003	0.747	-0.002	0.944	0.016	0.312
658	pm33-pm35	0.011	0.305	0.033	0.215	0.029	0.225
659	pm33-pm36	-0.03	0.296	-0.002	0.677	-0.038	0.223
660	pm33-pm37	-0.008	0.482	0.031	0.294	-0.001	0.886
661	pm34-pm35	0.006	0.59	-0.02	0.364	-0.02	0.355
662	pm34-pm36	0.008	0.439	-0.027	0.29	-0.019	0.315
663	pm34-pm37	-0.012	0.363	0.006	0.431	-0.006	0.463
664	pm35-pm36	-0.002	0.839	-0.014	0.377	0.029	0.249
665	pm35-pm37	0.017	0.393	0.025	0.244	0.013	0.39
666	pm36-pm37	-0.021	0.223	-0.01	0.355	0.011	0.394

References for Supplementary Information

1. Tyson JJ, Novak B. Functional motifs in biochemical reaction networks. Annual review of physical chemistry. 2010;61:219-40.
2. Alon U. An introduction to systems biology: design principles of biological circuits: CRC press; 2006.
3. Won JK, Yang HW, Shin SY, Lee JH, Heo WD, Cho KH. The crossregulation between ERK and PI3K signaling pathways determines the tumoricidal efficacy of MEK inhibitor. J Mol Cell Biol. 2012;4(3):153-63.
